# A comprehensive microsatellite landscape of human Y-DNA at kilobase resolution

**DOI:** 10.1186/s12864-021-07389-5

**Published:** 2021-01-22

**Authors:** Douyue Li, Saichao Pan, Hongxi Zhang, Yongzhuo Fu, Zhuli Peng, Liang Zhang, Shan Peng, Fei Xu, Hanrou Huang, Ruixue Shi, Heping Zheng, Yousong Peng, Zhongyang Tan

**Affiliations:** 1 Bioinformatics Center, College of Biology, Hunan University, Changsha, 410082 China; 2 Department of Mathematics, Wilfrid Laurier University, Waterloo, Ontario N2L 3C5 Canada

**Keywords:** Simple sequence repeat, Human Y-DNA, SSR landscape, 1 Kbp differential unit, SSR density peak, Extremely low SSR density region

## Abstract

**Background:**

Though interest in human simple sequence repeats (SSRs) is increasing, little is known about the exact distributional features of numerous SSRs in human Y-DNA at chromosomal level. Herein, totally 540 maps were established, which could clearly display SSR landscape in every bin of 1 k base pairs (Kbp) along the sequenced part of human reference Y-DNA (NC_000024.10), by our developed differential method for improving the existing method to reveal SSR distributional characteristics in large genomic sequences.

**Results:**

The maps show that SSRs accumulate significantly with forming density peaks in at least 2040 bins of 1 Kbp, which involve different coding, noncoding and intergenic regions of the Y-DNA, and 10 especially high density peaks were reported to associate with biological significances, suggesting that the other hundreds of especially high density peaks might also be biologically significant and worth further analyzing. In contrast, the maps also show that SSRs are extremely sparse in at least 207 bins of 1 Kbp, including many noncoding and intergenic regions of the Y-DNA, which is inconsistent with the widely accepted view that SSRs are mostly rich in these regions, and these sparse distributions are possibly due to powerfully regional selection. Additionally, many regions harbor SSR clusters with same or similar motif in the Y-DNA.

**Conclusions:**

These 540 maps may provide the important information of clearly position-related SSR distributional features along the human reference Y-DNA for better understanding the genome structures of the Y-DNA. This study may contribute to further exploring the biological significance and distribution law of the huge numbers of SSRs in human Y-DNA.

**Supplementary Information:**

The online version contains supplementary material available at 10.1186/s12864-021-07389-5.

## Background

Simple sequence repeats (SSRs/microsatellites) are ubiquitous in eukaryotic, prokaryotic, and also viral genomes with repeat-units of 1–6 bp/nt [1–5]. SSRs have been reported to nonrandomly occur in genomes and associate with different biological significances, which have been gradually recognized as important elements [2, 6, 7]. They have been discovered in both coding and noncoding regions with important roles in modifying morphological features [8], regulating gene expression [9], protecting sequence structures [7], acting as essential boundaries [10], modulating RNA structure and function [11], creating available variants to survive in the host [12] and contributing to genomic evolution [13]. Lots of medical studies have revealed abnormal SSRs in different genomic positions related with more than 40 genetic diseases like fragile X syndrome, Huntington’s disease, Friedreich’s ataxia and spinocerebellar ataxias type 8, or in many cancers like colorectal cancer, endometrial cancer, gastrointestinal cancer and breast cancer [14–16].

SSRs have been reported to constitute ~ 12% of Japanese pufferfish genome, 15% of rabbit genome, 10% of primate genome and so on [17]; and it has been estimated that SSRs represent over 1 million sites covering 3% of human genome [1, 4]. Though numerous studies and interests were paid to the SSRs in human genome in past decades [1, 4, 15, 17], the clarified SSRs still involve only very few human genomic positions [2]. Rough SSR distributional features have been investigated in human genome [18, 19], and the Genome Browser also provides chance for surveying part of the elementary position of relatively longer SSRs in full human genome [20].

Human chromosome Y is unique with sex-determining genomic compositions and unusual evolutionary history [21, 22]. Human X and Y chromosomes originated from ordinary autosomes beginning at millions of years ago, and the Y chromosome specifically evolved with frequent gene decay and a lack of recombination, making it strikingly different from the X chromosome in size, structure and gene content [23, 24]. Owing to the gene decay and lacking recombination, Y chromosome formed a male-specific region (MSY) that comprise 95% of its length, and this region is flanked on both sides by pseudoautomosomal region (PAR), which can process mitotic recombination with chromosome X. Though human Y chromosome harbors a few genes, it is rich in repetitive and ampliconic elements, including SSRs [23–25]. The mutation rates of many SSRs are significantly high in this chromosome; Willems et al. predicted that the load of de novo SSR mutations is at least 75 mutations per generation in human Y chromosome [26] and Ballantyne et al. estimated that the mutation rates are from 3.78 × 10^− 4^ to 7.44 × 10^− 2^ per Y SSR marker they selected per generation [27]. And researches always prefer to work on these highly polymorphic SSRs in human Y-DNA, like DYS19 or called DYS394, whose sequence is (TAGA)_3_(TAGG)(TAGA)_7–15_ in Yp11.1 and mutation rate is 2.5 × 10^− 4^, as they can be widely applied in forensic investigation, paternity test, population study and evolutionary research [26, 28–33]. The investigations of SSR distributional features are limited to these several highly polymorphic sites, so it is necessary to reveal the exact distributional features of many thousands of SSRs in human Y-DNA at chromosomal level.

Here, we developed a differential calculating method for further exploring the exact distributional features of the SSRs with human reference Y-DNA (NC_000024.10). Hundreds of maps were established to clearly show SSR landscape in every 1 kilobase (Kbp) genomic region of human reference Y-DNA. These SSR landscapes revealed significant regional variation of SSR distributional features in this Y-DNA at the differential resolution of 1 Kbp. This study may provide an important guide for further exploring biological significance and distributional laws of numerous SSRs in human Y-DNA.

## Results

**Fig. 1:**
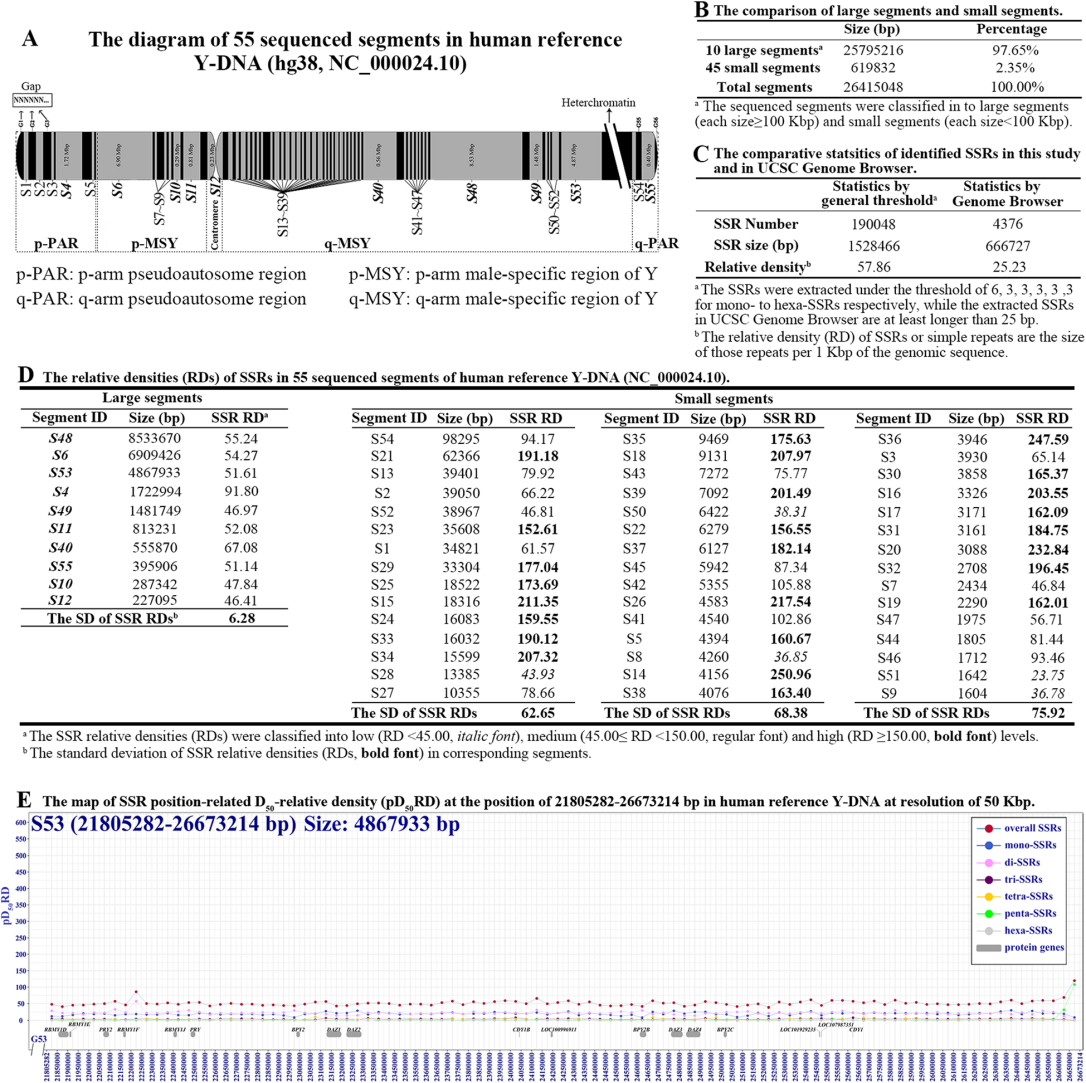
The densities of identified SSRs in the sequenced segments of reported human reference Y-DNA (NC_000024.10). **a** The diagram of 55 sequenced segments in human reference Y-DNA (NC_000024.10). **b** The comparison of sequenced segments and small segments. **c** The comparative statistics of identified SSRs in the study and in UCSC Genome Browser. **d** The relative densities (RDs) of SSRs in 55 sequenced segments in human reference Y-DNA (NC_000024.10). **e** The map of SSR position-related D_50_-relative density at the position of 21,805,282–26,673,214 bp in human reference Y-DNA at resolution of 50 Kbp

The exact distributional features of the SSRs were investigated in the reference sequence of human Y-DNA (NC_000024.10), and this well reviewed Y-DNA is still incompletely sequenced with 55 sequenced segments and 56 gaps (Fig. 1a and Table S1). The sizes of 55 sequenced segments are in range of 1604 ~ 8,533,670 bp, which can be grouped into large (≥100 Kbp) and small (< 100 Kbp) size segments; the large sequenced segments are totally 25,805,216 bp representing about 97.65% of the sequenced part of the reference Y-DNA, and the other 45 small segments represent only 2.35% of the sequenced part (Fig. 1b and Table S1). The total number and size of SSRs are 190,048 and 1,528,466 in this reference Y-DNA under the threshold of 6, 3, 3, 3, 3, 3 (Fig. 1c); and the threshold was widely applied to analyze SSRs in many reported studies [34, 35], which could extract much more SSRs than those in The UCSC (University of California, Santa Cruz) Genome Browser, where the total number of SSRs is only 4376 due to excluding the SSRs shorter than 25 bp by the default settings [20, 36].

The SSR distributions were widely studied with the statistics of relative density (RD) [18, 34, 37]. The average relative density of the SSRs is 57.86 in the total 55 sequenced segments, but the relative density is very different in every sequenced segment. The relative densities of SSRs vary a little from 46.41 to 91.80 with a standard deviation (SD) equaling to 6.28 in the 10 large segments; and that vary a lot from 23.75 to 250.96 with standard deviations more than 62.65 in the 45 small segments, the standard deviation of SSR relative densities was showed to increase obviously as the sizes of investigated segments decreasing (Fig. 1d). These data suggested that the result of SSR relative density is seriously influenced by the statistical segment size; it may not correctly reveal the true features of SSR distributions in these large segments, and the big size may have masked the true distribution features of SSRs in those large segments. As the small segments were showed a great SSR relative density variation and separately located in different parts of the human Y-DNA, the SSR relative density variation may be significantly related to the genome position. These analyses indicate that such relative density method is possibly very limited for analysis of SSRs in big sequence like human genome, and it is necessary to develop new approaches for investigating the exact distribution feature of SSRs in large genomic sequence.

**Fig. 2:**
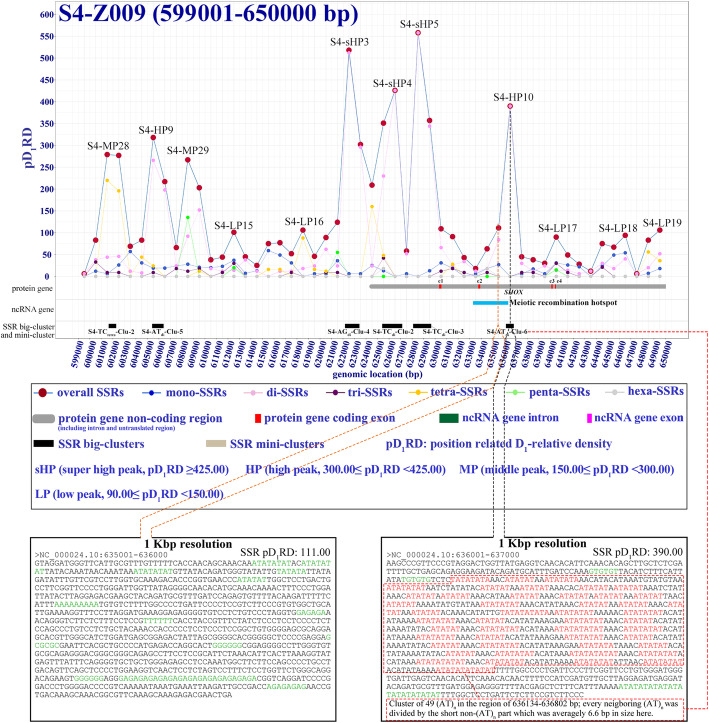
A typical map of SSR position-related D_1_-relative density (pD_1_RD) in human reference Y-DNA (NC_000024.10) at resolution of 1 Kbp

To explore the exact features of SSR distributions in the large segment sequences, we developed a Differential Calculator of Microsatellites Version 2 (DCM V2) method, which can calculate SSR densities by dividing the large segments into many differential units, and the alteration of differential unit size may give different resolutions to reveal the feature of SSR distribution; herein, the differential unit size (D_n_) was used as the resolutions of 100, 50, 10, 5, 2 and 1 Kbp in 10 large segments. So a SSR position-related D_n_-relative density (pD_n_RD) concept was introduced in this method. The differential resolutions more than 50 Kbp revealed that the SSR pD_n_RD only vary a little around the average relative density value in the sequenced regions of the Y-DNA (Fig. 1e and Fig. S2). As the differential resolution size decreasing, the pD_n_RD variation level usually increases in the large segments (Fig. S3), and the 1 Kbp resolution can reveal a clearest pD_1_RD variation feature in these large segments of the reference Y-DNA (Fig. 2; Fig. S1.1-S1.540).

### The SSRs landscape at 1 Kbp resolution

**Fig. 3:**
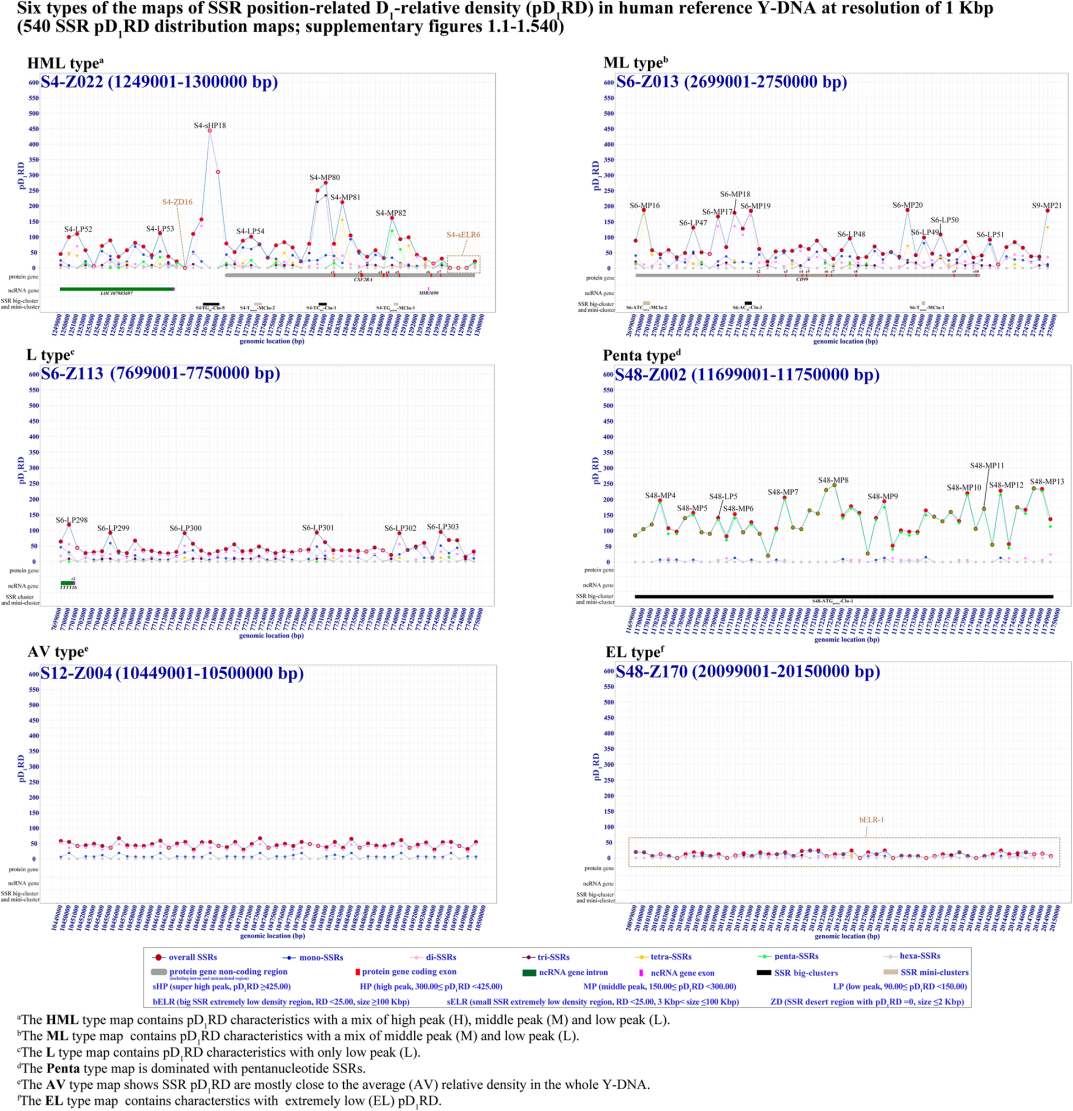
The six types of SSR pD_1_RD distribution maps in human reference Y-DNA (NC_000024.10) at resolution of 1 Kbp

**Fig. 4:**
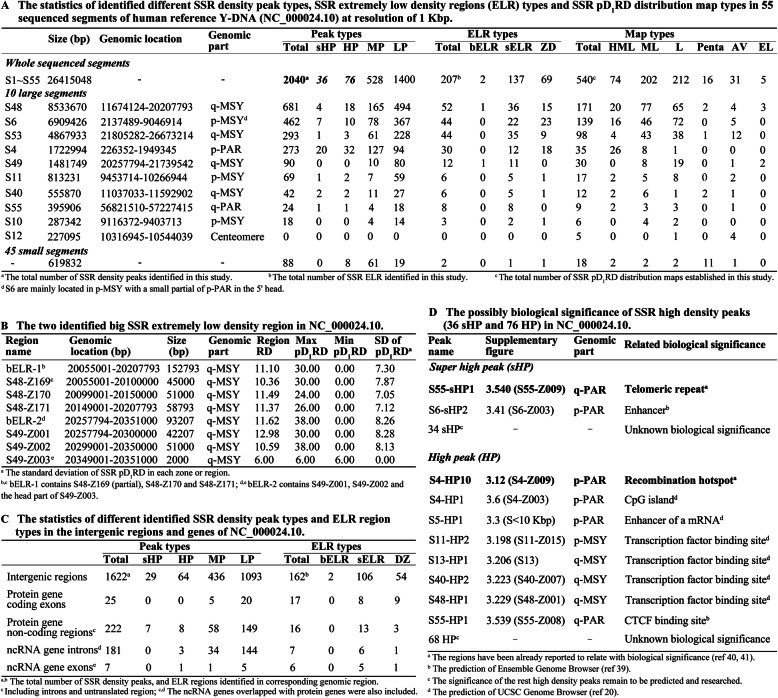
The statistics of different feature types of SSR pD_1_RD distributions in human reference Y-DNA (NC_000024.10) at resolution of 1 Kbp**. a** The statistics of identified different SSR density peak types, SSR extremely low density regions (ELR) types and SSR pD_1_RD distribution map types. **b** The two identified big SSR extremely low density region. **c** The statistics of different identified SSR density peak types and ELR region types in the intergenic regions and genes. **d** The possibly biological significance of SSR high density peaks (36 sHP and 76 HP), and details were listed in Table S4

We obtained 540 maps of SSR position-related relative densities in the reference sequence of human Y-DNA by investigation at 1 Kbp differential resolution, and each map usually contains 51 bins of 1 Kbp with overlapping 1 bin to bilateral maps (Figs. S1.1-S1.540). These maps show an exact landscape of SSR distribution with significant variation of position D_1_-relative SSR densities at different genomic positions as described in Figs. 2 and 3. The SSRs were observed to accumulate in 2040 differential bins of 1 Kbp genomic region forming mountain peak like SSR density peaks with pD_1_RD much higher than the average relative density in sequenced part of human reference Y-DNA; the SSR density peaks can be divided into 4 levels including 36 super high density peaks (sHP, pD_1_RD ≥ 425.00), 76 high density peaks (HP, 300.00 ≤ pD_1_RD < 425.00), 528 middle density peaks (MP, 150.00 ≤ pD_1_RD < 300.00) and 1400 low density peaks (LP, 90.00 ≤ pD_1_RD < 150.00) (Fig. 4 and Figs. S1.1–1.540). On the contrary, SSRs appear with extremely low densities in some genomic regions, and these regions can be grouped into 3 kinds including 2 big SSR extremely low density regions (bELR, RD < 25.00, size ≥100 Kbp), 137 small SSR extremely low density regions (sELR, RD < 25.00, 3 Kbp ≤ size < 100 Kbp) and 69 SSR desert regions (ZD, pD_1_RD = 0, size ≤2 Kbp) (Fig. 4 and Figs. S1.1-S1.540). Therefore, the 51 bins usually have different pD_1_RD making each map mixed with different SSR density peaks and extremely low density region, and the 540 maps can be typically classified into 6 types: 74 HML type maps with mix of high, middle and low density peaks, 202 ML type maps with mix of middle and low peaks, 212 L type maps with only low peaks, 16 Penta type maps with domination of pentanucleotide SSRs, 31 AV type maps with all pD_1_RD close to the genomic average relative density, and 5 EL type maps with all pD_1_RD very lower than average (Fig. 3 and Figs. S1.1-S1.540).

### Clusters of microsatellites

**Fig. 5:**
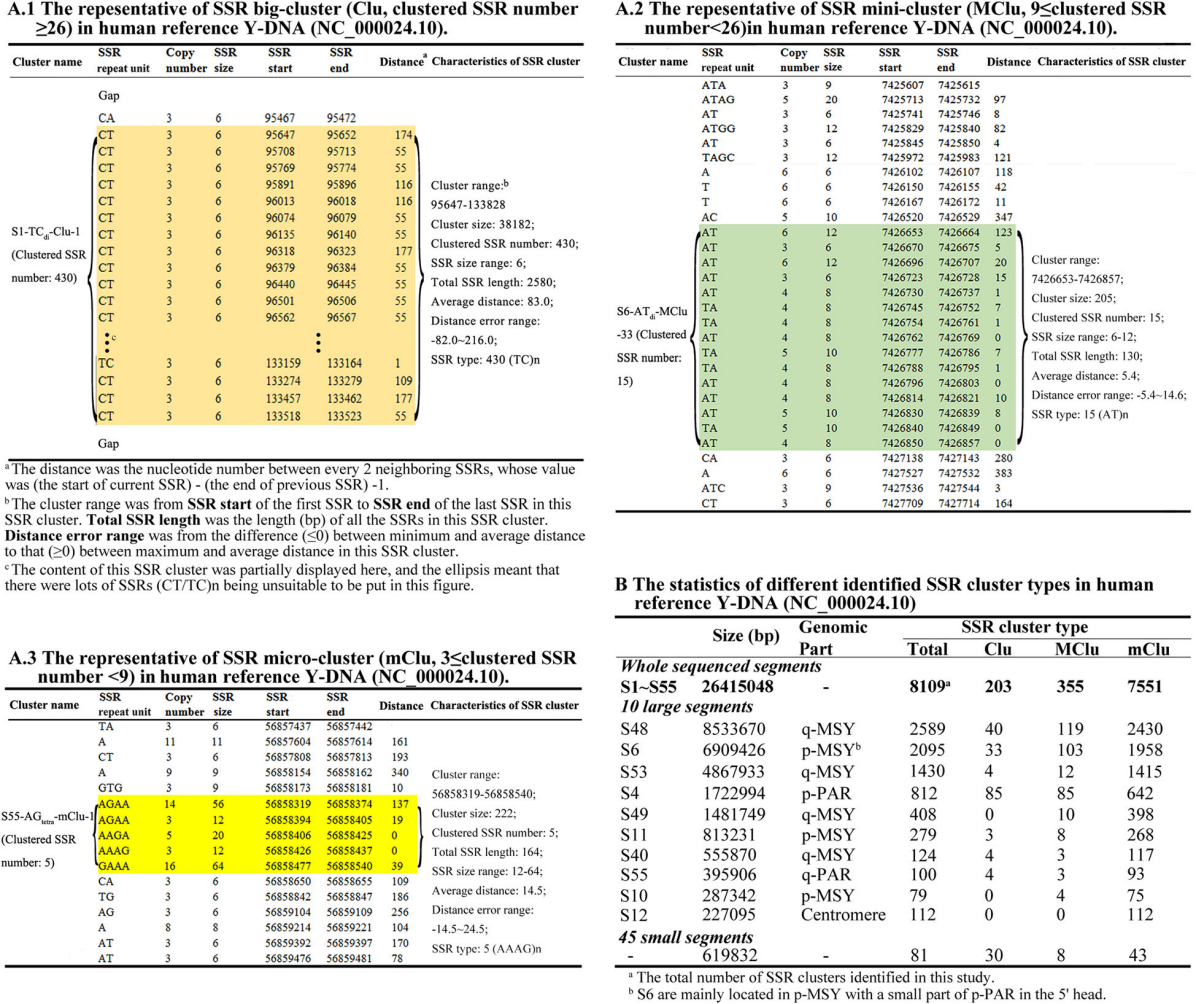
The clusters of many SSRs with same or similar motif in human reference Y-DNA (NC_000024.10). **(A.1-A.3)** The typical 3 levels of SSR clusters including SSR big clusters, mini-clusters and micro-clusters. (**B)** The statistics of different identified SSR cluster types in human reference Y-DNA (NC_000024.10)

It was also found that there are large numbers of SSRs with same or similar motif which neighborly locate together without other SSR motif in many regions of this human reference Y-DNA (Table S2). Some of these regions even harbor hundreds of such kind of same or similar SSR motifs, for example, there are 430 (CT/TC)_6_ without other SSR motif at the region of 95,647–133,828 bp of the Y-DNA (Fig. 5A.1); and some harbor dozens of or more than 3 same or similar SSR motifs, like 15 (AT/TA)_n_ at the region of 7,426,653–7,426,857 bp (Fig. 5A.2) and 5 (AAAG/AAGA/AGAA/GAAA)_n_ at the region of 56,858,319–56,858,540 bp of the Y-DNA (Fig. 5A.3). The regions of these specific SSR distributions can be defined as SSR clusters in this study; there are totally 8109 identified SSR clusters in sequenced part of the Y-DNA, which can be grouped into 3 levels including 203 big clusters (Clu, clustered same (similar) SSR number ≥ 26), 355 mini-clusters (MClu, 9 ≤ clustered same (similar) SSR number < 26) and 7551 micro-clusters (mClu, 3 ≤ clustered same SSR number < 9) (Fig. 5b and Table S3).

## Discussion

Our comprehensive survey of microsatellite distributions at 1 Kbp differential resolution to gain an exact landscape in the human reference Y-DNA (NC_000024.10), and 540 SSR landscape maps were obtained; these maps show that SSRs are accumulated significantly in some small regions and also seriously sparse in some regions; and many same or similar motif SSRs were observed to locate neighborly forming SSR clusters. Large numbers of SSRs in human Y-DNA have been previously understudied because the related studies usually focus on some significant Y SSR markers, or only analyzed the average distributions in the coding, noncoding and intergenic regions [7, 18, 19, 25, 28, 29, 38]. And UCSC Genome Browser might be not specific to highlight microsatellite distributional variation in every genomic position [20] (Fig. S4). The 540 SSR landscape maps in this study can provide a comprehensive view of clear SSR distributional features in every 1 Kbp genomic region along the human reference Y-DNA, and these maps can detailedly highlight the significant variations of position-related microsatellite distributions in this Y-DNA. And our studies may be helpful to reveal the microsatellite distributional laws and to further explore the biological significance of SSRs in the human reference Y-DNA.

Our observation of significant SSR accumulations to form density peaks indicates an obviously statistic bias of SSR distributions in the human reference Y-DNA, and such accumulations were also observed in other human and mammal Y-DNA (Figs. S5 and S6), suggesting that these SSR accumulations with forming high density peaks were possibly selected for being related to some biological significances. There are 112 identified high density peaks including 36 super high peaks and 76 high peaks in this study, implicating that the highly significant SSR accumulating regions totally represent 0.4% (112 Kbp / 26,415 Kbp) of the whole sequenced regions of the human reference Y-DNA, which are worth focusing on. And 10 of these 112 peaks have been already reported to possibly be related with known biological significance (Fig. 4d) [10, 19, 20, 39–41], for example, S4-HP10 is in a reported recombination hotspot in the p-arm pseudoautosomal region (p-PAR) of the Y-DNA, which might contribute to the mitotic recombination (Fig. 2 and 4d, Table S4) [40]; S55-sHP1 is in the telomeric region at the q-arm of the Y-DNA, which might be the boundary between telomere and euchromatic region [7, 19] (Fig. 4d). Though the biological significances of the other 102 peaks are not reported as many SSRs being lacking of understanding in human Y-DNA originally, these peaks may also play some important biological roles potentially, which probably deserve to be further explored [6, 10, 42–45]. In addition, those middle and low peaks possibly be also helpful to some biological process. Therefore, our fine maps of SSR landscape may provide the guide for mining the biological significance of such significant SSR accumulations with high densities in the human reference Y-DNA.

SSRs are widely considered to be more in noncoding and intergenic regions than coding regions [1–3, 6]. However, it is inconsistent with the wide consideration that the SSRs were detected to occur with extremely low densities in 2 big intergenic regions and many small noncoding and intergenic regions of the human reference Y-DNA, moreover, the SSRs were even not discovered in dozens of noncoding and intergenic regions of this Y-DNA, which have never been reported before to our knowledge (Figs. 3, 4B and C, Table S5). These events indicate the SSRs occurring in these regions might be not well tolerated and be exposed to strong negative selection as the commonly accepted view illustrating that many SSRs have evolved under serious selective constraints in genomes [1, 13, 46]. Our SSR landscape maps showed significantly regional variation of SSR densities in human reference Y-DNA, and it may be worthwhile to deeply explore the relationship between the selection and the significantly regional variation of SSR densities. Thus, our studies could significantly augment the knowledge of SSR landscapes in every different coding, noncoding and intergenic region of human Y-DNA, and also may contribute to the study of SSR evolution in human Y-DNA.

Besides clearly showing SSR landscapes, this study also revealed that many SSRs with same or similar motif are located neighborly to form over 8000 different sizes of SSR clusters in the sequenced regions of human reference Y-DNA. Many SSR clusters were detected to distribute numbers of SSRs with same motif and small neighboring SSR distances, contributing to forming the SSR density peaks, and some SSR clusters even distribute hundreds of identical SSRs with regular neighboring SSR distances (Tables S2 and S3). SSRs were reported to likely have evolved from an initial expansion of a short existing sequence motif and to be the quickly expandable compositions in genomes [5, 13, 17, 46]; so, the SSR clusters can be also assumed to have evolved from the expansions of an existing SSR motif, and it may be valuable for further studying their evolutionary process in human Y-DNA.

## Conclusions

SSRs are commonly thought to be not just the simple sequences randomly distributed in genomes. The distributional laws of SSRs in human Y-DNA still remain to be further studied, but we hope that this study with 540 exact SSR landscape maps in human reference Y-DNA will help to elucidate the genetic and evolutionary mechanism involved, and our DCM 2.0 method can contribute to clarifying the SSR landscapes in other genomic sequences.

## Methods

### Genomic sequence selection

We selected the reported reference genomic sequence of human chromosome Y (NC_000024.10), which is the published human Y-DNA with the highest sequencing completion (yet unfinished) and most convincing accuracy. The non-sequenced compositions (gaps) separated this reference Y-DNA into 55 different sequenced segments, which can be grouped into large (≥100 Kbp) and small (< 100 Kbp) size segments (Table S1).

### SSR identification

IMEx, a program with friendly interface, was utilized to identify perfect SSRs in this analysis [47]. The lowest copy number of repeat units, which is the threshold to identify the SSRs, was 6, 3, 3, 3, 3, 3 for extracting mono-, di-, tri-, tetra-, penta- and hexanucleotide SSRs respectively according to empirical criterion and previous SSR studies [34, 35]. Some other programs like Tandem Repeat Finder and RepeatMasker, which usually exclude many short SSRs (with default parameters, SSRs shorter than 25 bp are filtered out), were mainly applied as the reference tools here [36, 48].

### Method for fine maps of SSR landscapes in large genomic sequence

The former statistics of SSR relative density (RD) was usually calculated by the total SSR size dividing the total size of the sample genomic sequence [35, 49, 50], which could be described as the following formula:

1 $$ {\mathrm{D}}_{\mathrm{n}}={\mathrm{n}}_1={\mathrm{n}}_2={\mathrm{n}}_3=\dots ={\mathrm{n}}_{\mathrm{i}}=\dots ={\mathrm{n}}_{\mathrm{la}} $$

In this formula (1), M is the total size of SSRs (microsatellites) in the sample genomic sequence; N is the size of the sample genomic sequence. This method can only illustrate the global average value of SSR distributions in the sample genomic sequences, and the size of sample genomic sequence is usually very large, so this average value is actually not able to represent exact features of the SSR distributions in corresponding sequence.

We developed the Differential Calculator of Microsatellites Version 2.0 (DCM v2.0) method in the basis of DCM v1.0 [51] to calculate SSR relative density in every region along the large genomic sequence, which is aimed to reveal fine SSR landscapes in the genomic sequences. Firstly, DCM v2.0 can partition the large genomic sequence (N) into numerous differential units, which can be described as:

2 $$ \mathrm{N}=\sum {\mathrm{n}}_{\mathrm{i}} $$

3 $$ {\mathrm{D}}_{\mathrm{n}}={\mathrm{n}}_1={\mathrm{n}}_2={\mathrm{n}}_3=\dots ={\mathrm{n}}_{\mathrm{i}}=\dots ={\mathrm{n}}_{\mathrm{la}} $$

In these 2 formulas, n_i_ is the size of the i-th differential bin (i = 1, 2, 3, … i, i + 1, … la; la = the last) in large genomic sequence; la is equal to rounding (N/D_n_) to up integer; D_n_ represents the resolution of differential unit size (Kbp), e.g., D_50_ means the differential resolution of 50 Kbp.

The differential resolution size (D_n_) critically affects the exactness of revealing SSR landscapes as well as the pixels affect the image quality, and la is negative proportional to D_n_. The large genomic sequence is like a large differential resolution size with la =1, which is not proper mentioned above, so it is necessary to process an investigation to adjust into the best size for revealing fine SSR landscapes.

Then DCM 2.0 can calculate the SSR relative density in each differential unit respectively, equaling to partitioning the total SSR size (M) into local SSR sizes in numerous differential units of the large genomic sequence, expressed as the following formula:

4 $$ \mathrm{M}=\sum {\mathrm{m}}_{\mathrm{i}} $$

In this formula (4), m_i_ is the size of SSRs in the i-th differential bin; it is also critically affected by the differential resolution size (D_n_).

Each differential unit represents a small region of the large genomic sequence, so a concept of SSR position-related D_n_-relative density (pD_n_RD) was introduced in this method, which reflects SSR distributional features are critically influenced by both position (the i-th differential bin) and differential resolution size (D_n_), and it can be expressed as:

5 $$ {\mathrm{pD}}_{\mathrm{n}}{\mathrm{RD}}_{\mathrm{i}}=\frac{{\mathrm{m}}_{\mathrm{i}}}{{\mathrm{D}}_{\mathrm{n}}}\times 100 $$

pD_n_RD_i_ is the pD_n_RD in the i-th differential bin at the large genomic sequence. And the standard deviation (SD) of pD_n_RD was used to test the exactness of SSR distributional features at the corresponding differential resolution size.

### Visualization for exact SSR landscapes

Ggplot2, a R package, was utilized to visualize the SSR pD_n_RD distributions in the reference human Y-DNA into the Figures. Among the maps of SSR pD_n_RD distribution at 1 Kbp resolution (pD_1_RD), a normal size map represents a zone including 51 differential units of 1 Kbp with overlapping 1 unit to bilateral maps, and each zone was labeled into a zone serial number, e.g., S4-Z003 represents the third zone in S4. Owing to the gaps in this Y-DNA, some zones are in unnormal size, including those of the starts and ends of large sequenced segments, and those of short sequenced segments (labeled into only segment name, e.g., S13); the much short sequenced segments (size < 10 Kbp) were all integrated into the same map. The reported protein coding genes and ncRNA genes were also marked in these maps according to the annotation of NC_000024.10.

## Supplementary Information

**Additional file 1: Table S1.** The sequenced segments and SSR statistics of human reference Y-DNA (NC_000024.10).

**Additional file 2: Table S2**. The SSR clusters in the original statistics of SSR extraction in human reference Y-DNA (NC_000024.10)(S1: 10001–44,821 bp).

**Additional file 3: Table S3**. The features of identified SSR mini-clusters in 55 sequenced segments of human reference Y-DNA (NC_000024.10) (mini-cluster (MClu), 9 ≤ clustered same or similar SSR number < 26).

**Additional file 4: Table S4**. The features of identified super high density peaks in 55 segments of human reference Y-DNA (NC_000024.10) at 1 Kbp resolution (super high peak (sHP), pD1RD ≥ 425.00).

**Additional file 5: Table S5**. The statistics of identified different position related D_1_-relative density (pD_1_RD) map types in 55 sequenced segments of human reference Y-DNA (NC_000024.10).

**Additional file 6: Figure S1.** 540 SSR position related  D_1_-relative density maps in human Y-DNA (NC_000024.10) at 1 kilobase resolution.

**Additional file 7: Figure S2.** The SSR position related D_50_-relative density (pD_50_RD) map in 10 large segments of human reference Y-DNA (NC_000024.10).

**Additional file 8: Figure S3.** The comparison of SSR position related D_n_-relative density maps at differential resolutions of 100, 50, 10, 5, 2, 1 Kbp in human reference Y-DNA (NC_000024.10).

**Additional file 9: Figure S4.** The comparison of showing SSR distributional features in SSR pD_1_RD map and UCSC Genome Browser at the position of 1200001-1300000 bp in human reference Y-DNA (NC_000024.10).

**Additional file 10: Figure S5.** The example of comparing high SSR accumulations between human reference Y-DNA and other human Y-DNA at the same locations.

**Additional file 11: Figure S6.** The similar SSR accumulations of high densities located at the flanking region (about 15000 bp away) of SRY in human chimpanzee, rhesus monkey, mouse and rat reference Y.

## Data Availability

The accession number of the reference human Y-DNA used in this analysis is NC_000024.10. The annotation and information of protein-coding and ncRNA genes were collected from the Genbank file of NC_000024.10 and the corresponding name is *Homo sapiens Updated Annotation Release 109.20201120* (https://www.ncbi.nlm.nih.gov/genome/annotation_euk/Homo_sapiens/109.20201120/). The annotations of the regulatory regions and specific genomic structures were collected from *Genome Browser at University of California, Santa Cruz* (http://genome.ucsc.edu/) and *Ensemble Genome Browser* (www.ensemble.org)*.* The accession number of samples (alignment file) from 1000 human genome project (https://ftp-trace.ncbi.nlm.nih.gov/1000genomes/ftp/1000G_2504_high_coverage/data/) are NA07051 (https://ftp-trace.ncbi.nlm.nih.gov/1000genomes/ftp/1000G_2504_high_coverage/data/ERR3239281/) and NA18874 (https://ftp-trace.ncbi.nlm.nih.gov/1000genomes/ftp/1000G_2504_high_coverage/data/ERR3243161/). The accession number of the Y-DNA of chimpanzee, rhesus monkey, mouse and rat used in this analysis is NC_006492.4 (annotation: https://www.ncbi.nlm.nih.gov/genome/annotation_euk/Pan_troglodytes/105/), NC_027914.1 (annotation: https://www.ncbi.nlm.nih.gov/genome/annotation_euk/Macaca_mulatta/103/), NC_000087.8 (annotation: https://www.ncbi.nlm.nih.gov/genome/annotation_euk/Mus_musculus/109/) and NC_024475.1 (annotation: https://www.ncbi.nlm.nih.gov/genome/annotation_euk/Rattus_norvegicus/106/). Tables S1-S5 are available in https://github.com/DooYal/human-Y-supplementary-material/tree/master/Supplementary%20tables Figs. S1.1-S1.540 are available in https://dooyal.github.io/human_y_ssr_maps/ DCM 2.0 is available in https://github.com/DooYal/DCM

## Abbreviations

SSR: Simple sequence repeat
MSY: Male-specific region in Y
PAR: Pesudoautomosomal region
Kbp: kilobase
UCSC: University of California, Santa Cruz
RD: Relative density
SD: Standard deviation
D_n_: Differential unit size
pD_n_RD: Position-related D_n_-relative density
DCM V2: Differential Calculator of Microsatellites Version 2
sHP: Super high peak
HP: High peak
MP: Middle peak
LP: Low peak
bELR: Big extremely low (density) region
sELR: Small extremely low (density) region
ZD: Zero (density) desert
Clu: Big-cluster
MClu: Mini-cluster
mClu: Micro-cluster
