# Supplementary material for: A comprehensive microsatellite landscape of human Y-DNA at kilobase resolution
Source: BMC Genomics. 2021 Jan 22;22:76. doi: 10.1186/s12864-021-07389-5 (PMC7821415; doi:10.1186/s12864-021-07389-5)
Supplement: Supplementary file 6 — Additional file 6: Figure S1. 540 SSR position related D1-relative density maps in human Y-DNA (NC_000024.10) at 1 kilobase resolution. [file 12864_2021_7389_MOESM6_ESM.pdf]

High resolution version of the figures are available in https://dooyal.github.io/human_y_ssr_maps/

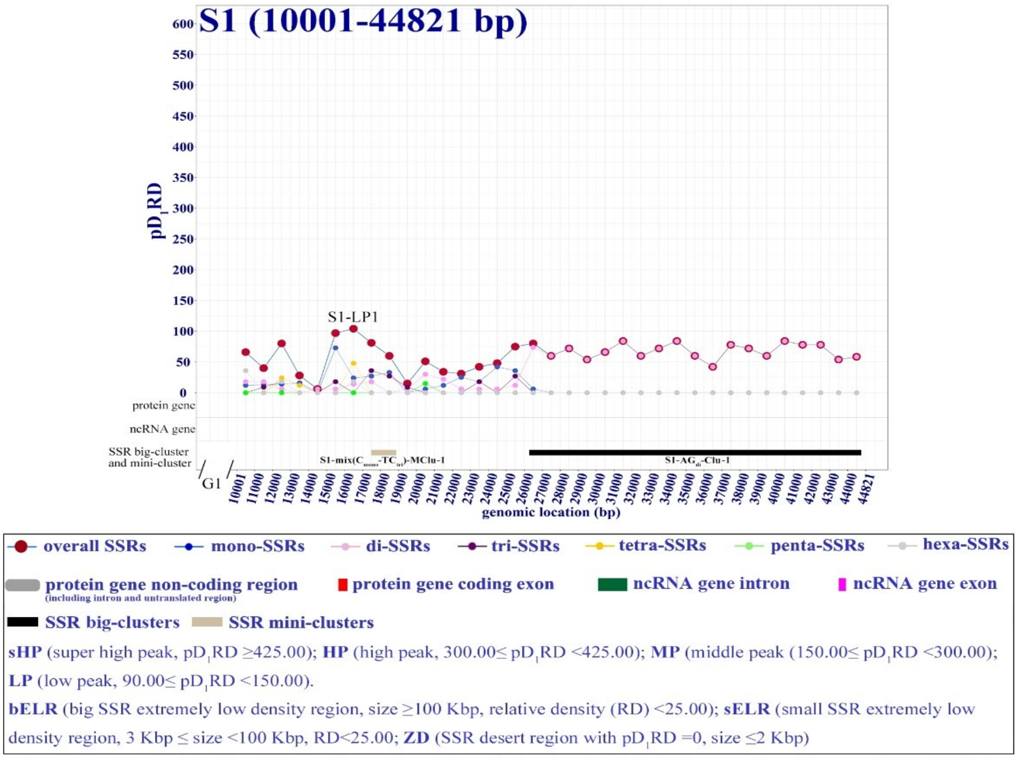

Supplemetary Figure 1.1. The SSR position related D_1-relative density (pD_1RD) map of position at 10001-44821 bp (unnormal zone <51000 Kbp) of human reference Y-DNA (NC_000024.10) at resolution of 1 Kbp.

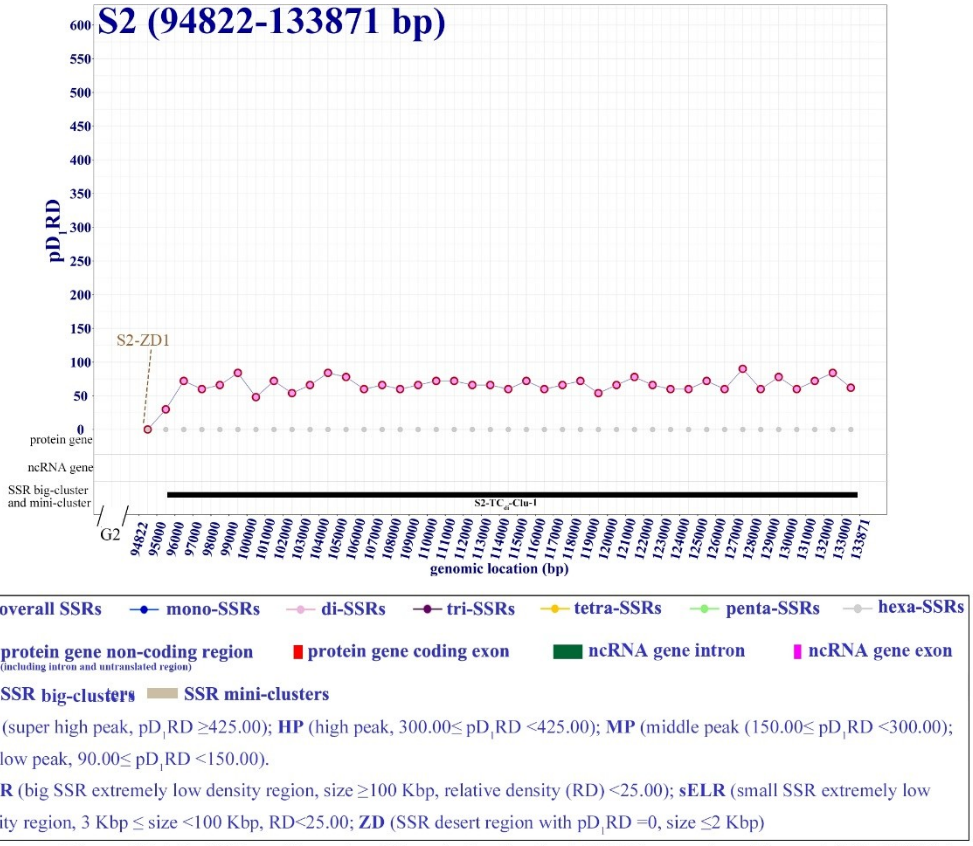

Supplemetary Figure 1.2. The SSR position related D_1-relative density (pD_1RD) map of position at 94822-133871 bp (unnormal zone <51000 Kbp) of human reference Y-DNA (NC_000024.10) at resolution of 1 Kbp.

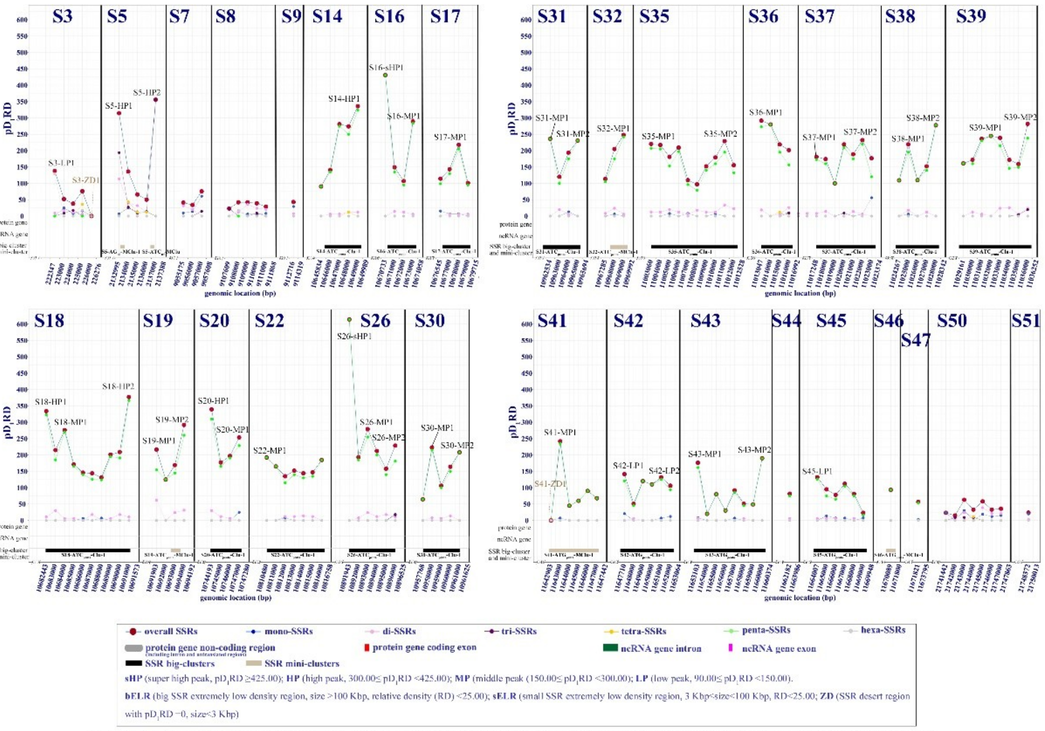

Supplementary Figure 1.3. The SSR position related D_1-relative density (pD_1RD) map of unnormal zones < 10 Kbp in human reference Y-DNA (NC_000024.10) at resolution of 1 Kbp.

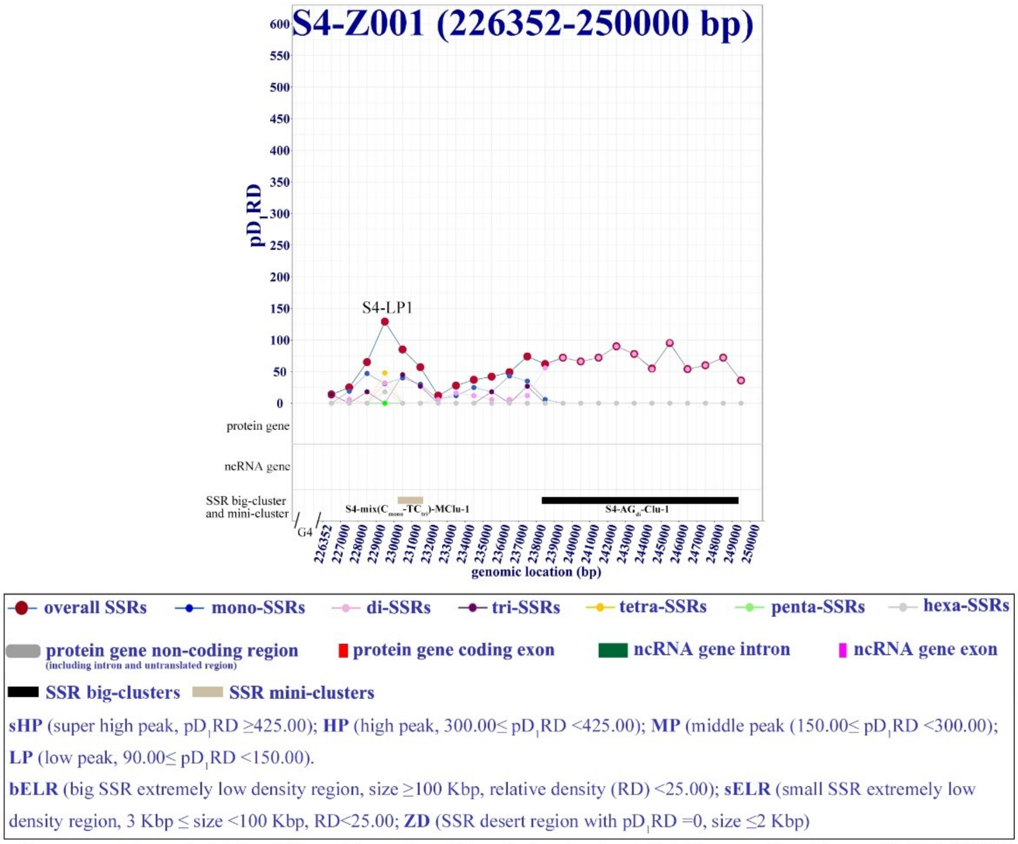

Supplementary Figure 1.4. The SSR position related D_1-relative density (pD_1RD) map of position at 226352-250000 bp (unnormal zone <51000 bp) of human reference Y-DNA (NC_000024.10) at resolution of 1 Kbp.

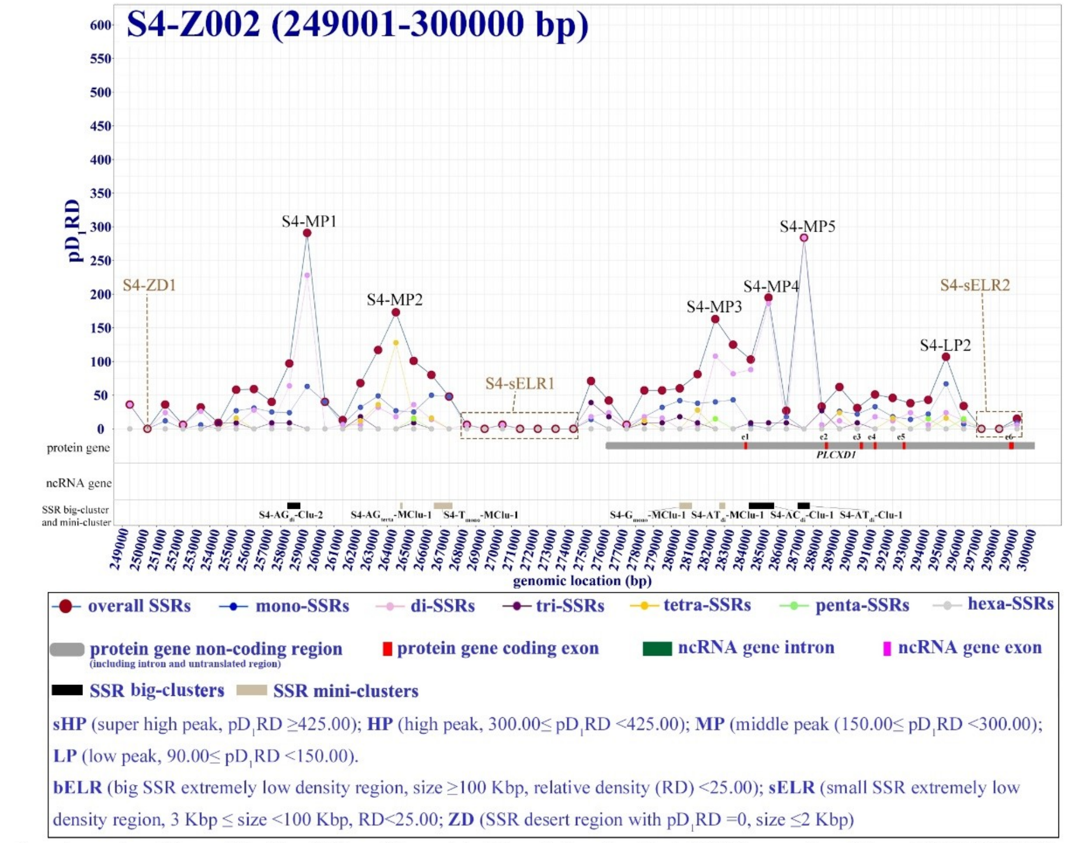

Supplementary Figure 1.5. The SSR position related D_1-relative density (pD_1RD) map of position at 249001-300000 bp of human reference Y-DNA (NC_000024.10) at resolution of 1 Kbp.

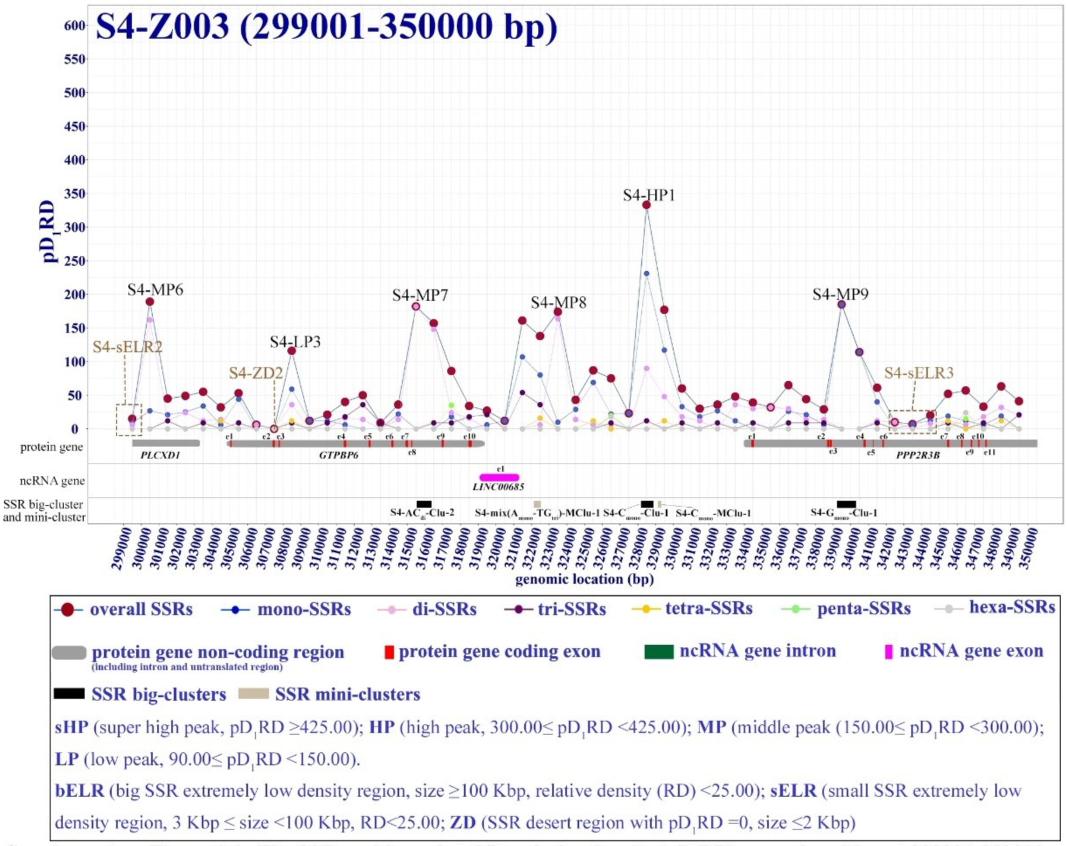

Supplementary Figure 1.6. The SSR position related D_1-relative density (pD_1RD) map of position at 299001-350000 bp of human reference Y-DNA (NC_000024.10) at resolution of 1 Kbp.

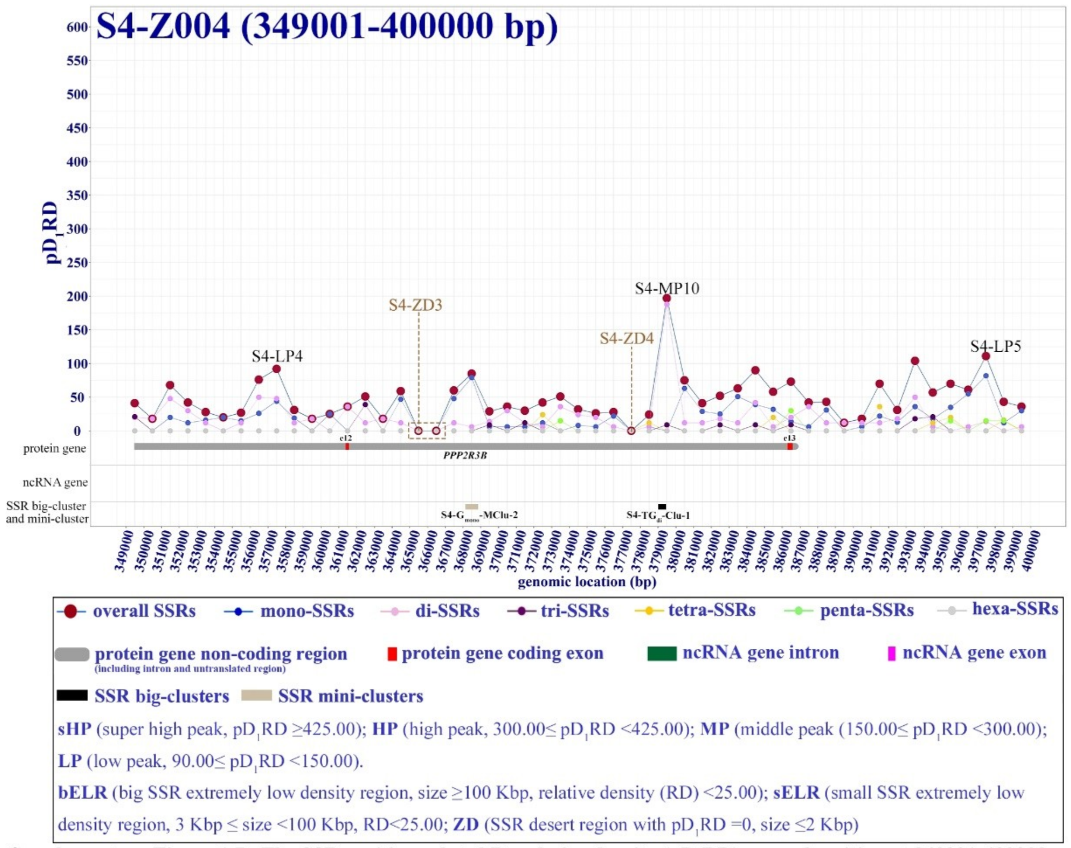

Supplementary Figure 1.7. The SSR position related D_1-relative density (pD_1RD) map of position at 349001-400000 bp of human reference Y-DNA (NC_000024.10) at resolution of 1 Kbp.

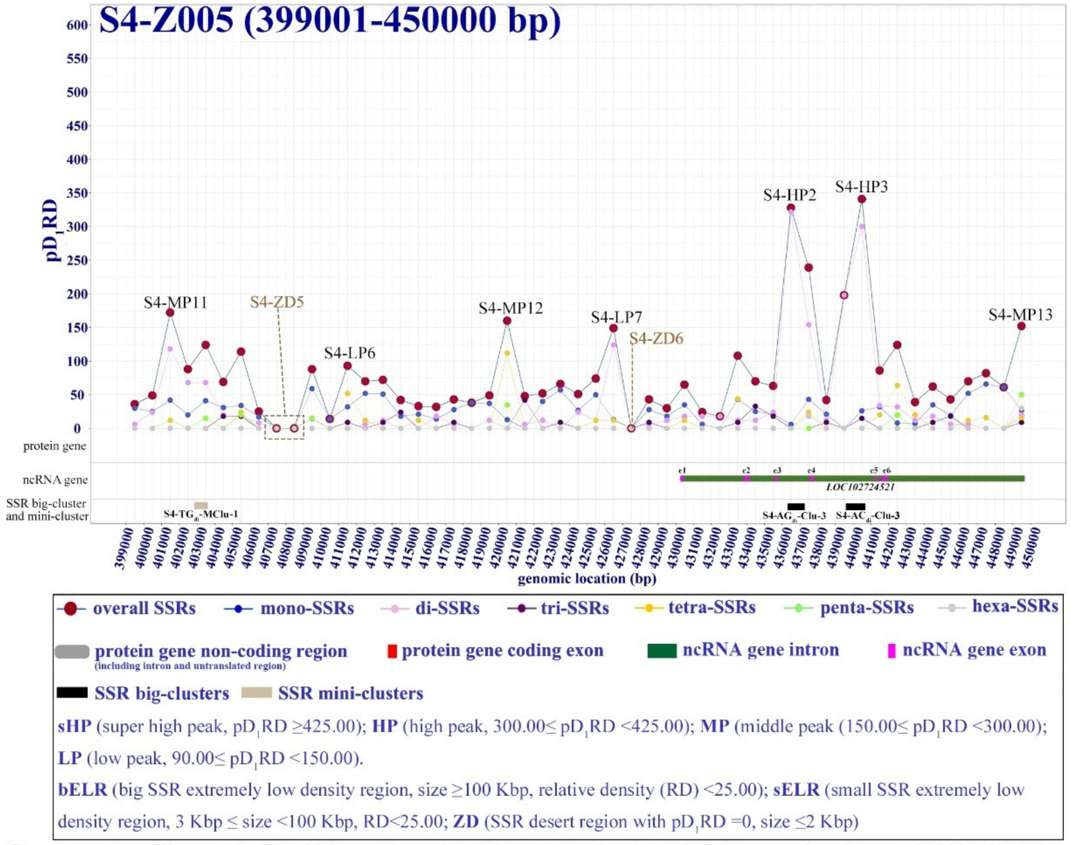

Supplementary Figure 1.8. The SSR position related D_1-relative density (pD_1RD) map of position at 399001-450000 bp of human reference Y-DNA (NC_000024.10) at resolution of 1 Kbp.

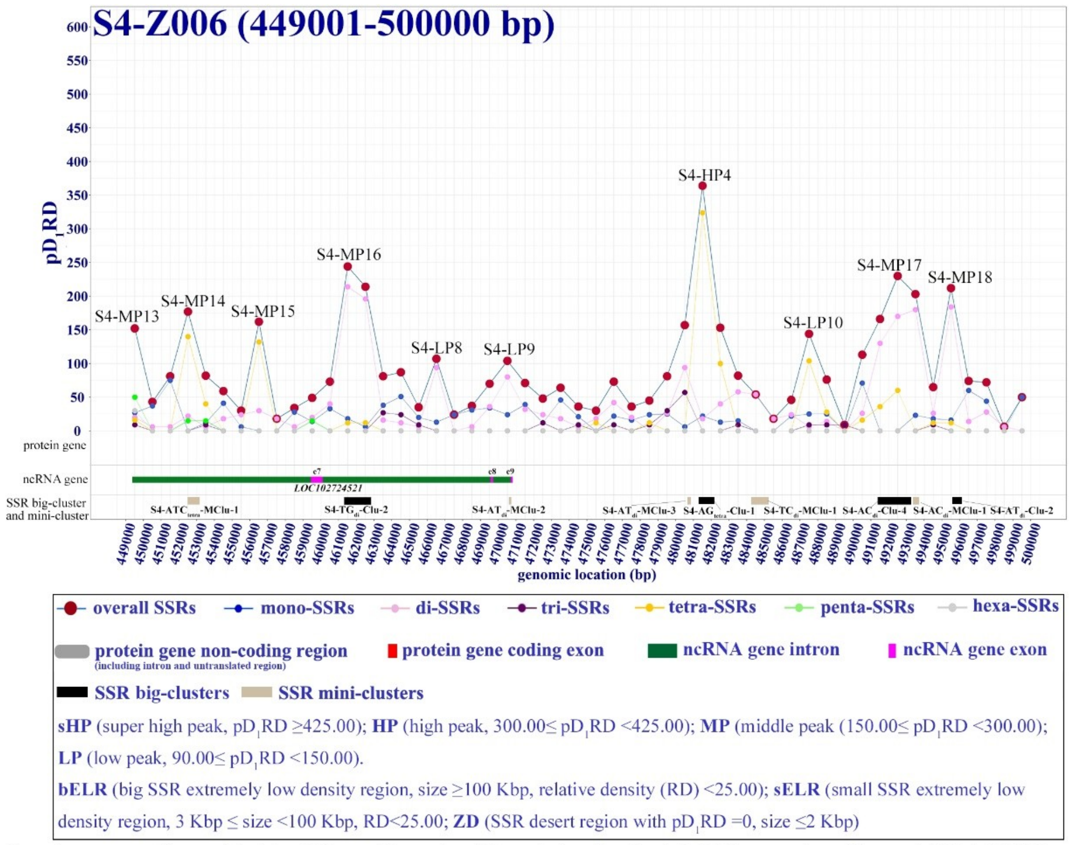

Supplementary Figure 1.9. The SSR position related D_1-relative density (pD_1RD) map of position at 449001-500000 bp of human reference Y-DNA (NC_000024.10) at resolution of 1 Kbp.

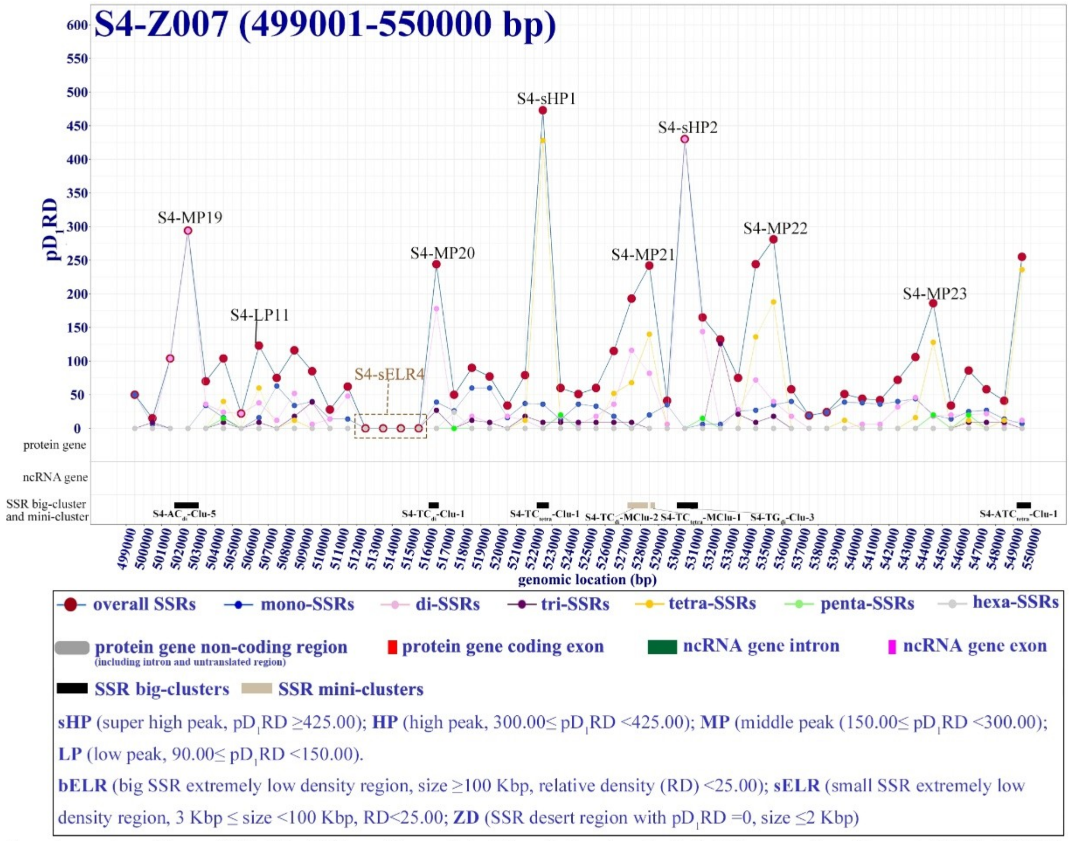

Supplementary Figure 1.10. The SSR position related D_1-relative density (pD_1RD) map of position at 499001-550000 bp of human reference Y-DNA (NC_000024.10) at resolution of 1 Kbp.

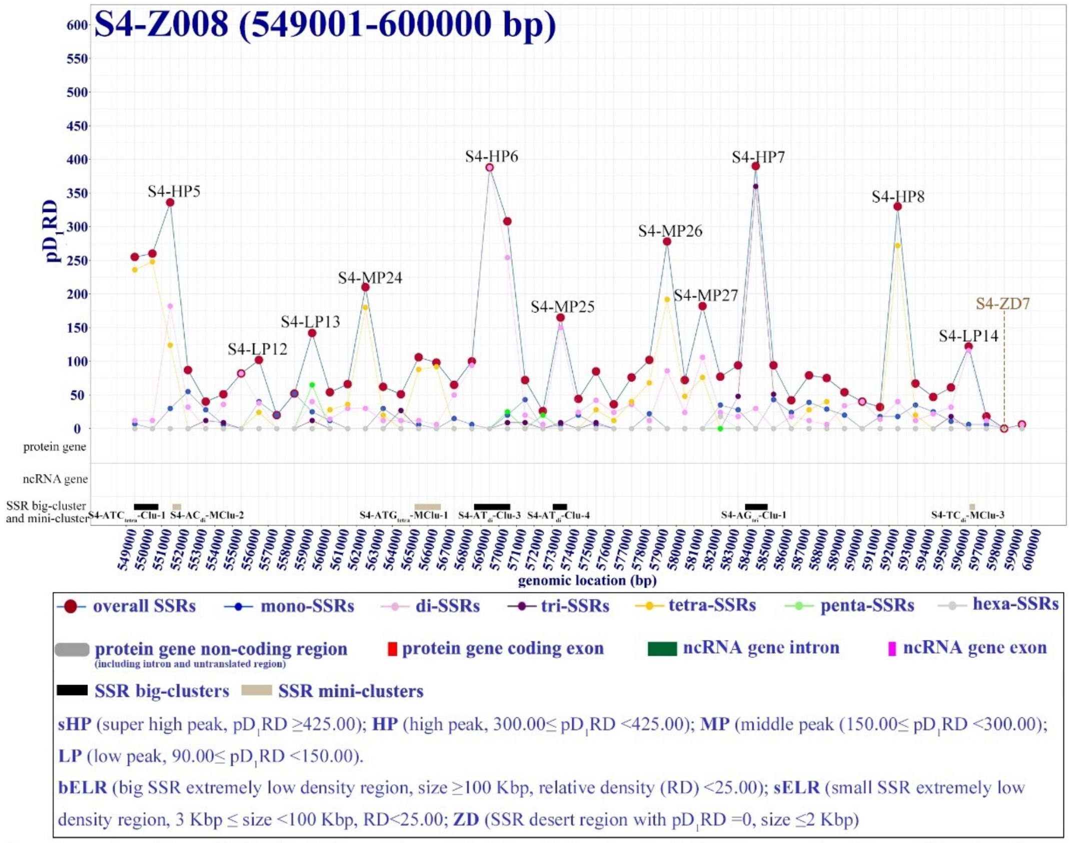

Supplementary Figure 1.11. The SSR position related D_1-relative density (pD_1RD) map of position at 549001-600000 bp of human reference Y-DNA (NC_000024.10) at resolution of 1 Kbp.

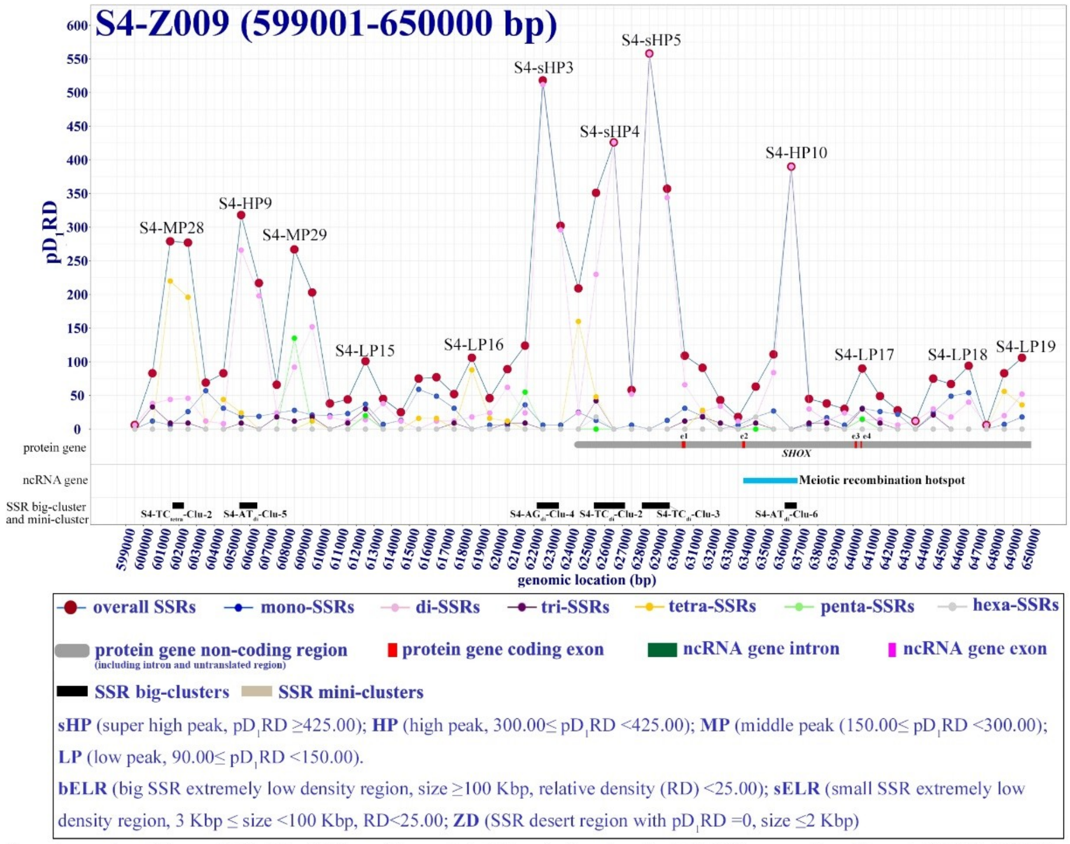

Supplementary Figure 1.12. The SSR position related D_1-relative density (pD_1RD) map of position at 599001-650000 bp of human reference Y-DNA (NC_000024.10) at resolution of 1 Kbp.

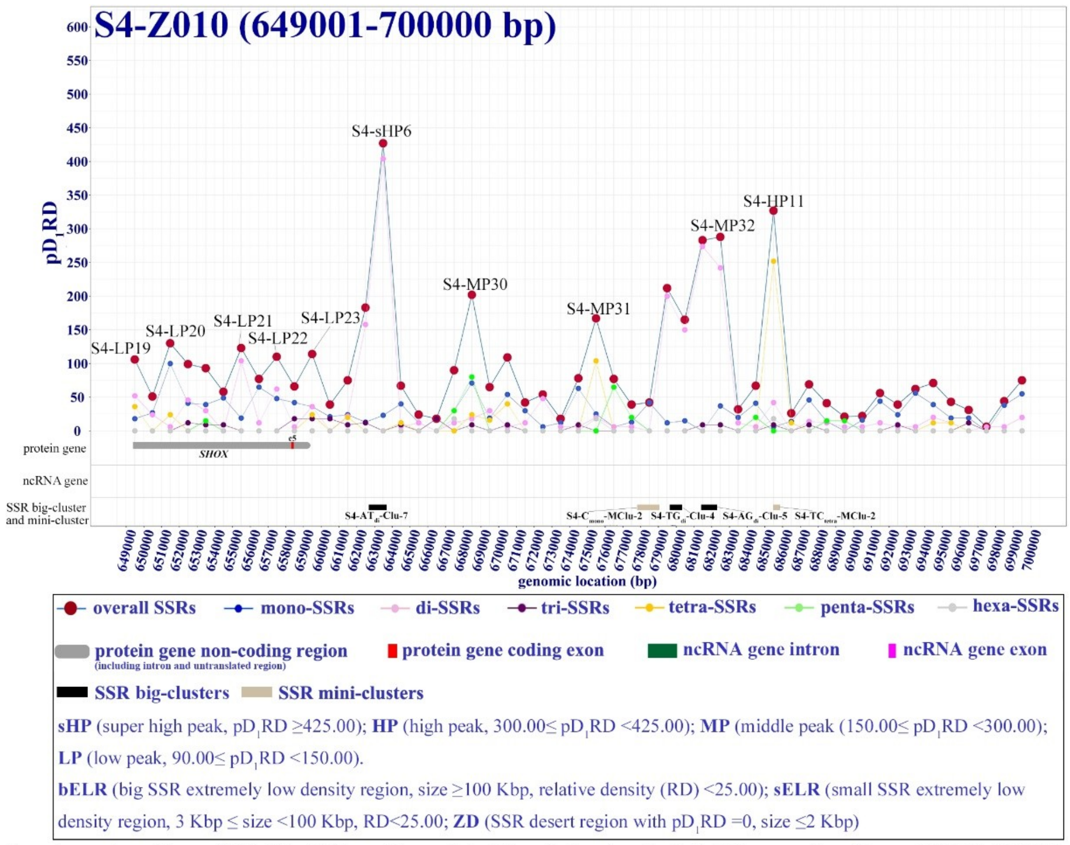

Supplementary Figure 1.13. The SSR position related D_1-relative density (pD_1RD) map of position at 649001-700000 bp of human reference Y-DNA (NC_000024.10) at resolution of 1 Kbp.

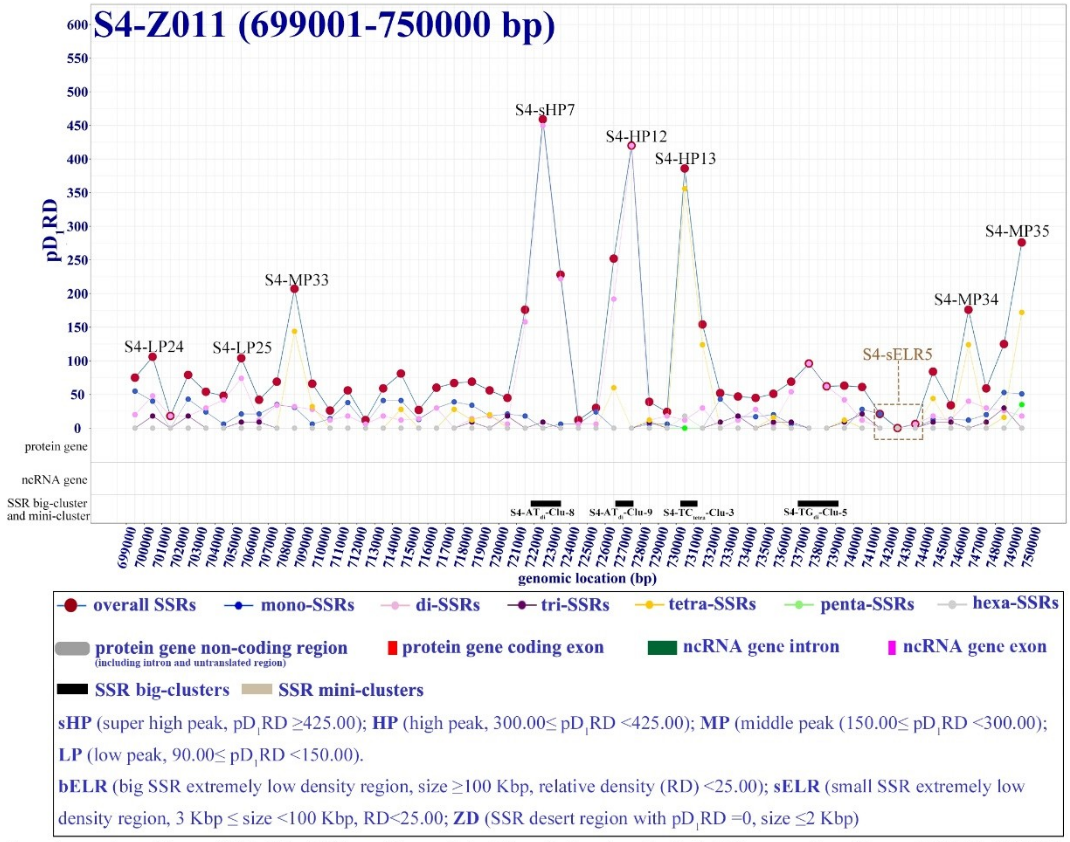

Supplementary Figure 1.14. The SSR position related D_1-relative density (pD_1RD) map of position at 699001-750000 bp of human reference Y-DNA (NC_000024.10) at resolution of 1 Kbp.

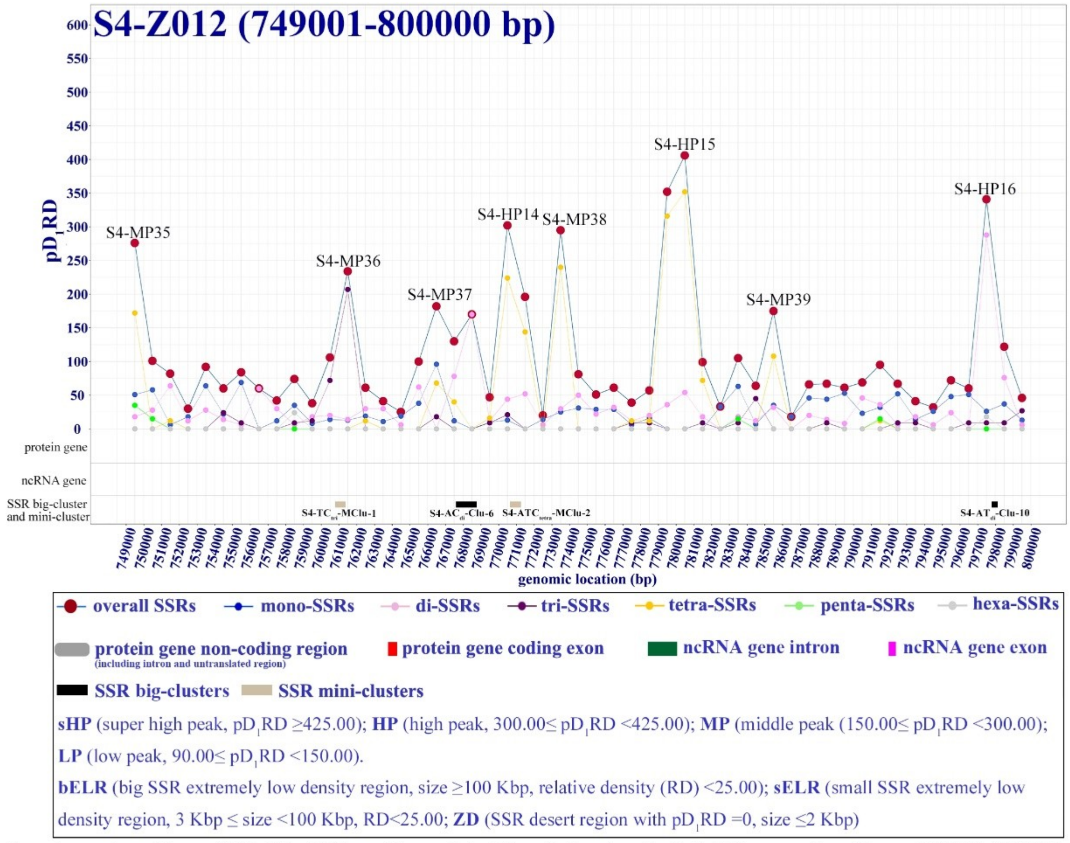

Supplementary Figure 1.15. The SSR position related D_1-relative density (pD_1RD) map of position at 749001-800000 bp of human reference Y-DNA (NC_000024.10) at resolution of 1 Kbp.

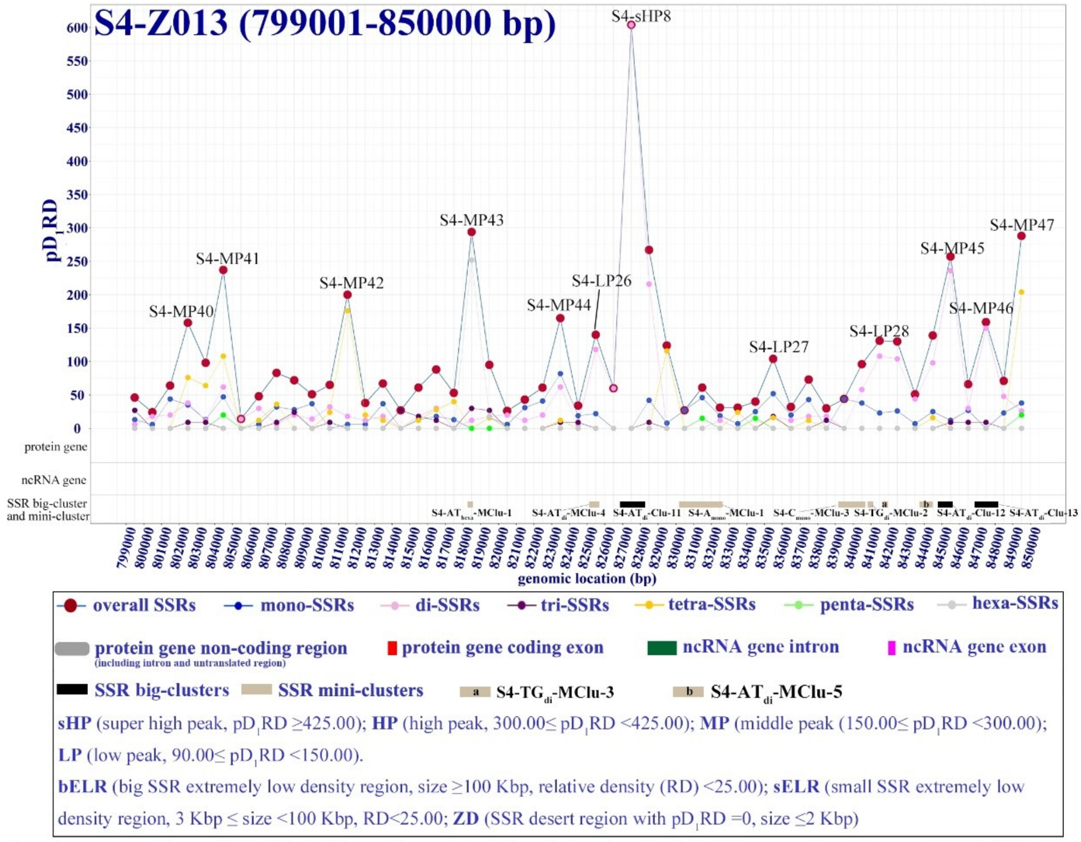

Supplementary Figure 1.16. The SSR position related D_1-relative density (pD_1RD) map of position at 799001-850000 bp of human reference Y-DNA (NC_000024.10) at resolution of 1 Kbp.

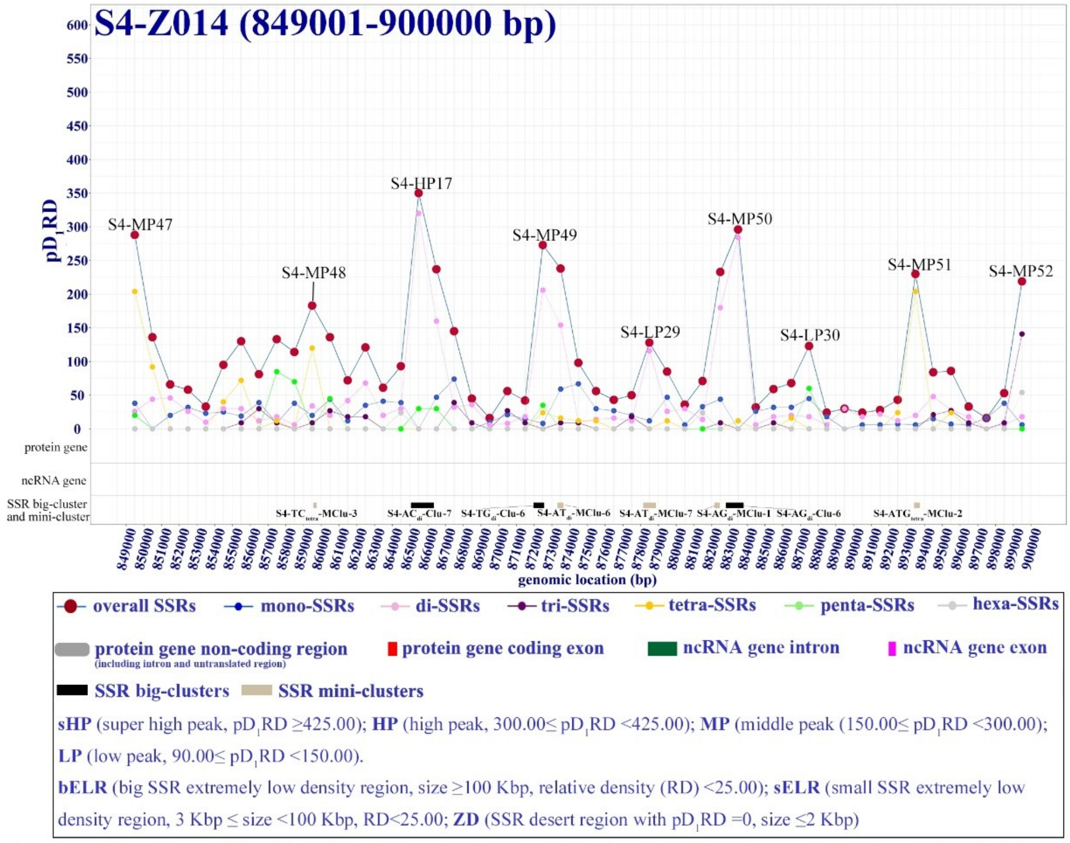

Supplementary Figure 1.17. The SSR position related D_1-relative density (pD_1RD) map of position at 849001-900000 bp of human reference Y-DNA (NC_000024.10) at resolution of 1 Kbp.

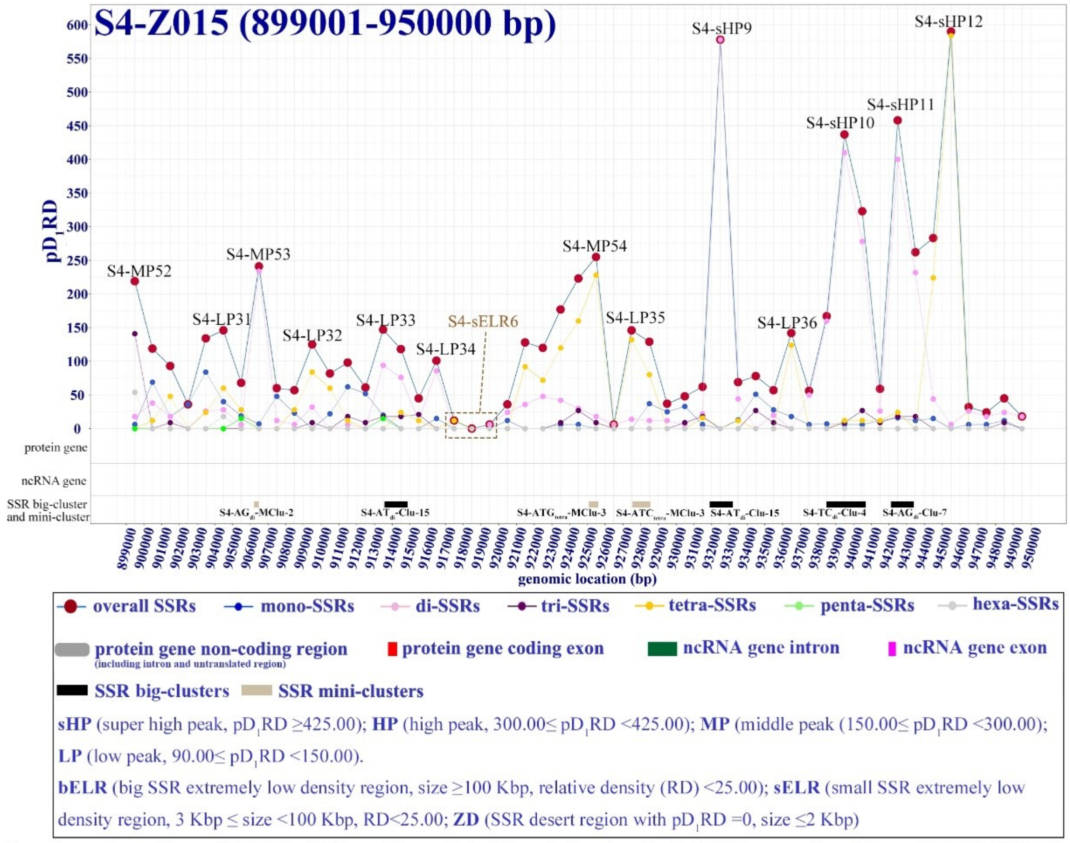

Supplementary Figure 1.18. The SSR position related D_1-relative density (pD_1RD) map of position at 899001-950000 bp of human reference Y-DNA (NC_000024.10) at resolution of 1 Kbp.

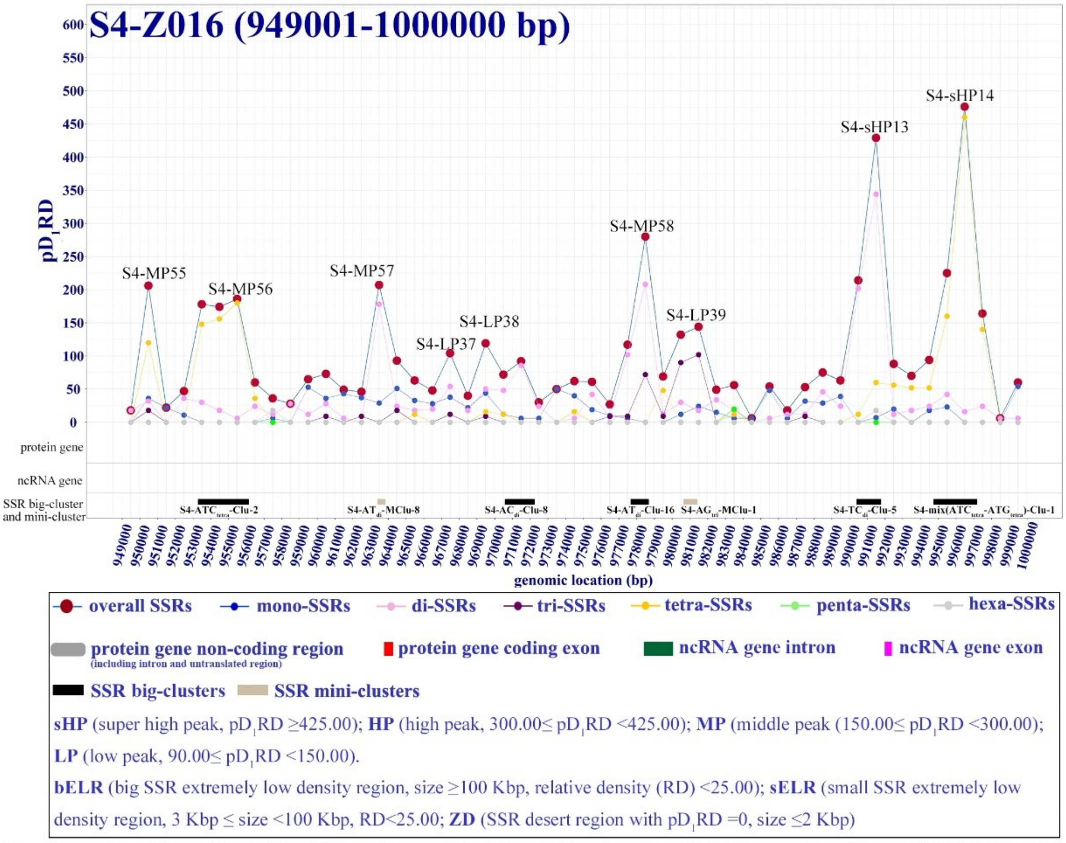

Supplementary Figure 1.19. The SSR position related D_1-relative density (pD_1RD) map of position at 949001-1000000 bp of human reference Y-DNA (NC_000024.10) at resolution of 1 Kbp.

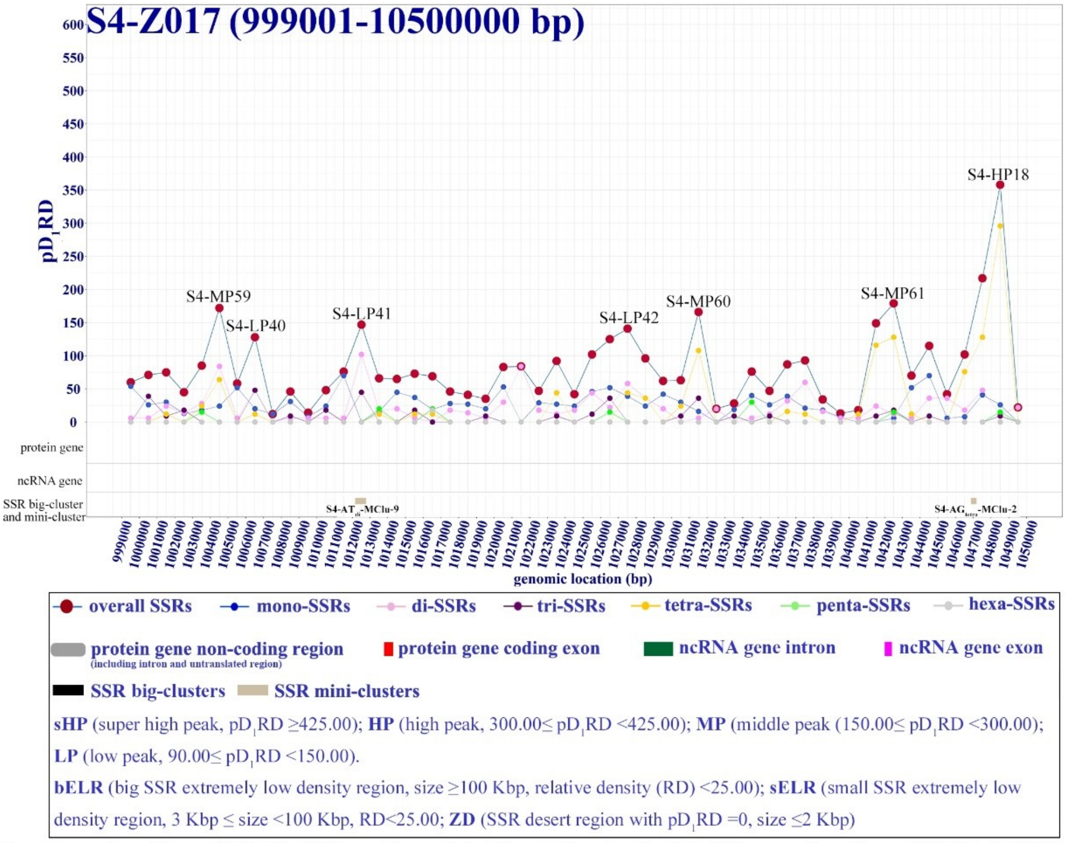

Supplementary Figure 1.20. The SSR position related D_1-relative density (pD_1RD) map of position at 999001-1050000 bp of human reference Y-DNA (NC_000024.10) at resolution of 1 Kbp.

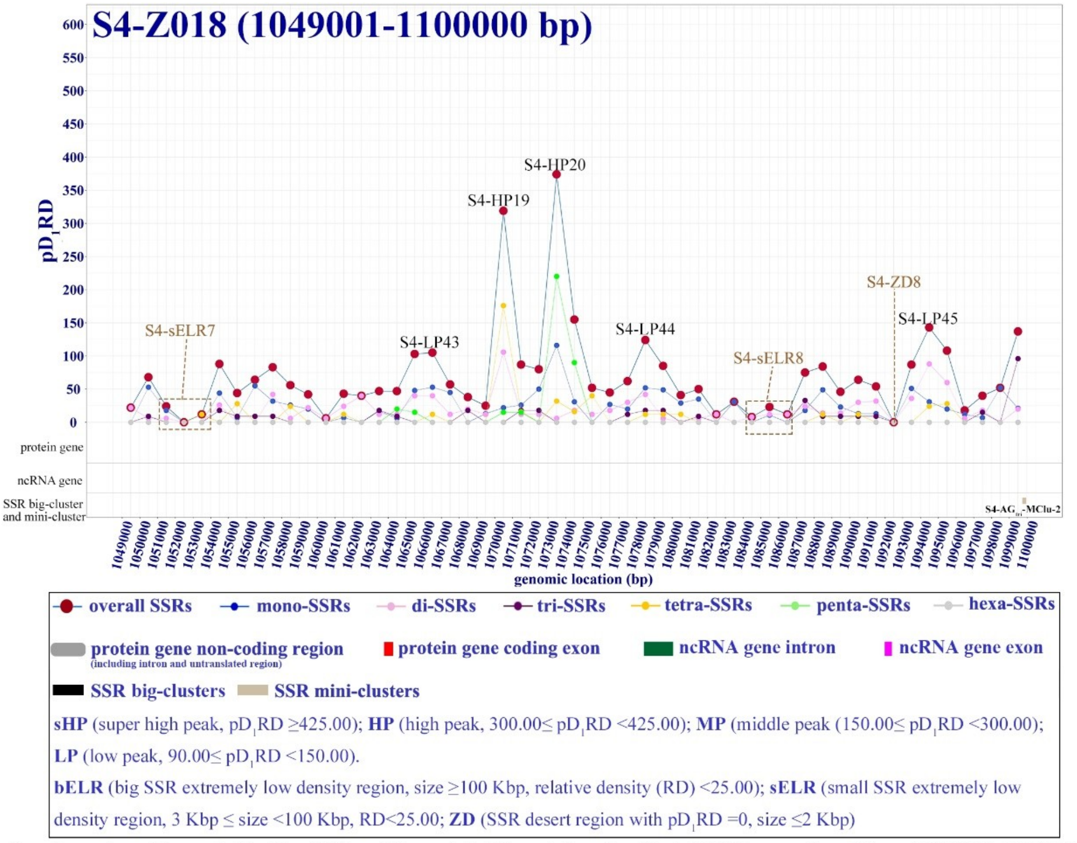

Supplementary Figure 1.21. The SSR position related D_1-relative density (pD_1RD) map of position at 1049001-1100000 bp of human reference Y-DNA (NC_000024.10) at resolution of 1 Kbp.

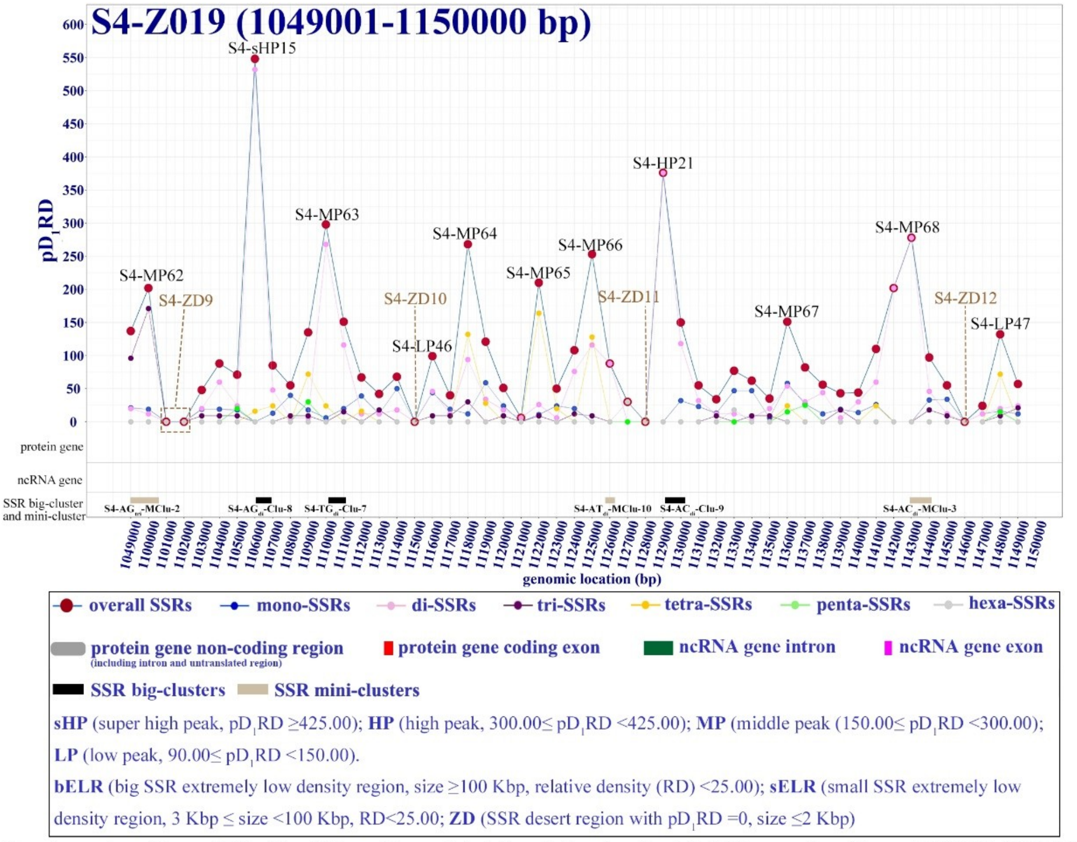

Supplementary Figure 1.22. The SSR position related D_1-relative density (pD_1RD) map of position at 1099001-1150000 bp of human reference Y-DNA (NC_000024.10) at resolution of 1 Kbp.

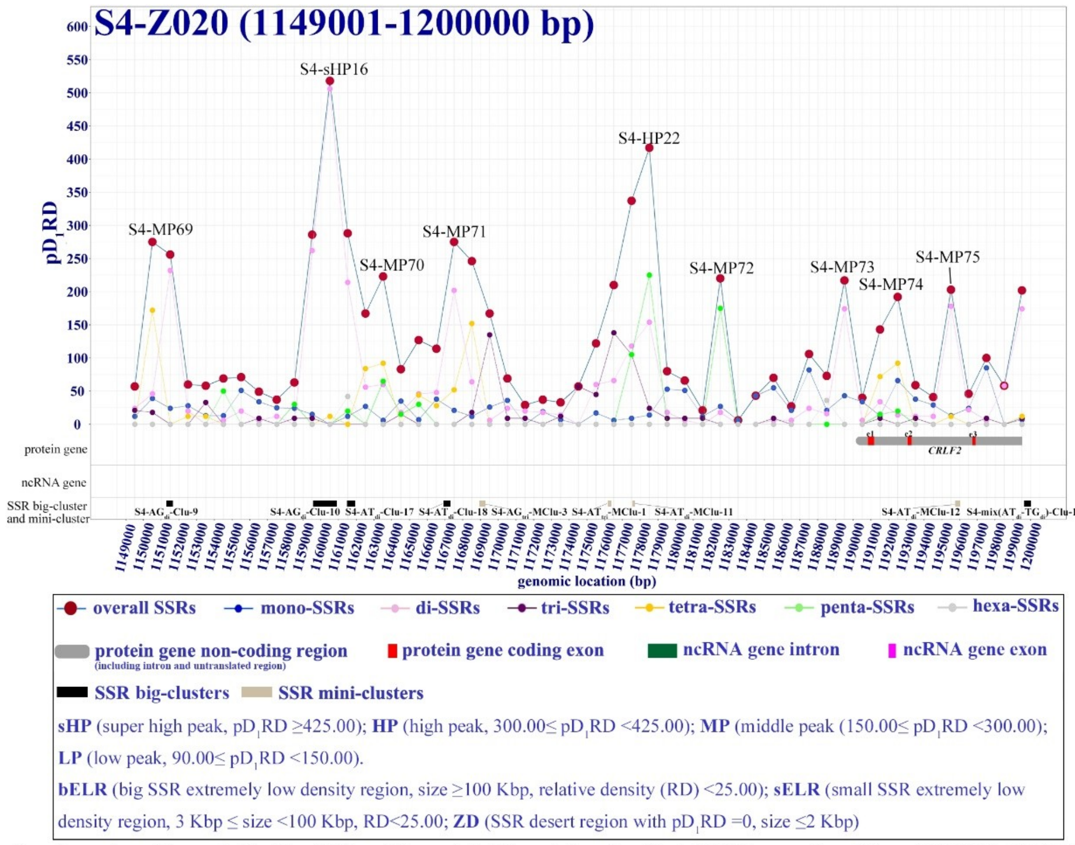

Supplementary Figure 1.23. The SSR position related D_1-relative density (pD_1RD) map of position at 1149001-1200000 bp of human reference Y-DNA (NC_000024.10) at resolution of 1 Kbp.

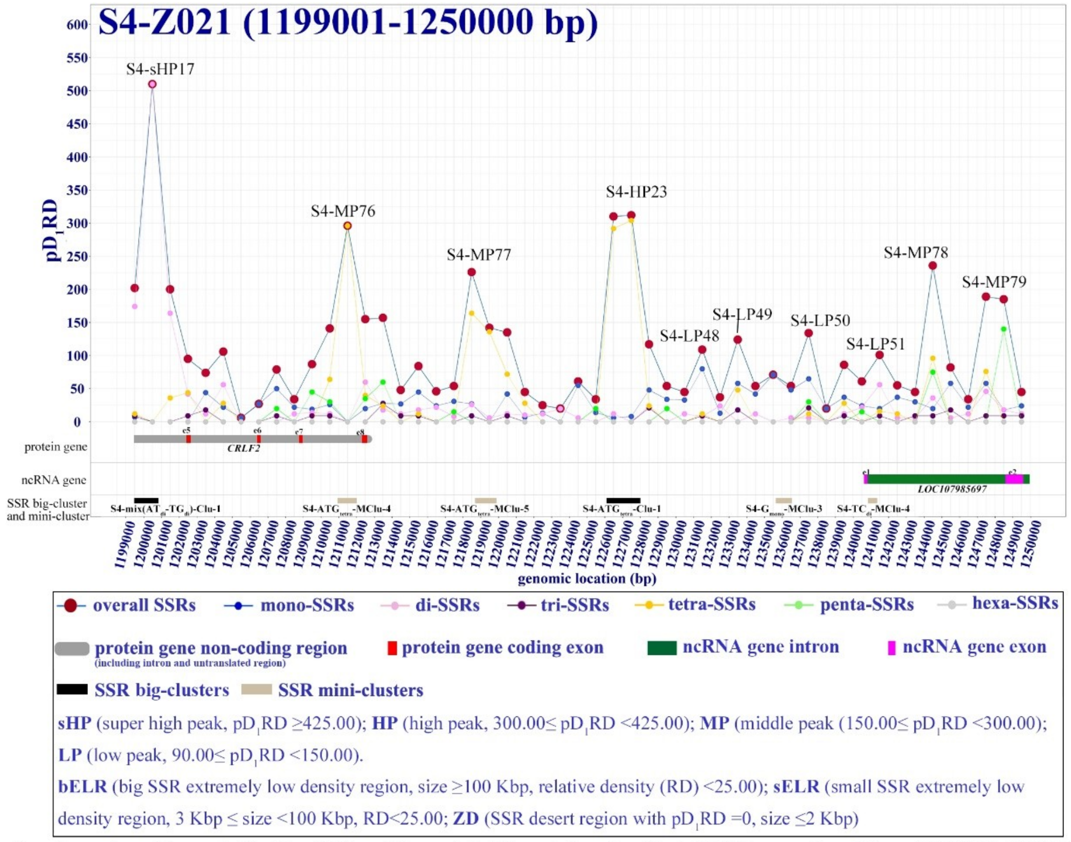

Supplementary Figure 1.24. The SSR position related D_1-relative density (pD_1RD) map of position at 1199001-1250000 bp of human reference Y-DNA (NC_000024.10) at resolution of 1 Kbp.

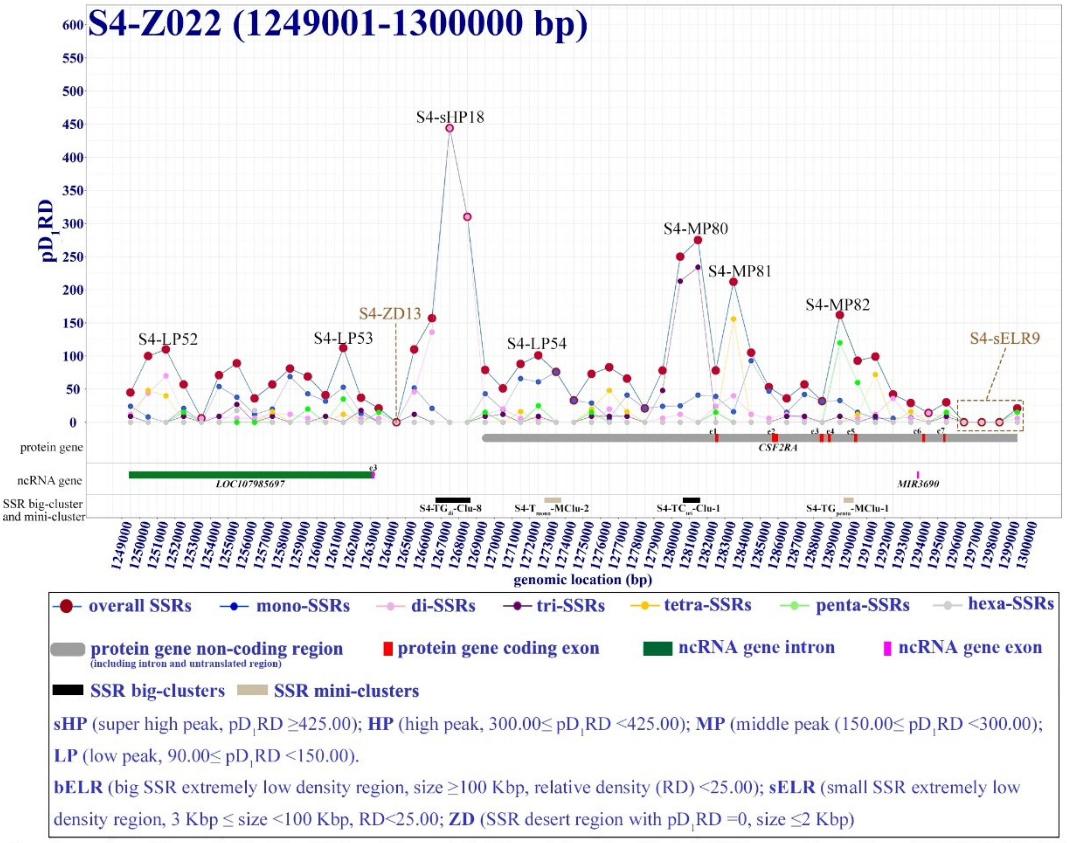

Supplementary Figure 1.25. The SSR position related D_1-relative density (pD_1RD) map of position at 1249001-1300000 bp of human reference Y-DNA (NC_000024.10) at resolution of 1 Kbp.

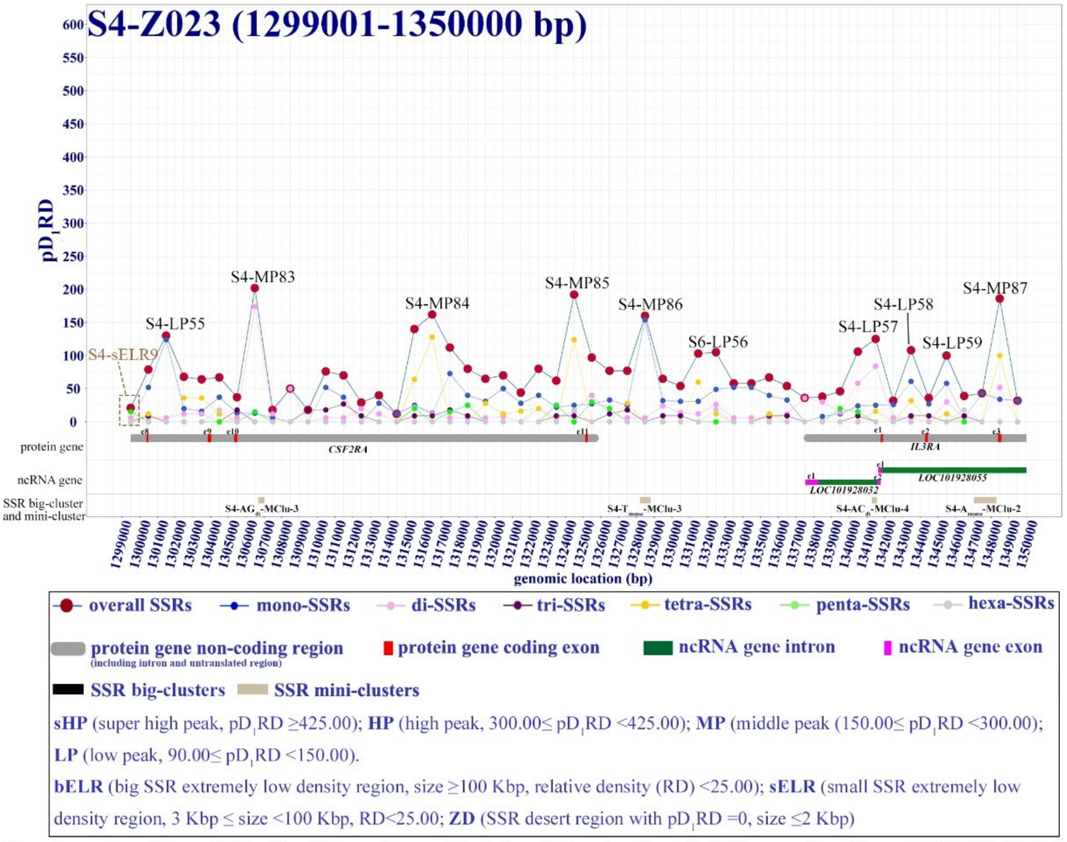

Supplementary Figure 1.26. The SSR position related D_1-relative density (pD_1RD) map of position at 1299001-1350000 bp of human reference Y-DNA (NC_000024.10) at resolution of 1 Kbp. .

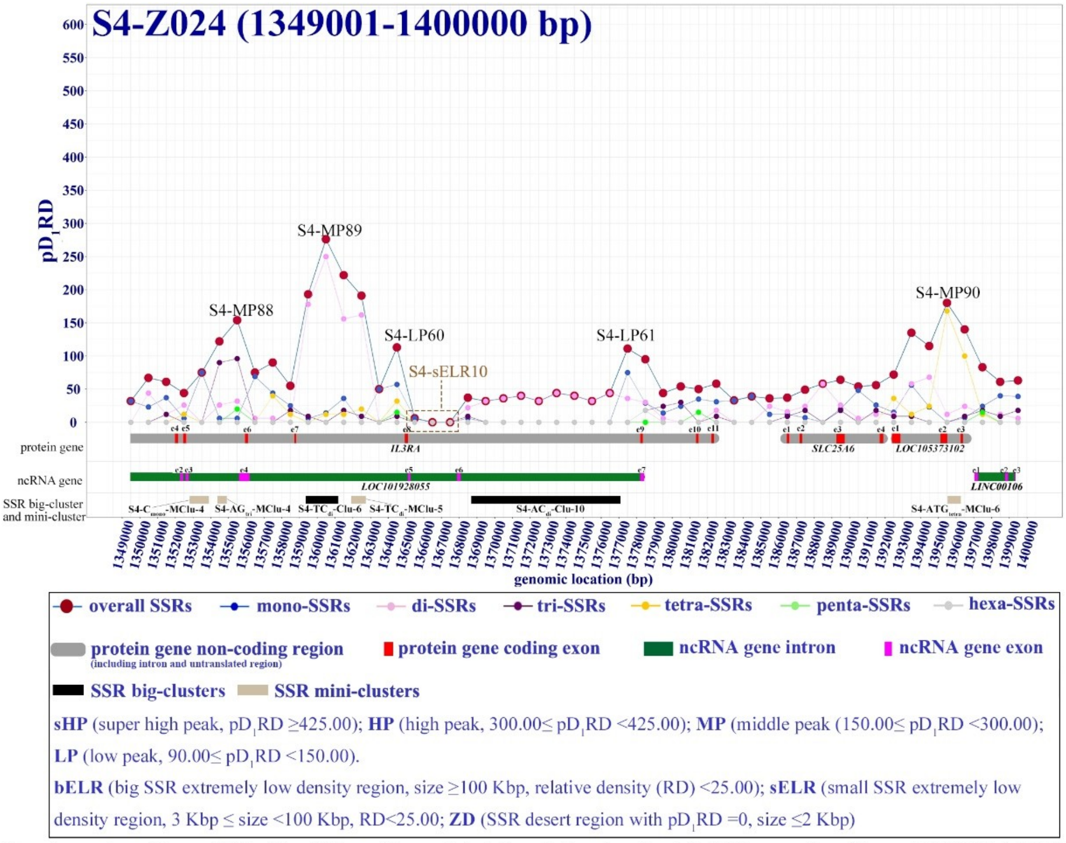

Supplementary Figure 1.27. The SSR position related D_1-relative density (pD_1RD) map of position at 1349001-1400000 bp of human reference Y-DNA (NC_000024.10) at resolution of 1 Kbp.

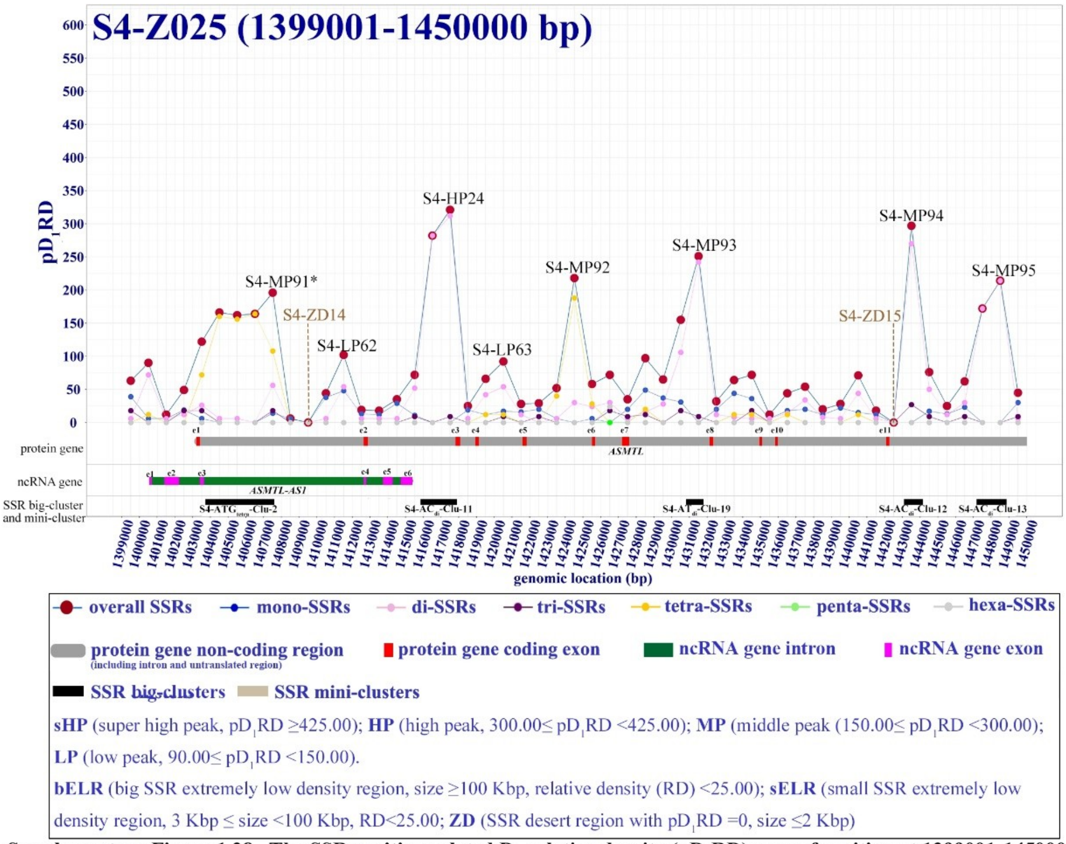

Supplementary Figure 1.28. The SSR position related D_1-relative density (pD_1RD) map of position at 1399001-1450000 bp of human reference Y-DNA (NC_000024.10) at resolution of 1 Kbp. * It was also marked as the SSR density peak.

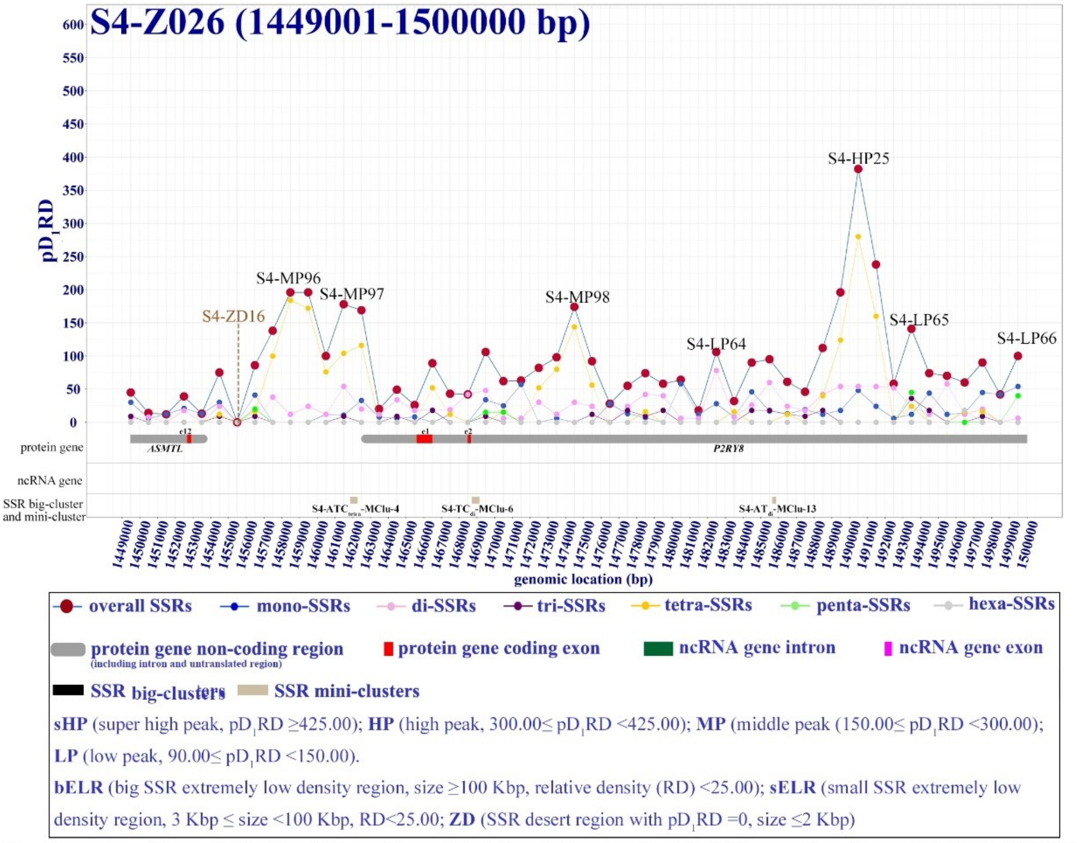

Supplementary Figure 1.29. The SSR position related D_1-relative density (pD_1RD) map of position at 1449001-1500000 bp of human reference Y-DNA (NC_000024.10) at resolution of 1 Kbp.

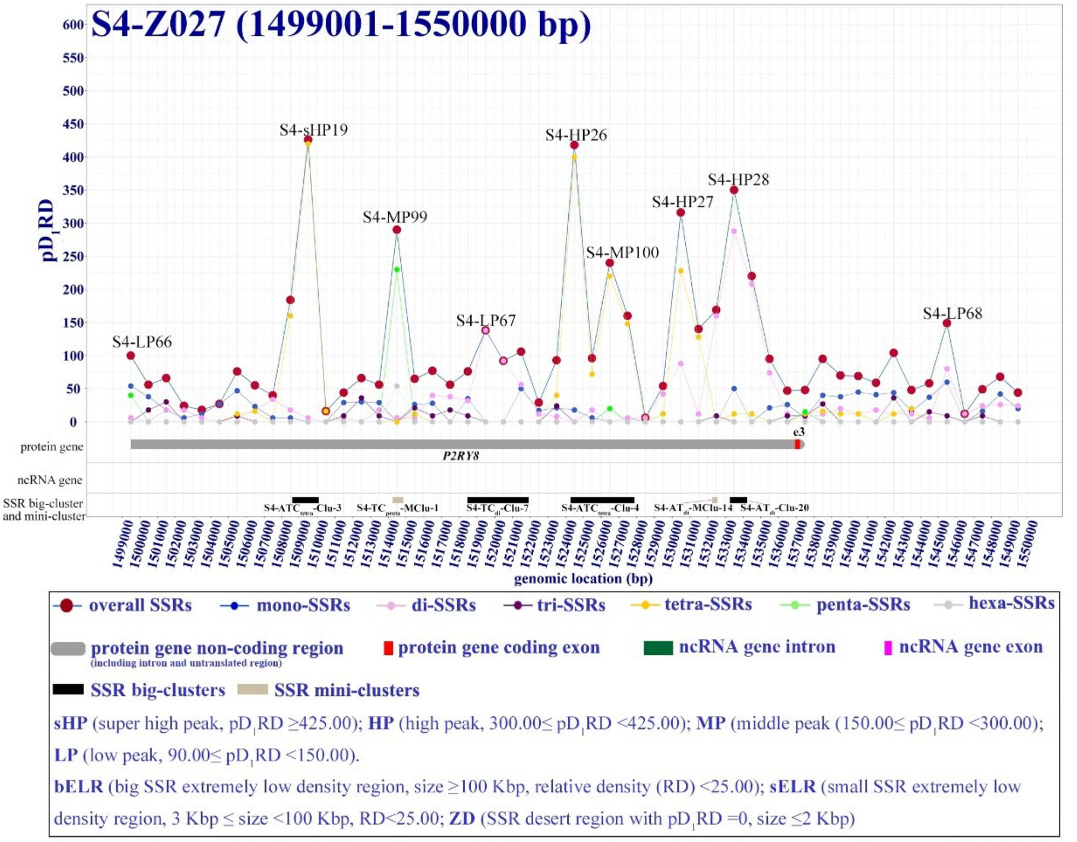

Supplementary Figure 1.30. The SSR position related D_1-relative density (pD_1RD) map of position at 1499001-1550000 bp of human reference Y-DNA (NC_000024.10) at resolution of 1 Kbp.

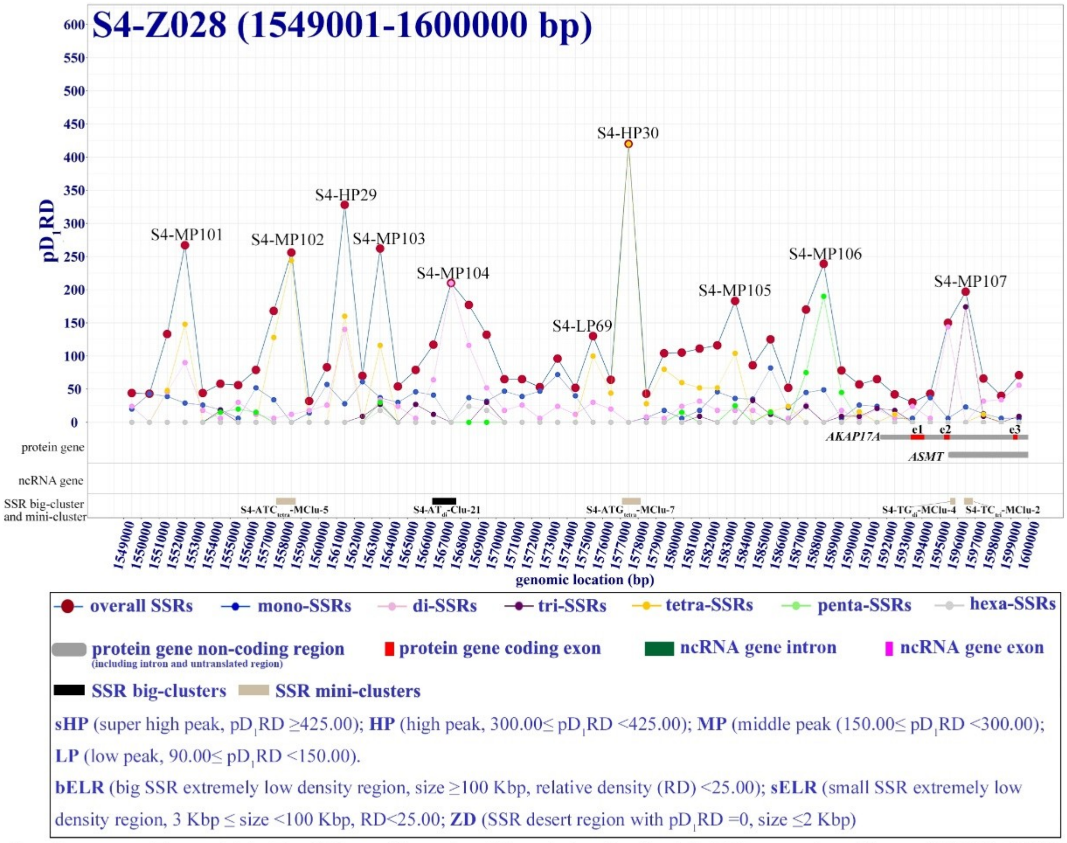

Supplementary Figure 1.31. The SSR position related D_1-relative density (pD_1RD) map of position at 1549001-1600000 bp of human reference Y-DNA (NC_000024.10) at resolution of 1 Kbp.

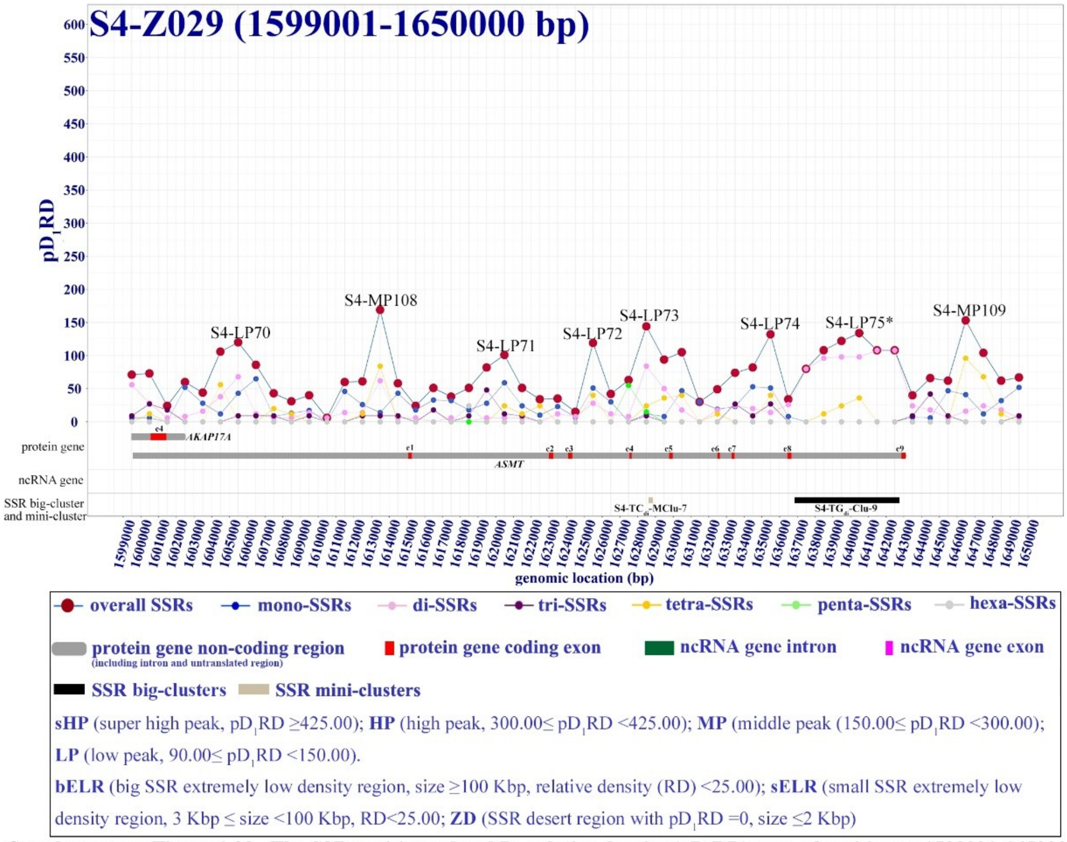

Supplementary Figure 1.32. The SSR position related D_1-relative density (pD_1RD) map of position at 1599001-1650000 bp of human reference Y-DNA (NC_000024.10) at resolution of 1 Kbp. *It was also marked as the density peak.

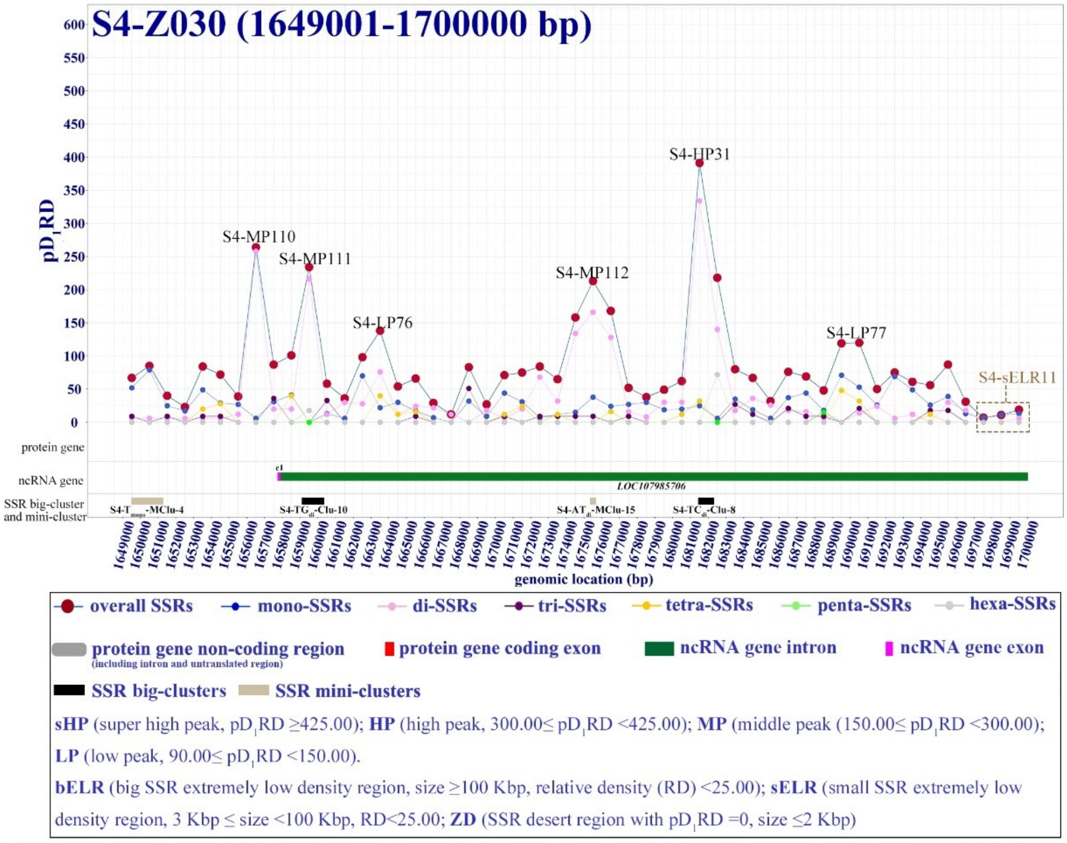

Supplementary Figure 1.33. The SSR position related D_1-relative density (pD_1RD) map of position at 1649001-1700000 bp of human reference Y-DNA (NC_000024.10) at resolution of 1 Kbp.

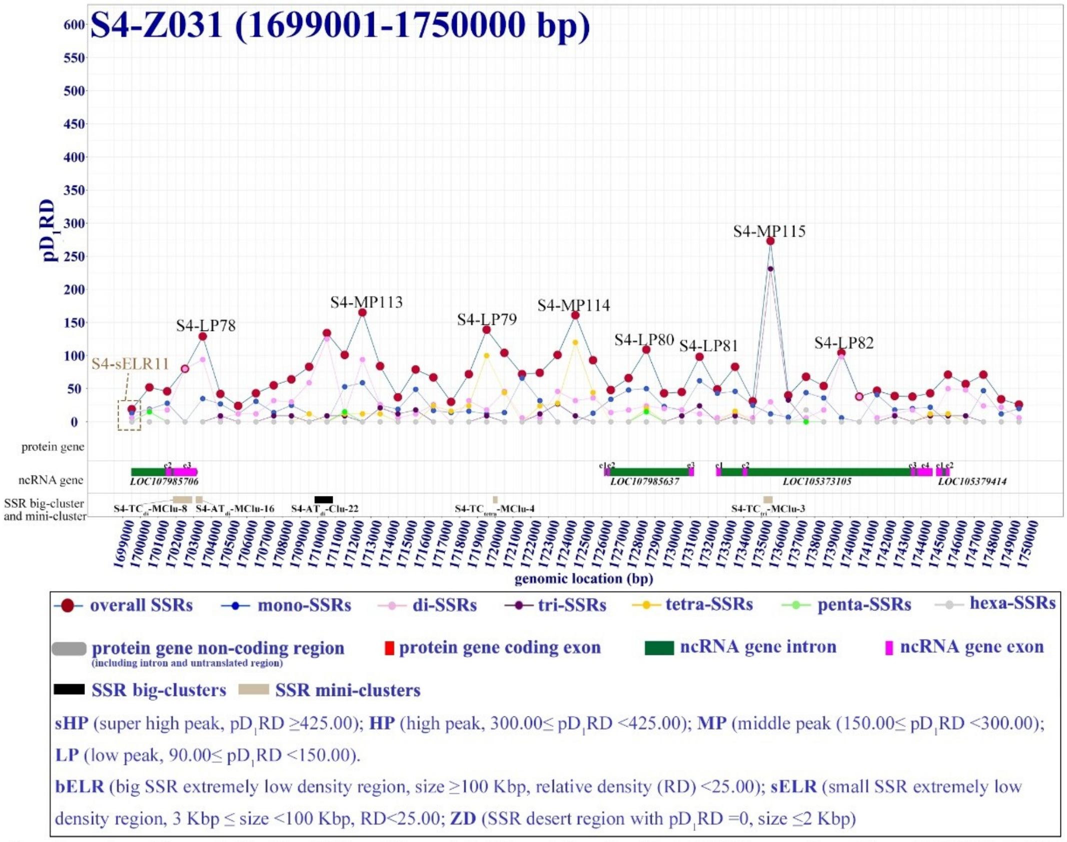

Supplementary Figure 1.34. The SSR position related D_1-relative density (pD_1RD) map of position at 1699001-1750000 bp of human reference Y-DNA (NC_000024.10) at resolution of 1 Kbp.

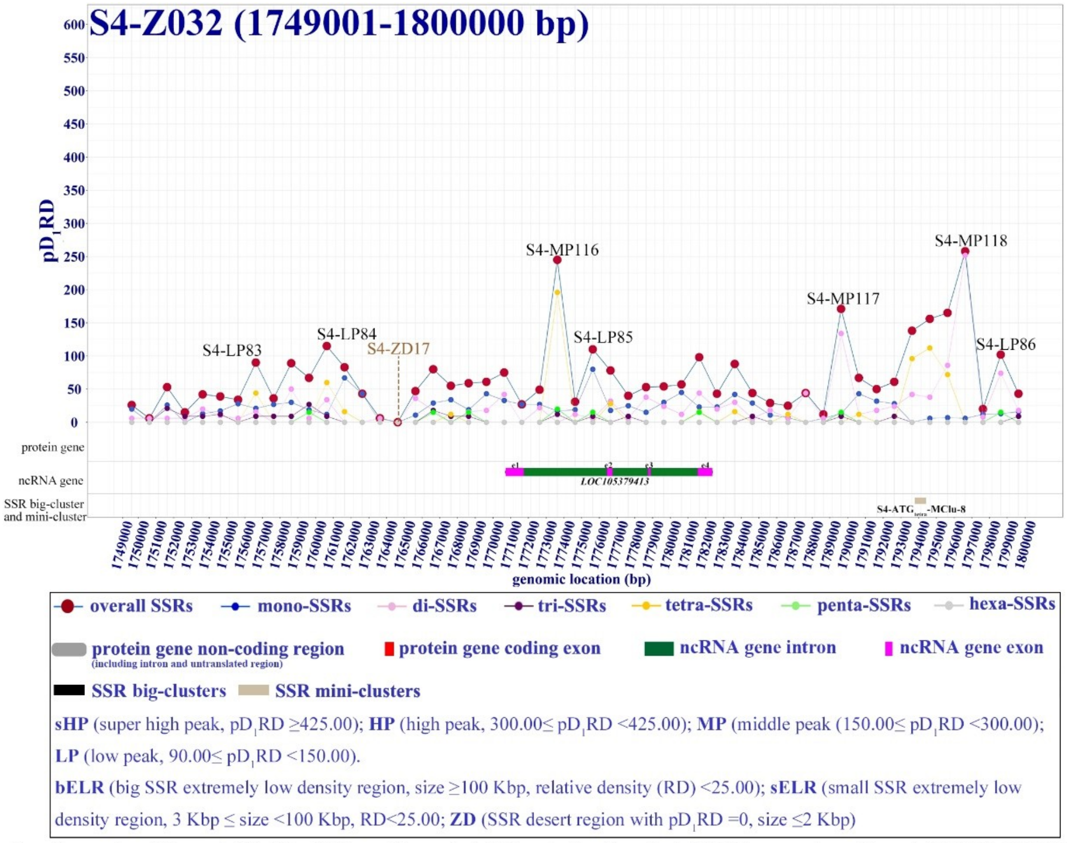

Supplementary Figure 1.35. The SSR position related D_1-relative density (pD_1RD) map of position at 1749001-1800000 bp of human reference Y-DNA (NC_000024.10) at resolution of 1 Kbp.

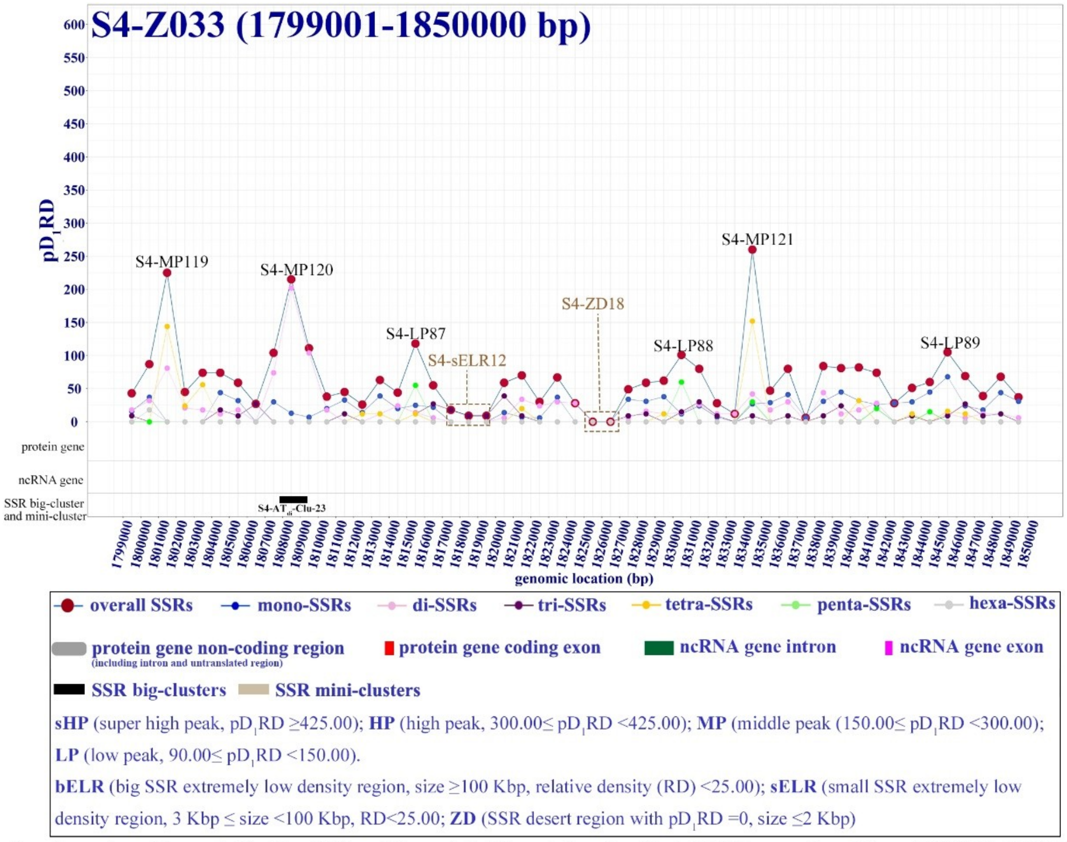

Supplementary Figure 1.36. The SSR position related D_1-relative density (pD_1RD) map of position at 1799001-1850000 bp of human reference Y-DNA (NC_000024.10) at resolution of 1 Kbp.

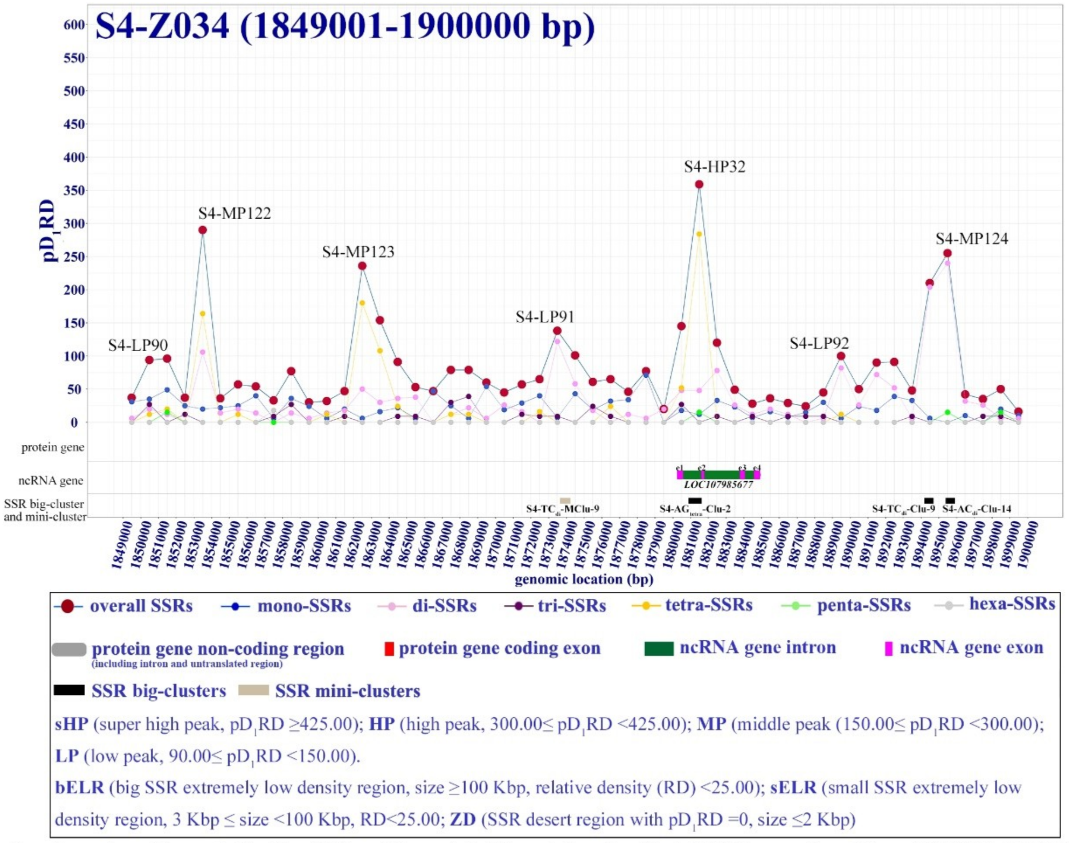

Supplementary Figure 1.37. The SSR position related D_1-relative density (pD_1RD) map of position at 1849001-1900000 bp of human reference Y-DNA (NC_000024.10) at resolution of 1 Kbp.

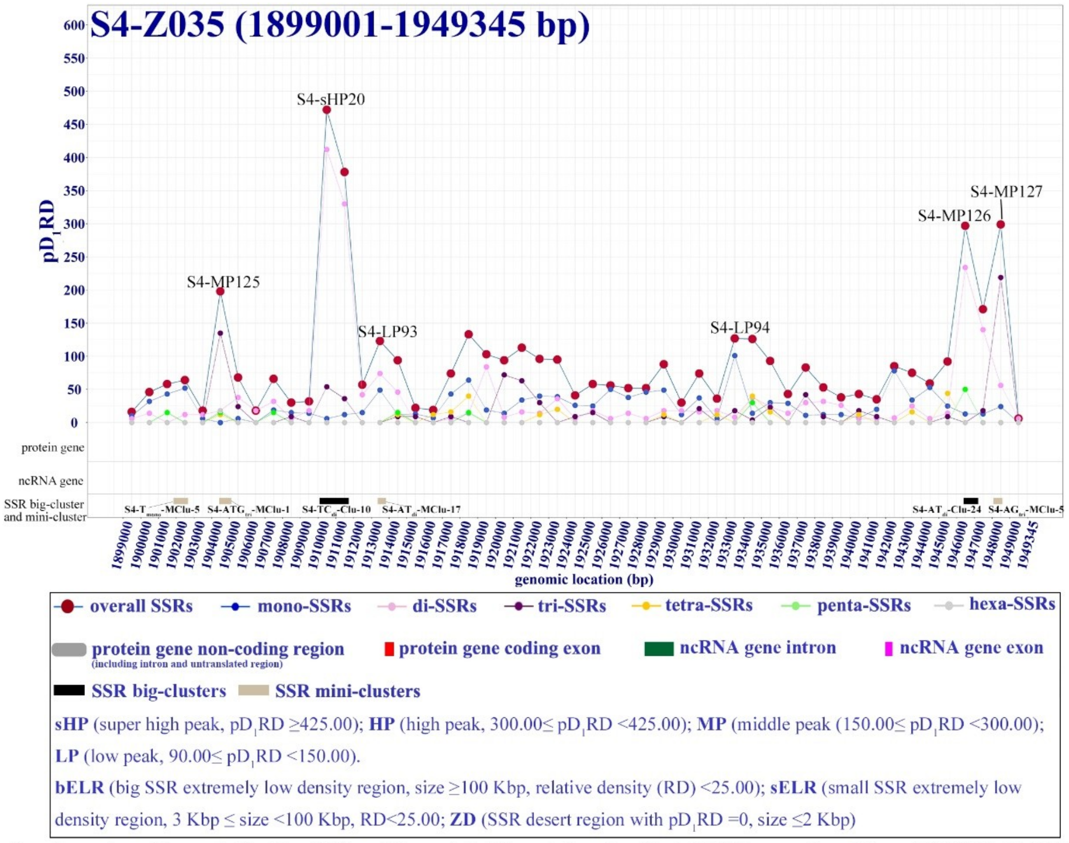

Supplementary Figure 1.38. The SSR position related D_1-relative density (pD_1RD) map of position at 1899001-1949345 bp (unnormal zone<51000 bp) of human reference Y-DNA (NC_000024.10) at resolution of 1 Kbp.

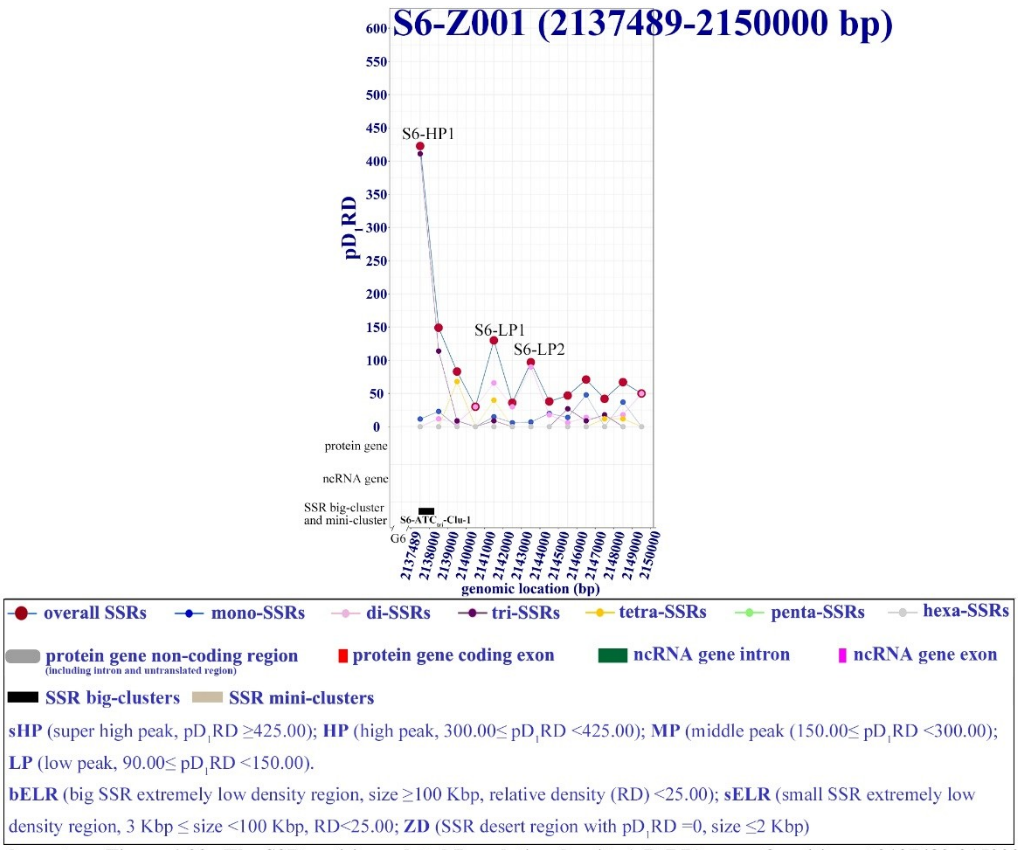

Supplementary Figure 1.39. The SSR position related D_1-relative density (pD_1RD) map of position at 2137489-2150000 bp of (unnormal zone <51000 bp) human reference Y-DNA (NC_000024.10) at resolution of 1 Kbp.

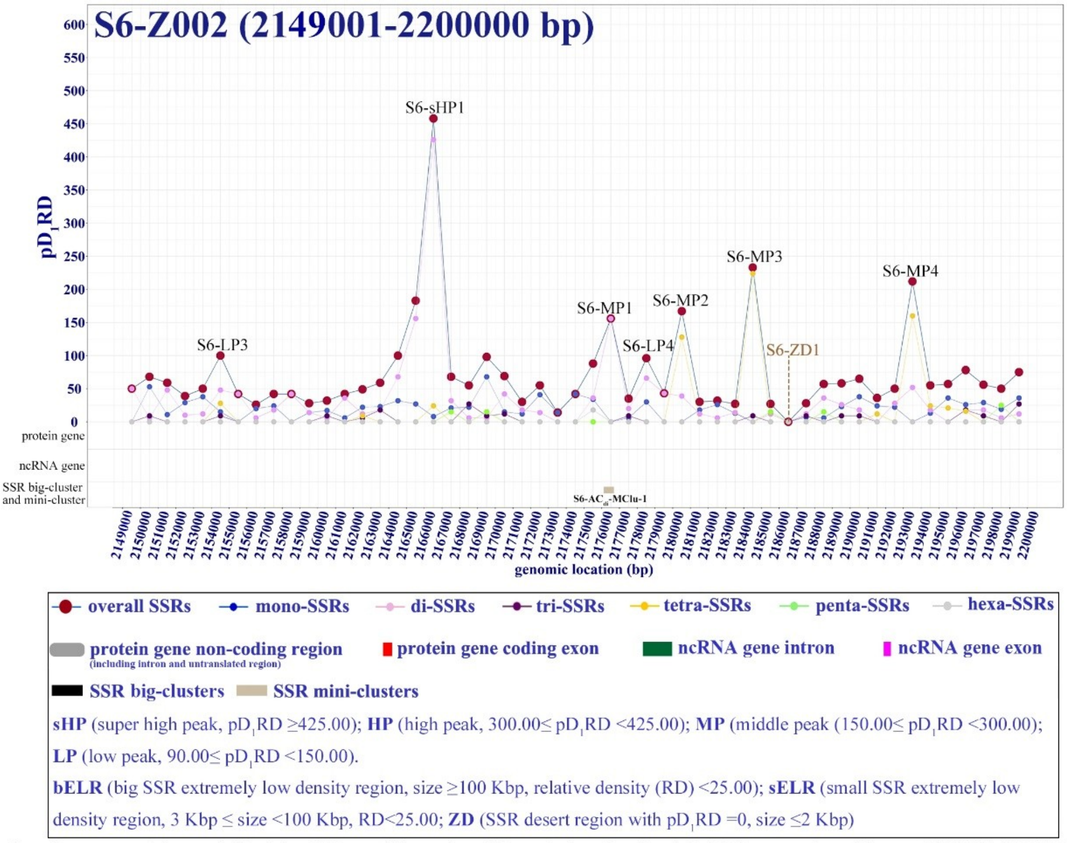

Supplementary Figure 1.40. The SSR position related D_1-relative density (pD_1RD) map of position at 2149001-2200000 bp of human reference Y-DNA (NC_000024.10) at resolution of 1 Kbp.

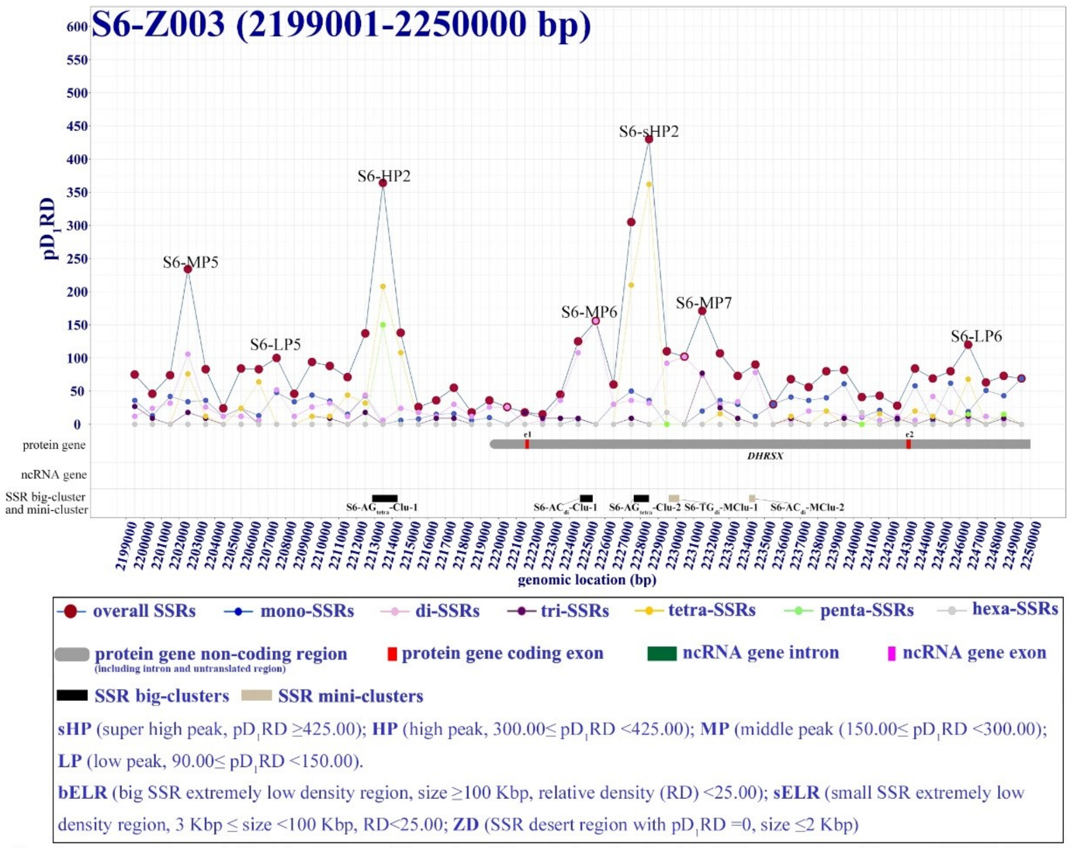

Supplementary Figure 1.41. The SSR position related D_1-relative density (pD_1RD) map of position at 2199001-2250000 bp of human reference Y-DNA (NC_000024.10) at resolution of 1 Kbp.

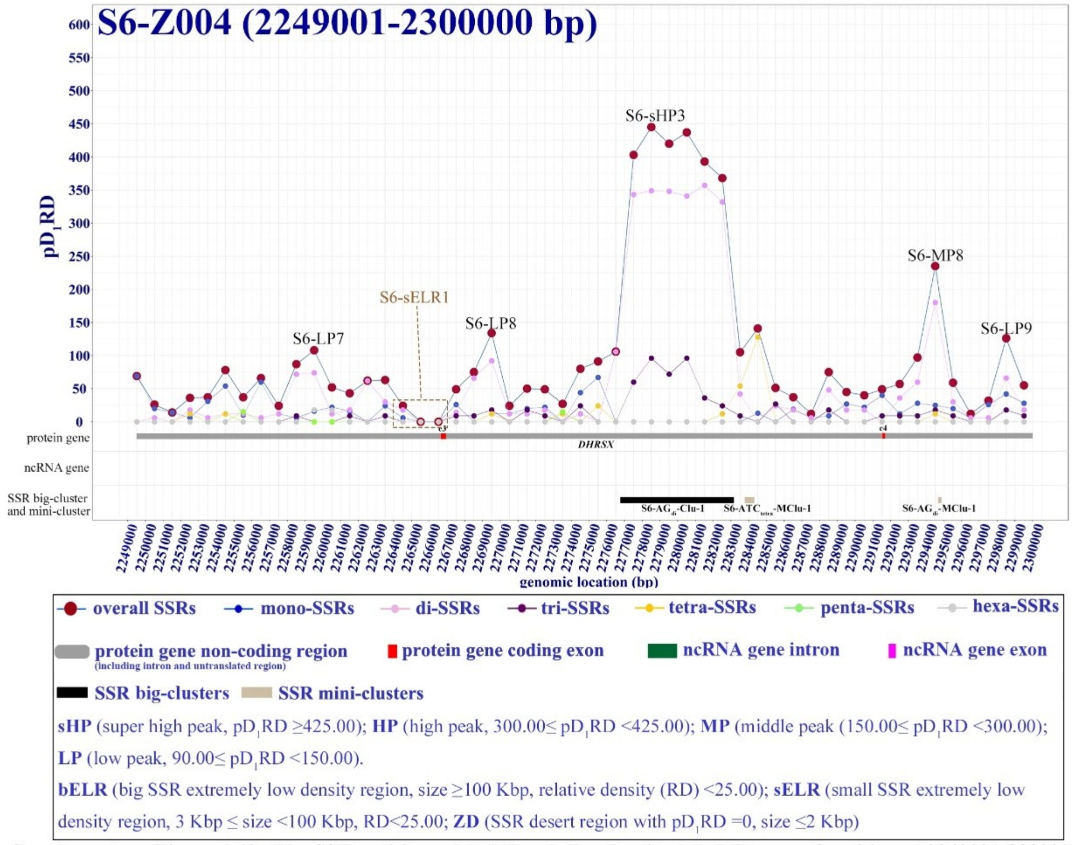

Supplementary Figure 1.42. The SSR position related D_1-relative density (pD_1RD) map of position at 2249001-2300000 bp of human reference Y-DNA (NC_000024.10) at resolution of 1 Kbp.

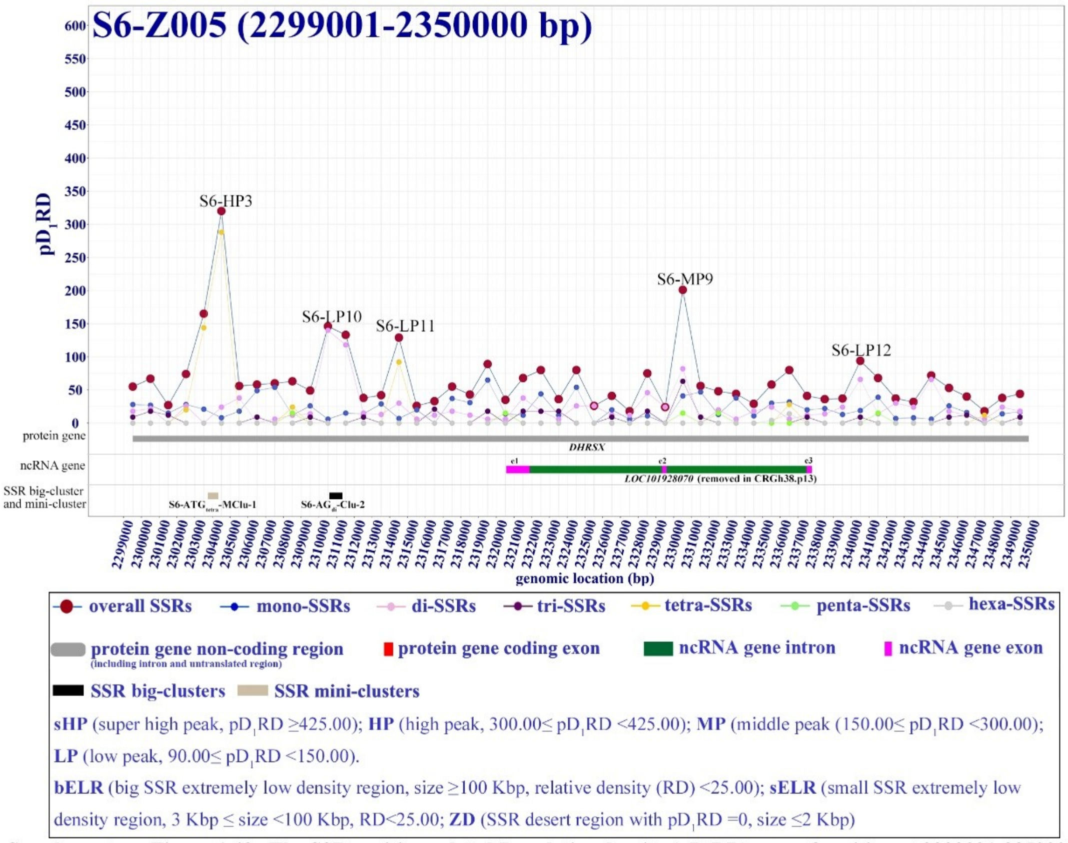

Supplementary Figure 1.43. The SSR position related D_1-relative density (pD_1RD) map of position at 2299001-2350000 bp of human reference Y-DNA (NC_000024.10) at resolution of 1 Kbp.

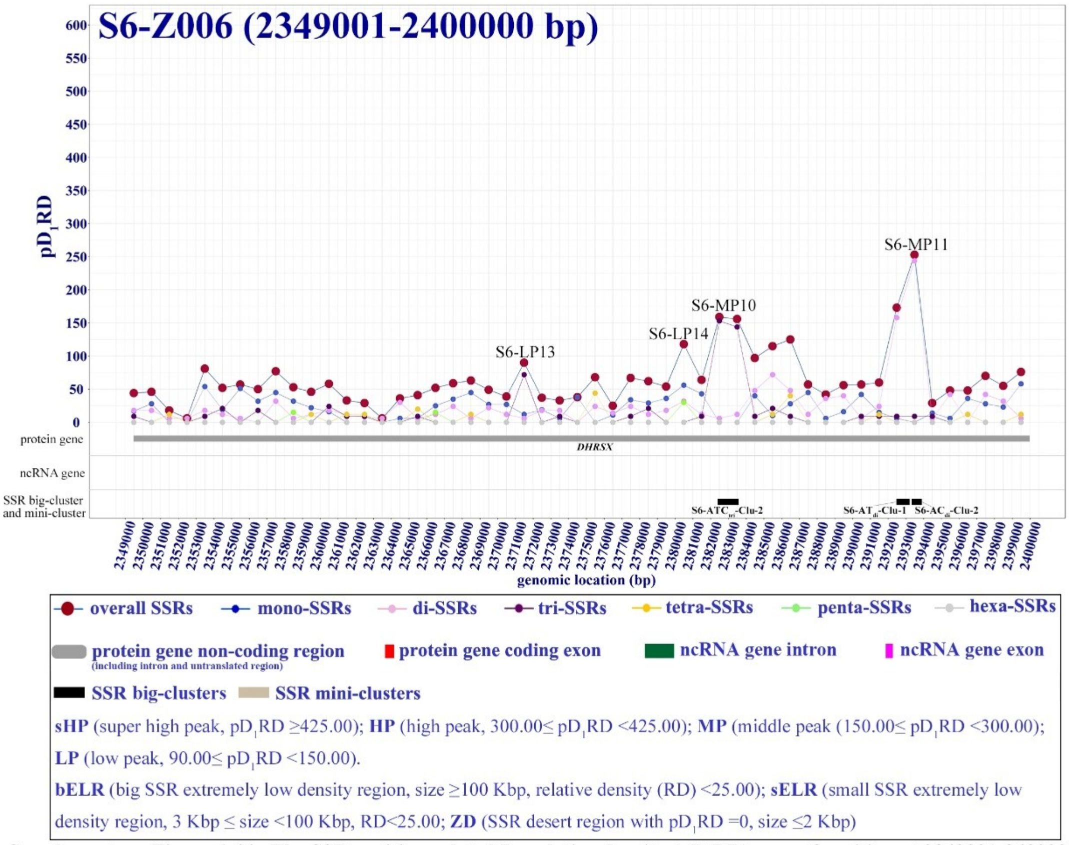

Supplementary Figure 1.44. The SSR position related D_1-relative density (pD_1RD) map of position at 2349001-2400000 bp of human reference Y-DNA (NC_000024.10) at resolution of 1 Kbp.

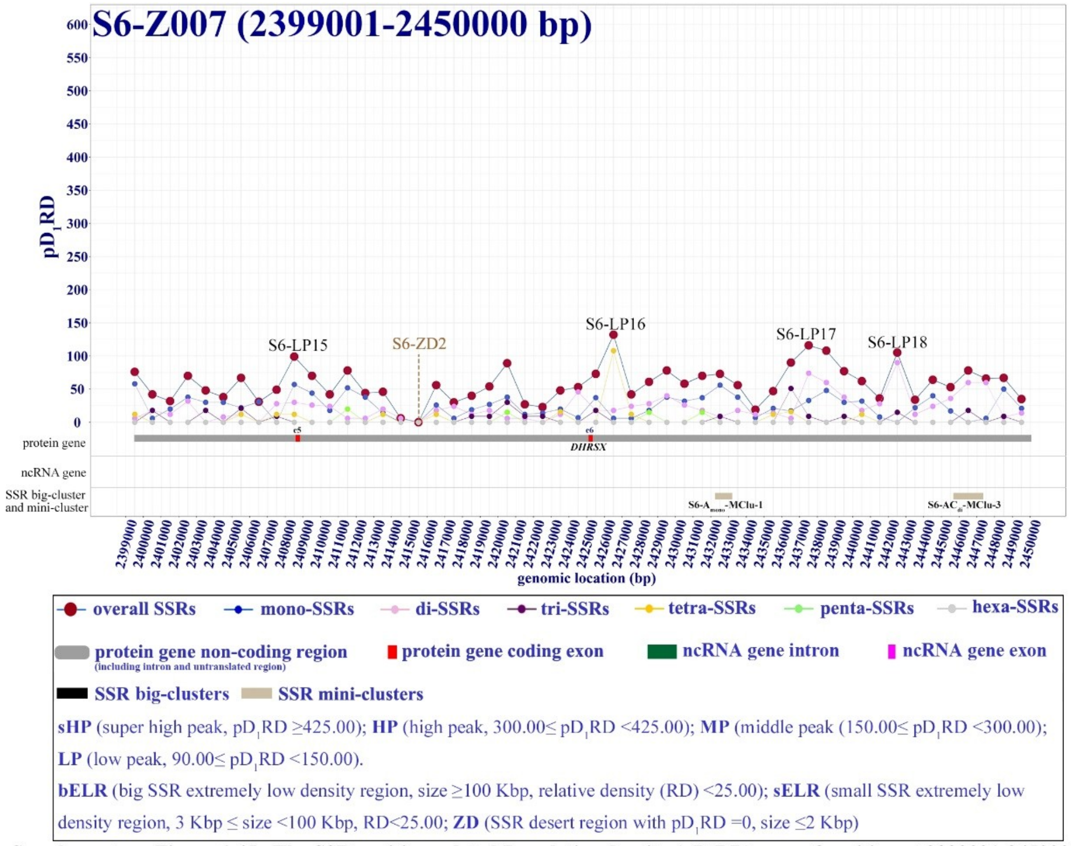

Supplementary Figure 1.45. The SSR position related D_1-relative density (pD_1RD) map of position at 2399001-2450000 bp of human reference Y-DNA (NC_000024.10) at resolution of 1 Kbp.

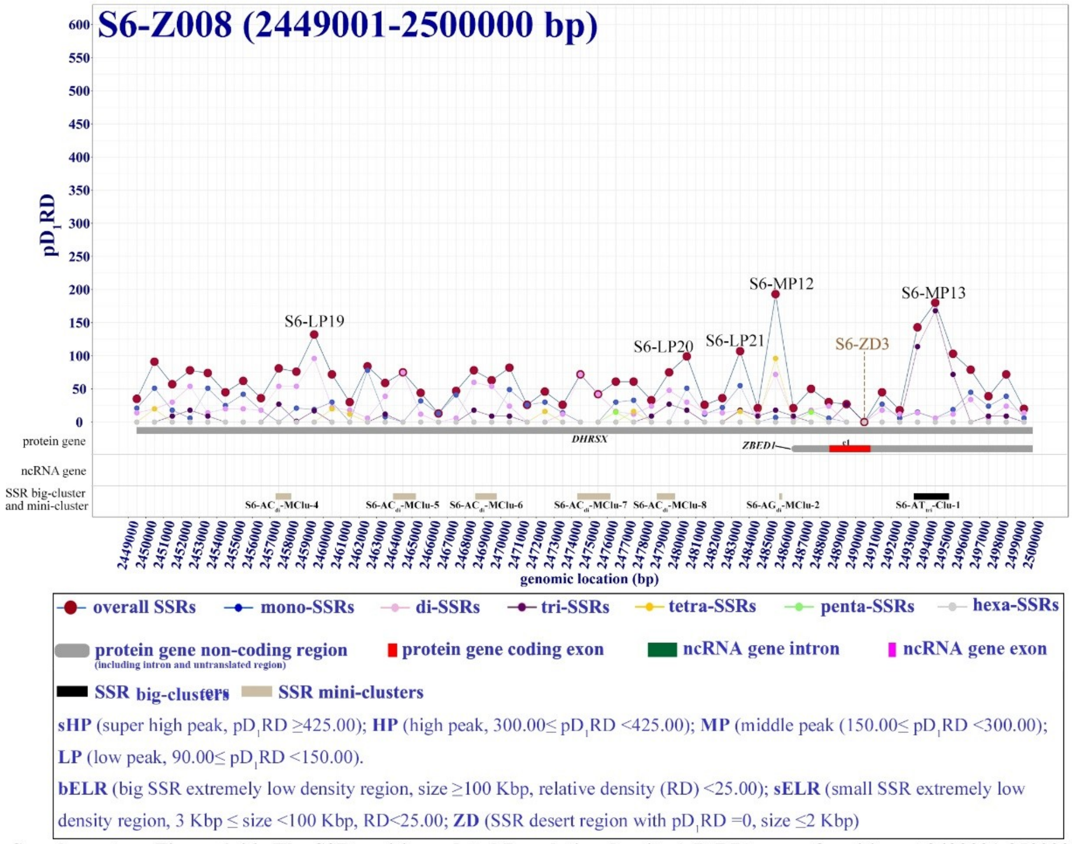

Supplementary Figure 1.46. The SSR position related D_1-relative density (pD_1RD) map of position at 2499001-2500000 bp of human reference Y-DNA (NC_000024.10) at resolution of 1 Kbp.

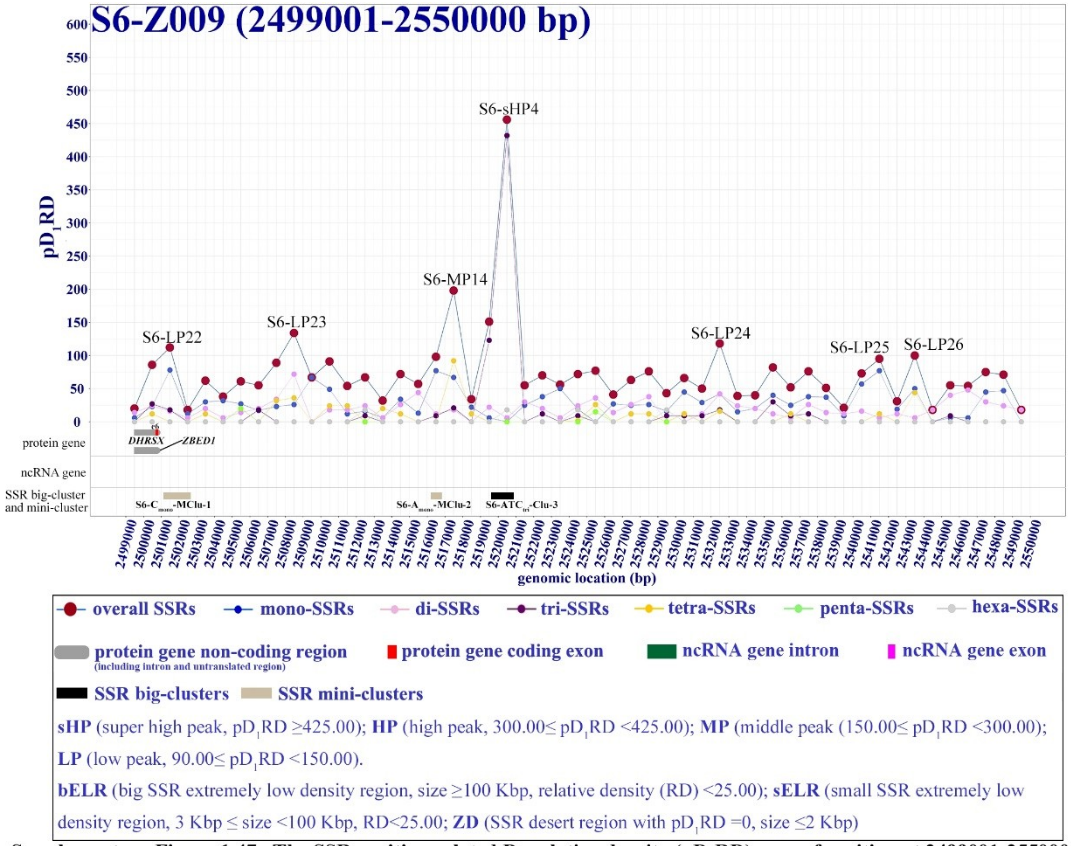

Supplementary Figure 1.47. The SSR position related D_1-relative density (pD_1RD) map of position at 2499001-2550000 bp of human reference Y-DNA (NC_000024.10) at resolution of 1 Kbp.

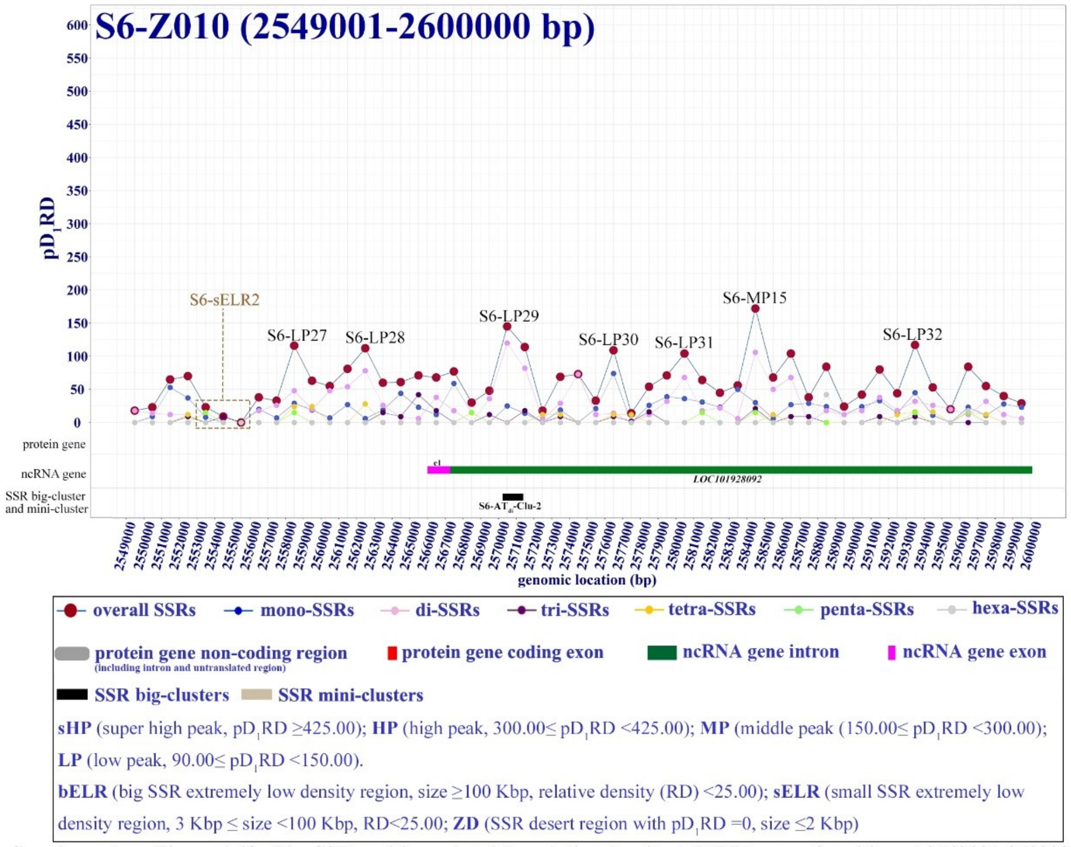

Supplementary Figure 1.48. The SSR position related D_1-relative density (pD_1RD) map of position at 2549001-2600000 bp of human reference Y-DNA (NC_000024.10) at resolution of 1 Kbp.

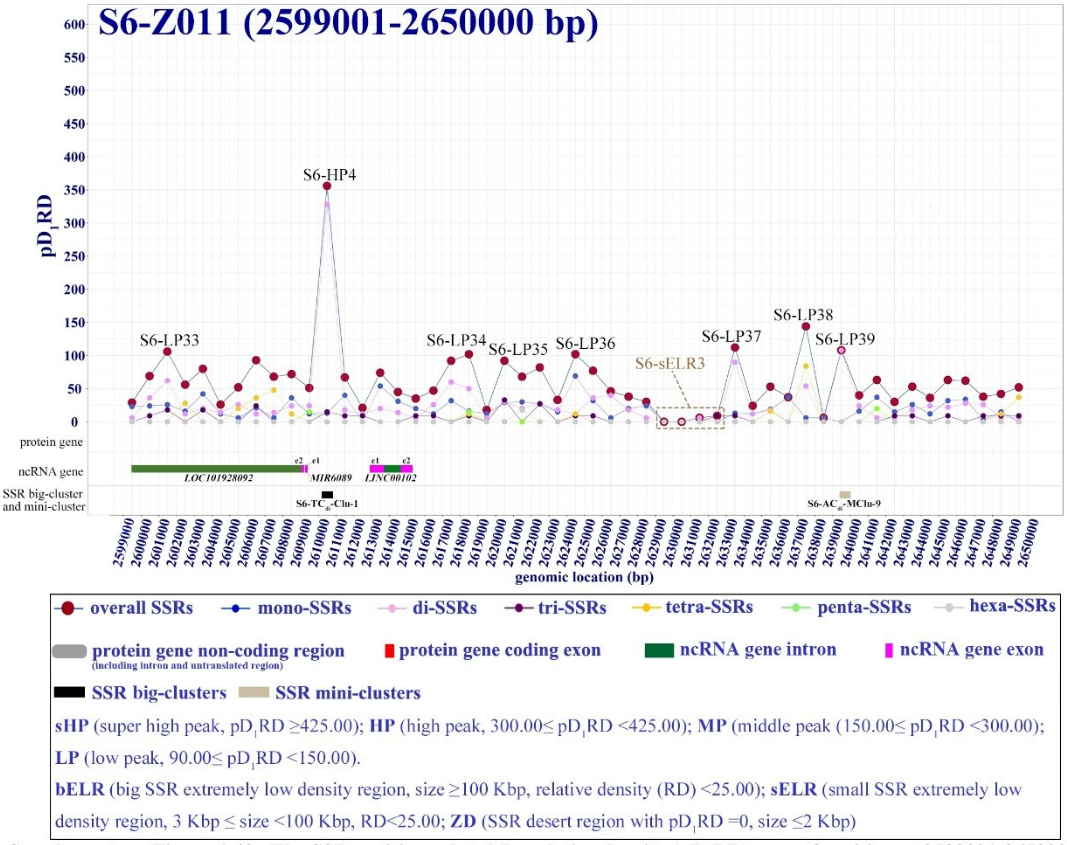

Supplementary Figure 1.49. The SSR position related D_1-relative density (pD_1RD) map of position at 2599001-2650000 bp of human reference Y-DNA (NC_000024.10) at resolution of 1 Kbp.

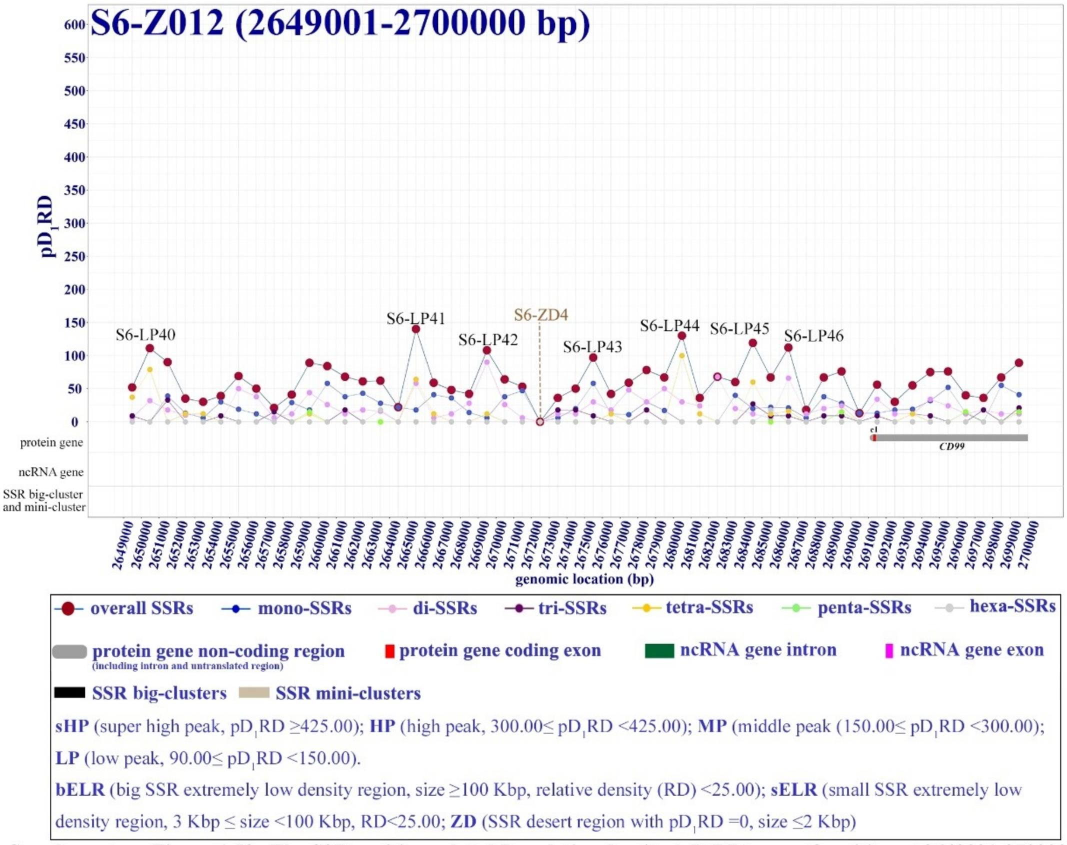

Supplementary Figure 1.50. The SSR position related D_1-relative density (pD_1RD) map of position at 2649001-2700000 bp of human reference Y-DNA (NC_000024.10) at resolution of 1 Kbp.

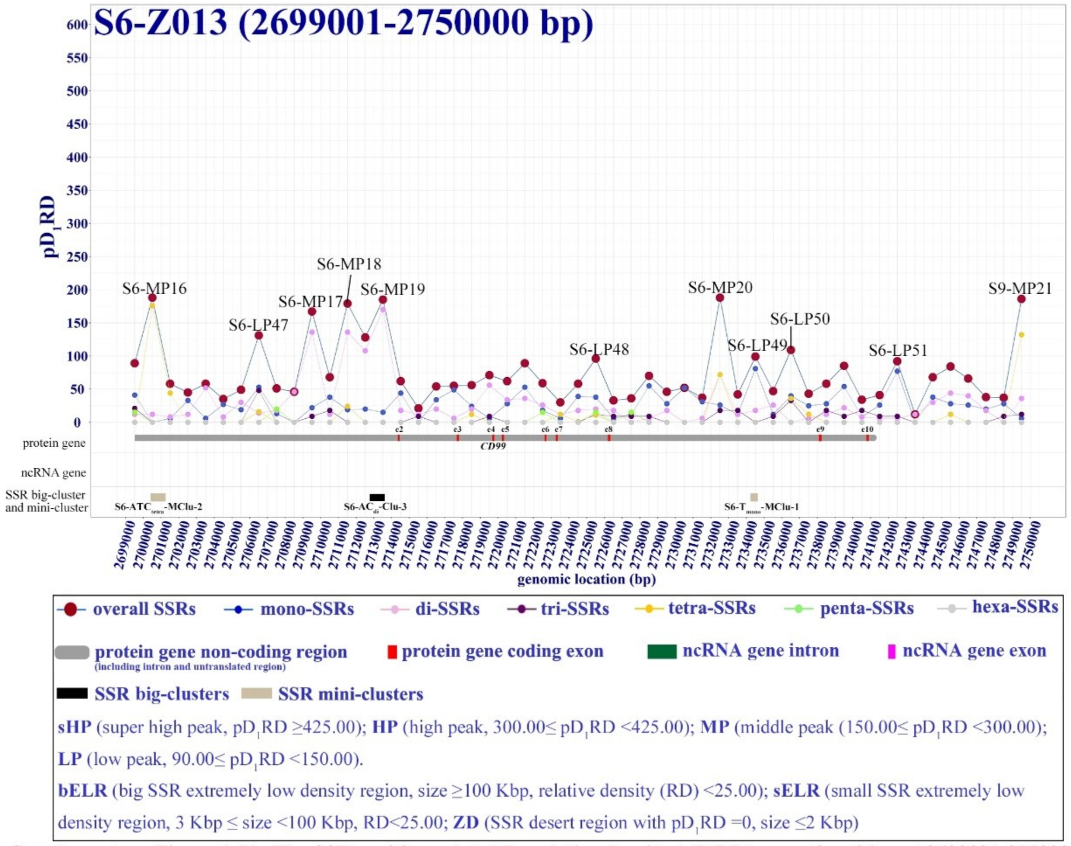

Supplementary Figure 1.51. The SSR position related D_1-relative density (pD_1RD) map of position at 2699001-2750000 bp of human reference Y-DNA (NC_000024.10) at resolution of 1 Kbp.

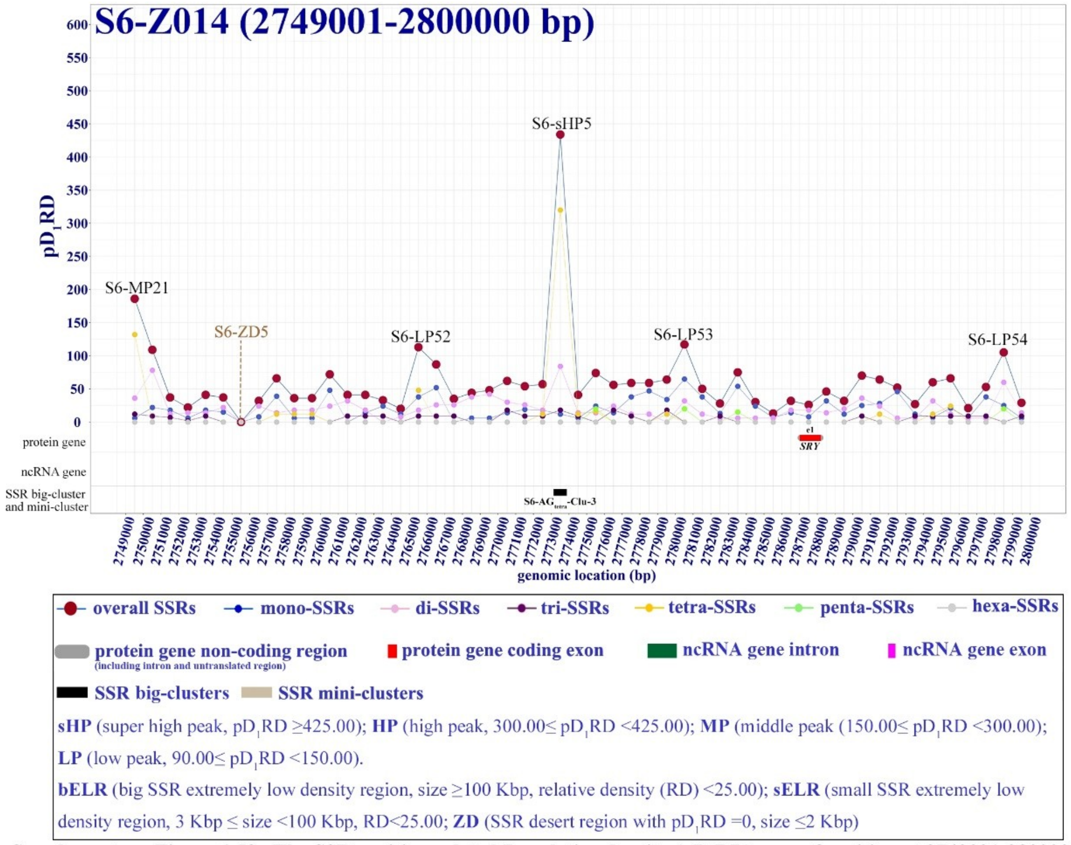

Supplementary Figure 1.52. The SSR position related D_1-relative density (pD_1RD) map of position at 2749001-2800000 bp of human reference Y-DNA (NC_000024.10) at resolution of 1 Kbp.

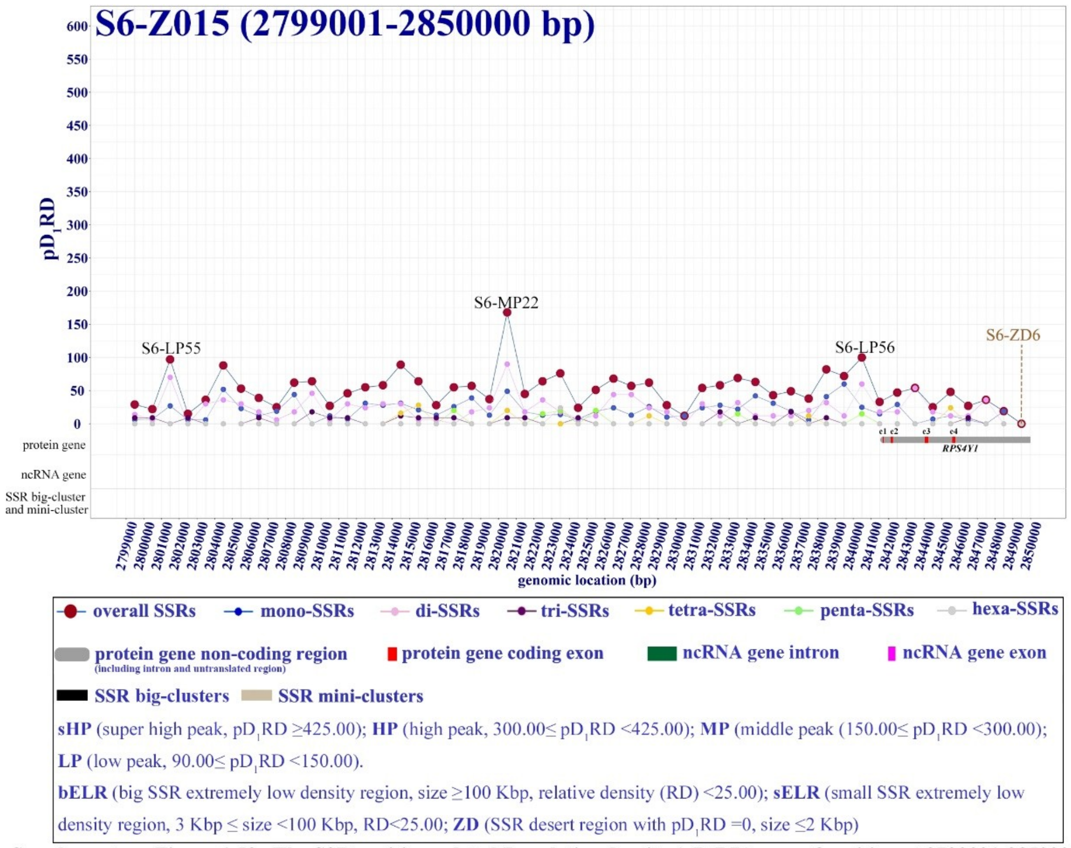

Supplementary Figure 1.53. The SSR position related D_1-relative density (pD_1RD) map of position at 2799001-2850000 bp of human reference Y-DNA (NC_000024.10) at resolution of 1 Kbp.

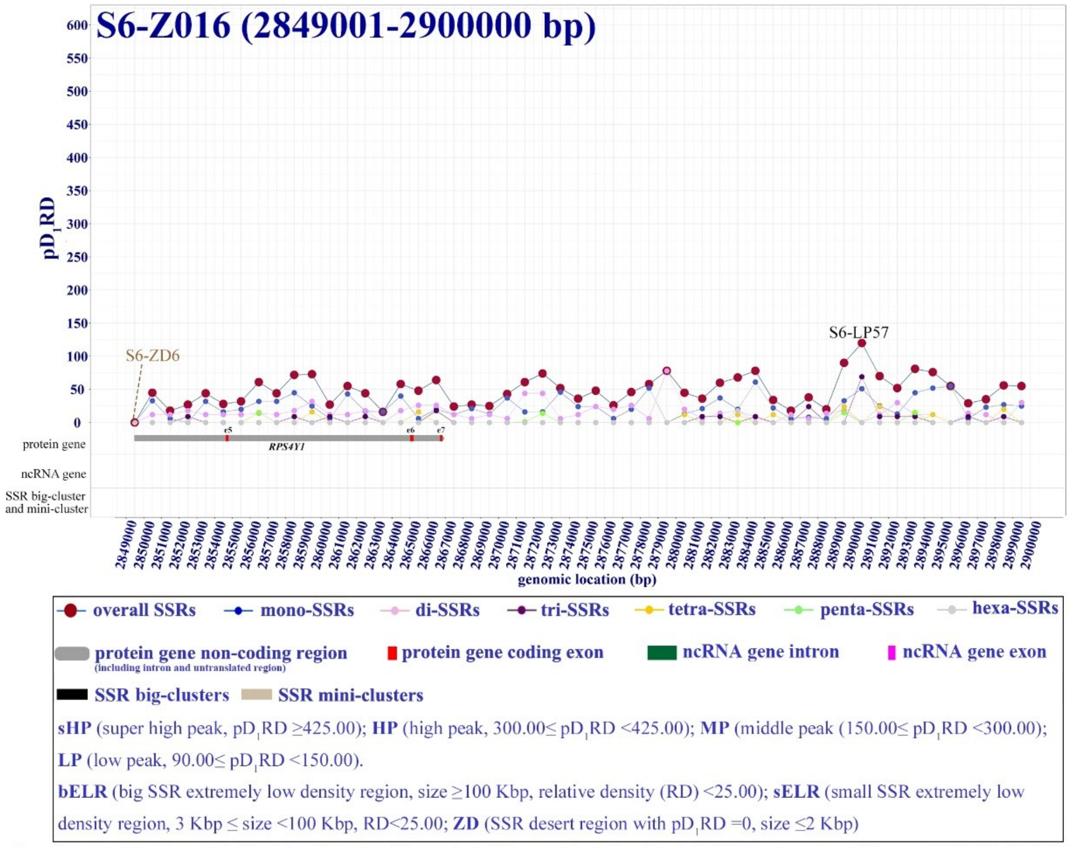

Supplementary Figure 1.54. The SSR position related D_1-relative density (pD_1RD) map of position at 2849001-2900000 bp of human reference Y-DNA (NC_000024.10) at resolution of 1 Kbp.

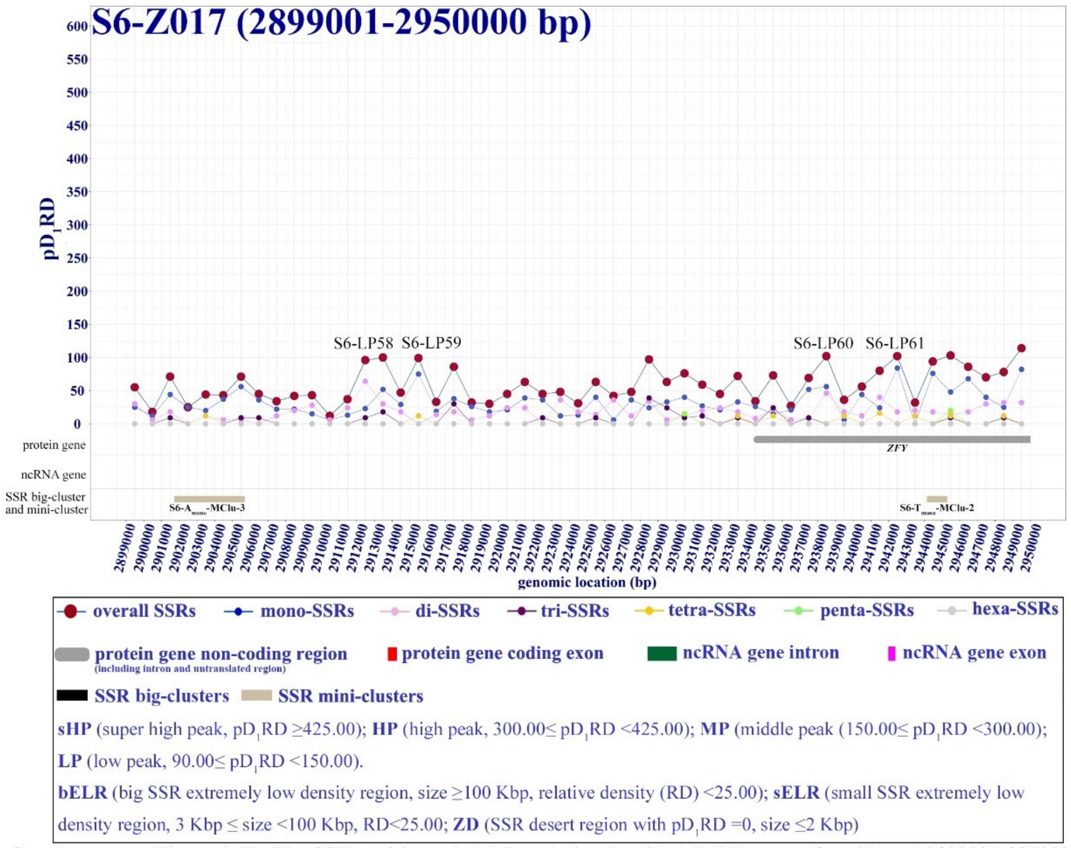

Supplementary Figure 1.55. The SSR position related D_1-relative density (pD_1RD) map of position at 2899001-2950000 bp of human reference Y-DNA (NC_000024.10) at resolution of 1 Kbp.

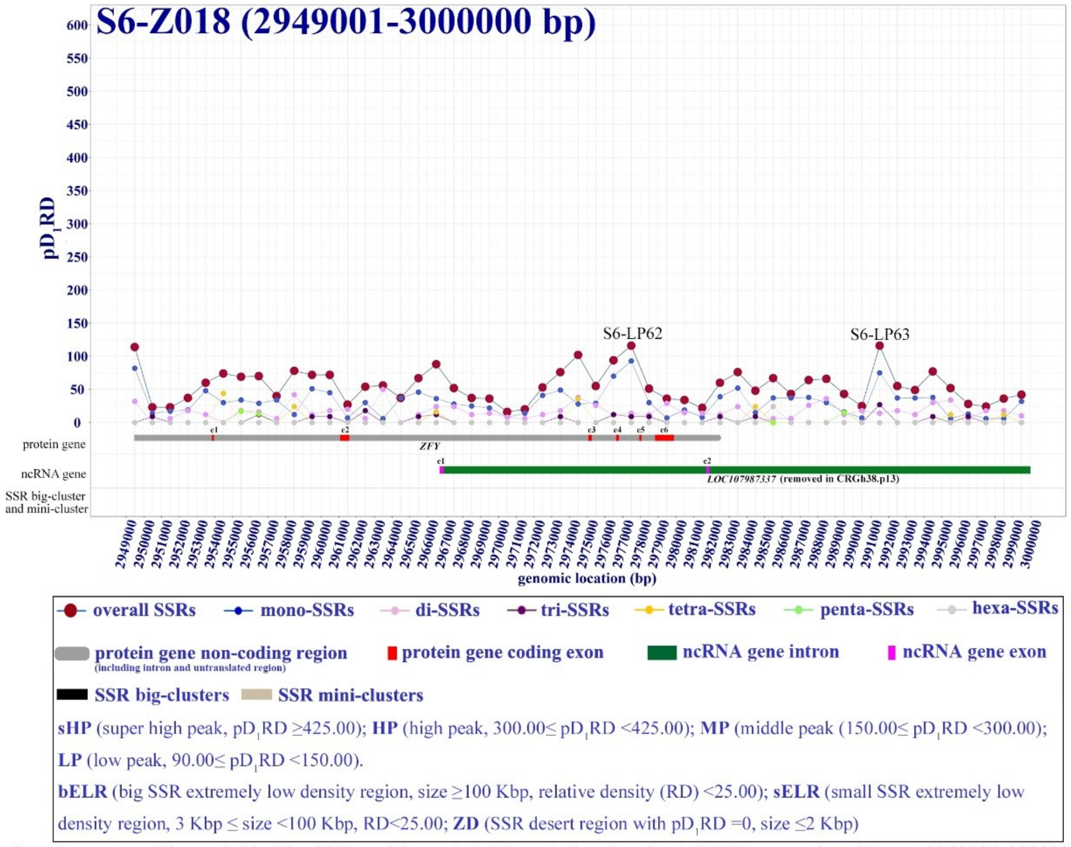

Supplementary Figure 1.56. The SSR position related D_1-relative density (pD_1RD) map of position at 2949001-3000000 bp of human reference Y-DNA (NC_000024.10) at resolution of 1 Kbp.

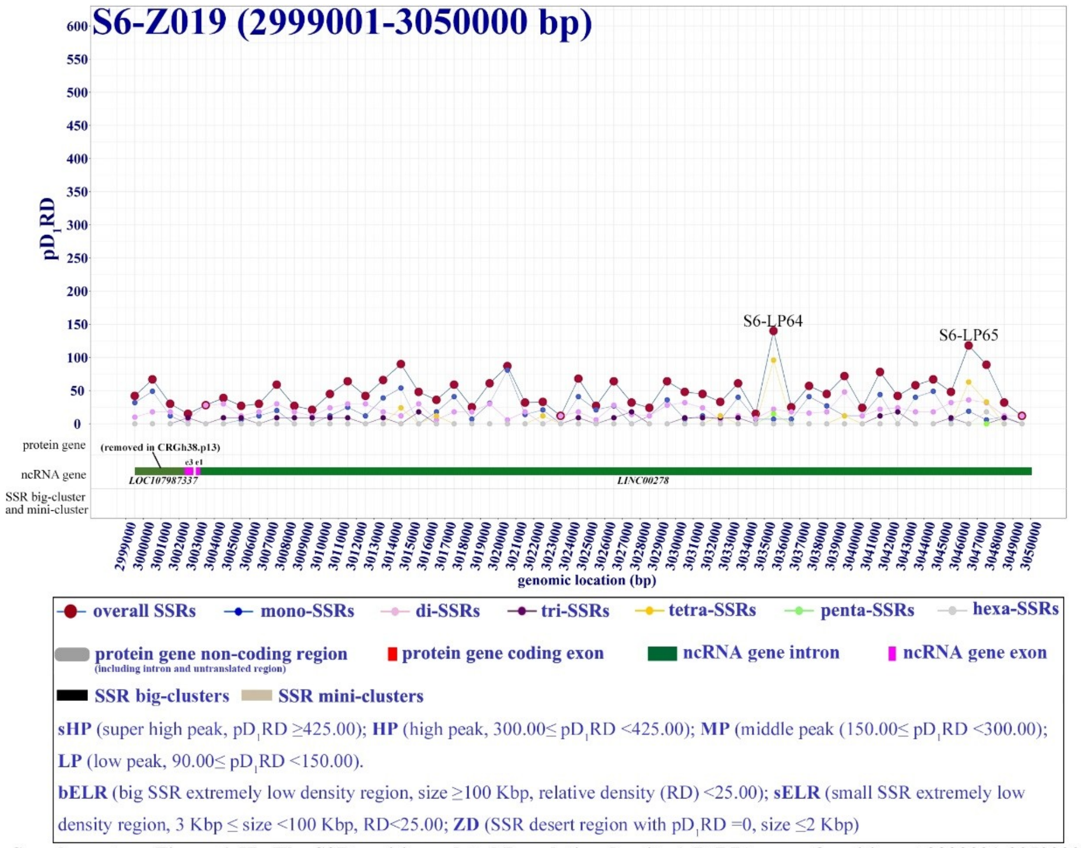

Supplementary Figure 1.57. The SSR position related D_1-relative density (pD_1RD) map of position at 2999001-3050000 bp of human reference Y-DNA (NC_000024.10) at resolution of 1 Kbp.

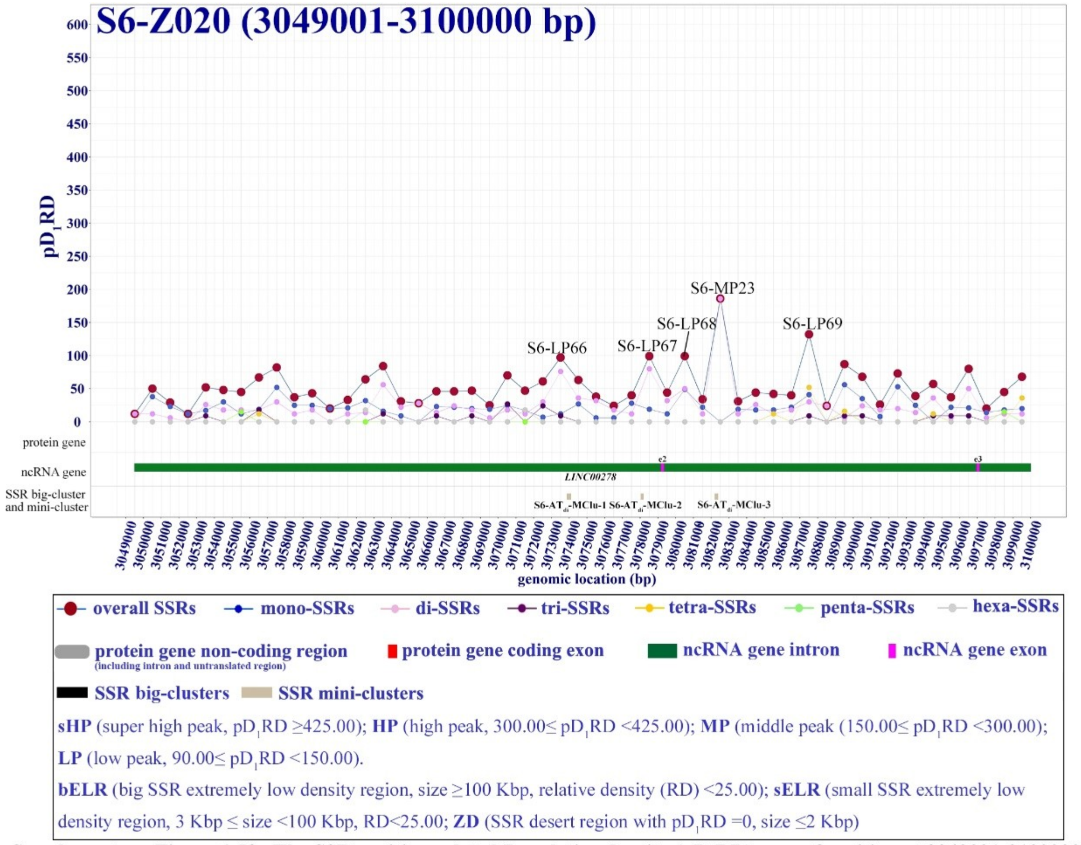

Supplementary Figure 1.58. The SSR position related D_1-relative density (pD_1RD) map of position at 3049001-3100000 bp of human reference Y-DNA (NC_000024.10) at resolution of 1 Kbp.

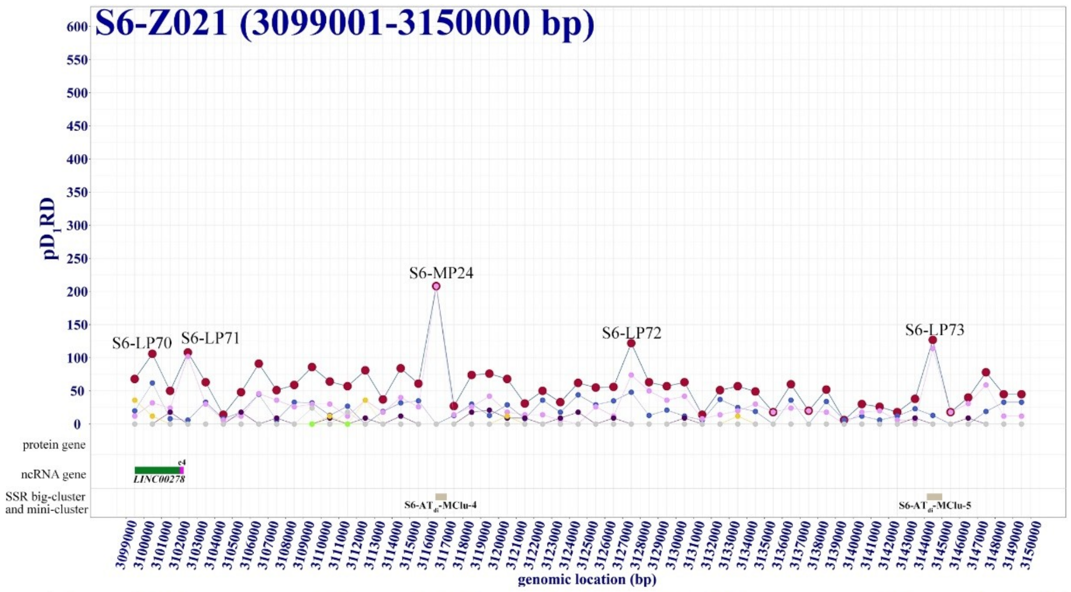

overall SSRs — mono-SSRs — di-SSRs — tri-SSRs — tetra-SSRs — penta-SSRs — hexa-SSRs

protein gene non-coding region (including intron and untranslated region) — protein gene coding exon — ncRNA gene intron — ncRNA gene exon

SSR big-clusters — SSR mini-clusters

sHP (super high peak, $pD_1RD \geq 425.00$); **HP** (high peak, $300.00 \leq pD_1RD < 425.00$); **MP** (middle peak ($150.00 \leq pD_1RD < 300.00$); **LP** (low peak, $90.00 \leq pD_1RD < 150.00$).

bELR (big SSR extremely low density region, size ≥100 Kbp, relative density (RD) <25.00); **sELR** (small SSR extremely low density region, 3 Kbp ≤ size <100 Kbp, RD<25.00; **ZD** (SSR desert region with pD_1RD =0, size ≤2 Kbp)

Supplementary Figure 1.59. The SSR position related D_1-relative density (pD_1RD) map of position at 3099001-3150000 bp of human reference Y-DNA (NC_000024.10) at resolution of 1 Kbp.

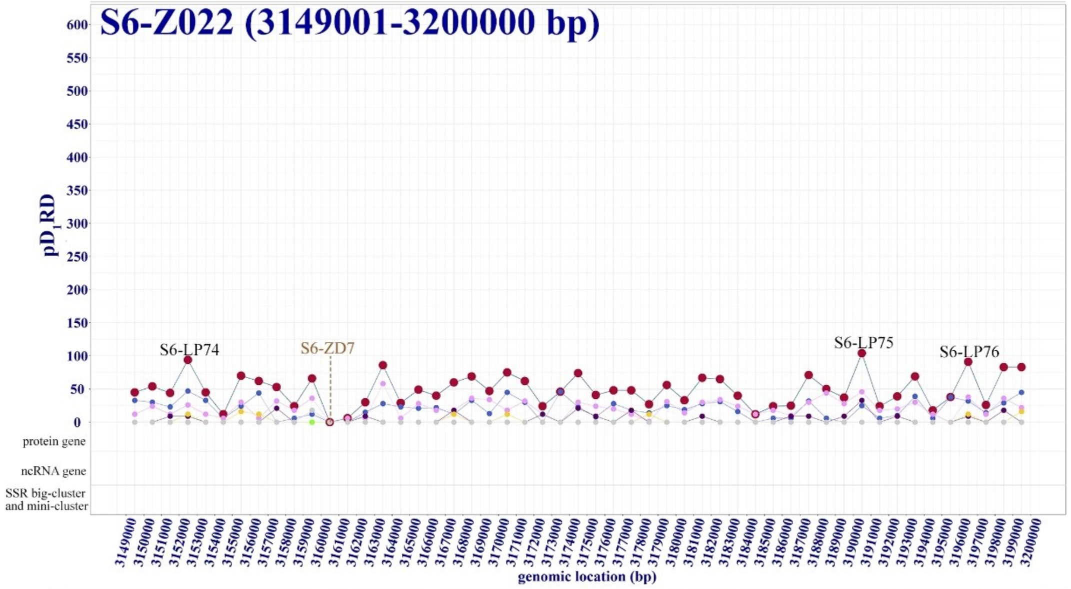

overall SSRs — mono-SSRs — di-SSRs — tri-SSRs — tetra-SSRs — penta-SSRs — hexa-SSRs

protein gene non-coding region (including intron and untranslated region) — protein gene coding exon — ncRNA gene intron — ncRNA gene exon

SSR big-clusters — SSR mini-clusters

sHP (super high peak, $pD_1RD \geq 425.00$); **HP** (high peak, $300.00 \leq pD_1RD < 425.00$); **MP** (middle peak ($150.00 \leq pD_1RD < 300.00$); **LP** (low peak, $90.00 \leq pD_1RD < 150.00$).

bELR (big SSR extremely low density region, size ≥100 Kbp, relative density (RD) <25.00); **sELR** (small SSR extremely low density region, 3 Kbp ≤ size <100 Kbp, RD<25.00; **ZD** (SSR desert region with pD_1RD =0, size ≤2 Kbp)

Supplementary Figure 1.60. The SSR position related D_1-relative density (pD_1RD) map of position at 3149001-3200000 bp of human reference Y-DNA (NC_000024.10) at resolution of 1 Kbp.

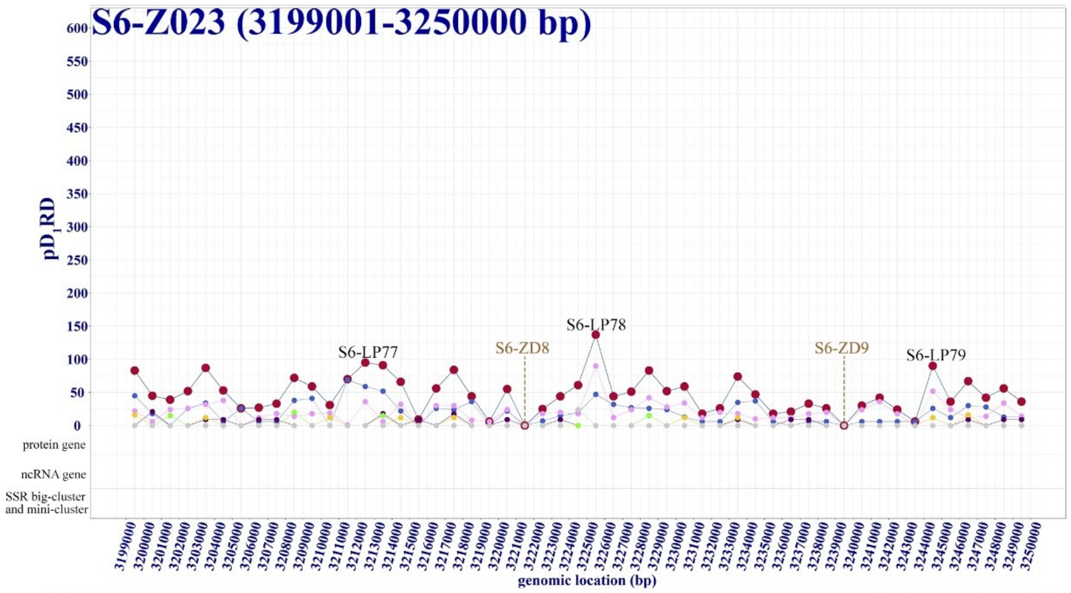

overall SSRs | mono-SSRs | di-SSRs | tri-SSRs | tetra-SSRs | penta-SSRs | hexa-SSRs

protein gene non-coding region (including intron and untranslated region) | protein gene coding exon | ncRNA gene intron | ncRNA gene exon

SSR big-clusters | SSR mini-clusters

sHP (super high peak, $pD_1RD \geq 425.00$); **HP** (high peak, $300.00 \leq pD_1RD < 425.00$); **MP** (middle peak ($150.00 \leq pD_1RD < 300.00$); **LP** (low peak, $90.00 \leq pD_1RD < 150.00$).

bELR (big SSR extremely low density region, size ≥100 Kbp, relative density (RD) <25.00); **sELR** (small SSR extremely low density region, 3 Kbp ≤ size <100 Kbp, RD<25.00; **ZD** (SSR desert region with $pD_1RD = 0$, size ≤2 Kbp)

Supplementary Figure 1.61. The SSR position related D_1-relative density (pD_1RD) map of position at 3199001-3250000 bp of human reference Y-DNA (NC_000024.10) at resolution of 1 Kbp.

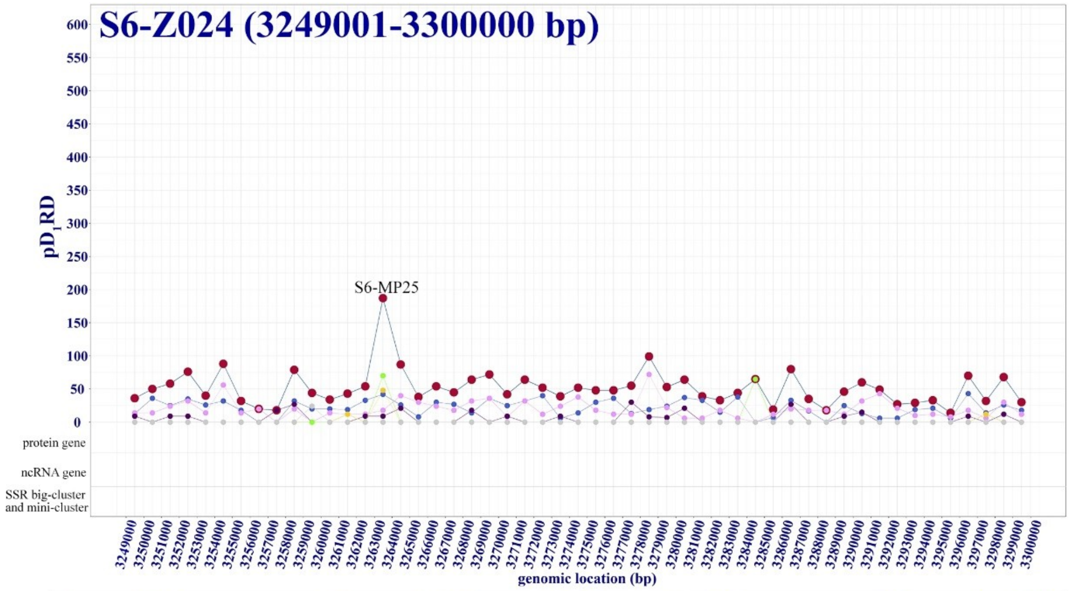

overall SSRs | mono-SSRs | di-SSRs | tri-SSRs | tetra-SSRs | penta-SSRs | hexa-SSRs

protein gene non-coding region (including intron and untranslated region) | protein gene coding exon | ncRNA gene intron | ncRNA gene exon

SSR big-clusters | SSR mini-clusters

sHP (super high peak, $pD_1RD \geq 425.00$); **HP** (high peak, $300.00 \leq pD_1RD < 425.00$); **MP** (middle peak ($150.00 \leq pD_1RD < 300.00$); **LP** (low peak, $90.00 \leq pD_1RD < 150.00$).

bELR (big SSR extremely low density region, size ≥100 Kbp, relative density (RD) <25.00); **sELR** (small SSR extremely low density region, 3 Kbp ≤ size <100 Kbp, RD<25.00; **ZD** (SSR desert region with $pD_1RD = 0$, size ≤2 Kbp)

Supplementary Figure 1.62. The SSR position related D_1-relative density (pD_1RD) map of position at 3249001-3300000 bp of human reference Y-DNA (NC_000024.10) at resolution of 1 Kbp.

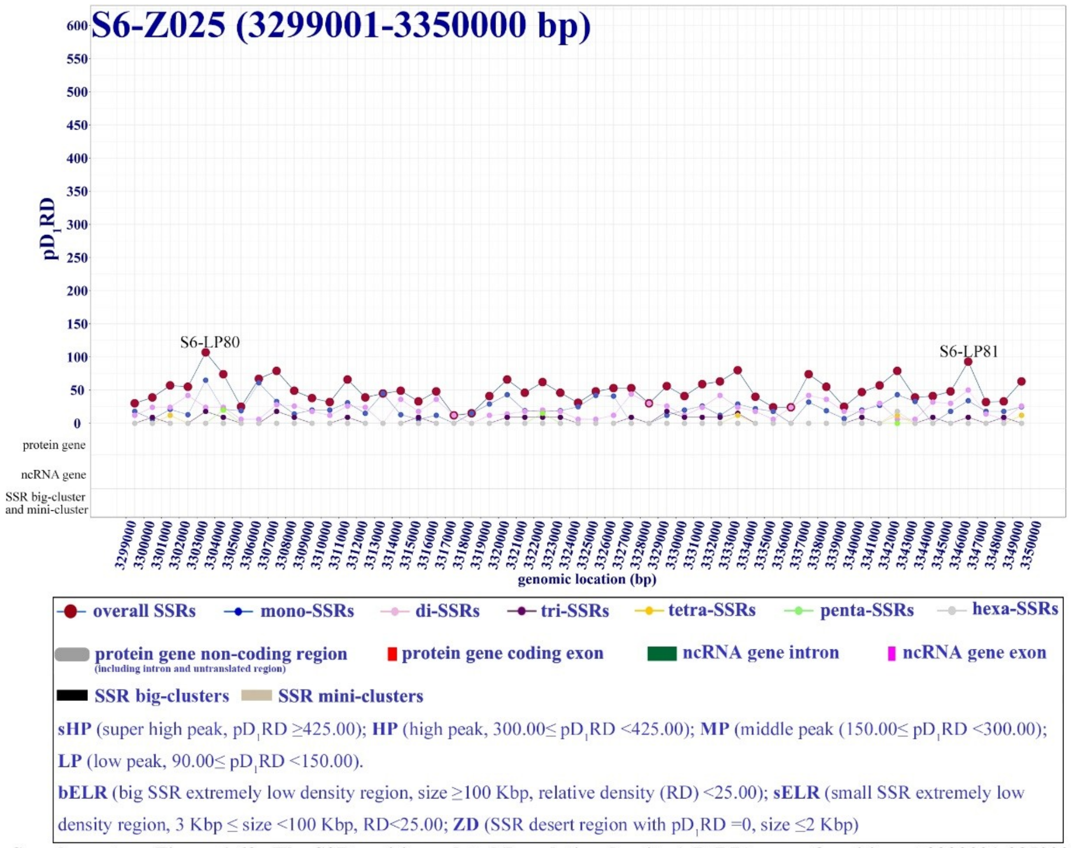

Supplementary Figure 1.63. The SSR position related D_1-relative density (pD_1RD) map of position at 3299001-3350000 bp of human reference Y-DNA (NC_000024.10) at resolution of 1 Kbp.

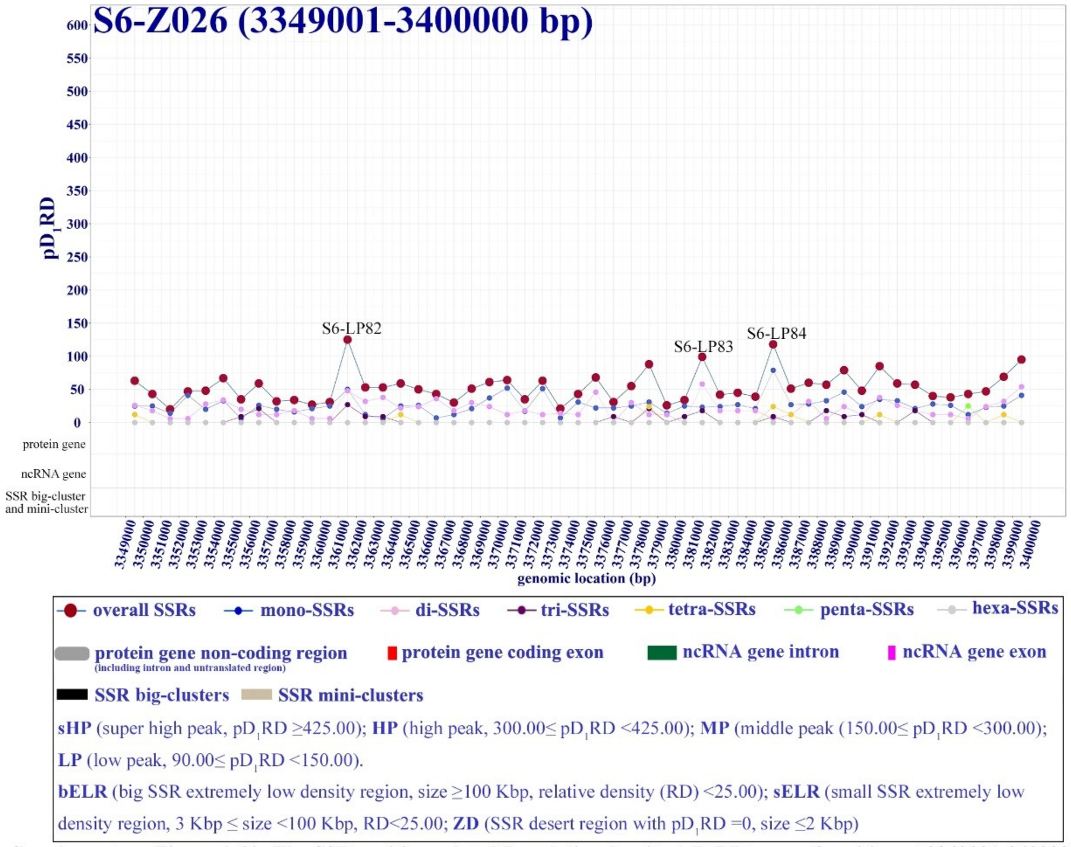

Supplementary Figure 1.64. The SSR position related D_1-relative density (pD_1RD) map of position at 3349001-3400000 bp of human reference Y-DNA (NC_000024.10) at resolution of 1 Kbp.

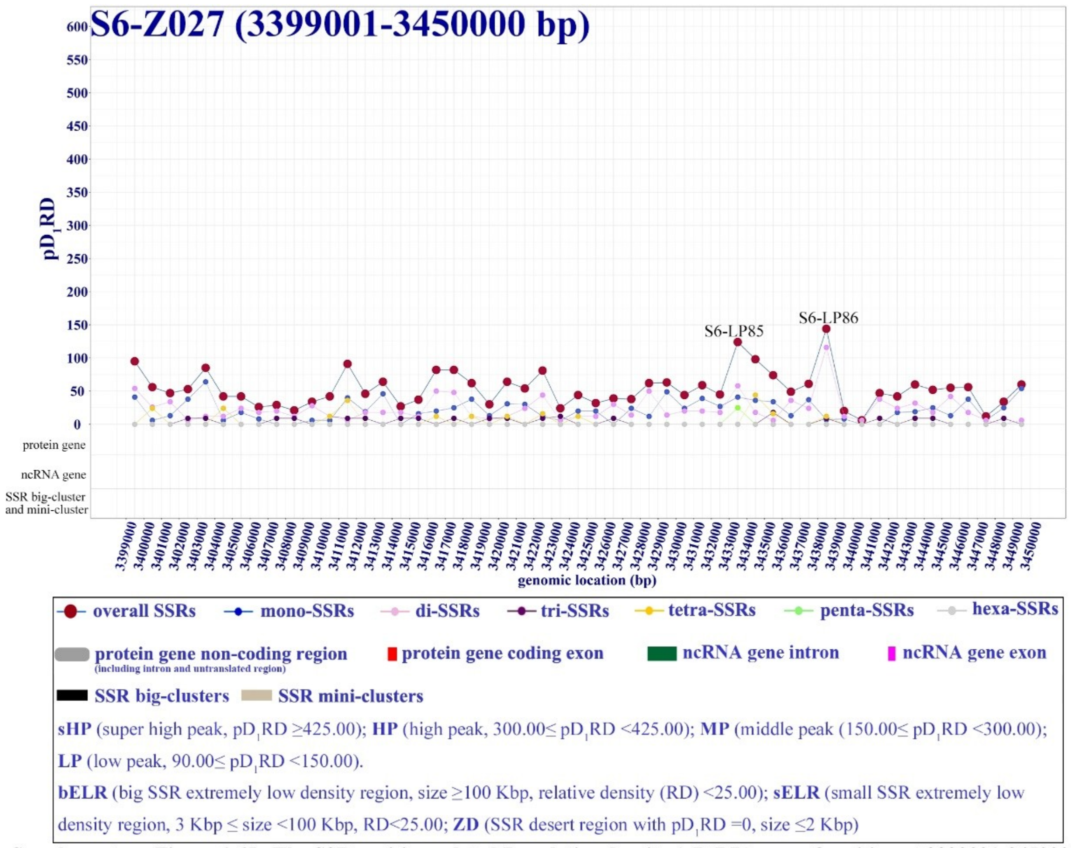

Supplementary Figure 1.65. The SSR position related D_1-relative density (pD_1RD) map of position at 3399001-3450000 bp of human reference Y-DNA (NC_000024.10) at resolution of 1 Kbp.

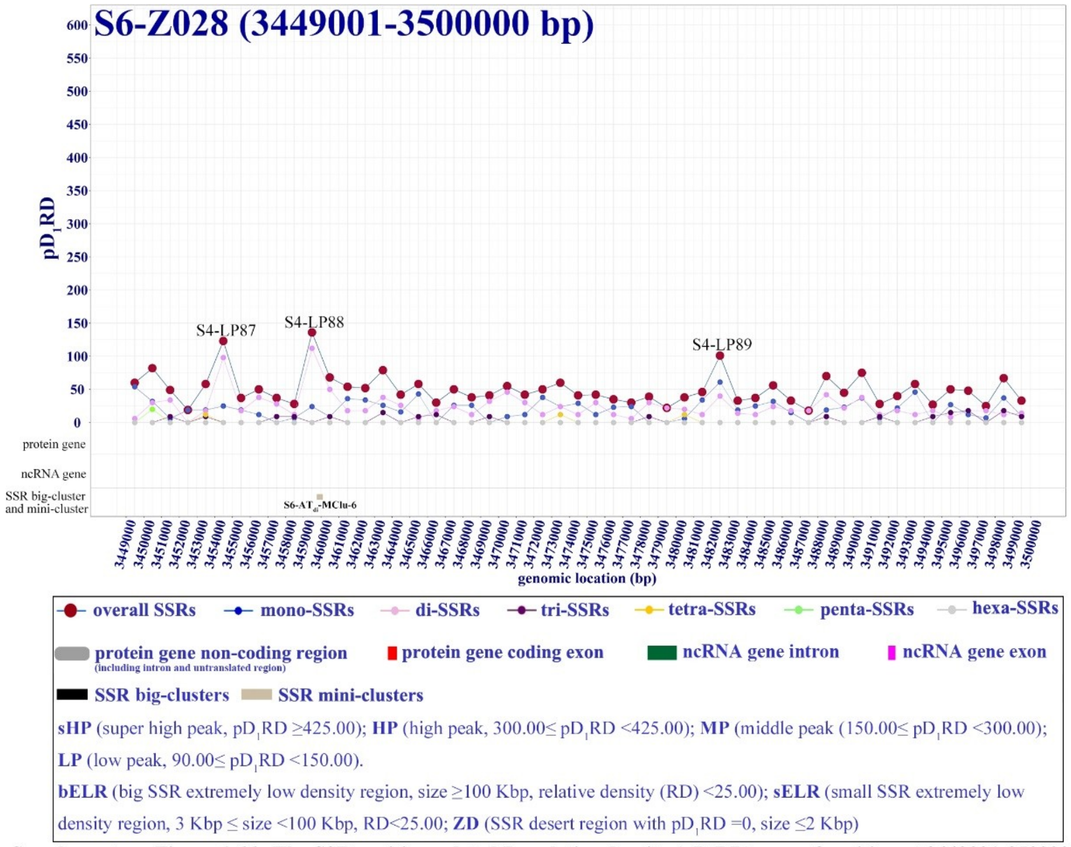

Supplementary Figure 1.66. The SSR position related D_1-relative density (pD_1RD) map of position at 3449001-3500000 bp of human reference Y-DNA (NC_000024.10) at resolution of 1 Kbp.

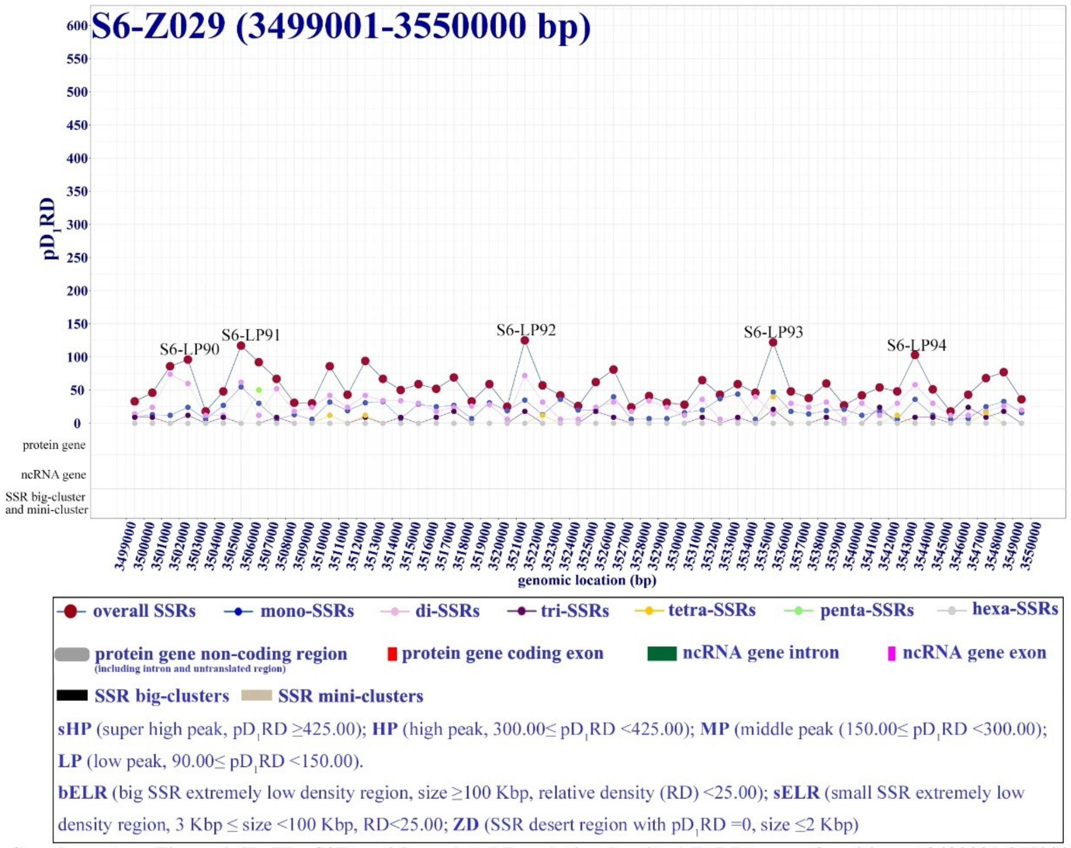

Supplementary Figure 1.67. The SSR position related D_1-relative density (pD_1RD) map of position at 3499001-3550000 bp of human reference Y-DNA (NC_000024.10) at resolution of 1 Kbp.

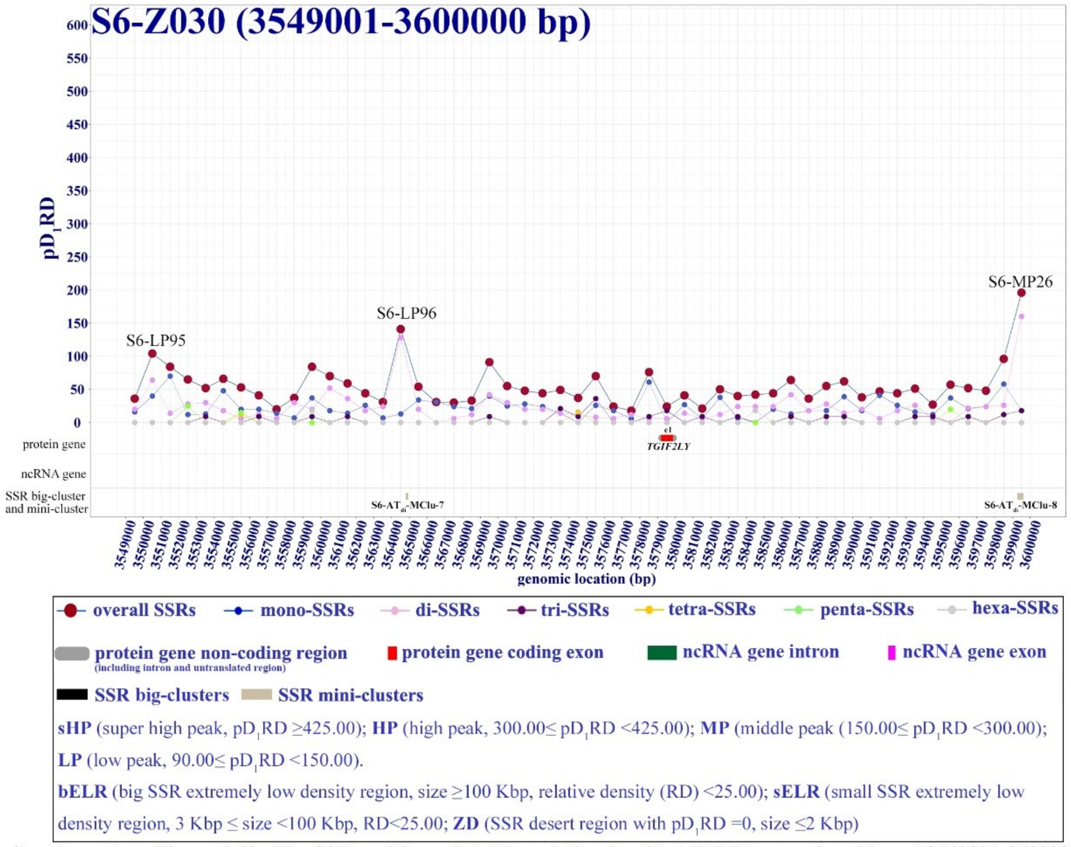

Supplementary Figure 1.68. The SSR position related D_1-relative density (pD_1RD) map of position at 3549001-3600000 bp of human reference Y-DNA (NC_000024.10) at resolution of 1 Kbp.

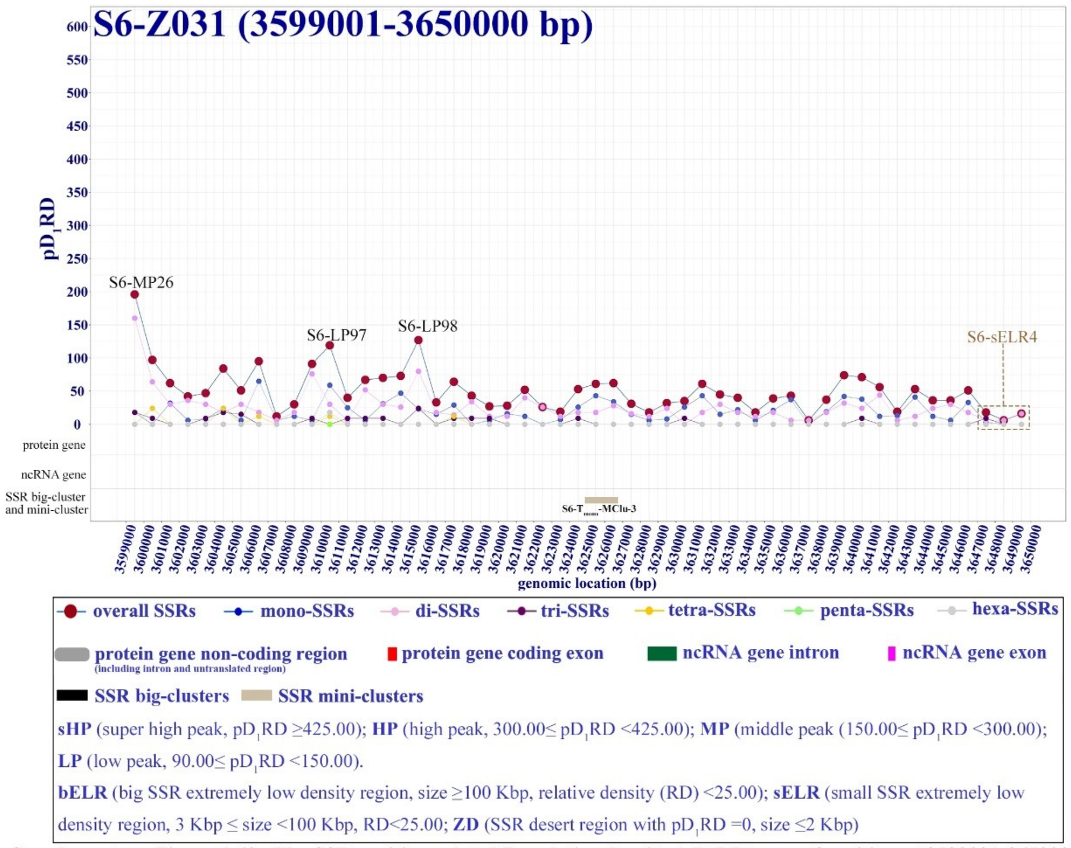

Supplementary Figure 1.69. The SSR position related D_1-relative density (pD_1RD) map of position at 3599001-3650000 bp of human reference Y-DNA (NC_000024.10) at resolution of 1 Kbp.

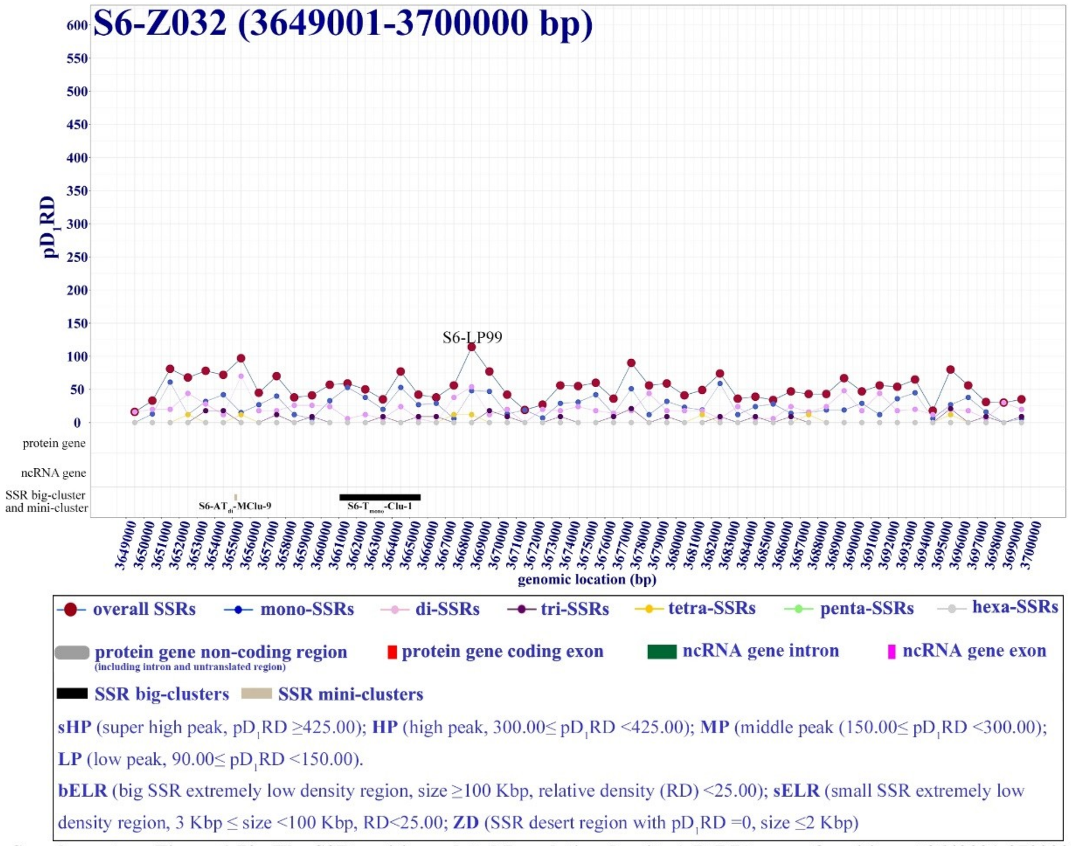

Supplementary Figure 1.70. The SSR position related D_1-relative density (pD_1RD) map of position at 3649001-3700000 bp of human reference Y-DNA (NC_000024.10) at resolution of 1 Kbp.

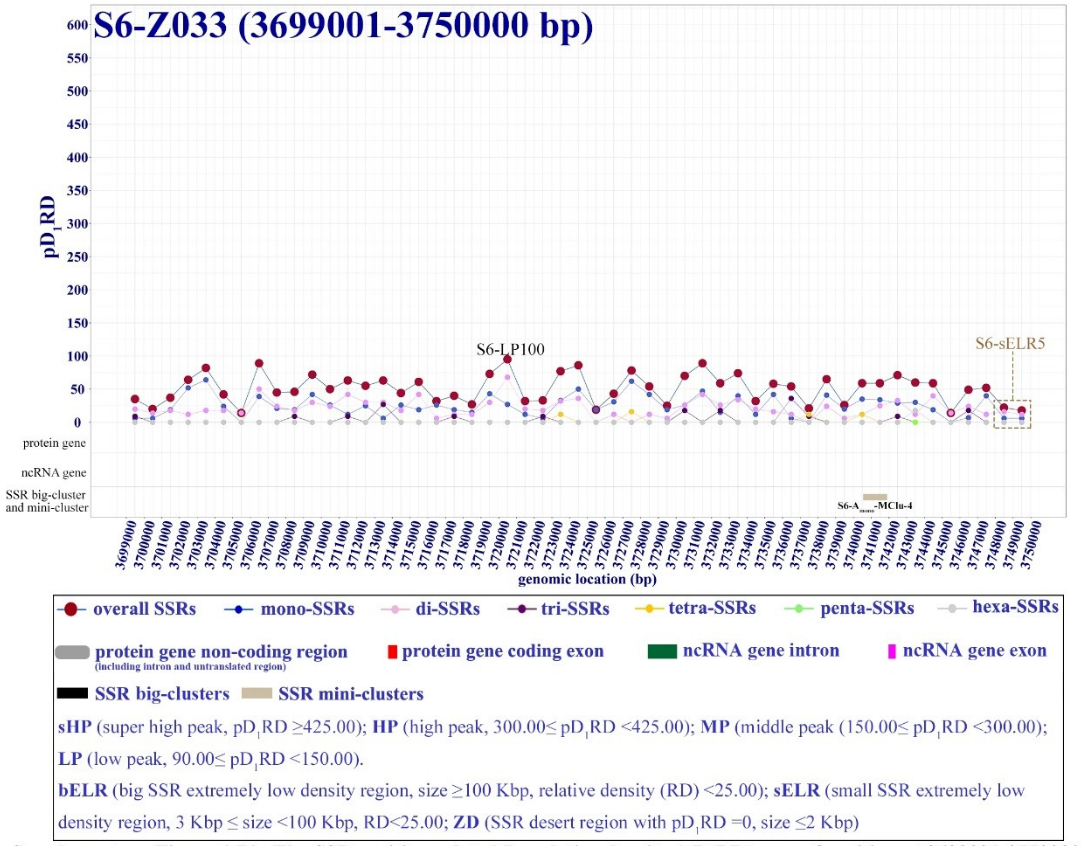

Supplementary Figure 1.71. The SSR position related D_1-relative density (pD_1RD) map of position at 3699001-3750000 bp of human reference Y-DNA (NC_000024.10) at resolution of 1 Kbp.

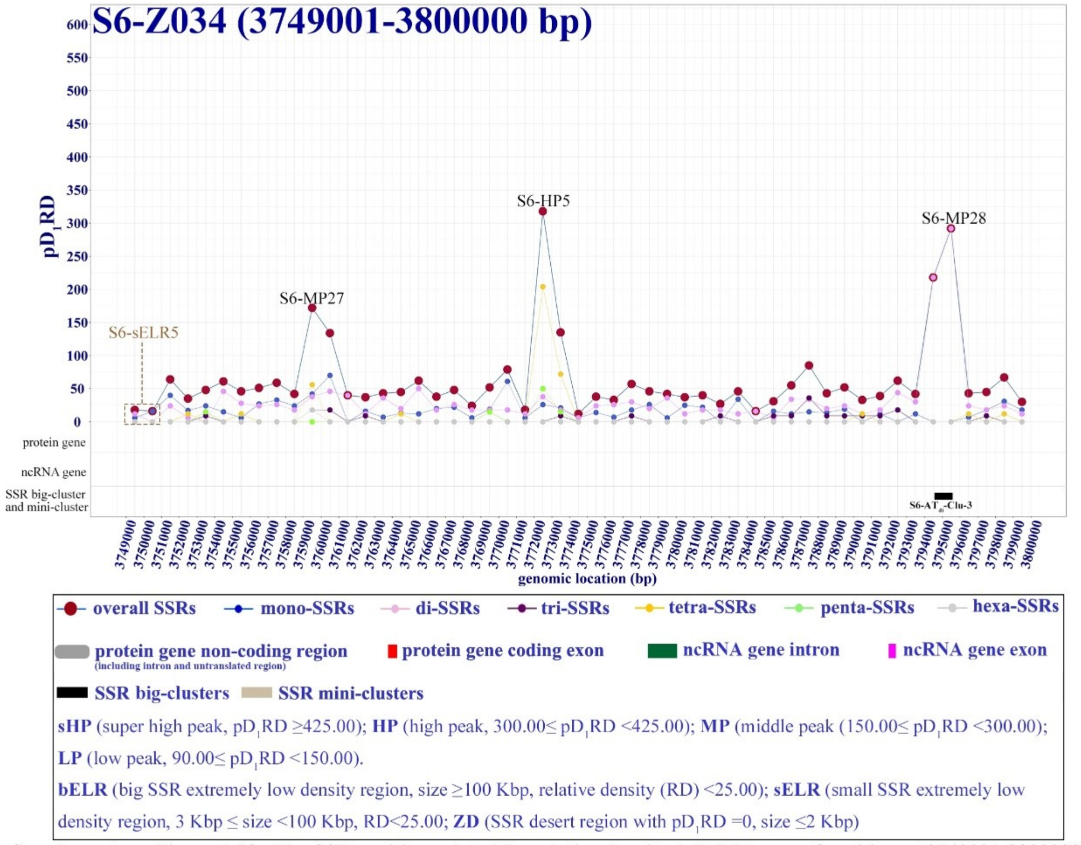

Supplementary Figure 1.72. The SSR position related D_1-relative density (pD_1RD) map of position at 3749001-3800000 bp of human reference Y-DNA (NC_000024.10) at resolution of 1 Kbp.

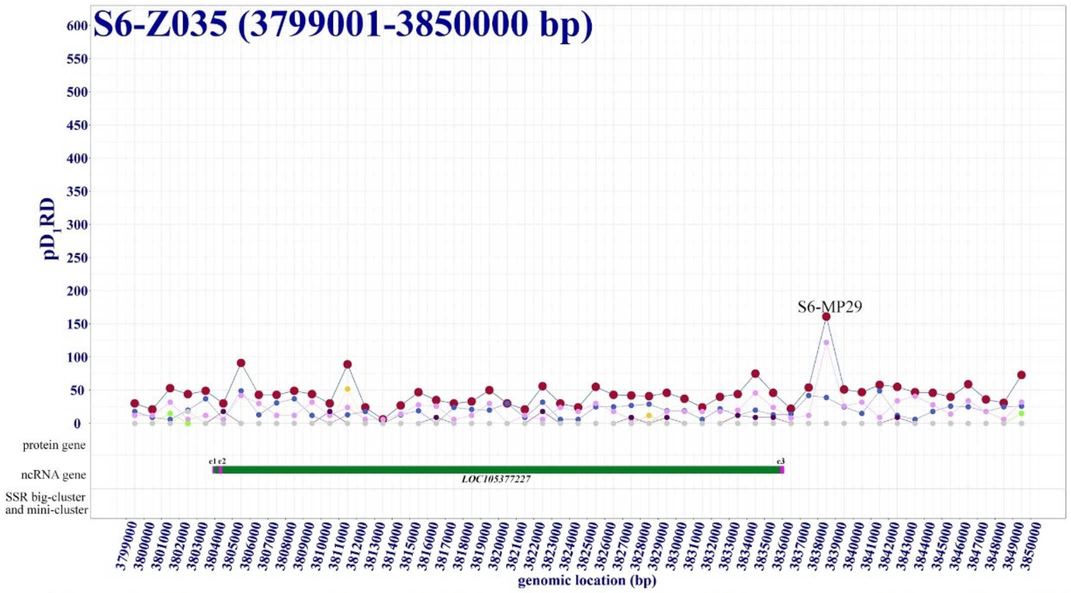

overall SSRs — mono-SSRs — di-SSRs — tri-SSRs — tetra-SSRs — penta-SSRs — hexa-SSRs

protein gene non-coding region (including intron and untranslated region) | protein gene coding exon | ncRNA gene intron | ncRNA gene exon

SSR big-clusters | SSR mini-clusters

sHP (super high peak, $pD_1RD \geq 425.00$); **HP** (high peak, $300.00 \leq pD_1RD < 425.00$); **MP** (middle peak ($150.00 \leq pD_1RD < 300.00$); **LP** (low peak, $90.00 \leq pD_1RD < 150.00$).

bELR (big SSR extremely low density region, size ≥100 Kbp, relative density (RD) <25.00); **sELR** (small SSR extremely low density region, 3 Kbp ≤ size <100 Kbp, RD<25.00; **ZD** (SSR desert region with $pD_1RD = 0$, size ≤2 Kbp)

Supplementary Figure 1.73. The SSR position related D_1-relative density (pD_1RD) map of position at 3799001-3850000 bp of human reference Y-DNA (NC_000024.10) at resolution of 1 Kbp.

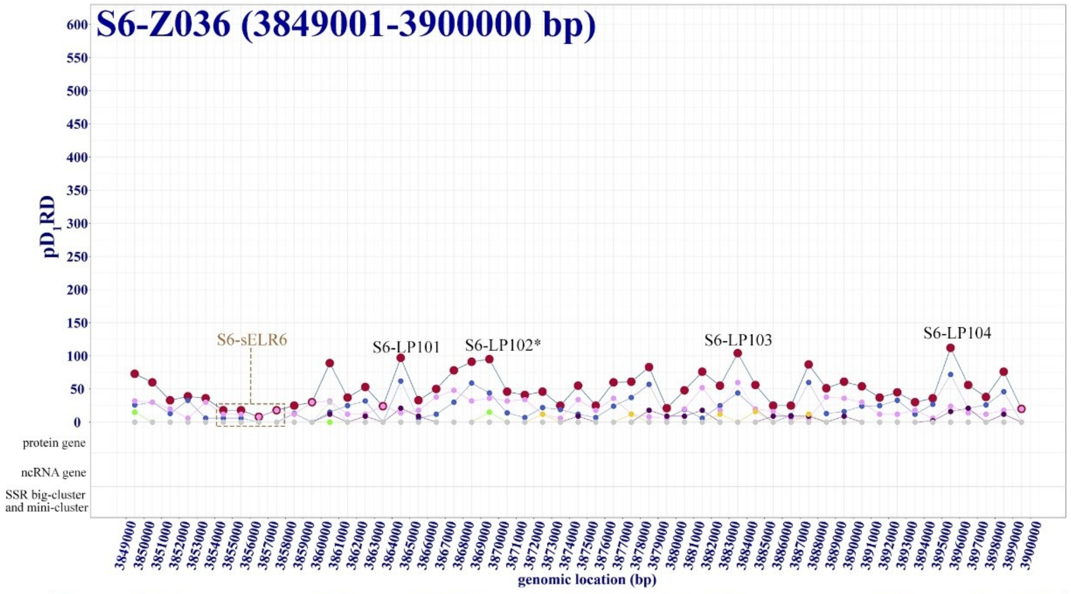

overall SSRs — mono-SSRs — di-SSRs — tri-SSRs — tetra-SSRs — penta-SSRs — hexa-SSRs

protein gene non-coding region (including intron and untranslated region) | protein gene coding exon | ncRNA gene intron | ncRNA gene exon

SSR big-clusters | SSR mini-clusters

sHP (super high peak, $pD_1RD \geq 425.00$); **HP** (high peak, $300.00 \leq pD_1RD < 425.00$); **MP** (middle peak ($150.00 \leq pD_1RD < 300.00$); **LP** (low peak, $90.00 \leq pD_1RD < 150.00$).

bELR (big SSR extremely low density region, size ≥100 Kbp, relative density (RD) <25.00); **sELR** (small SSR extremely low density region, 3 Kbp ≤ size <100 Kbp, RD<25.00; **ZD** (SSR desert region with $pD_1RD = 0$, size ≤2 Kbp)

Supplementary Figure 1.74. The SSR position related D_1-relative density (pD_1RD) map of position at 3849001-3900000 bp of human reference Y-DNA (NC_000024.10) at resolution of 1 Kbp.

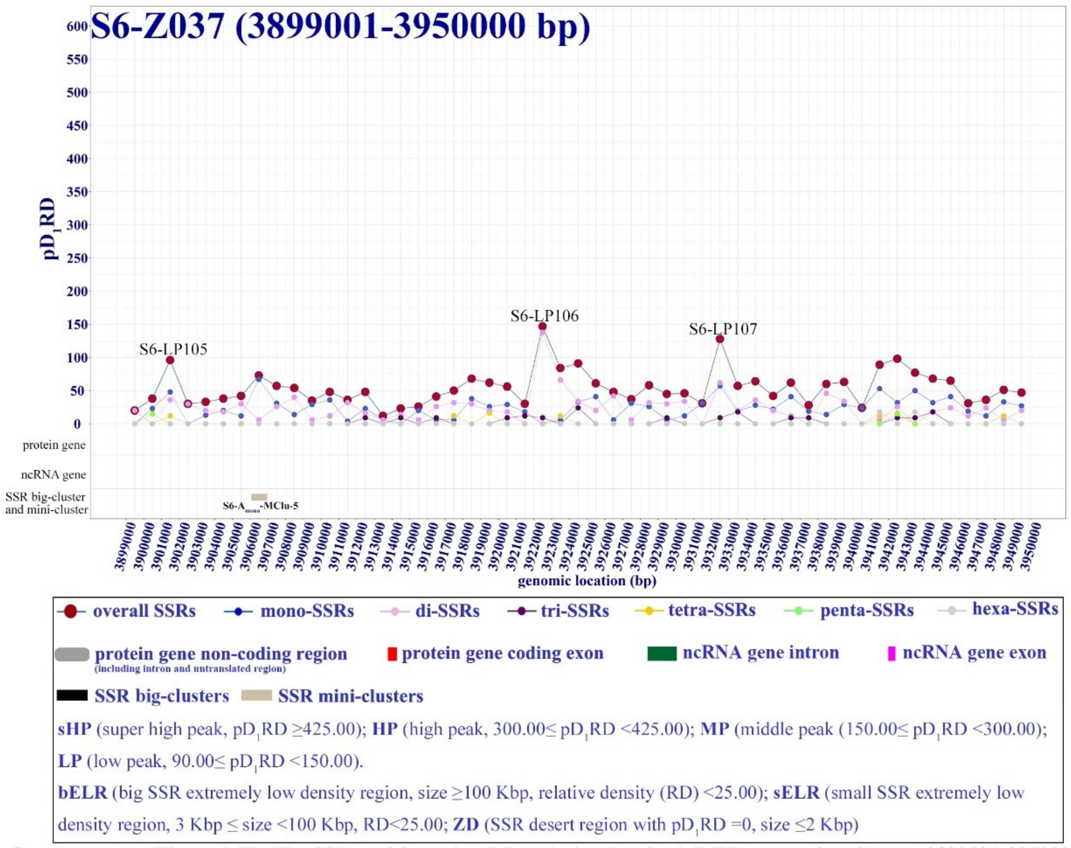

Supplementary Figure 1.75. The SSR position related D_1-relative density (pD_1RD) map of position at 3899001-3950000 bp of human reference Y-DNA (NC_000024.10) at resolution of 1 Kbp.

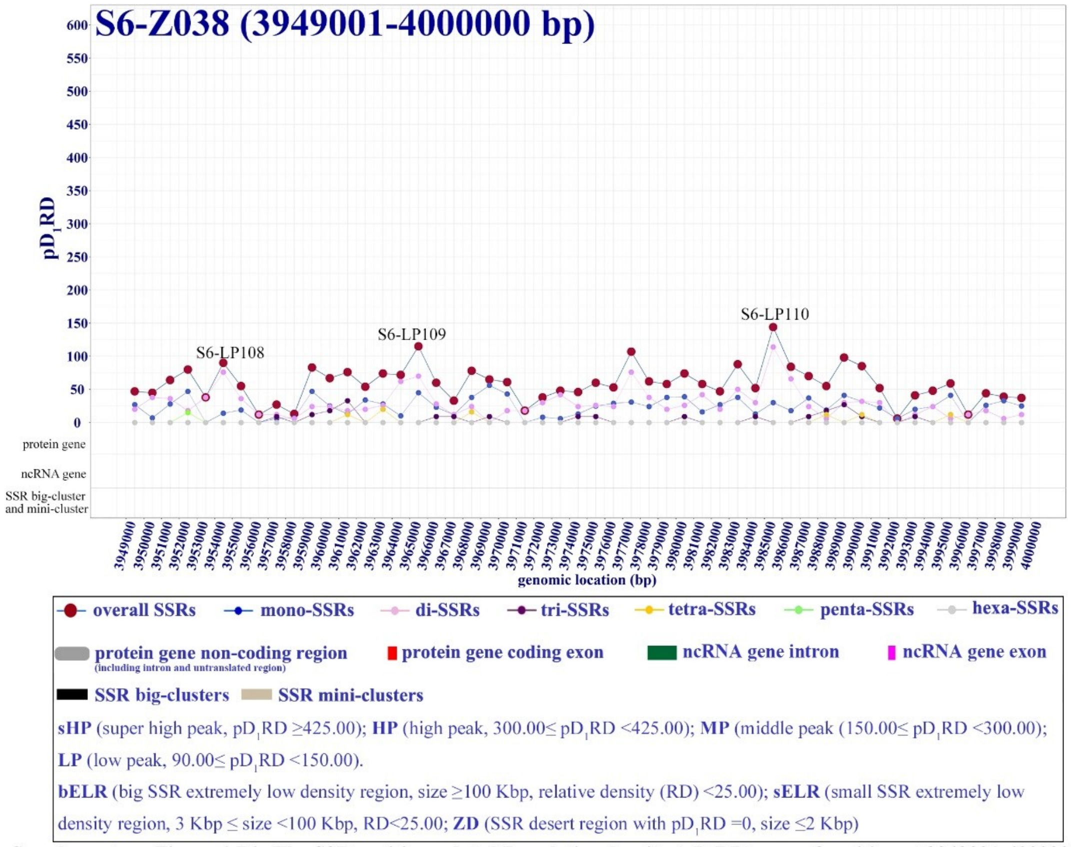

Supplementary Figure 1.76. The SSR position related D_1-relative density (pD_1RD) map of position at 3949001-4000000 bp of human reference Y-DNA (NC_000024.10) at resolution of 1 Kbp.

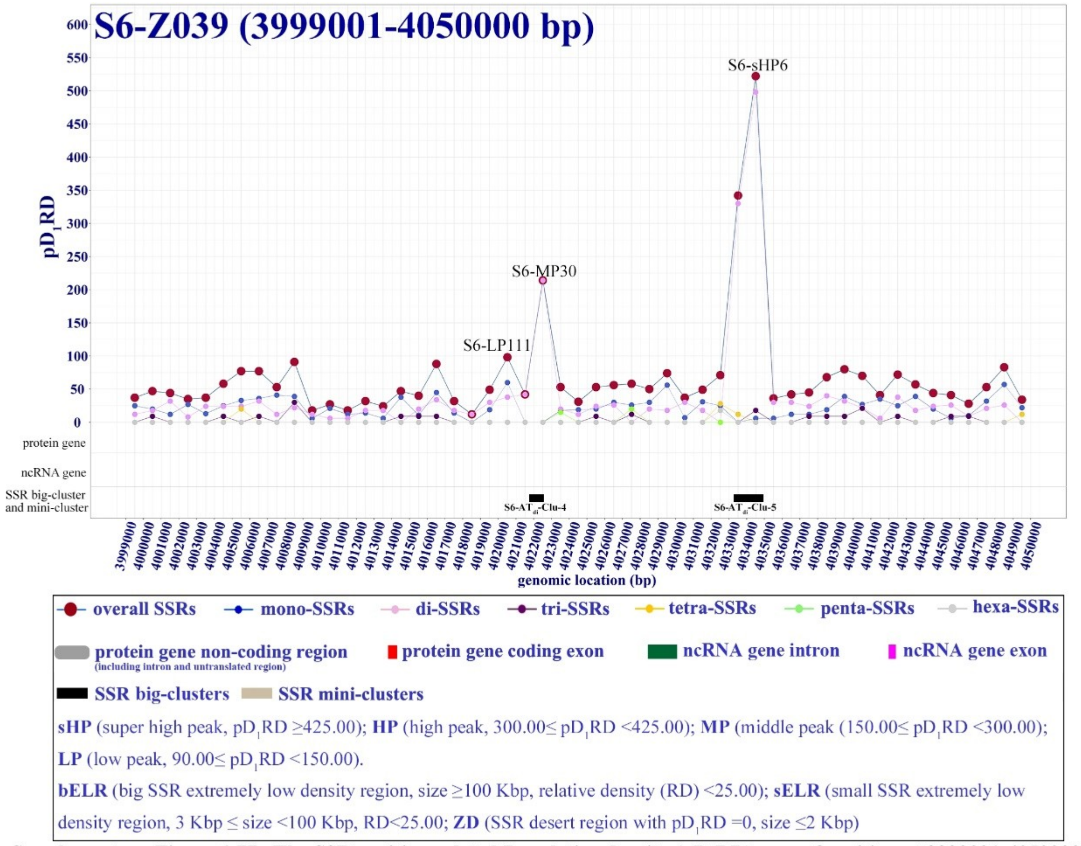

Supplementary Figure 1.77. The SSR position related D_1-relative density (pD_1RD) map of position at 3999001-4050000 bp of human reference Y-DNA (NC_000024.10) at resolution of 1 Kbp.

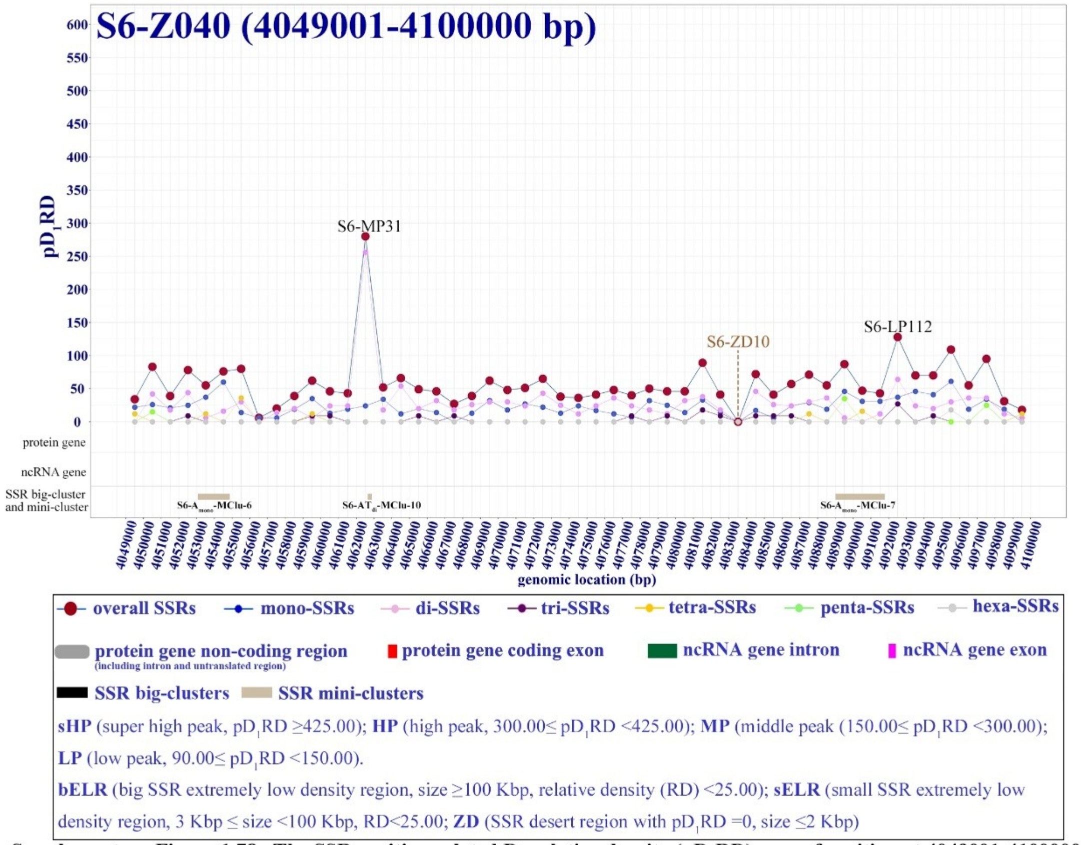

Supplementary Figure 1.78. The SSR position related D_1-relative density (pD_1RD) map of position at 4049001-4100000 bp of human reference Y-DNA (NC_000024.10) at resolution of 1 Kbp.

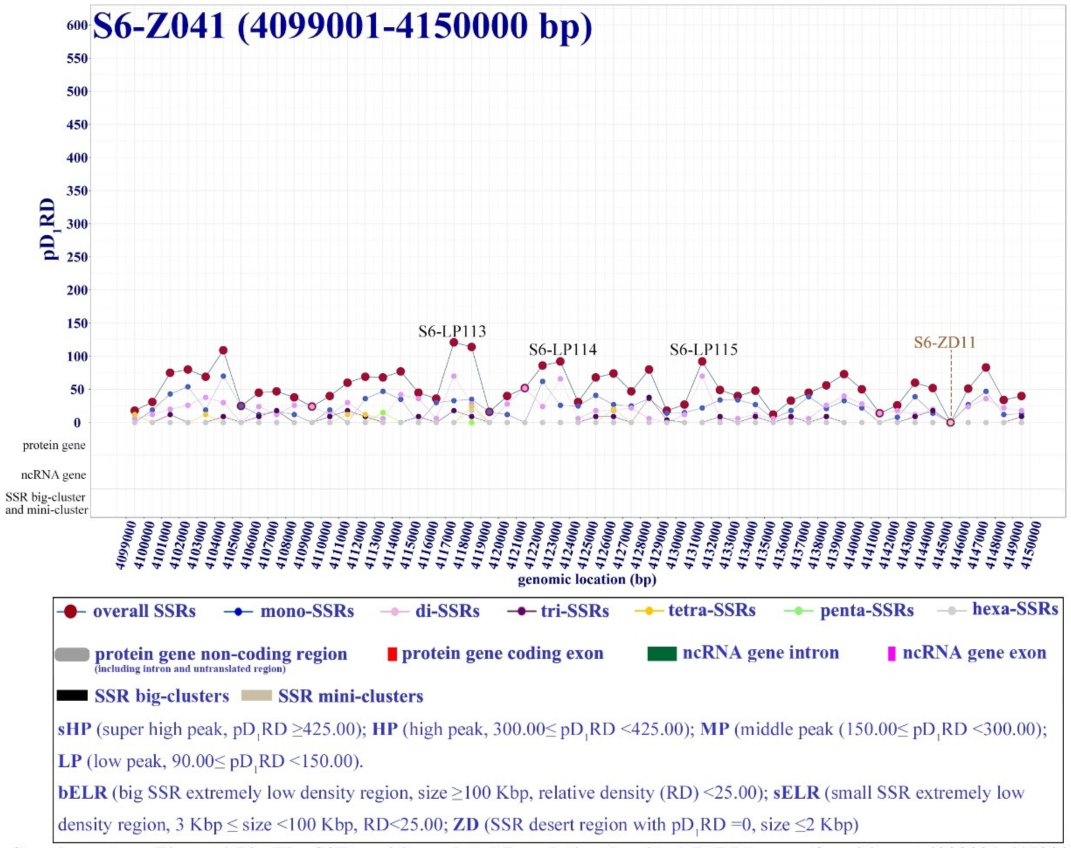

Supplementary Figure 1.79. The SSR position related D_1-relative density (pD_1RD) map of position at 4099001-4150000 bp of human reference Y-DNA (NC_000024.10) at resolution of 1 Kbp.

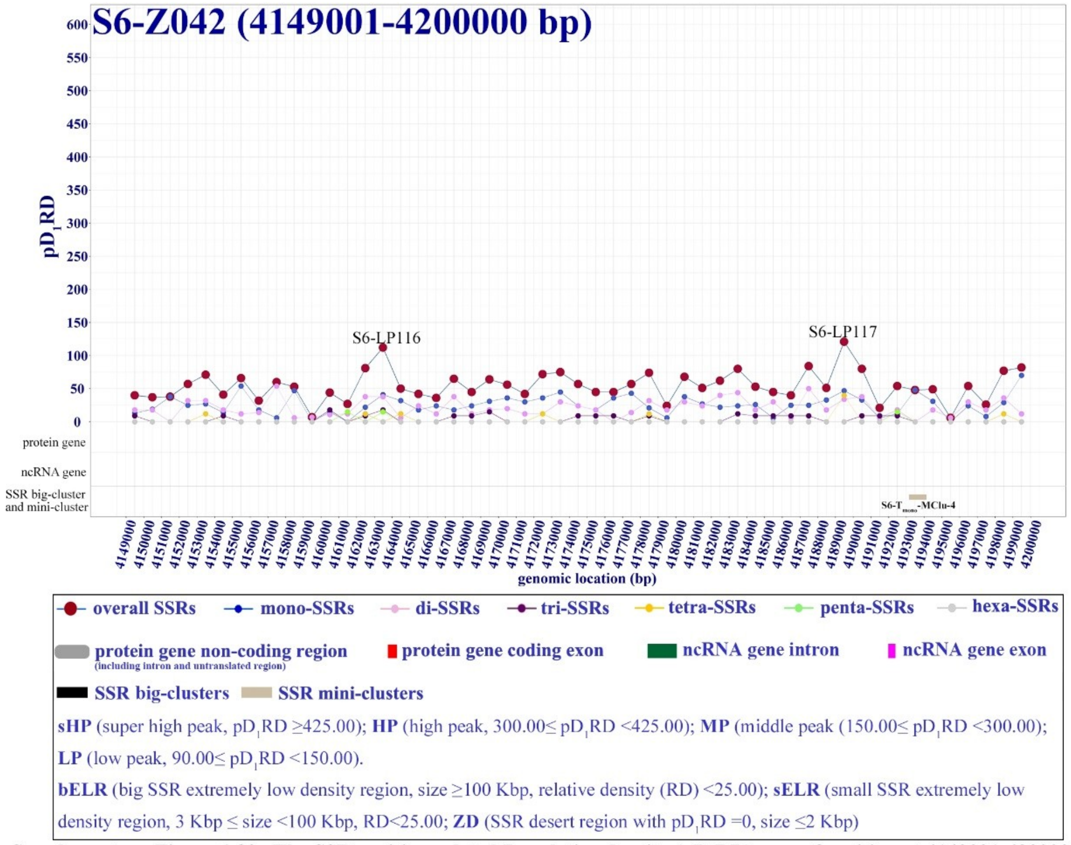

Supplementary Figure 1.80. The SSR position related D_1-relative density (pD_1RD) map of position at 4149001-4200000 bp of human reference Y-DNA (NC_000024.10) at resolution of 1 Kbp.

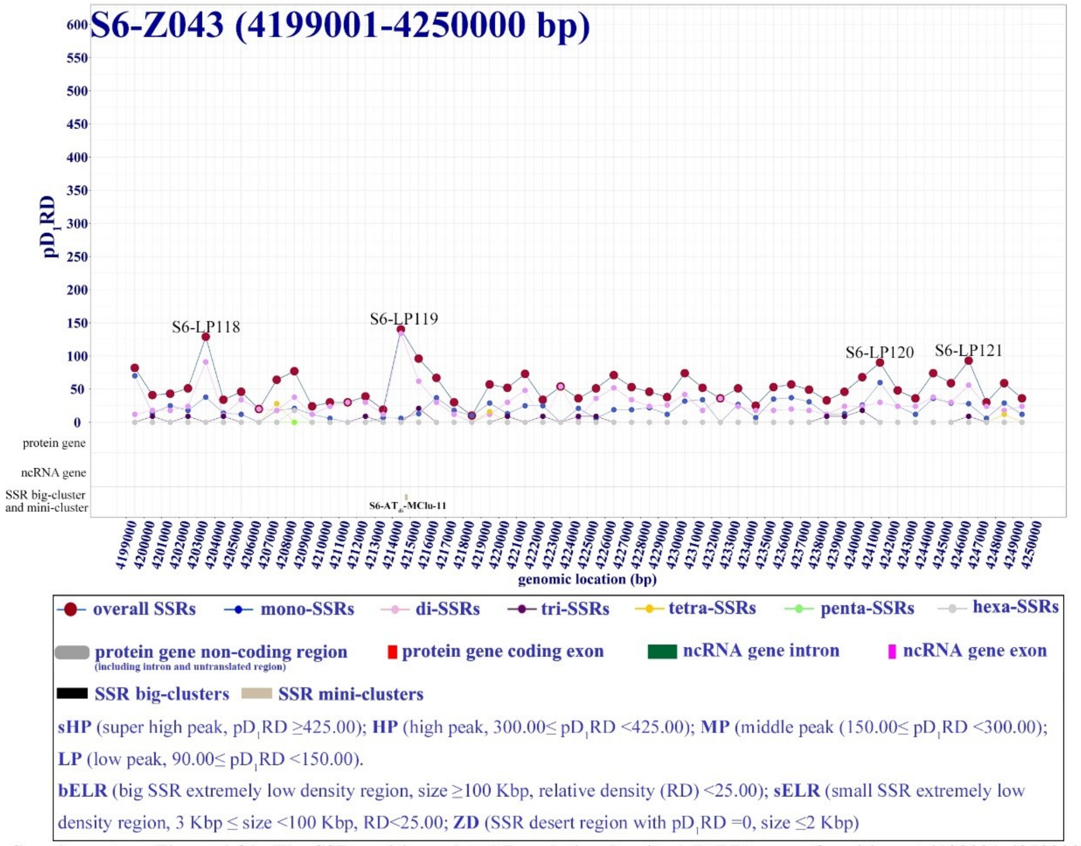

Supplementary Figure 1.81. The SSR position related D_1-relative density (pD_1RD) map of position at 4199001-4250000 bp of human reference Y-DNA (NC_000024.10) at resolution of 1 Kbp.

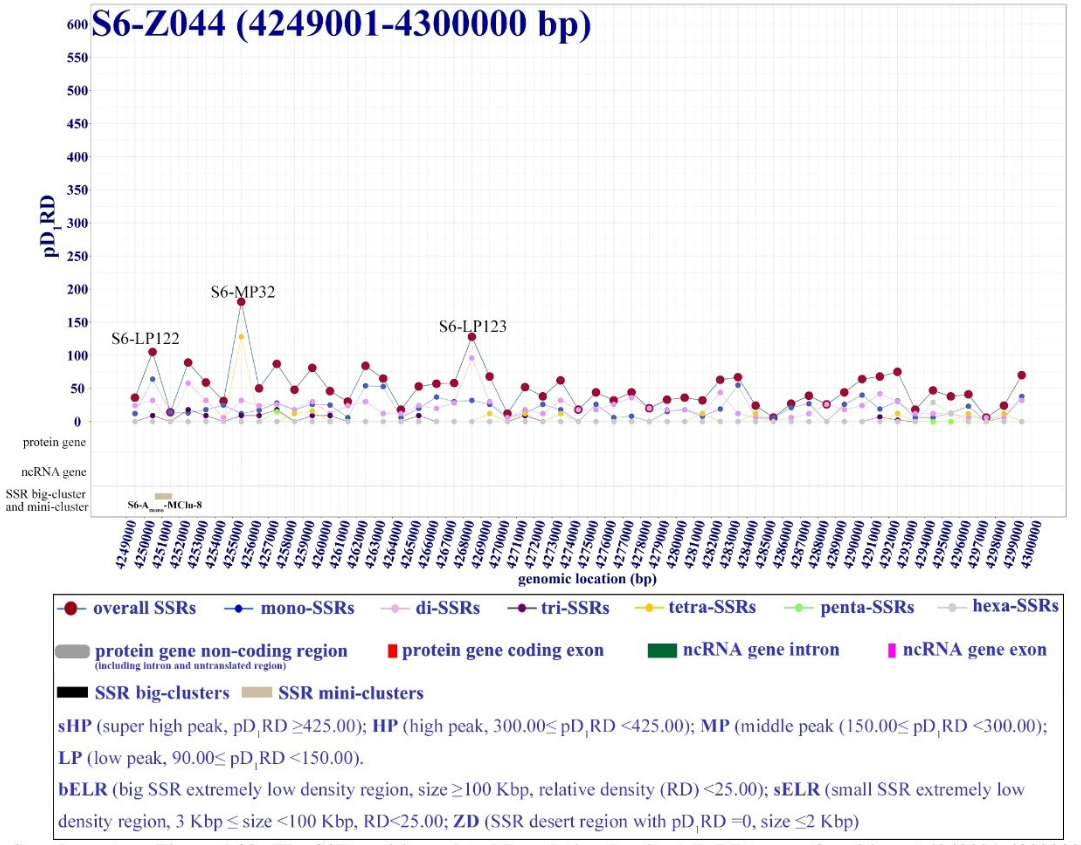

Supplementary Figure 1.82. The SSR position related D_1-relative density (pD_1RD) map of position at 4249001-4300000 bp of human reference Y-DNA (NC_000024.10) at resolution of 1 Kbp.

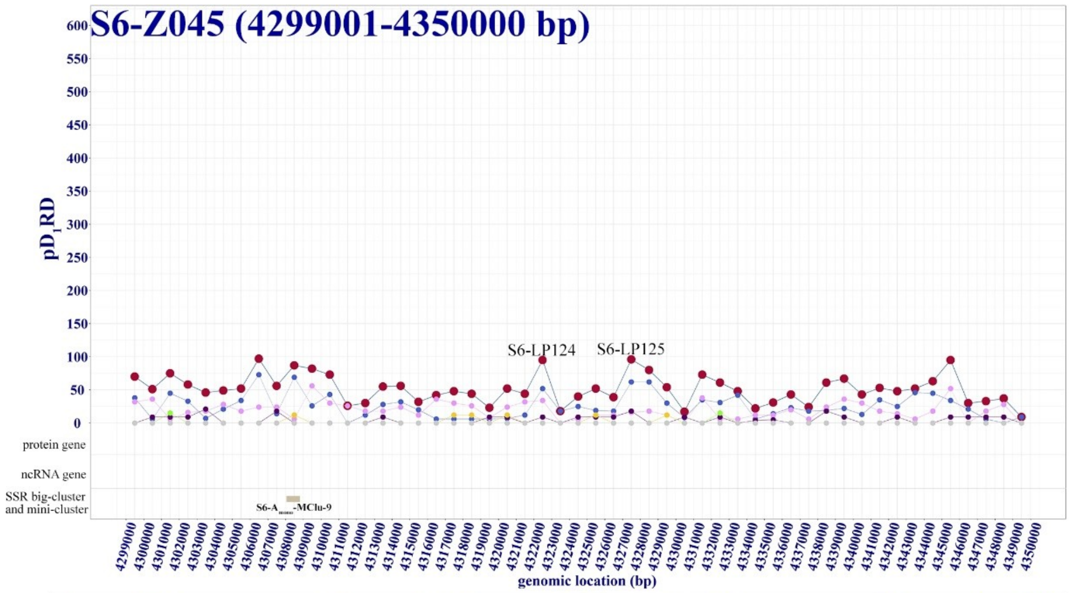

overall SSRs | mono-SSRs | di-SSRs | tri-SSRs | tetra-SSRs | penta-SSRs | hexa-SSRs

protein gene non-coding region (including intron and untranslated region) | protein gene coding exon | ncRNA gene intron | ncRNA gene exon

SSR big-clusters | SSR mini-clusters

sHP (super high peak, $pD_1RD \geq 425.00$); **HP** (high peak, $300.00 \leq pD_1RD < 425.00$); **MP** (middle peak ($150.00 \leq pD_1RD < 300.00$); **LP** (low peak, $90.00 \leq pD_1RD < 150.00$).

bELR (big SSR extremely low density region, size ≥100 Kbp, relative density (RD) <25.00); **sELR** (small SSR extremely low density region, 3 Kbp ≤ size <100 Kbp, RD<25.00; **ZD** (SSR desert region with $pD_1RD = 0$, size ≤2 Kbp)

Supplementary Figure 1.83. The SSR position related D_1-relative density (pD_1RD) map of position at 4299001-4350000 bp of human reference Y-DNA (NC_000024.10) at resolution of 1 Kbp.

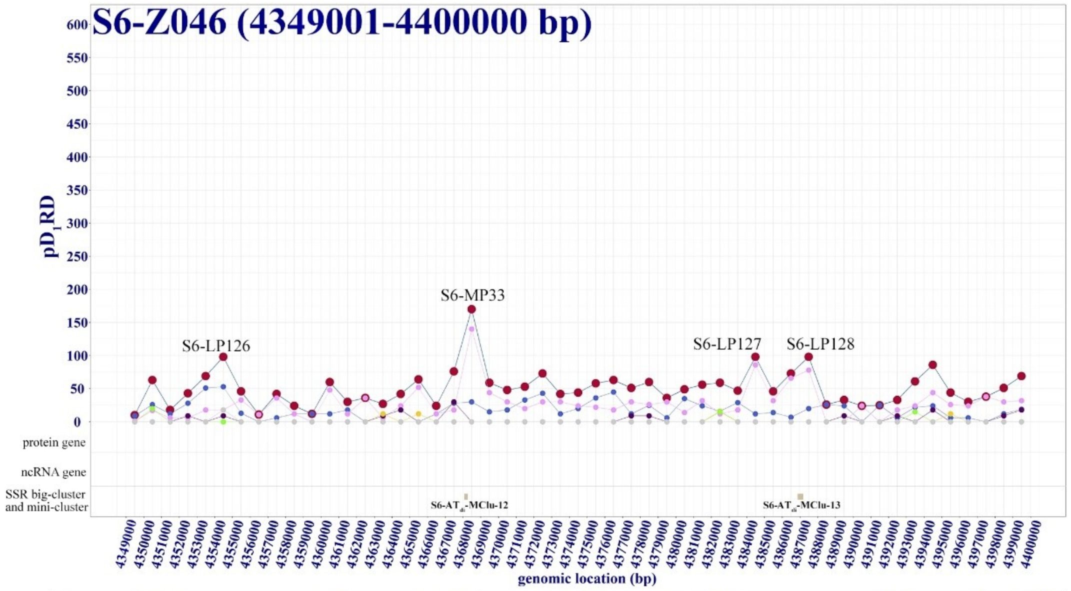

overall SSRs | mono-SSRs | di-SSRs | tri-SSRs | tetra-SSRs | penta-SSRs | hexa-SSRs

protein gene non-coding region (including intron and untranslated region) | protein gene coding exon | ncRNA gene intron | ncRNA gene exon

SSR big-clusters | SSR mini-clusters

sHP (super high peak, $pD_1RD \geq 425.00$); **HP** (high peak, $300.00 \leq pD_1RD < 425.00$); **MP** (middle peak ($150.00 \leq pD_1RD < 300.00$); **LP** (low peak, $90.00 \leq pD_1RD < 150.00$).

bELR (big SSR extremely low density region, size ≥100 Kbp, relative density (RD) <25.00); **sELR** (small SSR extremely low density region, 3 Kbp ≤ size <100 Kbp, RD<25.00; **ZD** (SSR desert region with $pD_1RD = 0$, size ≤2 Kbp)

Supplementary Figure 1.84. The SSR position related D_1-relative density (pD_1RD) map of position at 4349001-4400000 bp of human reference Y-DNA (NC_000024.10) at resolution of 1 Kbp.

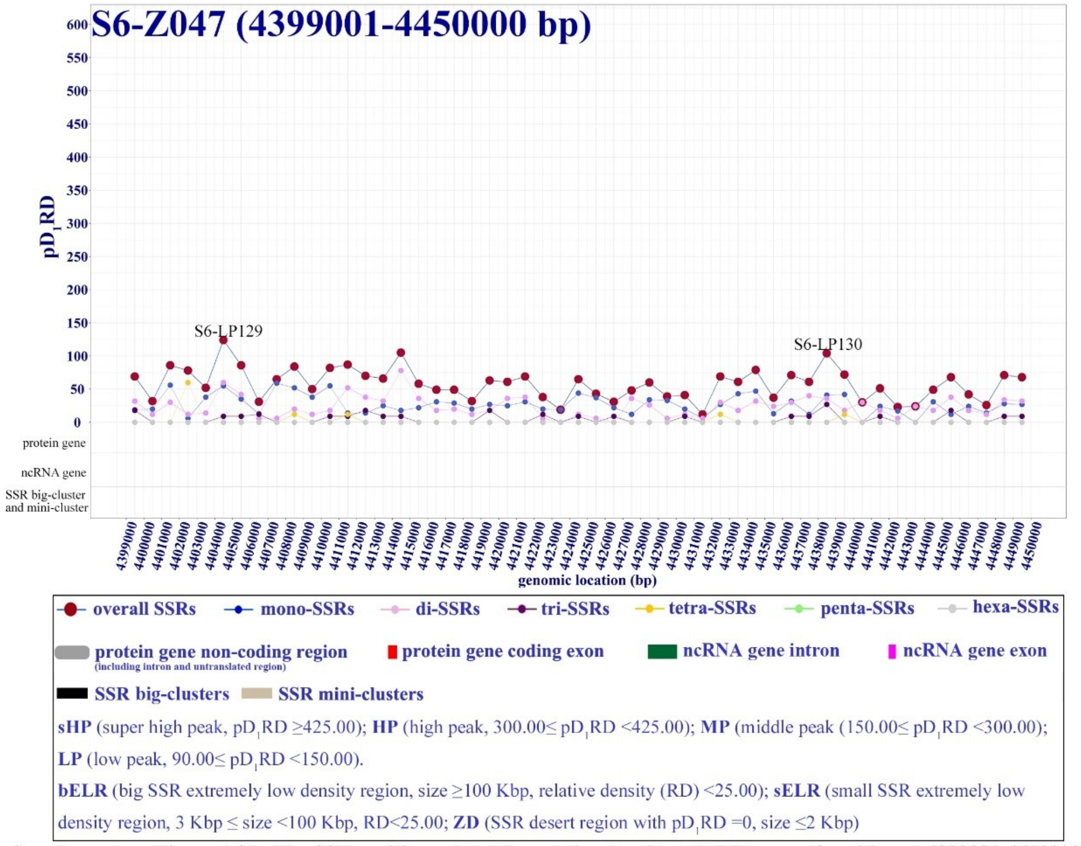

Supplementary Figure 1.85. The SSR position related D_1-relative density (pD_1RD) map of position at 4399001-4450000 bp of human reference Y-DNA (NC_000024.10) at resolution of 1 Kbp.

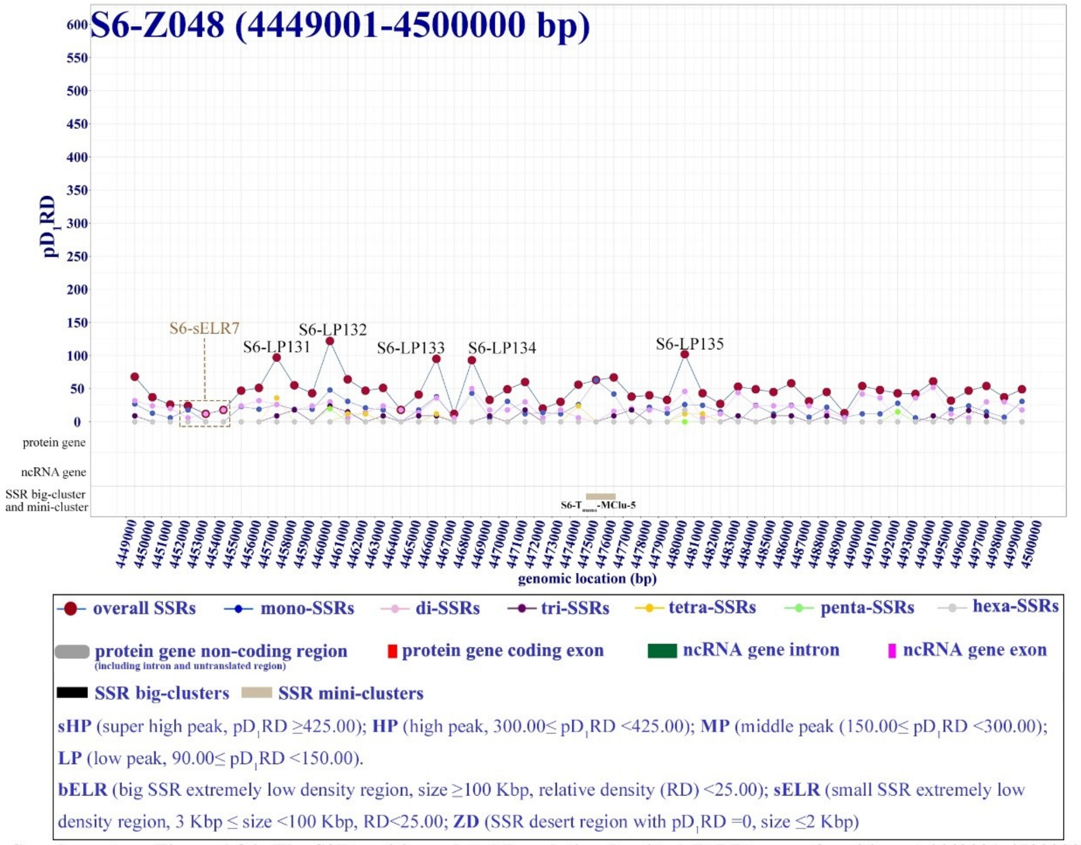

Supplementary Figure 1.86. The SSR position related D_1-relative density (pD_1RD) map of position at 4449001-4500000 bp of human reference Y-DNA (NC_000024.10) at resolution of 1 Kbp.

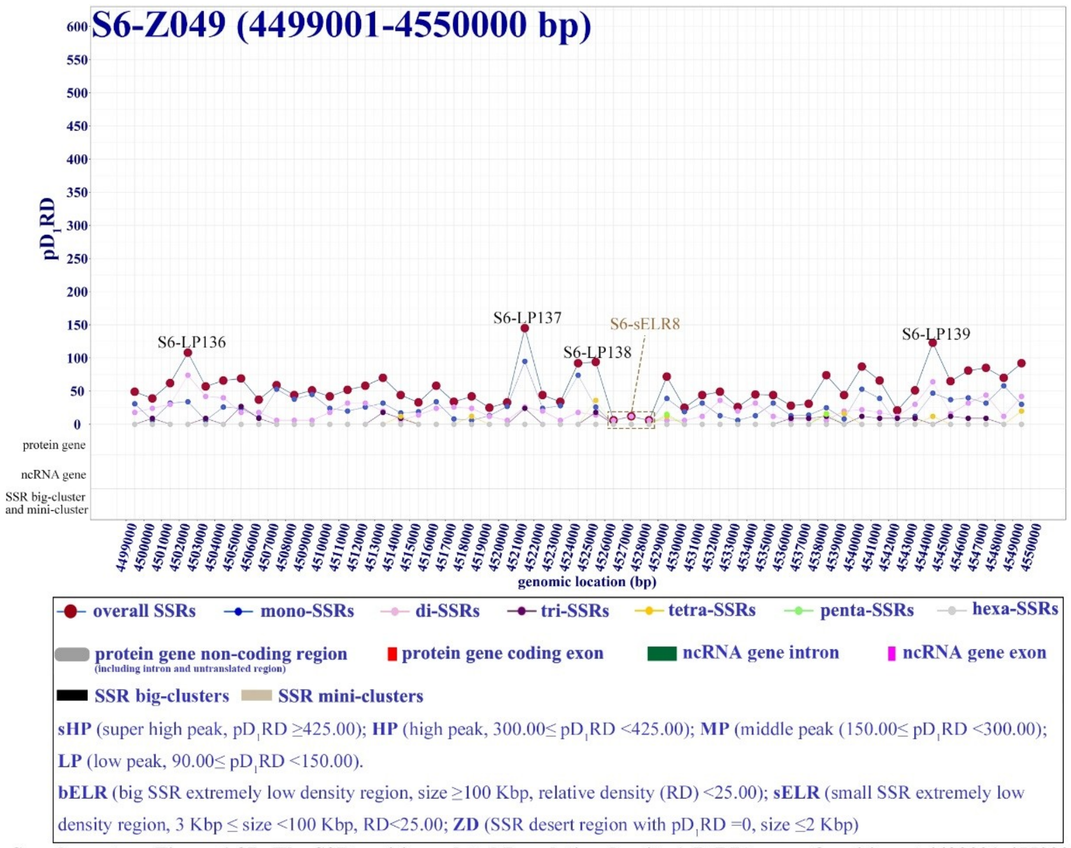

Supplementary Figure 1.87. The SSR position related D_1-relative density (pD_1RD) map of position at 4499001-4550000 bp of human reference Y-DNA (NC_000024.10) at resolution of 1 Kbp.

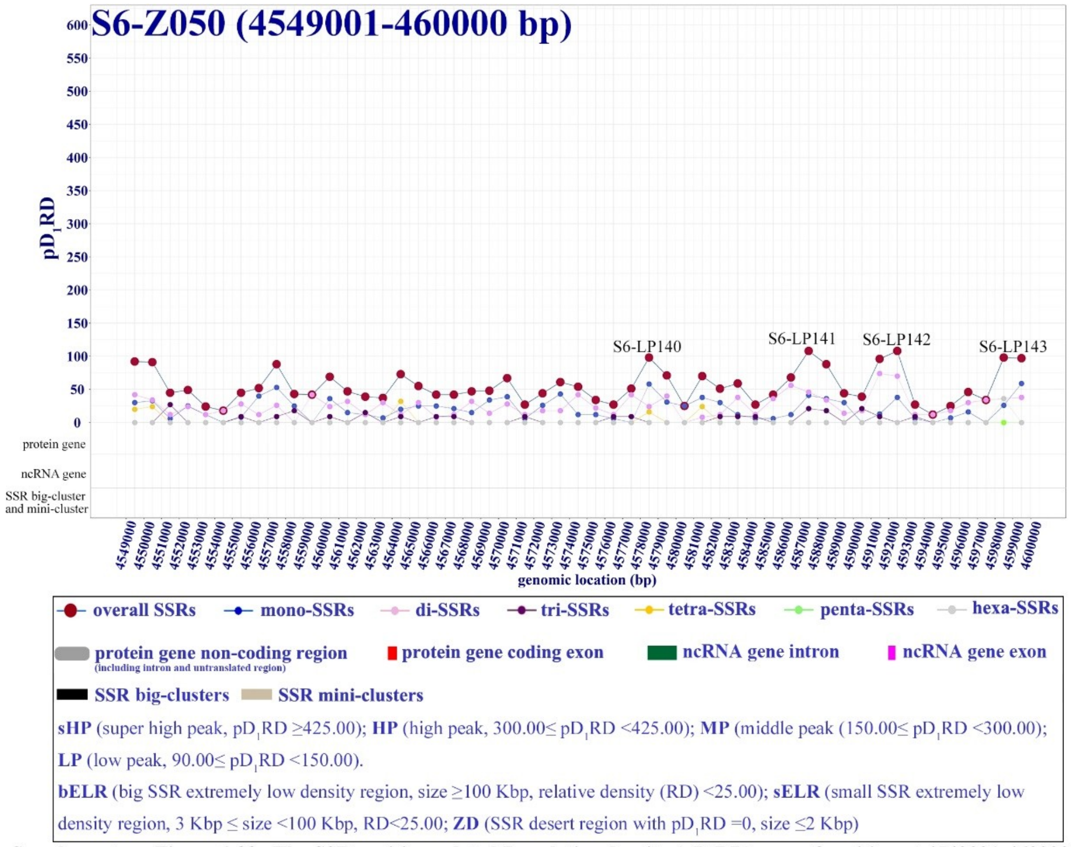

Supplementary Figure 1.88. The SSR position related D_1-relative density (pD_1RD) map of position at 4549001-4600000 bp of human reference Y-DNA (NC_000024.10) at resolution of 1 Kbp.

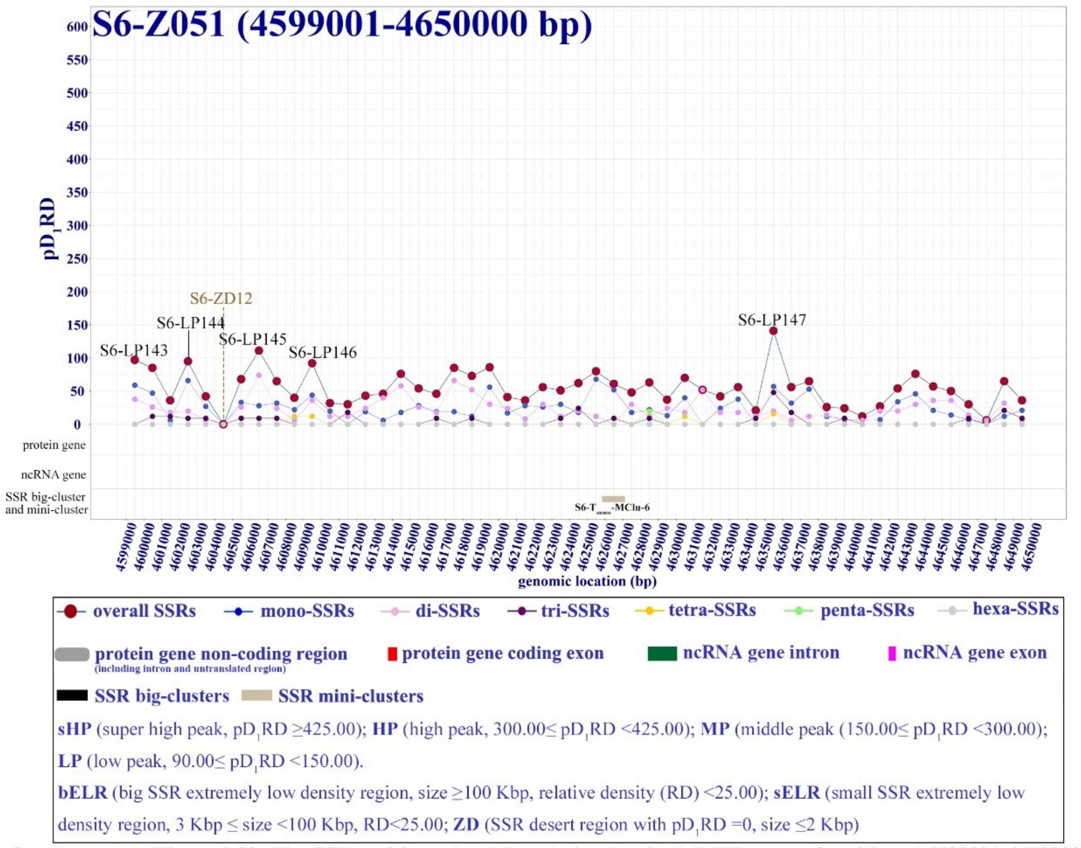

Supplementary Figure 1.89. The SSR position related D_1-relative density (pD_1RD) map of position at 4599001-4650000 bp of human reference Y-DNA (NC_000024.10) at resolution of 1 Kbp.

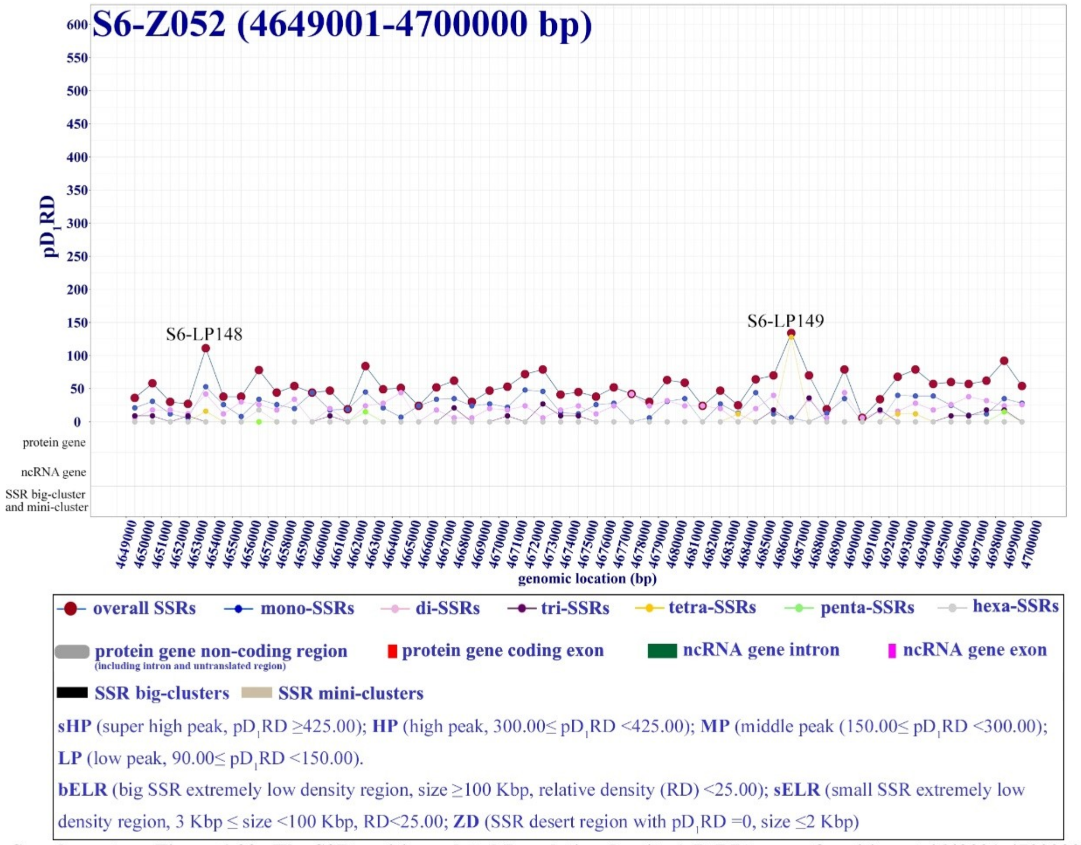

Supplementary Figure 1.90. The SSR position related D_1-relative density (pD_1RD) map of position at 4649001-4700000 bp of human reference Y-DNA (NC_000024.10) at resolution of 1 Kbp.

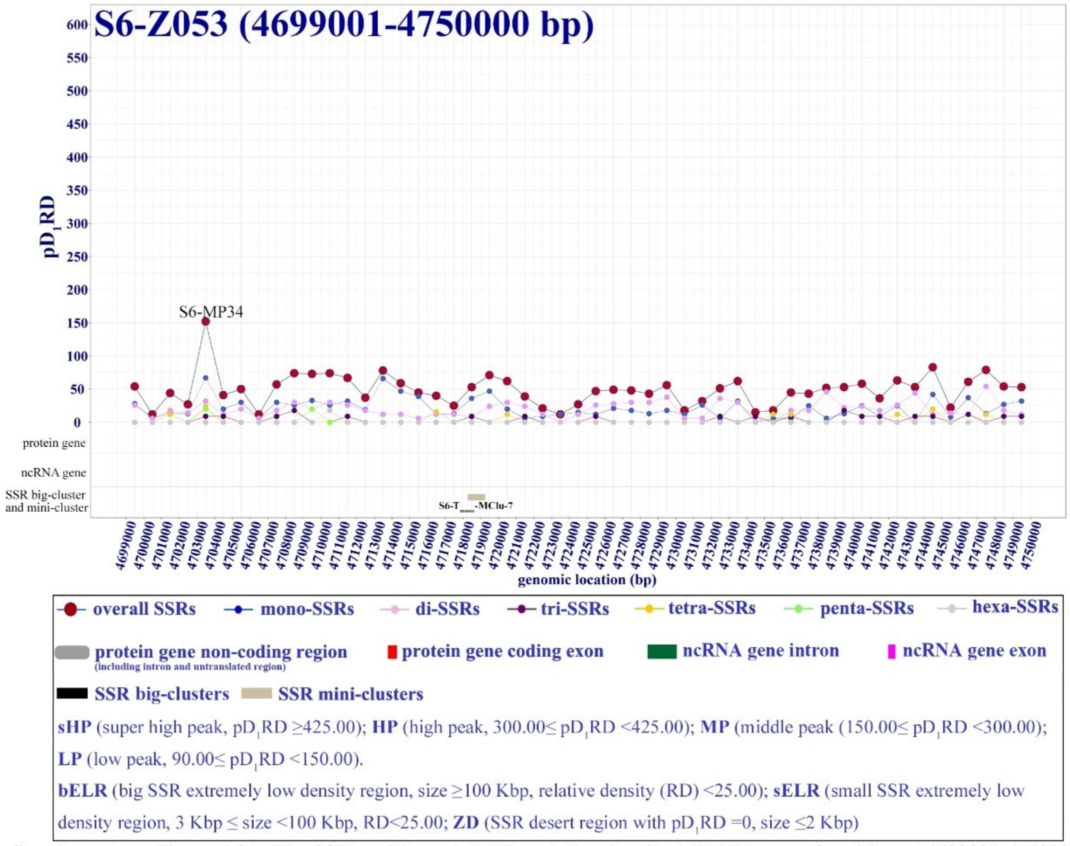

Supplementary Figure 1.91. The SSR position related D_1-relative density (pD_1RD) map of position at 4699001-4750000 bp of human reference Y-DNA (NC_000024.10) at resolution of 1 Kbp.

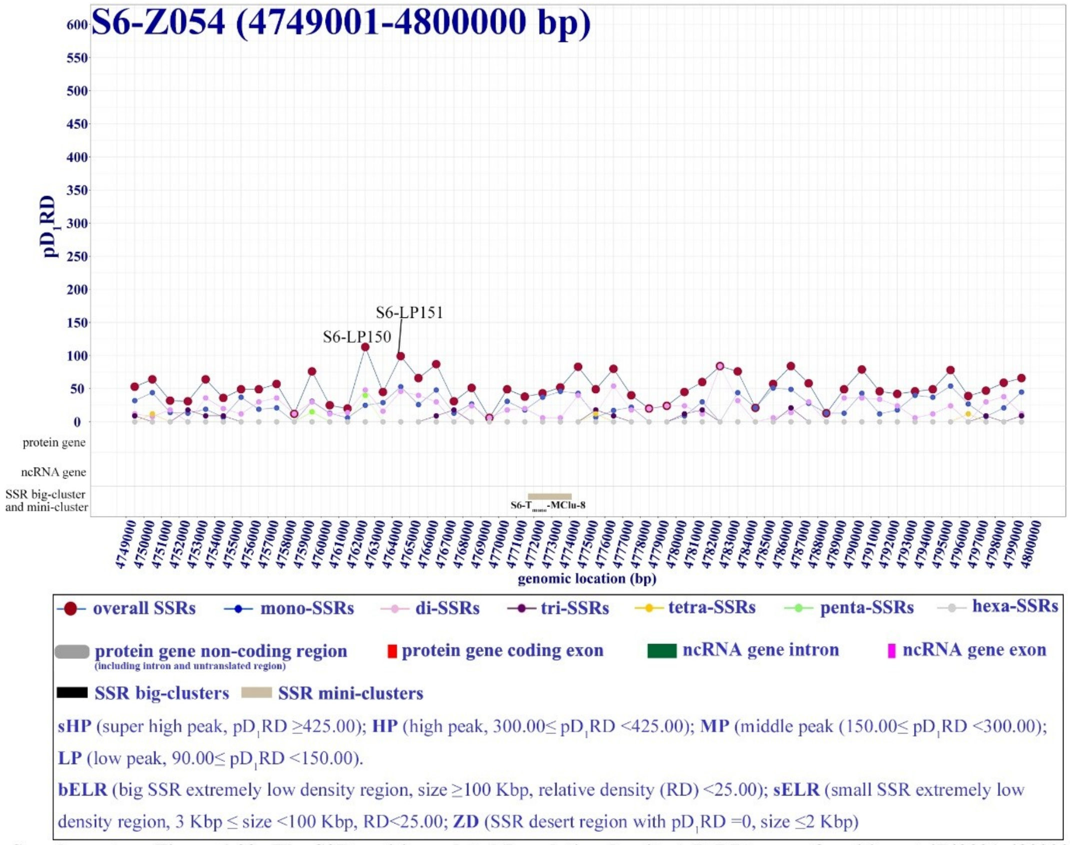

Supplementary Figure 1.92. The SSR position related D_1-relative density (pD_1RD) map of position at 4749001-4800000 bp of human reference Y-DNA (NC_000024.10) at resolution of 1 Kbp.

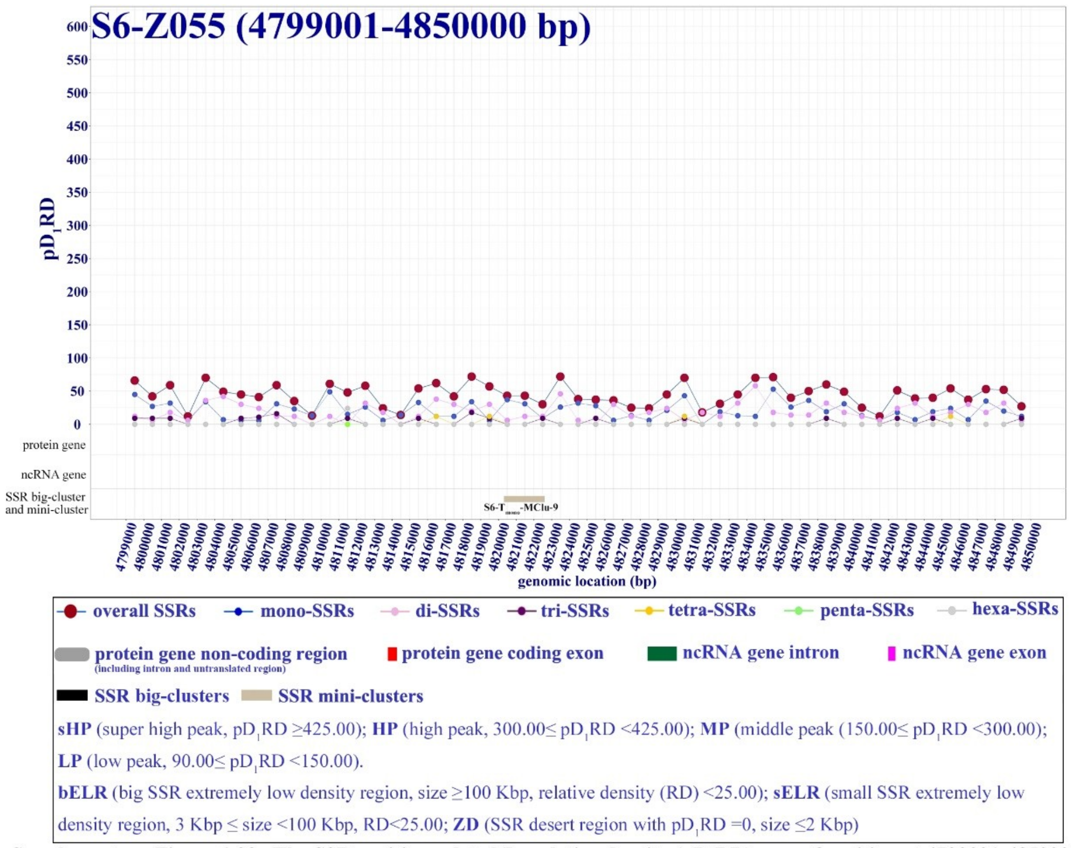

Supplementary Figure 1.93. The SSR position related D_1-relative density (pD_1RD) map of position at 4799001-4850000 bp of human reference Y-DNA (NC_000024.10) at resolution of 1 Kbp.

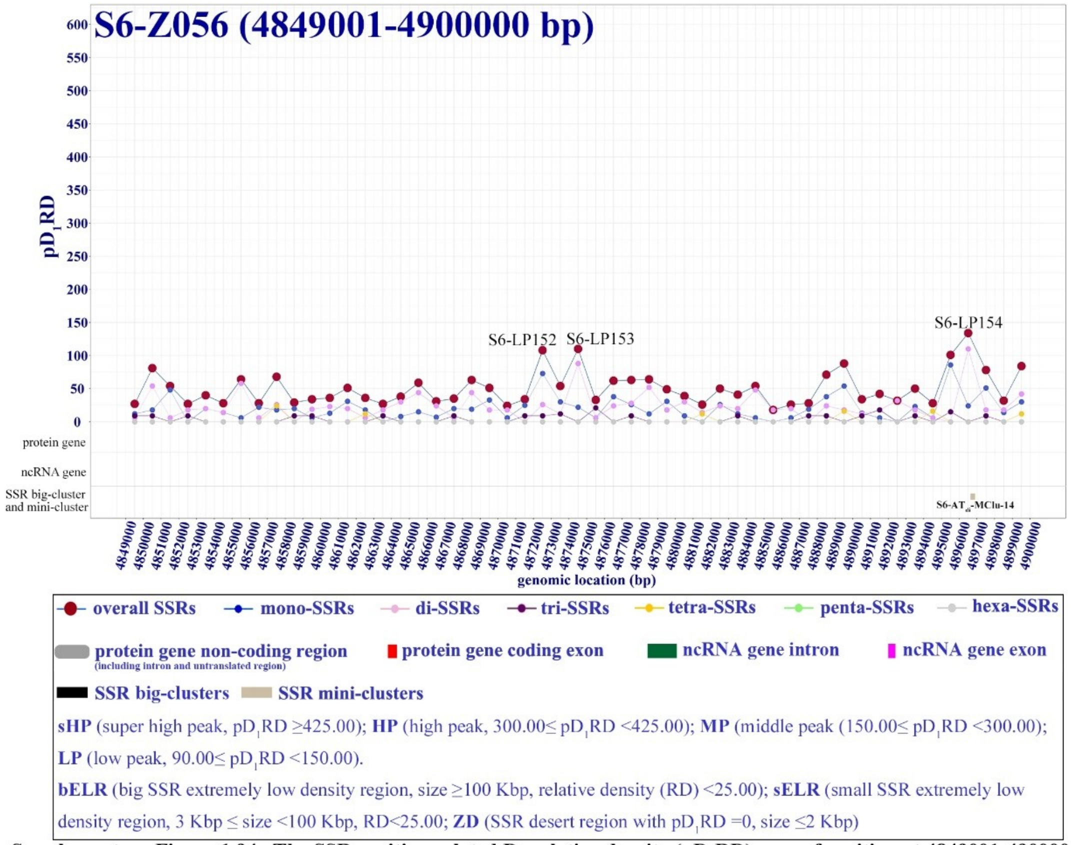

Supplementary Figure 1.94. The SSR position related D_1-relative density (pD_1RD) map of position at 4849001-4900000 bp of human reference Y-DNA (NC_000024.10) at resolution of 1 Kbp.

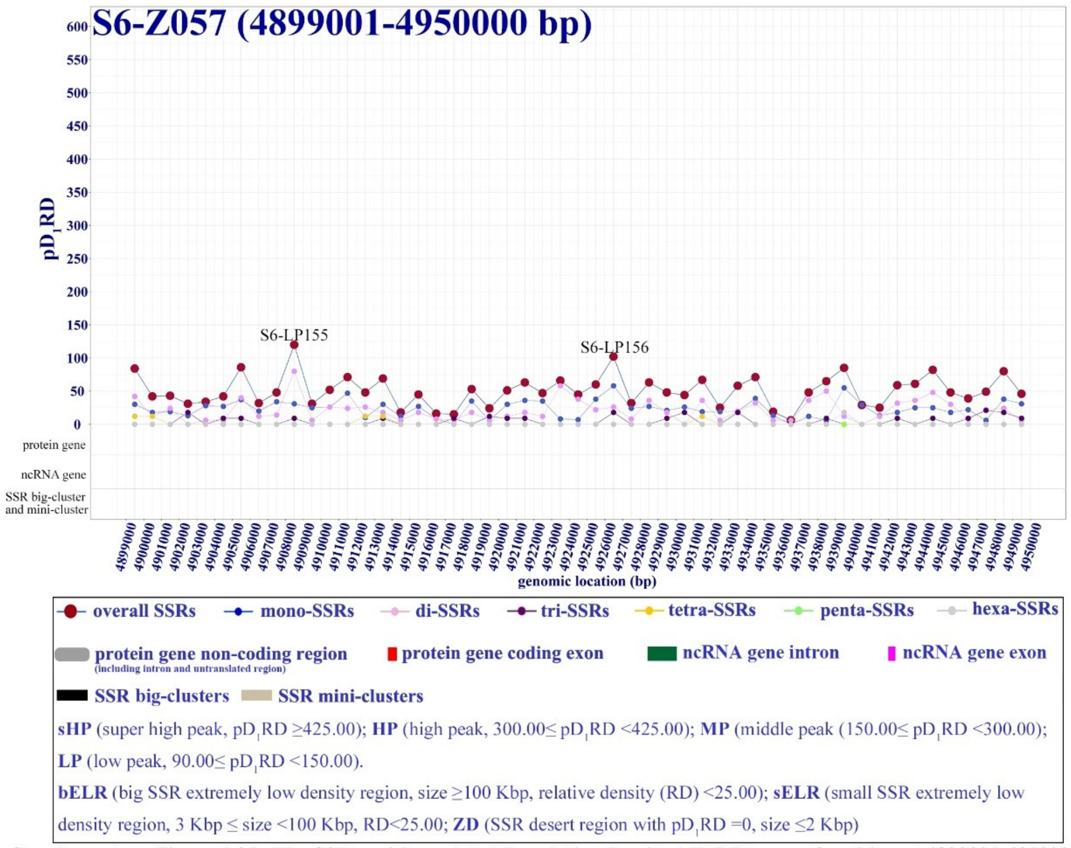

Supplementary Figure 1.95. The SSR position related D_1-relative density (pD_1RD) map of position at 4899001-4950000 bp of human reference Y-DNA (NC_000024.10) at resolution of 1 Kbp.

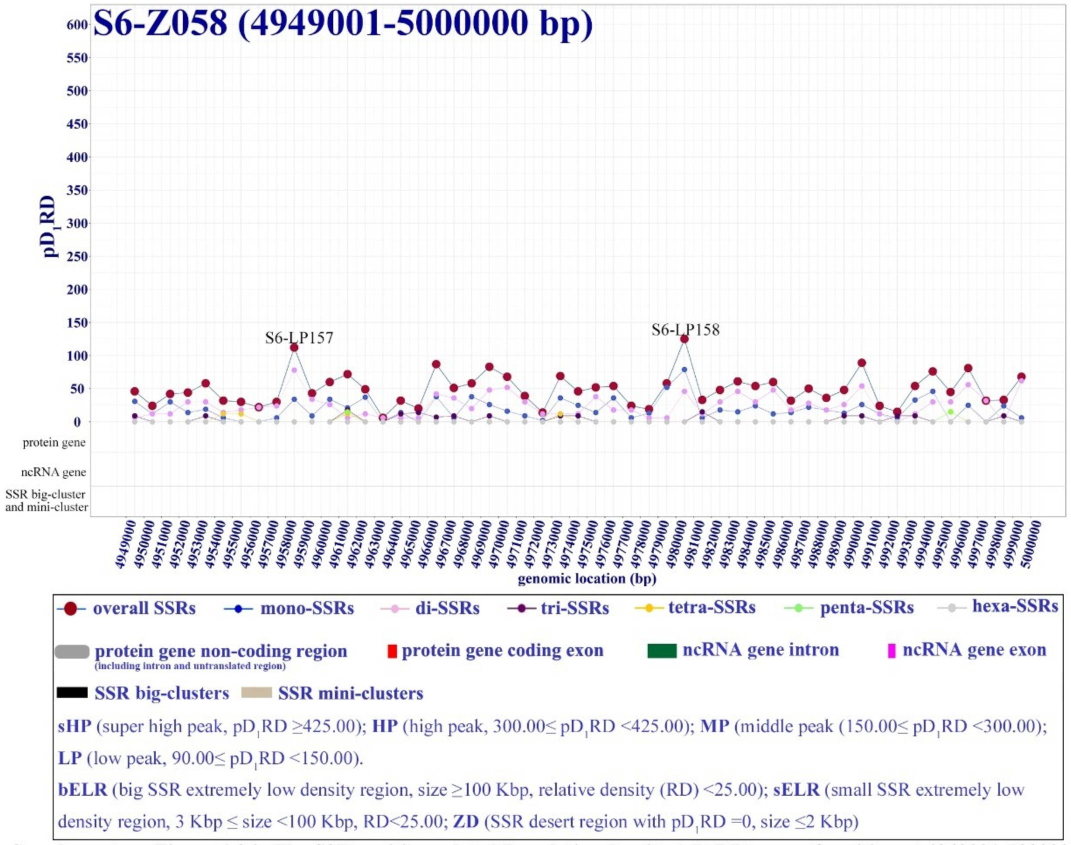

Supplementary Figure 1.96. The SSR position related D_1-relative density (pD_1RD) map of position at 4949001-5000000 bp of human reference Y-DNA (NC_000024.10) at resolution of 1 Kbp.

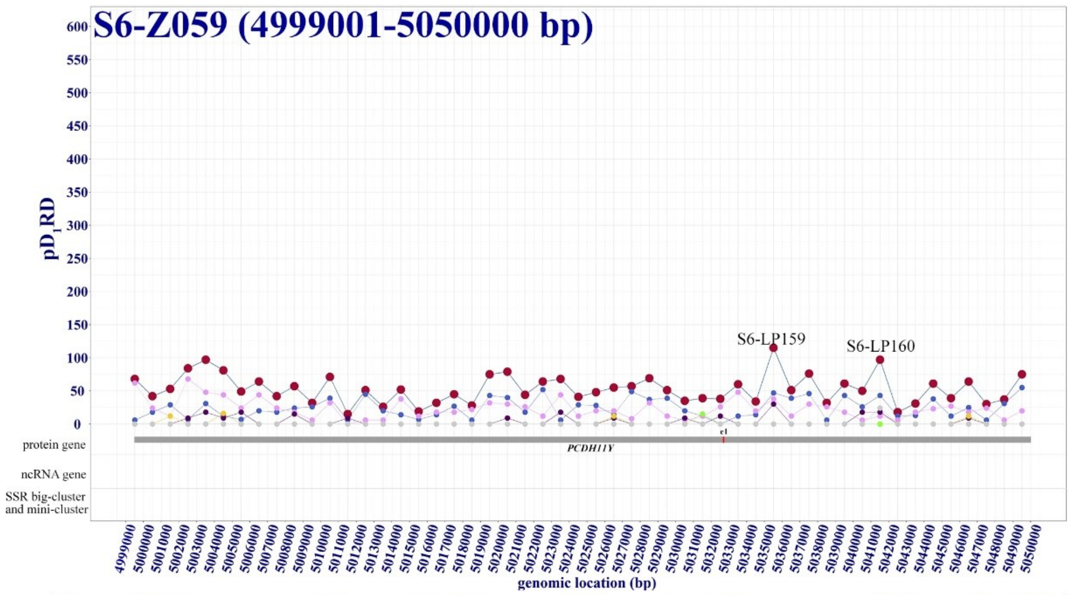

overall SSRs | mono-SSRs | di-SSRs | tri-SSRs | tetra-SSRs | penta-SSRs | hexa-SSRs

protein gene non-coding region (including intron and untranslated region) | protein gene coding exon | ncRNA gene intron | ncRNA gene exon

SSR big-clusters | SSR mini-clusters

sHP (super high peak, $pD_1RD \geq 425.00$); **HP** (high peak, $300.00 \leq pD_1RD < 425.00$); **MP** (middle peak ($150.00 \leq pD_1RD < 300.00$); **LP** (low peak, $90.00 \leq pD_1RD < 150.00$).

bELR (big SSR extremely low density region, size ≥100 Kbp, relative density (RD) <25.00); **sELR** (small SSR extremely low density region, 3 Kbp ≤ size <100 Kbp, RD<25.00; **ZD** (SSR desert region with $pD_1RD = 0$, size ≤2 Kbp)

Supplementary Figure 1.97. The SSR position related D_1-relative density (pD_1RD) map of position at 4999001-5050000 bp of human reference Y-DNA (NC_000024.10) at resolution of 1 Kbp.

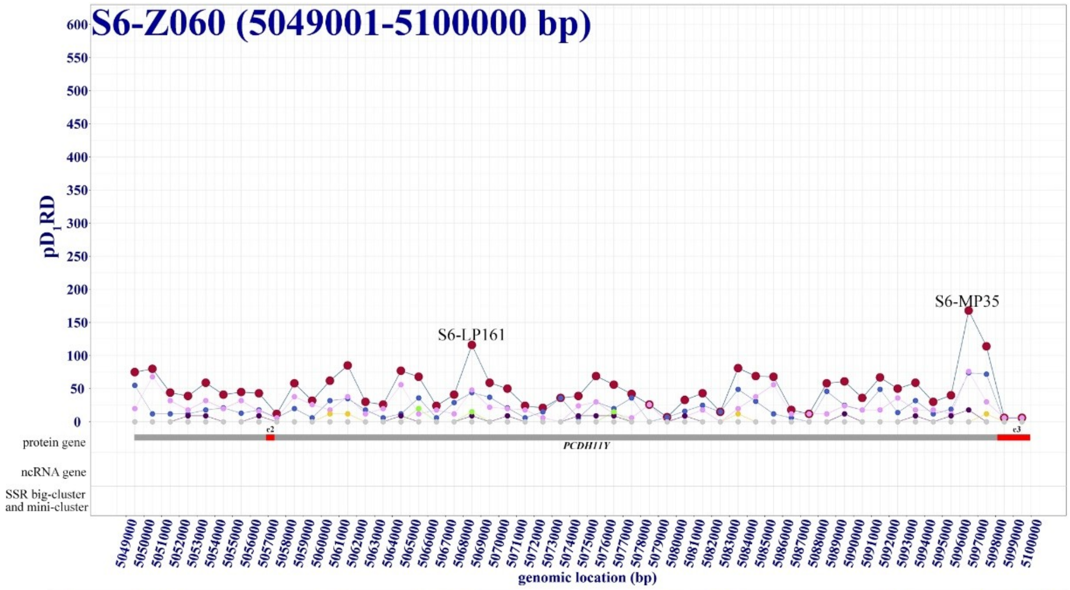

overall SSRs | mono-SSRs | di-SSRs | tri-SSRs | tetra-SSRs | penta-SSRs | hexa-SSRs

protein gene non-coding region (including intron and untranslated region) | protein gene coding exon | ncRNA gene intron | ncRNA gene exon

SSR big-clusters | SSR mini-clusters

sHP (super high peak, $pD_1RD \geq 425.00$); **HP** (high peak, $300.00 \leq pD_1RD < 425.00$); **MP** (middle peak ($150.00 \leq pD_1RD < 300.00$); **LP** (low peak, $90.00 \leq pD_1RD < 150.00$).

bELR (big SSR extremely low density region, size ≥100 Kbp, relative density (RD) <25.00); **sELR** (small SSR extremely low density region, 3 Kbp ≤ size <100 Kbp, RD<25.00; **ZD** (SSR desert region with $pD_1RD = 0$, size ≤2 Kbp)

Supplementary Figure 1.98. The SSR position related D_1-relative density (pD_1RD) map of position at 5049001-5100000 bp of human reference Y-DNA (NC_000024.10) at resolution of 1 Kbp.

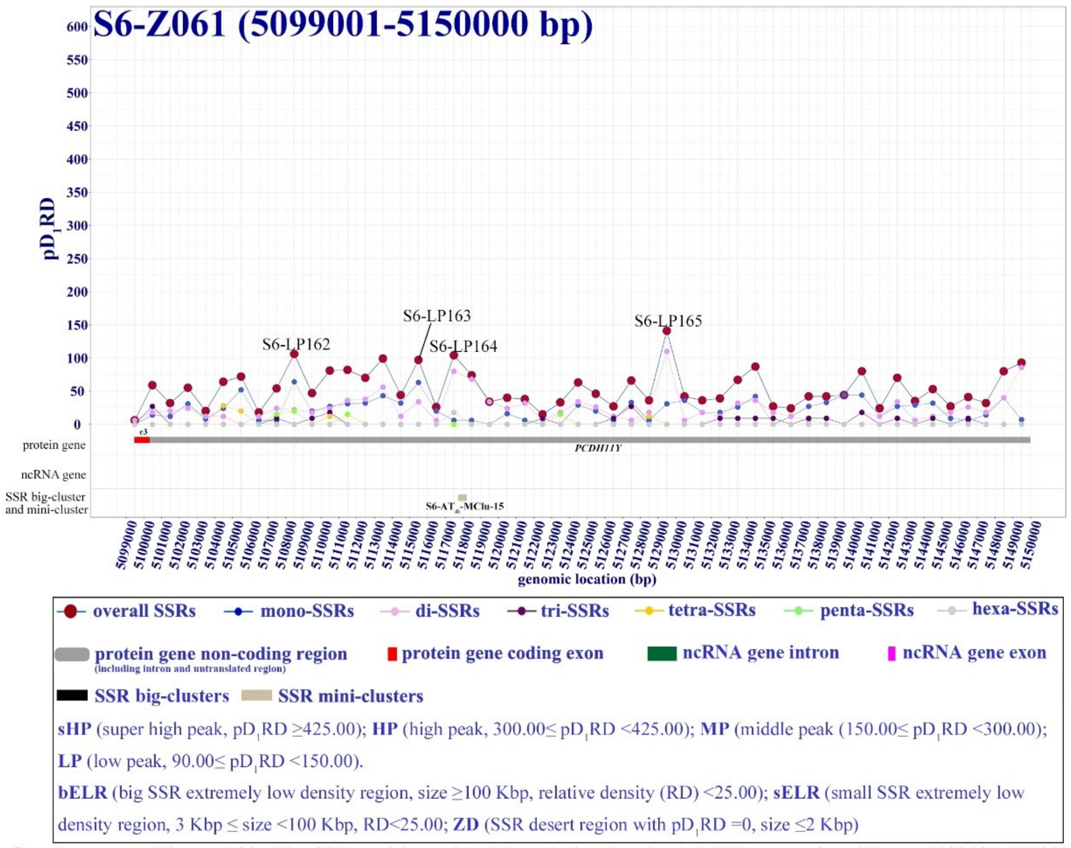

Supplementary Figure 1.99. The SSR position related D_1-relative density (pD_1RD) map of position at 5099001-5150000 bp of human reference Y-DNA (NC_000024.10) at resolution of 1 Kbp.

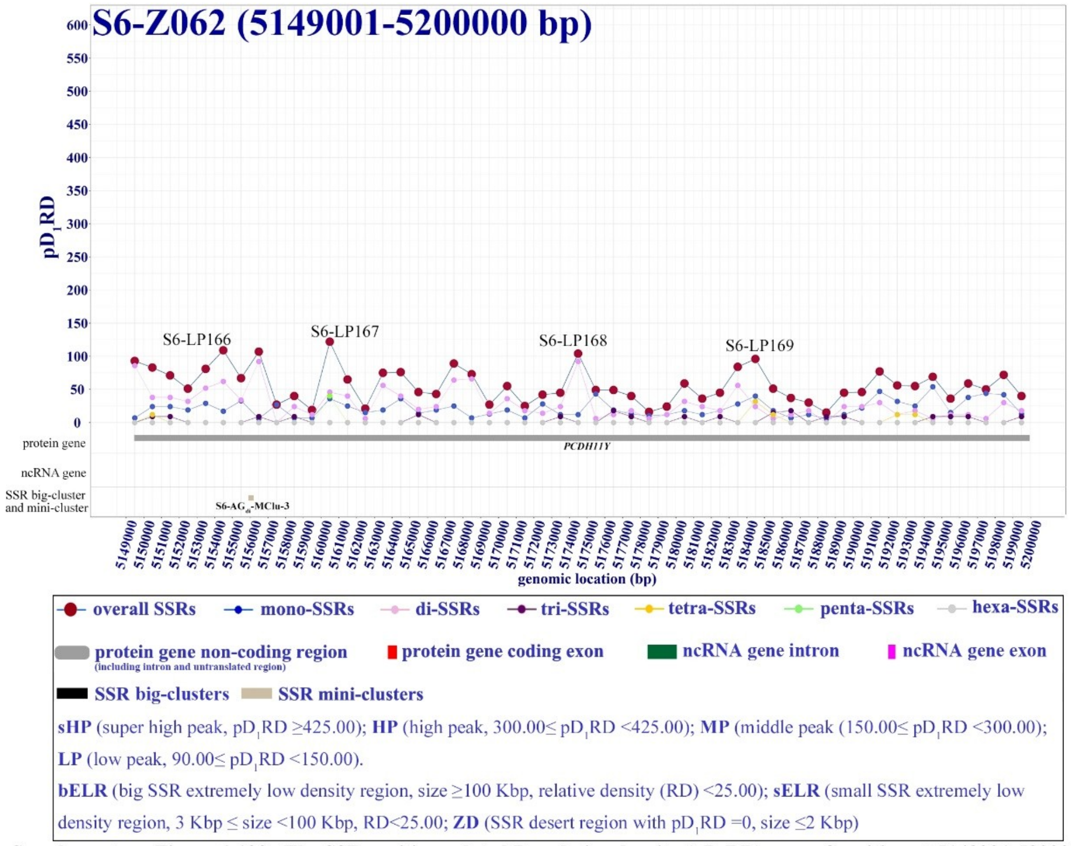

Supplementary Figure 1.100. The SSR position related D_1-relative density (pD_1RD) map of position at 5149001-5200000 bp of human reference Y-DNA (NC_000024.10) at resolution of 1 Kbp.

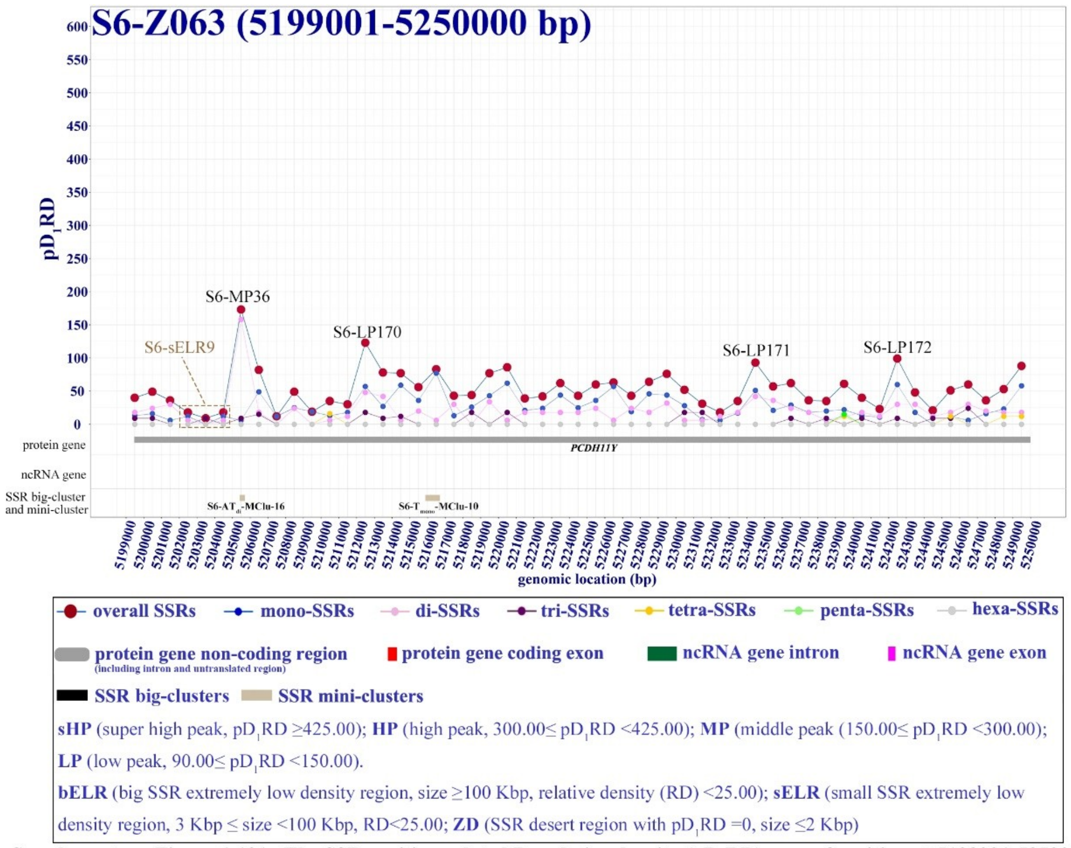

Supplementary Figure 1.101. The SSR position related D_1-relative density (pD_1RD) map of position at 5199001-5250000 bp of human reference Y-DNA (NC_000024.10) at resolution of 1 Kbp.

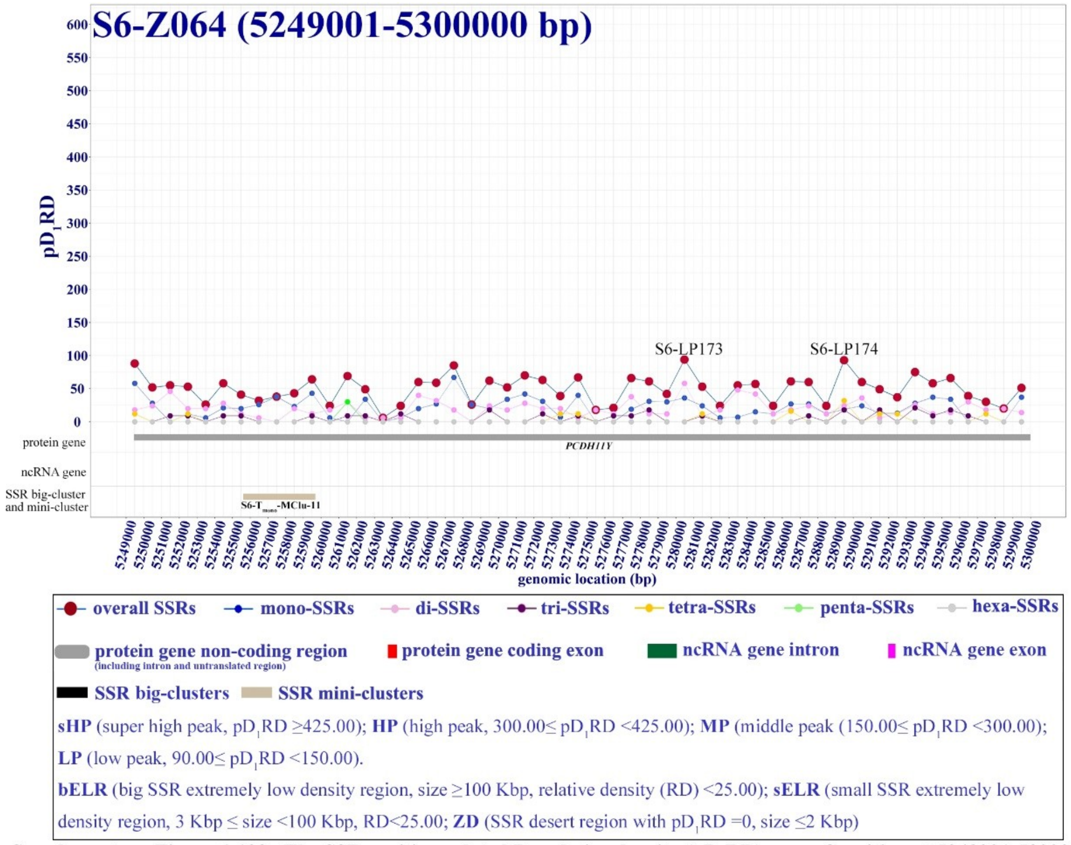

Supplementary Figure 1.102. The SSR position related D_1-relative density (pD_1RD) map of position at 5249001-5300000 bp of human reference Y-DNA (NC_000024.10) at resolution of 1 Kbp.

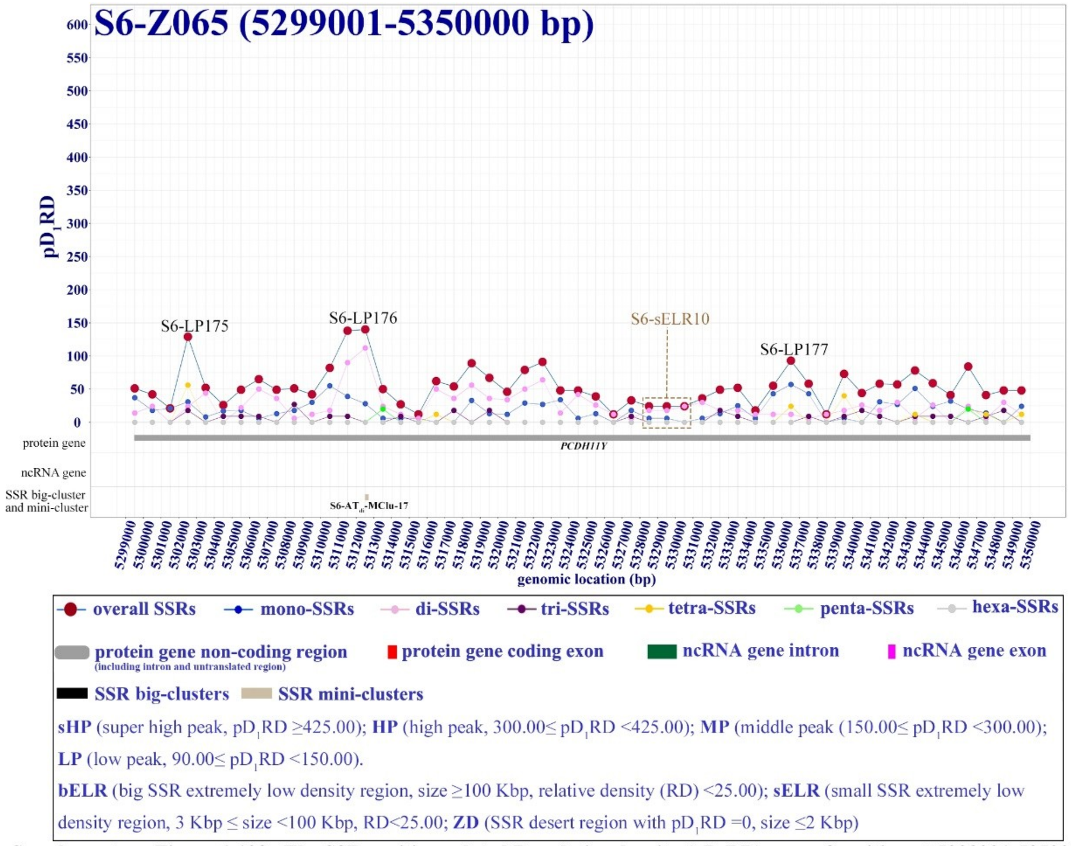

Supplementary Figure 1.103. The SSR position related D_1-relative density (pD_1RD) map of position at 5299001-5350000 bp of human reference Y-DNA (NC_000024.10) at resolution of 1 Kbp.

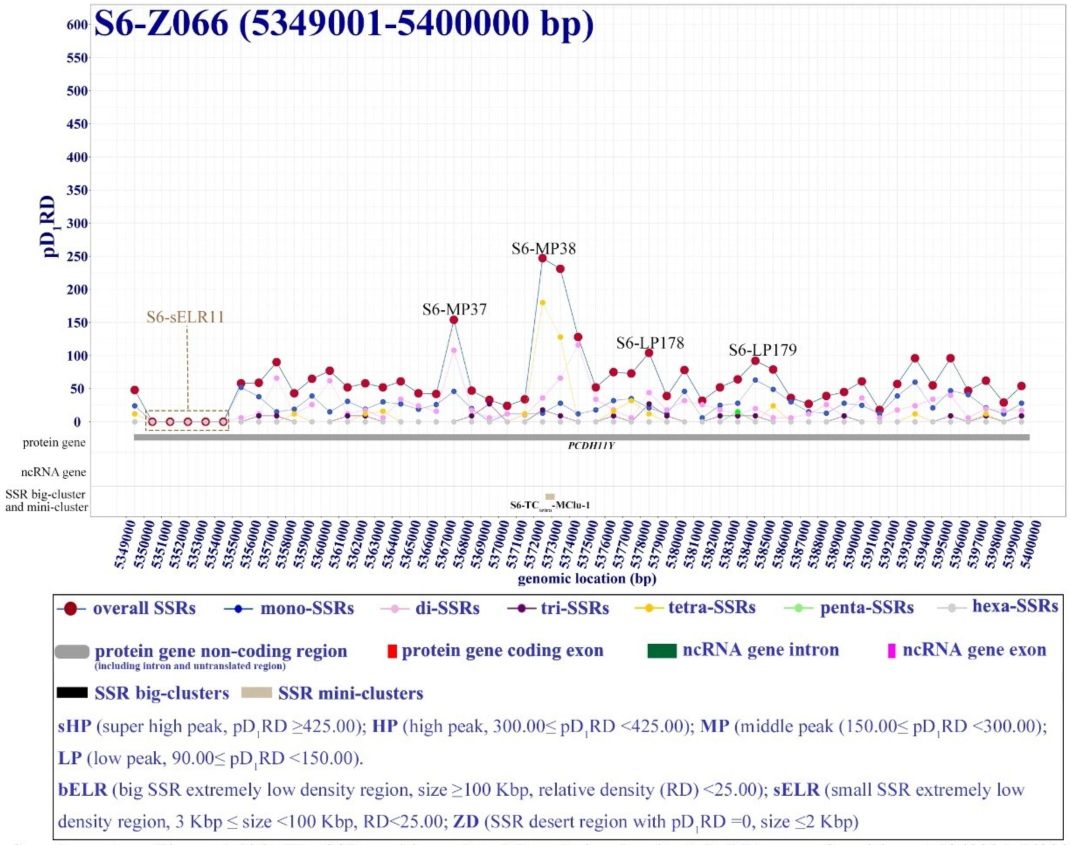

Supplementary Figure 1.104. The SSR position related D_1-relative density (pD_1RD) map of position at 5349001-5400000 bp of human reference Y-DNA (NC_000024.10) at resolution of 1 Kbp.

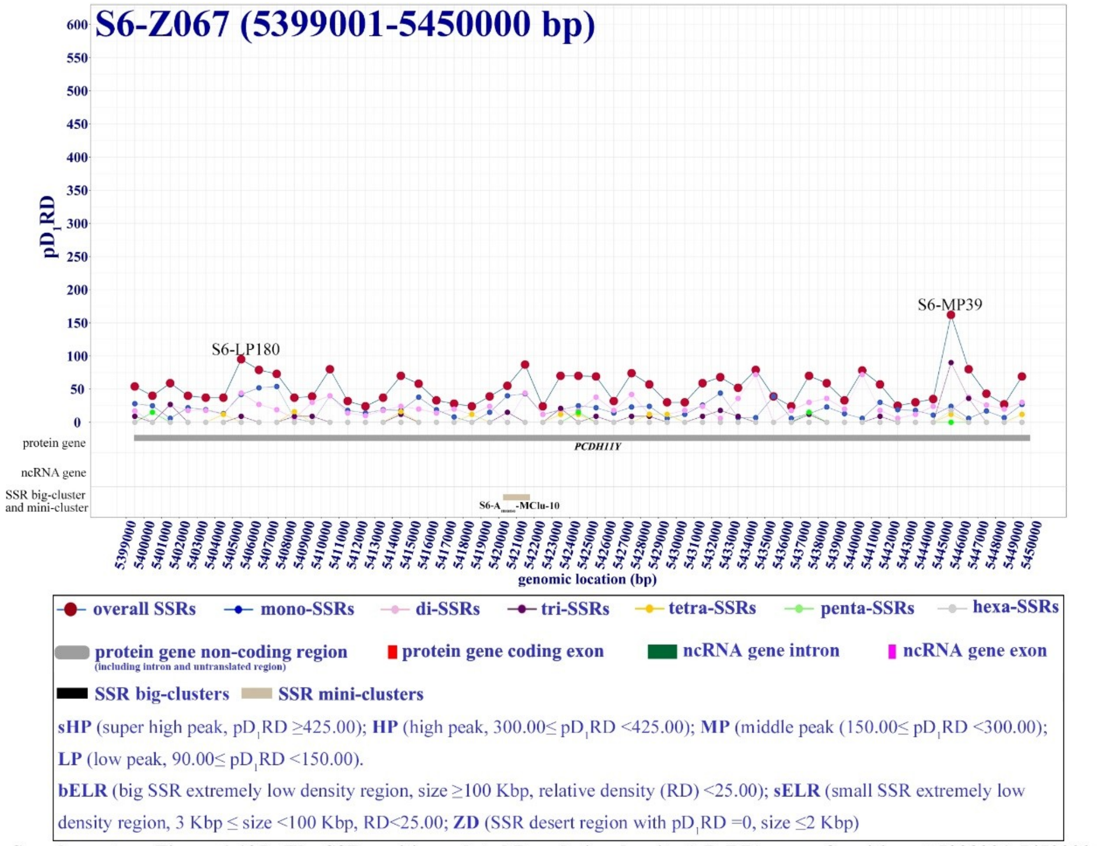

Supplementary Figure 1.105. The SSR position related D_1-relative density (pD_1RD) map of position at 5399001-5450000 bp of human reference Y-DNA (NC_000024.10) at resolution of 1 Kbp.

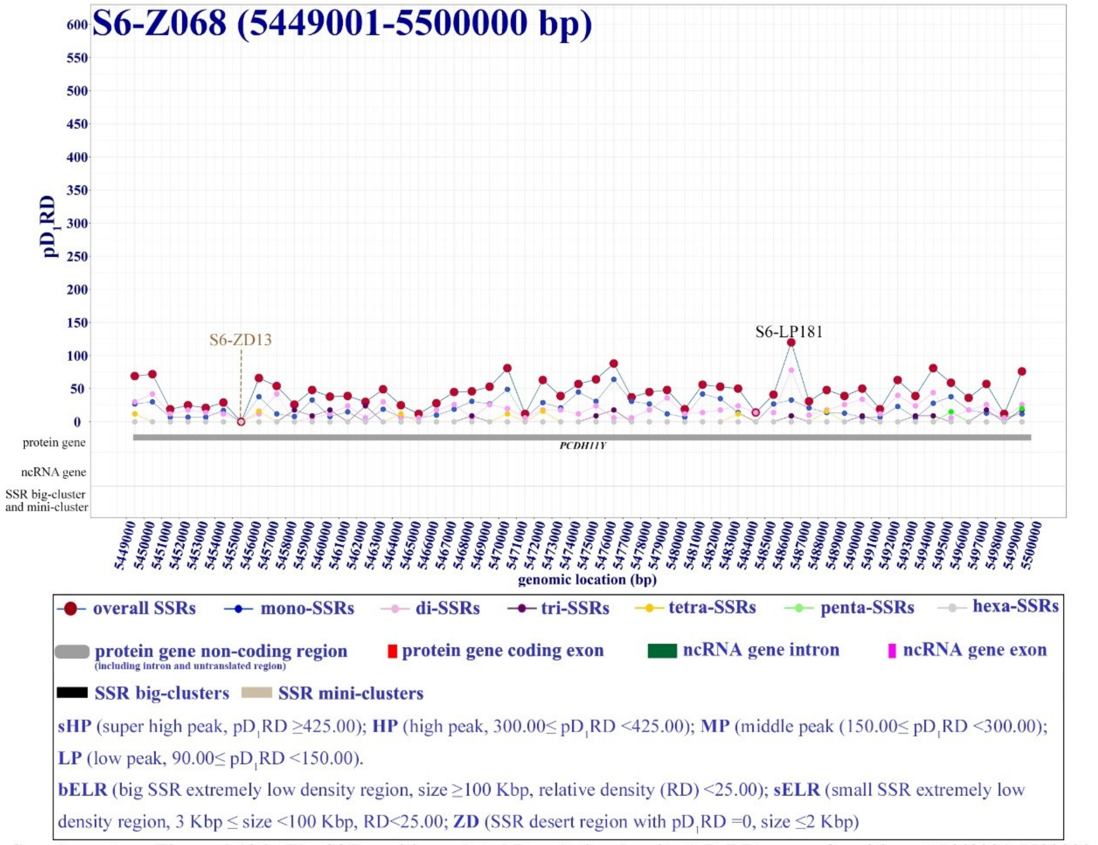

Supplementary Figure 1.106. The SSR position related D_1-relative density (pD_1RD) map of position at 5449001-5500000 bp of human reference Y-DNA (NC_000024.10) at resolution of 1 Kbp.

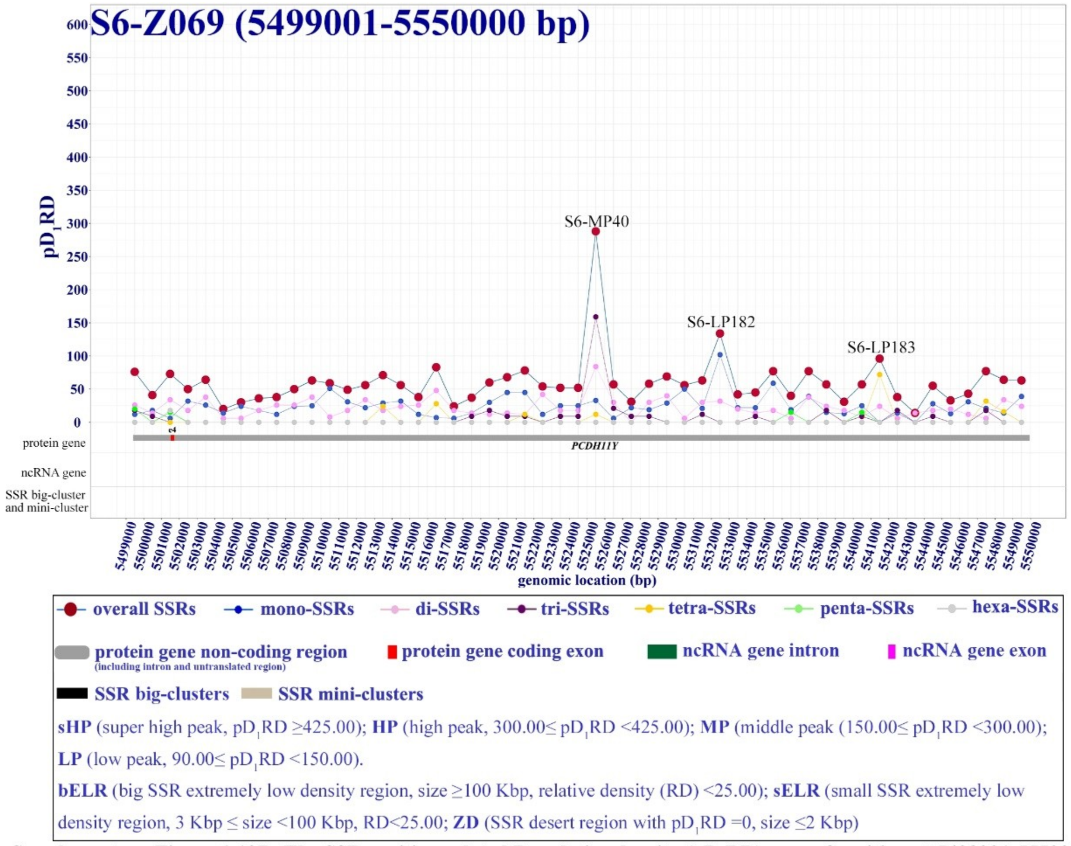

Supplementary Figure 1.107. The SSR position related D_1-relative density (pD_1RD) map of position at 5499001-5550000 bp of human reference Y-DNA (NC_000024.10) at resolution of 1 Kbp.

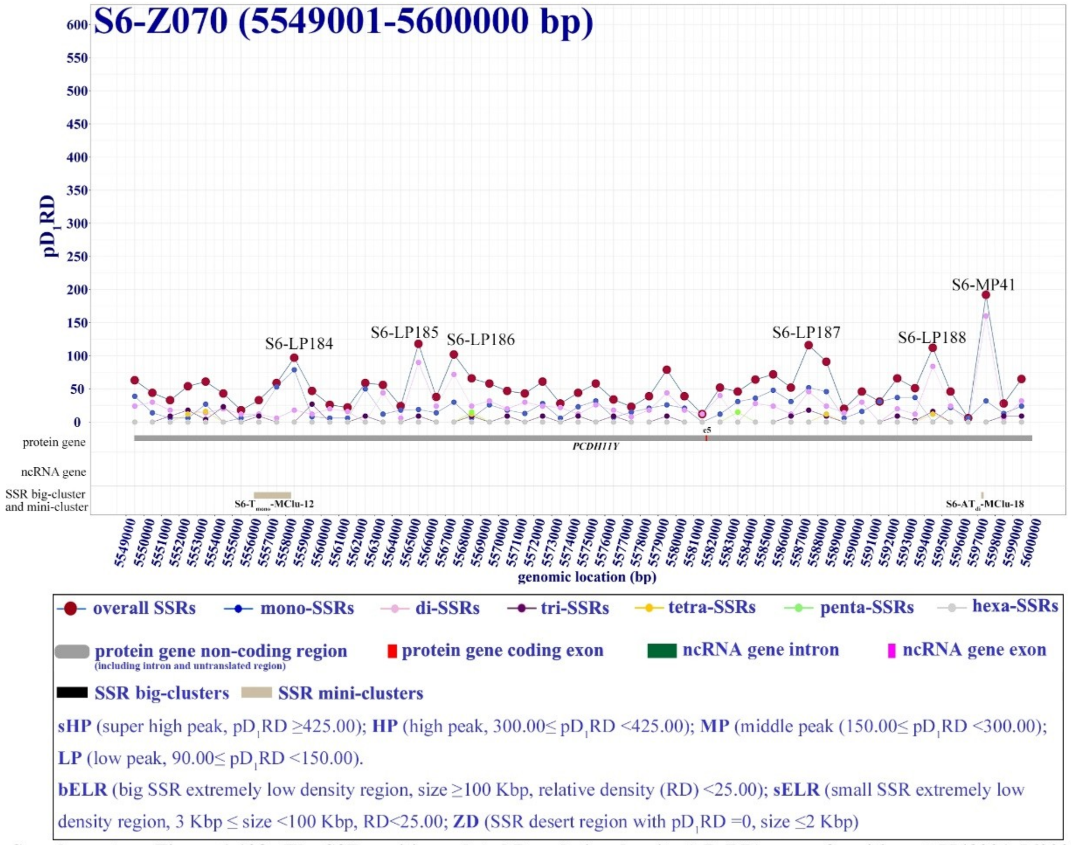

Supplementary Figure 1.108. The SSR position related D_1-relative density (pD_1RD) map of position at 5549001-5600000 bp of human reference Y-DNA (NC_000024.10) at resolution of 1 Kbp.

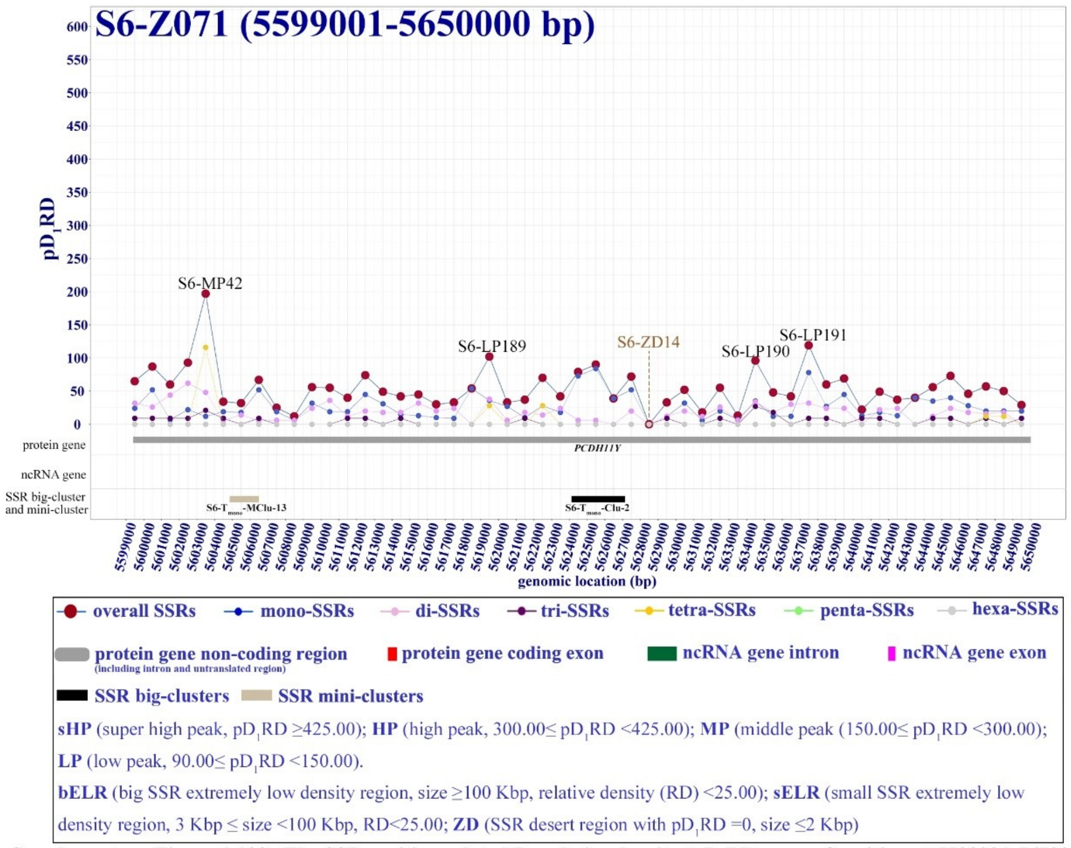

Supplementary Figure 1.109. The SSR position related D_1-relative density (pD_1RD) map of position at 5599001-5650000 bp of human reference Y-DNA (NC_000024.10) at resolution of 1 Kbp.

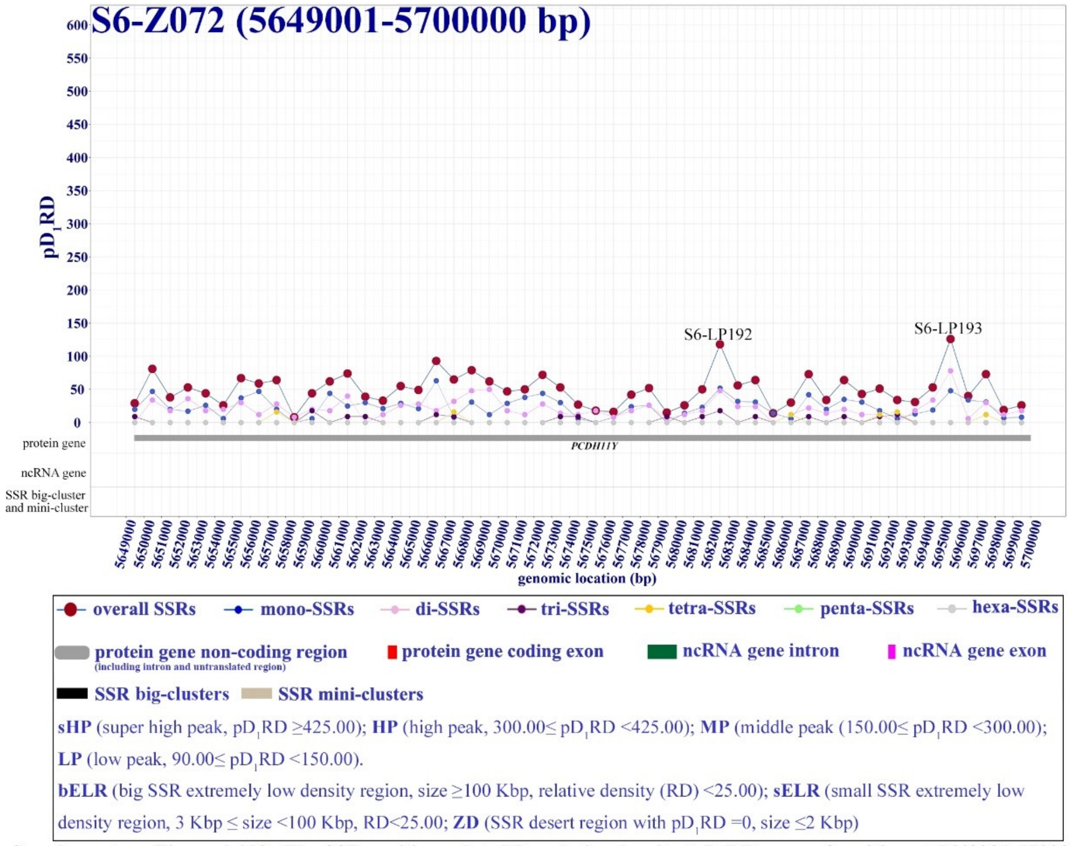

Supplementary Figure 1.110. The SSR position related D_1-relative density (pD_1RD) map of position at 5649001-5700000 bp of human reference Y-DNA (NC_000024.10) at resolution of 1 Kbp.

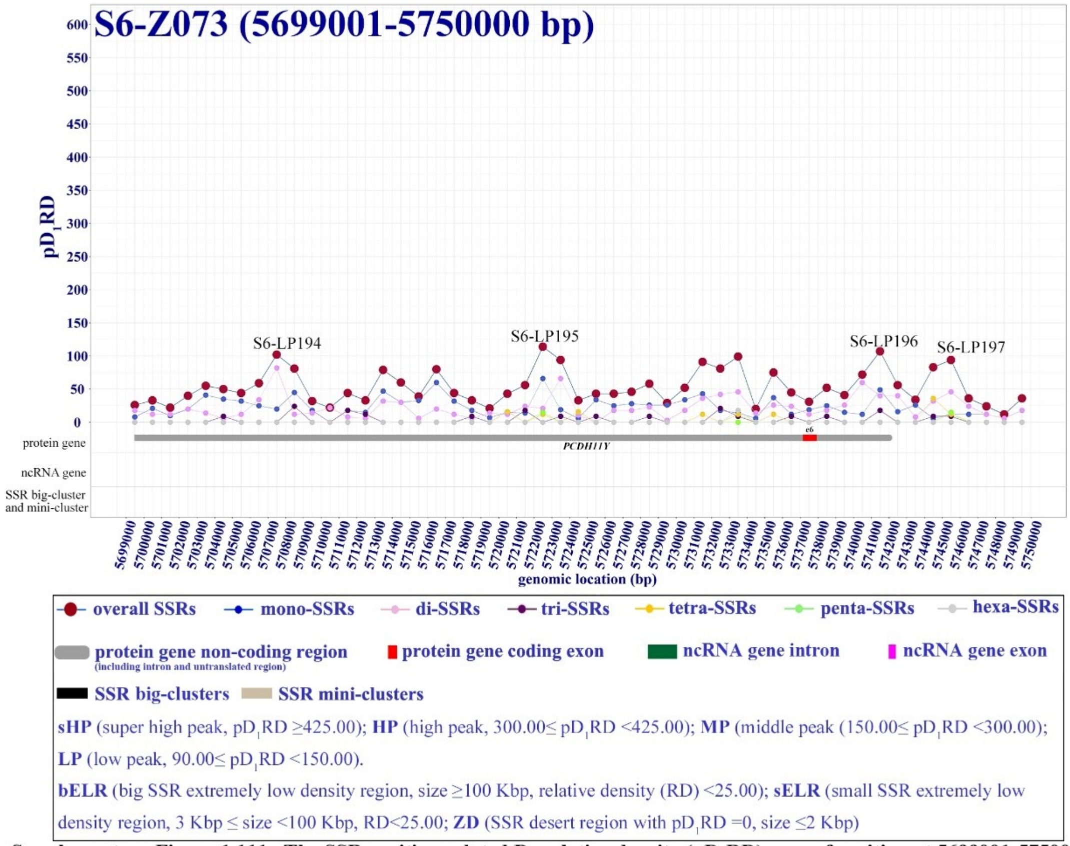

Supplementary Figure 1.111. The SSR position related D_1-relative density (pD_1RD) map of position at 5699001-5750000 bp of human reference Y-DNA (NC_000024.10) at resolution of 1 Kbp.

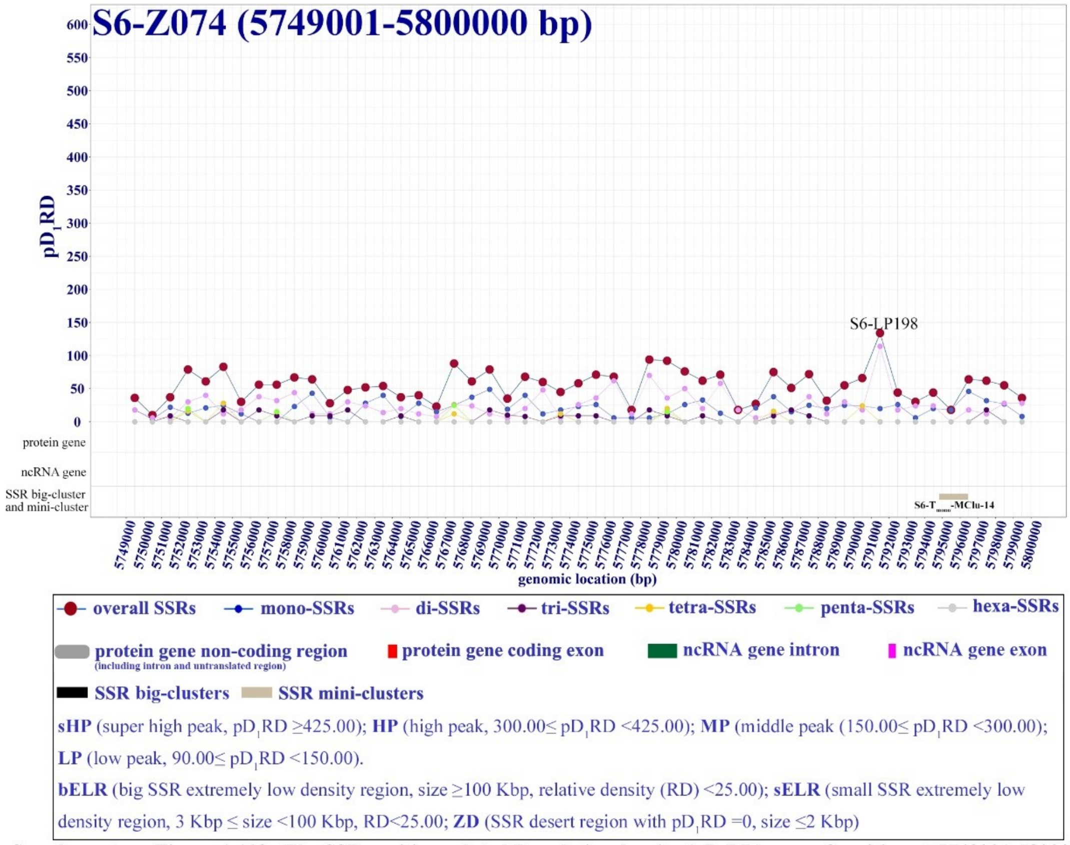

Supplementary Figure 1.112. The SSR position related D_1-relative density (pD_1RD) map of position at 5749001-5800000 bp of human reference Y-DNA (NC_000024.10) at resolution of 1 Kbp.

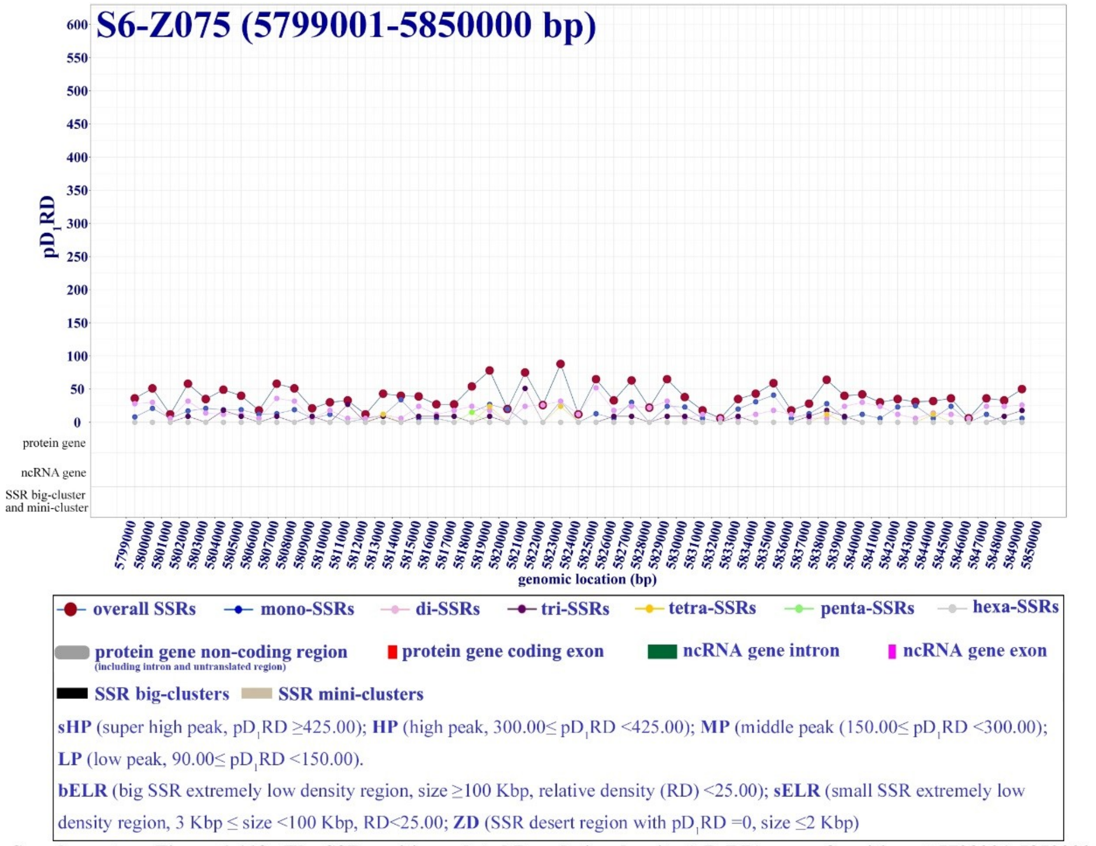

Supplementary Figure 1.113. The SSR position related D_1-relative density (pD_1RD) map of position at 5799001-5850000 bp of human reference Y-DNA (NC_000024.10) at resolution of 1 Kbp.

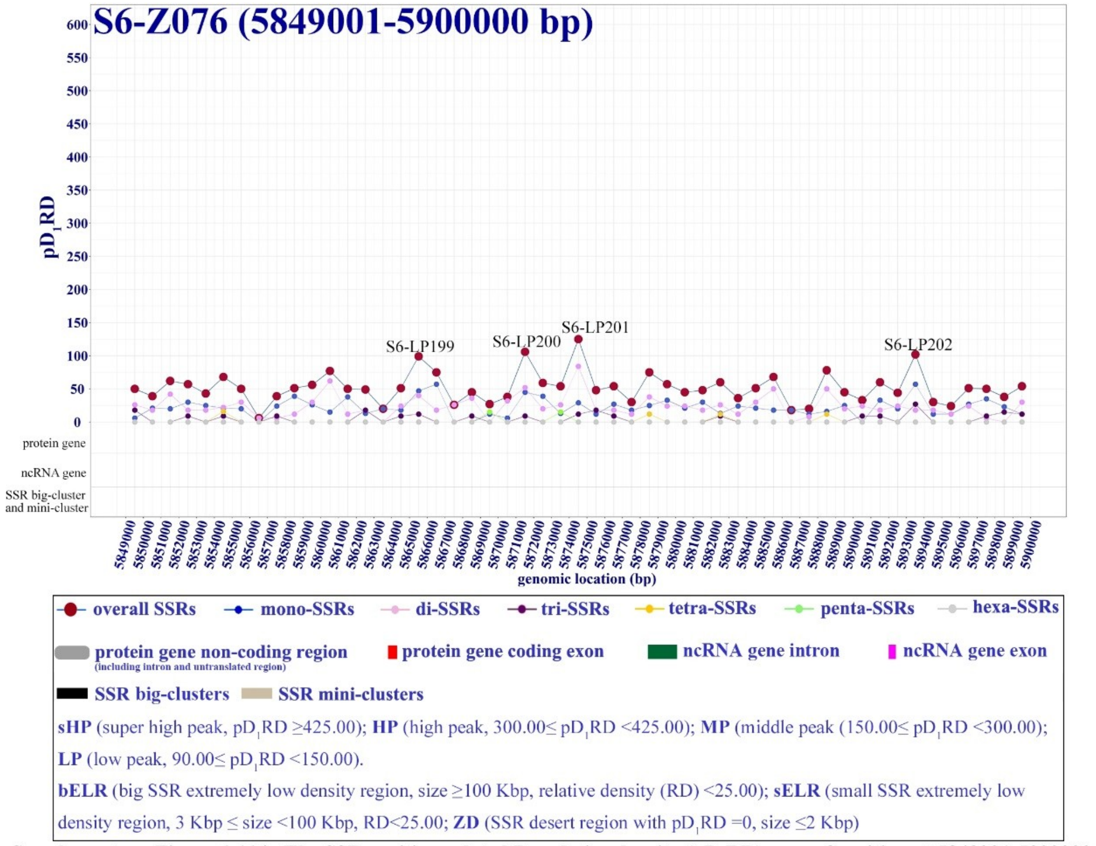

Supplementary Figure 1.114. The SSR position related D_1-relative density (pD_1RD) map of position at 5849001-5900000 bp of human reference Y-DNA (NC_000024.10) at resolution of 1 Kbp.

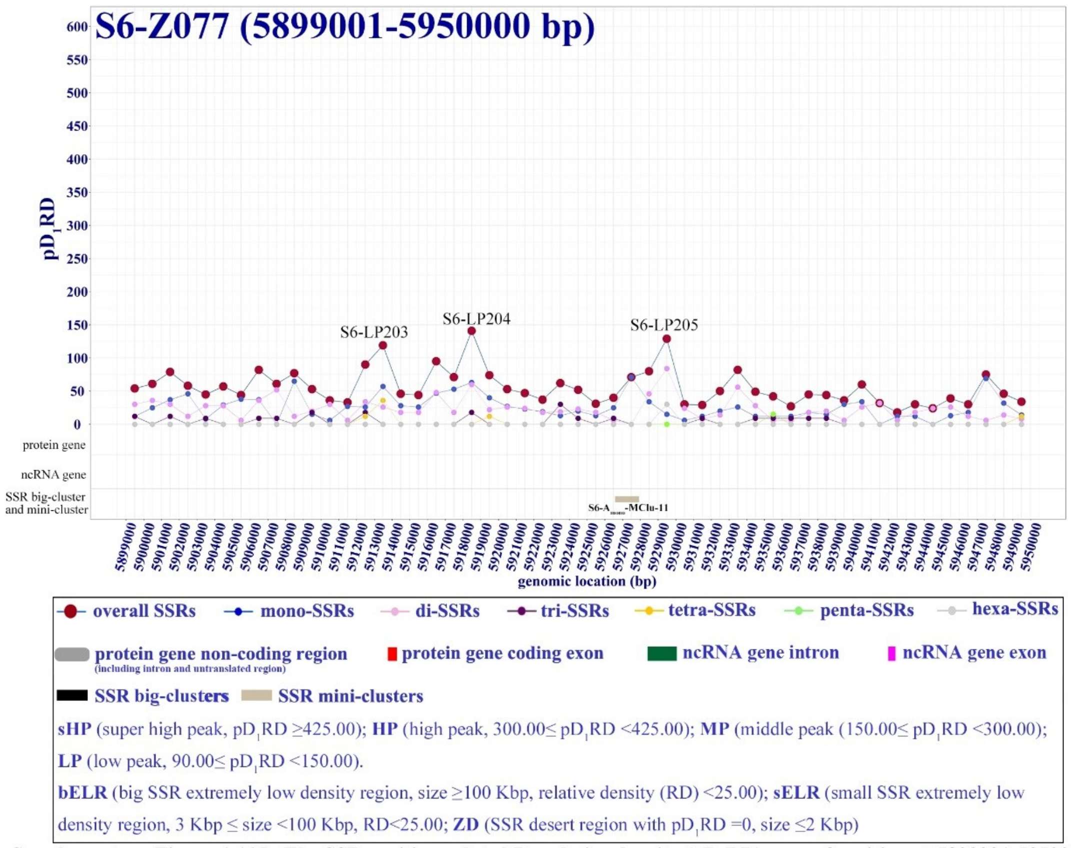

Supplementary Figure 1.115. The SSR position related D_1-relative density (pD_1RD) map of position at 5899001-5950000 bp of human reference Y-DNA (NC_000024.10) at resolution of 1 Kbp.

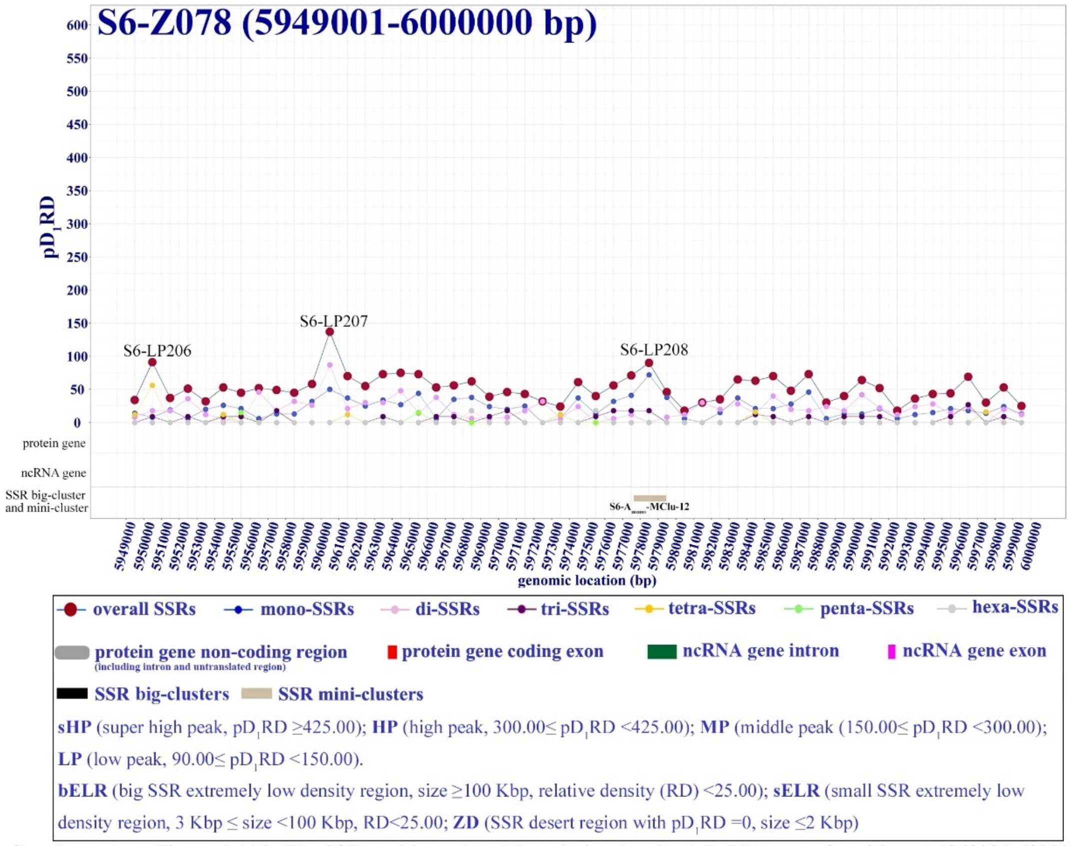

Supplementary Figure 1.116. The SSR position related D_1-relative density (pD_1RD) map of position at 5949001-6000000 bp of human reference Y-DNA (NC_000024.10) at resolution of 1 Kbp.

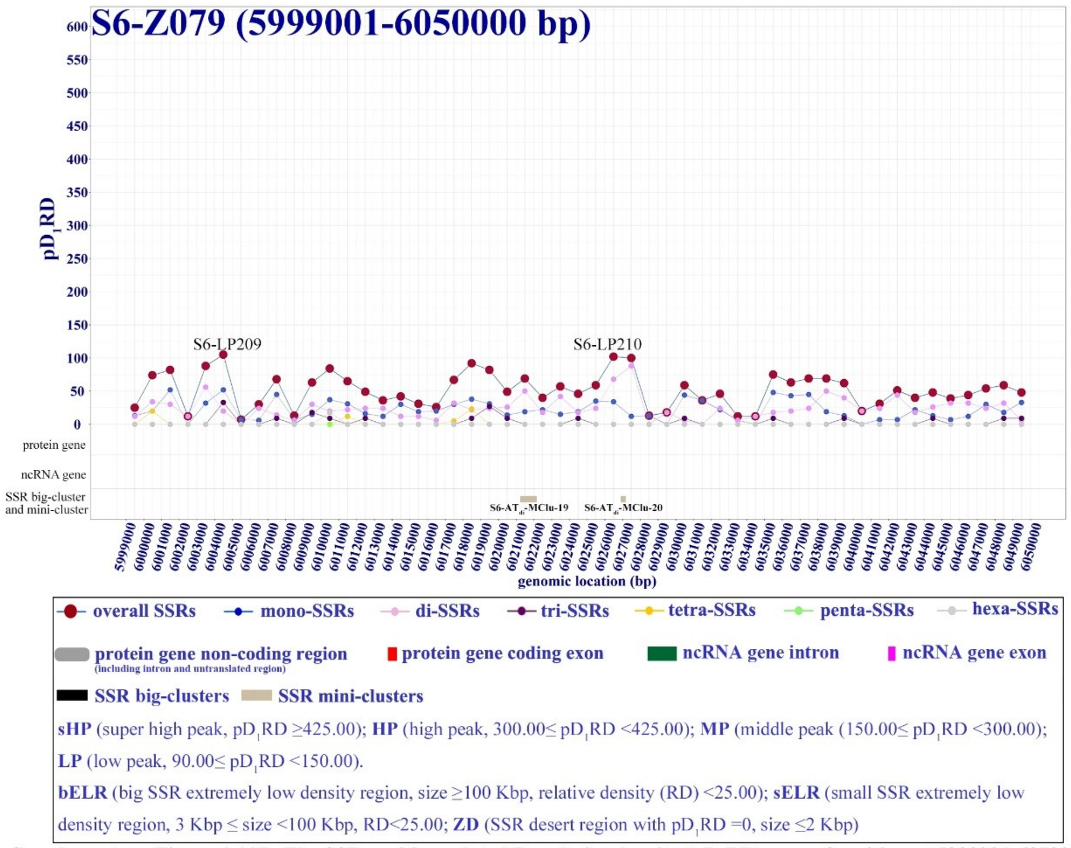

Supplementary Figure 1.117. The SSR position related D_1-relative density (pD_1RD) map of position at 5999001-6050000 bp of human reference Y-DNA (NC_000024.10) at resolution of 1 Kbp.

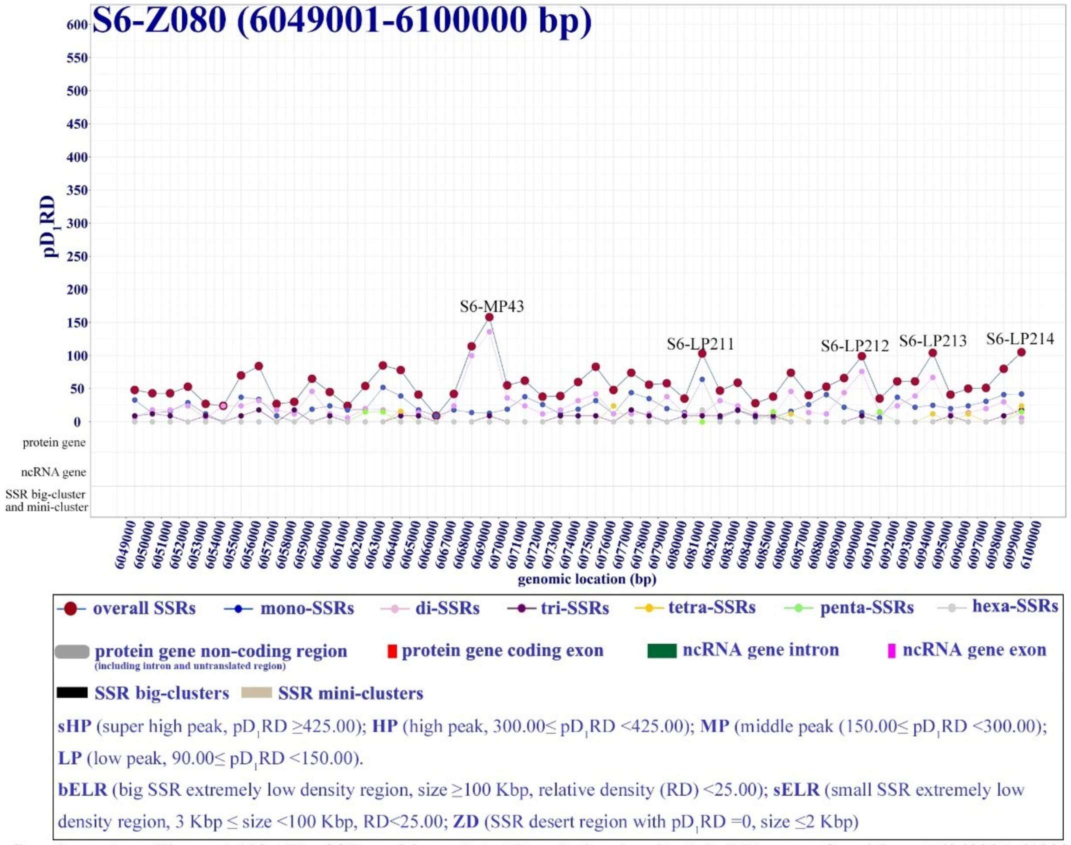

Supplementary Figure 1.118. The SSR position related D_1-relative density (pD_1RD) map of position at 6049001-6100000 bp of human reference Y-DNA (NC_000024.10) at resolution of 1 Kbp.

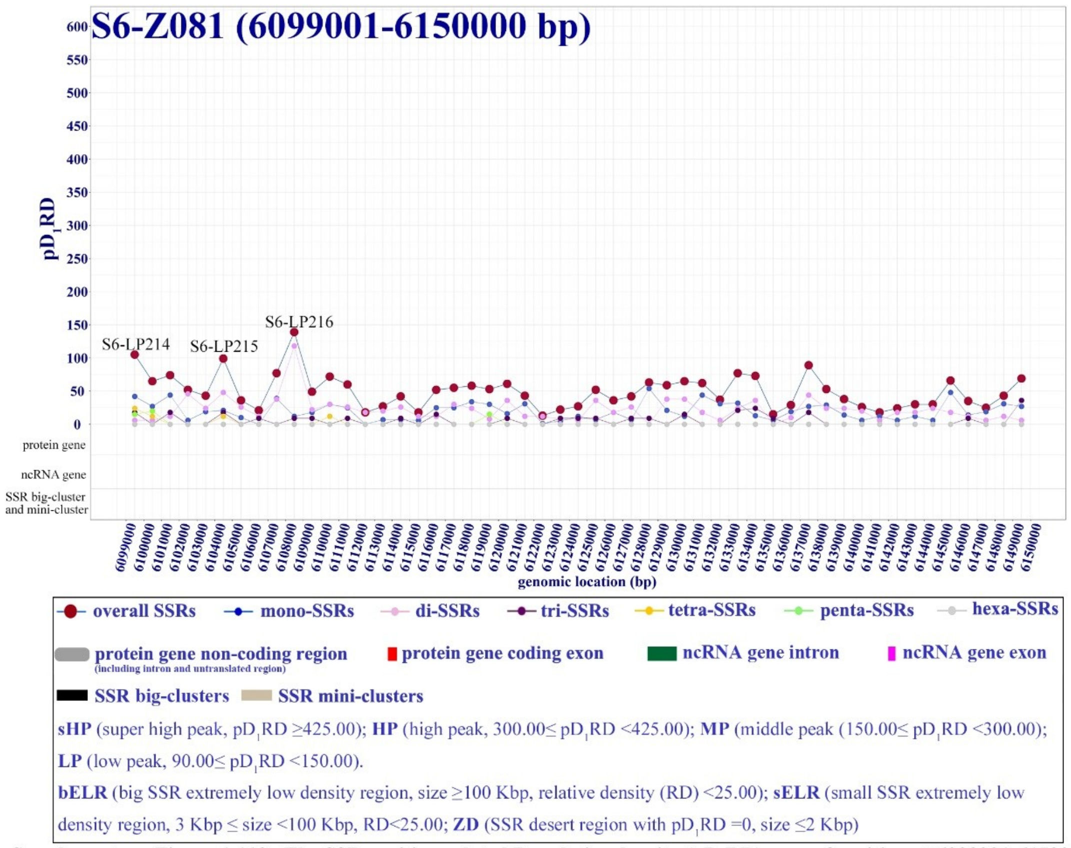

Supplementary Figure 1.119. The SSR position related D_1-relative density (pD_1RD) map of position at 6099001-6150000 bp of human reference Y-DNA (NC_000024.10) at resolution of 1 Kbp.

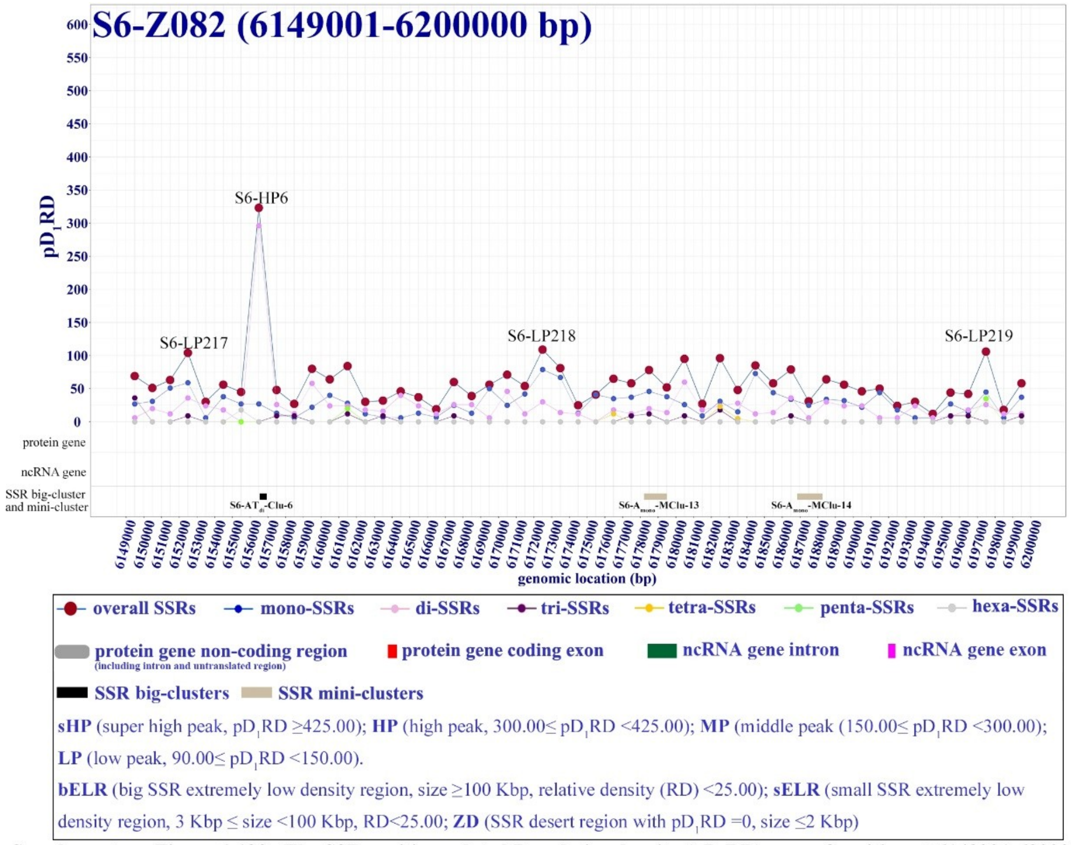

Supplementary Figure 1.120. The SSR position related D_1-relative density (pD_1RD) map of position at 6149001-6200000 bp of human reference Y-DNA (NC_000024.10) at resolution of 1 Kbp.

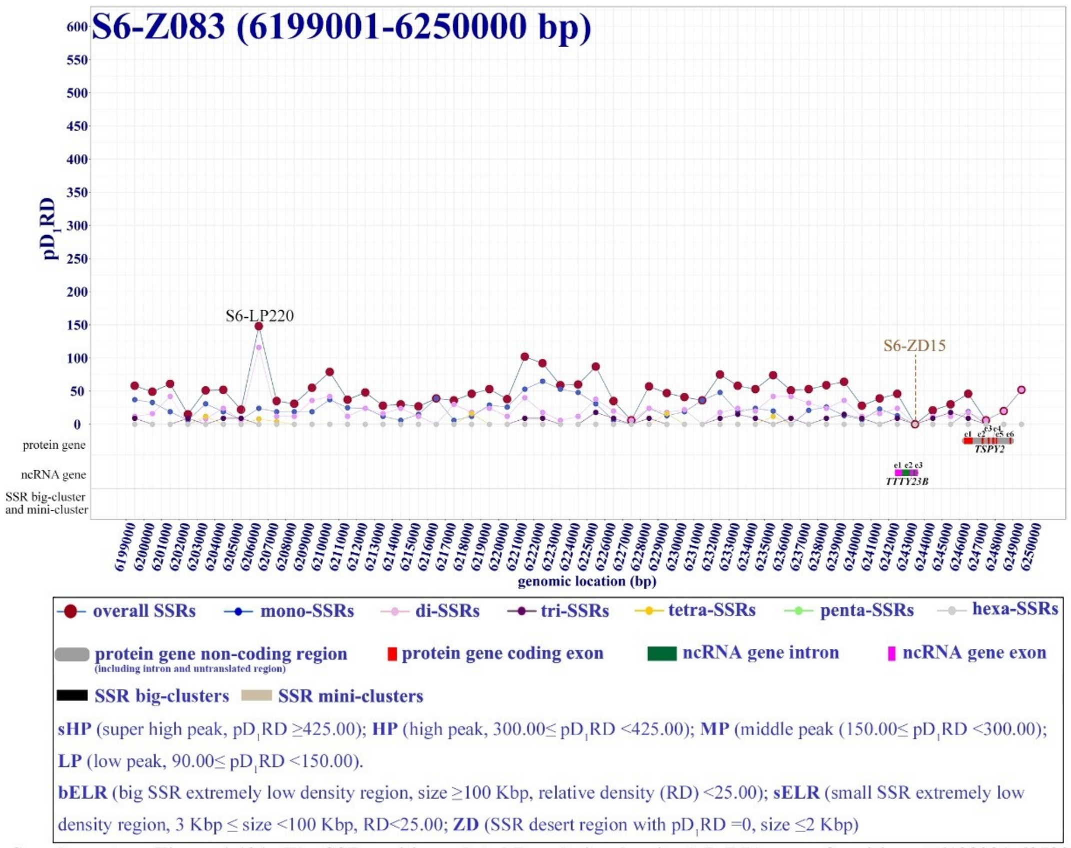

Supplementary Figure 1.121. The SSR position related D_1-relative density (pD_1RD) map of position at 6199001-6250000 bp of human reference Y-DNA (NC_000024.10) at resolution of 1 Kbp.

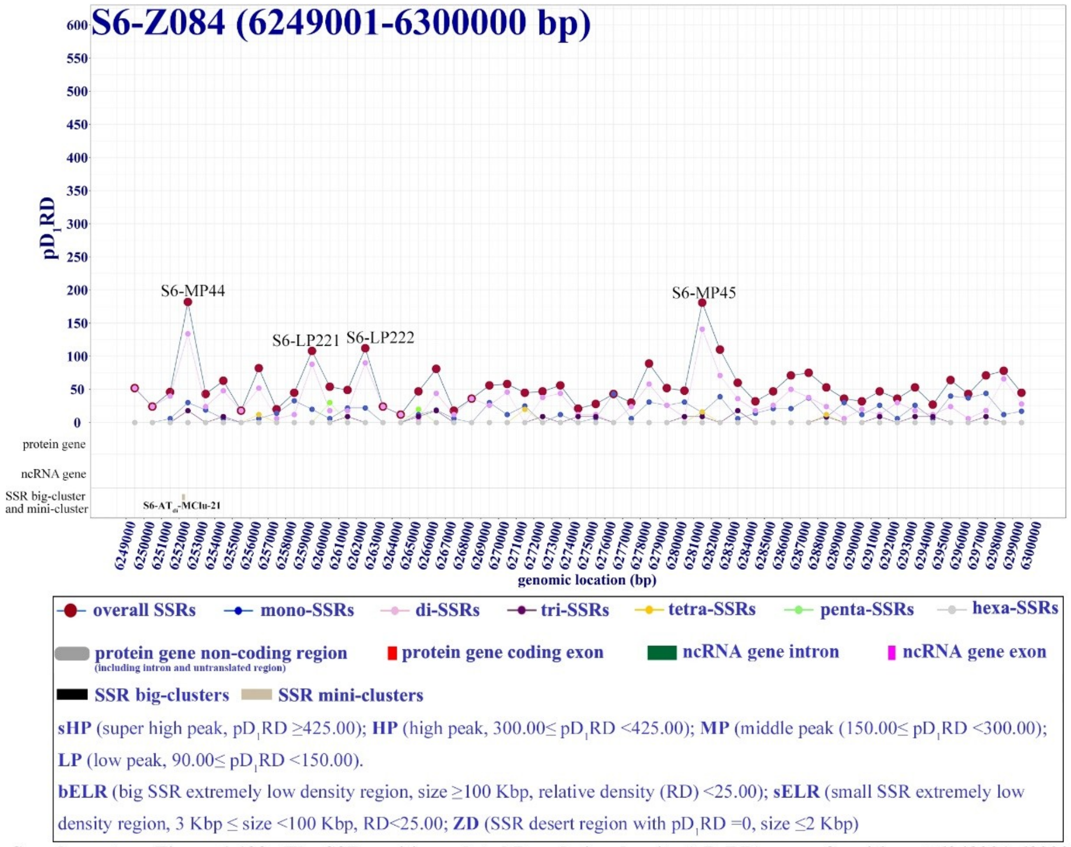

Supplementary Figure 1.122. The SSR position related D_1-relative density (pD_1RD) map of position at 6249001-6300000 bp of human reference Y-DNA (NC_000024.10) at resolution of 1 Kbp.

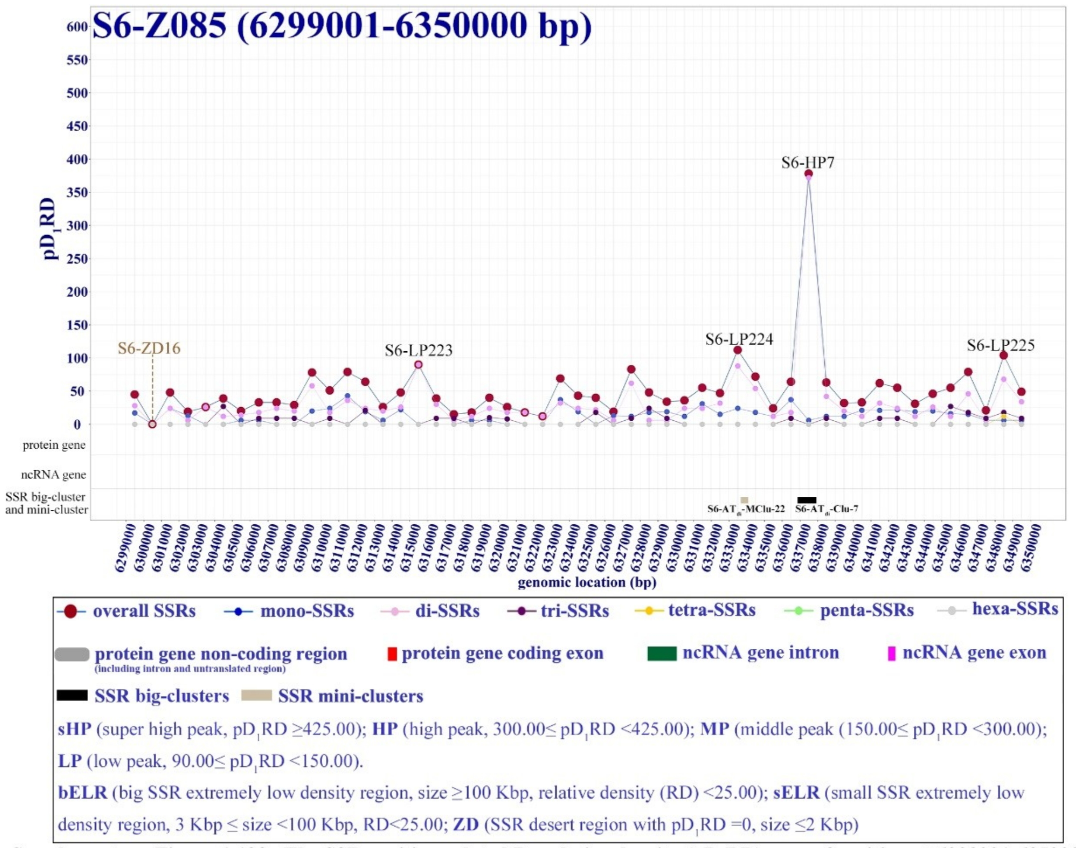

Supplementary Figure 1.123. The SSR position related D_1-relative density (pD_1RD) map of position at 6299001-6350000 bp of human reference Y-DNA (NC_000024.10) at resolution of 1 Kbp.

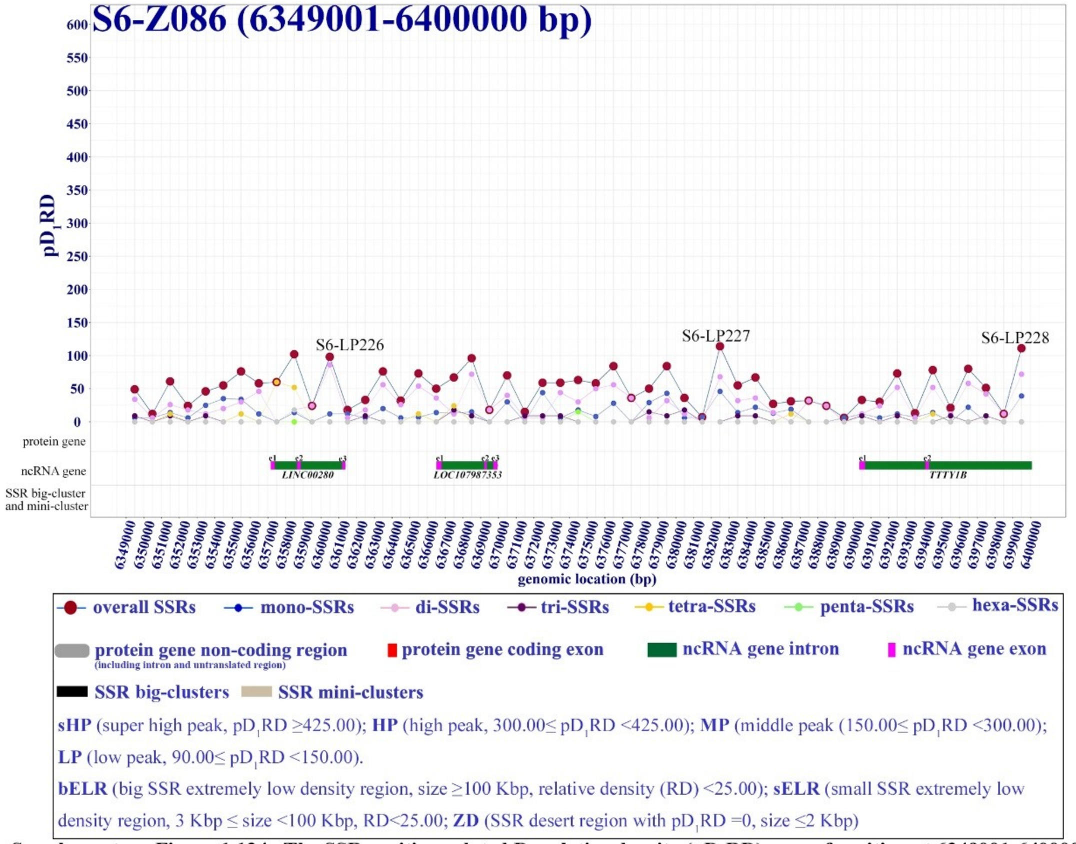

Supplementary Figure 1.124. The SSR position related D_1-relative density (pD_1RD) map of position at 6349001-6400000 bp of human reference Y-DNA (NC_000024.10) at resolution of 1 Kbp.

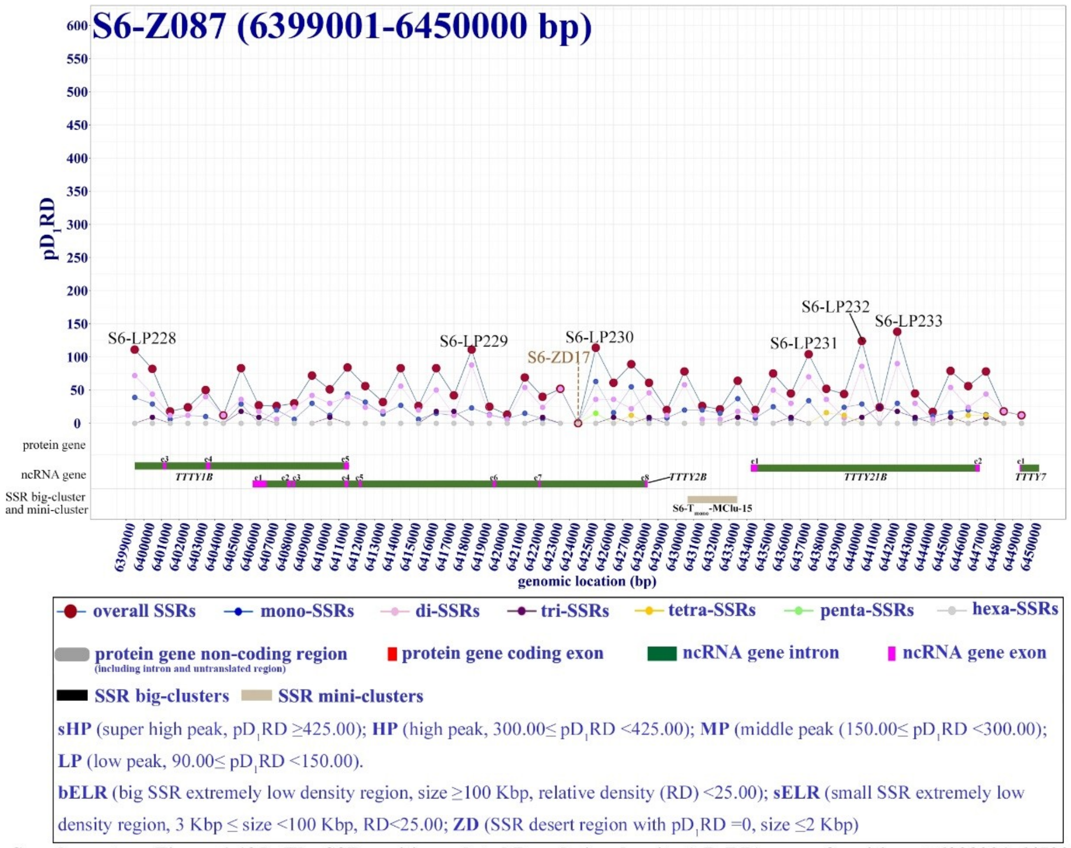

Supplementary Figure 1.125. The SSR position related D_1-relative density (pD_1RD) map of position at 6399001-6450000 bp of human reference Y-DNA (NC_000024.10) at resolution of 1 Kbp.

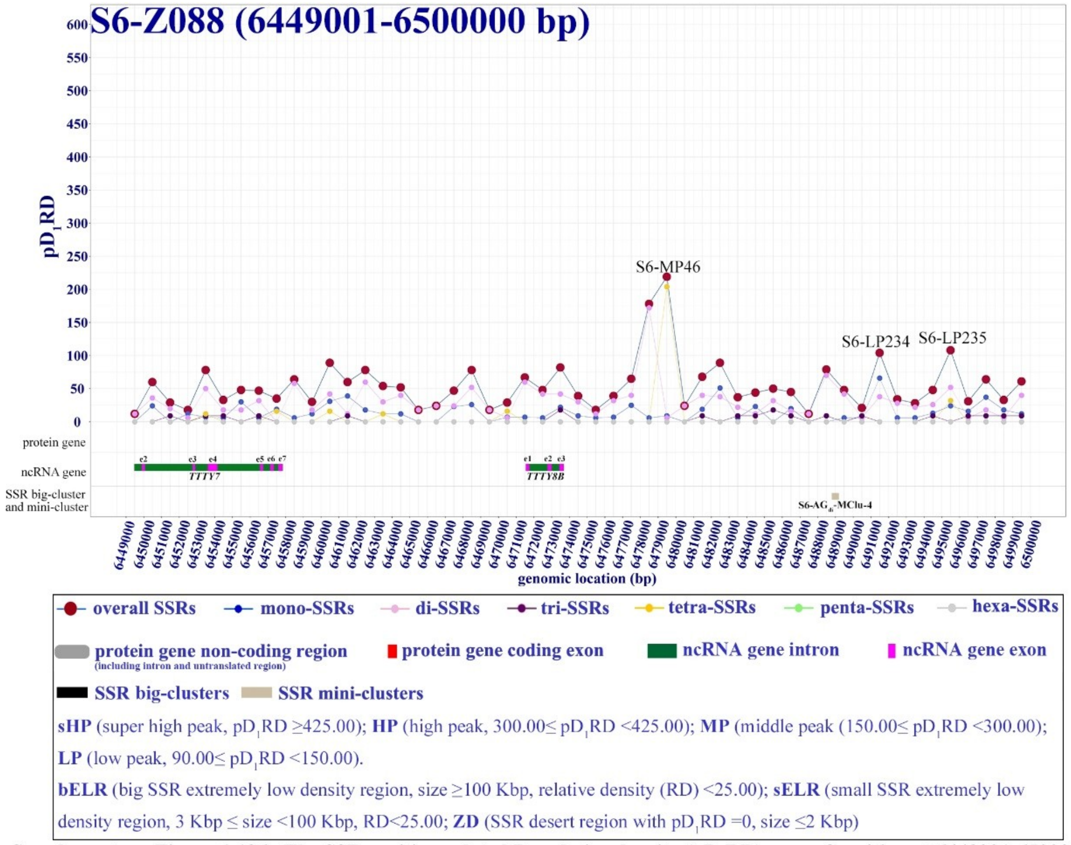

Supplementary Figure 1.126. The SSR position related D_1-relative density (pD_1RD) map of position at 6449001-6500000 bp of human reference Y-DNA (NC_000024.10) at resolution of 1 Kbp.

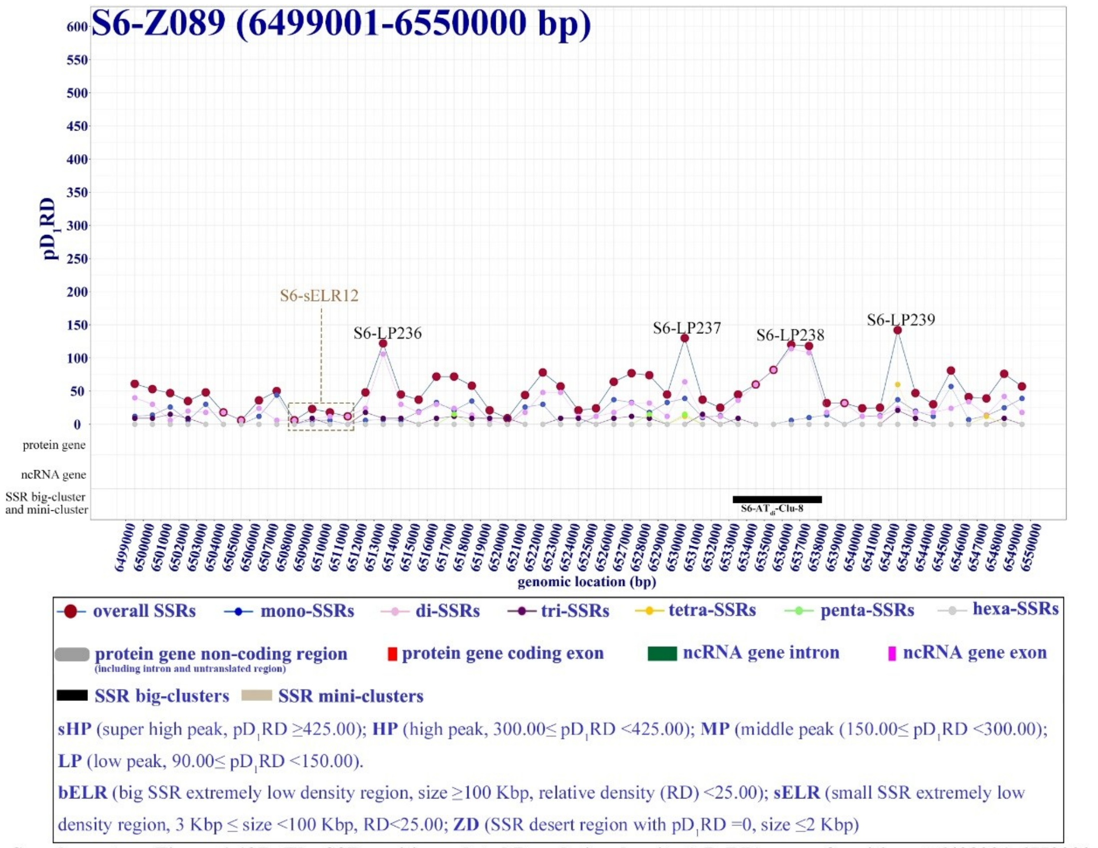

Supplementary Figure 1.127. The SSR position related D_1-relative density (pD_1RD) map of position at 6499001-6550000 bp of human reference Y-DNA (NC_000024.10) at resolution of 1 Kbp.

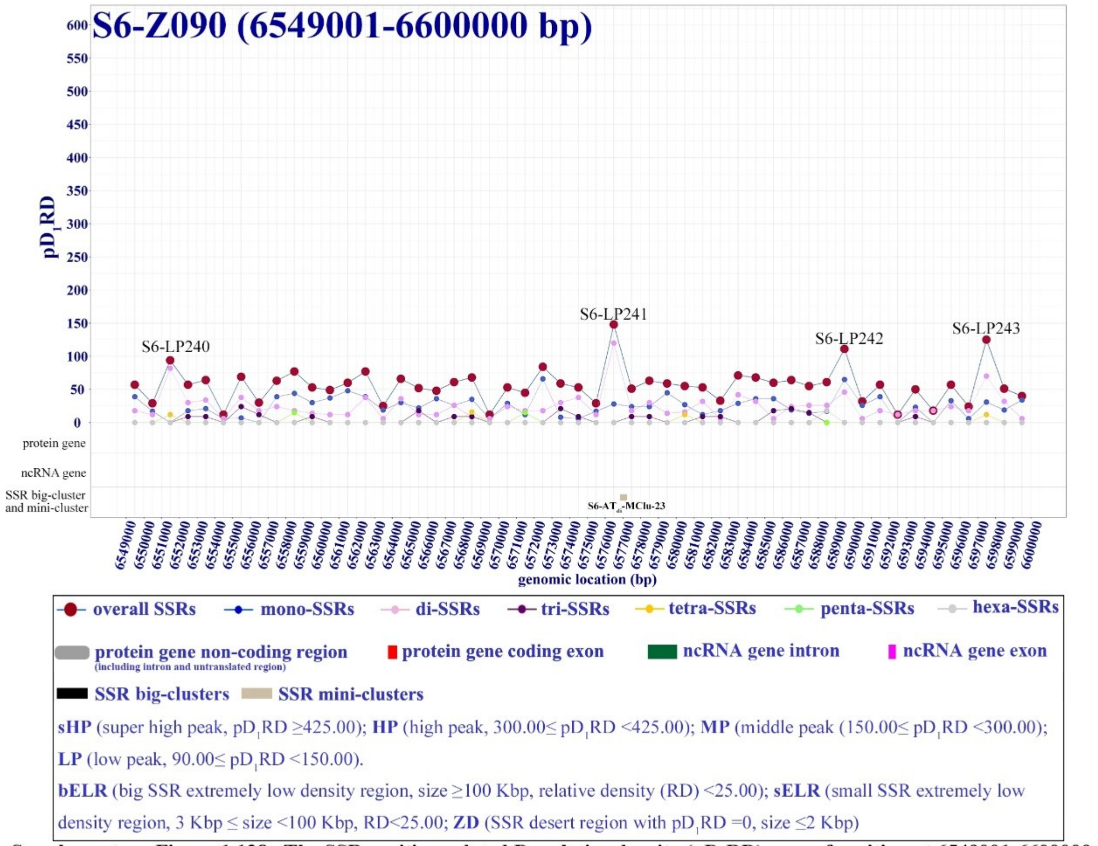

Supplementary Figure 1.128. The SSR position related D_1-relative density (pD_1RD) map of position at 6549001-6600000 bp of human reference Y-DNA (NC_000024.10) at resolution of 1 Kbp.

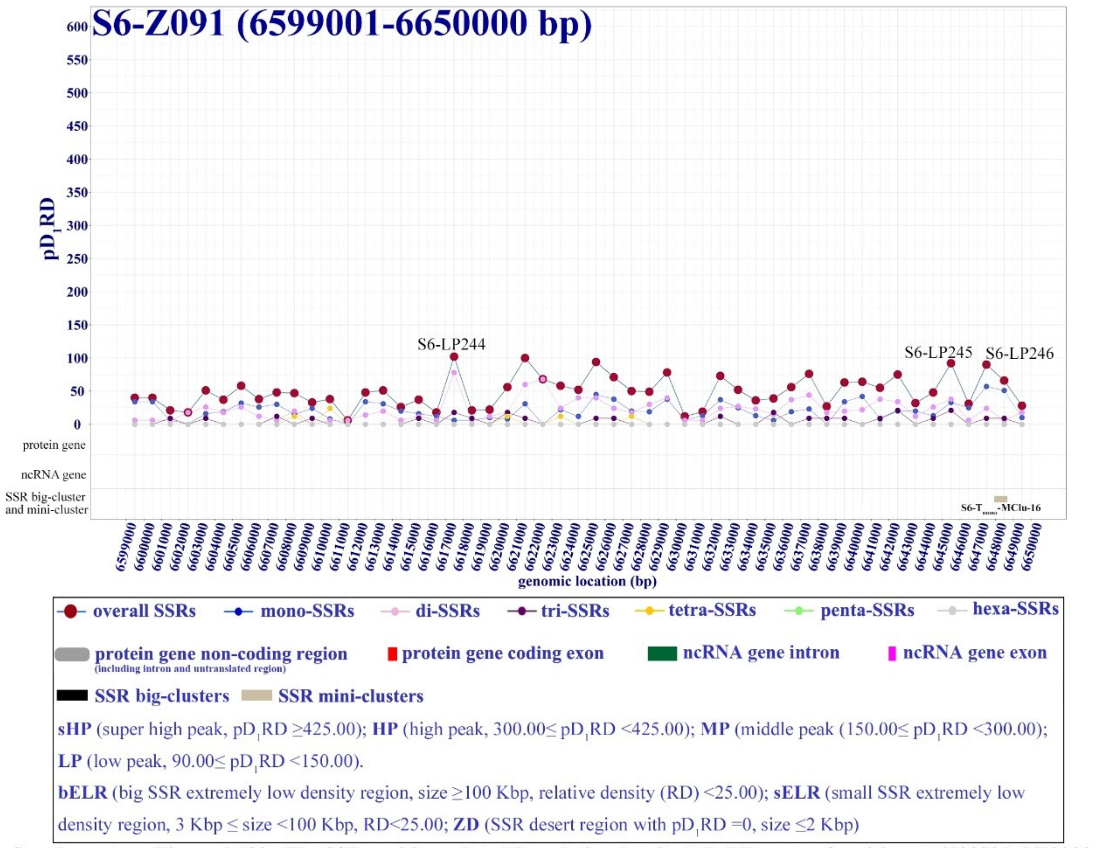

Supplementary Figure 1.129. The SSR position related D_1-relative density (pD_1RD) map of position at 6599001-6650000 bp of human reference Y-DNA (NC_000024.10) at resolution of 1 Kbp.

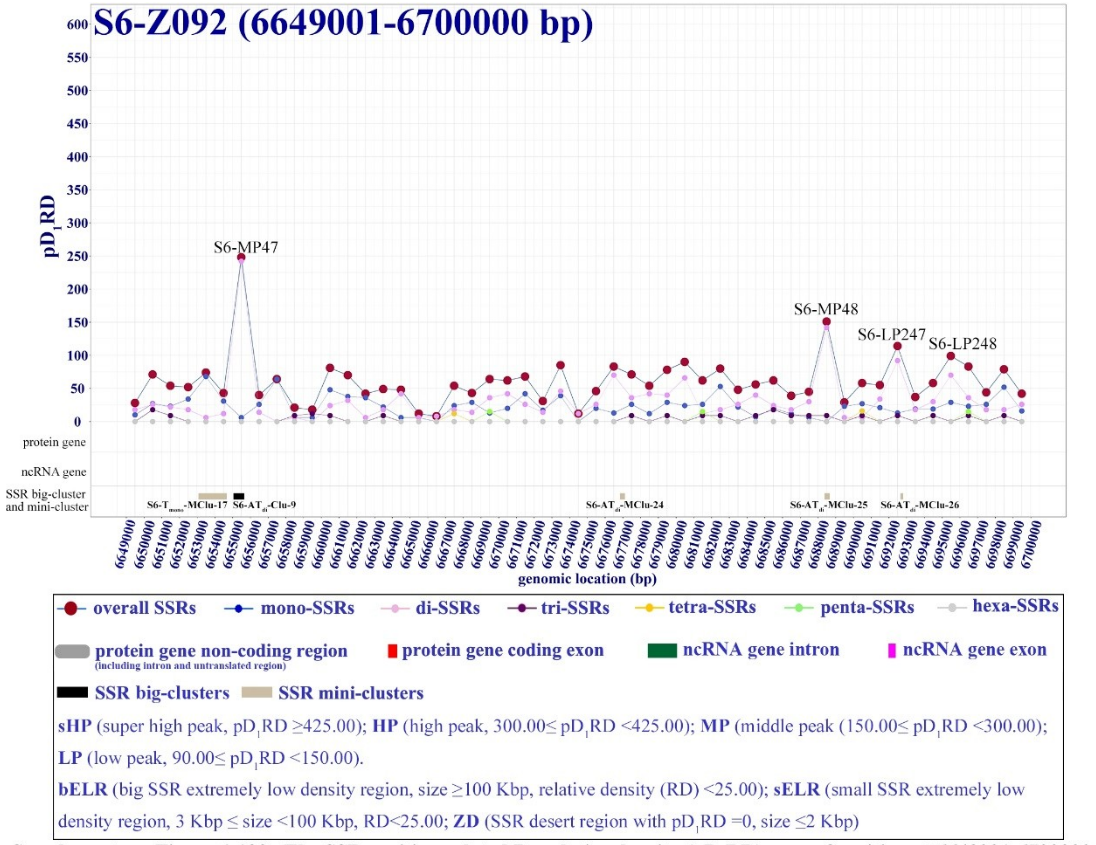

Supplementary Figure 1.130. The SSR position related D_1-relative density (pD_1RD) map of position at 6649001-6700000 bp of human reference Y-DNA (NC_000024.10) at resolution of 1 Kbp.

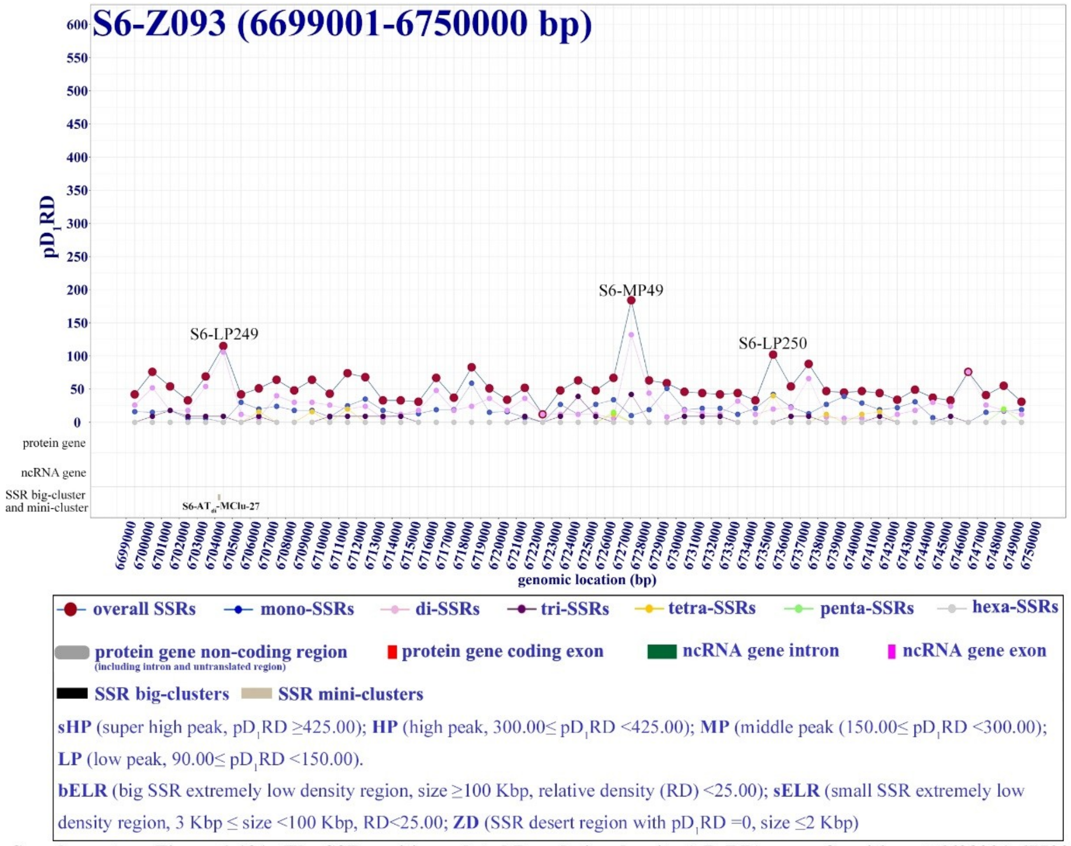

Supplementary Figure 1.131. The SSR position related D_1-relative density (pD_1RD) map of position at 6699001-6750000 bp of human reference Y-DNA (NC_000024.10) at resolution of 1 Kbp.

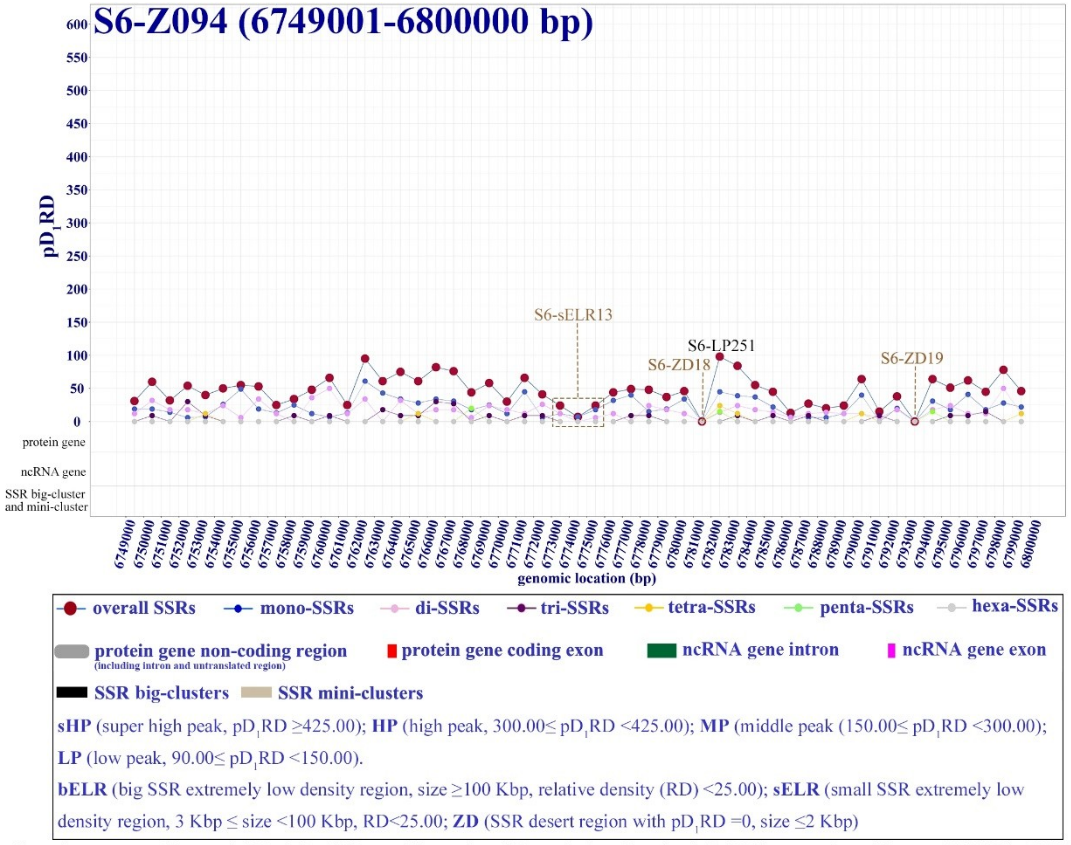

Supplementary Figure 1.132. The SSR position related D_1-relative density (pD_1RD) map of position at 6749001-6800000 bp of human reference Y-DNA (NC_000024.10) at resolution of 1 Kbp.

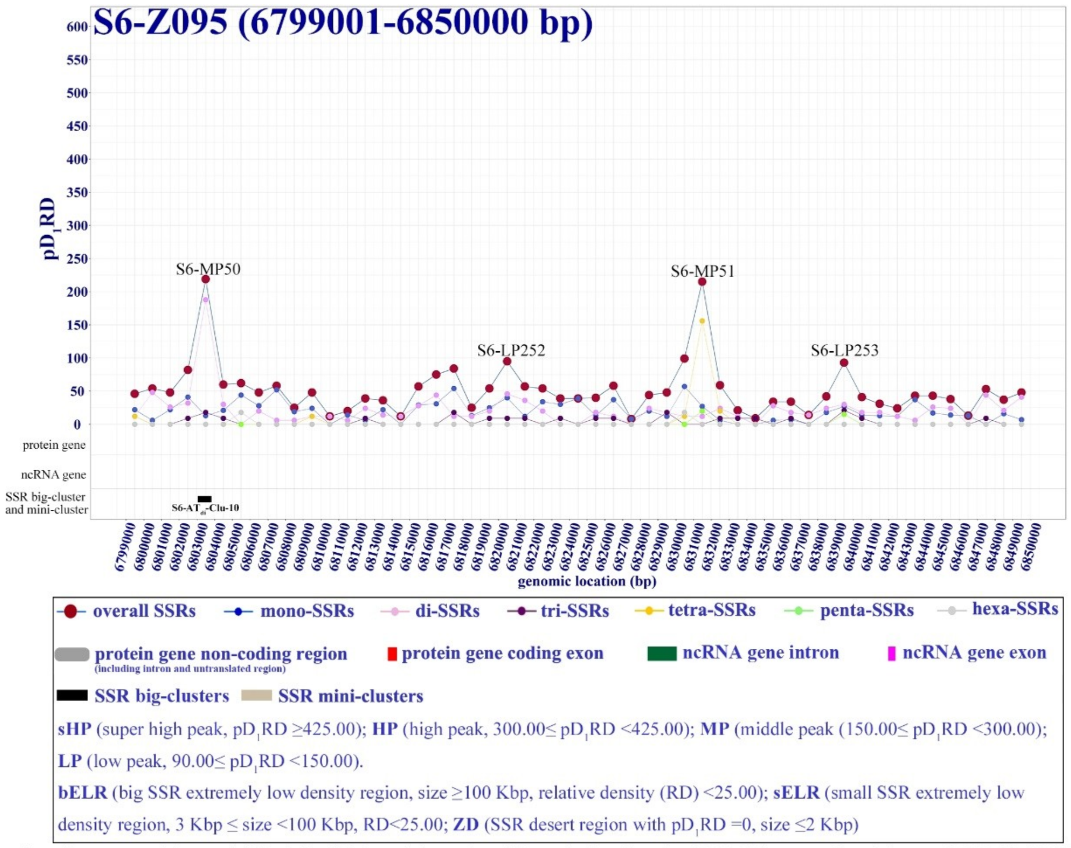

Supplementary Figure 1.133. The SSR position related D_1-relative density (pD_1RD) map of position at 6799001-6850000 bp of human reference Y-DNA (NC_000024.10) at resolution of 1 Kbp.

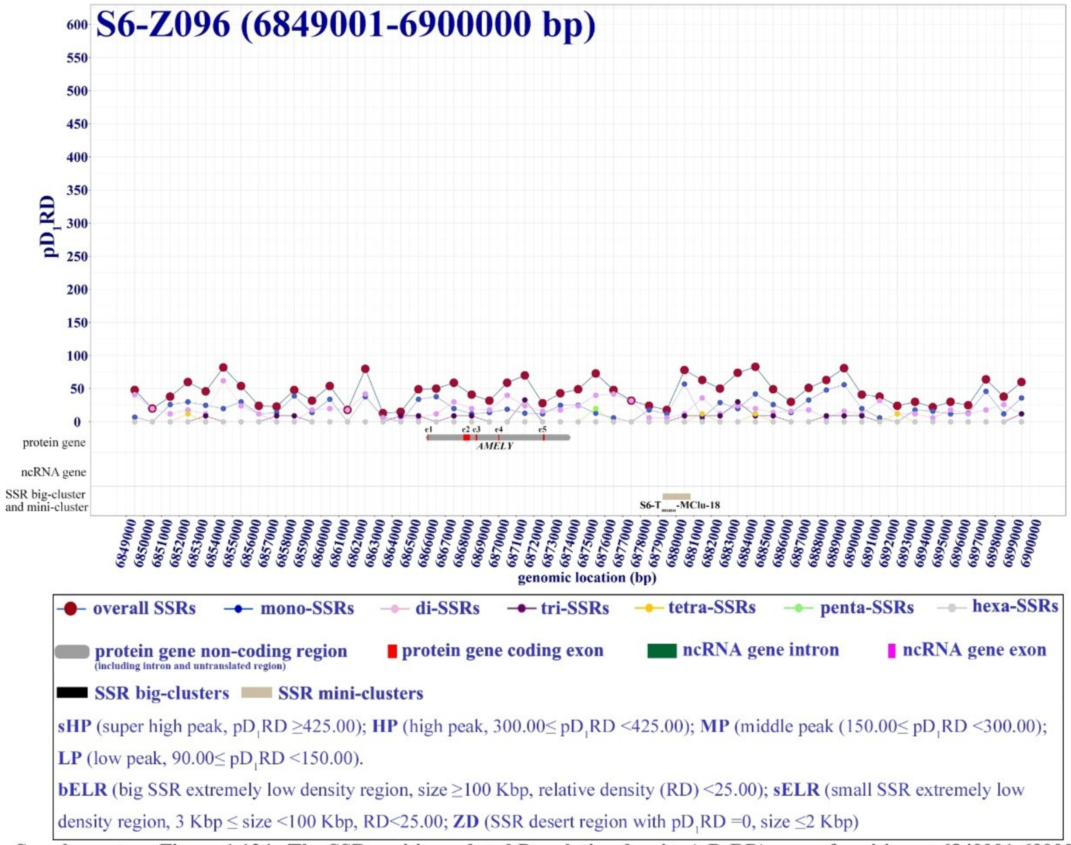

Supplementary Figure 1.134. The SSR position related D_1-relative density (pD_1RD) map of position at 6849001-6900000 bp of human reference Y-DNA (NC_000024.10) at resolution of 1 Kbp.

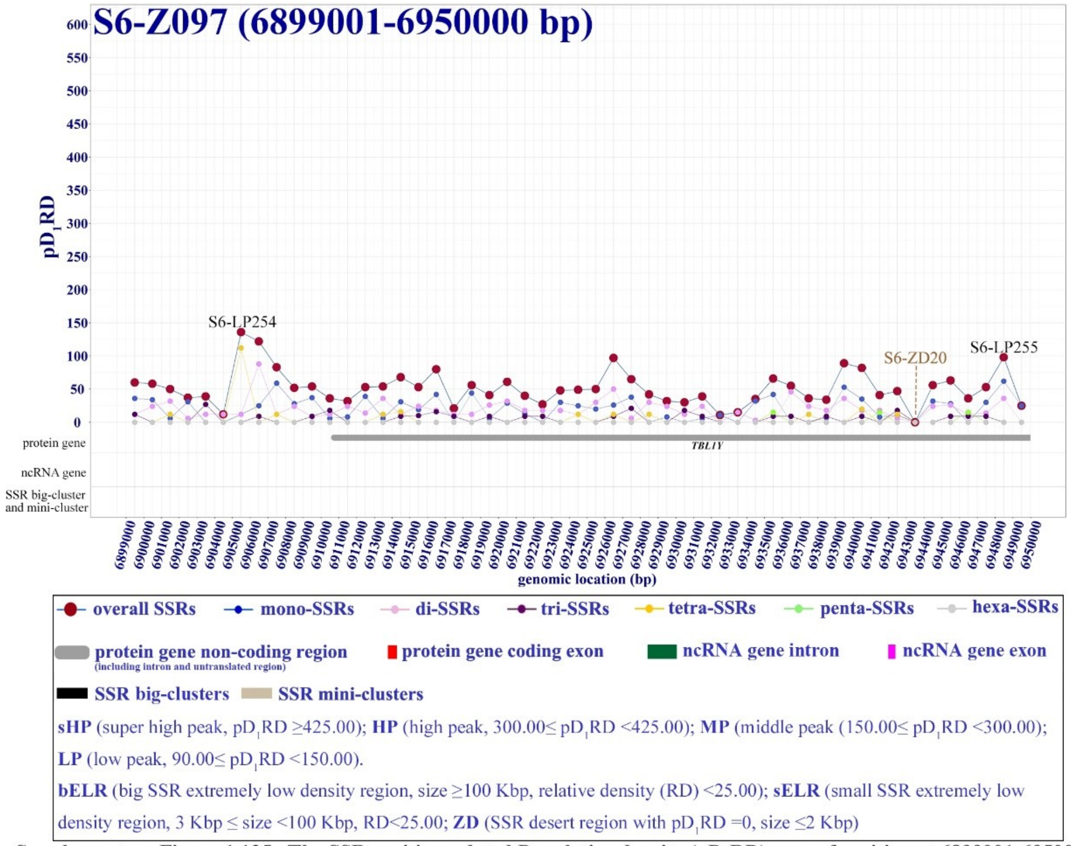

Supplementary Figure 1.135. The SSR position related D_1-relative density (pD_1RD) map of position at 6899001-6950000 bp of human reference Y-DNA (NC_000024.10) at resolution of 1 Kbp.

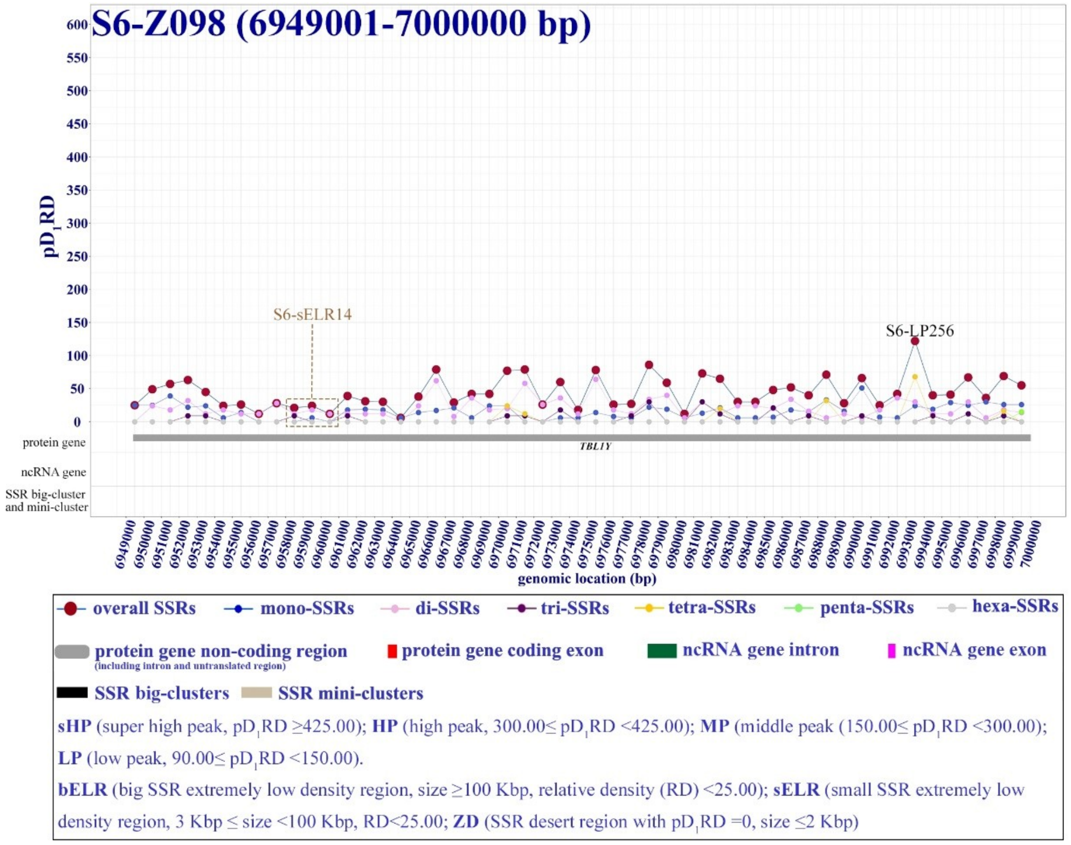

Supplementary Figure 1.136. The SSR position related D_1-relative density (pD_1RD) map of position at 6949001-7000000 bp of human reference Y-DNA (NC_000024.10) at resolution of 1 Kbp.

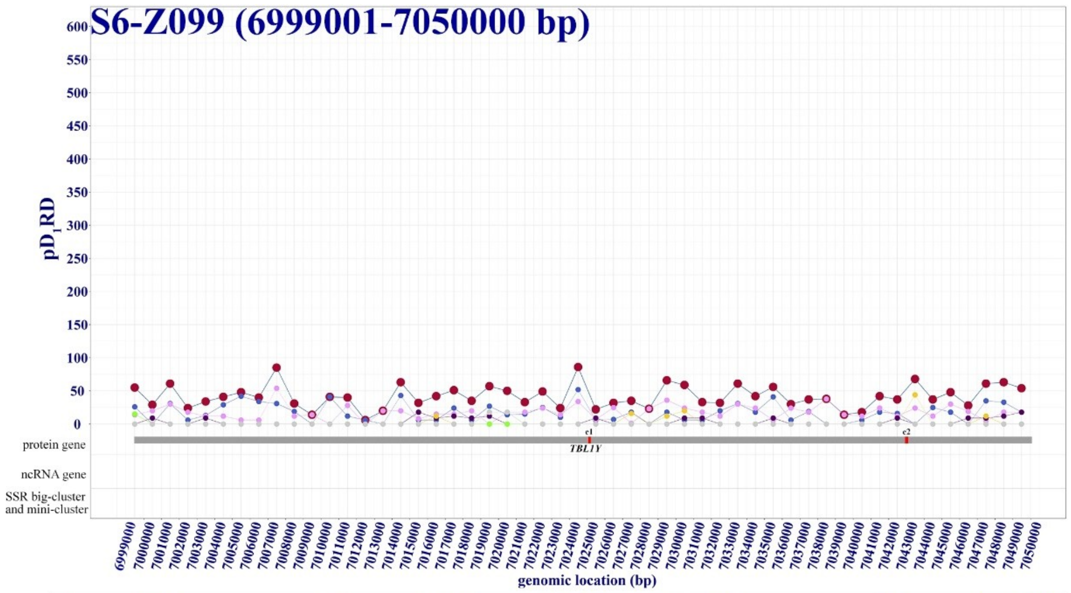

overall SSRs | mono-SSRs | di-SSRs | tri-SSRs | tetra-SSRs | penta-SSRs | hexa-SSRs

protein gene non-coding region (including intron and untranslated region) | protein gene coding exon | ncRNA gene intron | ncRNA gene exon

SSR big-clusters | SSR mini-clusters

sHP (super high peak, $pD_1RD \geq 425.00$); **HP** (high peak, $300.00 \leq pD_1RD < 425.00$); **MP** (middle peak ($150.00 \leq pD_1RD < 300.00$); **LP** (low peak, $90.00 \leq pD_1RD < 150.00$).

bELR (big SSR extremely low density region, size ≥100 Kbp, relative density (RD) <25.00); **sELR** (small SSR extremely low density region, 3 Kbp ≤ size <100 Kbp, RD<25.00; **ZD** (SSR desert region with $pD_1RD = 0$, size ≤2 Kbp)

Supplementary Figure 1.137. The SSR position related D_1-relative density (pD_1RD) map of position at 6999001-7050000 bp of human reference Y-DNA (NC_000024.10) at resolution of 1 Kbp.

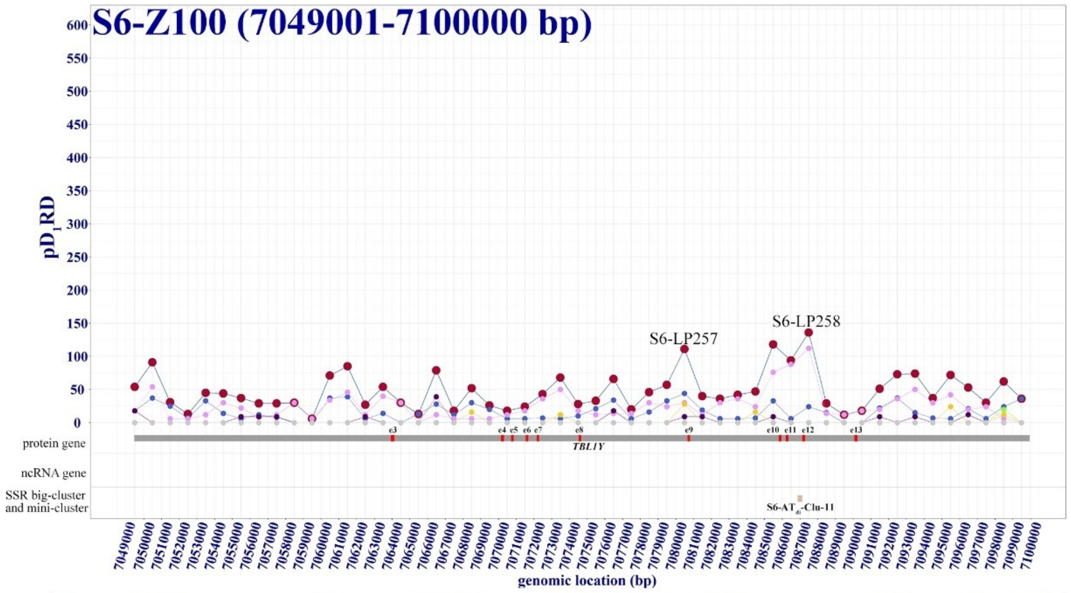

overall SSRs | mono-SSRs | di-SSRs | tri-SSRs | tetra-SSRs | penta-SSRs | hexa-SSRs

protein gene non-coding region (including intron and untranslated region) | protein gene coding exon | ncRNA gene intron | ncRNA gene exon

SSR big-clusters | SSR mini-clusters

sHP (super high peak, $pD_1RD \geq 425.00$); **HP** (high peak, $300.00 \leq pD_1RD < 425.00$); **MP** (middle peak ($150.00 \leq pD_1RD < 300.00$); **LP** (low peak, $90.00 \leq pD_1RD < 150.00$).

bELR (big SSR extremely low density region, size ≥100 Kbp, relative density (RD) <25.00); **sELR** (small SSR extremely low density region, 3 Kbp ≤ size <100 Kbp, RD<25.00; **ZD** (SSR desert region with $pD_1RD = 0$, size ≤2 Kbp)

Supplementary Figure 1.138. The SSR position related D_1-relative density (pD_1RD) map of position at 7049001-7100000 bp of human reference Y-DNA (NC_000024.10) at resolution of 1 Kbp.

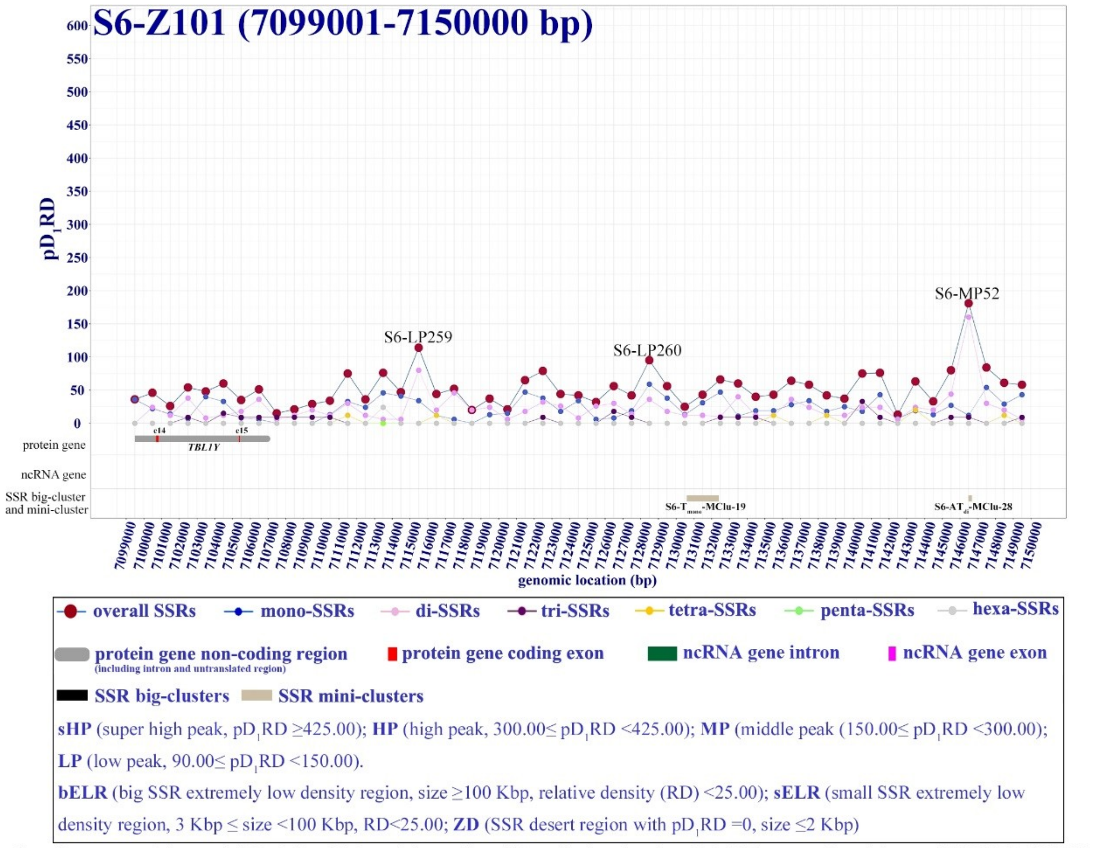

Supplementary Figure 1.139. The SSR position related D_1-relative density (pD_1RD) map of position at 7099001-7150000 bp of human reference Y-DNA (NC_000024.10) at resolution of 1 Kbp.

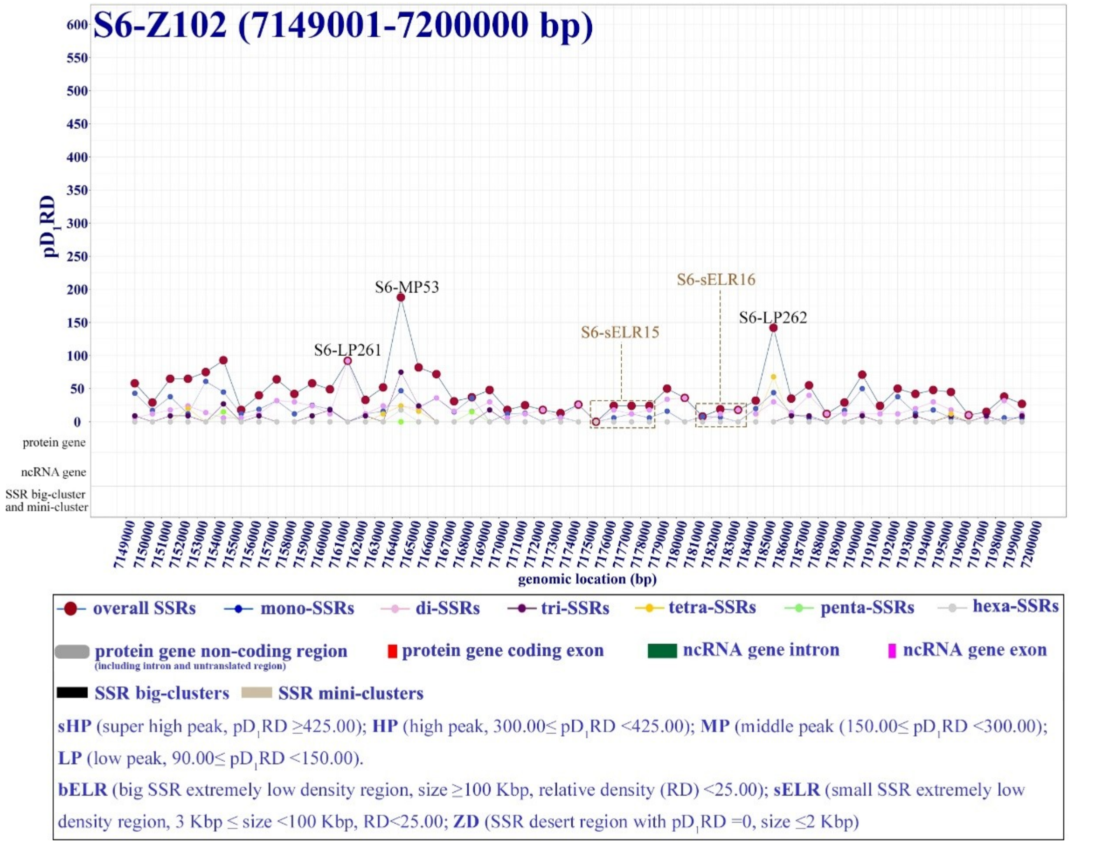

Supplementary Figure 1.140. The SSR position related D_1-relative density (pD_1RD) map of position at 7149001-7200000 bp of human reference Y-DNA (NC_000024.10) at resolution of 1 Kbp.

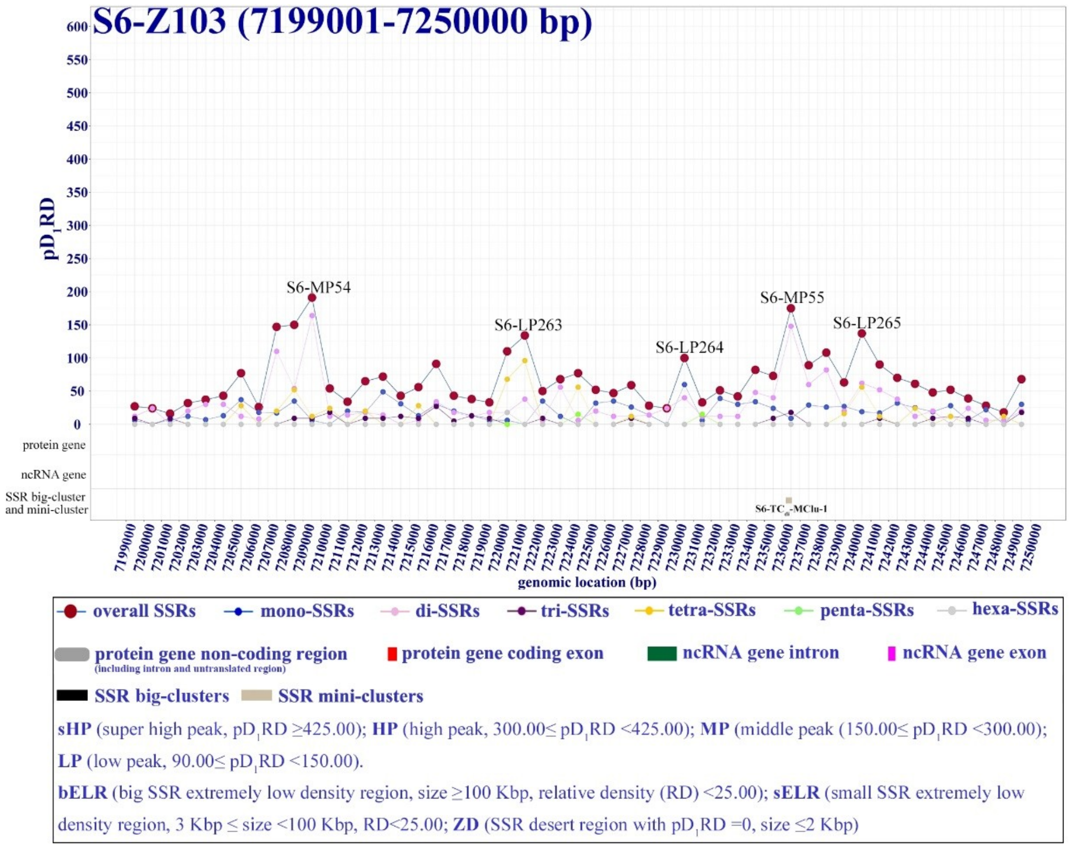

Supplementary Figure 1.141. The SSR position related D_1-relative density (pD_1RD) map of position at 7199001-7250000 bp of human reference Y-DNA (NC_000024.10) at resolution of 1 Kbp.

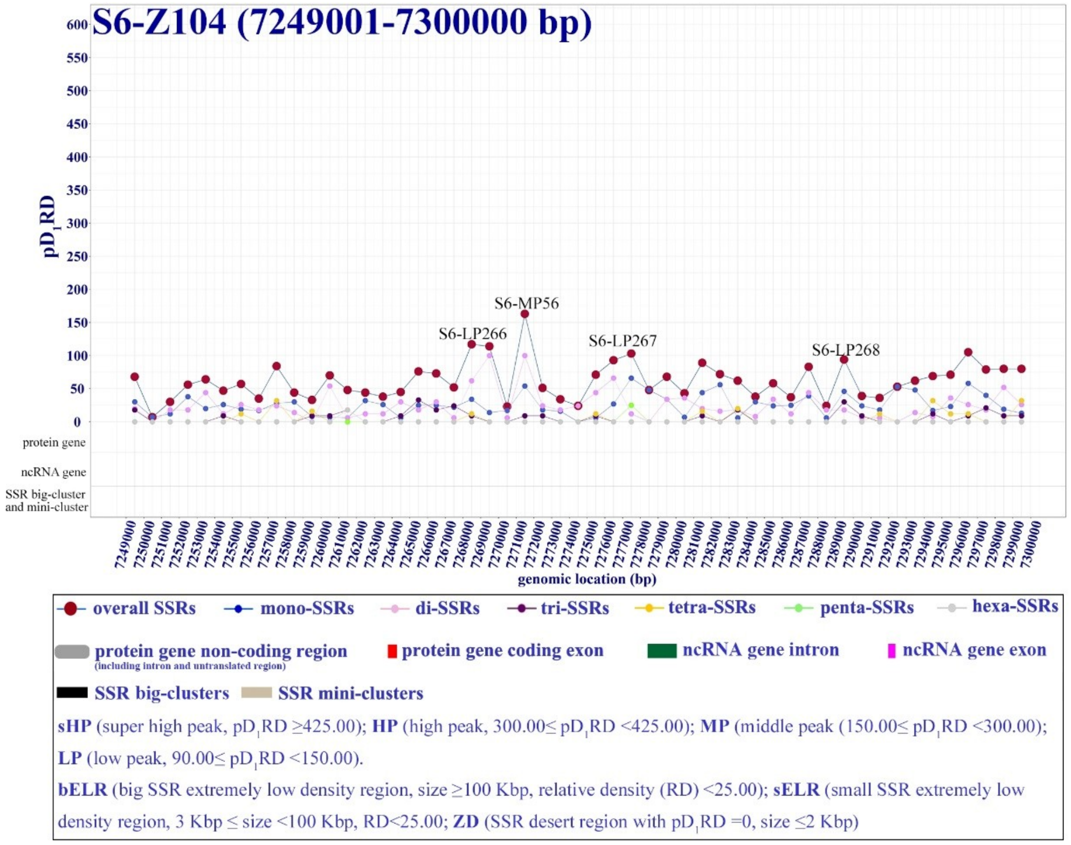

Supplementary Figure 1.142. The SSR position related D_1-relative density (pD_1RD) map of position at 7249001-7300000 bp of human reference Y-DNA (NC_000024.10) at resolution of 1 Kbp.

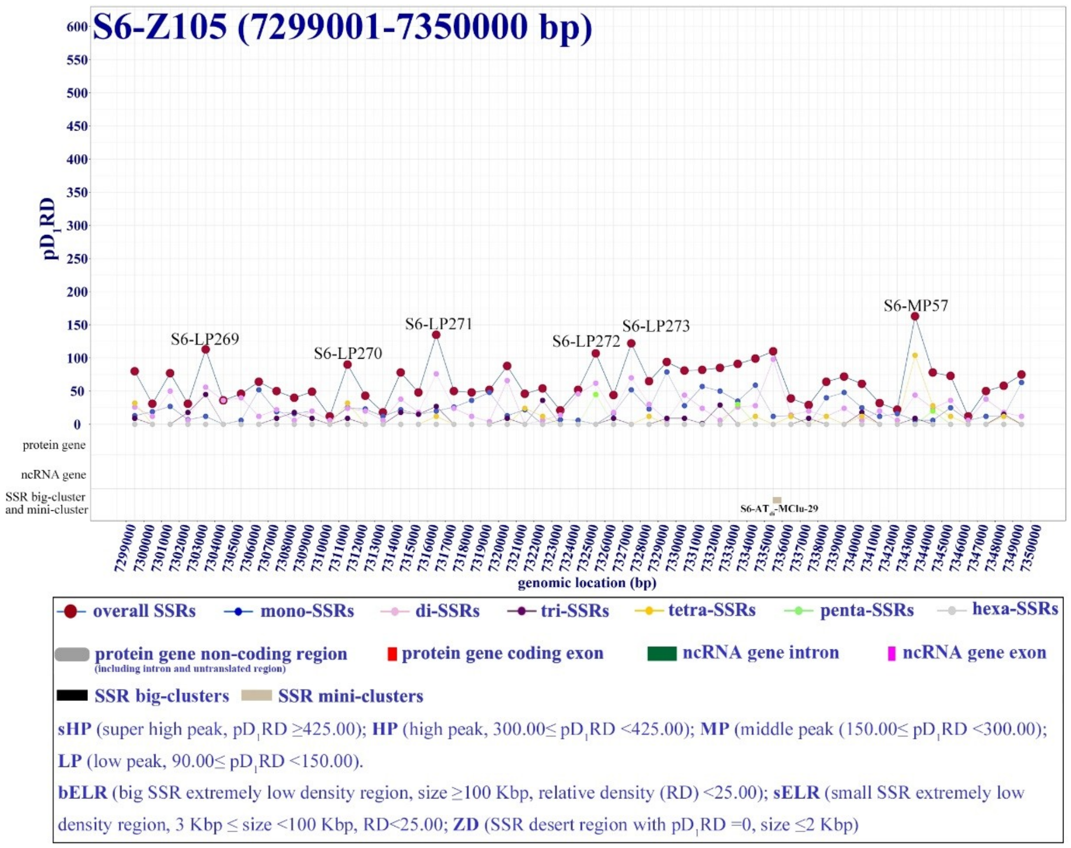

Supplementary Figure 1.143. The SSR position related D_1-relative density (pD_1RD) map of position at 7299001-7350000 bp of human reference Y-DNA (NC_000024.10) at resolution of 1 Kbp.

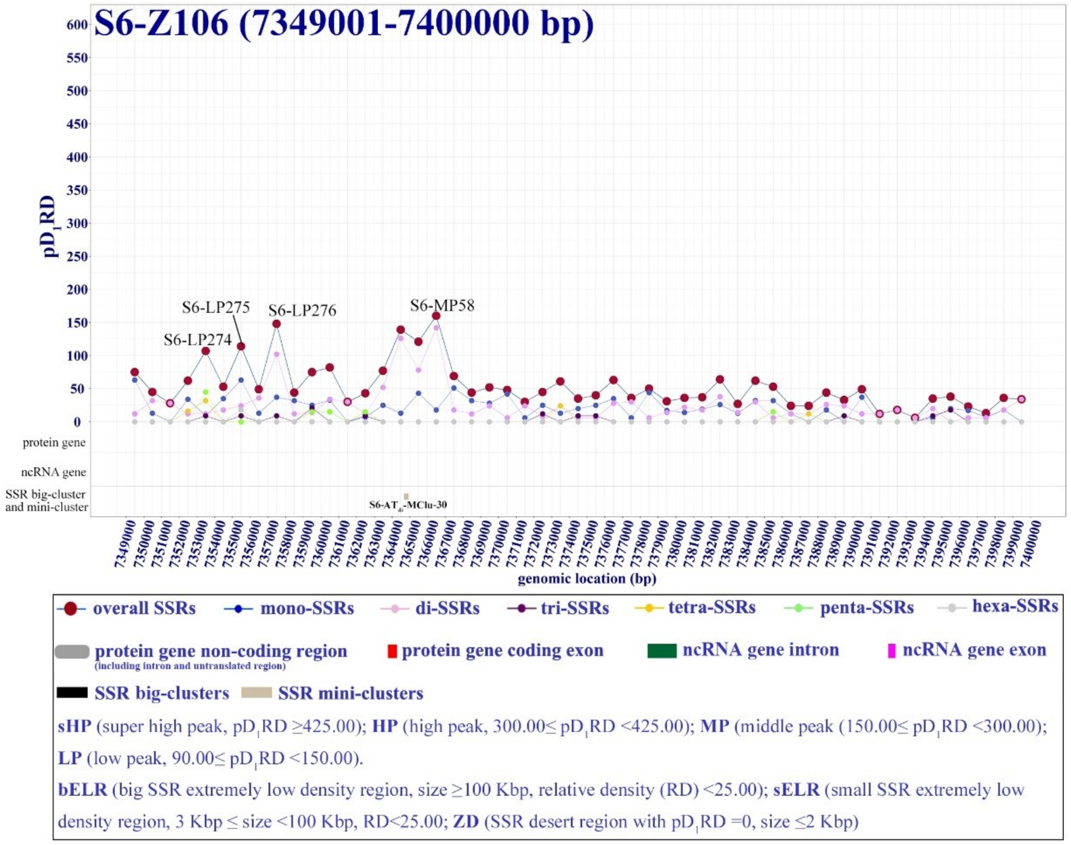

Supplementary Figure 1.144. The SSR position related D_1-relative density (pD_1RD) map of position at 7349001-7400000 bp of human reference Y-DNA (NC_000024.10) at resolution of 1 Kbp.

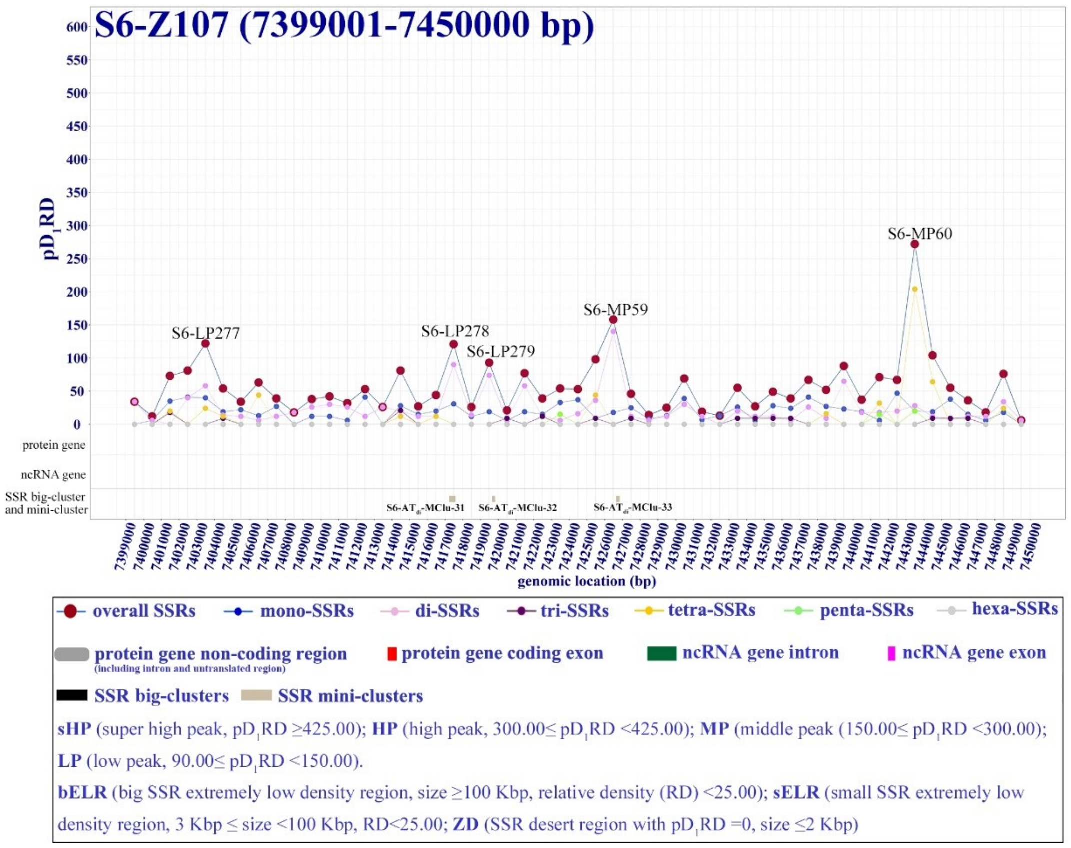

Supplementary Figure 1.145. The SSR position related D_1-relative density (pD_1RD) map of position at 7399001-7450000 bp of human reference Y-DNA (NC_000024.10) at resolution of 1 Kbp.

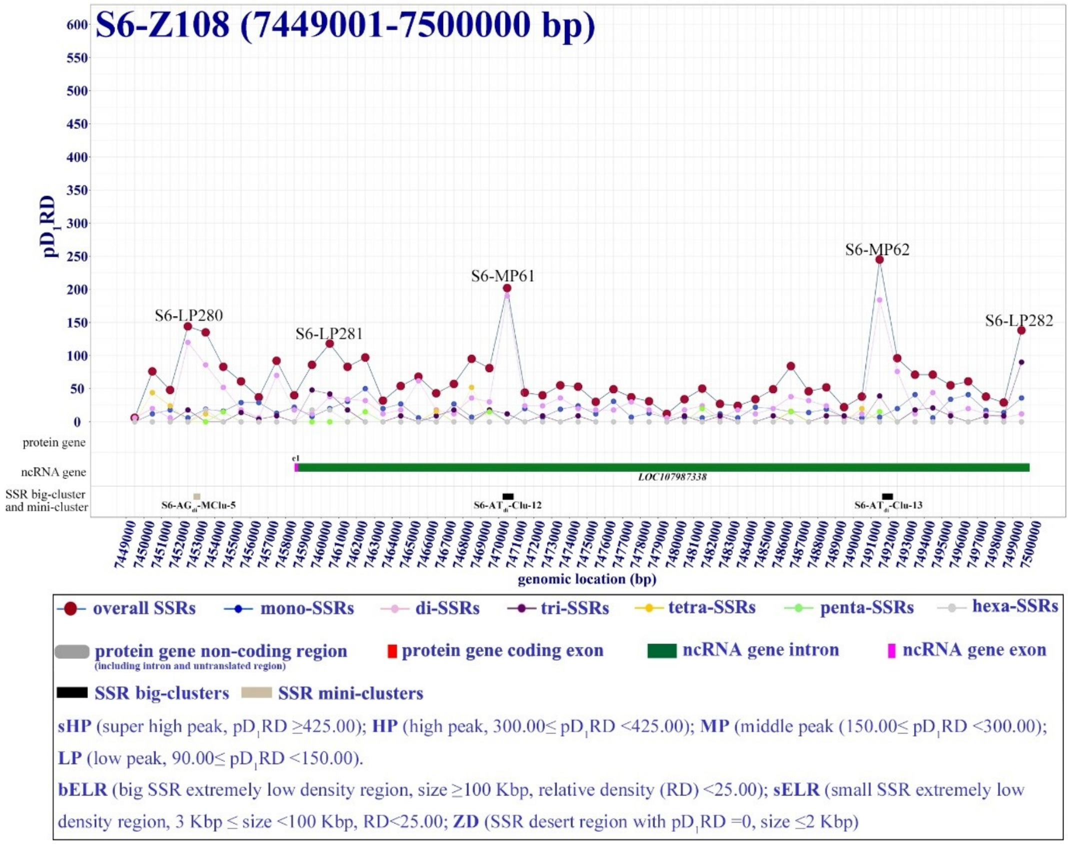

Supplementary Figure 1.146. The SSR position related D_1-relative density (pD_1RD) map of position at 7449001-7500000 bp of human reference Y-DNA (NC_000024.10) at resolution of 1 Kbp.

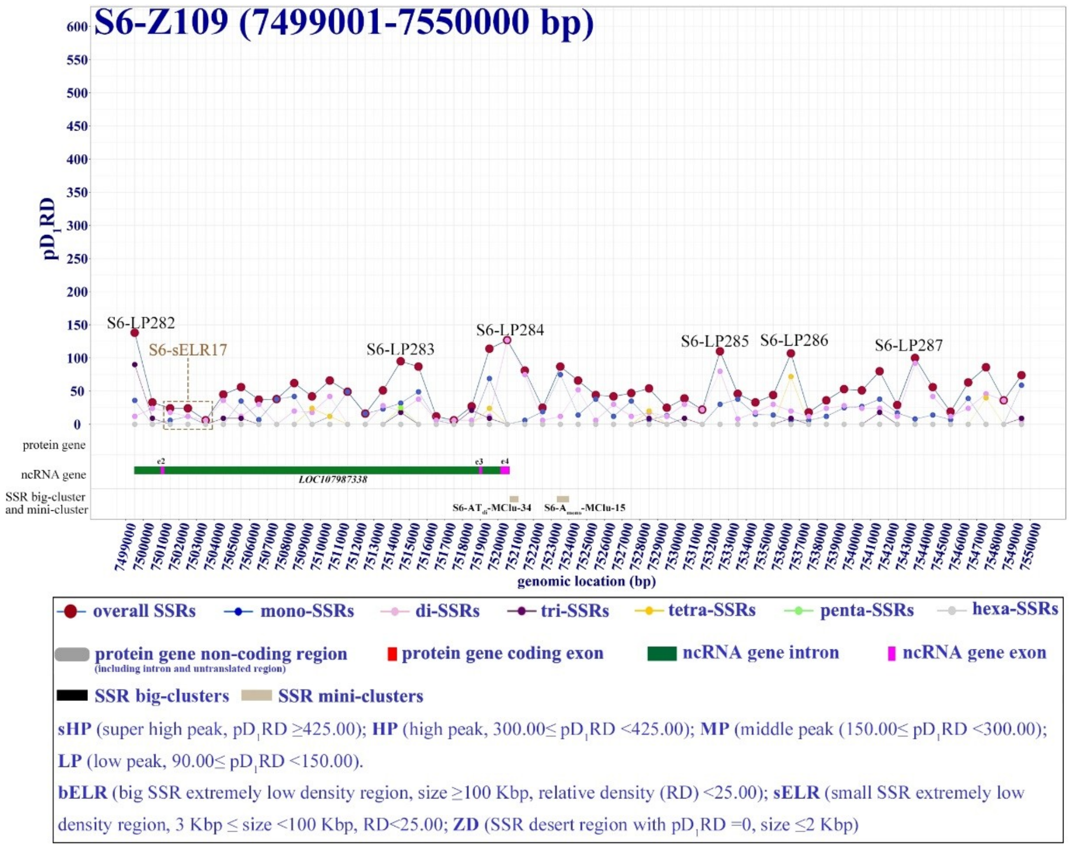

Supplementary Figure 1.147. The SSR position related D_1-relative density (pD_1RD) map of position at 7499001-7550000 bp of human reference Y-DNA (NC_000024.10) at resolution of 1 Kbp.

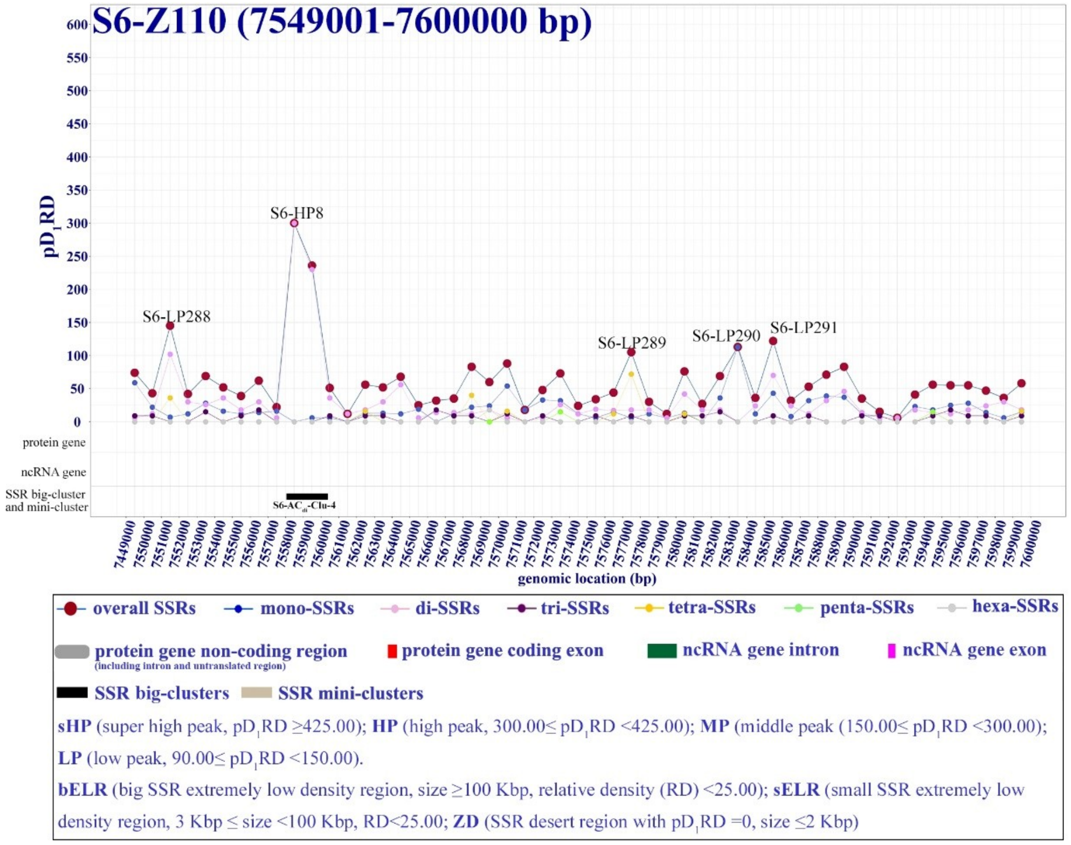

Supplementary Figure 1.148. The SSR position related D_1-relative density (pD_1RD) map of position at 7549001-7600000 bp of human reference Y-DNA (NC_000024.10) at resolution of 1 Kbp.

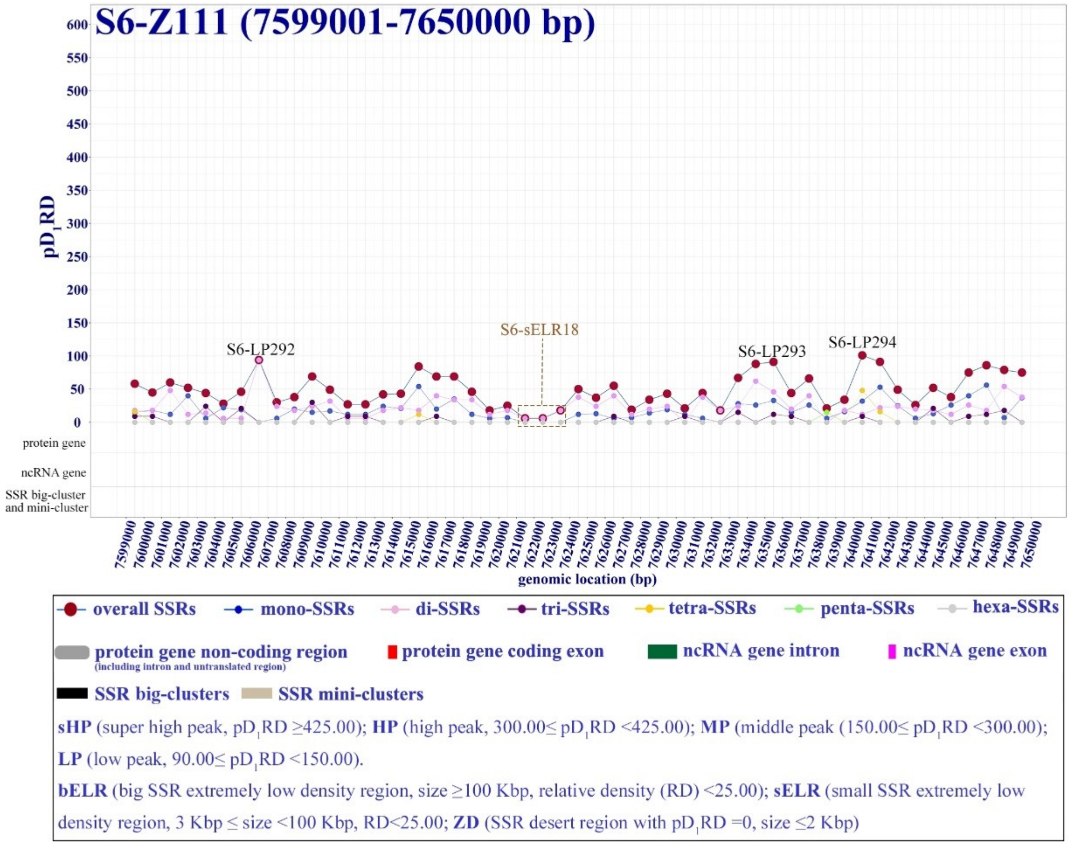

Supplementary Figure 1.149. The SSR position related D_1-relative density (pD_1RD) map of position at 7599001-7650000 bp of human reference Y-DNA (NC_000024.10) at resolution of 1 Kbp.

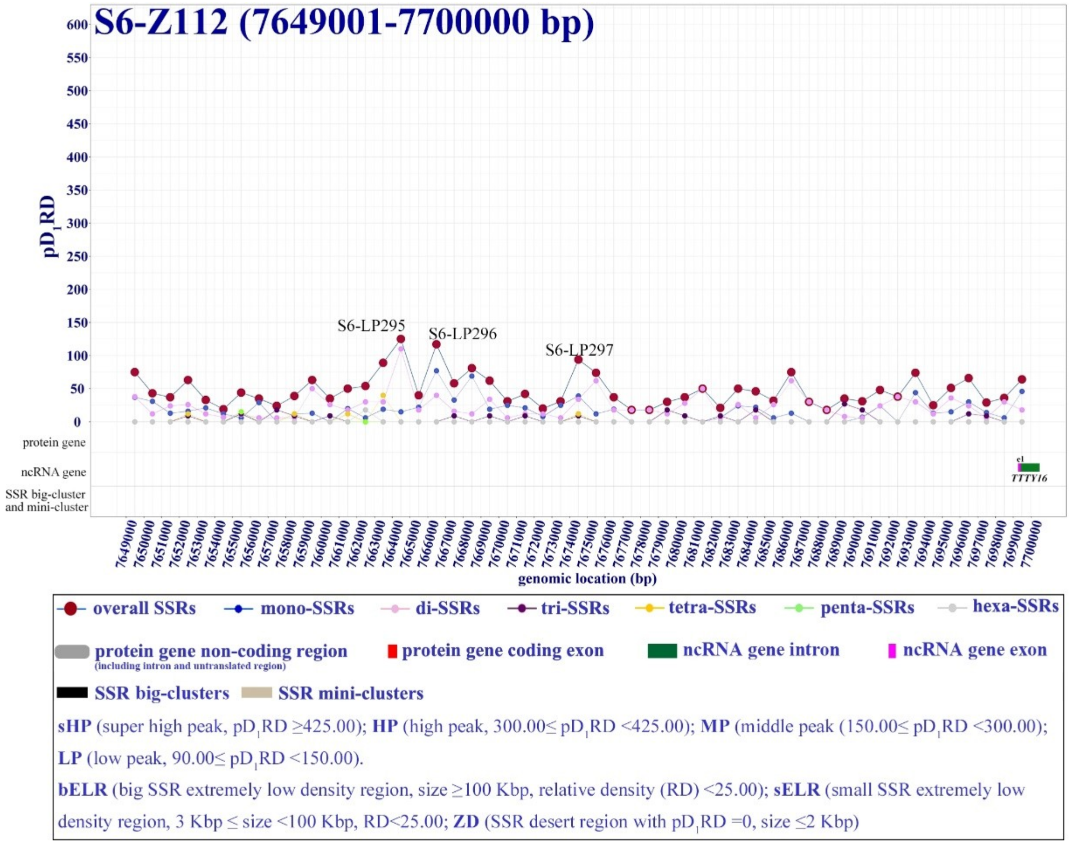

Supplementary Figure 1.150. The SSR position related D_1-relative density (pD_1RD) map of position at 7649001-7700000 bp of human reference Y-DNA (NC_000024.10) at resolution of 1 Kbp.

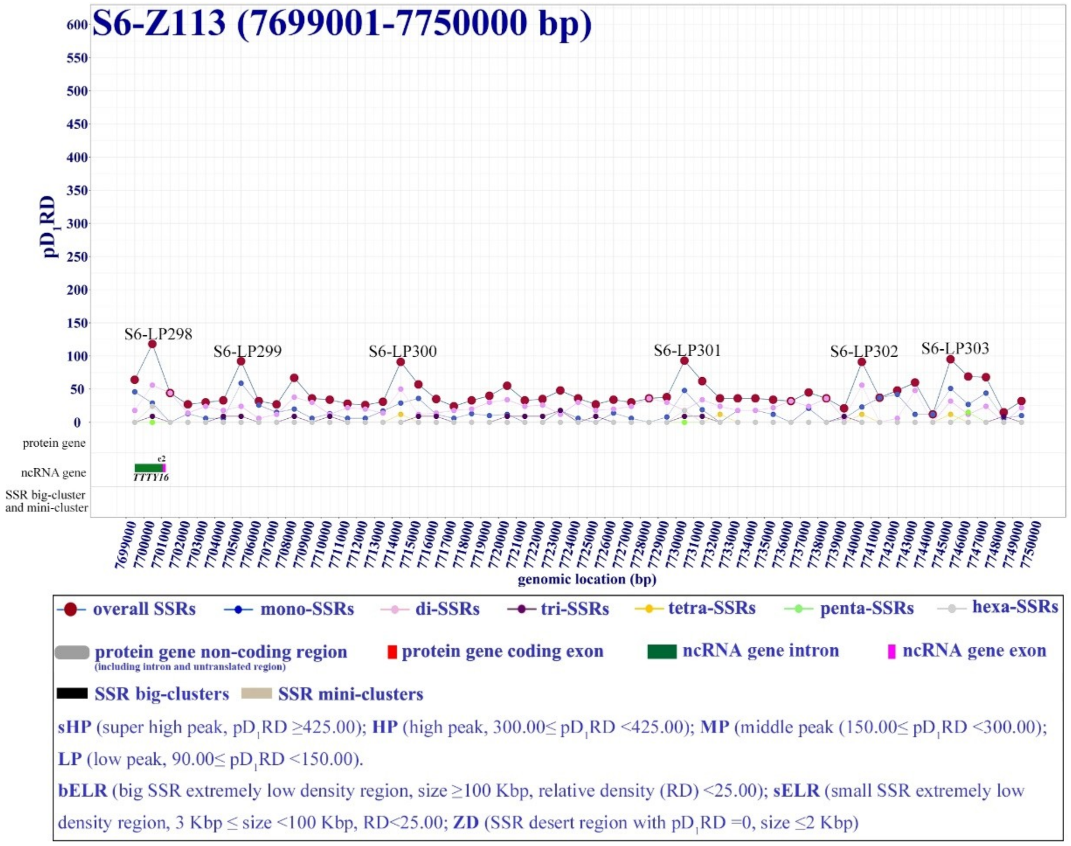

Supplementary Figure 1.151. The SSR position related D_1-relative density (pD_1RD) map of position at 7699001-7750000 bp of human reference Y-DNA (NC_000024.10) at resolution of 1 Kbp.

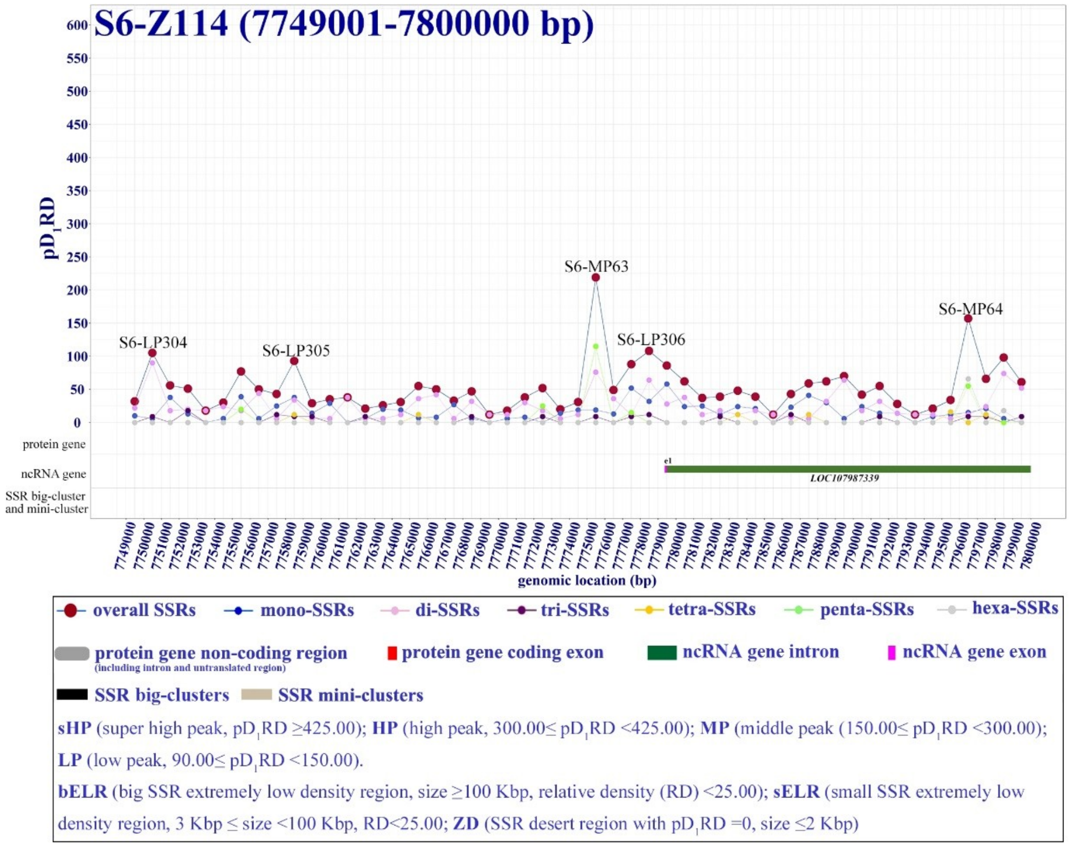

Supplementary Figure 1.152. The SSR position related D_1-relative density (pD_1RD) map of position at 7749001-7800000 bp of human reference Y-DNA (NC_000024.10) at resolution of 1 Kbp.

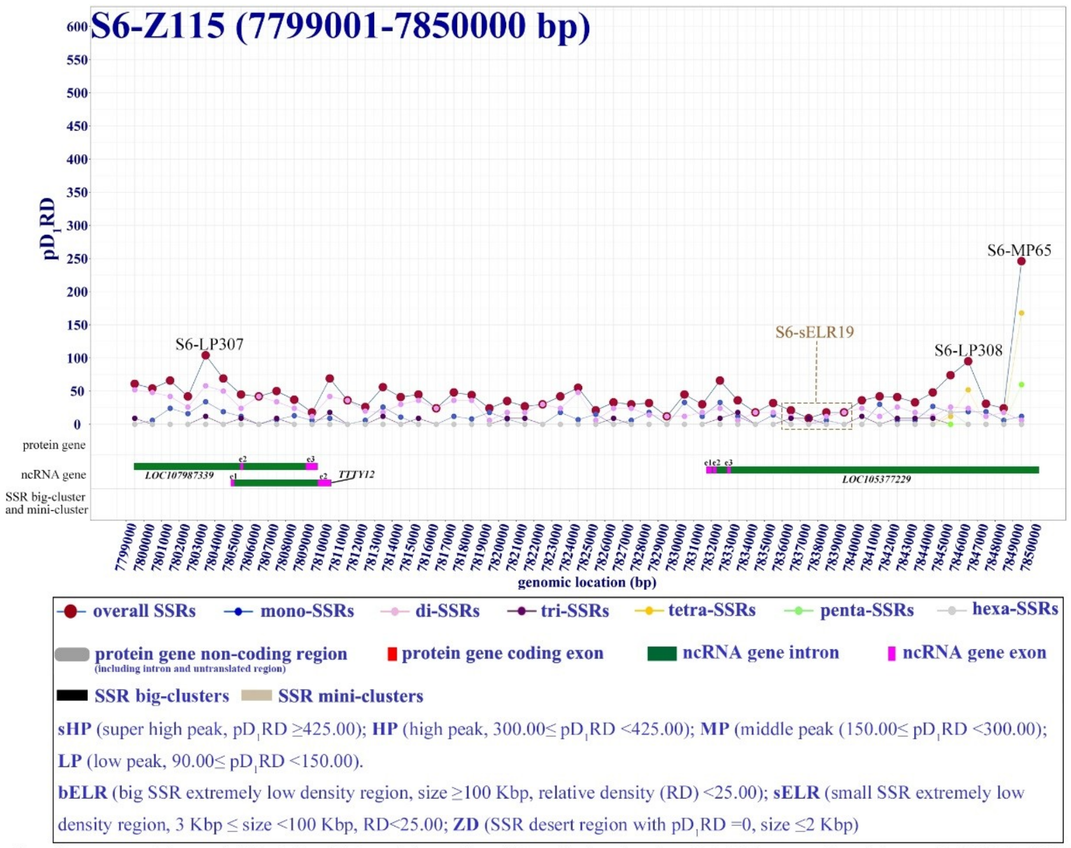

Supplementary Figure 1.153. The SSR position related D_1-relative density (pD_1RD) map of position at 7799001-7850000 bp of human reference Y-DNA (NC_000024.10) at resolution of 1 Kbp.

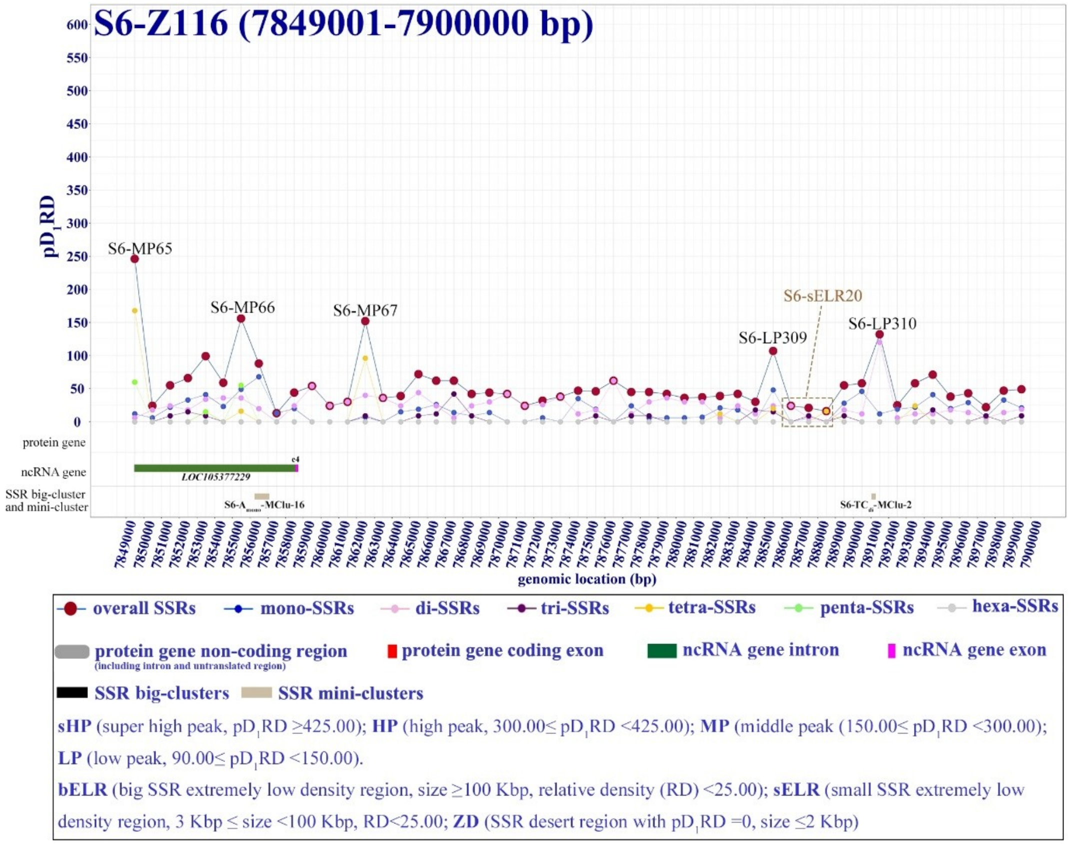

Supplementary Figure 1.154. The SSR position related D_1-relative density (pD_1RD) map of position at 7849001-7900000 bp of human reference Y-DNA (NC_000024.10) at resolution of 1 Kbp.

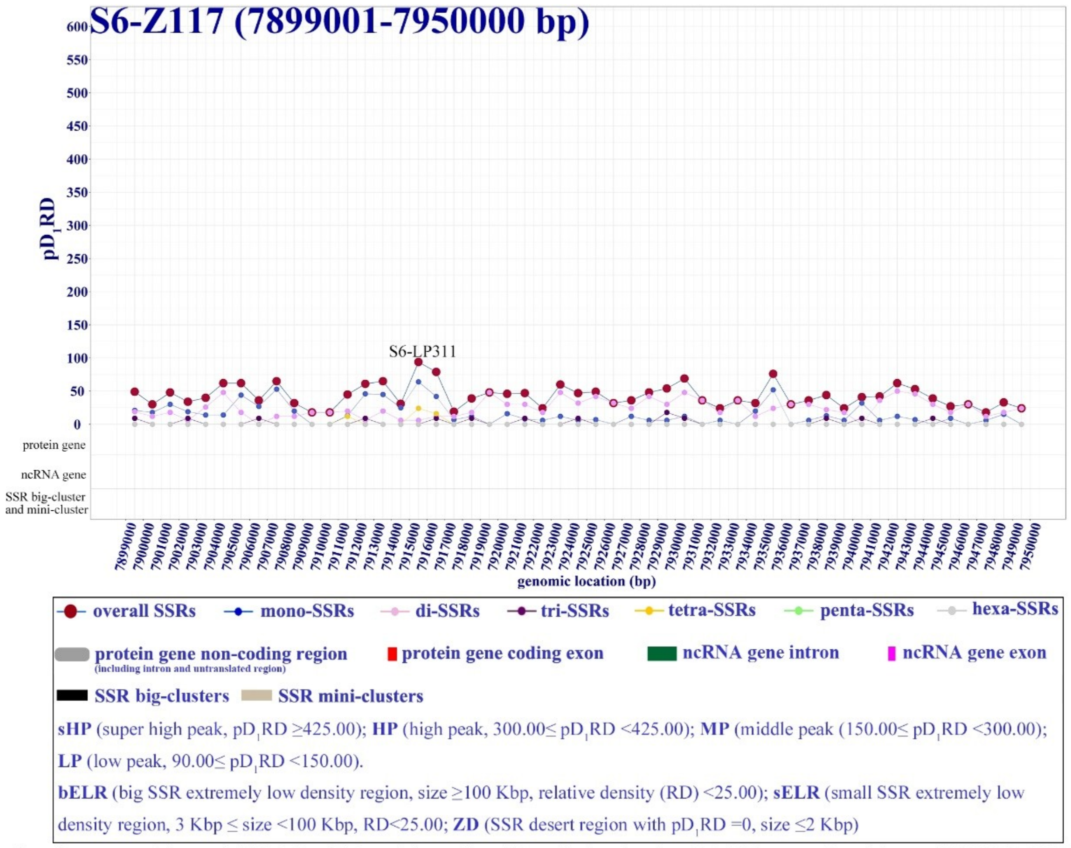

Supplementary Figure 1.155. The SSR position related D_1-relative density (pD_1RD) map of position at 7899001-7950000 bp of human reference Y-DNA (NC_000024.10) at resolution of 1 Kbp.

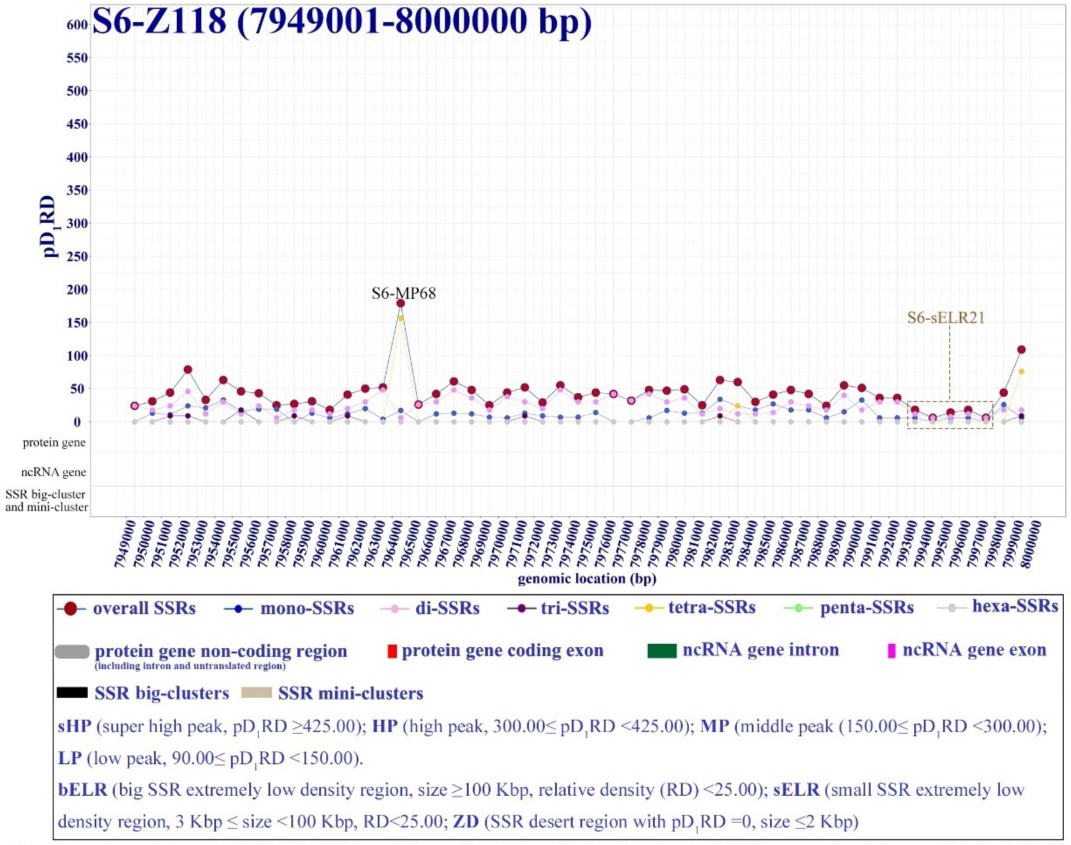

Supplementary Figure 1.156. The SSR position related D_1-relative density (pD_1RD) map of position at 7949001-8000000 bp of human reference Y-DNA (NC_000024.10) at resolution of 1 Kbp.

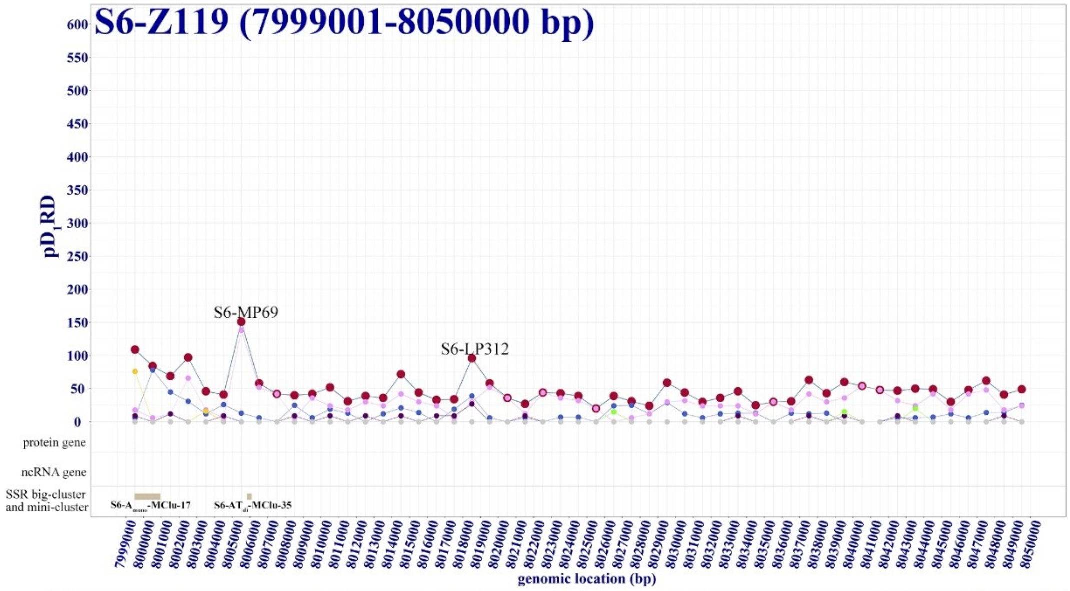

overall SSRs, mono-SSRs, di-SSRs, tri-SSRs, tetra-SSRs, penta-SSRs, hexa-SSRs

protein gene non-coding region (including intron and untranslated region), protein gene coding exon, ncRNA gene intron, ncRNA gene exon

SSR big-clusters, SSR mini-clusters

sHP (super high peak, $pD_1RD \geq 425.00$); **HP** (high peak, $300.00 \leq pD_1RD < 425.00$); **MP** (middle peak ($150.00 \leq pD_1RD < 300.00$); **LP** (low peak, $90.00 \leq pD_1RD < 150.00$).

bELR (big SSR extremely low density region, size ≥100 Kbp, relative density (RD) <25.00); **sELR** (small SSR extremely low density region, 3 Kbp ≤ size <100 Kbp, RD<25.00; **ZD** (SSR desert region with $pD_1RD = 0$, size ≤2 Kbp)

Supplementary Figure 1.157. The SSR position related D_1-relative density (pD_1RD) map of position at 7999001-8050000 bp of human reference Y-DNA (NC_000024.10) at resolution of 1 Kbp.

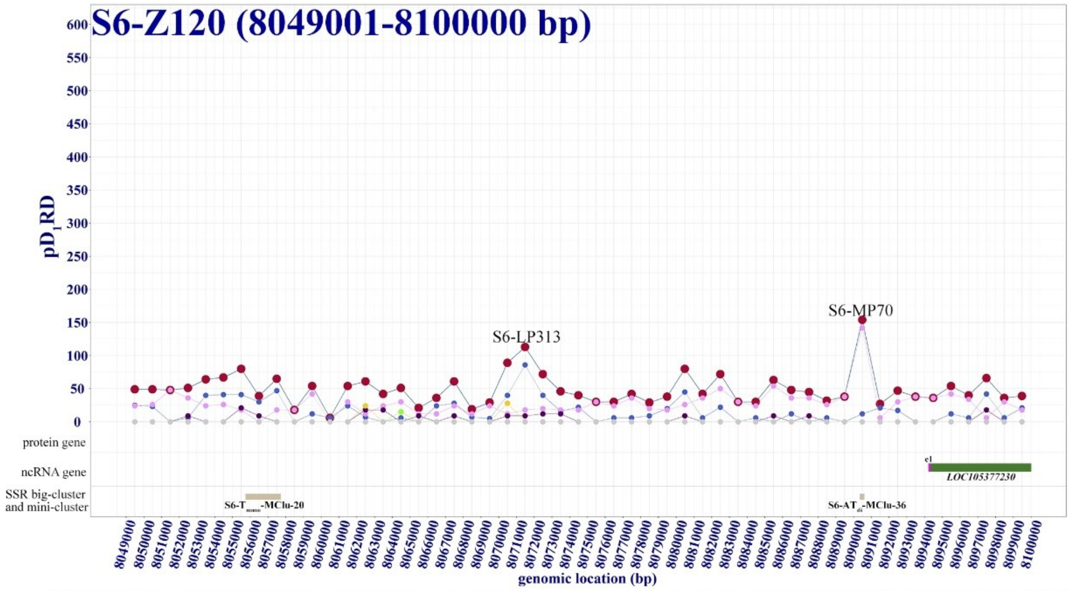

overall SSRs, mono-SSRs, di-SSRs, tri-SSRs, tetra-SSRs, penta-SSRs, hexa-SSRs

protein gene non-coding region (including intron and untranslated region), protein gene coding exon, ncRNA gene intron, ncRNA gene exon

SSR big-clusters, SSR mini-clusters

sHP (super high peak, $pD_1RD \geq 425.00$); **HP** (high peak, $300.00 \leq pD_1RD < 425.00$); **MP** (middle peak ($150.00 \leq pD_1RD < 300.00$); **LP** (low peak, $90.00 \leq pD_1RD < 150.00$).

bELR (big SSR extremely low density region, size ≥100 Kbp, relative density (RD) <25.00); **sELR** (small SSR extremely low density region, 3 Kbp ≤ size <100 Kbp, RD<25.00; **ZD** (SSR desert region with $pD_1RD = 0$, size ≤2 Kbp)

Supplementary Figure 1.158. The SSR position related D_1-relative density (pD_1RD) map of position at 8049001-8100000 bp of human reference Y-DNA (NC_000024.10) at resolution of 1 Kbp.

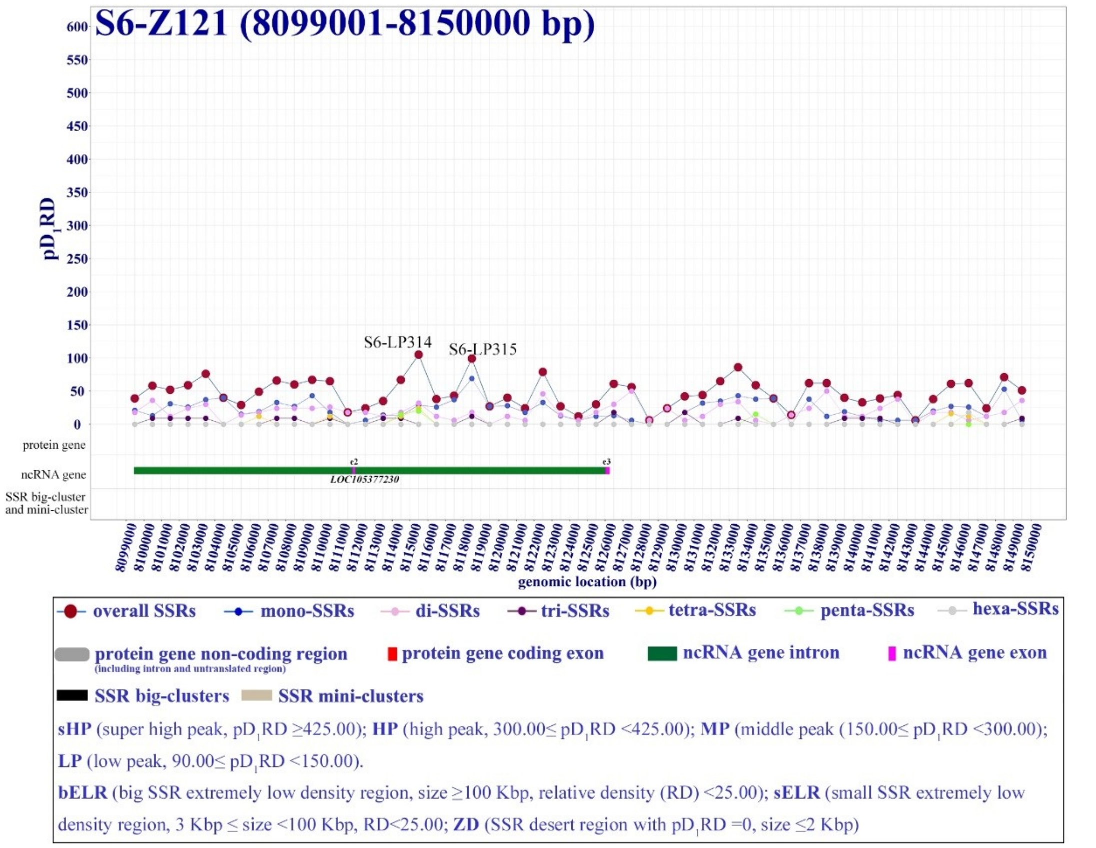

Supplementary Figure 1.159. The SSR position related D_1-relative density (pD_1RD) map of position at 8099001-8150000 bp of human reference Y-DNA (NC_000024.10) at resolution of 1 Kbp.

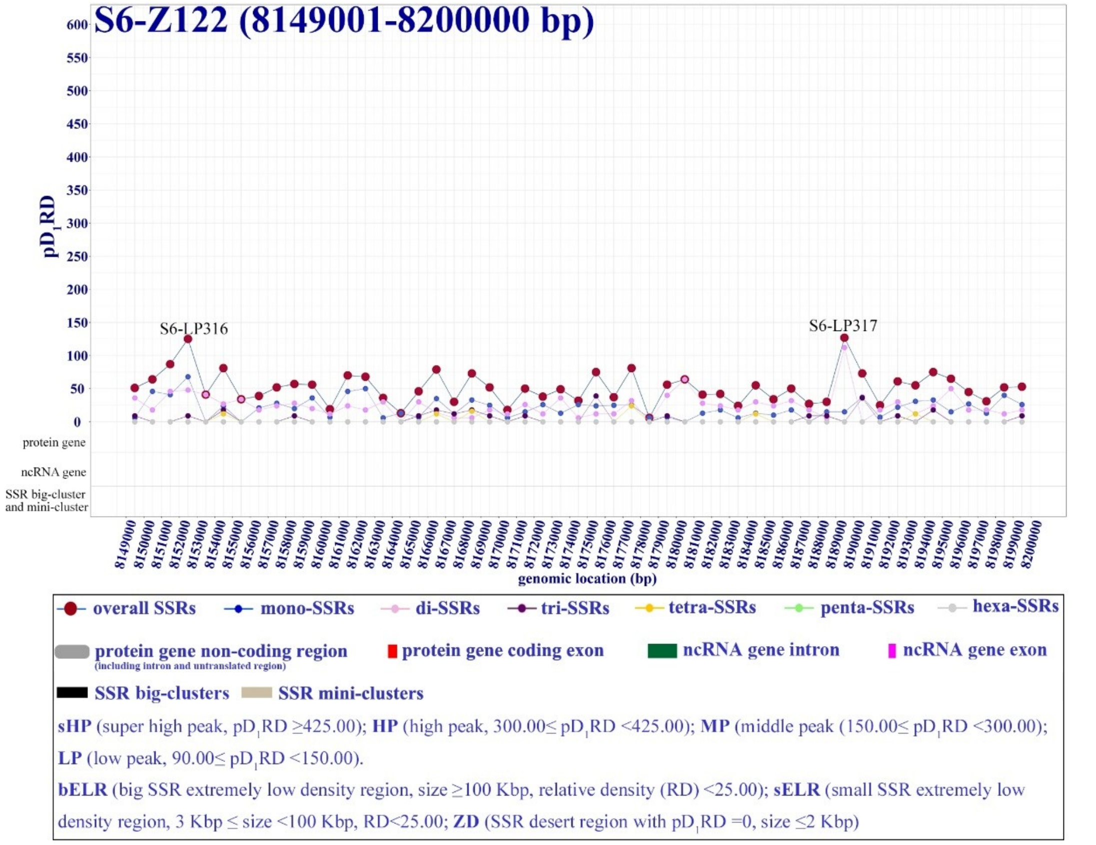

Supplementary Figure 1.160. The SSR position related D_1-relative density (pD_1RD) map of position at 8149001-8200000 bp of human reference Y-DNA (NC_000024.10) at resolution of 1 Kbp.

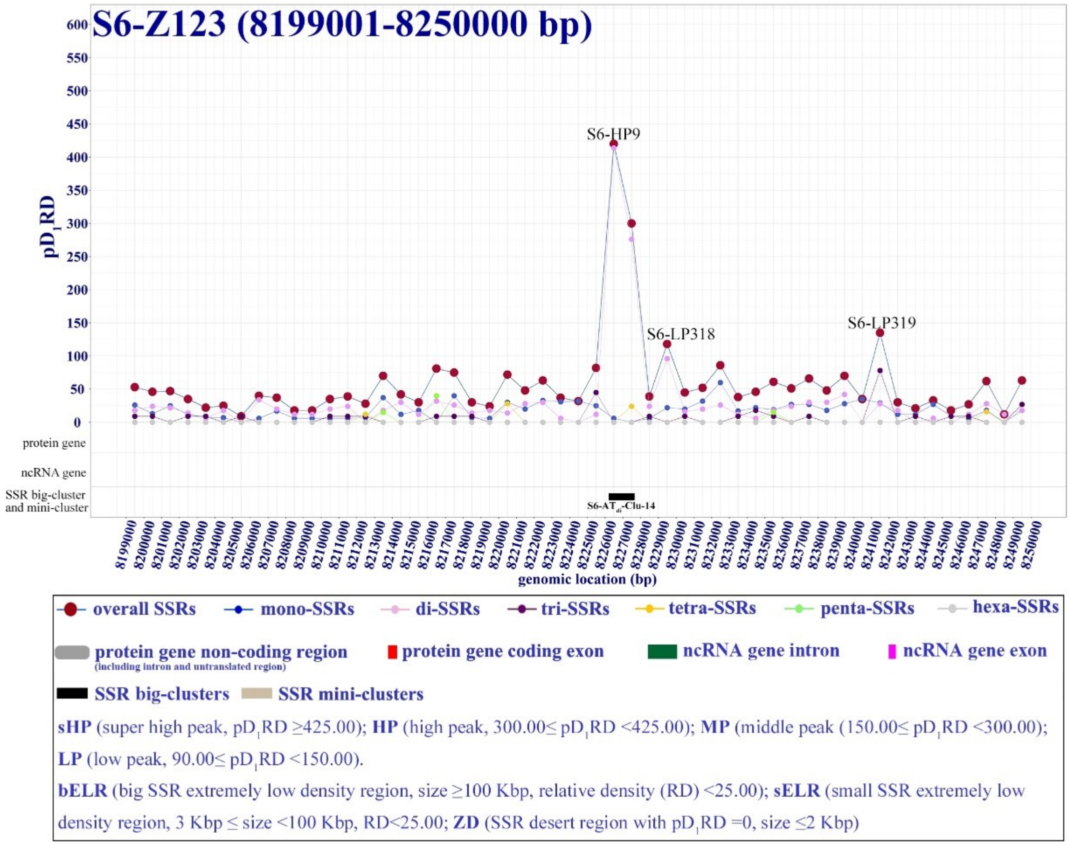

Supplementary Figure 1.161. The SSR position related D_1-relative density (pD_1RD) map of position at 8199001-8250000 bp of human reference Y-DNA (NC_000024.10) at resolution of 1 Kbp.

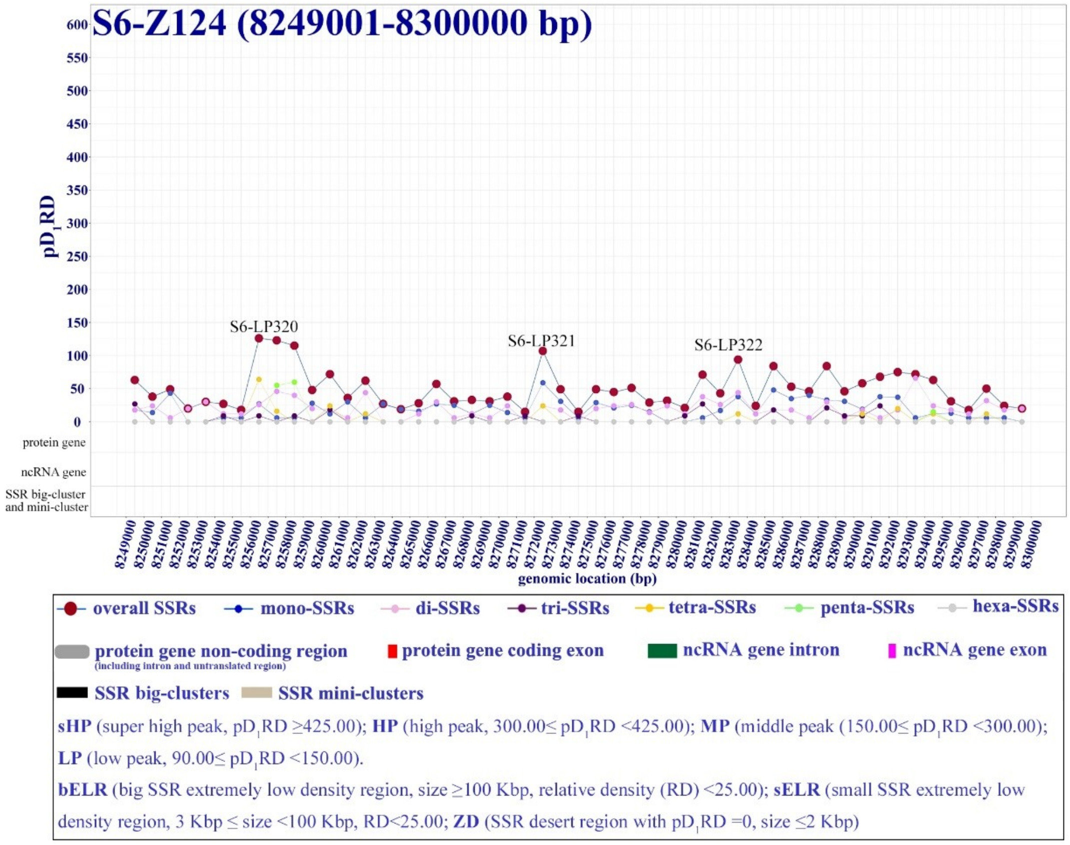

Supplementary Figure 1.162. The SSR position related D_1-relative density (pD_1RD) map of position at 8249001-8300000 bp of human reference Y-DNA (NC_000024.10) at resolution of 1 Kbp.

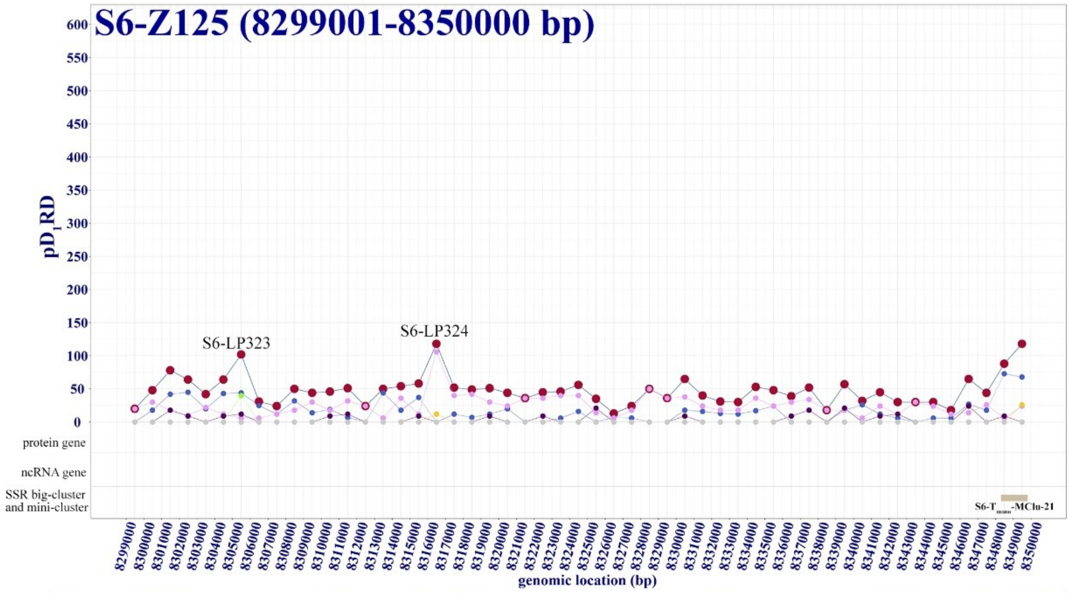

overall SSRs — mono-SSRs — di-SSRs — tri-SSRs — tetra-SSRs — penta-SSRs — hexa-SSRs

protein gene non-coding region (including intron and untranslated region) — protein gene coding exon — ncRNA gene intron — ncRNA gene exon

SSR big-clusters — SSR mini-clusters

sHP (super high peak, $pD_1RD \geq 425.00$); **HP** (high peak, $300.00 \leq pD_1RD < 425.00$); **MP** (middle peak ($150.00 \leq pD_1RD < 300.00$); **LP** (low peak, $90.00 \leq pD_1RD < 150.00$).

bELR (big SSR extremely low density region, size ≥100 Kbp, relative density (RD) <25.00); **sELR** (small SSR extremely low density region, 3 Kbp ≤ size <100 Kbp, RD<25.00; **ZD** (SSR desert region with $pD_1RD = 0$, size ≤2 Kbp)

Supplementary Figure 1.163. The SSR position related D_1-relative density (pD_1RD) map of position at 8299001-8350000 bp of human reference Y-DNA (NC_000024.10) at resolution of 1 Kbp.

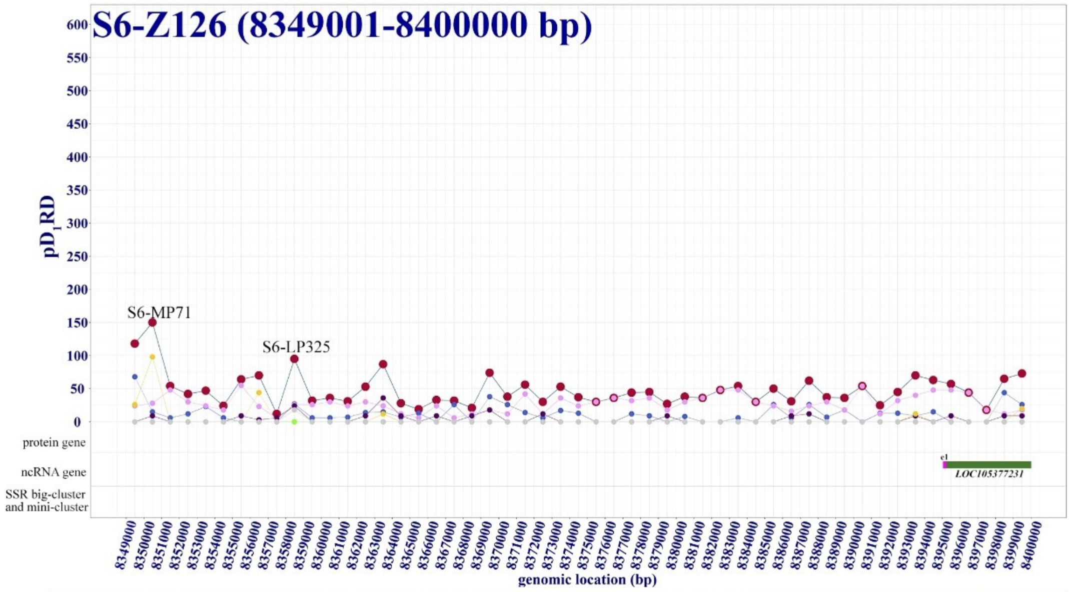

overall SSRs — mono-SSRs — di-SSRs — tri-SSRs — tetra-SSRs — penta-SSRs — hexa-SSRs

protein gene non-coding region (including intron and untranslated region) — protein gene coding exon — ncRNA gene intron — ncRNA gene exon

SSR big-clusters — SSR mini-clusters

sHP (super high peak, $pD_1RD \geq 425.00$); **HP** (high peak, $300.00 \leq pD_1RD < 425.00$); **MP** (middle peak ($150.00 \leq pD_1RD < 300.00$); **LP** (low peak, $90.00 \leq pD_1RD < 150.00$).

bELR (big SSR extremely low density region, size ≥100 Kbp, relative density (RD) <25.00); **sELR** (small SSR extremely low density region, 3 Kbp ≤ size <100 Kbp, RD<25.00; **ZD** (SSR desert region with $pD_1RD = 0$, size ≤2 Kbp)

Supplementary Figure 1.164. The SSR position related D_1-relative density (pD_1RD) map of position at 8349001-8400000 bp of human reference Y-DNA (NC_000024.10) at resolution of 1 Kbp.

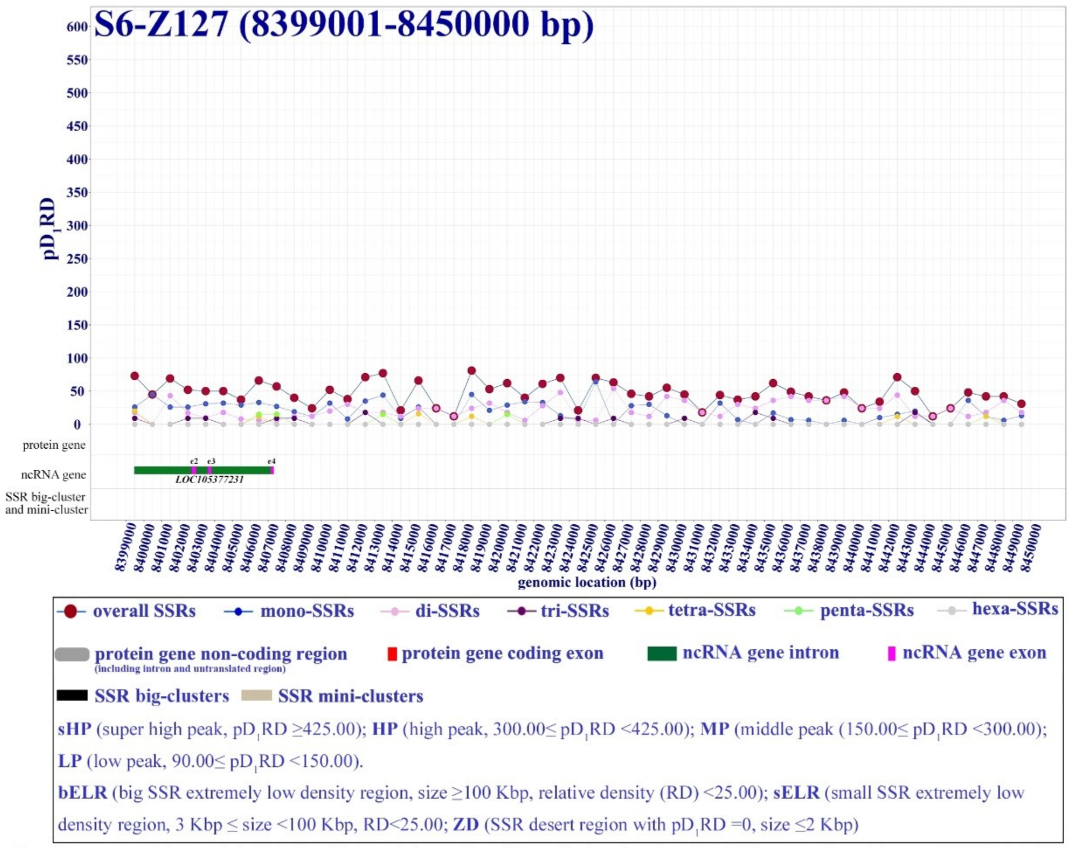

Supplementary Figure 1.165. The SSR position related D_1-relative density (pD_1RD) map of position at 8399001-8450000 bp of human reference Y-DNA (NC_000024.10) at resolution of 1 Kbp.

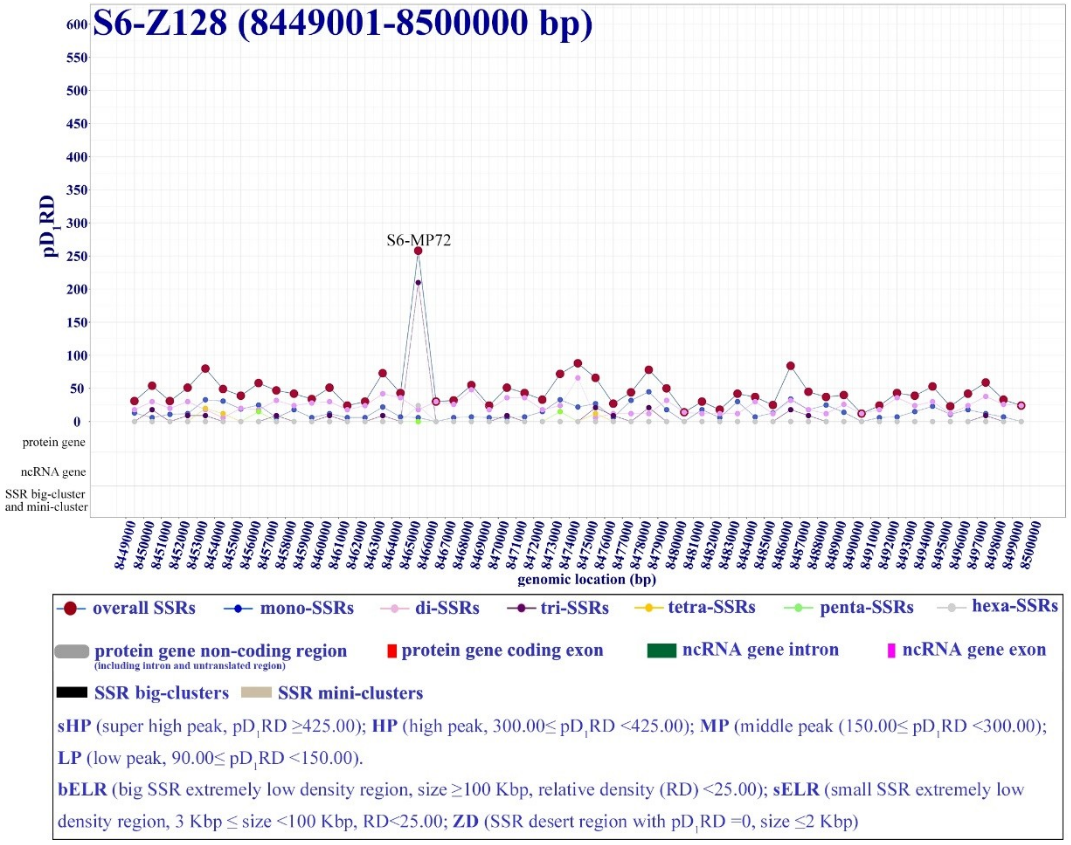

Supplementary Figure 1.166. The SSR position related D_1-relative density (pD_1RD) map of position at 8449001-8500000 bp of human reference Y-DNA (NC_000024.10) at resolution of 1 Kbp.

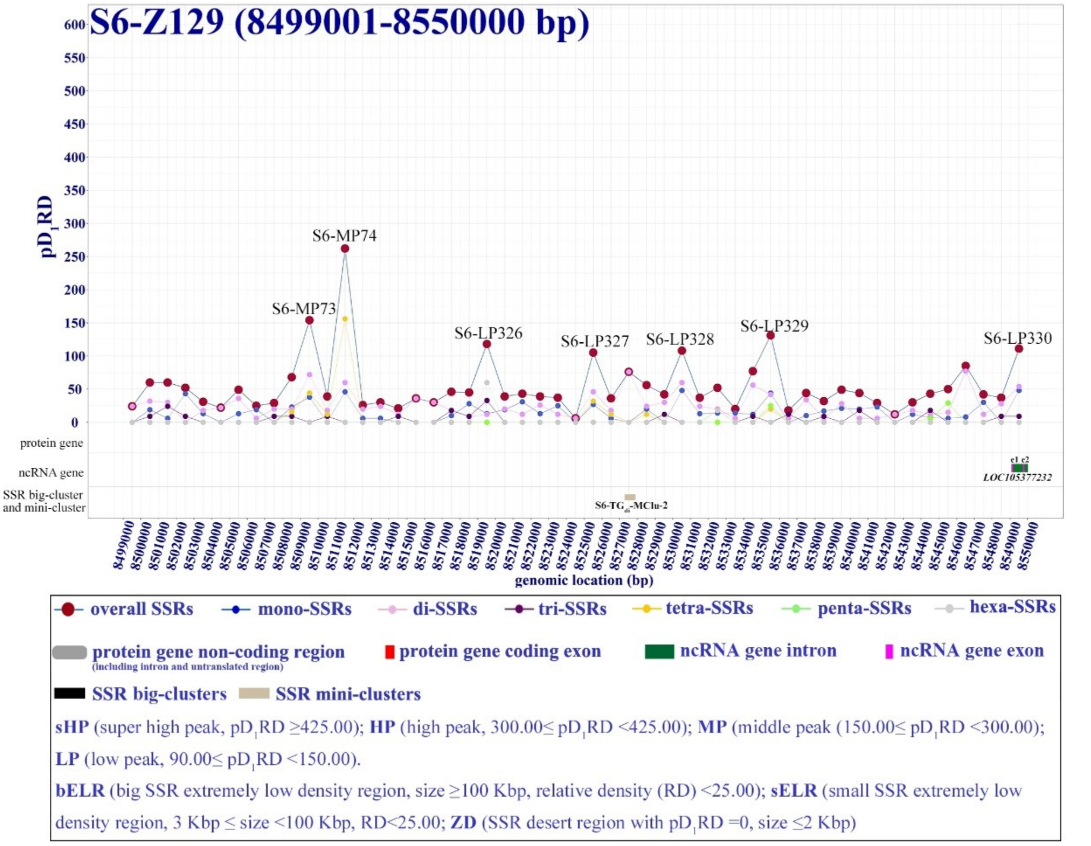

Supplementary Figure 1.167. The SSR position related D_1-relative density (pD_1RD) map of position at 8499001-8550000 bp of human reference Y-DNA (NC_000024.10) at resolution of 1 Kbp.

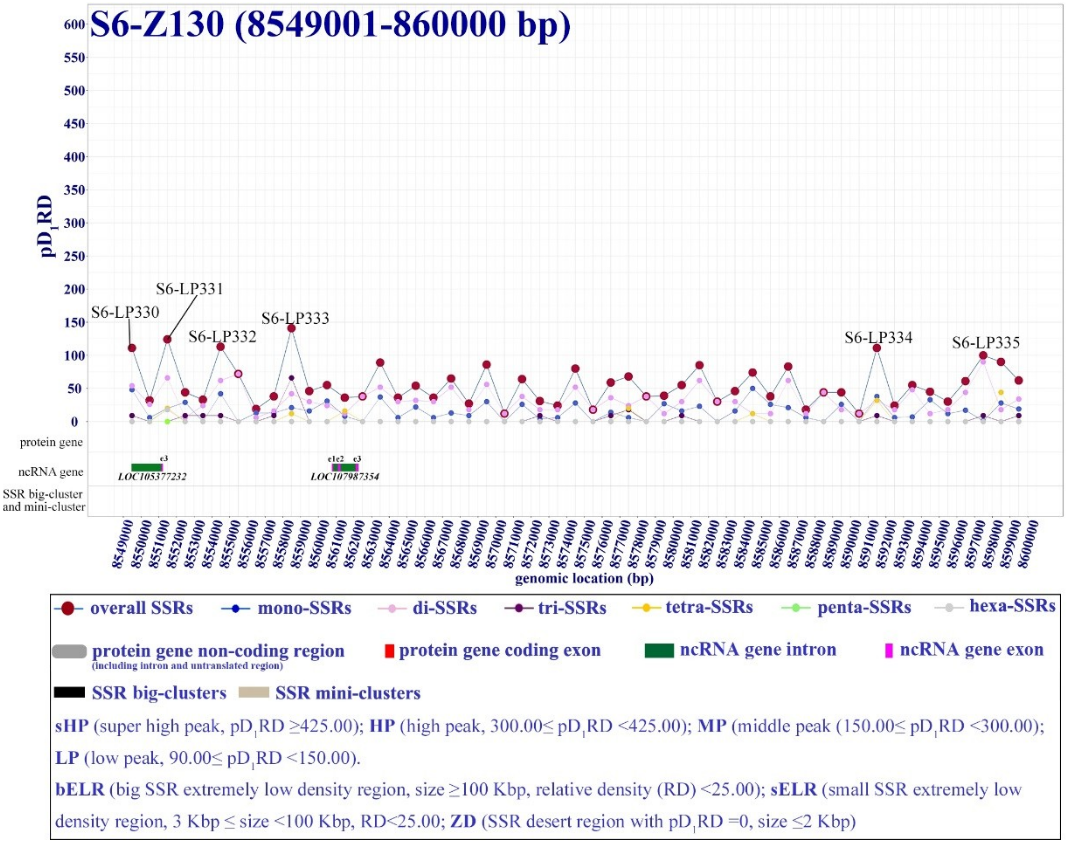

Supplementary Figure 1.168. The SSR position related D_1-relative density (pD_1RD) map of position at 8549001-8600000 bp of human reference Y-DNA (NC_000024.10) at resolution of 1 Kbp.

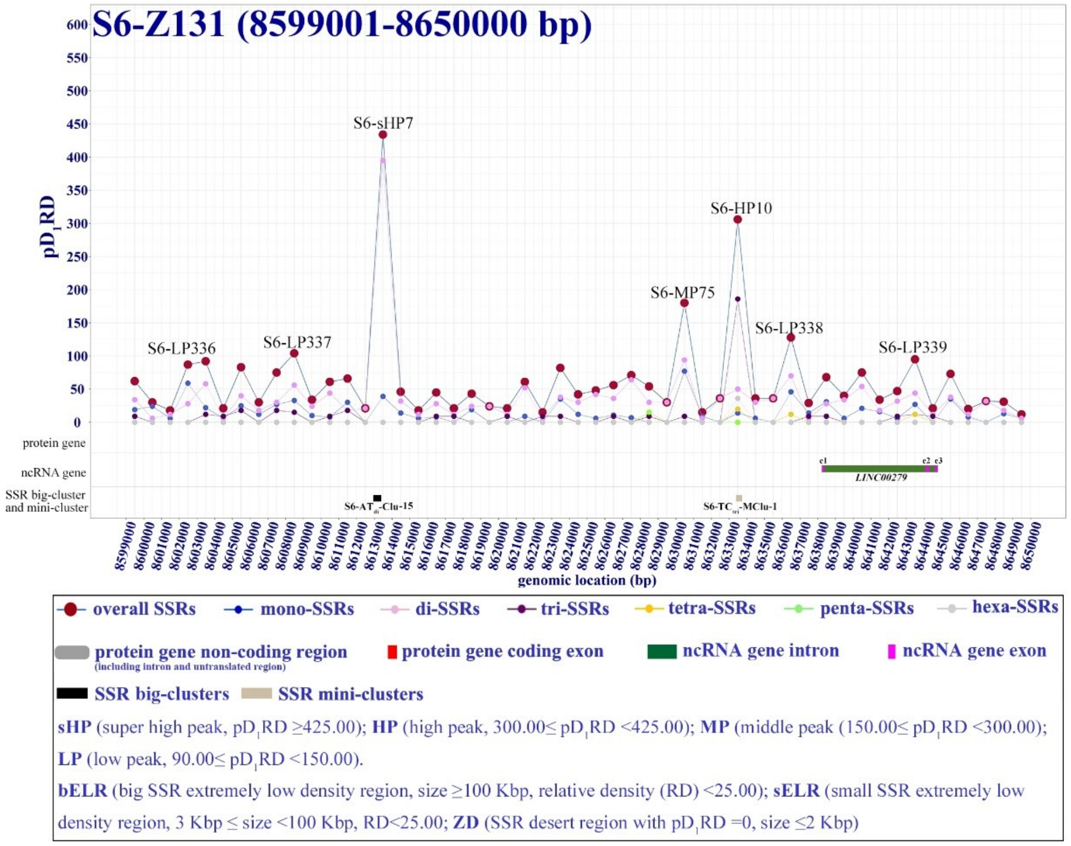

Supplementary Figure 1.169. The SSR position related D_1-relative density (pD_1RD) map of position at 8599001-8650000 bp of human reference Y-DNA (NC_000024.10) at resolution of 1 Kbp.

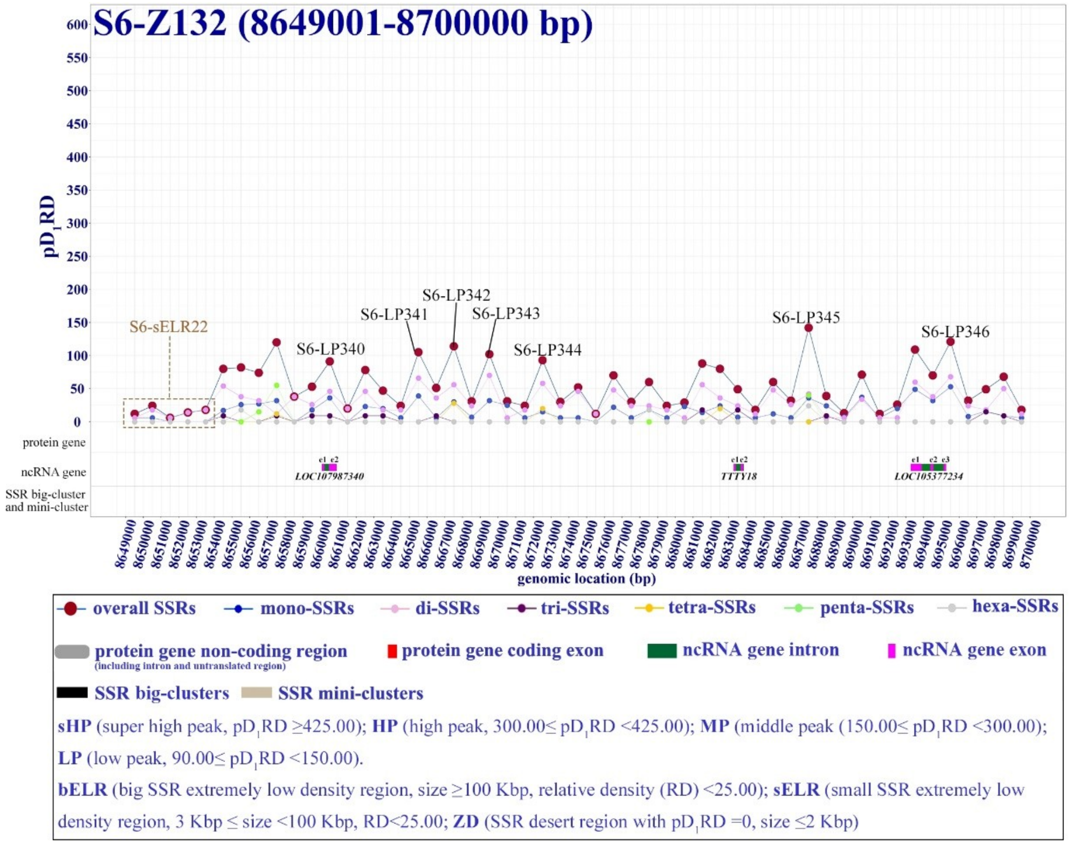

Supplementary Figure 1.170. The SSR position related D_1-relative density (pD_1RD) map of position at 8649001-8700000 bp of human reference Y-DNA (NC_000024.10) at resolution of 1 Kbp.

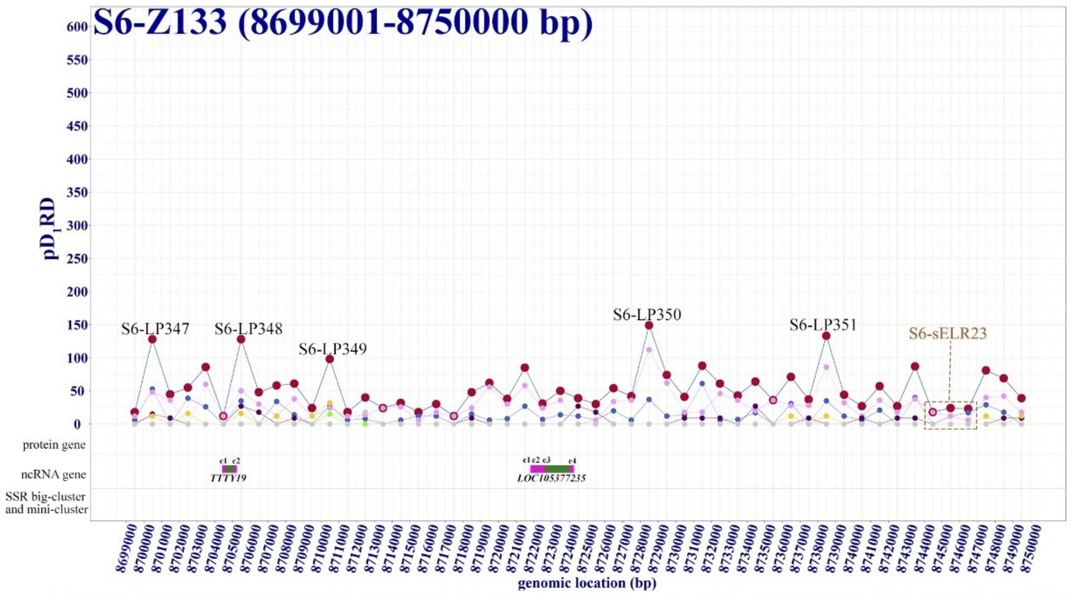

overall SSRs · mono-SSRs · di-SSRs · tri-SSRs · tetra-SSRs · penta-SSRs · hexa-SSRs

protein gene non-coding region (including intron and untranslated region) · protein gene coding exon · ncRNA gene intron · ncRNA gene exon

SSR big-clusters · SSR mini-clusters

sHP (super high peak, $pD_1RD \geq 425.00$); **HP** (high peak, $300.00 \leq pD_1RD < 425.00$); **MP** (middle peak ($150.00 \leq pD_1RD < 300.00$); **LP** (low peak, $90.00 \leq pD_1RD < 150.00$).

bELR (big SSR extremely low density region, size ≥100 Kbp, relative density (RD) <25.00); **sELR** (small SSR extremely low density region, 3 Kbp ≤ size <100 Kbp, RD<25.00; **ZD** (SSR desert region with $pD_1RD = 0$, size ≤2 Kbp)

Supplementary Figure 1.171. The SSR position related D_1-relative density (pD_1RD) map of position at 8699001-8750000 bp of human reference Y-DNA (NC_000024.10) at resolution of 1 Kbp.

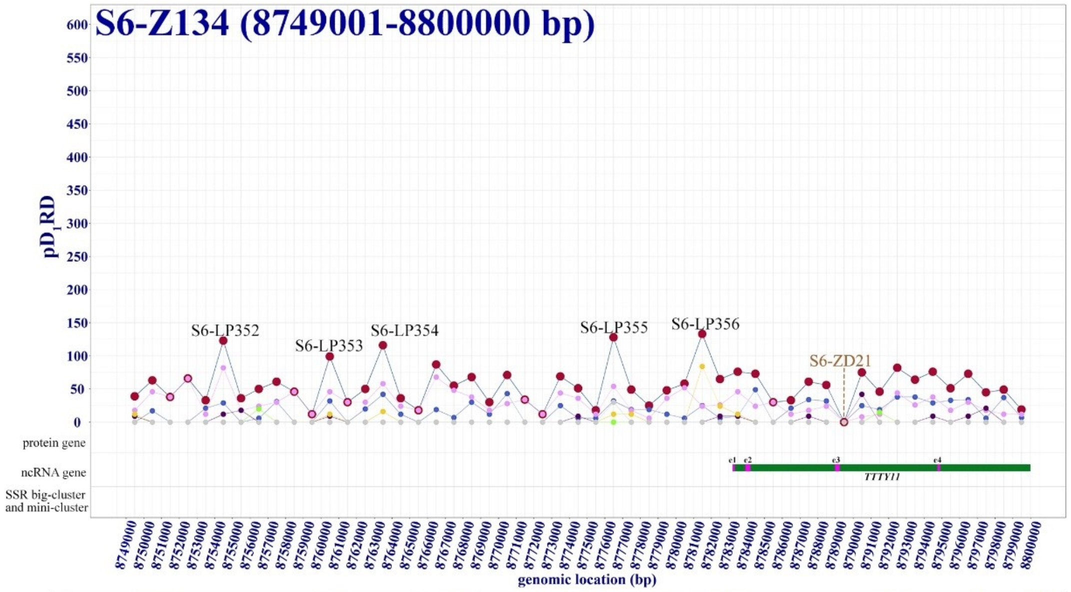

overall SSRs · mono-SSRs · di-SSRs · tri-SSRs · tetra-SSRs · penta-SSRs · hexa-SSRs

protein gene non-coding region (including intron and untranslated region) · protein gene coding exon · ncRNA gene intron · ncRNA gene exon

SSR big-clusters · SSR mini-clusters

sHP (super high peak, $pD_1RD \geq 425.00$); **HP** (high peak, $300.00 \leq pD_1RD < 425.00$); **MP** (middle peak ($150.00 \leq pD_1RD < 300.00$); **LP** (low peak, $90.00 \leq pD_1RD < 150.00$).

bELR (big SSR extremely low density region, size ≥100 Kbp, relative density (RD) <25.00); **sELR** (small SSR extremely low density region, 3 Kbp ≤ size <100 Kbp, RD<25.00; **ZD** (SSR desert region with $pD_1RD = 0$, size ≤2 Kbp)

Supplementary Figure 1.172. The SSR position related D_1-relative density (pD_1RD) map of position at 8749001-8800000 bp of human reference Y-DNA (NC_000024.10) at resolution of 1 Kbp.

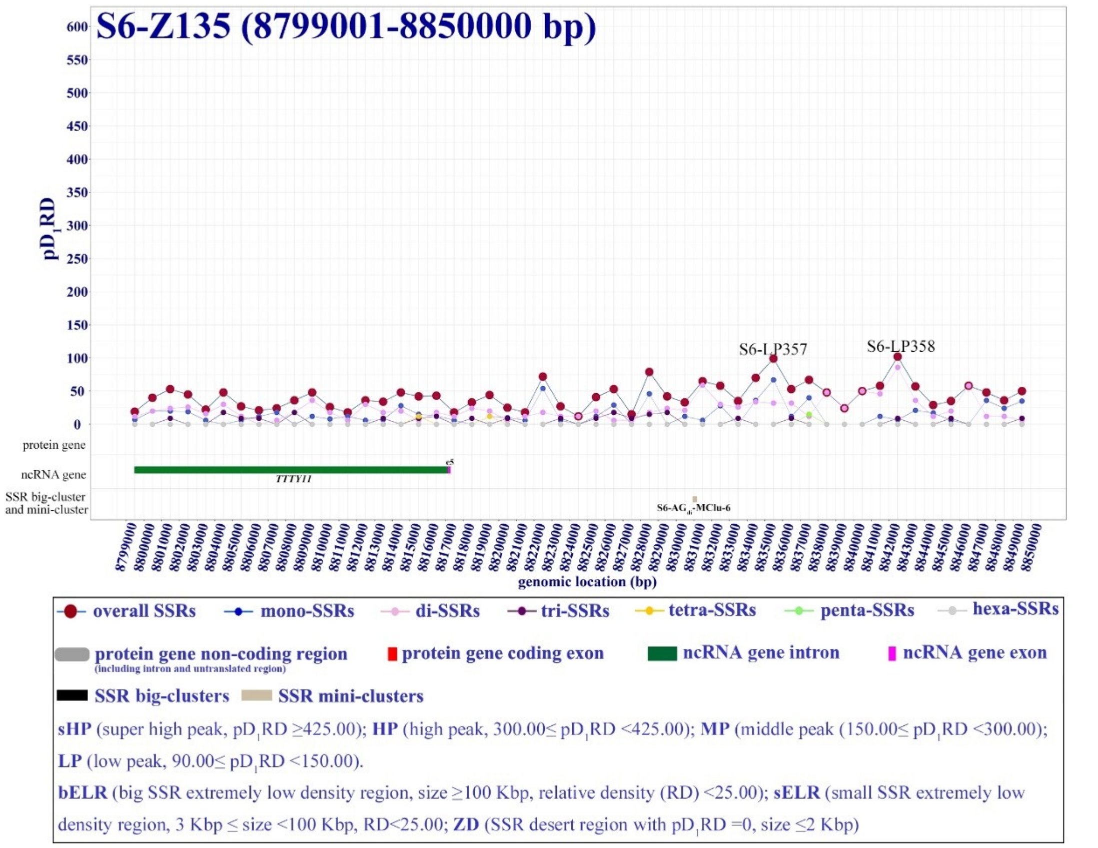

Supplementary Figure 1.173. The SSR position related D_1-relative density (pD_1RD) map of position at 8799001-8850000 bp of human reference Y-DNA (NC_000024.10) at resolution of 1 Kbp.

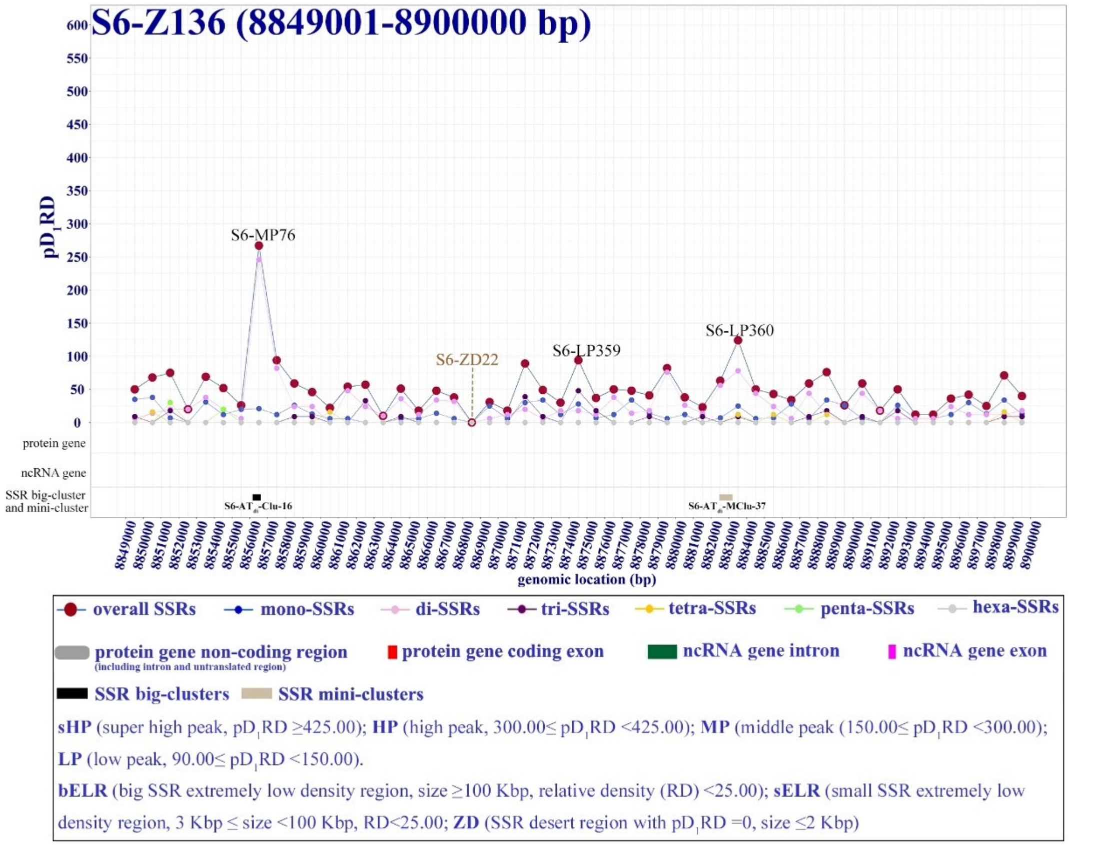

Supplementary Figure 1.174. The SSR position related D_1-relative density (pD_1RD) map of position at 8849001-8900000 bp of human reference Y-DNA (NC_000024.10) at resolution of 1 Kbp.

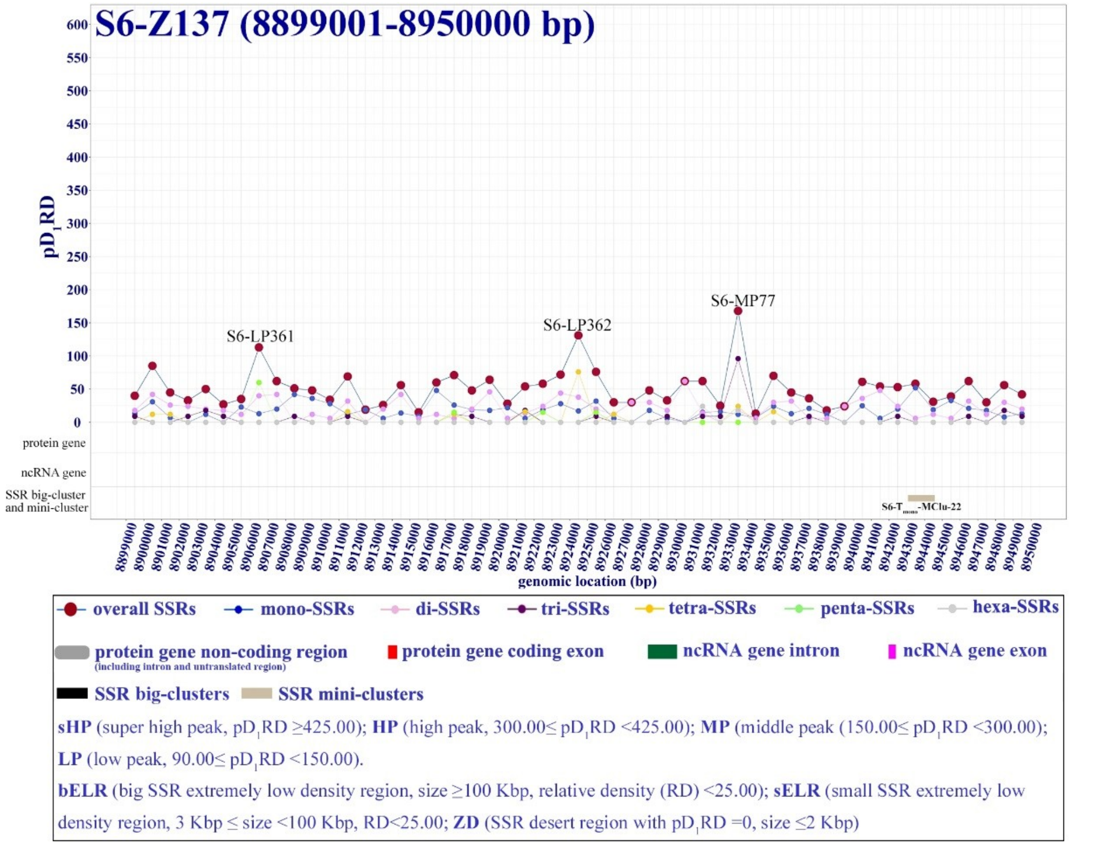

Supplementary Figure 1.175. The SSR position related D_1-relative density (pD_1RD) map of position at 8899001-8950000 bp of human reference Y-DNA (NC_000024.10) at resolution of 1 Kbp.

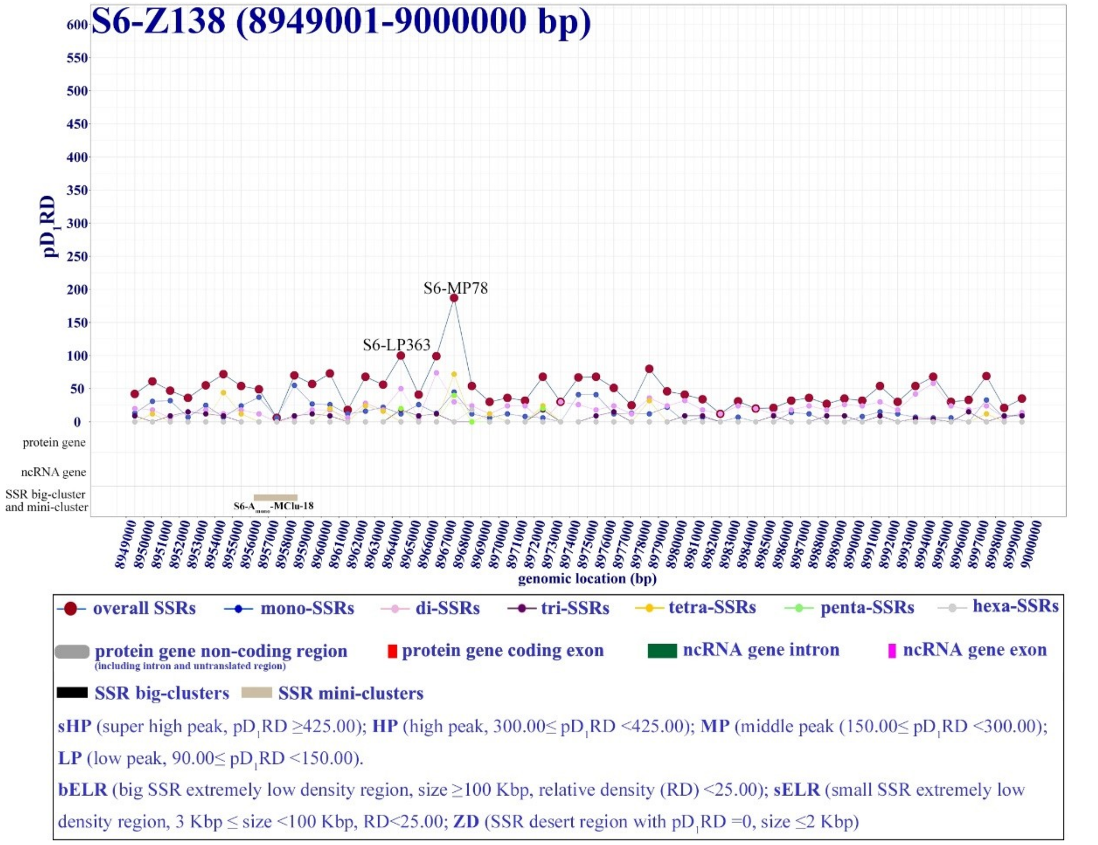

Supplementary Figure 1.176. The SSR position related D_1-relative density (pD_1RD) map of position at 8949001-9000000 bp of human reference Y-DNA (NC_000024.10) at resolution of 1 Kbp.

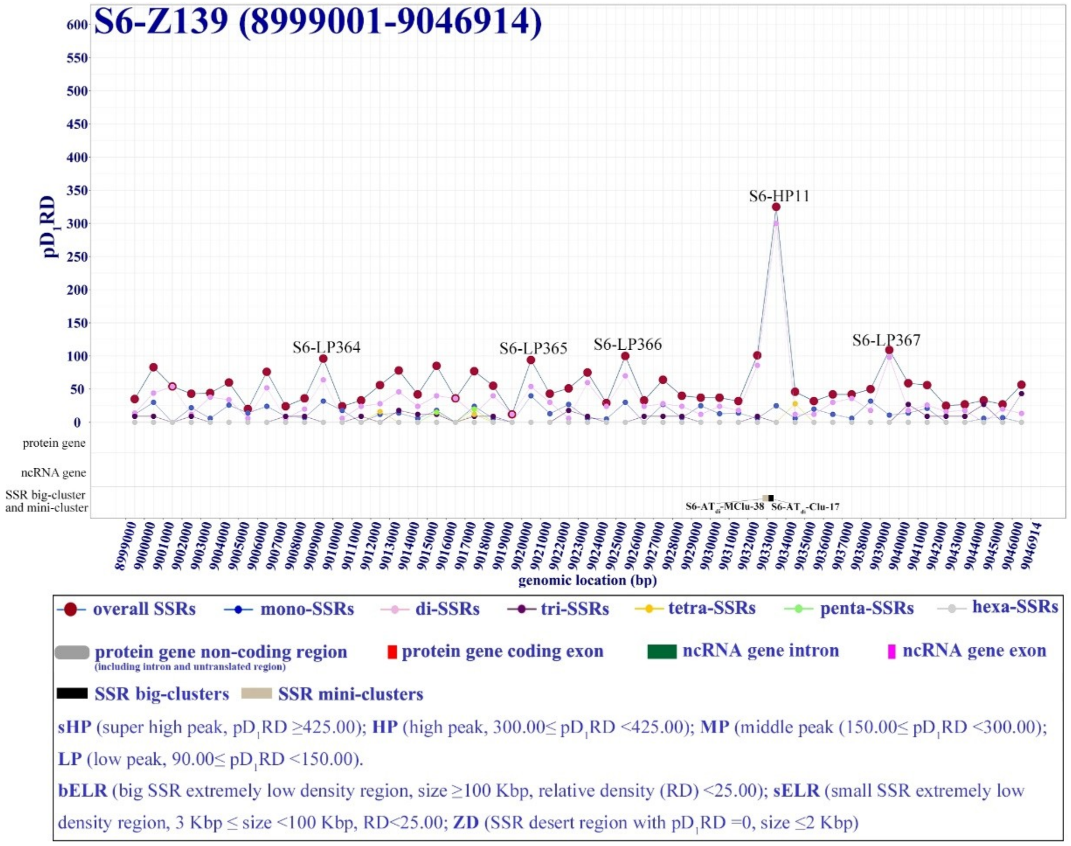

Supplementary Figure 1.177. The SSR position related D_1-relative density (pD_1RD) map of position at 8999001-9046914 bp (unnormal zone <51000 bp) of human reference Y-DNA (NC_000024.10) at resolution of 1 Kbp.

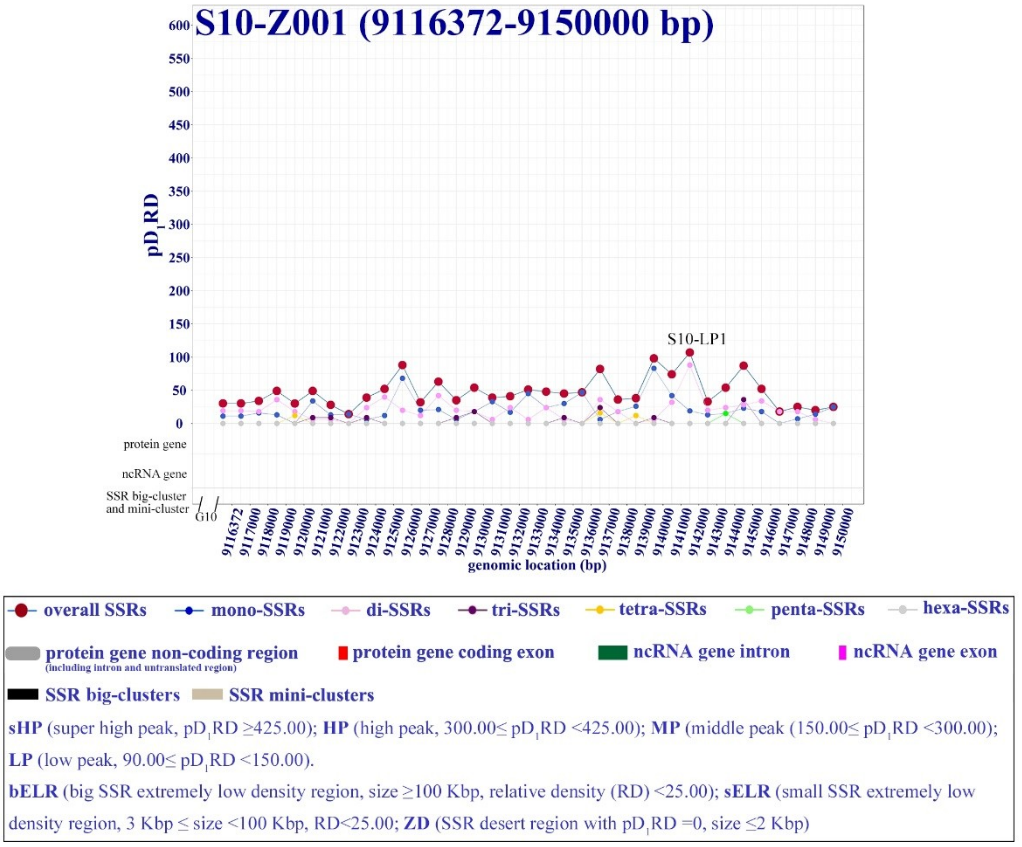

Supplementary Figure 1.178. The SSR position related D_1-relative density (pD_1RD) map of position at 9116372-9150000 bp (unnormal zone <51000 bp) of human reference Y-DNA (NC_000024.10) at resolution of 1 Kbp.

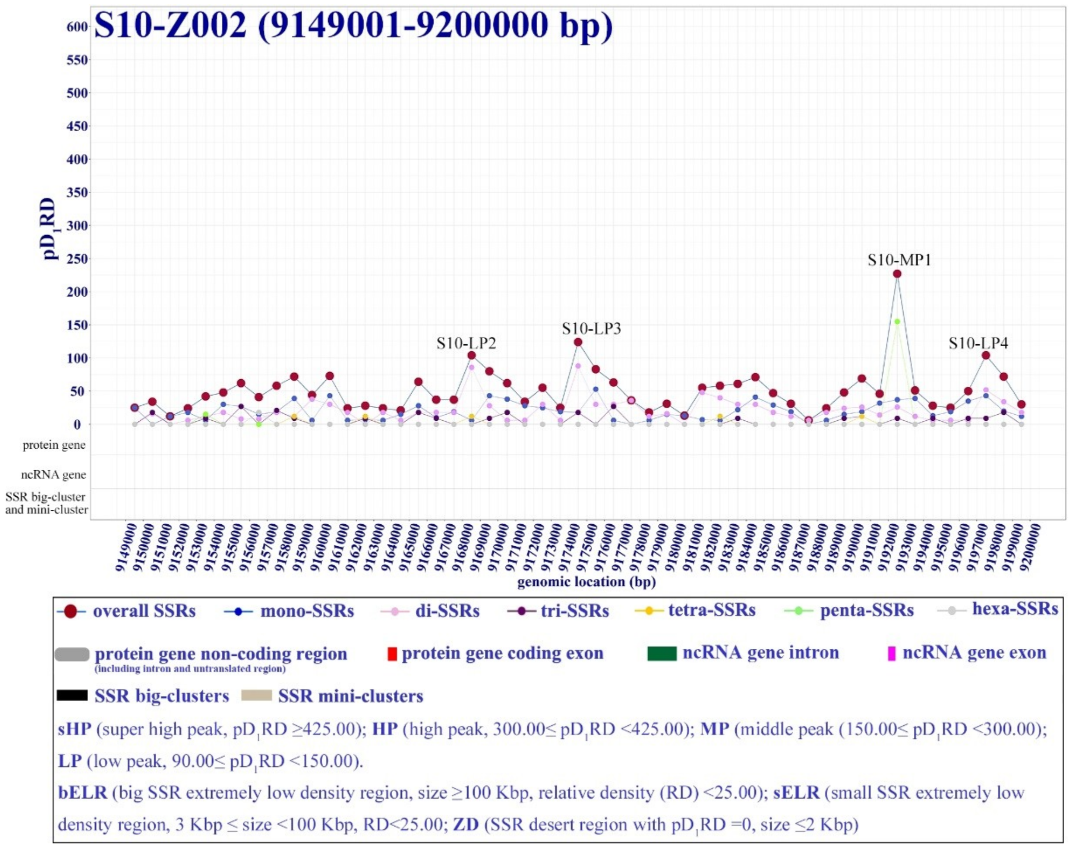

Supplementary Figure 1.179. The SSR position related D_1-relative density (pD_1RD) map of position at 9149001-9200000 bp of human reference Y-DNA (NC_000024.10) at resolution of 1 Kbp.

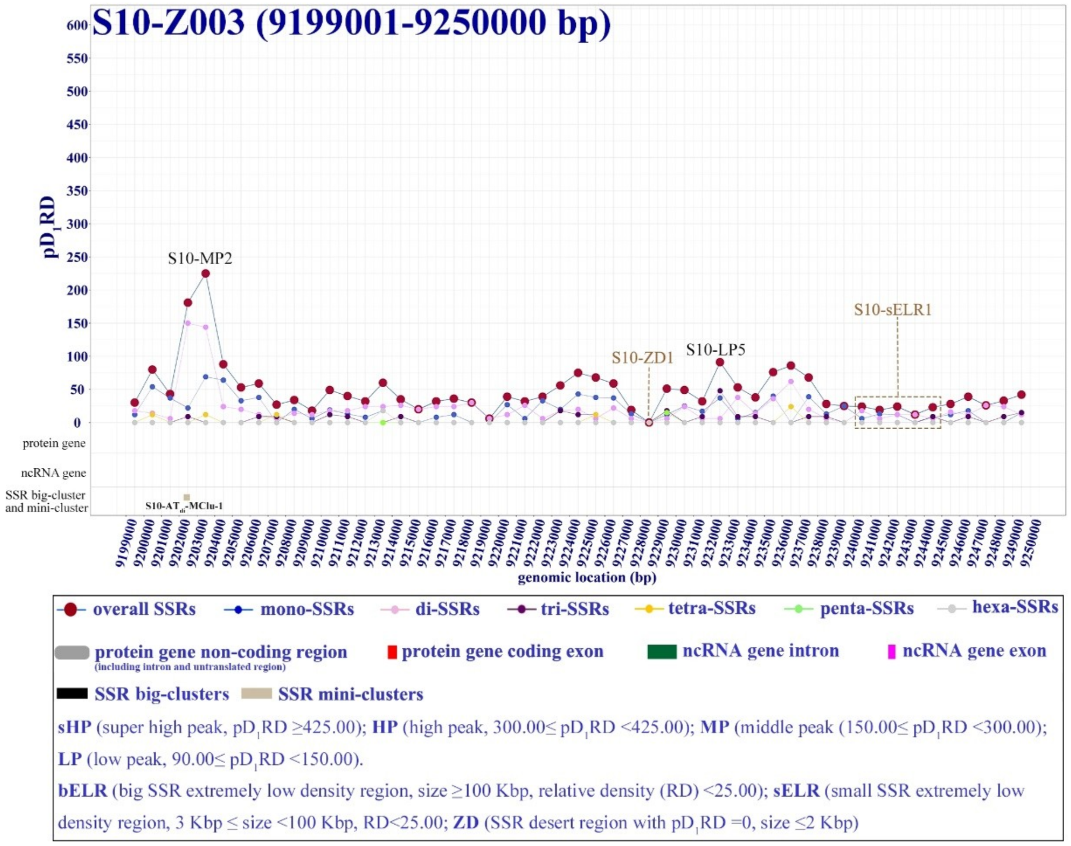

Supplementary Figure 1.180. The SSR position related D_1-relative density (pD_1RD) map of position at 9199001-9250000 bp of human reference Y-DNA (NC_000024.10) at resolution of 1 Kbp.

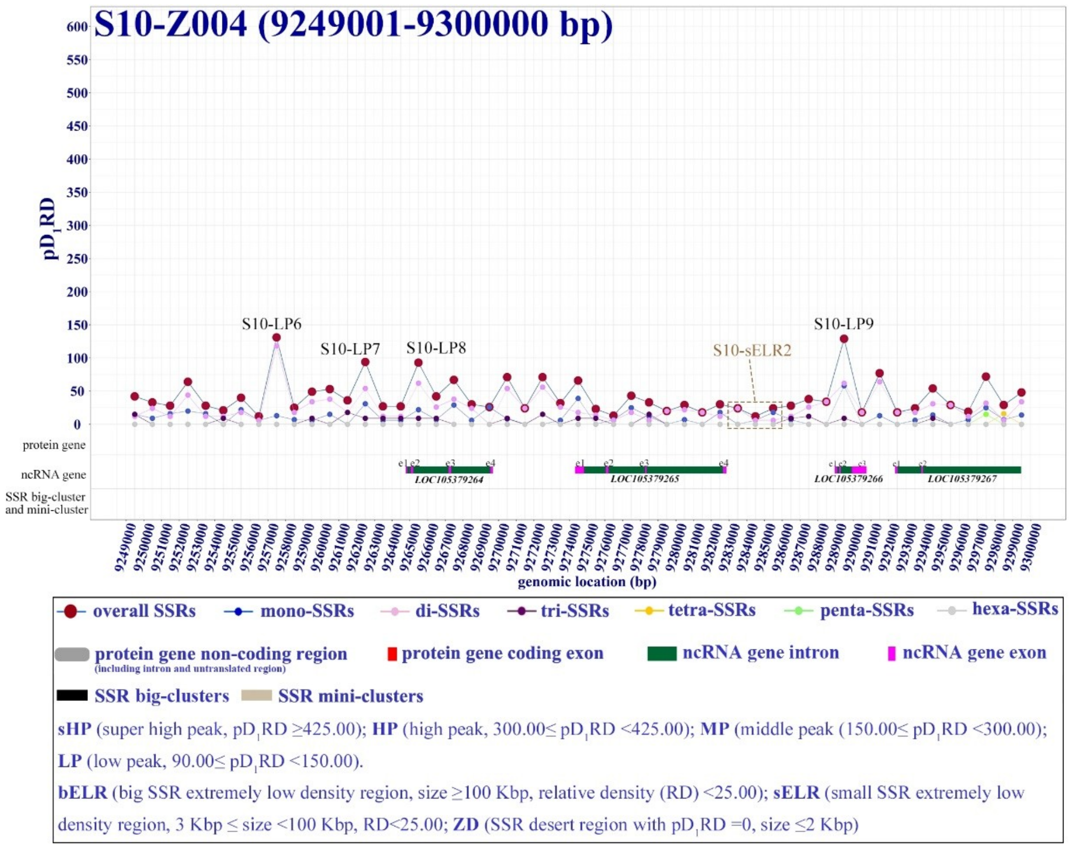

Supplementary Figure 1.181. The SSR position related D_1-relative density (pD_1RD) map of position at 9249001-9300000 bp of human reference Y-DNA (NC_000024.10) at resolution of 1 Kbp.

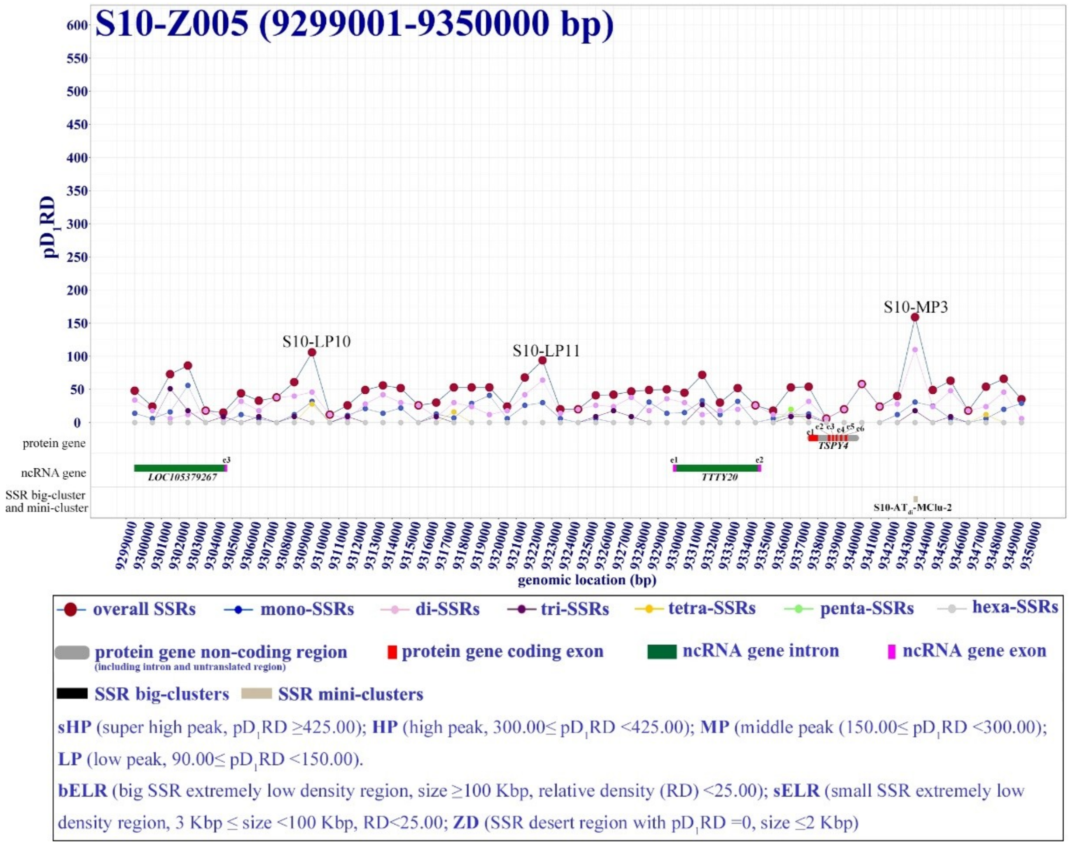

Supplementary Figure 1.182. The SSR position related D_1-relative density (pD_1RD) map of position at 9299001-9350000 bp of human reference Y-DNA (NC_000024.10) at resolution of 1 Kbp.

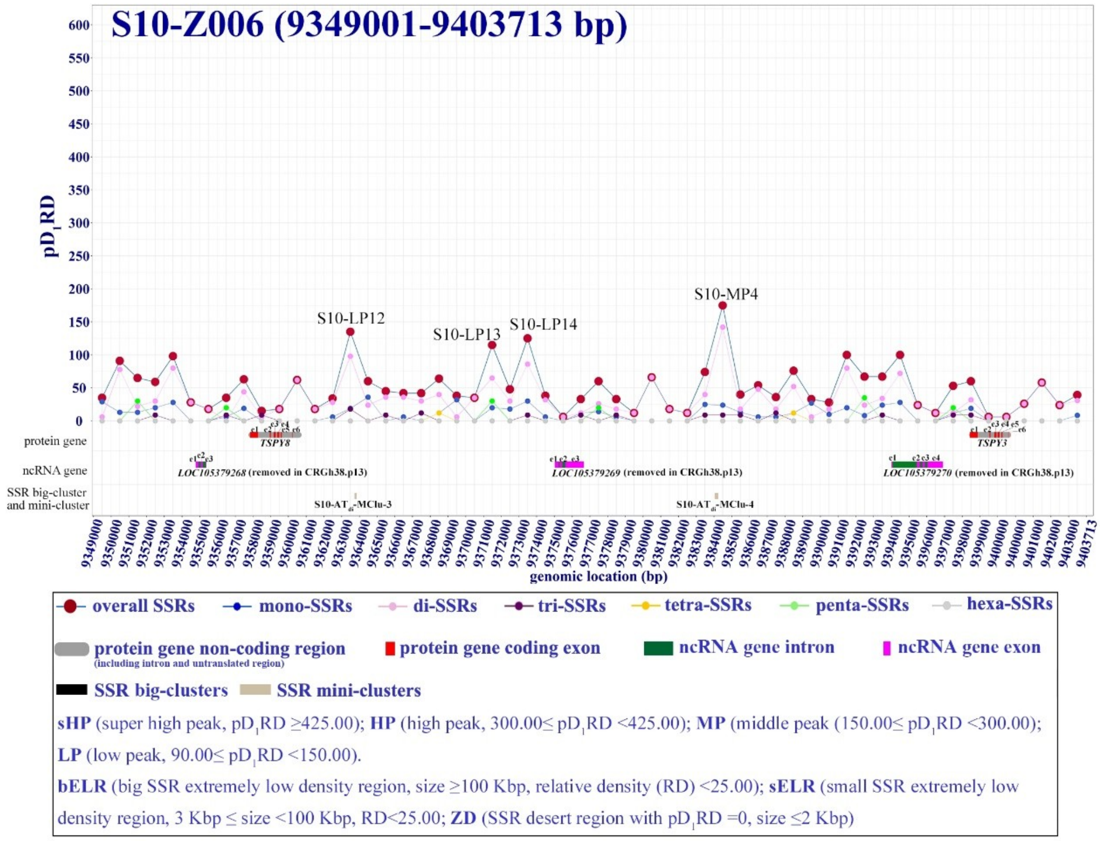

Supplementary Figure 1.183. The SSR position related D_1-relative density (pD_1RD) map of position at 9349001-9403713 bp of human reference Y-DNA (NC_000024.10) at resolution of 1 Kbp.

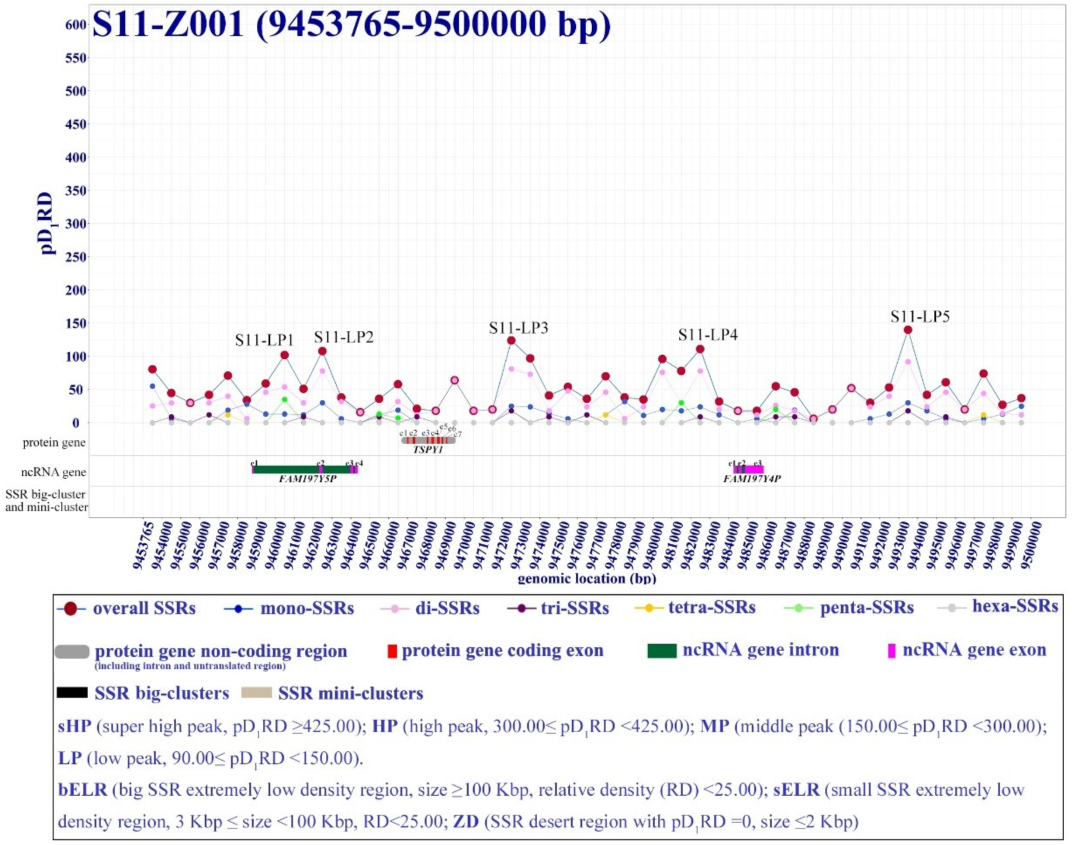

Supplementary Figure 1.184. The SSR position related D_1-relative density (pD_1RD) map of position at 9453765-9500000 bp (unnormal zone <51000 bp) of human reference Y-DNA (NC_000024.10) at resolution of 1 Kbp.

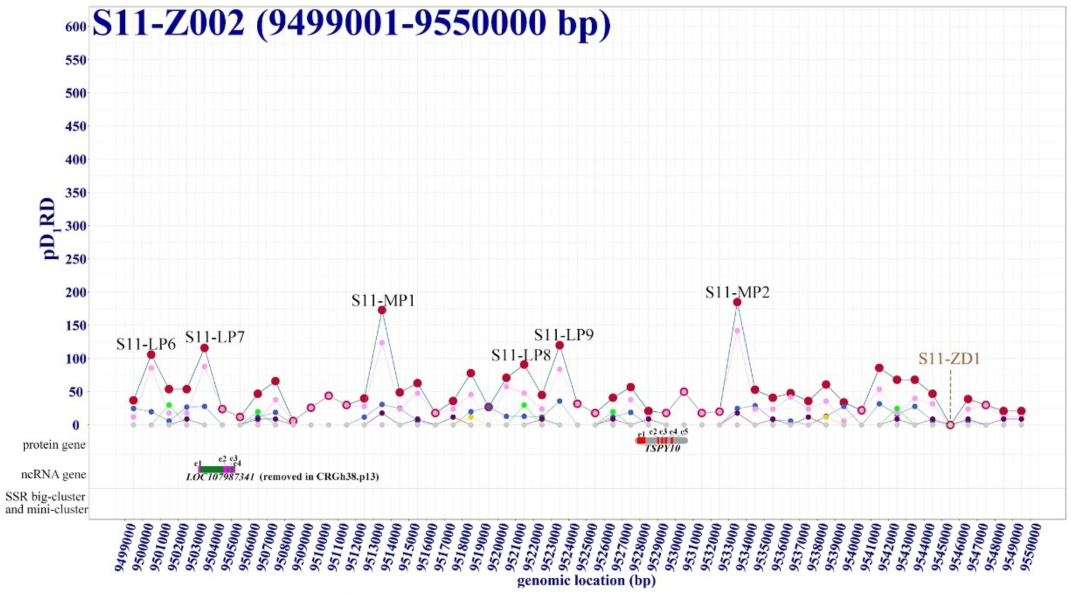

overall SSRs — mono-SSRs — di-SSRs — tri-SSRs — tetra-SSRs — penta-SSRs — hexa-SSRs

protein gene non-coding region (including intron and untranslated region) — protein gene coding exon — ncRNA gene intron — ncRNA gene exon

SSR big-clusters — SSR mini-clusters

sHP (super high peak, $pD_1RD \geq 425.00$); **HP** (high peak, $300.00 \leq pD_1RD < 425.00$); **MP** (middle peak ($150.00 \leq pD_1RD < 300.00$); **LP** (low peak, $90.00 \leq pD_1RD < 150.00$).

bELR (big SSR extremely low density region, size ≥100 Kbp, relative density (RD) <25.00); **sELR** (small SSR extremely low density region, 3 Kbp ≤ size <100 Kbp, RD<25.00; **ZD** (SSR desert region with $pD_1RD = 0$, size ≤2 Kbp)

Supplementary Figure 1.185. The SSR position related D_1-relative density (pD_1RD) map of position at 9499001-9550000 bp of human reference Y-DNA (NC_000024.10) at resolution of 1 Kbp.

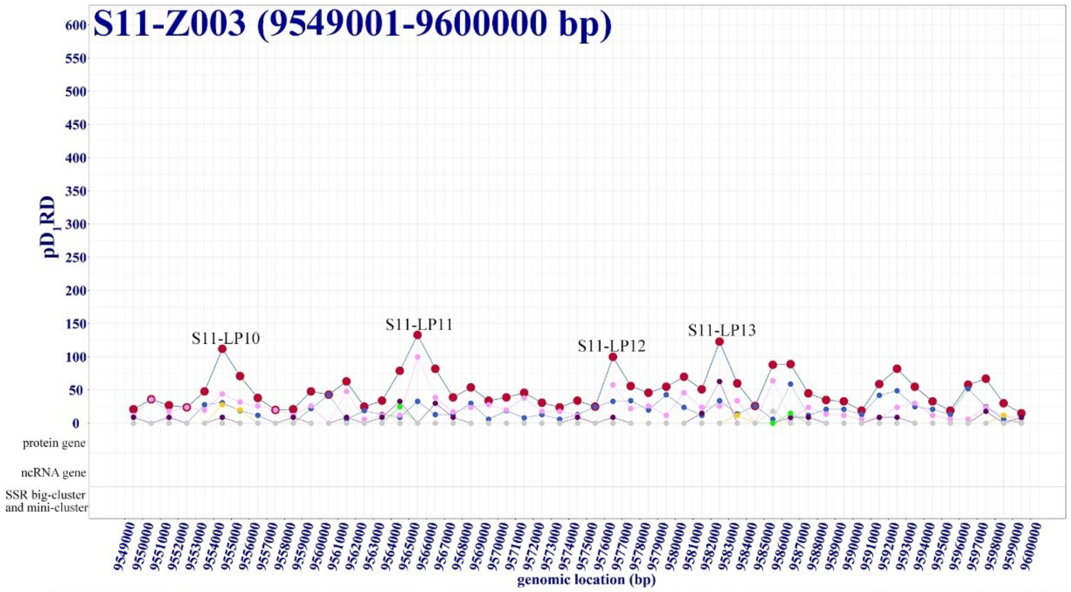

overall SSRs — mono-SSRs — di-SSRs — tri-SSRs — tetra-SSRs — penta-SSRs — hexa-SSRs

protein gene non-coding region (including intron and untranslated region) — protein gene coding exon — ncRNA gene intron — ncRNA gene exon

SSR big-clusters — SSR mini-clusters

sHP (super high peak, $pD_1RD \geq 425.00$); **HP** (high peak, $300.00 \leq pD_1RD < 425.00$); **MP** (middle peak ($150.00 \leq pD_1RD < 300.00$); **LP** (low peak, $90.00 \leq pD_1RD < 150.00$).

bELR (big SSR extremely low density region, size ≥100 Kbp, relative density (RD) <25.00); **sELR** (small SSR extremely low density region, 3 Kbp ≤ size <100 Kbp, RD<25.00; **ZD** (SSR desert region with $pD_1RD = 0$, size ≤2 Kbp)

Supplementary Figure 1.186. The SSR position related D_1-relative density (pD_1RD) map of position at 9549001-9600000 bp of human reference Y-DNA (NC_000024.10) at resolution of 1 Kbp.

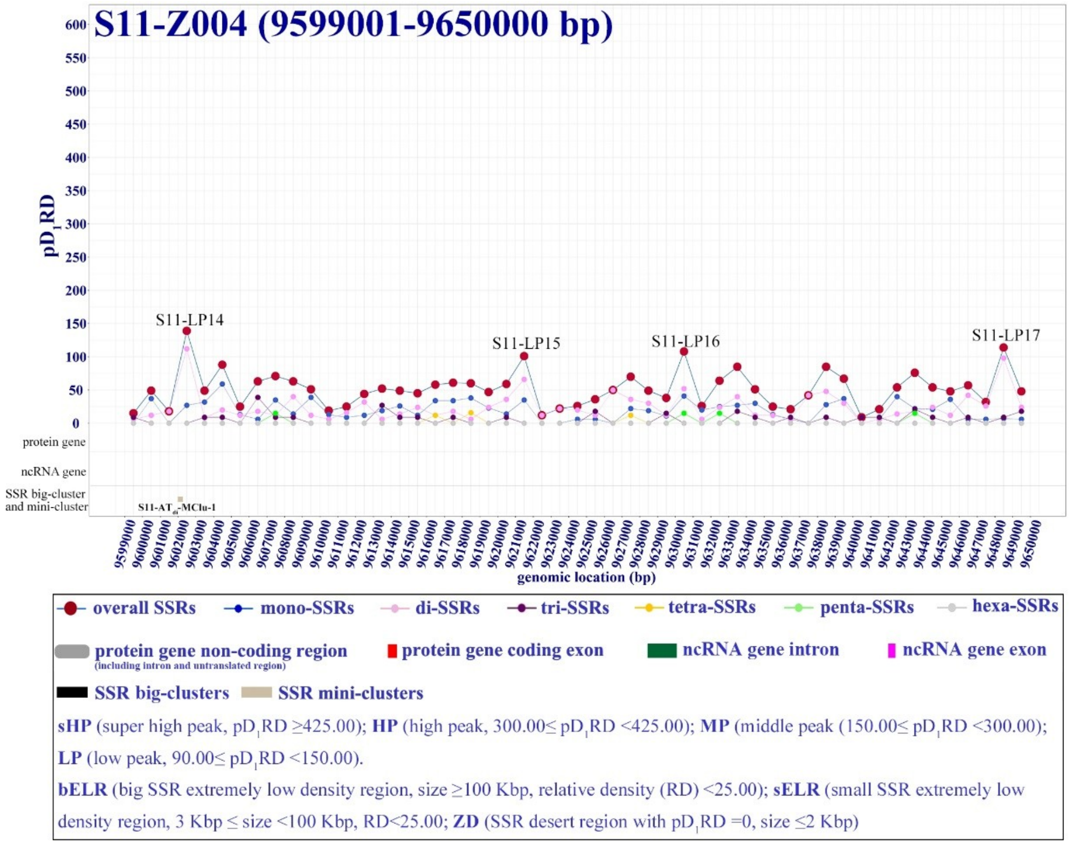

Supplementary Figure 1.187. The SSR position related D_1-relative density (pD_1RD) map of position at 9599001-9650000 bp of human reference Y-DNA (NC_000024.10) at resolution of 1 Kbp.

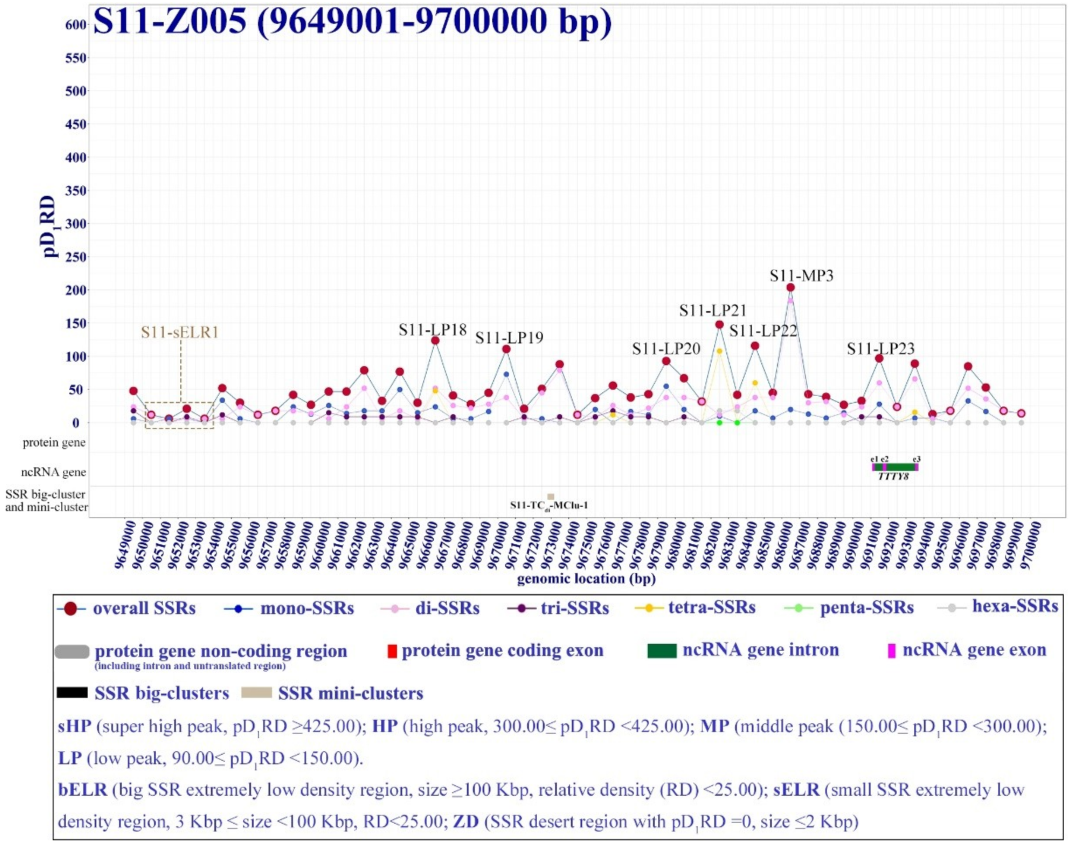

Supplementary Figure 1.188. The SSR position related D_1-relative density (pD_1RD) map of position at 9649001-9700000 bp of human reference Y-DNA (NC_000024.10) at resolution of 1 Kbp.

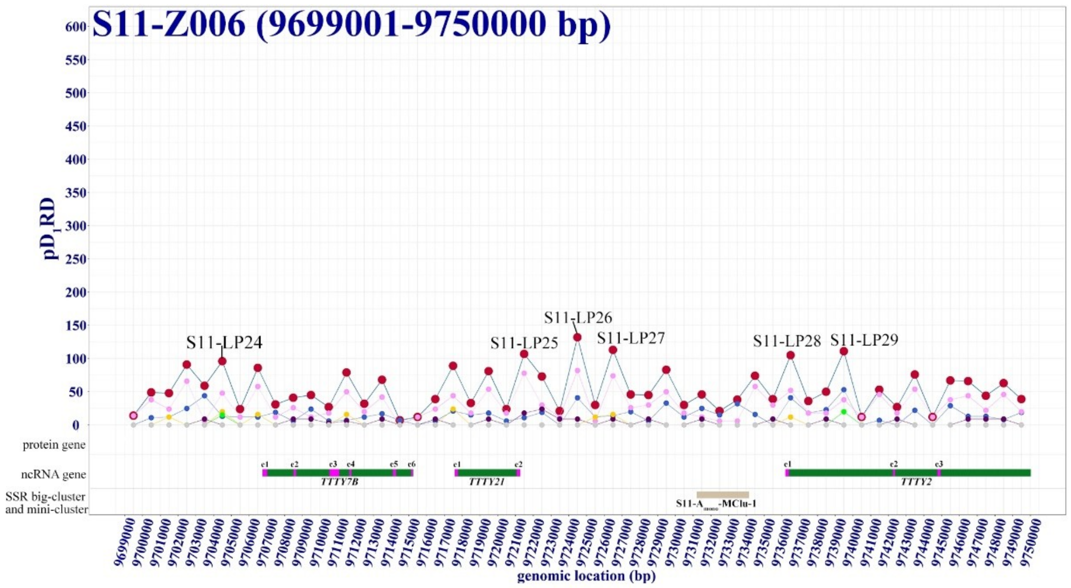

overall SSRs — mono-SSRs — di-SSRs — tri-SSRs — tetra-SSRs — penta-SSRs — hexa-SSRs

protein gene non-coding region (including intron and untranslated region) — protein gene coding exon — ncRNA gene intron — ncRNA gene exon

SSR big-clusters — SSR mini-clusters

sHP (super high peak, $pD_1RD \geq 425.00$); **HP** (high peak, $300.00 \leq pD_1RD < 425.00$); **MP** (middle peak ($150.00 \leq pD_1RD < 300.00$); **LP** (low peak, $90.00 \leq pD_1RD < 150.00$).

bELR (big SSR extremely low density region, size ≥100 Kbp, relative density (RD) <25.00); **sELR** (small SSR extremely low density region, 3 Kbp ≤ size <100 Kbp, RD<25.00; **ZD** (SSR desert region with $pD_1RD = 0$, size ≤2 Kbp)

Supplementary Figure 1.189. The SSR position related D_1-relative density (pD_1RD) map of position at 9699001-9750000 bp of human reference Y-DNA (NC_000024.10) at resolution of 1 Kbp.

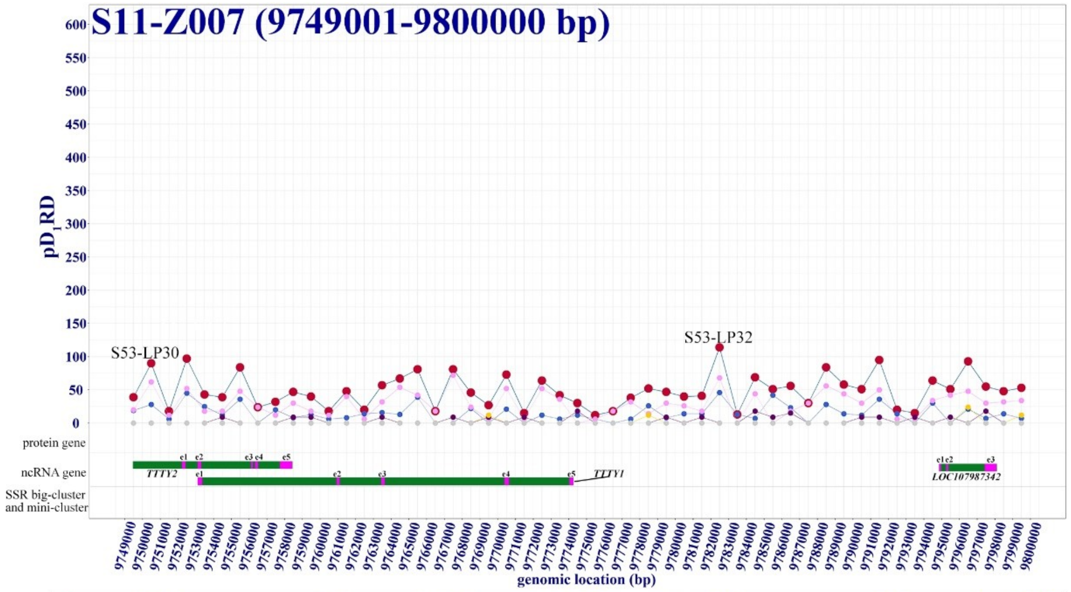

overall SSRs — mono-SSRs — di-SSRs — tri-SSRs — tetra-SSRs — penta-SSRs — hexa-SSRs

protein gene non-coding region (including intron and untranslated region) — protein gene coding exon — ncRNA gene intron — ncRNA gene exon

SSR big-clusters — SSR mini-clusters

sHP (super high peak, $pD_1RD \geq 425.00$); **HP** (high peak, $300.00 \leq pD_1RD < 425.00$); **MP** (middle peak ($150.00 \leq pD_1RD < 300.00$); **LP** (low peak, $90.00 \leq pD_1RD < 150.00$).

bELR (big SSR extremely low density region, size ≥100 Kbp, relative density (RD) <25.00); **sELR** (small SSR extremely low density region, 3 Kbp ≤ size <100 Kbp, RD<25.00; **ZD** (SSR desert region with $pD_1RD = 0$, size ≤2 Kbp)

Supplementary Figure 1.190. The SSR position related D_1-relative density (pD_1RD) map of position at 9749001-9800000 bp of human reference Y-DNA (NC_000024.10) at resolution of 1 Kbp.

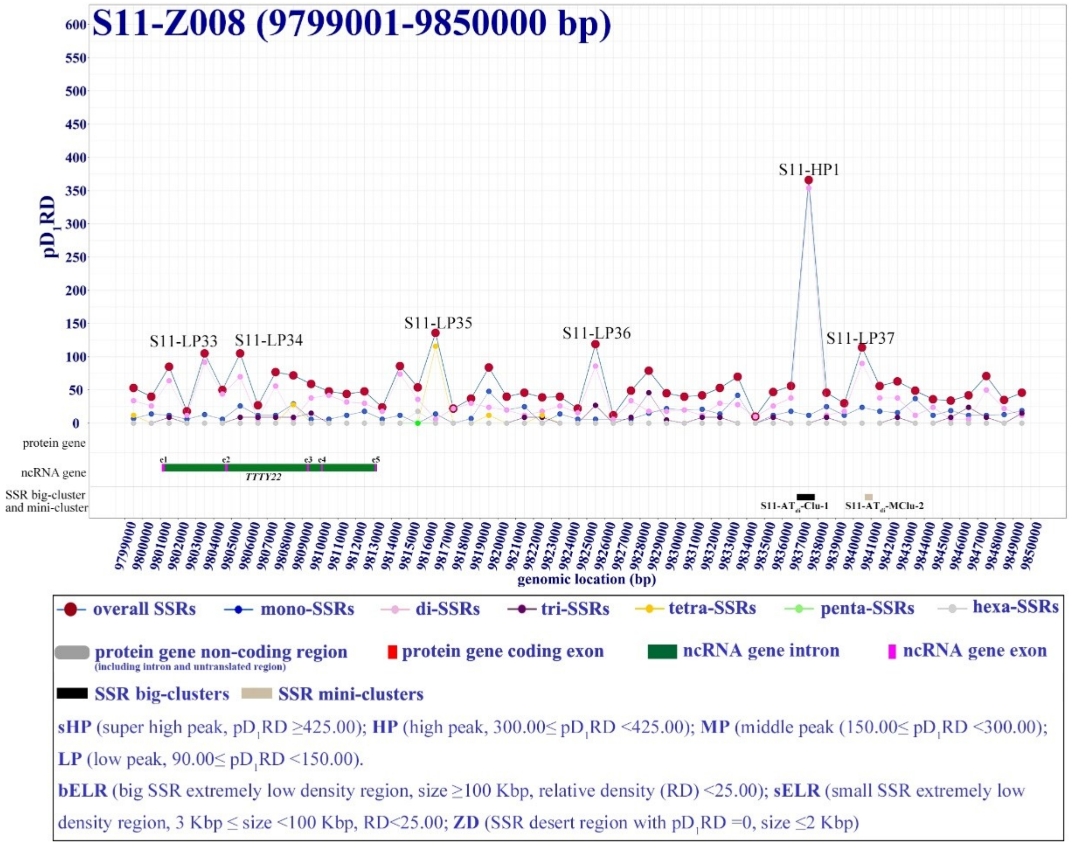

Supplementary Figure 1.191. The SSR position related D_1-relative density (pD_1RD) map of position at 9799001-9850000 bp of human reference Y-DNA (NC_000024.10) at resolution of 1 Kbp.

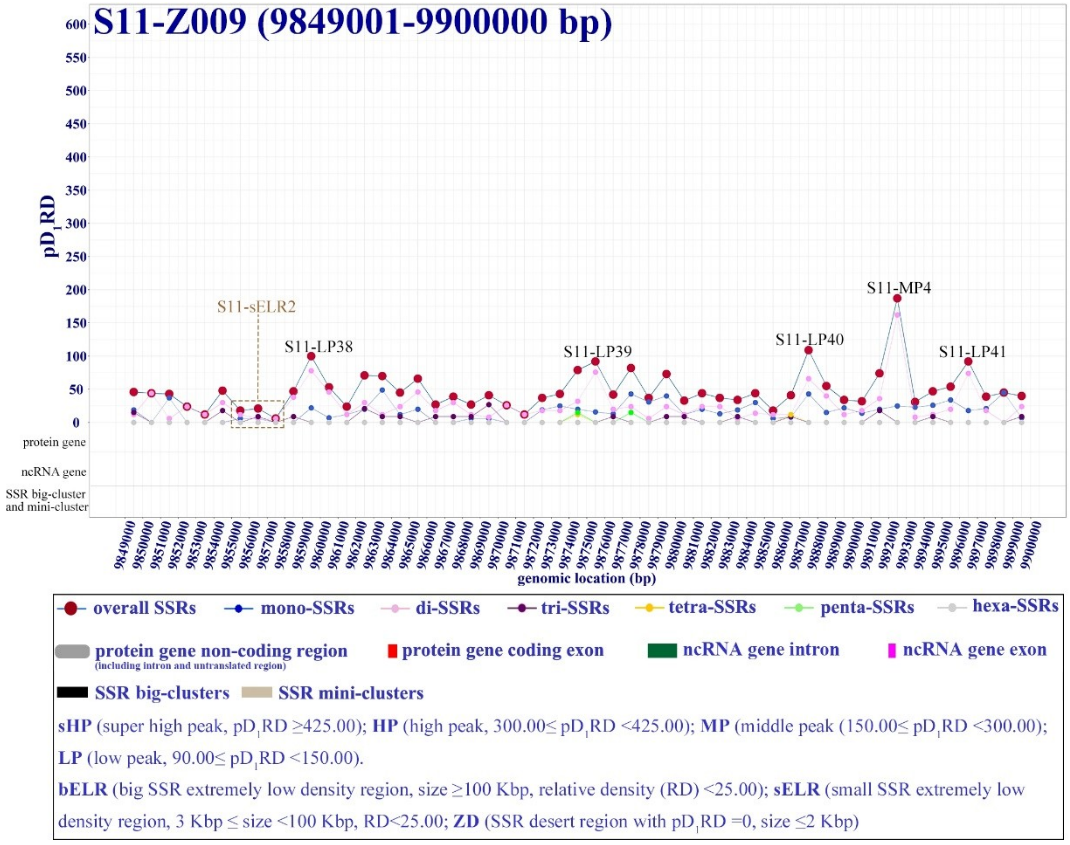

Supplementary Figure 1.192. The SSR position related D_1-relative density (pD_1RD) map of position at 9849001-9900000 bp of human reference Y-DNA (NC_000024.10) at resolution of 1 Kbp.

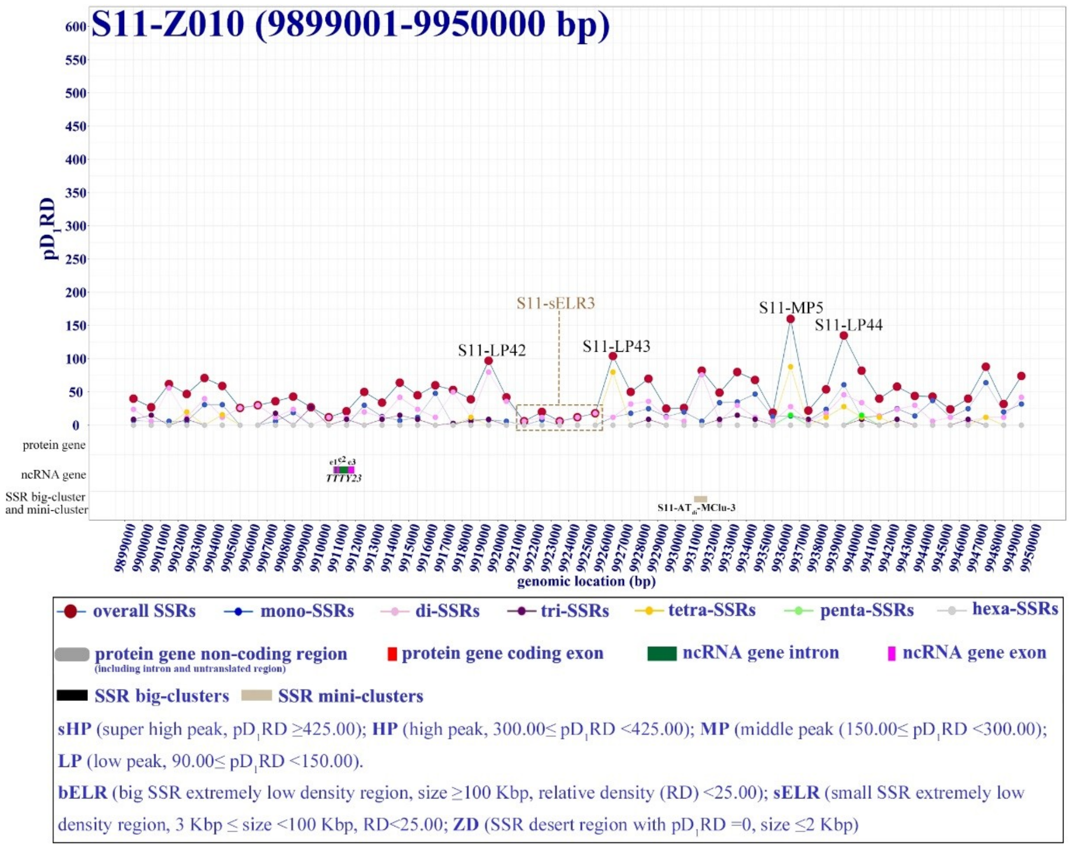

Supplementary Figure 1.193. The SSR position related D_1-relative density (pD_1RD) map of position at 9899001-9950000 bp of human reference Y-DNA (NC_000024.10) at resolution of 1 Kbp.

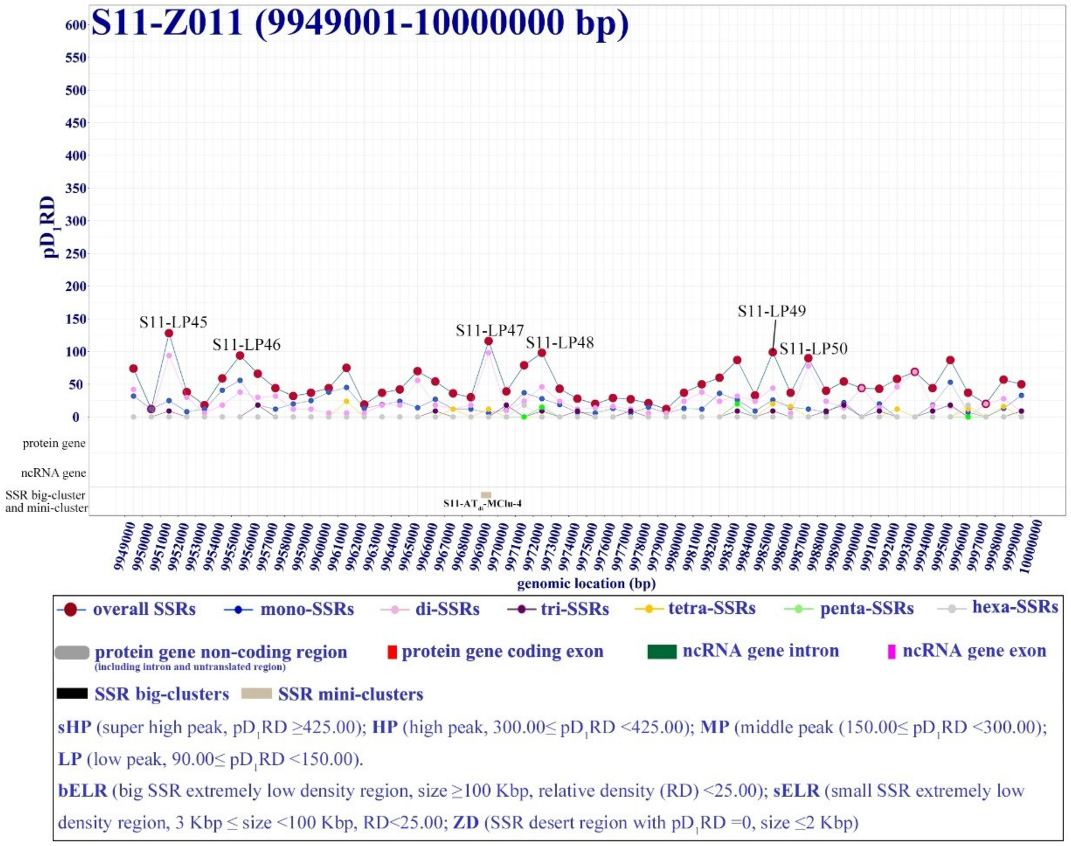

Supplementary Figure 1.194. The SSR position related D_1-relative density (pD_1RD) map of position at 9949001-10000000 bp of human reference Y-DNA (NC_000024.10) at resolution of 1 Kbp.

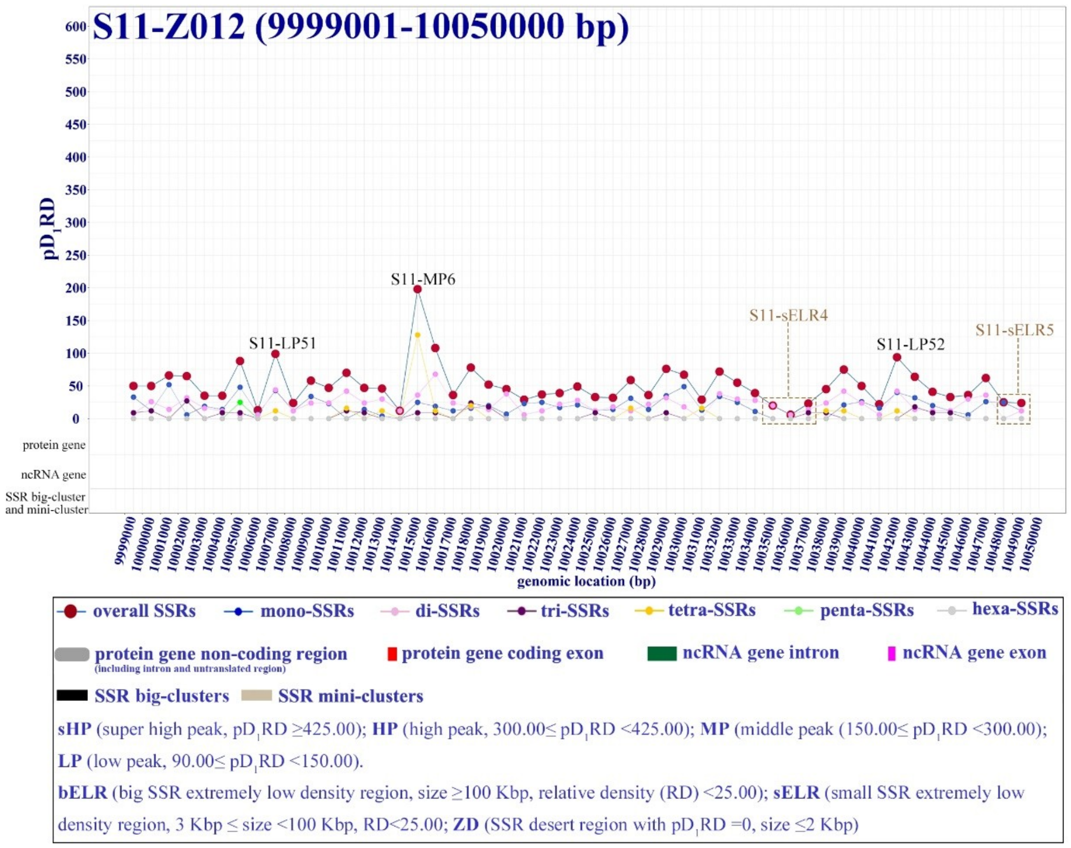

Supplementary Figure 1.195. The SSR position related D_1-relative density (pD_1RD) map of position at 9999001-10050000 bp of human reference Y-DNA (NC_000024.10) at resolution of 1 Kbp.

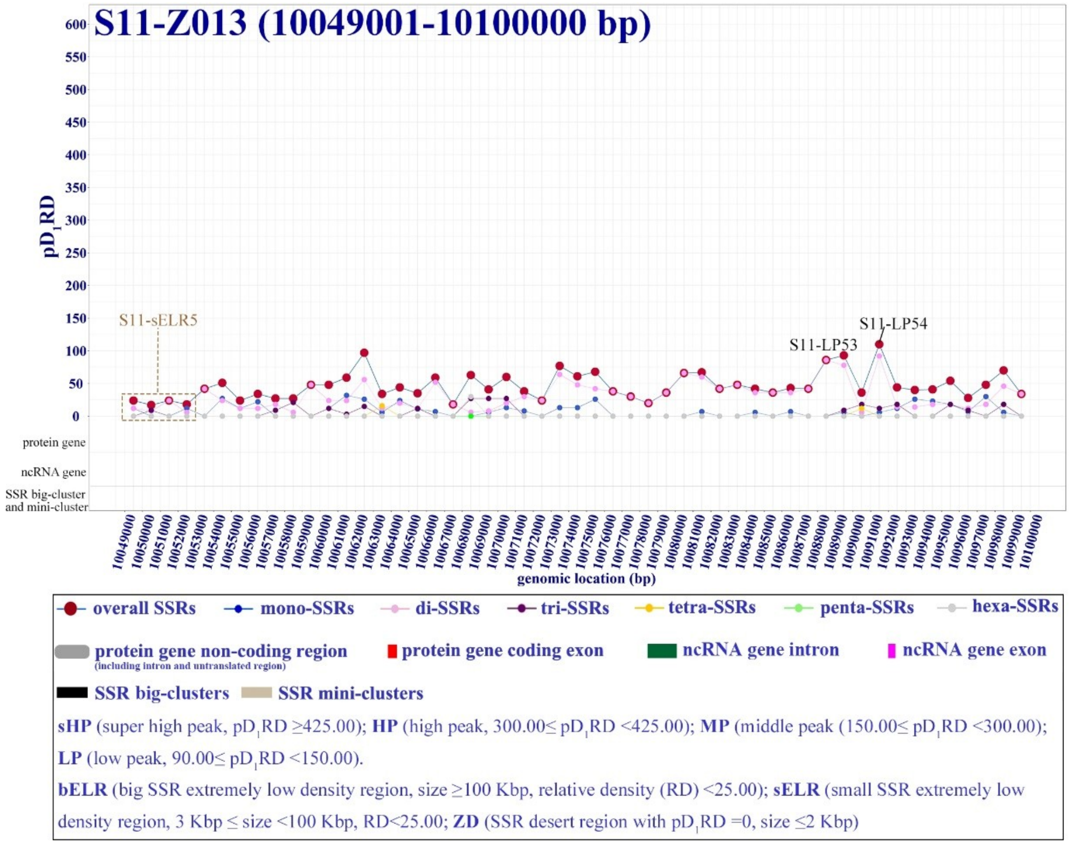

Supplementary Figure 1.196. The SSR position related D_1-relative density (pD_1RD) map of position at 10049001-10100000 bp of human reference Y-DNA (NC_000024.10) at resolution of 1 Kbp.

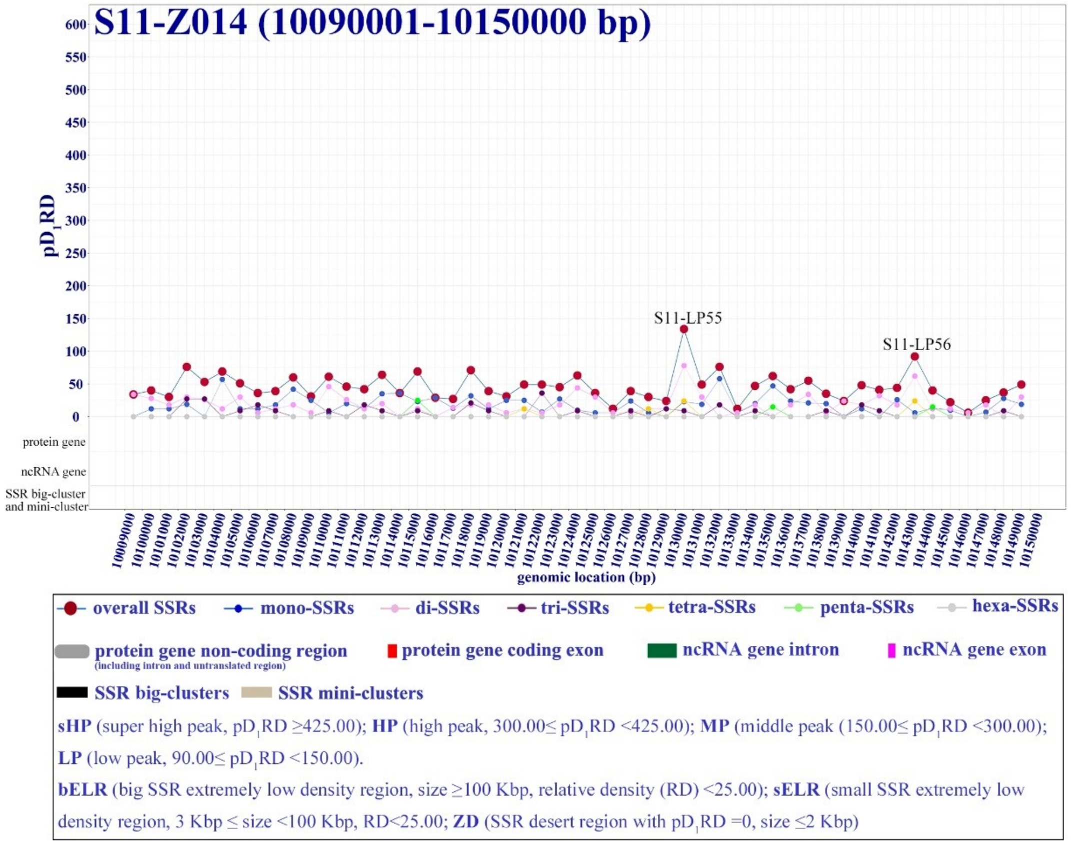

Supplementary Figure 1.197. The SSR position related D_1-relative density (pD_1RD) map of position at 10099001-10150000 bp of human reference Y-DNA (NC_000024.10) at resolution of 1 Kbp.

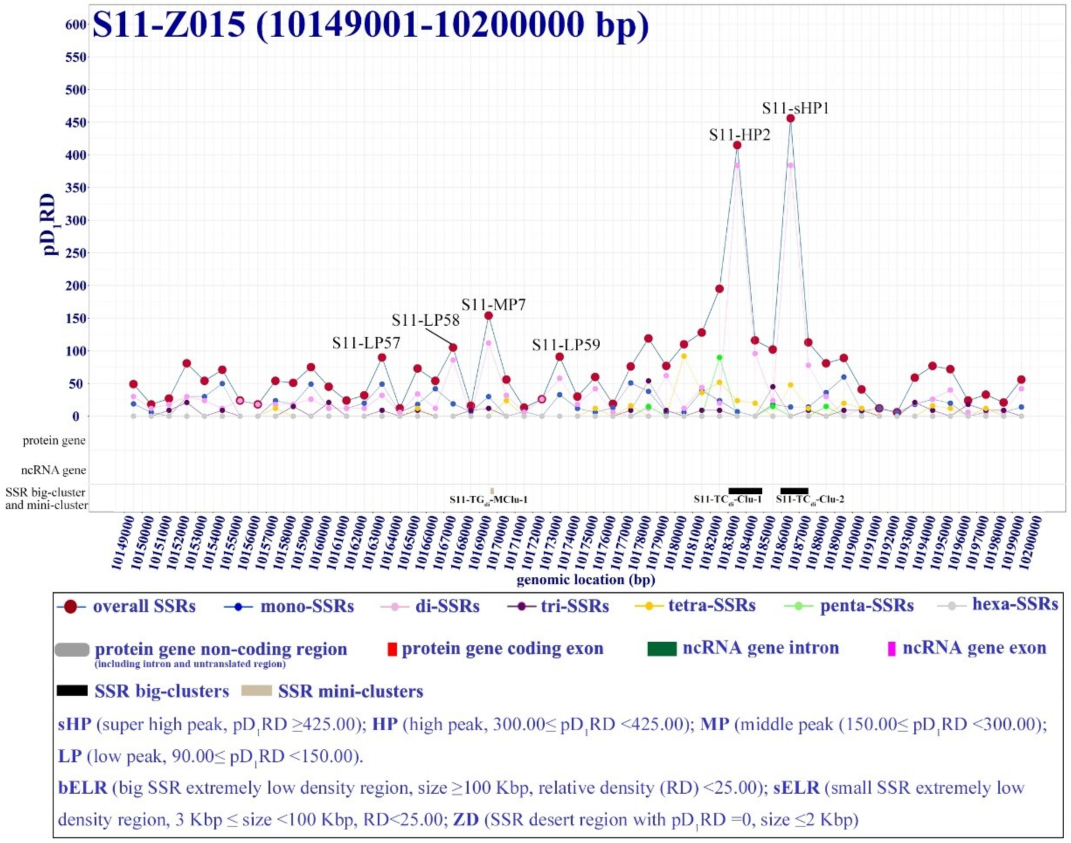

Supplementary Figure 1.198. The SSR position related D_1-relative density (pD_1RD) map of position at 10149001-10200000 bp of human reference Y-DNA (NC_000024.10) at resolution of 1 Kbp.

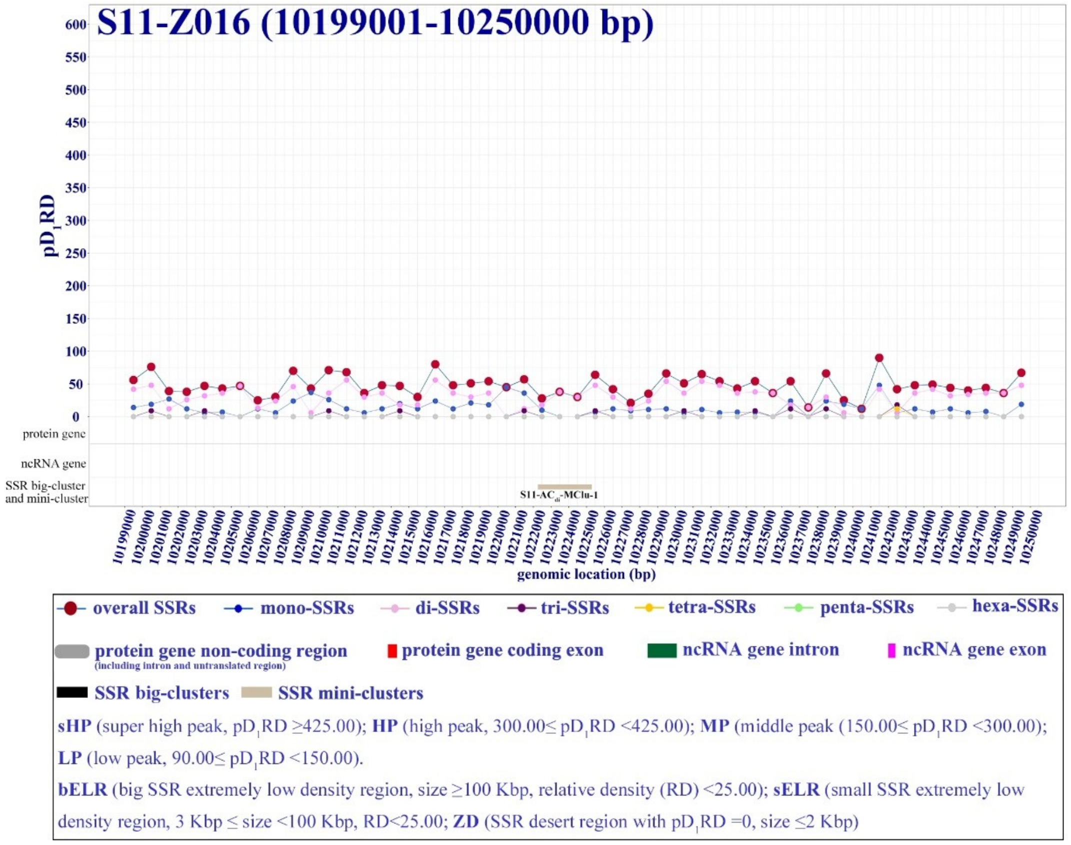

Supplementary Figure 1.199. The SSR position related D_1-relative density (pD_1RD) map of position at 10199001-10250000 bp of human reference Y-DNA (NC_000024.10) at resolution of 1 Kbp.

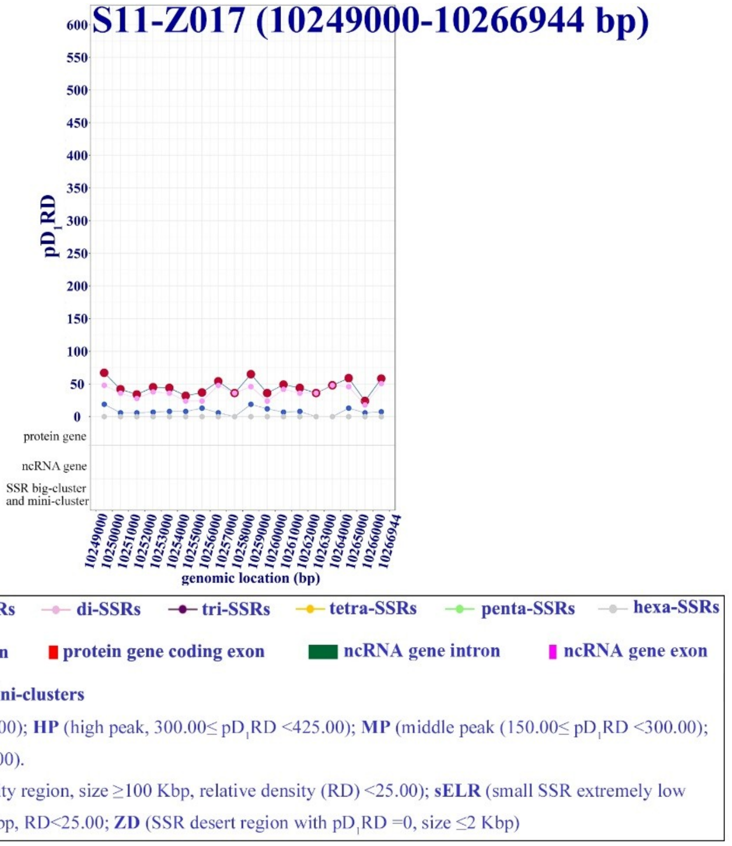

Supplementary Figure 1.200. The SSR position related D_1-relative density (pD_1RD) map of position at 10249001-10266944 bp (unnormal zone <51000 bp) of human reference Y-DNA (NC_000024.10) at resolution of 1 Kbp.

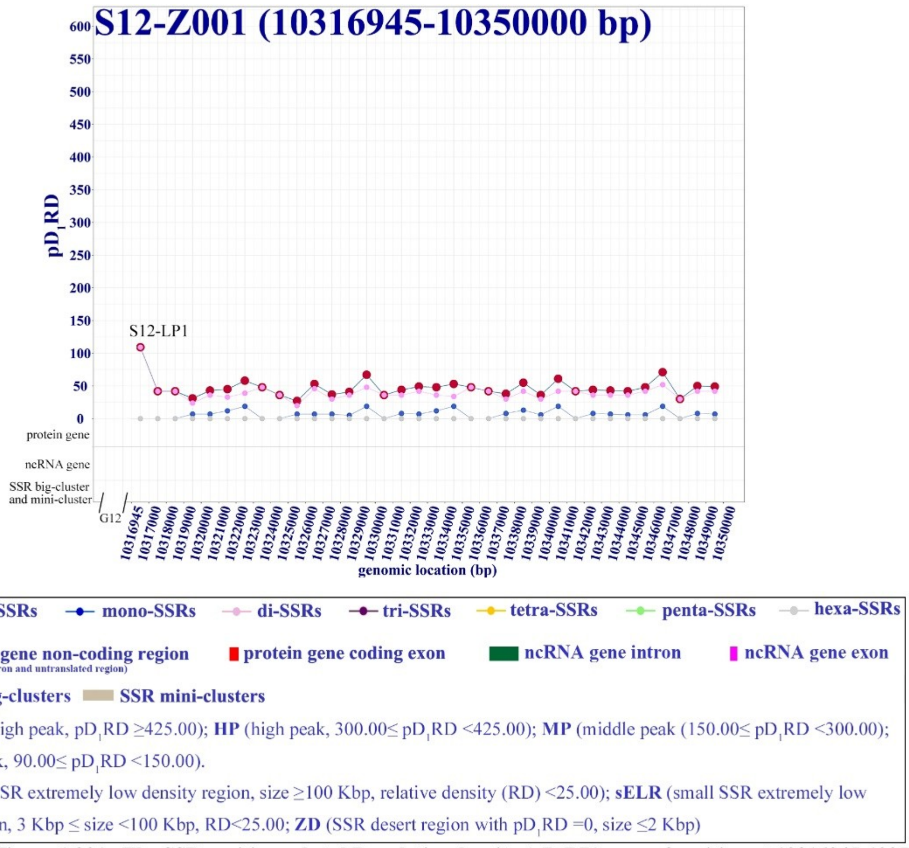

Supplementary Figure 1.201. The SSR position related D_1-relative density (pD_1RD) map of position at 10316945-10350000 bp (unnormal zone<51000 bp) of human reference Y-DNA (NC_000024.10) at resolution of 1 Kbp.

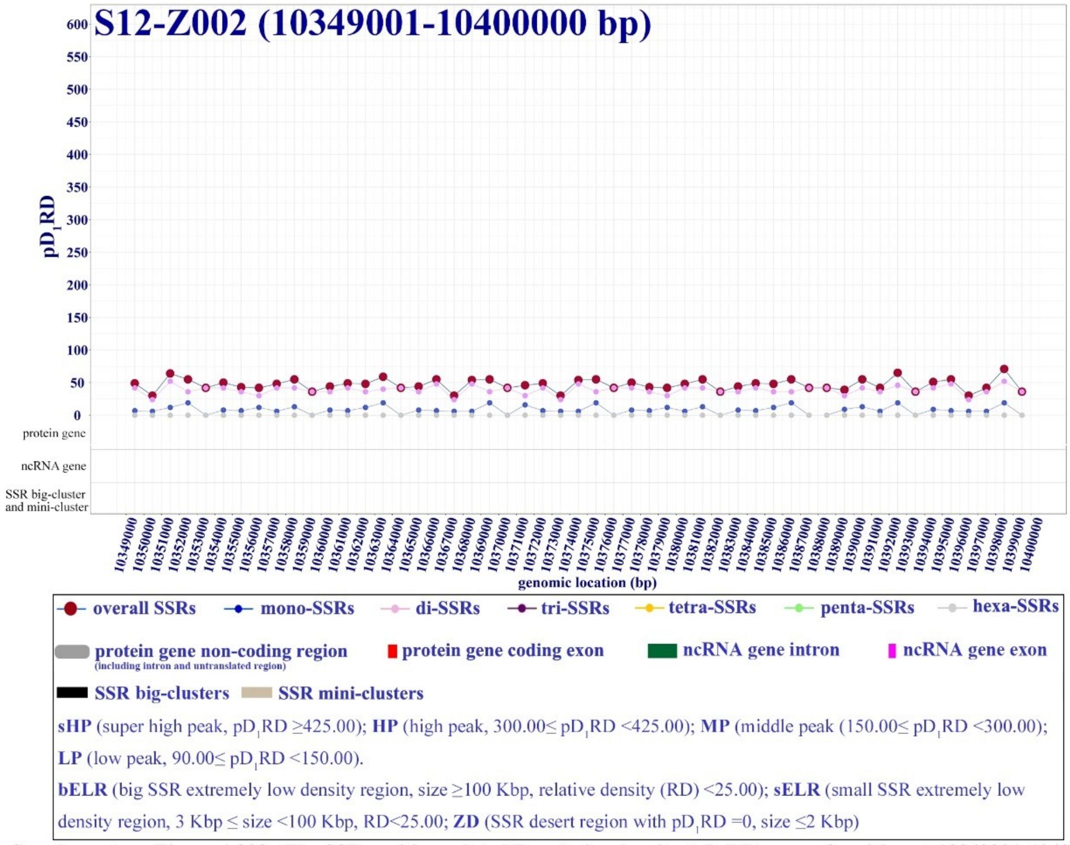

Supplementary Figure 1.202. The SSR position related D_1-relative density (pD_1RD) map of position at 10349001-10400000 bp of human reference Y-DNA (NC_000024.10) at resolution of 1 Kbp.

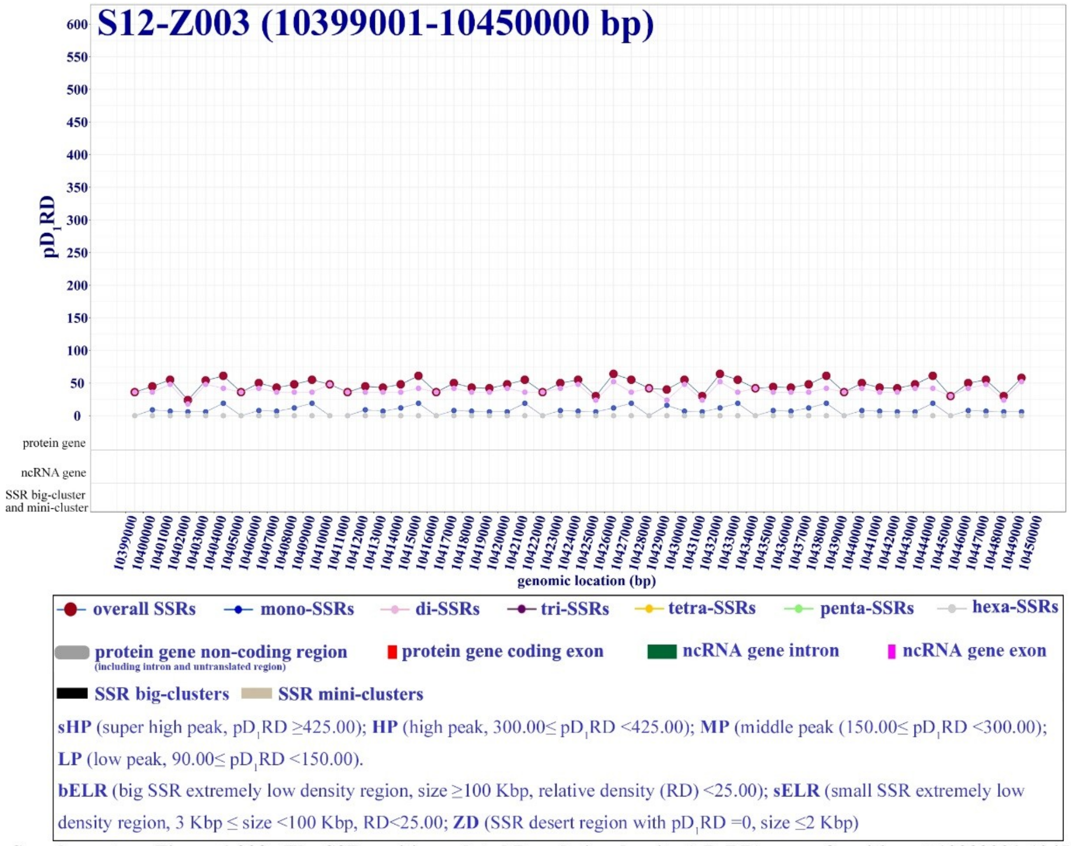

Supplementary Figure 1.203. The SSR position related D_1-relative density (pD_1RD) map of position at 10399001-10450000 bp of human reference Y-DNA (NC_000024.10) at resolution of 1 Kbp.

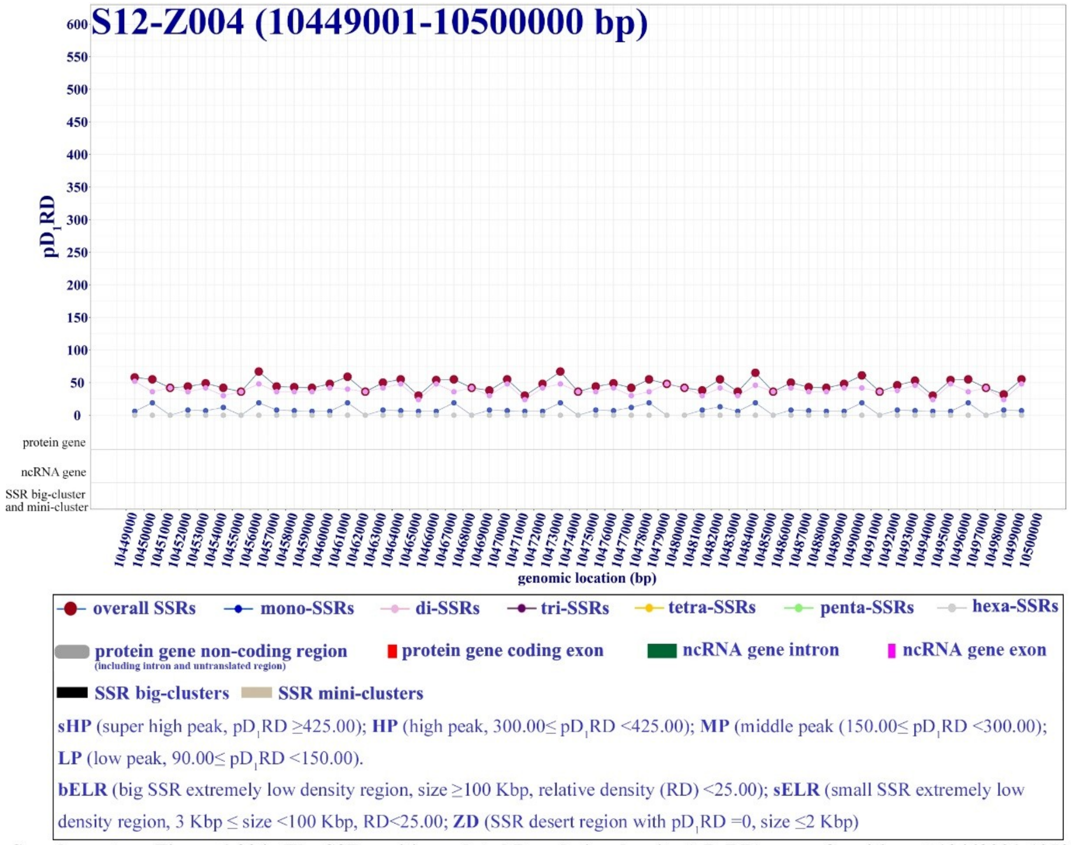

Supplementary Figure 1.204. The SSR position related D_1-relative density (pD_1RD) map of position at 10449001-10500000 bp of human reference Y-DNA (NC_000024.10) at resolution of 1 Kbp.

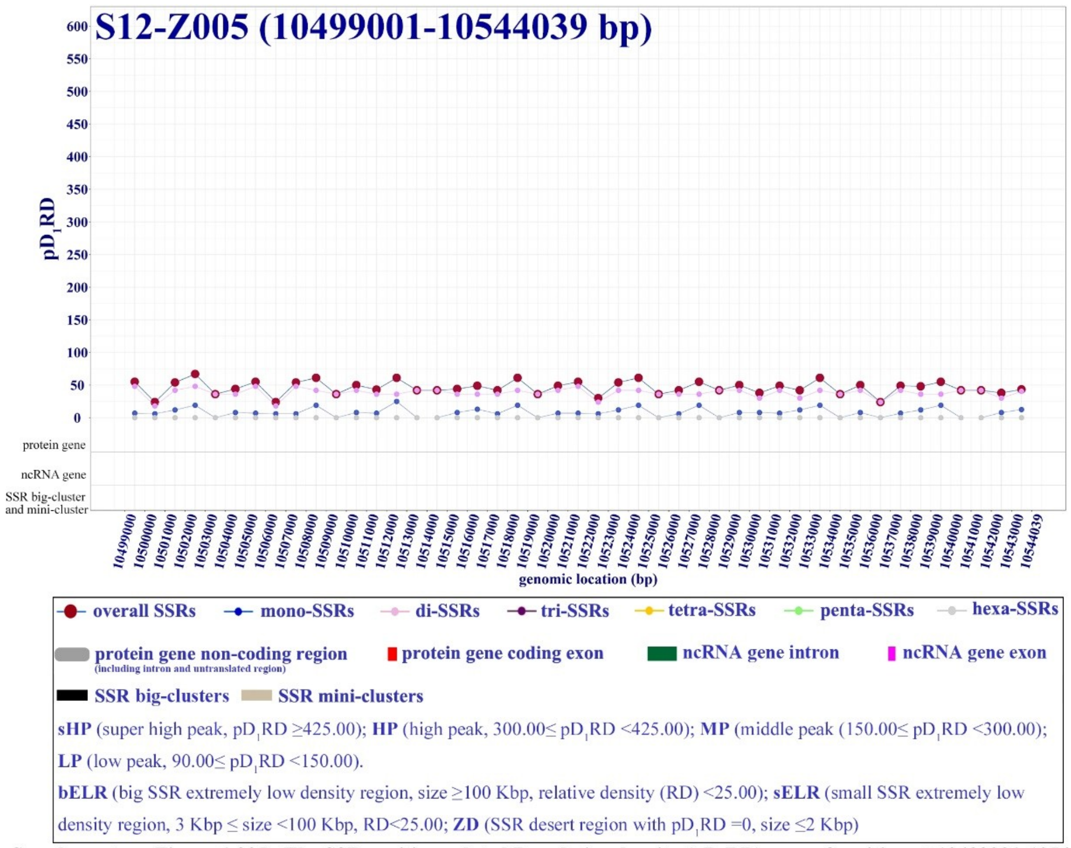

Supplementary Figure 1.205. The SSR position related D_1-relative density (pD_1RD) map of position at 10499001-10544039 bp of human reference Y-DNA (NC_000024.10) at resolution of 1 Kbp.

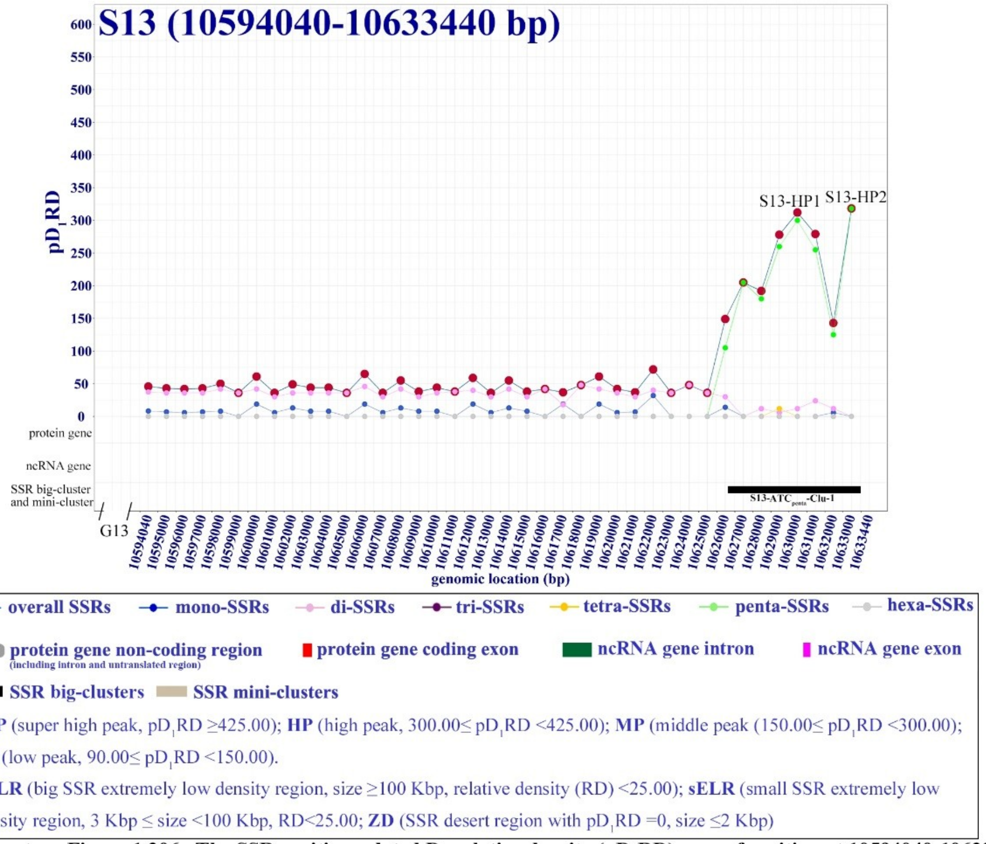

Supplementary Figure 1.206. The SSR position related D_1-relative density (pD_1RD) map of position at 10594040-10633440 bp (unnormal zone<51000 bp) of human reference Y-DNA (NC_000024.10) at resolution of 1 Kbp.

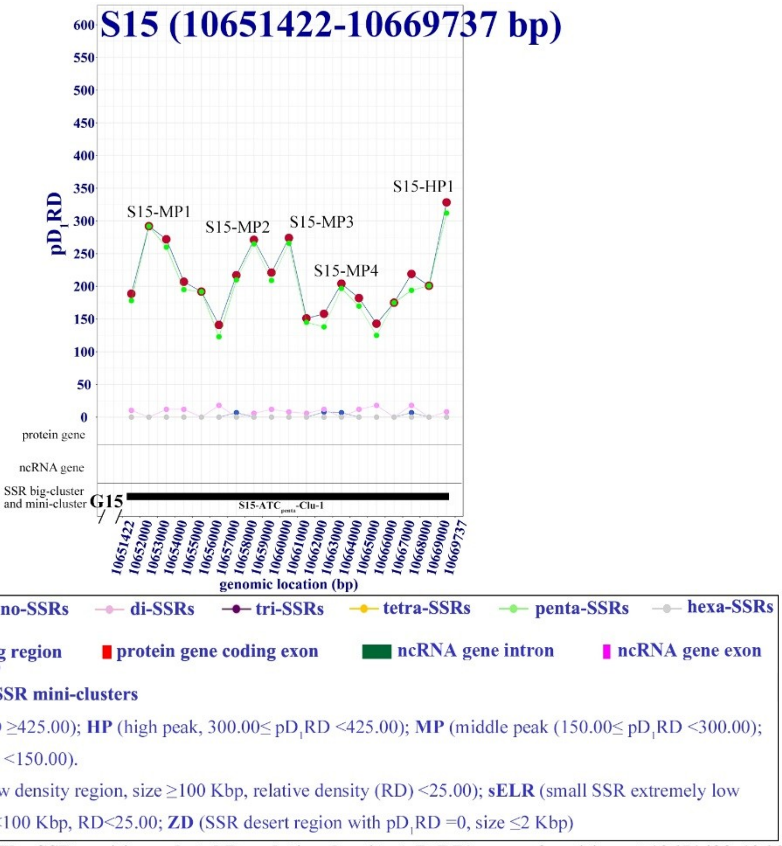

Supplementary Figure 1.207. The SSR position related D_1-relative density (pD_1RD) map of position at 10651422-10669737 bp (unnormal zone<51000 bp) of human reference Y-DNA (NC_000024.10) at resolution of 1 Kbp.

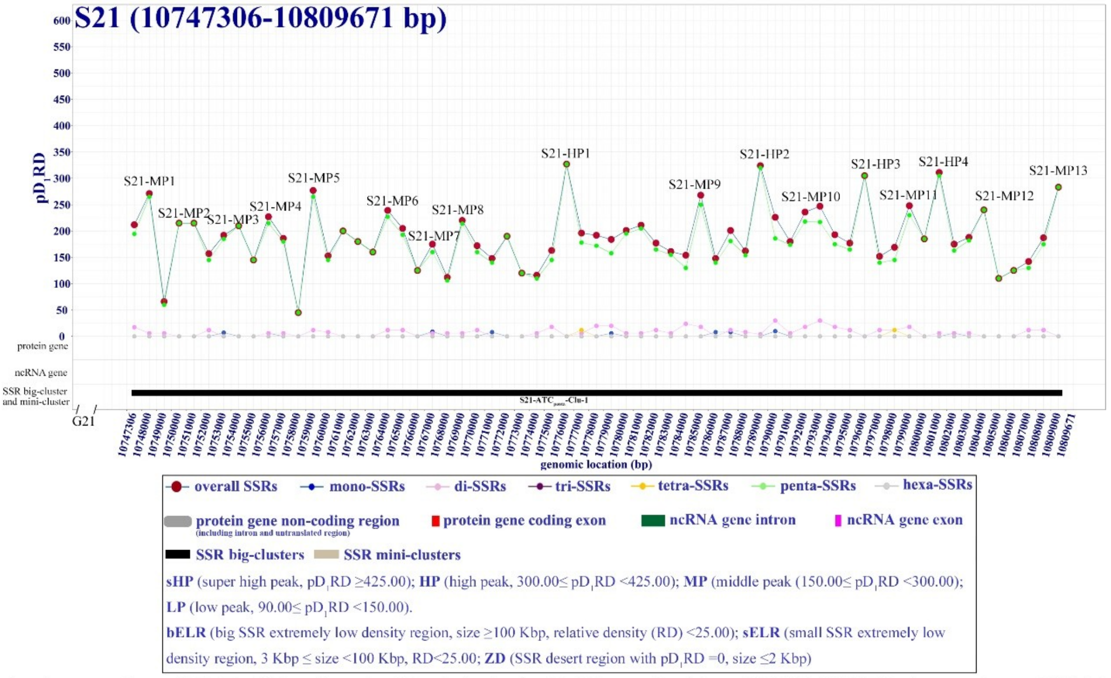

Supplementary Figure 1.208. The SSR position related D_1-relative density (pD_1RD) map of position at 10747306-10809671 bp (unnormal zone >51000 bp) of human reference Y-DNA (NC_000024.10) at resolution of 1 Kbp.

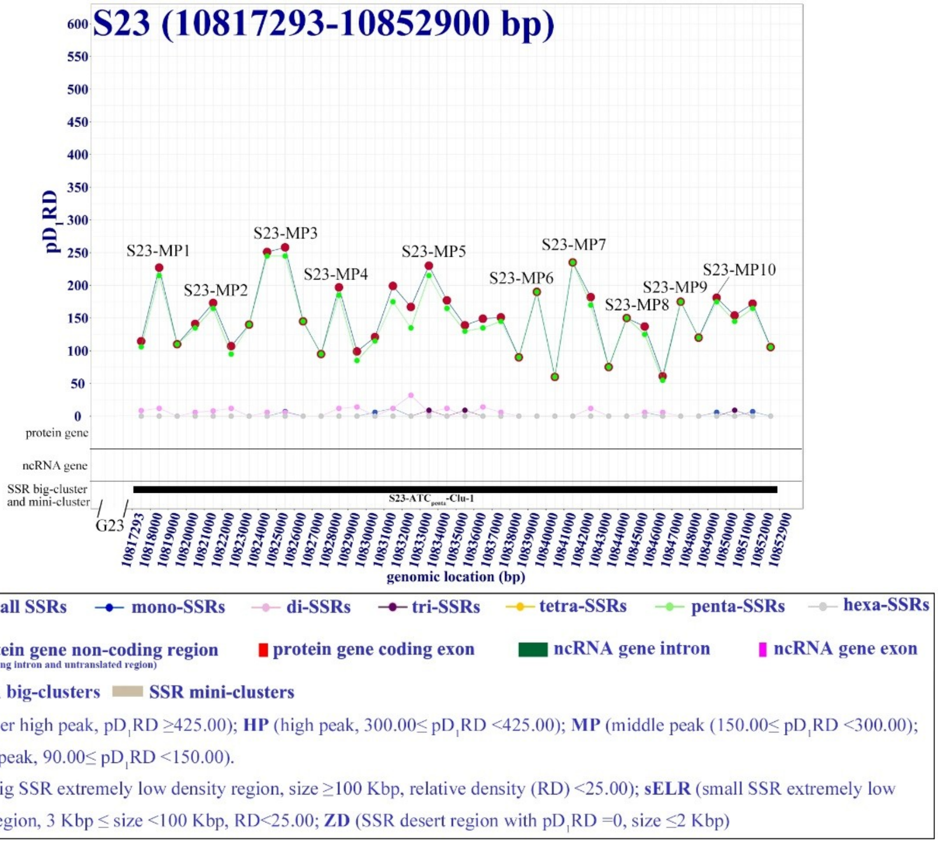

Supplementary Figure 1.209. The SSR position related D_1-relative density (pD_1RD) map of position at 10817293-10852900 bp (unnormal zone <51000 bp) of human reference Y-DNA (NC_000024.10) at resolution of 1 Kbp.

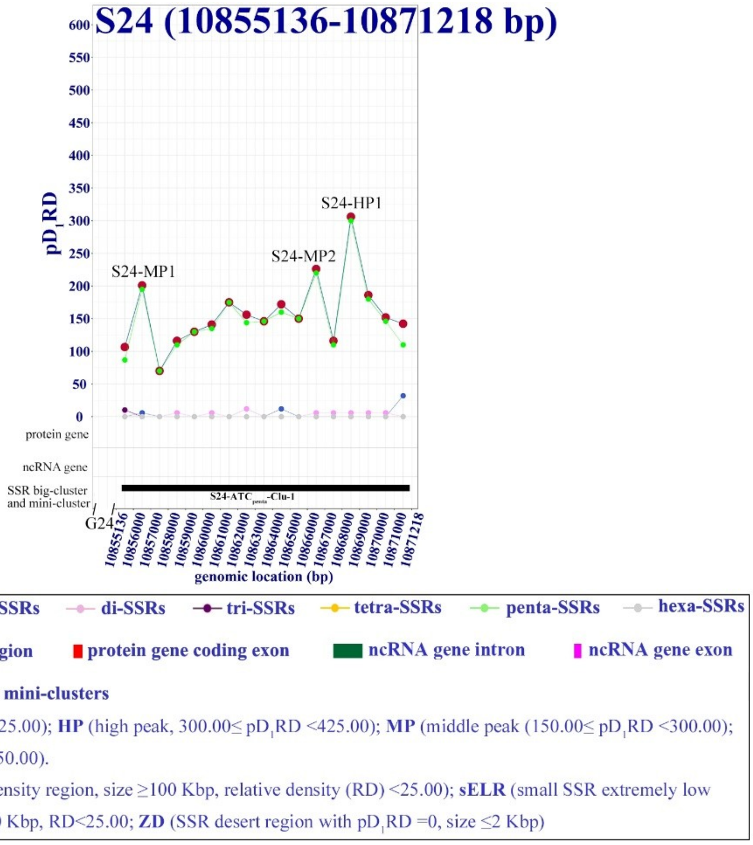

Supplementary Figure 1.210. The SSR position related D_1-relative density (pD_1RD) map of position at 10855136-10871218 bp (unnormal zone <51000 bp) of human reference Y-DNA (NC_000024.10) at resolution of 1 Kbp.

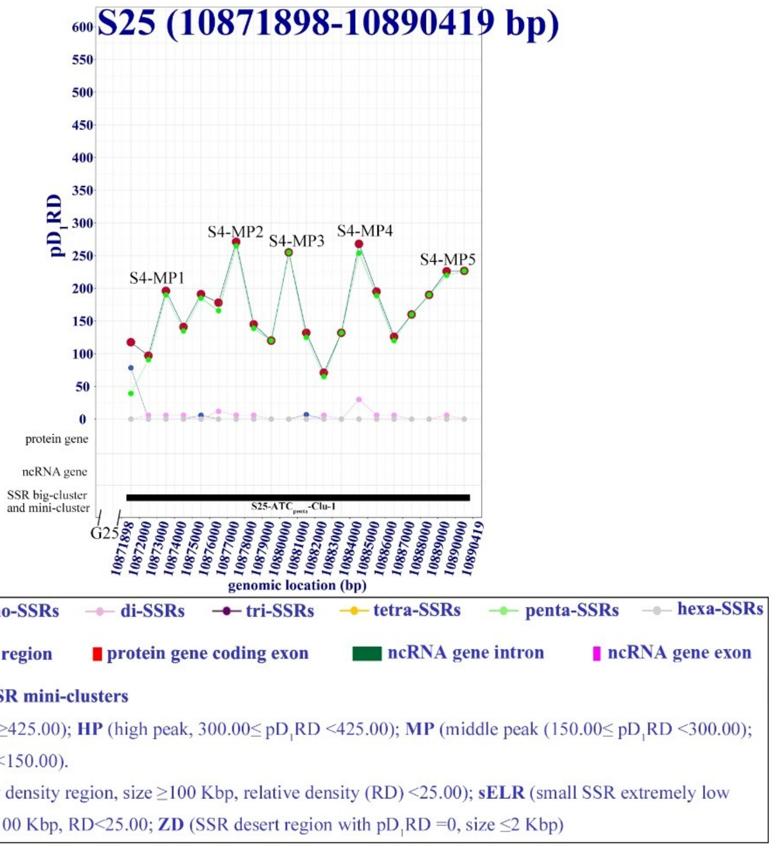

Supplementary Figure 1.211. **The SSR position related D_1-relative density (pD_1RD) map of position at 10871898-10890419 bp (unnormal zone <51000 bp) of human reference Y-DNA (NC_000024.10) at resolution of 1 Kbp.**

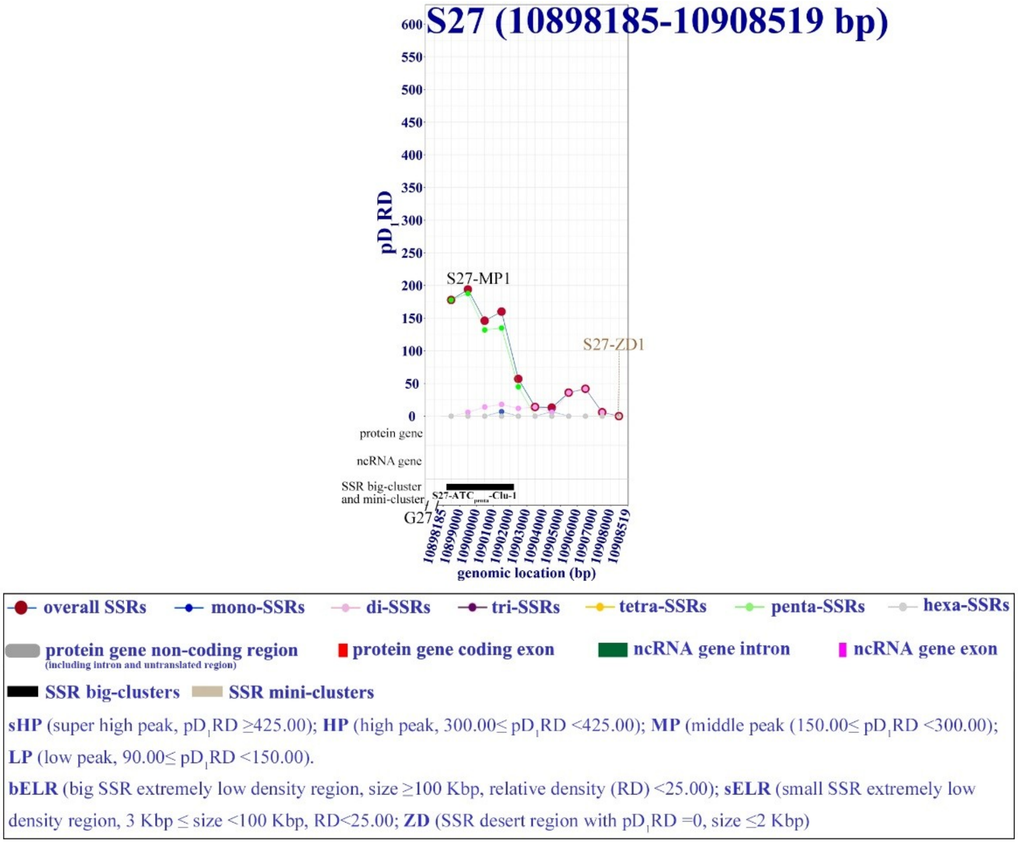

Supplementary Figure 1.212. **The SSR position related D_1-relative density (pD_1RD) map of position at 10898185-10908519 bp (unnormal zone <51000 bp) of human reference Y-DNA (NC_000024.10) at resolution of 1 Kbp.**

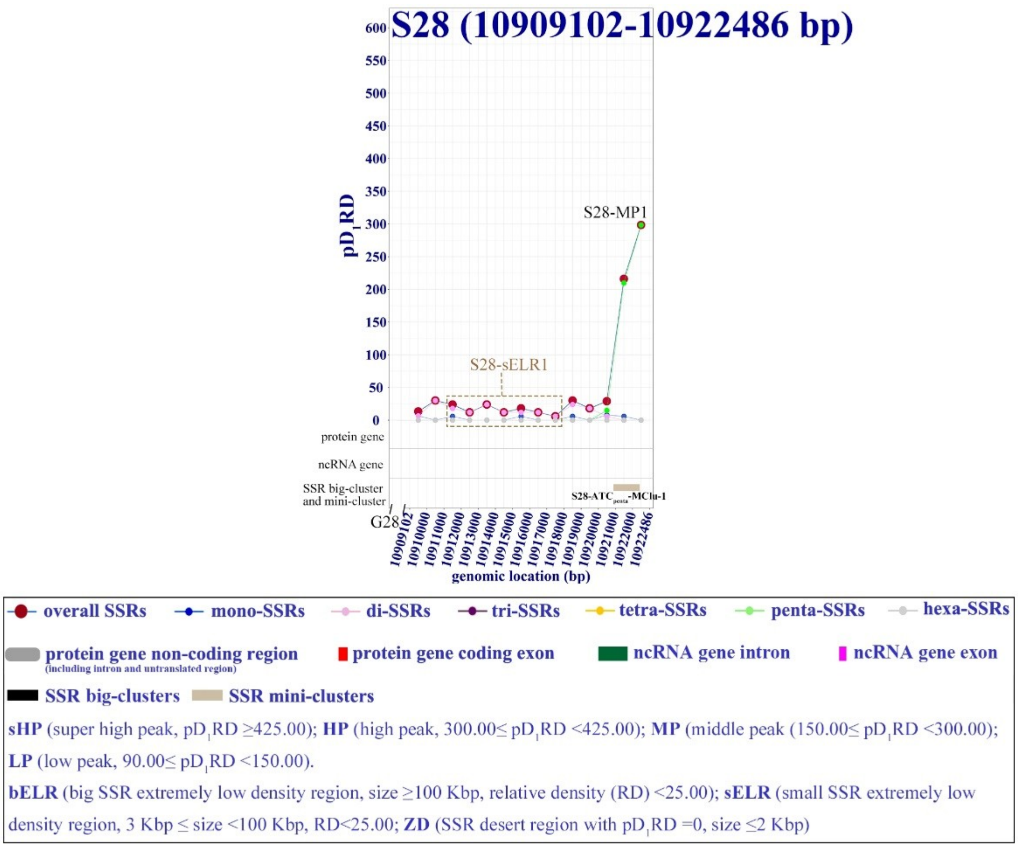

Supplementary Figure 1.213. The SSR position related D_1-relative density (pD_1RD) map of position at 10909102-10922486 bp (unnormal zone <51000 bp) of human reference Y-DNA (NC_000024.10) at resolution of 1 Kbp.

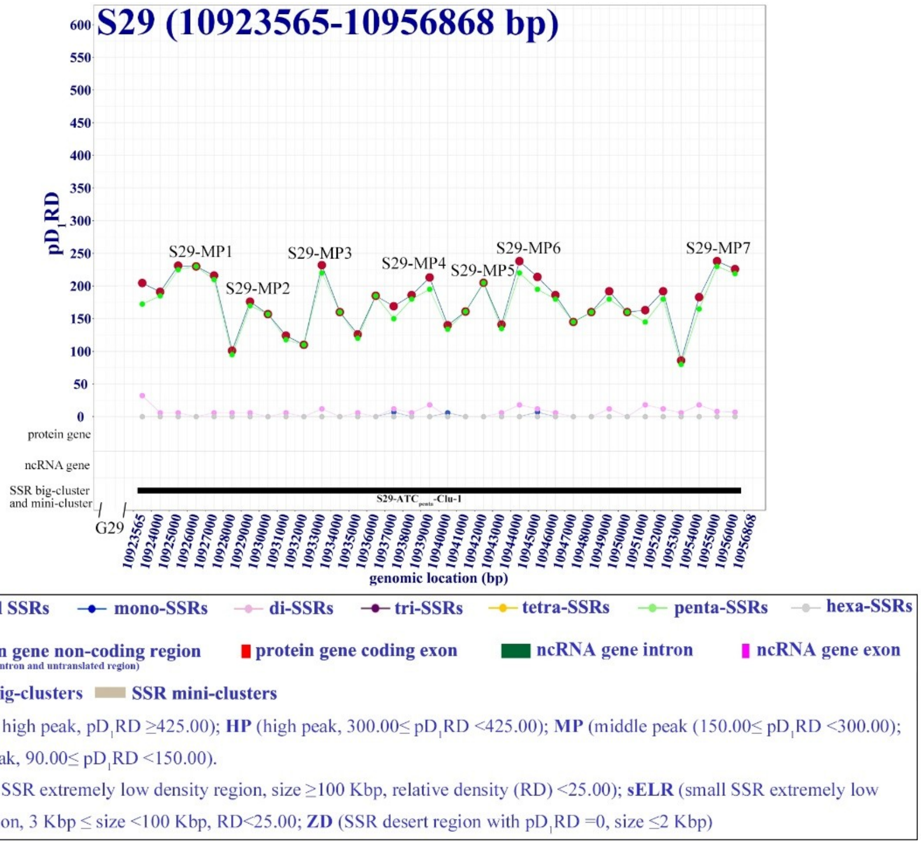

Supplementary Figure 1.214. The SSR position related D_1-relative density (pD_1RD) map of position at 10923565-10956868 bp (unnormal zone <51000 bp) of human reference Y-DNA (NC_000024.10) at resolution of 1 Kbp.

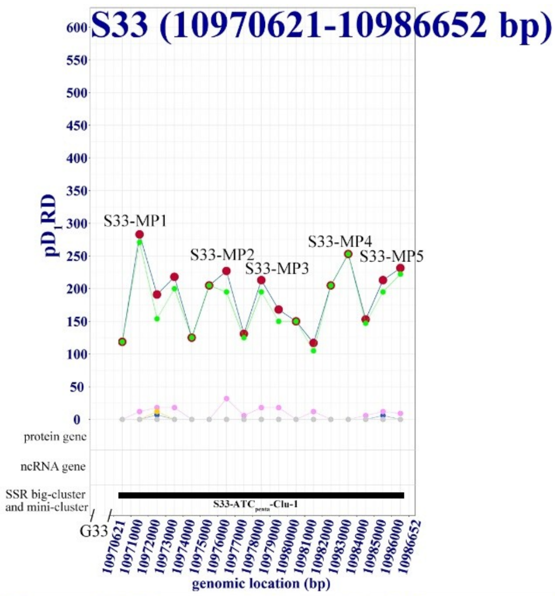

overall SSRs **mono-SSRs** **di-SSRs** **tri-SSRs** **tetra-SSRs** **penta-SSRs** **hexa-SSRs**

protein gene non-coding region (including intron and untranslated region) **protein gene coding exon** **ncRNA gene intron** **ncRNA gene exon**

SSR big-clusters **SSR mini-clusters**

sHP (super high peak, $pD_1RD \geq 425.00$); **HP** (high peak, $300.00 \leq pD_1RD < 425.00$); **MP** (middle peak ($150.00 \leq pD_1RD < 300.00$); **LP** (low peak, $90.00 \leq pD_1RD < 150.00$).

bELR (big SSR extremely low density region, size ≥100 Kbp, relative density (RD) <25.00); **sELR** (small SSR extremely low density region, 3 Kbp ≤ size <100 Kbp, RD<25.00; **ZD** (SSR desert region with $pD_1RD = 0$, size ≤2 Kbp)

Supplementary Figure 1.215. The SSR position related D_1-relative density (pD_1RD) map of position at 10970621-10986652 bp (unnormal zone <51000 bp) of human reference Y-DNA (NC_000024.10) at resolution of 1 Kbp.

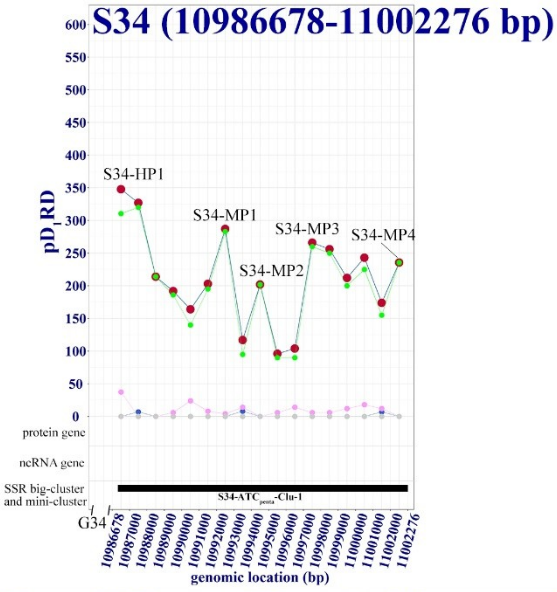

overall SSRs **mono-SSRs** **di-SSRs** **tri-SSRs** **tetra-SSRs** **penta-SSRs** **hexa-SSRs**

protein gene non-coding region (including intron and untranslated region) **protein gene coding exon** **ncRNA gene intron** **ncRNA gene exon**

SSR big-clusters **SSR mini-clusters**

sHP (super high peak, $pD_1RD \geq 425.00$); **HP** (high peak, $300.00 \leq pD_1RD < 425.00$); **MP** (middle peak ($150.00 \leq pD_1RD < 300.00$); **LP** (low peak, $90.00 \leq pD_1RD < 150.00$).

bELR (big SSR extremely low density region, size ≥100 Kbp, relative density (RD) <25.00); **sELR** (small SSR extremely low density region, 3 Kbp ≤ size <100 Kbp, RD<25.00; **ZD** (SSR desert region with $pD_1RD = 0$, size ≤2 Kbp)

Supplementary Figure 1.216. The SSR position related D_1-relative density (pD_1RD) map of position at 10986678-11002276 bp (unnormal zone <51000 bp) of human reference Y-DNA (NC_000024.10) at resolution of 1 Kbp.

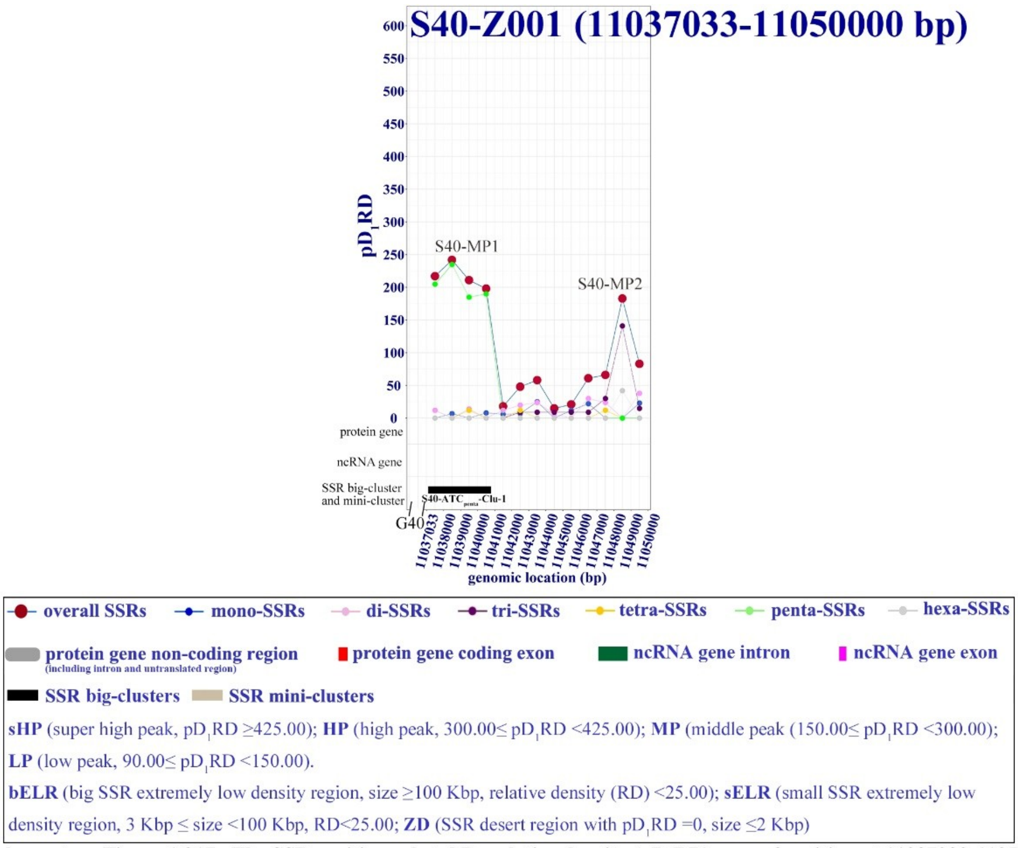

Supplementary Figure 1.217. The SSR position related D_1-relative density (pD_1RD) map of position at 11037033-11050000 bp (unnormal zone <51000 bp) of human reference Y-DNA (NC_000024.10) at resolution of 1 Kbp.

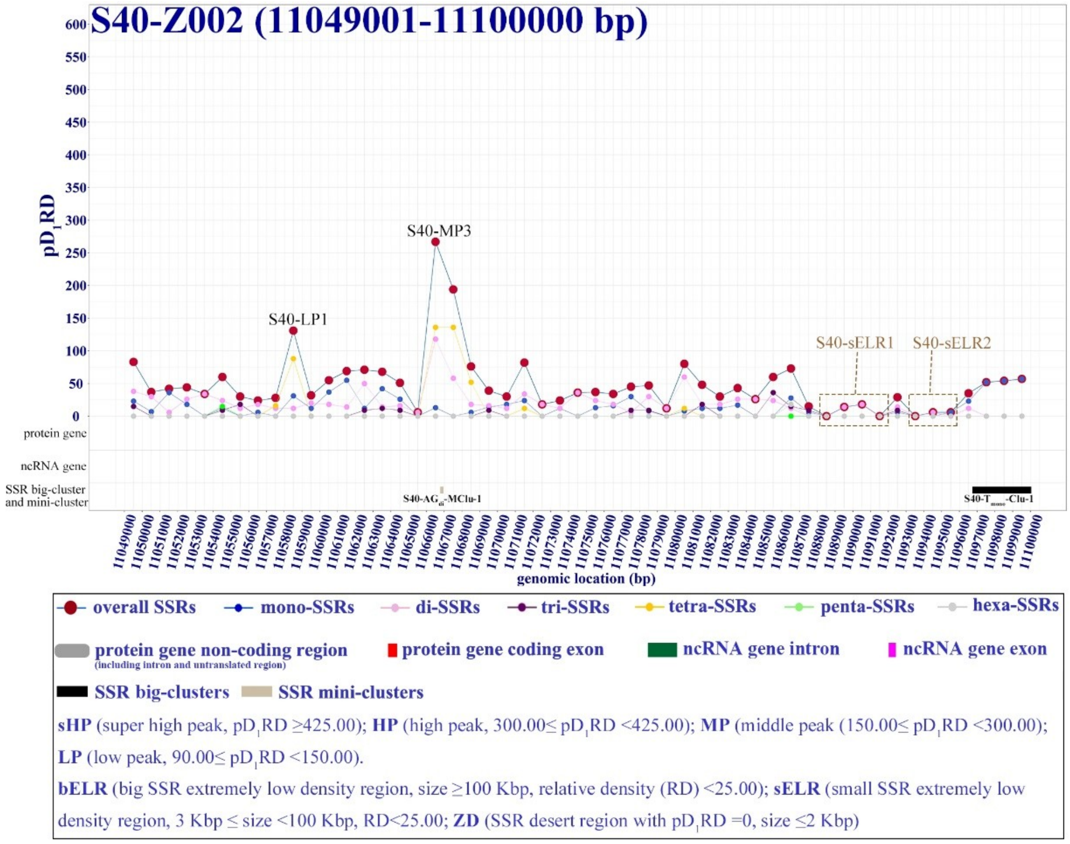

Supplementary Figure 1.218. The SSR position related D_1-relative density (pD_1RD) map of position at 11049001-11100000 bp of human reference Y-DNA (NC_000024.10) at resolution of 1 Kbp.

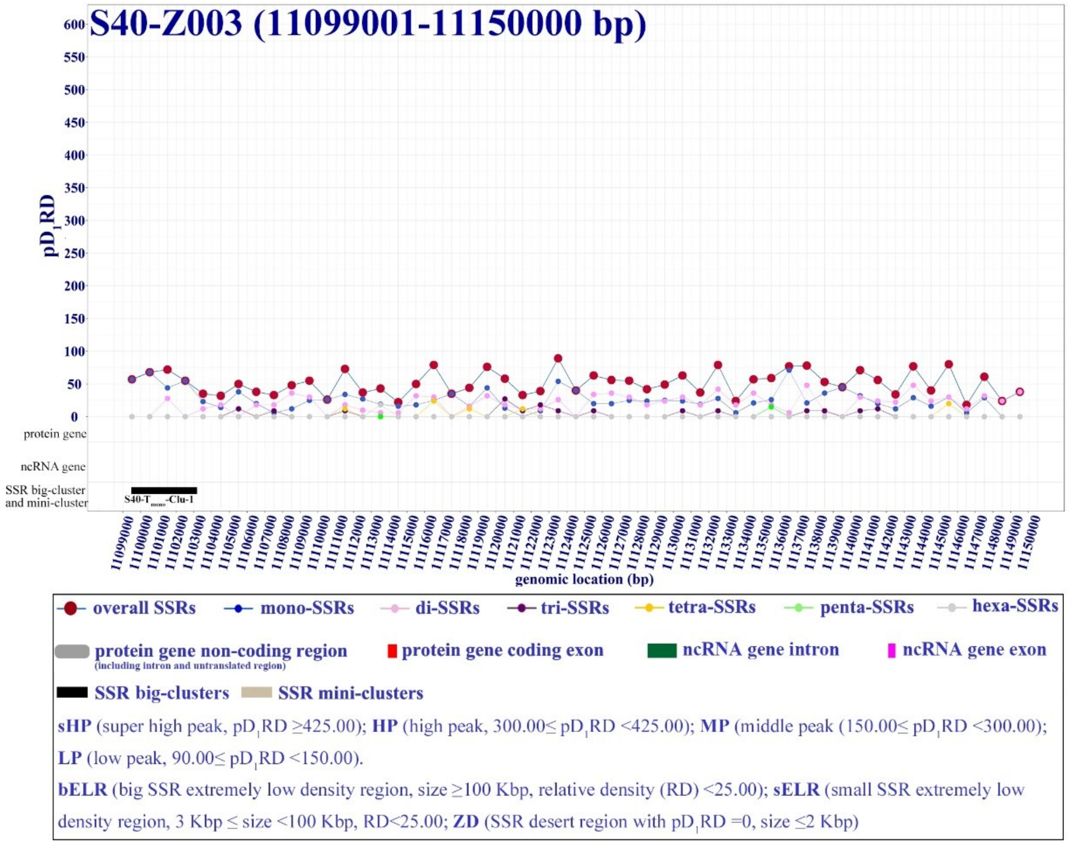

Supplementary Figure 1.219. The SSR position related D_1-relative density (pD_1RD) map of position at 11099001-11150000 bp of human reference Y-DNA (NC_000024.10) at resolution of 1 Kbp.

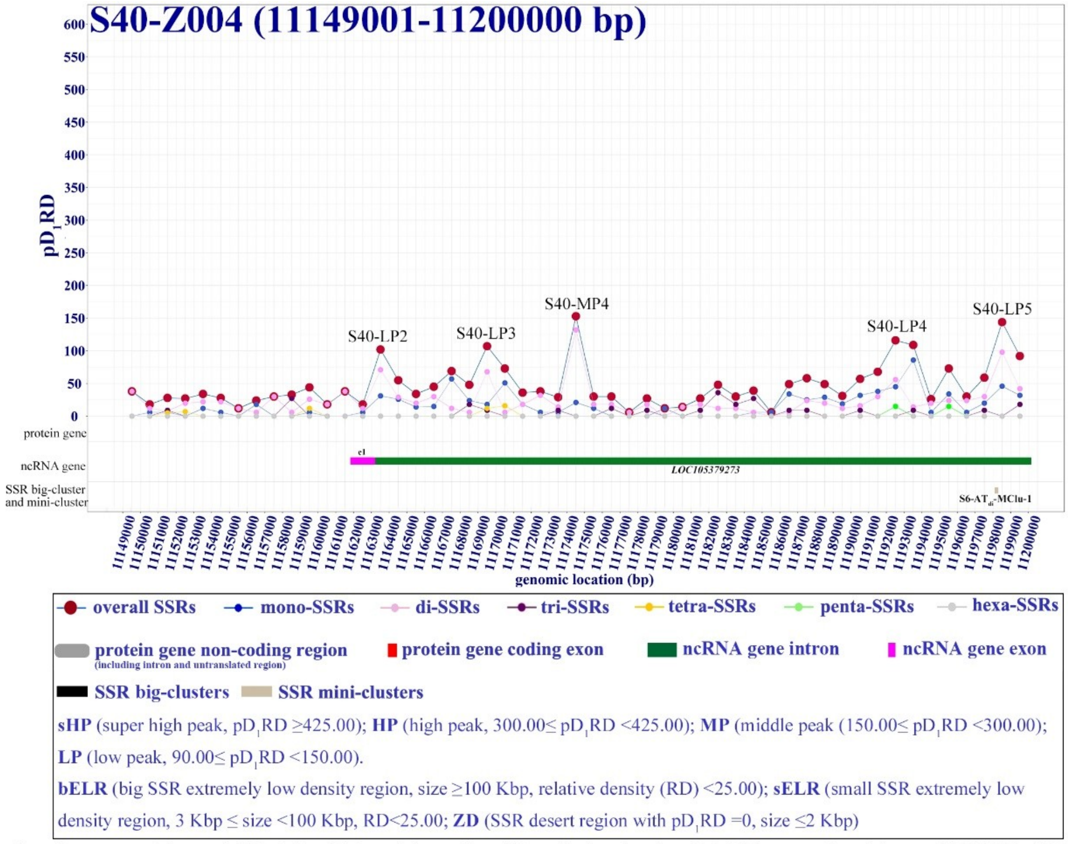

Supplementary Figure 1.220. The SSR position related D_1-relative density (pD_1RD) map of position at 11149001-11200000 bp of human reference Y-DNA (NC_000024.10) at resolution of 1 Kbp.

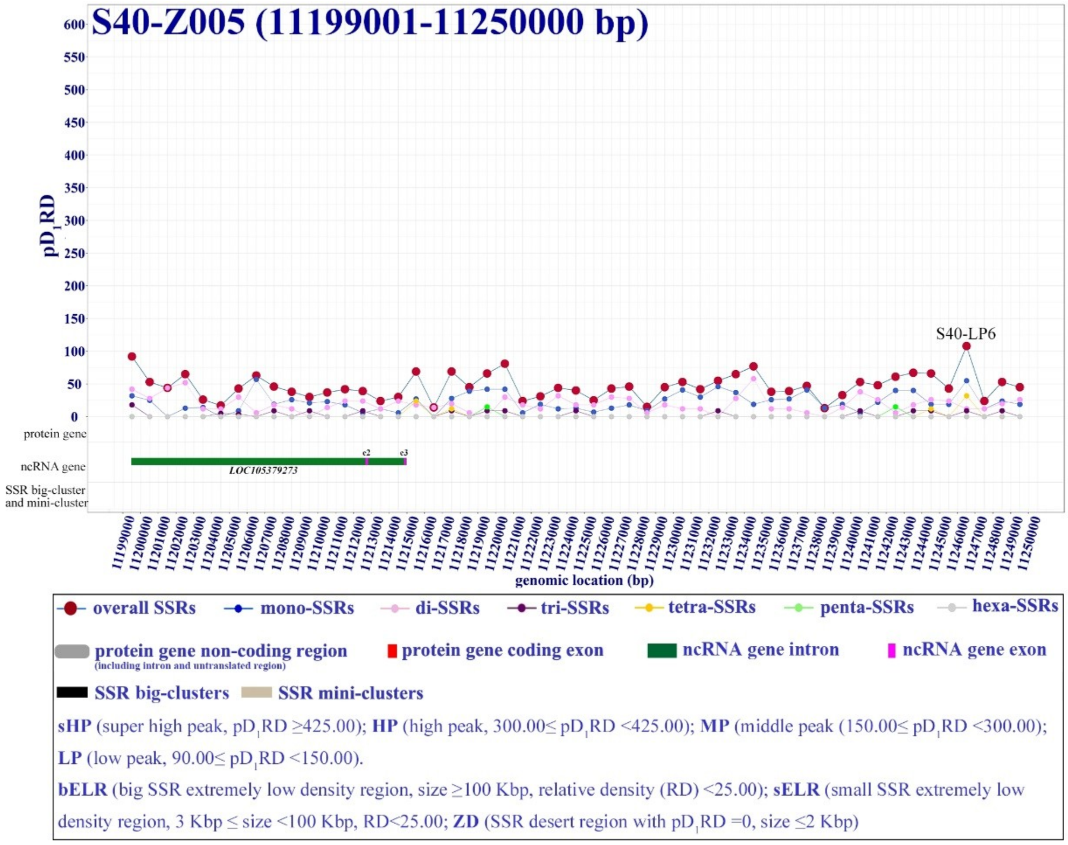

Supplementary Figure 1.221. The SSR position related D_1-relative density (pD_1RD) map of position at 11199001-11250000 bp of human reference Y-DNA (NC_000024.10) at resolution of 1 Kbp.

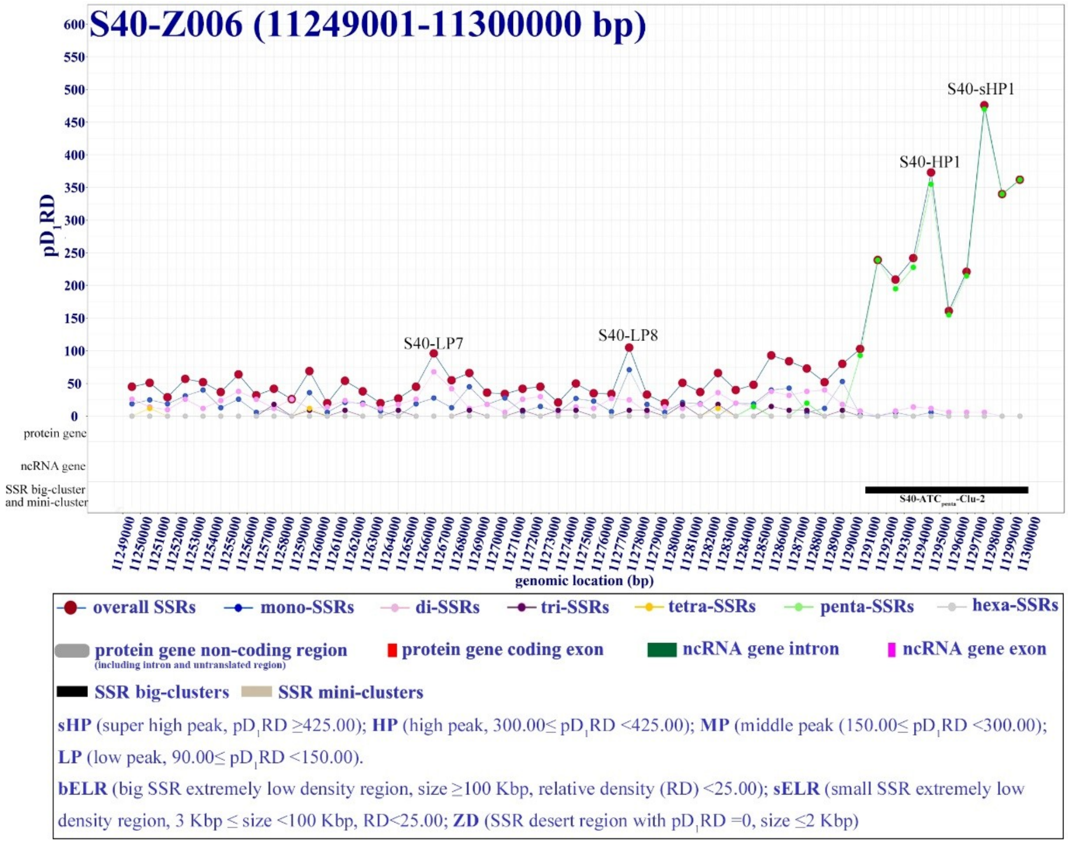

Supplementary Figure 1.222. The SSR position related D_1-relative density (pD_1RD) map of position at 11249001-11300000 bp of human reference Y-DNA (NC_000024.10) at resolution of 1 Kbp.

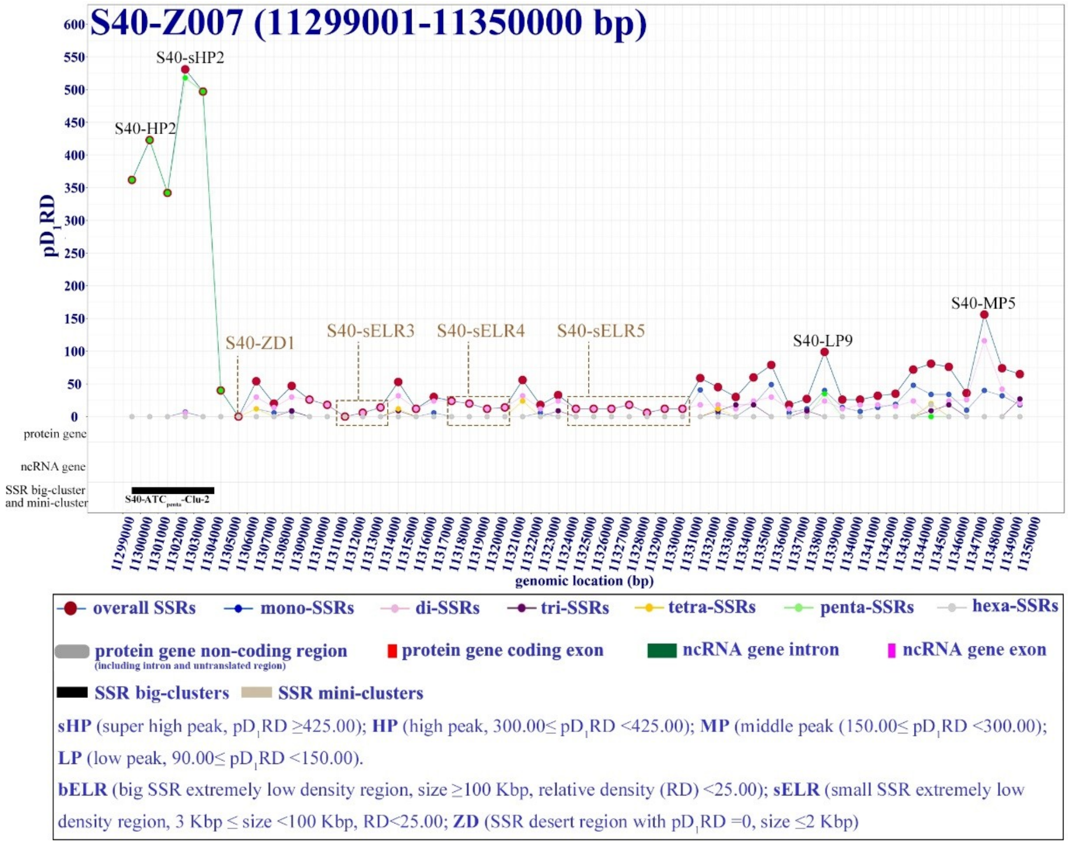

Supplementary Figure 1.223. The SSR position related D_1-relative density (pD_1RD) map of position at 11299001-11350000 bp of human reference Y-DNA (NC_000024.10) at resolution of 1 Kbp.

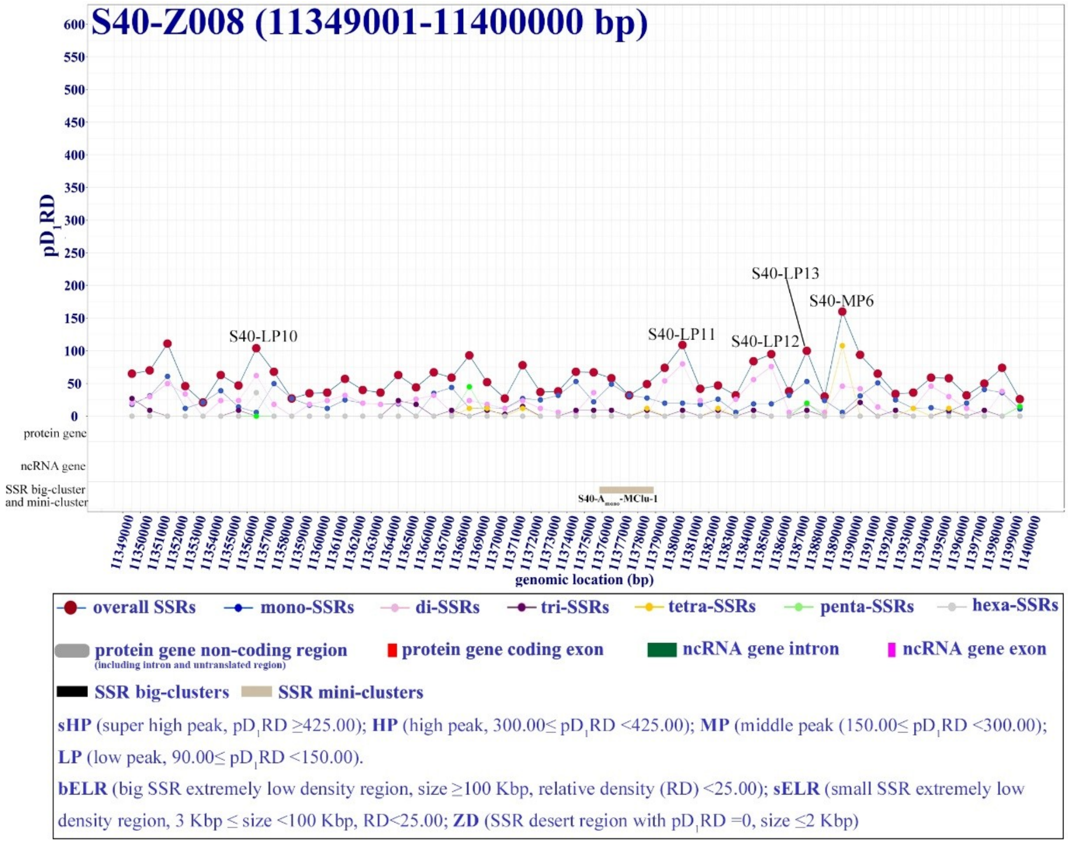

Supplementary Figure 1.224. The SSR position related D_1-relative density (pD_1RD) map of position at 11349001-11400000 bp of human reference Y-DNA (NC_000024.10) at resolution of 1 Kbp.

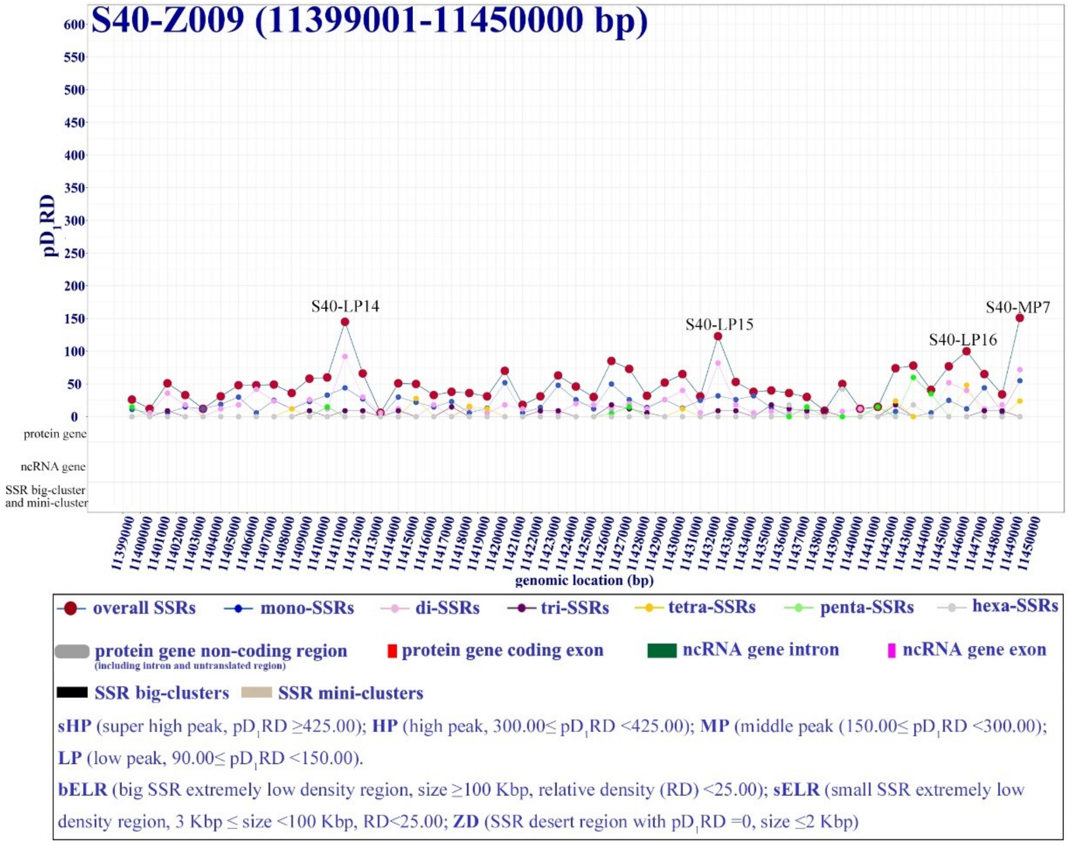

Supplementary Figure 1.225. The SSR position related D_1-relative density (pD_1RD) map of position at 11399001-11450000 bp of human reference Y-DNA (NC_000024.10) at resolution of 1 Kbp.

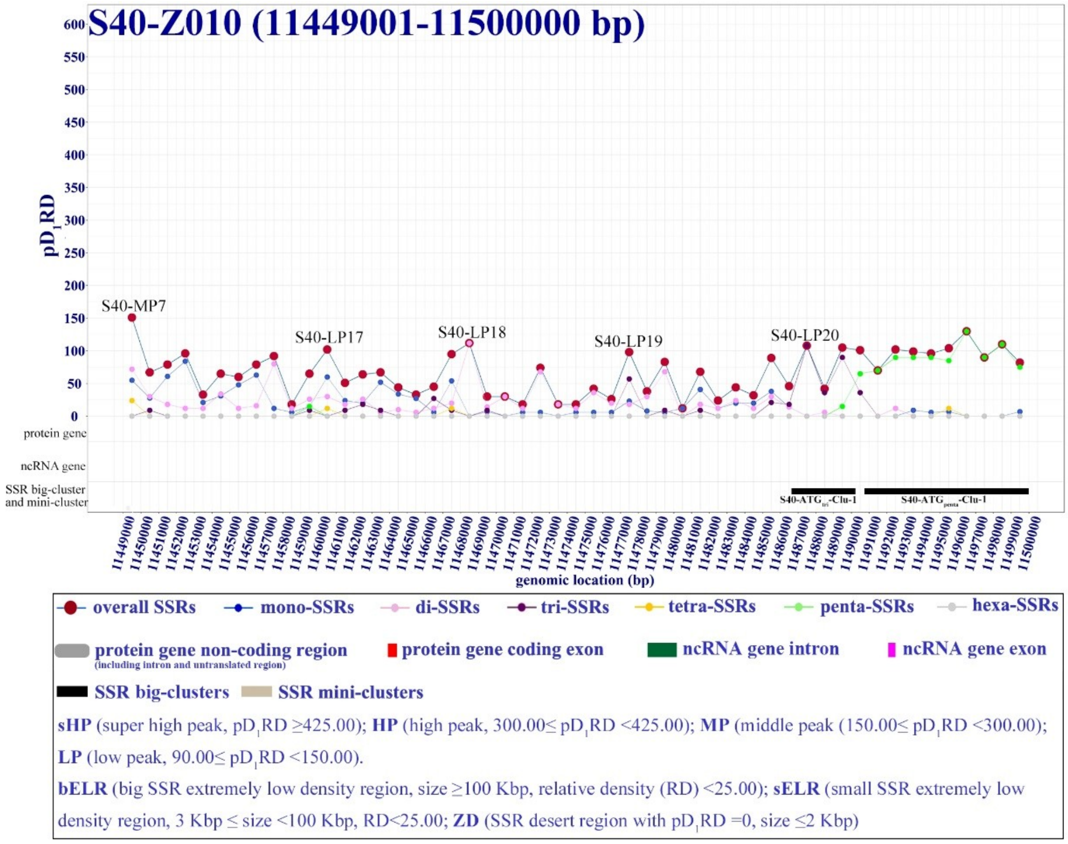

Supplementary Figure 1.226. The SSR position related D_1-relative density (pD_1RD) map of position at 11449001-11500000 bp of human reference Y-DNA (NC_000024.10) at resolution of 1 Kbp.

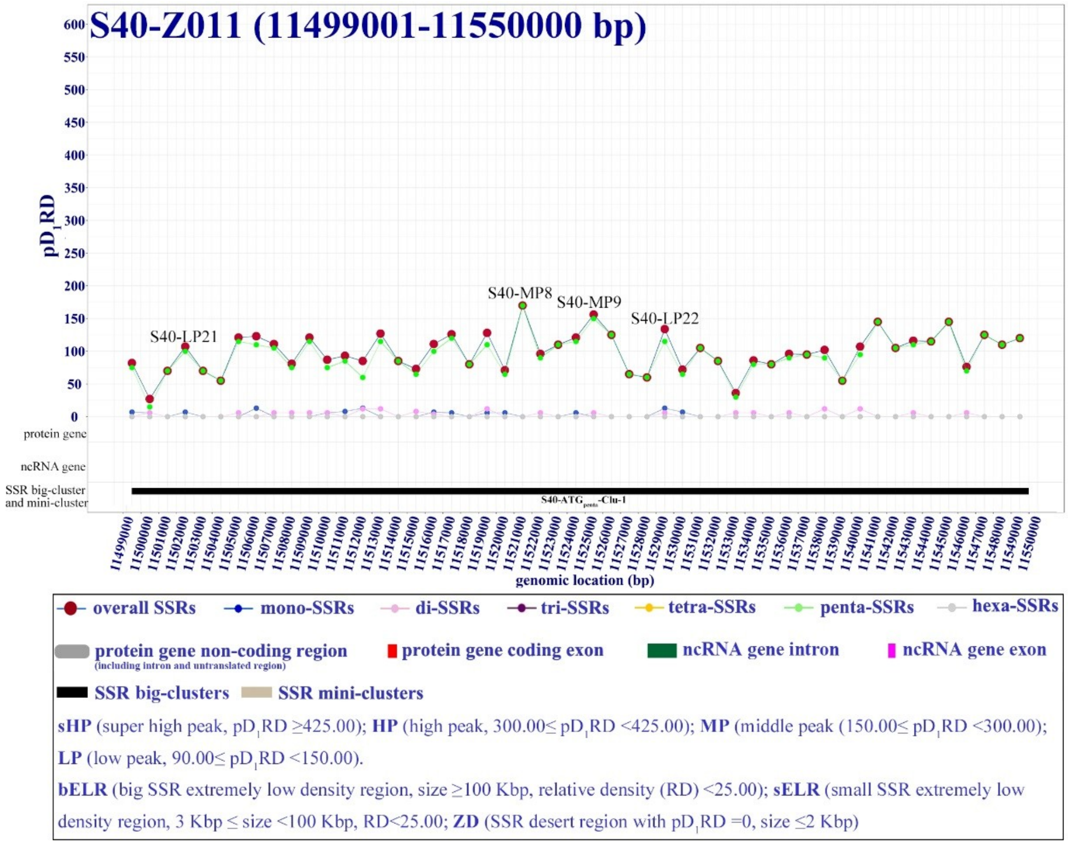

Supplementary Figure 1.227. The SSR position related D_1-relative density (pD_1RD) map of position at 11499001-11550000 bp of human reference Y-DNA (NC_000024.10) at resolution of 1 Kbp.

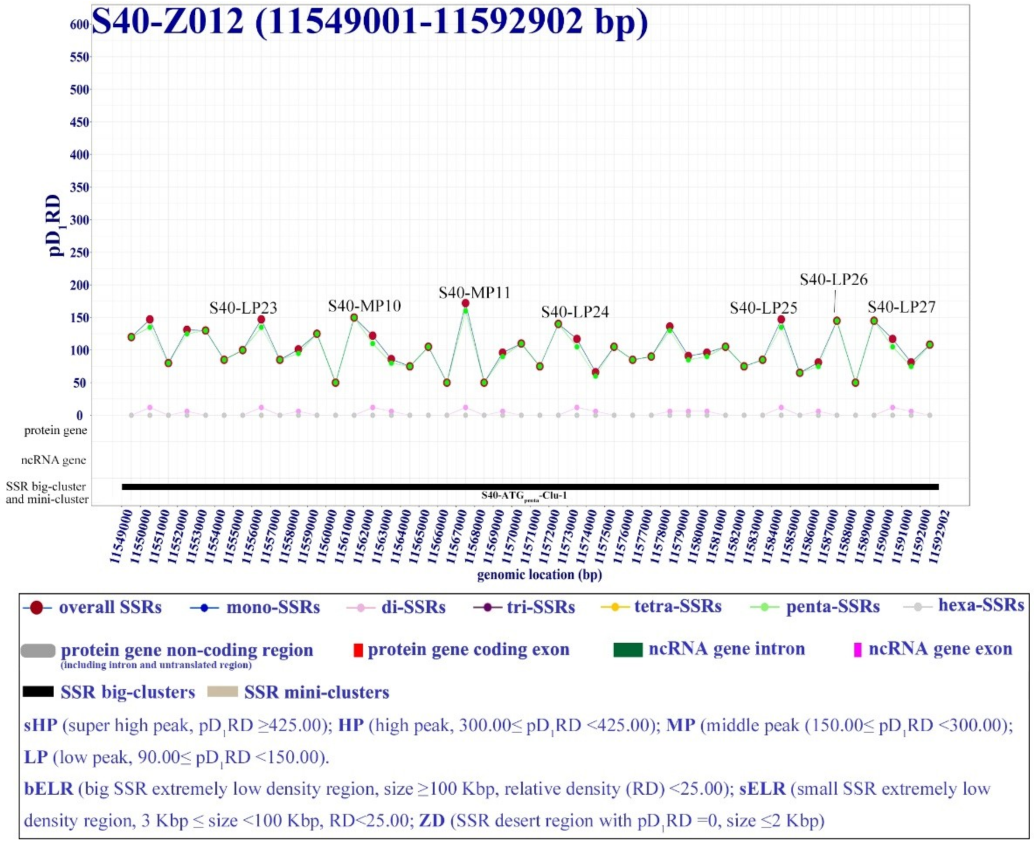

Supplementary Figure 1.228. The SSR position related D_1-relative density (pD_1RD) map of position at 11549001-11592902 bp (unnormal zone <51000 bp) of human reference Y-DNA (NC_000024.10) at resolution of 1 Kbp.

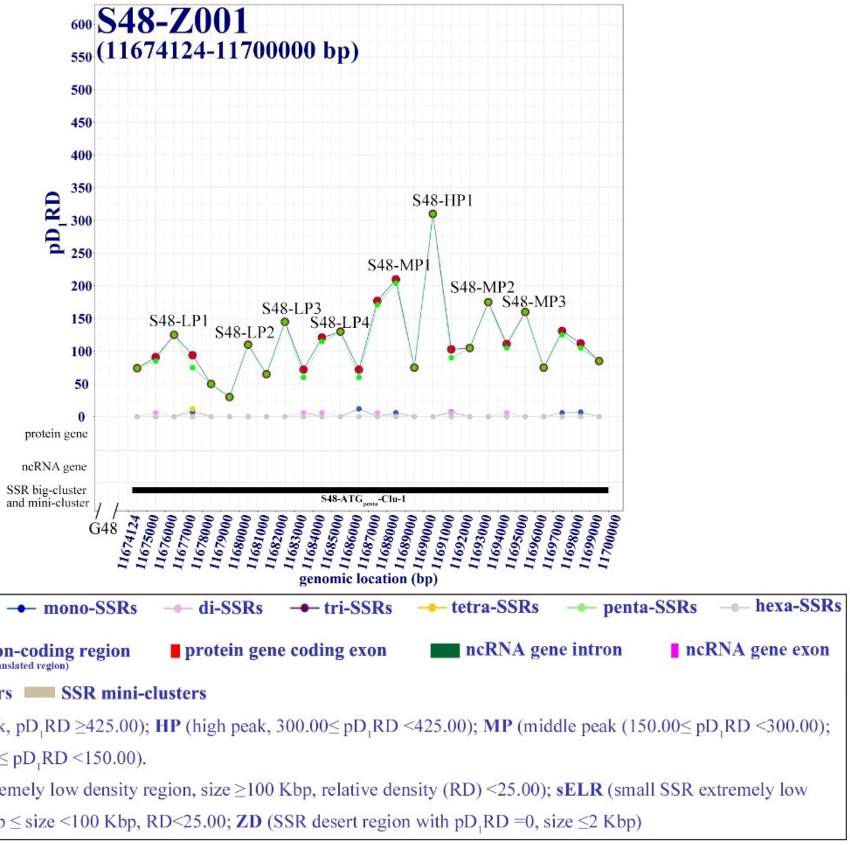

Supplementary Figure 1.229. The SSR position related D_1-relative density (pD_1RD) map of position at 11674124-11700000 bp (unnormal zone <51000 bp) of human reference Y-DNA (NC_000024.10) at resolution of 1 Kbp.

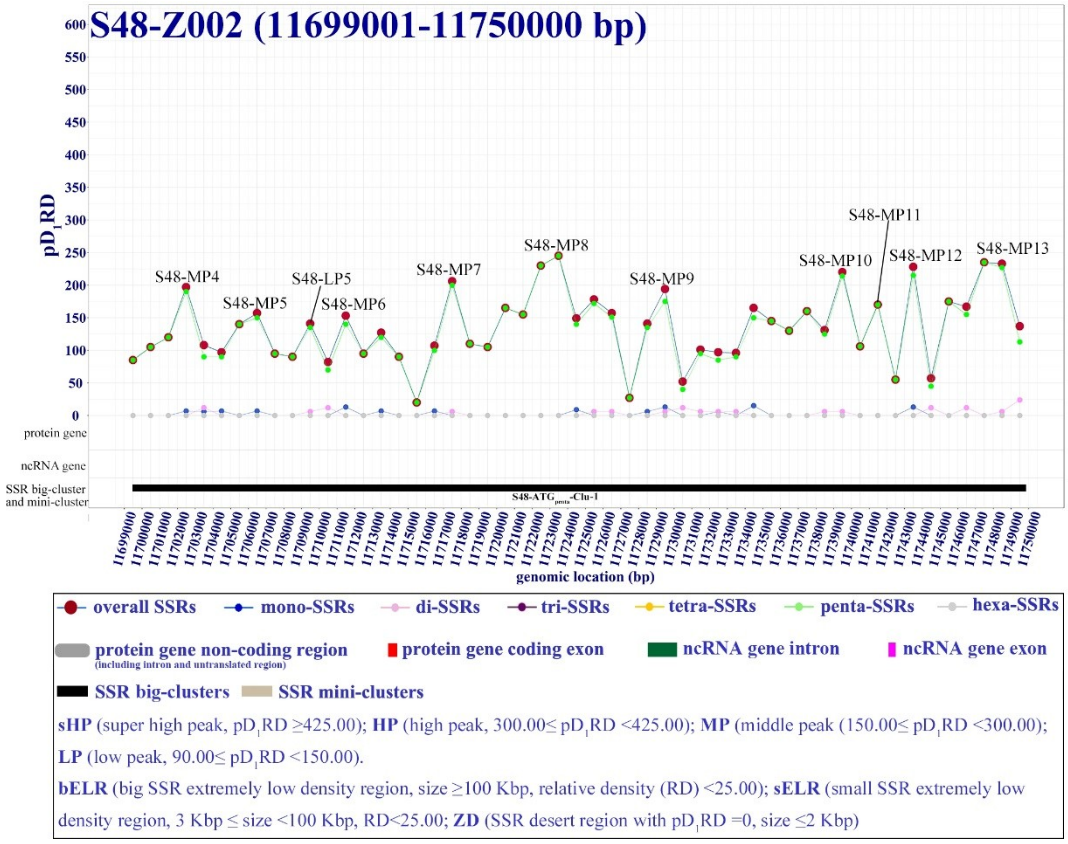

Supplementary Figure 1.230. The SSR position related D_1-relative density (pD_1RD) map of position at 11699001-11750000 bp of human reference Y-DNA (NC_000024.10) at resolution of 1 Kbp.

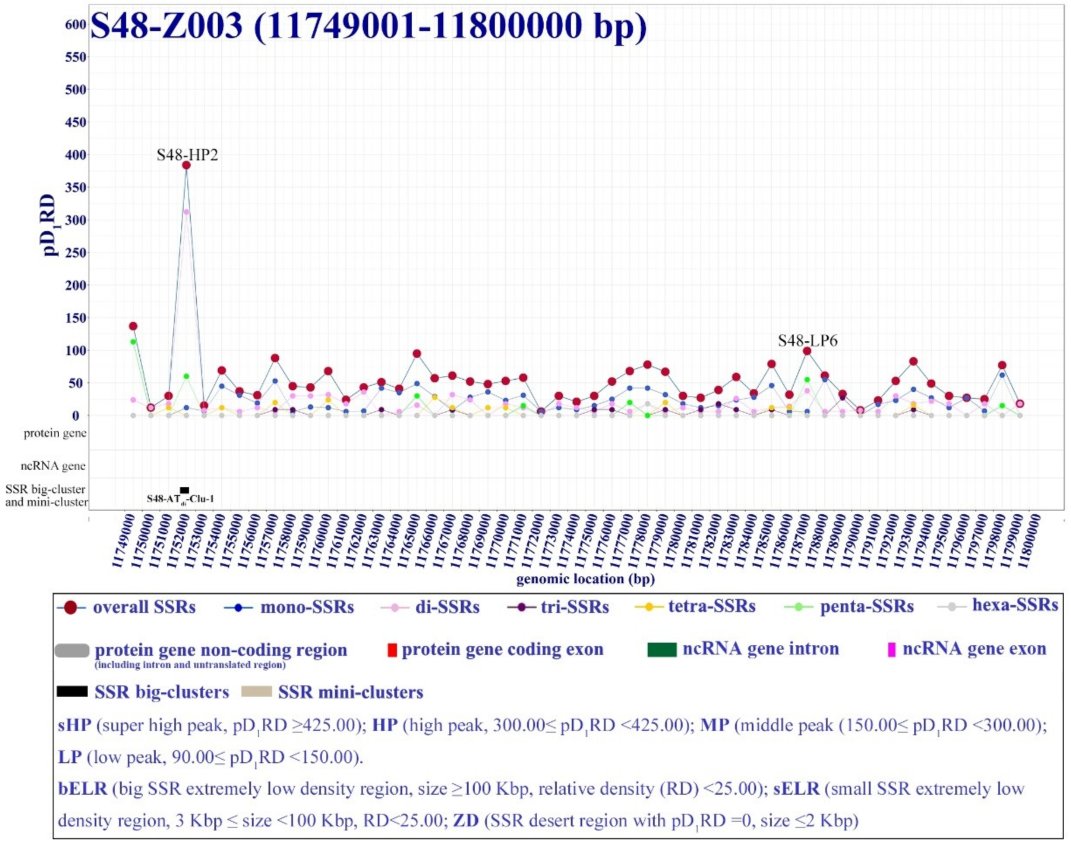

Supplementary Figure 1.231. The SSR position related D_1-relative density (pD_1RD) map of position at 11749001-11800000 bp of human reference Y-DNA (NC_000024.10) at resolution of 1 Kbp.

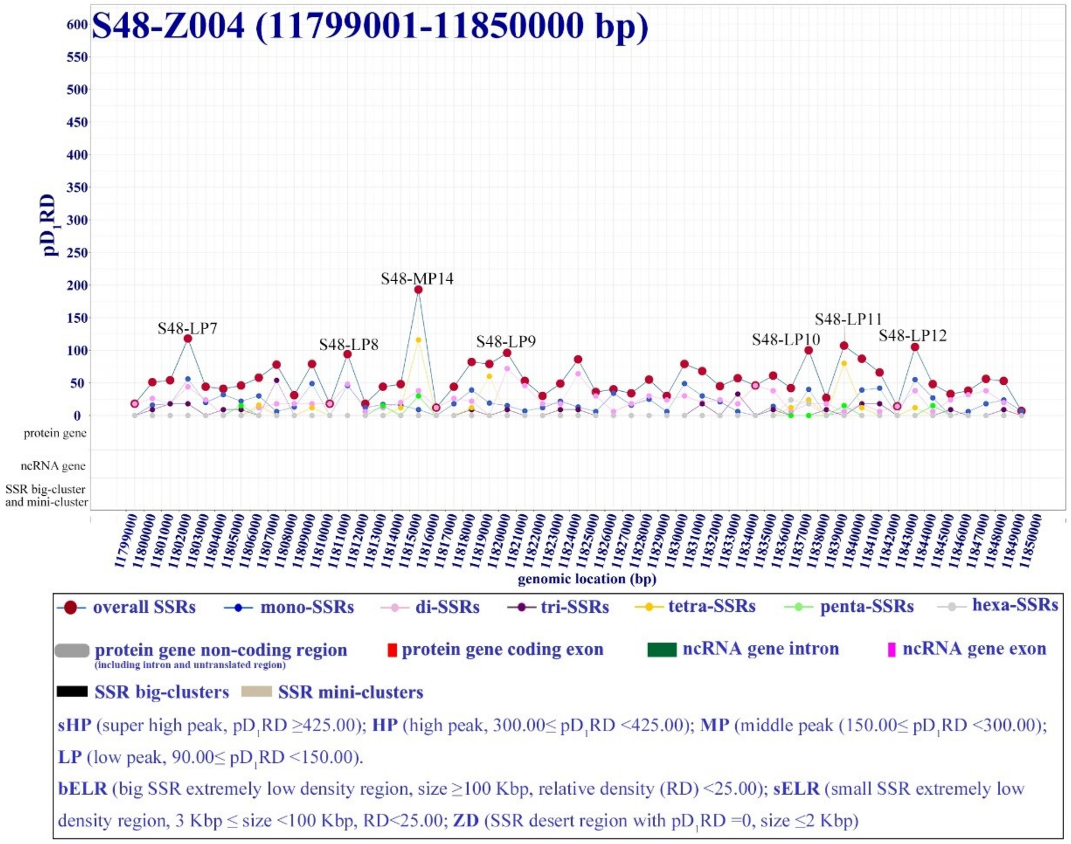

Supplementary Figure 1.232. The SSR position related D_1-relative density (pD_1RD) map of position at 11799001-11850000 bp of human reference Y-DNA (NC_000024.10) at resolution of 1 Kbp.

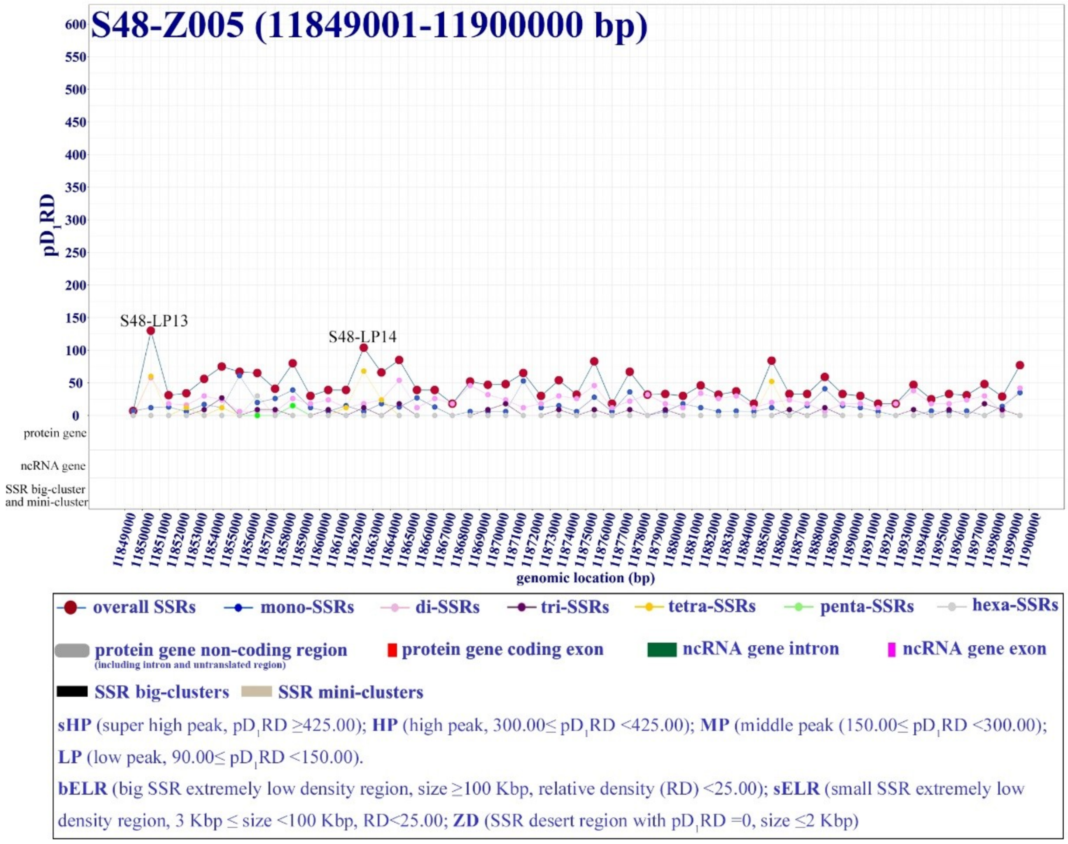

Supplementary Figure 1.233. The SSR position related D_1-relative density (pD_1RD) map of position at 11849001-11900000 bp of human reference Y-DNA (NC_000024.10) at resolution of 1 Kbp.

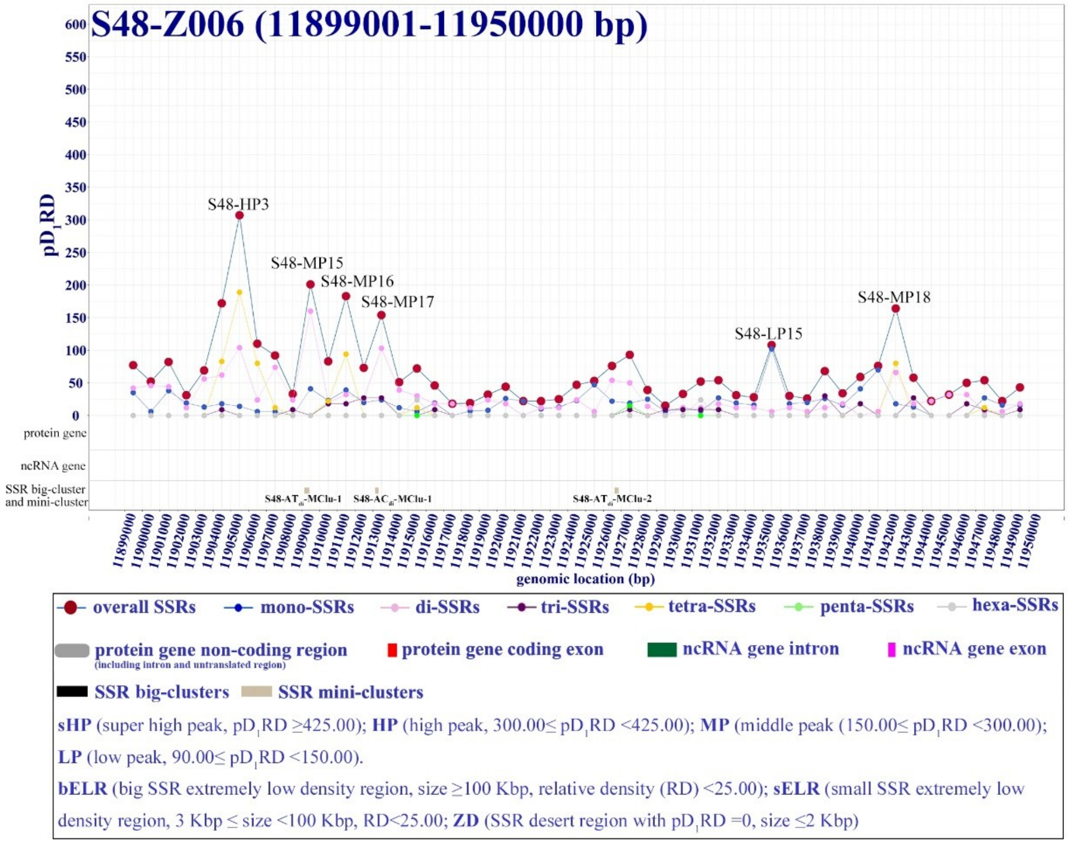

Supplementary Figure 1.234. The SSR position related D_1-relative density (pD_1RD) map of position at 11899001-11950000 bp of human reference Y-DNA (NC_000024.10) at resolution of 1 Kbp.

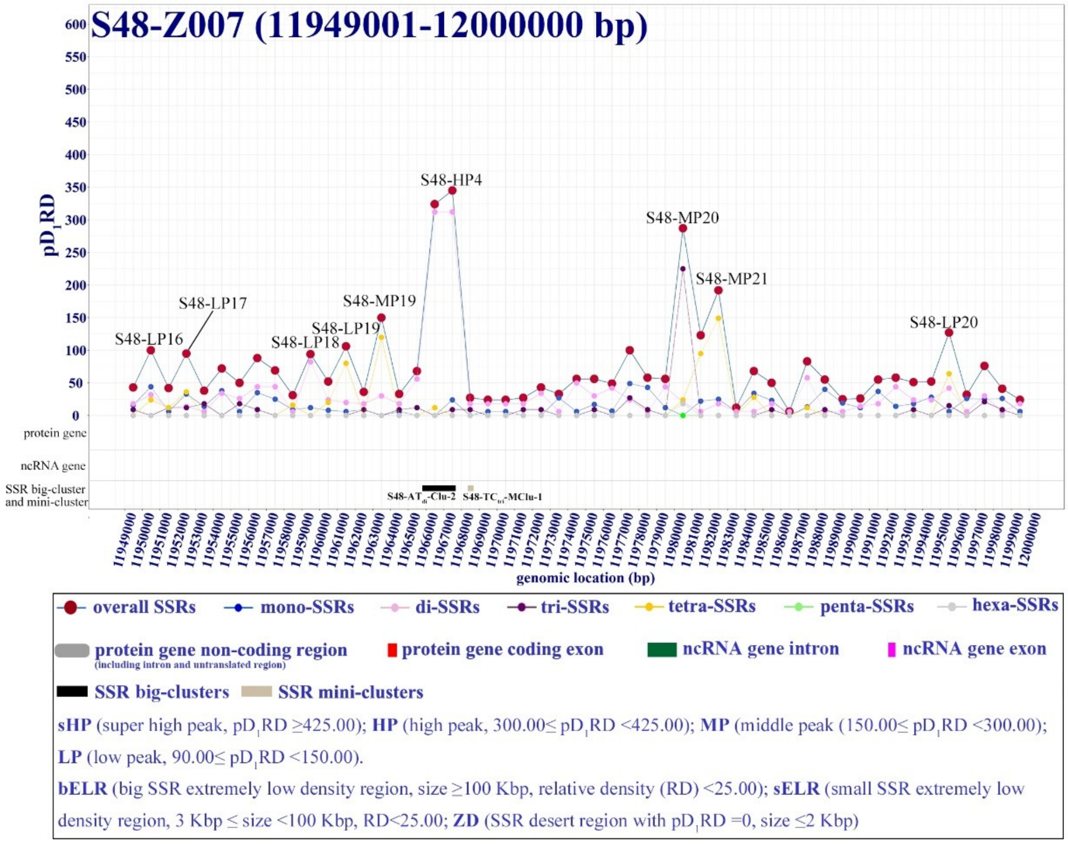

Supplementary Figure 1.235. The SSR position related D_1-relative density (pD_1RD) map of position at 11949001-12000000 bp of human reference Y-DNA (NC_000024.10) at resolution of 1 Kbp.

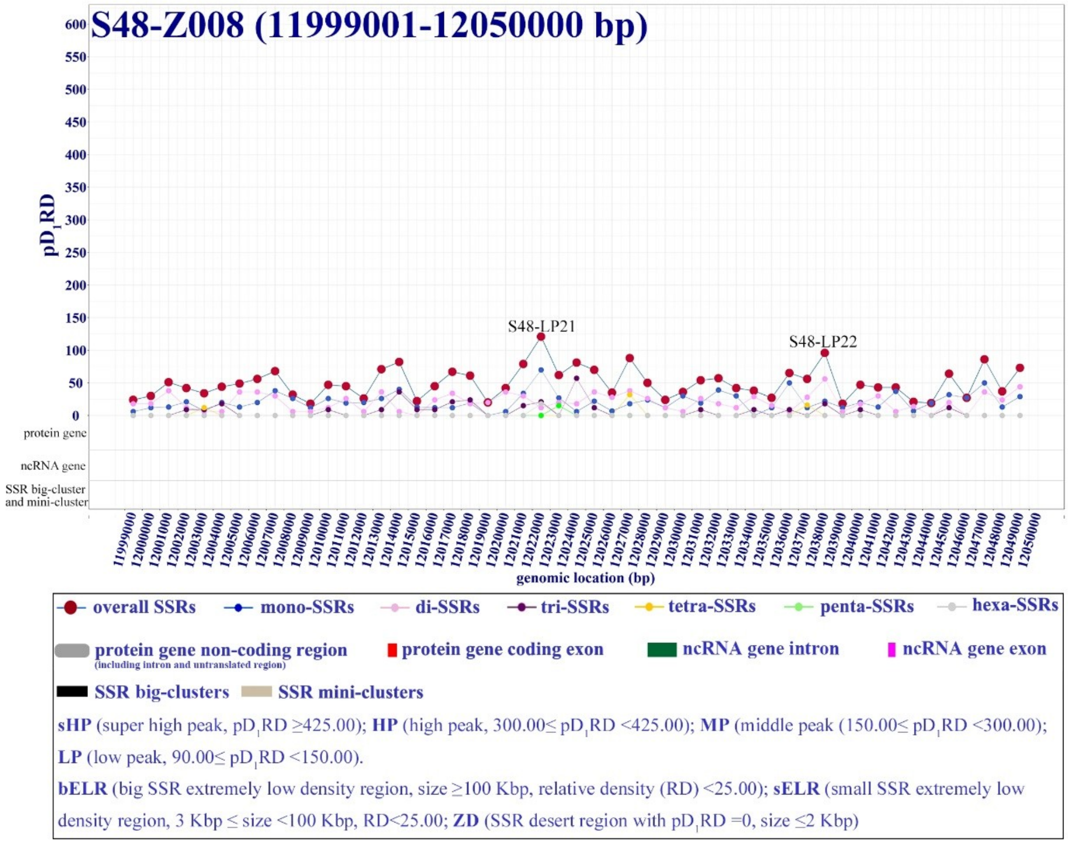

Supplementary Figure 1.236. The SSR position related D_1-relative density (pD_1RD) map of position at 11999001-12050000 bp of human reference Y-DNA (NC_000024.10) at resolution of 1 Kbp.

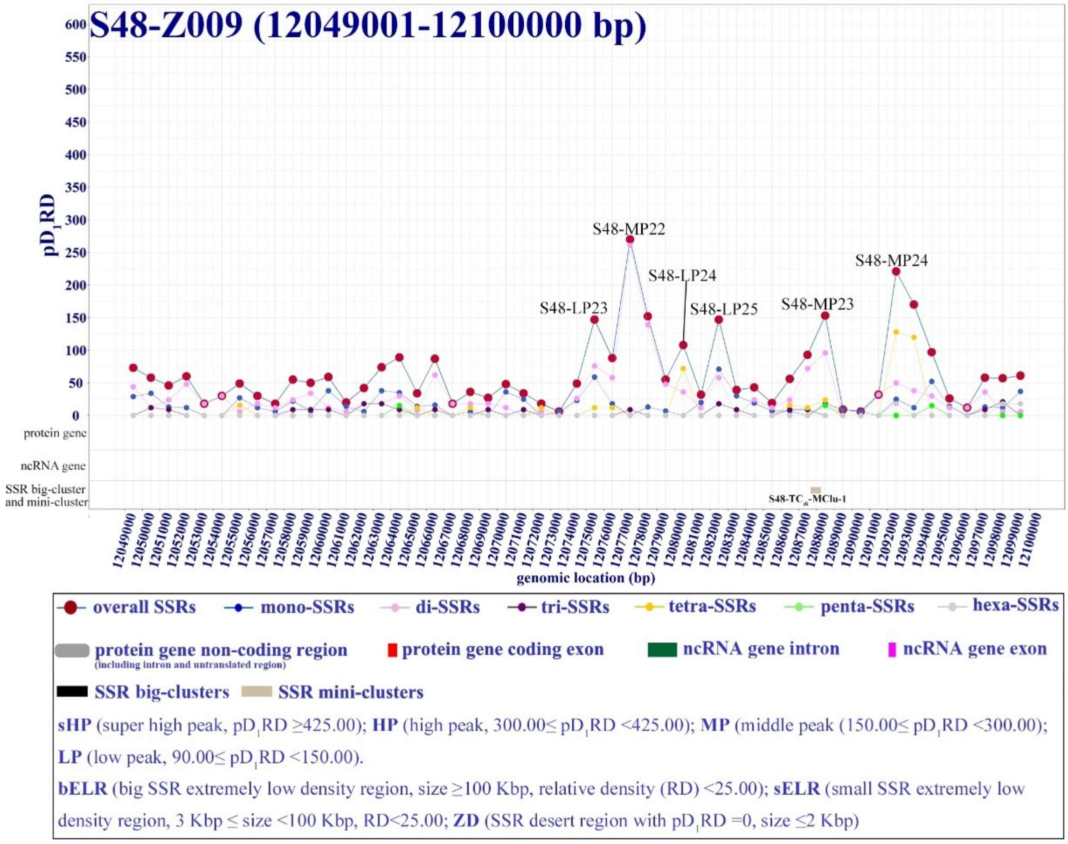

Supplementary Figure 1.237. The SSR position related D_1-relative density (pD_1RD) map of position at 12049001-12100000 bp of human reference Y-DNA (NC_000024.10) at resolution of 1 Kbp.

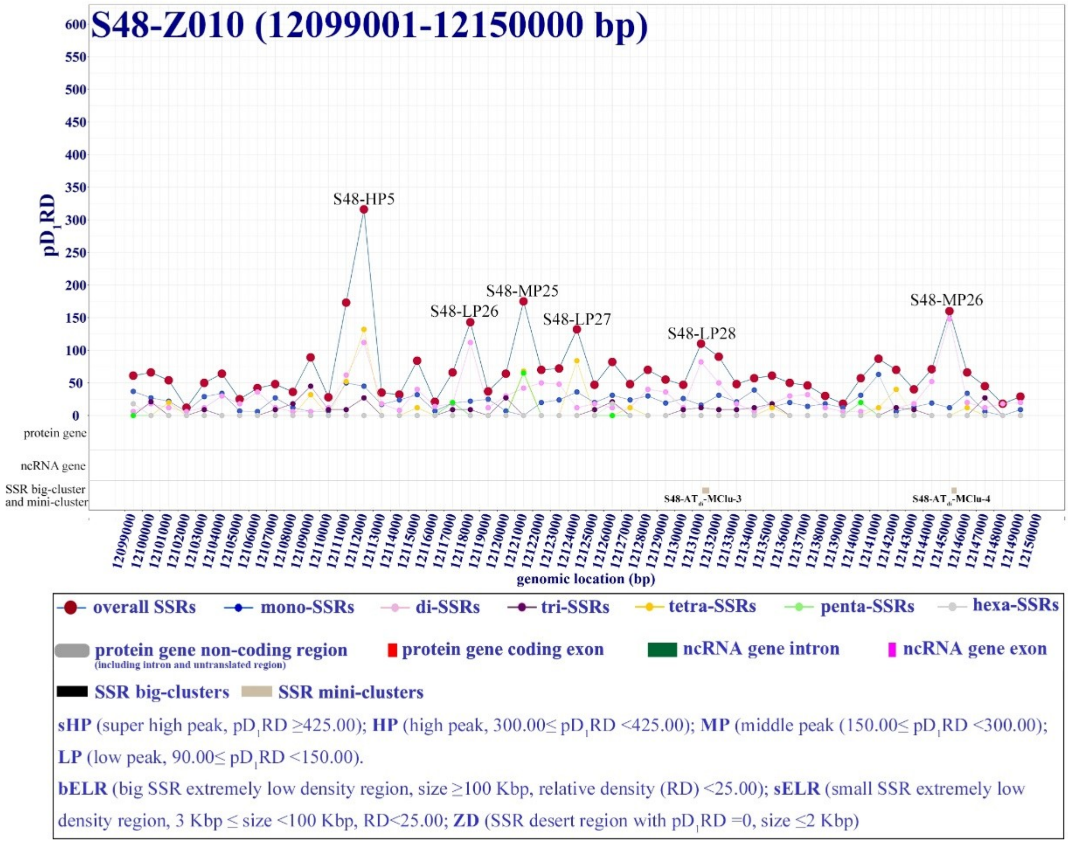

Supplementary Figure 1.238. The SSR position related D_1-relative density (pD_1RD) map of position at 12099001-12150000 bp of human reference Y-DNA (NC_000024.10) at resolution of 1 Kbp.

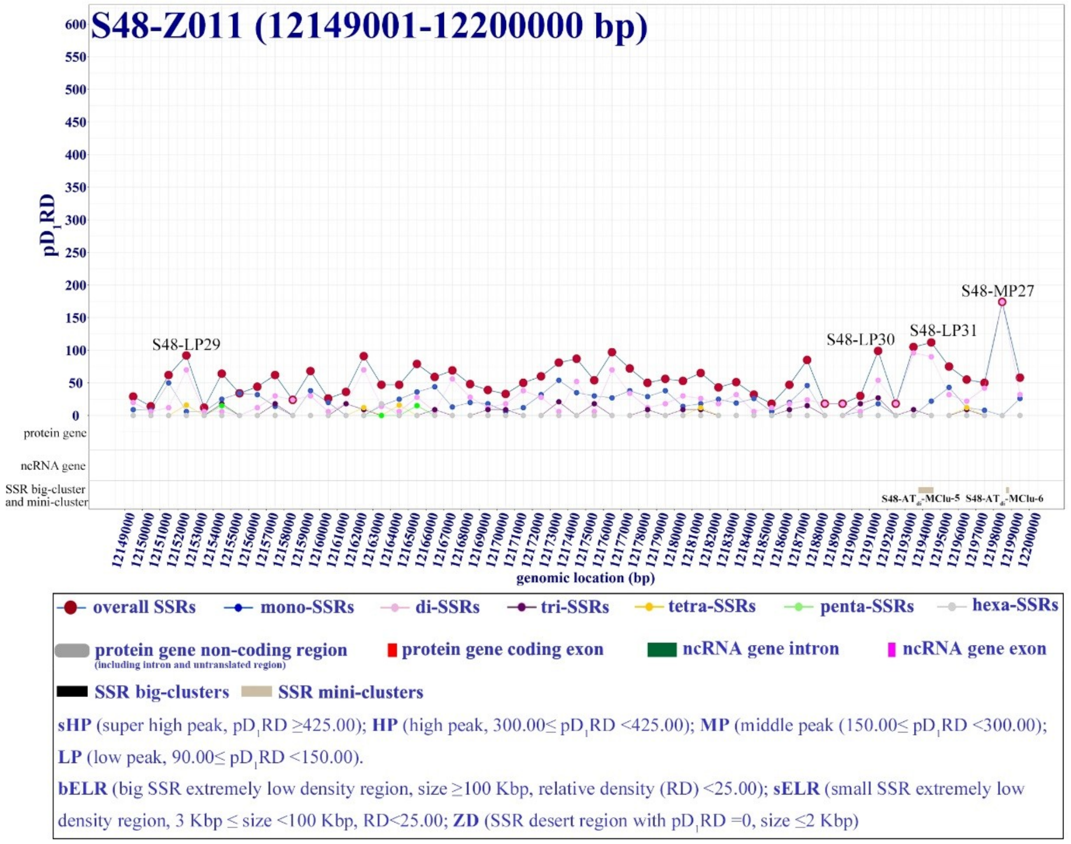

Supplementary Figure 1.239. The SSR position related D_1-relative density (pD_1RD) map of position at 12149001-12200000 bp of human reference Y-DNA (NC_000024.10) at resolution of 1 Kbp.

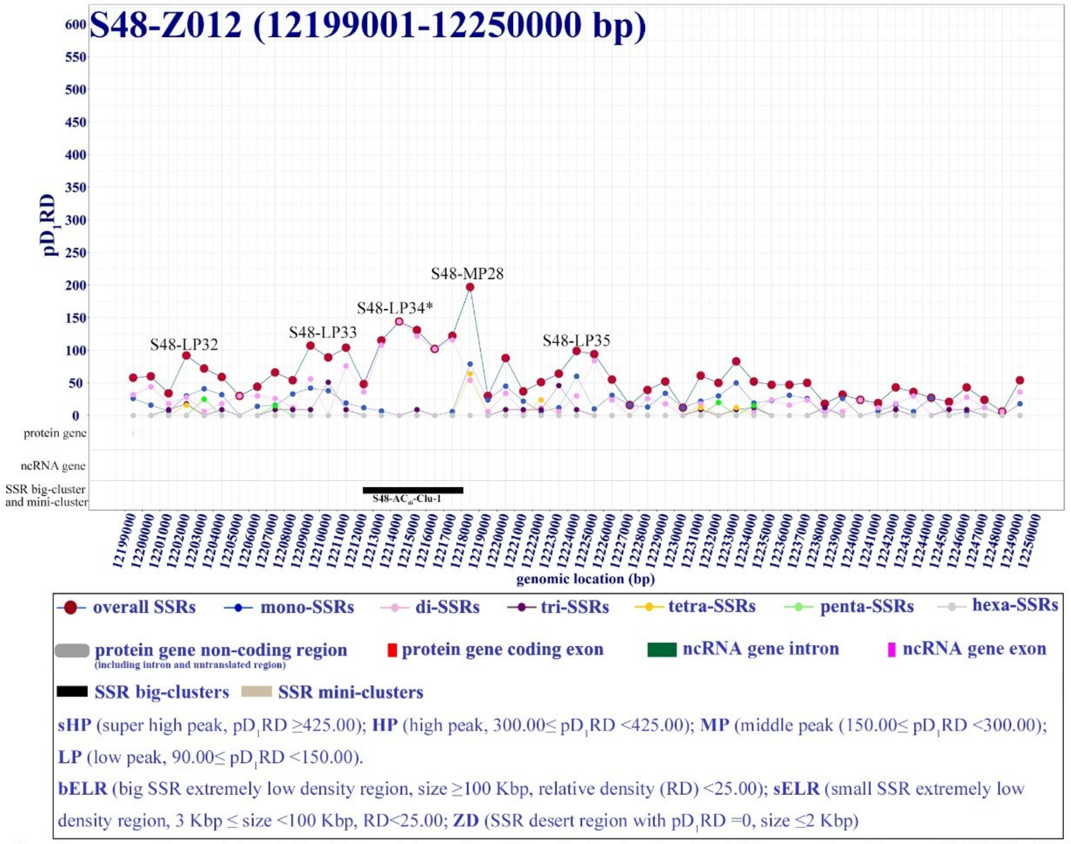

Supplementary Figure 1.240. The SSR position related D_1-relative density (pD_1RD) map of position at 12199001-12250000 bp of human reference Y-DNA (NC_000024.10) at resolution of 1 Kbp.

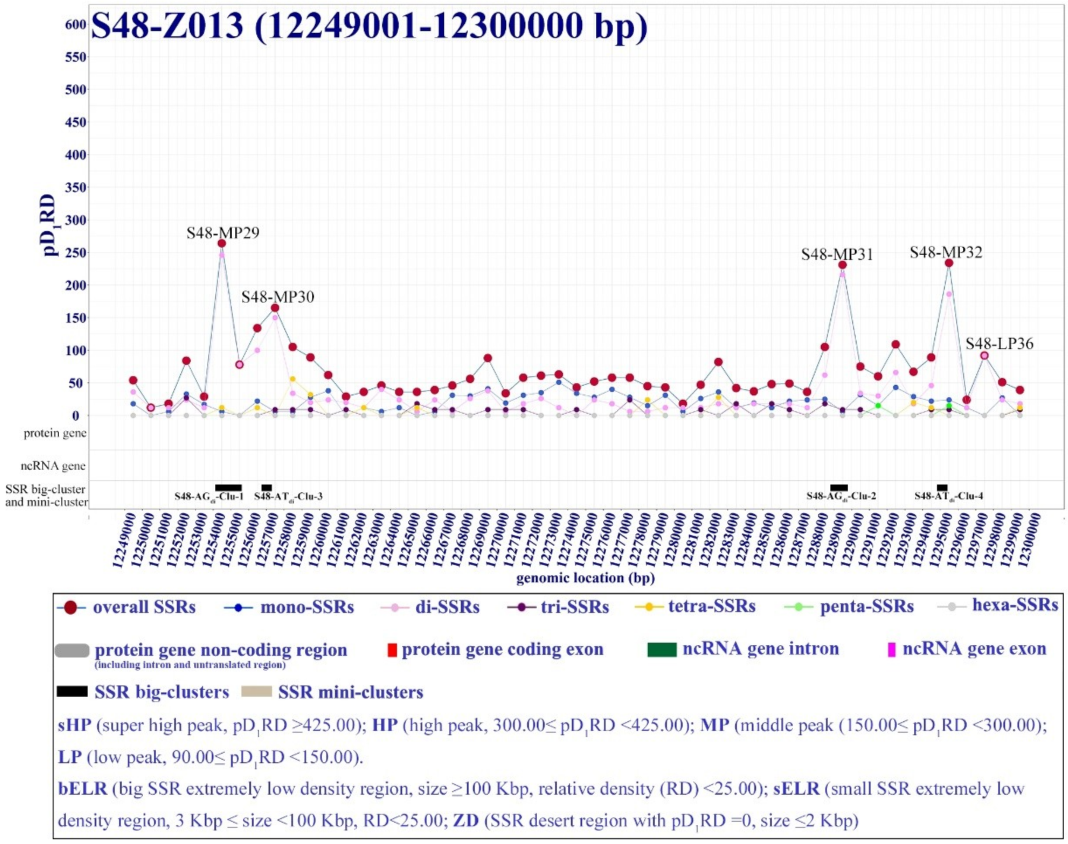

Supplementary Figure 1.241. The SSR position related D_1-relative density (pD_1RD) map of position at 12249001-12300000 bp of human reference Y-DNA (NC_000024.10) at resolution of 1 Kbp.

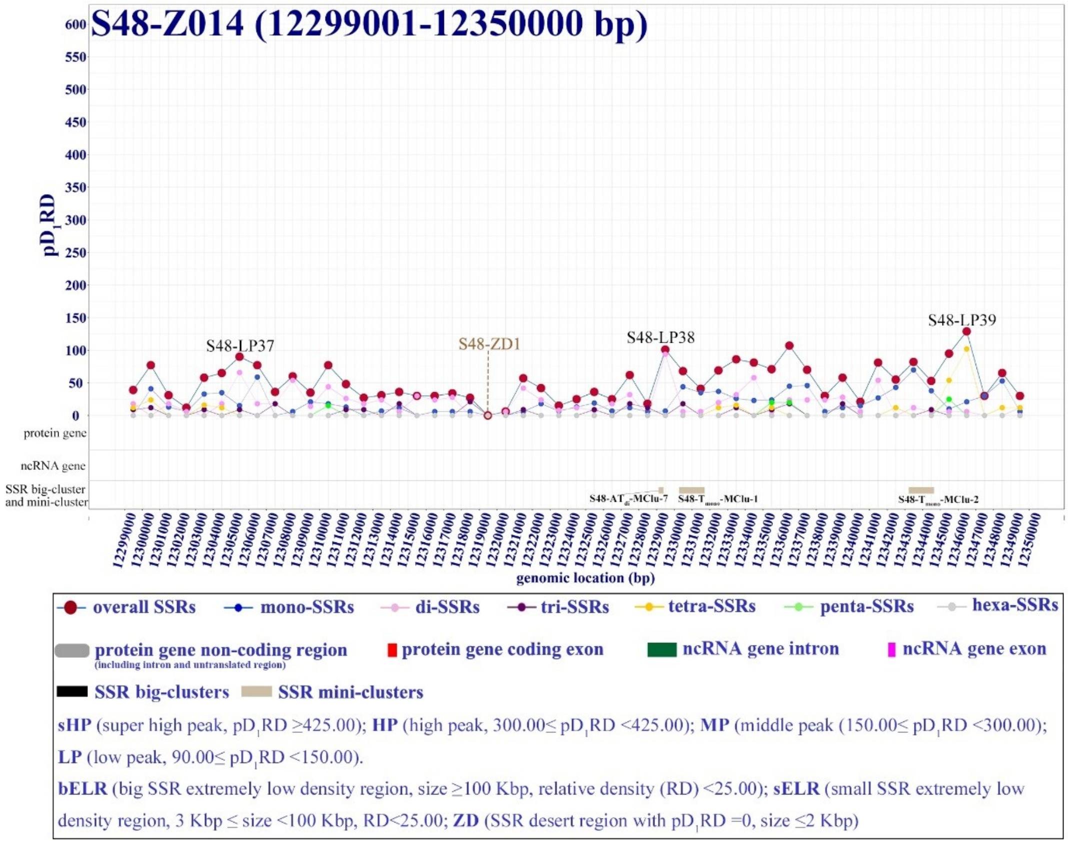

Supplementary Figure 1.242. The SSR position related D_1-relative density (pD_1RD) map of position at 12299001-12350000 bp of human reference Y-DNA (NC_000024.10) at resolution of 1 Kbp.

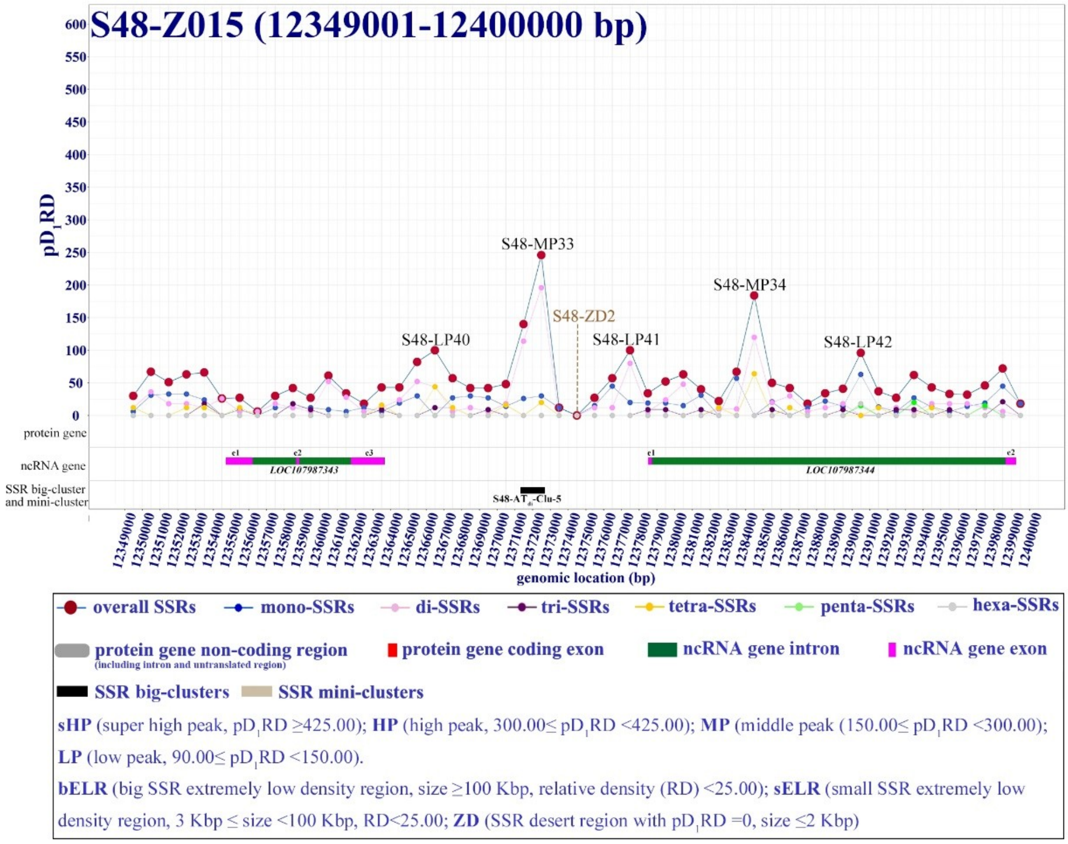

Supplementary Figure 1.243. The SSR position related D_1-relative density (pD_1RD) map of position at 12349001-12400000 bp of human reference Y-DNA (NC_000024.10) at resolution of 1 Kbp.

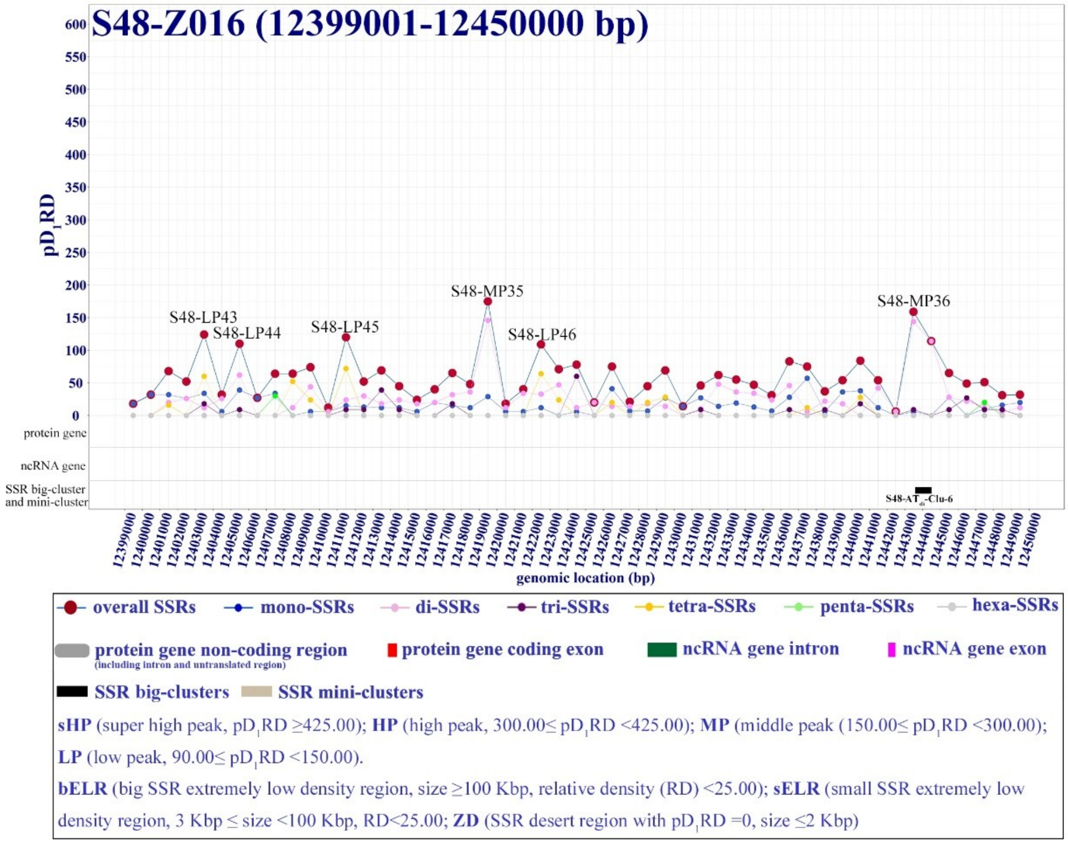

Supplementary Figure 1.244. The SSR position related D_1-relative density (pD_1RD) map of position at 12399001-12450000 bp of human reference Y-DNA (NC_000024.10) at resolution of 1 Kbp.

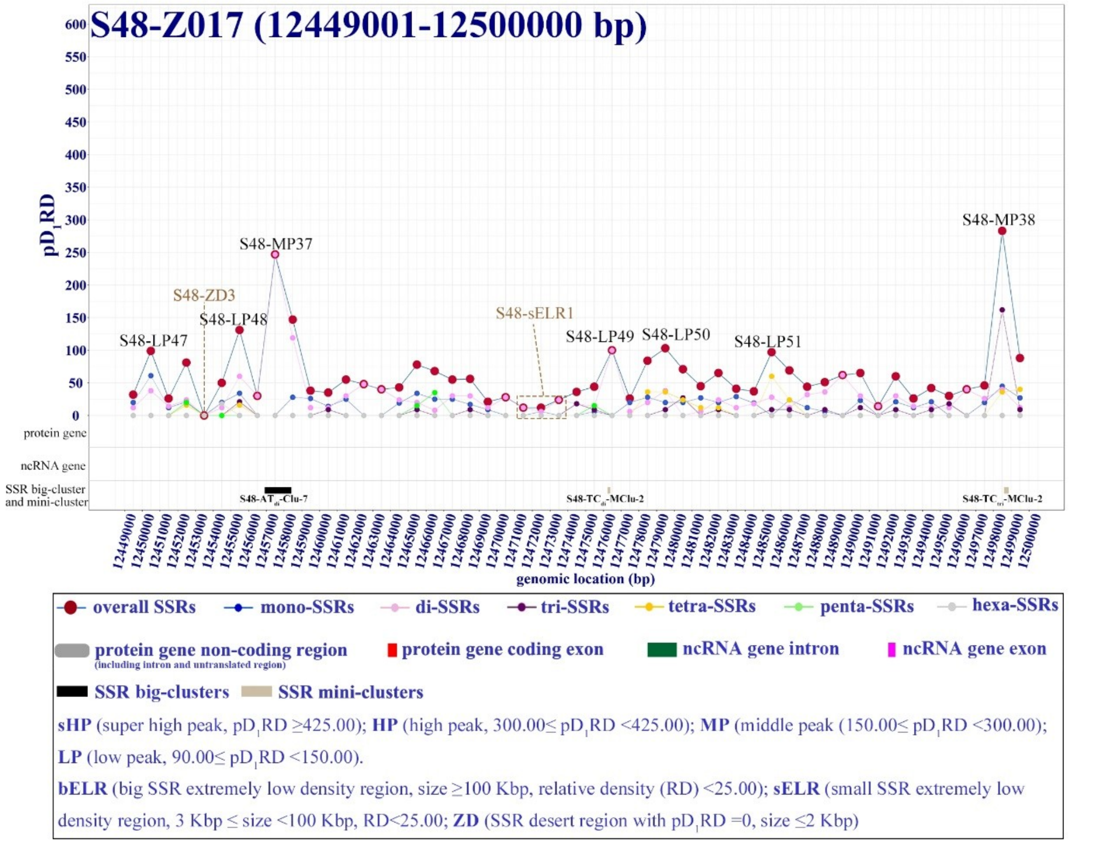

Supplementary Figure 1.245. The SSR position related D_1-relative density (pD_1RD) map of position at 12449001-12500000 bp of human reference Y-DNA (NC_000024.10) at resolution of 1 Kbp.

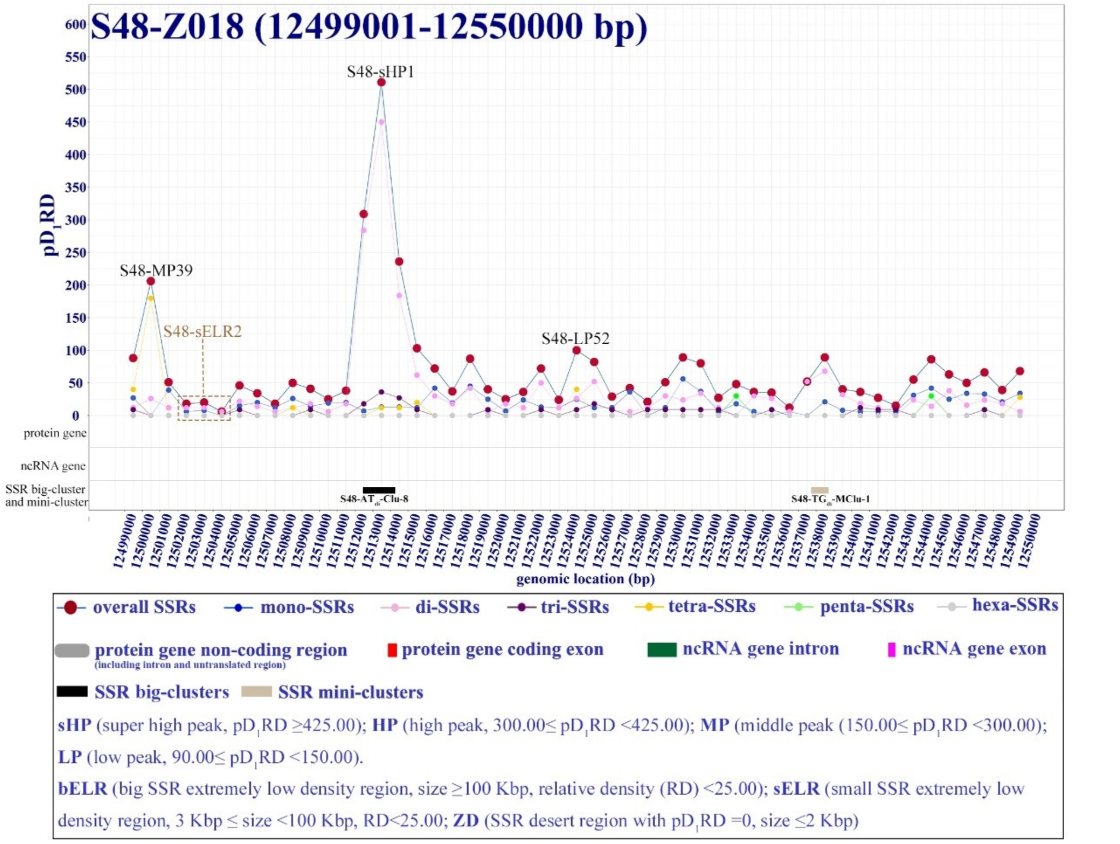

Supplementary Figure 1.246. The SSR position related D_1-relative density (pD_1RD) map of position at 12499001-12550000 bp of human reference Y-DNA (NC_000024.10) at resolution of 1 Kbp.

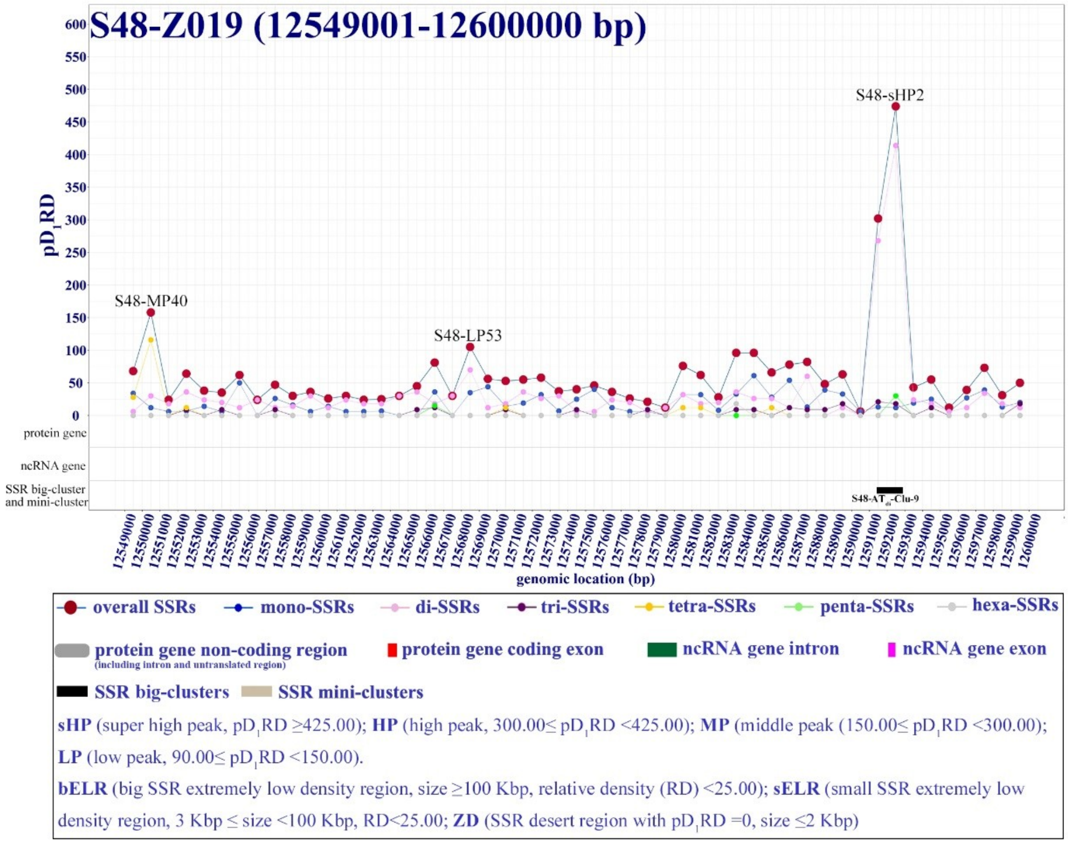

Supplementary Figure 1.247. The SSR position related D_1-relative density (pD_1RD) map of position at 12549001-12600000 bp of human reference Y-DNA (NC_000024.10) at resolution of 1 Kbp.

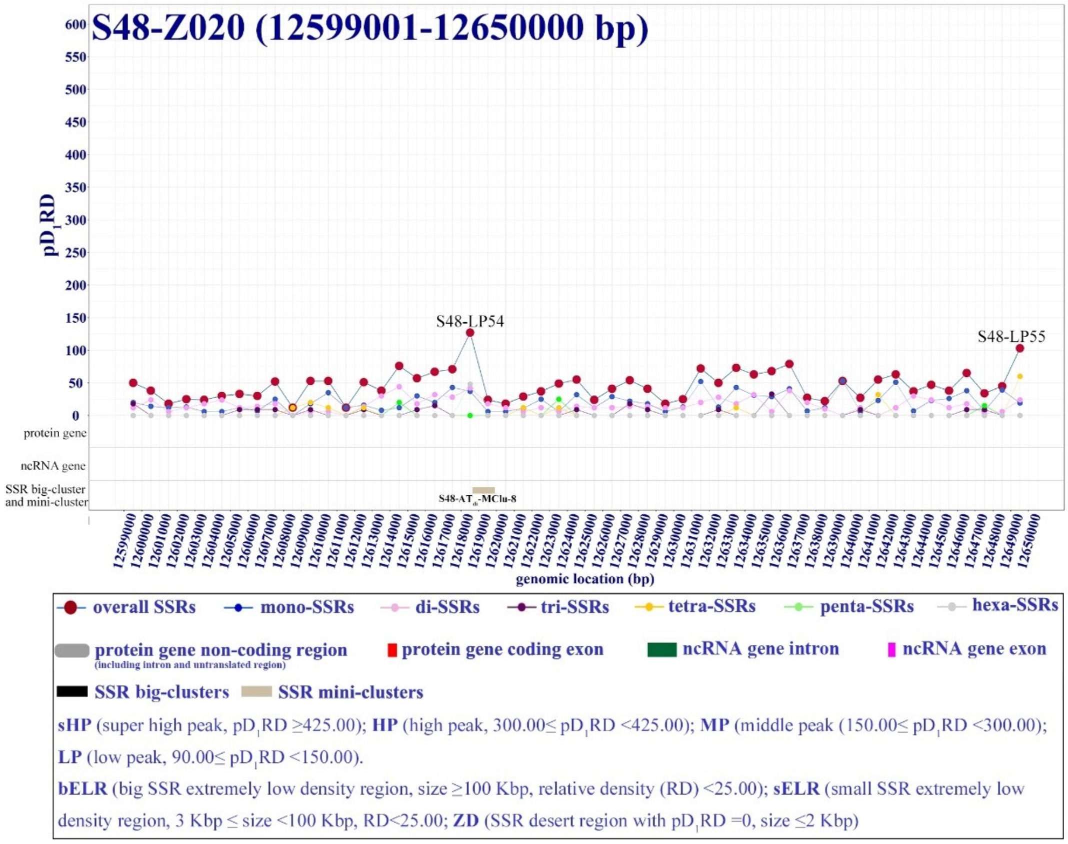

Supplementary Figure 1.248. The SSR position related D_1-relative density (pD_1RD) map of position at 12599001-12650000 bp of human reference Y-DNA (NC_000024.10) at resolution of 1 Kbp.

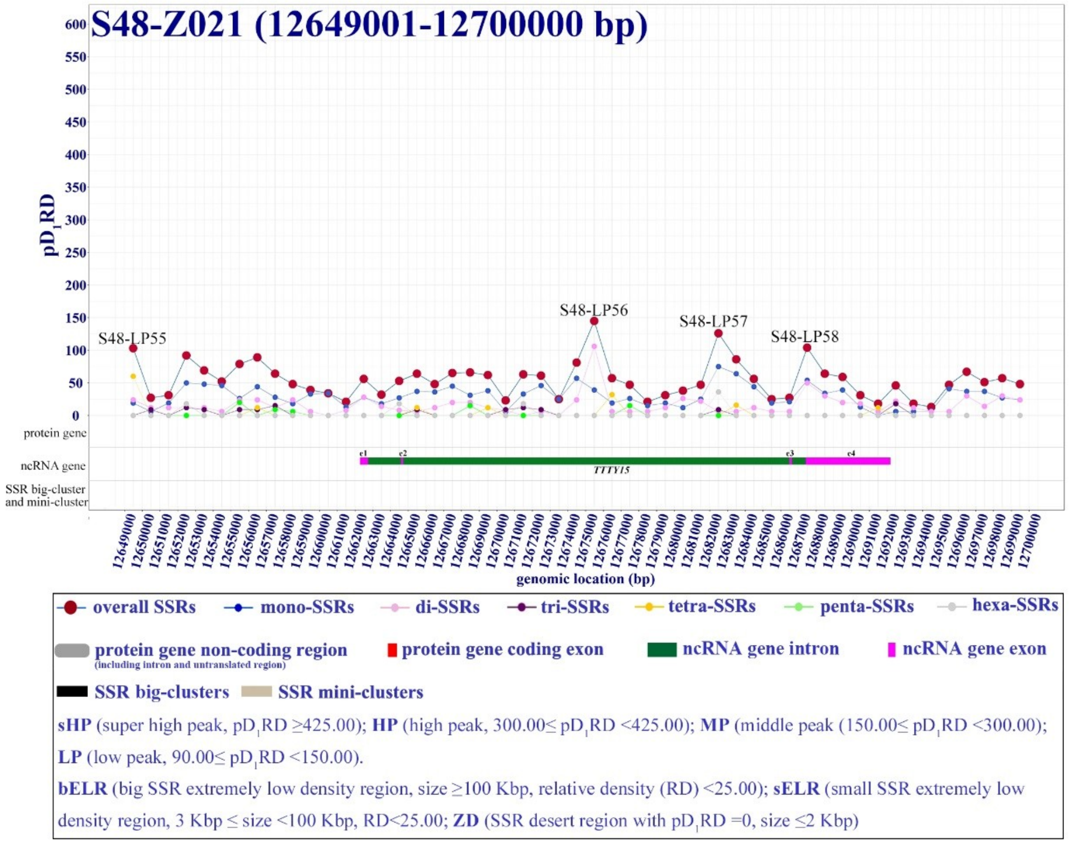

Supplementary Figure 1.249. The SSR position related D_1-relative density (pD_1RD) map of position at 12649001-12700000 bp of human reference Y-DNA (NC_000024.10) at resolution of 1 Kbp.

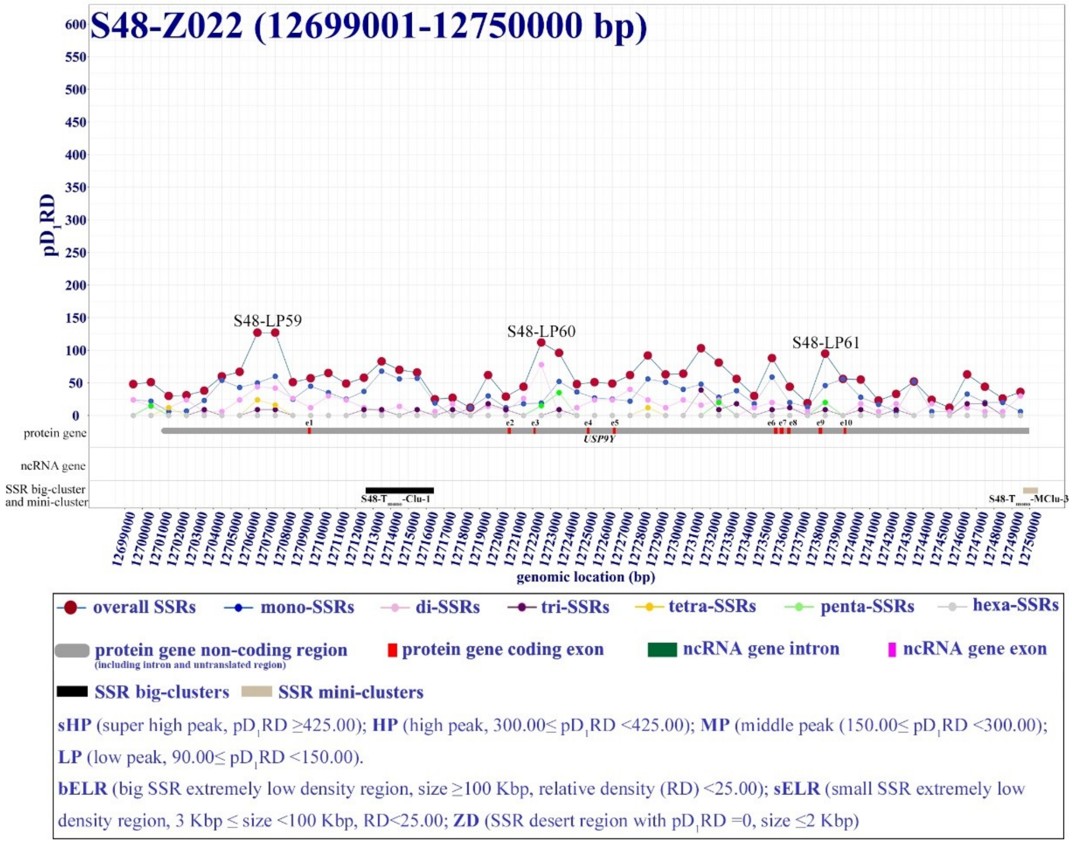

Supplementary Figure 1.250. The SSR position related D_1-relative density (pD_1RD) map of position at 12699001-12750000 bp of human reference Y-DNA (NC_000024.10) at resolution of 1 Kbp.

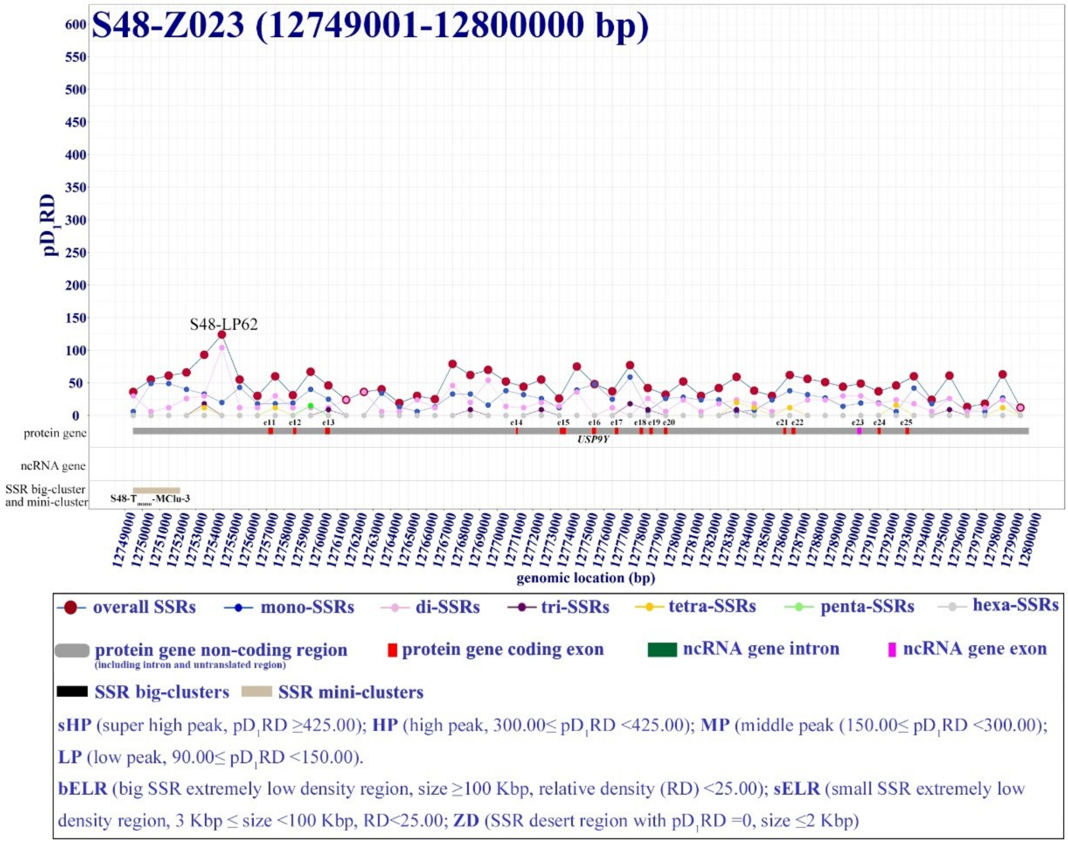

Supplementary Figure 1.251. The SSR position related D_1-relative density (pD_1RD) map of position at 12749001-12800000 bp of human reference Y-DNA (NC_000024.10) at resolution of 1 Kbp.

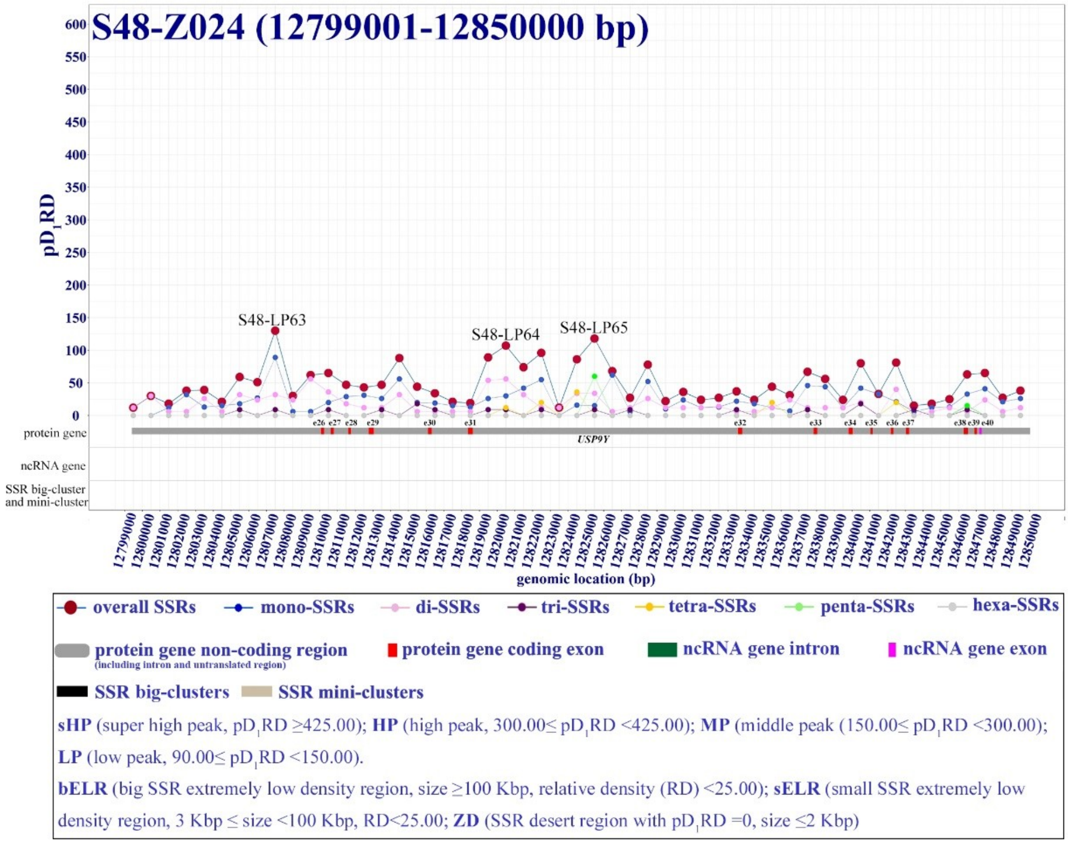

Supplementary Figure 1.252. The SSR position related D_1-relative density (pD_1RD) map of position at 12799001-12850000 bp of human reference Y-DNA (NC_000024.10) at resolution of 1 Kbp.

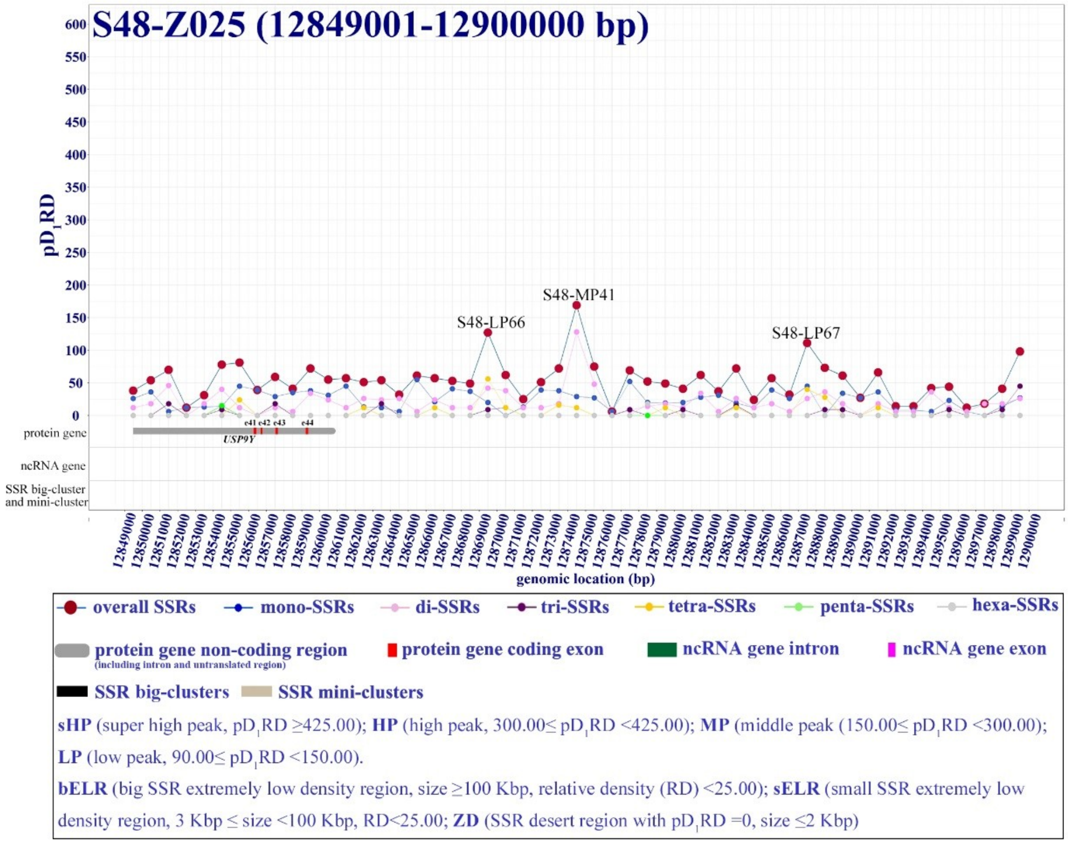

Supplementary Figure 1.253. The SSR position related D_1-relative density (pD_1RD) map of position at 12849001-12900000 bp of human reference Y-DNA (NC_000024.10) at resolution of 1 Kbp.

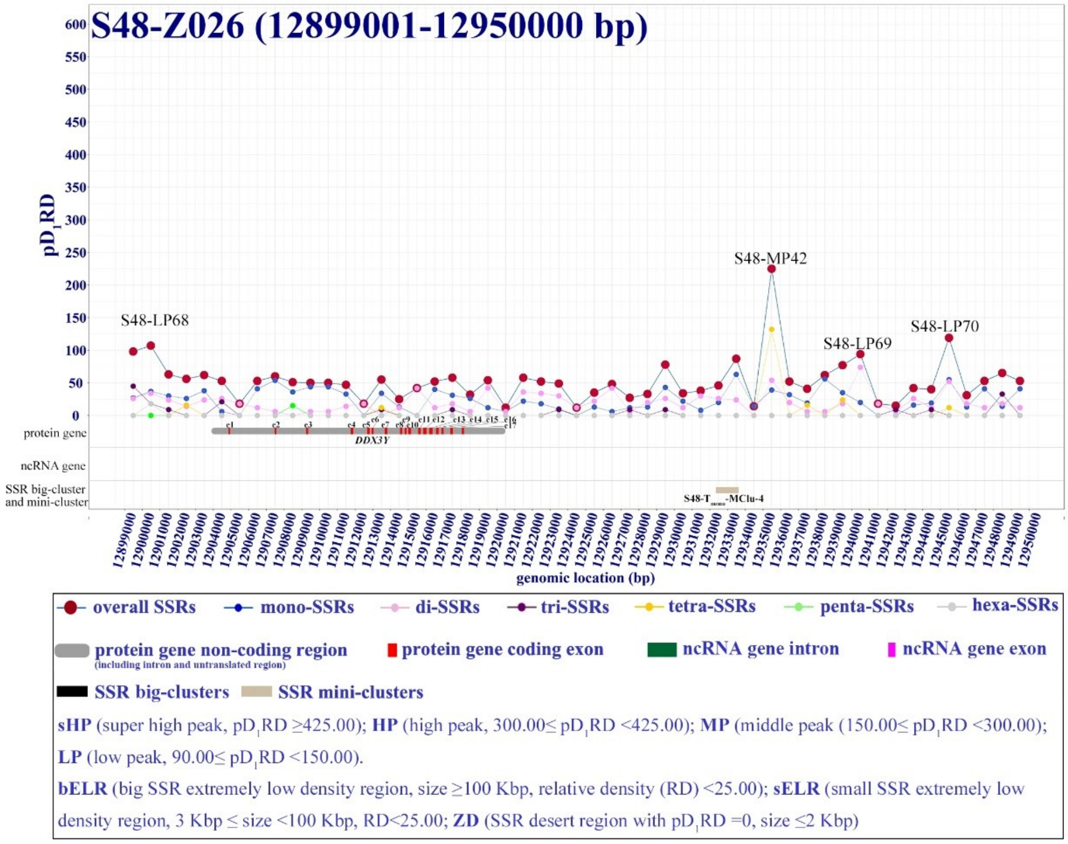

Supplementary Figure 1.254. The SSR position related D_1-relative density (pD_1RD) map of position at 12899001-12950000 bp of human reference Y-DNA (NC_000024.10) at resolution of 1 Kbp.

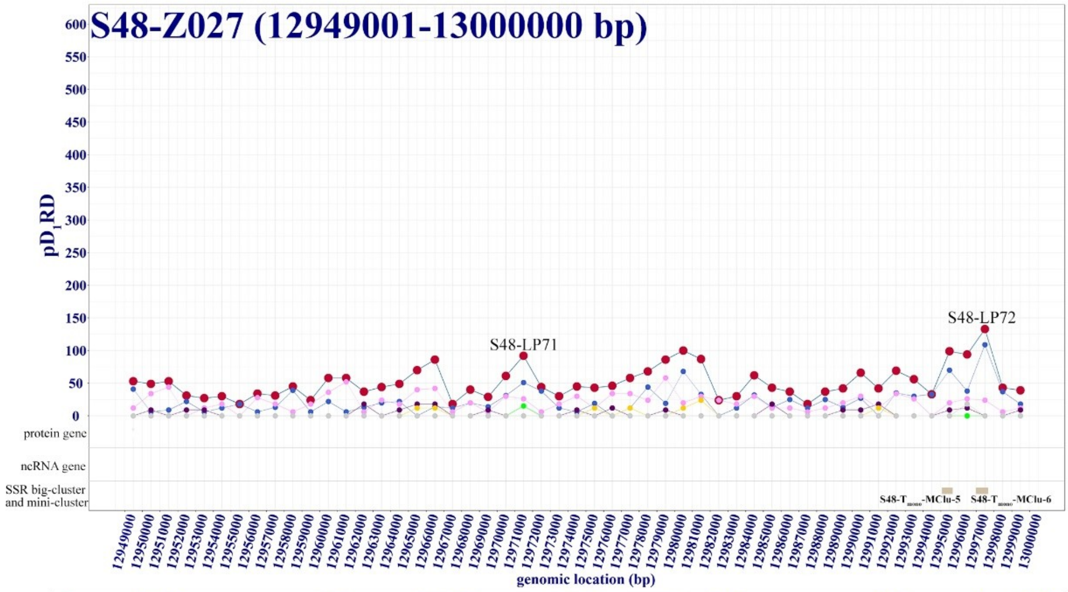

- overall SSRs — mono-SSRs — di-SSRs — tri-SSRs — tetra-SSRs — penta-SSRs — hexa-SSRs

protein gene non-coding region (including intron and untranslated region) | protein gene coding exon | ncRNA gene intron | ncRNA gene exon

SSR big-clusters | SSR mini-clusters

sHP (super high peak, $pD_1RD \geq 425.00$); **HP** (high peak, $300.00 \leq pD_1RD < 425.00$); **MP** (middle peak ($150.00 \leq pD_1RD < 300.00$); **LP** (low peak, $90.00 \leq pD_1RD < 150.00$).

bELR (big SSR extremely low density region, size ≥100 Kbp, relative density (RD) <25.00); **sELR** (small SSR extremely low density region, 3 Kbp ≤ size <100 Kbp, RD<25.00; **ZD** (SSR desert region with $pD_1RD = 0$, size ≤2 Kbp)

Supplementary Figure 1.255. The SSR position related D_1-relative density (pD_1RD) map of position at 12949001-13000000 bp of human reference Y-DNA (NC_000024.10) at resolution of 1 Kbp.

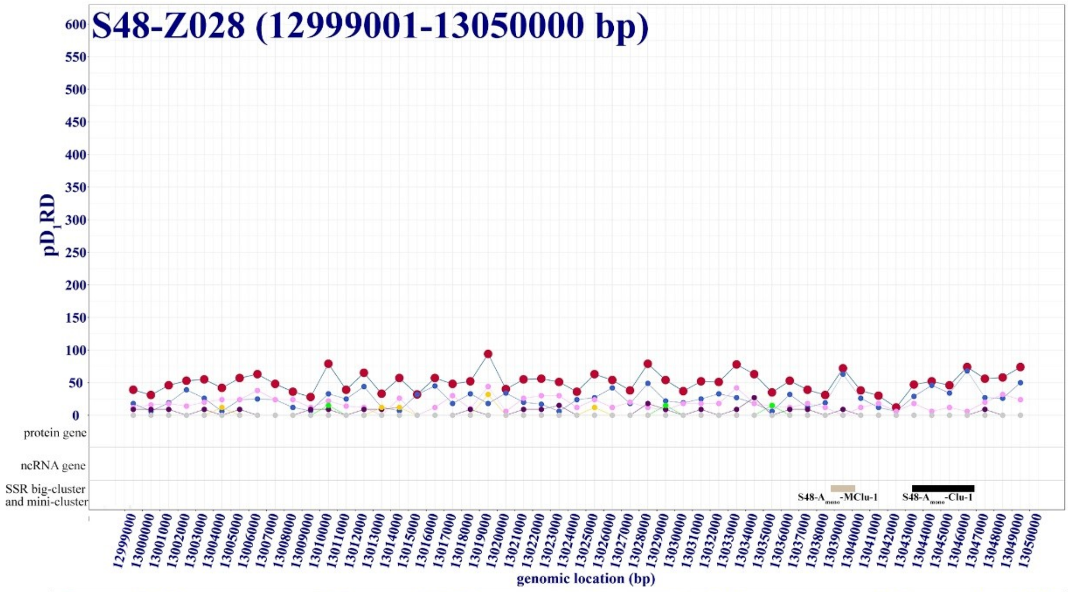

- overall SSRs — mono-SSRs — di-SSRs — tri-SSRs — tetra-SSRs — penta-SSRs — hexa-SSRs

protein gene non-coding region (including intron and untranslated region) | protein gene coding exon | ncRNA gene intron | ncRNA gene exon

SSR big-clusters | SSR mini-clusters

sHP (super high peak, $pD_1RD \geq 425.00$); **HP** (high peak, $300.00 \leq pD_1RD < 425.00$); **MP** (middle peak ($150.00 \leq pD_1RD < 300.00$); **LP** (low peak, $90.00 \leq pD_1RD < 150.00$).

bELR (big SSR extremely low density region, size ≥100 Kbp, relative density (RD) <25.00); **sELR** (small SSR extremely low density region, 3 Kbp ≤ size <100 Kbp, RD<25.00; **ZD** (SSR desert region with $pD_1RD = 0$, size ≤2 Kbp)

Supplementary Figure 1.256. The SSR position related D_1-relative density (pD_1RD) map of position at 12999001-13050000 bp of human reference Y-DNA (NC_000024.10) at resolution of 1 Kbp.

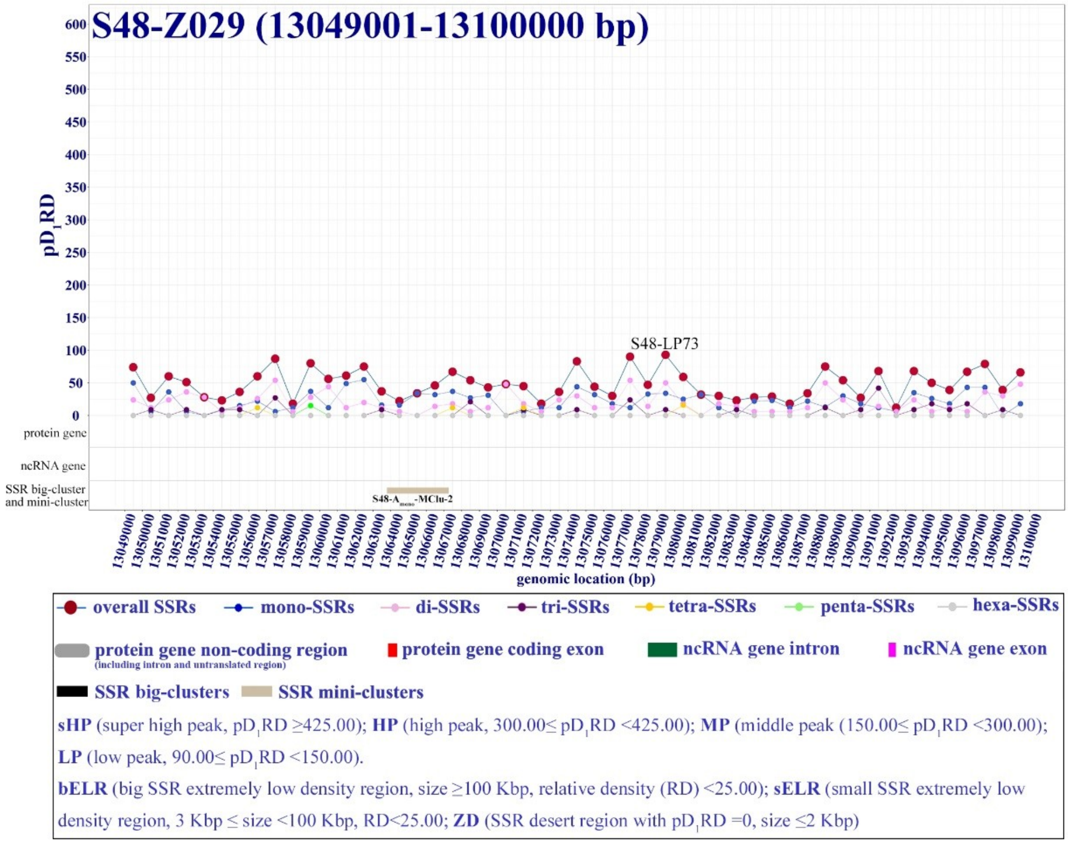

Supplementary Figure 1.257. The SSR position related D_1-relative density (pD_1RD) map of position at 13049001-13100000 bp of human reference Y-DNA (NC_000024.10) at resolution of 1 Kbp.

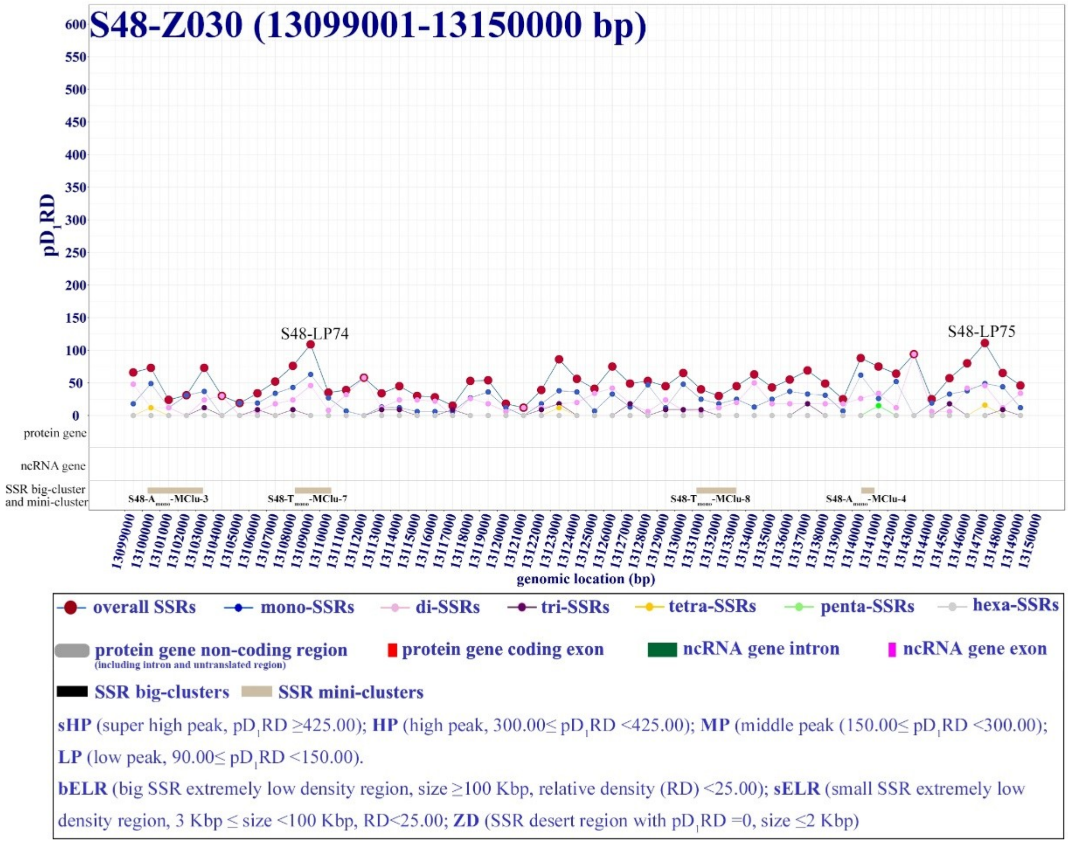

Supplementary Figure 1.258. The SSR position related D_1-relative density (pD_1RD) map of position at 13099001-13150000 bp of human reference Y-DNA (NC_000024.10) at resolution of 1 Kbp.

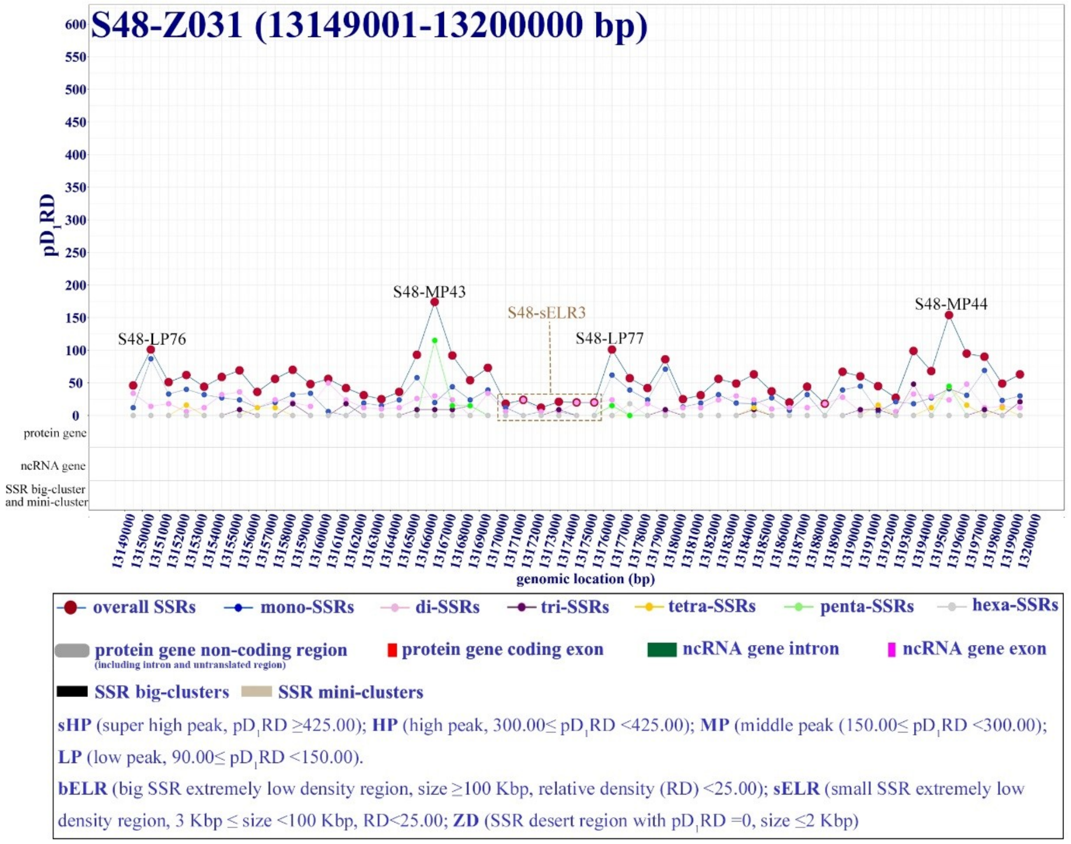

Supplementary Figure 1.259. The SSR position related D_1-relative density (pD_1RD) map of position at 13149001-13200000 bp of human reference Y-DNA (NC_000024.10) at resolution of 1 Kbp.

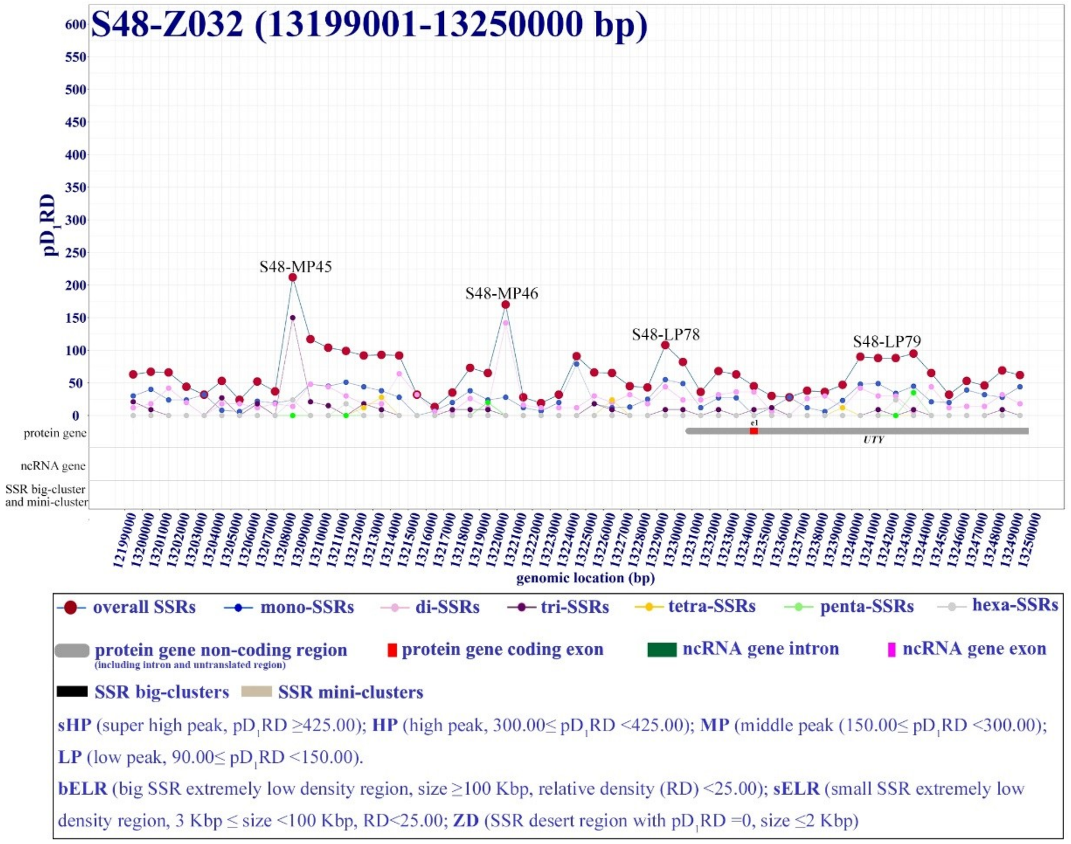

Supplementary Figure 1.260. The SSR position related D_1-relative density (pD_1RD) map of position at 13199001-13250000 bp of human reference Y-DNA (NC_000024.10) at resolution of 1 Kbp.

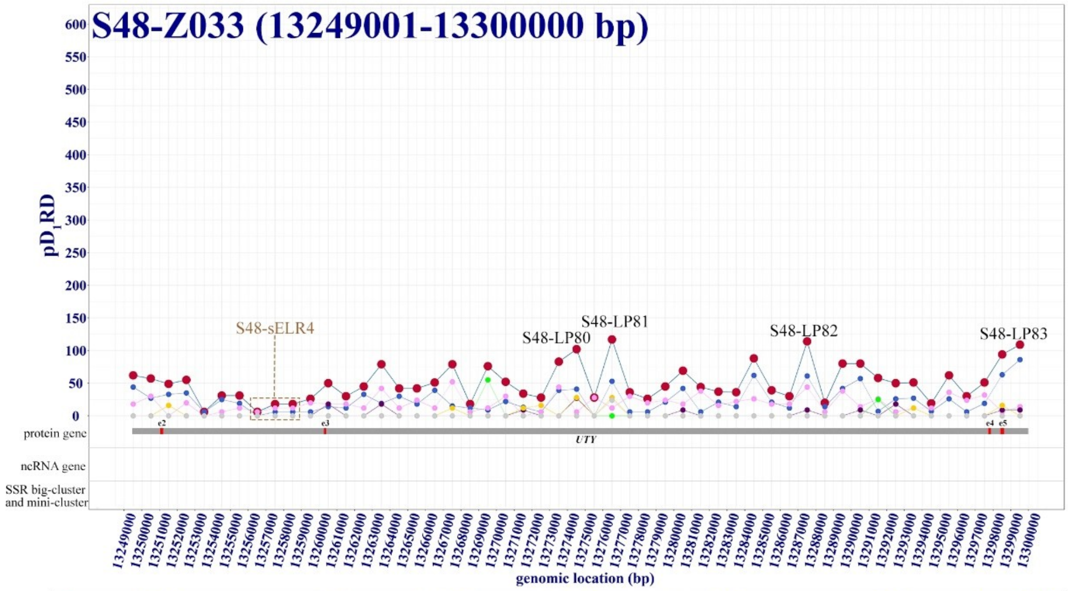

overall SSRs | mono-SSRs | di-SSRs | tri-SSRs | tetra-SSRs | penta-SSRs | hexa-SSRs

protein gene non-coding region (including intron and untranslated region) | protein gene coding exon | ncRNA gene intron | ncRNA gene exon

SSR big-clusters | SSR mini-clusters

sHP (super high peak, $pD_1RD \geq 425.00$); **HP** (high peak, $300.00 \leq pD_1RD < 425.00$); **MP** (middle peak ($150.00 \leq pD_1RD < 300.00$); **LP** (low peak, $90.00 \leq pD_1RD < 150.00$).

bELR (big SSR extremely low density region, size ≥100 Kbp, relative density (RD) <25.00); **sELR** (small SSR extremely low density region, 3 Kbp ≤ size <100 Kbp, RD<25.00; **ZD** (SSR desert region with $pD_1RD = 0$, size ≤2 Kbp)

Supplementary Figure 1.261. The SSR position related D_1-relative density (pD_1RD) map of position at 13249001-13300000 bp of human reference Y-DNA (NC_000024.10) at resolution of 1 Kbp.

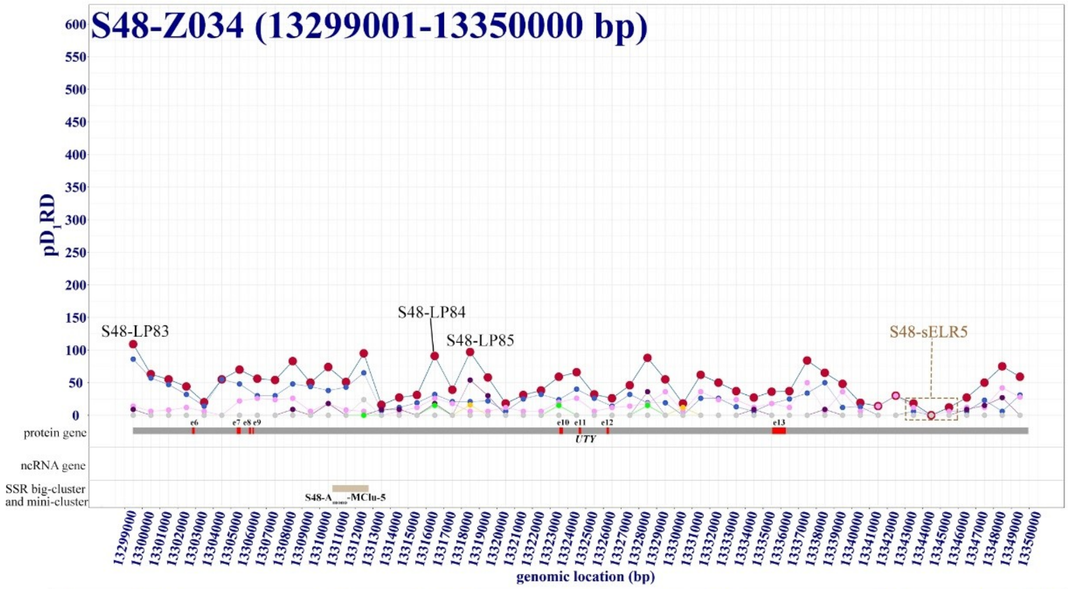

overall SSRs | mono-SSRs | di-SSRs | tri-SSRs | tetra-SSRs | penta-SSRs | hexa-SSRs

protein gene non-coding region (including intron and untranslated region) | protein gene coding exon | ncRNA gene intron | ncRNA gene exon

SSR big-clusters | SSR mini-clusters

sHP (super high peak, $pD_1RD \geq 425.00$); **HP** (high peak, $300.00 \leq pD_1RD < 425.00$); **MP** (middle peak ($150.00 \leq pD_1RD < 300.00$); **LP** (low peak, $90.00 \leq pD_1RD < 150.00$).

bELR (big SSR extremely low density region, size ≥100 Kbp, relative density (RD) <25.00); **sELR** (small SSR extremely low density region, 3 Kbp ≤ size <100 Kbp, RD<25.00; **ZD** (SSR desert region with $pD_1RD = 0$, size ≤2 Kbp)

Supplementary Figure 1.262. The SSR position related D_1-relative density (pD_1RD) map of position at 13299001-13350000 bp of human reference Y-DNA (NC_000024.10) at resolution of 1 Kbp.

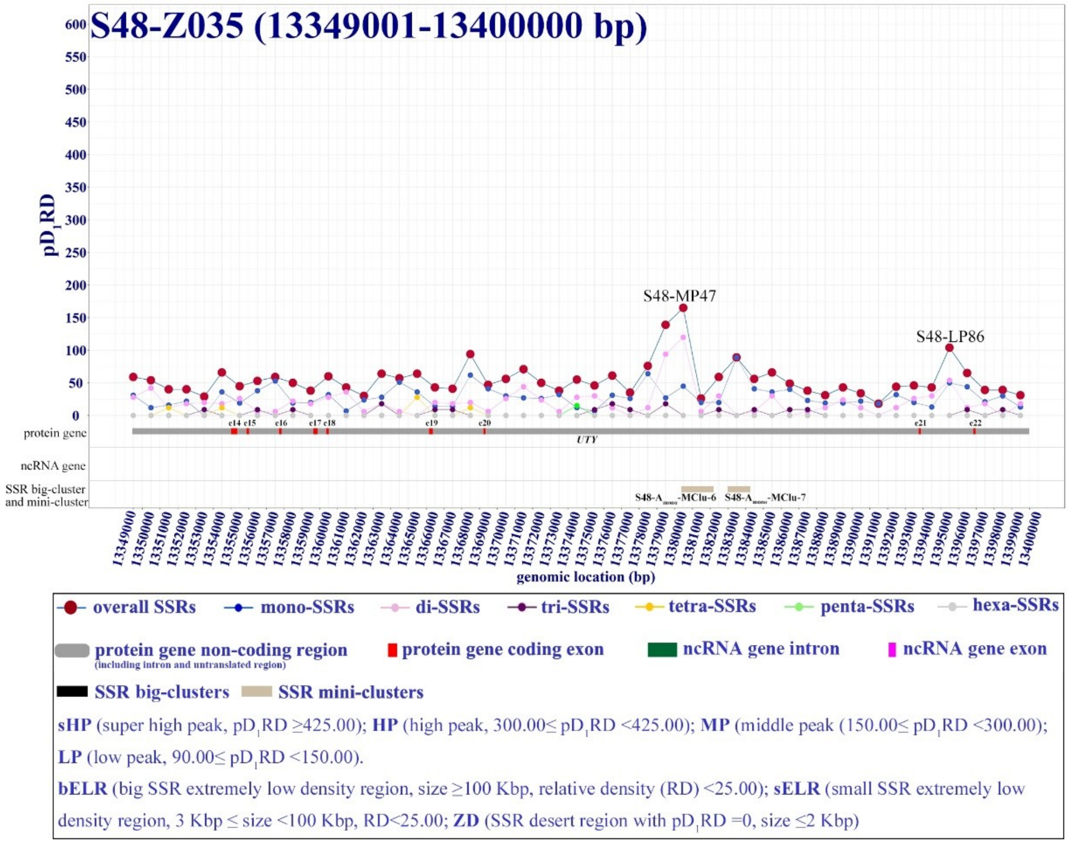

Supplementary Figure 1.263. The SSR position related D_1-relative density (pD_1RD) map of position at 13349001-13400000 bp of human reference Y-DNA (NC_000024.10) at resolution of 1 Kbp.

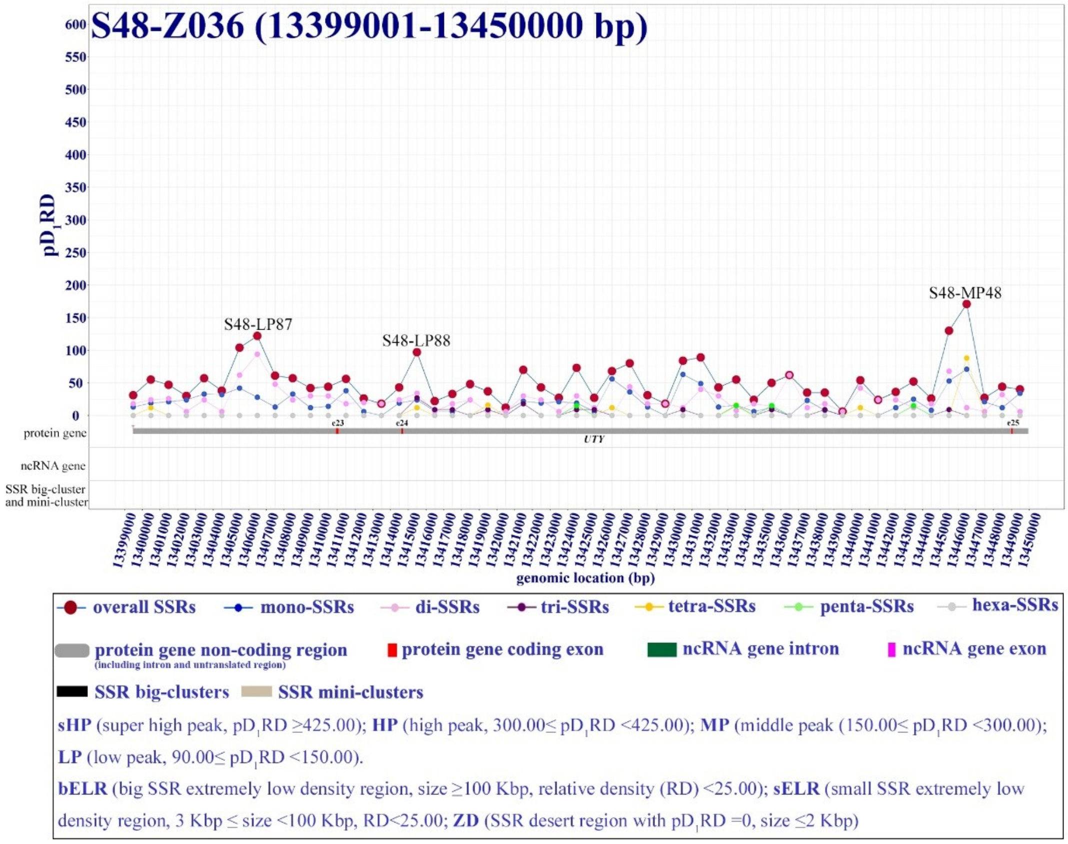

Supplementary Figure 1.264. The SSR position related D_1-relative density (pD_1RD) map of position at 13399001-13450000 bp of human reference Y-DNA (NC_000024.10) at resolution of 1 Kbp.

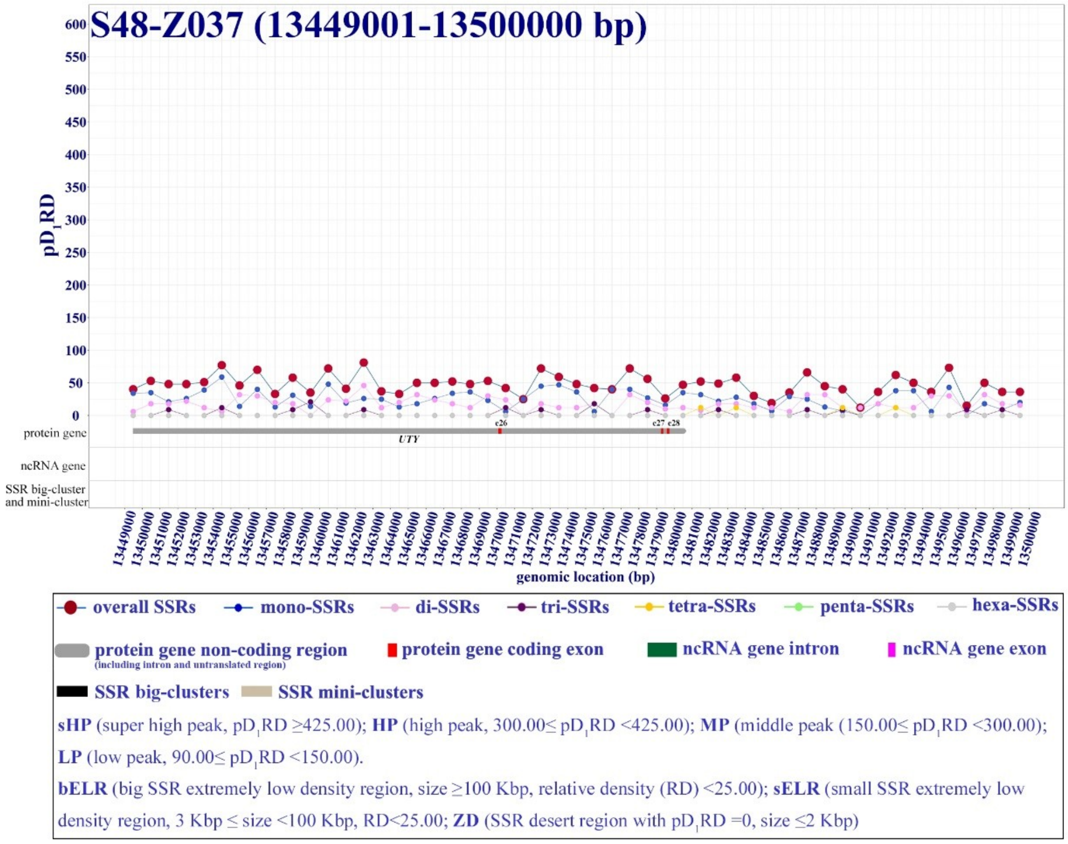

Supplementary Figure 1.265. The SSR position related D_1-relative density (pD_1RD) map of position at 13449001-13500000 bp of human reference Y-DNA (NC_000024.10) at resolution of 1 Kbp.

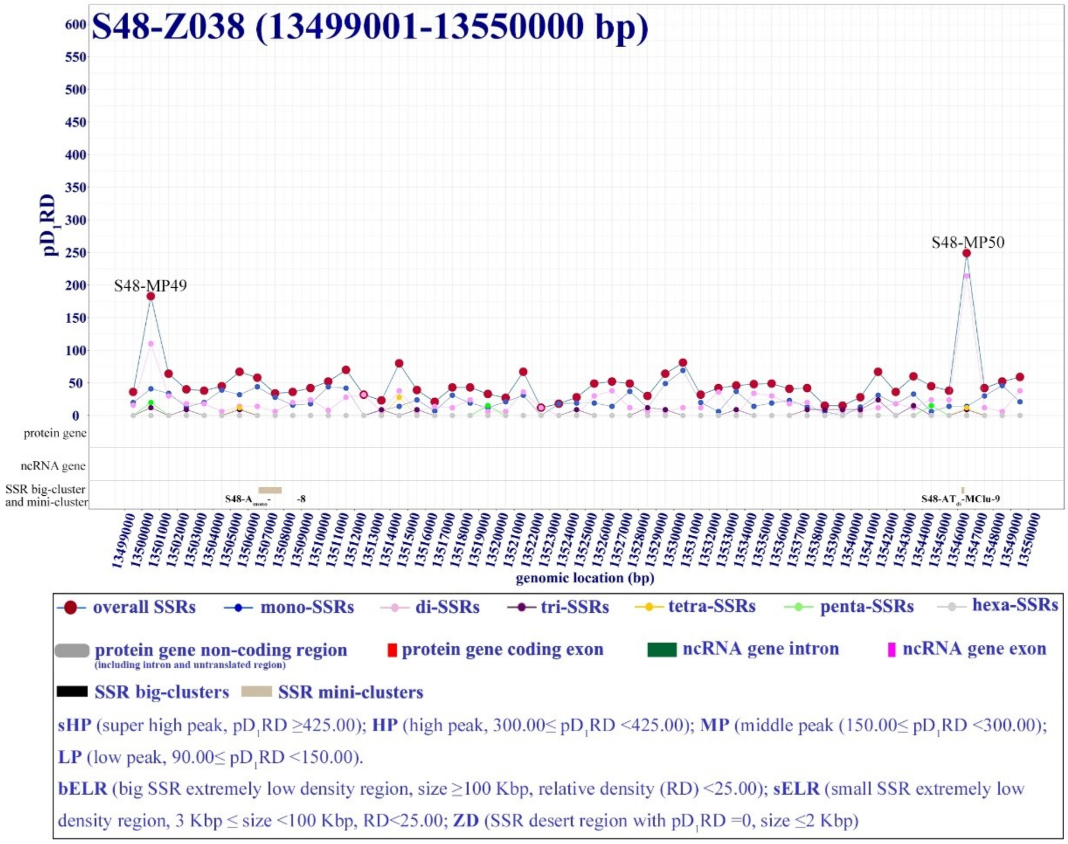

Supplementary Figure 1.266. The SSR position related D_1-relative density (pD_1RD) map of position at 13499001-13550000 bp of human reference Y-DNA (NC_000024.10) at resolution of 1 Kbp.

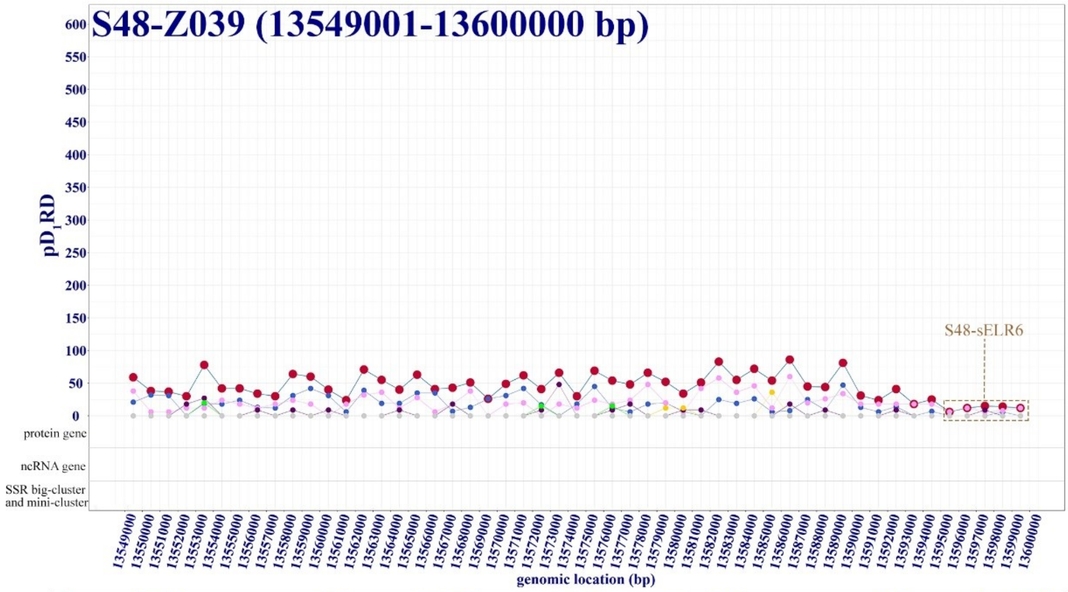

overall SSRs | mono-SSRs | di-SSRs | tri-SSRs | tetra-SSRs | penta-SSRs | hexa-SSRs

protein gene non-coding region (including intron and untranslated region) | protein gene coding exon | ncRNA gene intron | ncRNA gene exon

SSR big-clusters | SSR mini-clusters

sHP (super high peak, $pD_1RD \geq 425.00$); **HP** (high peak, $300.00 \leq pD_1RD < 425.00$); **MP** (middle peak ($150.00 \leq pD_1RD < 300.00$); **LP** (low peak, $90.00 \leq pD_1RD < 150.00$).

bELR (big SSR extremely low density region, size ≥100 Kbp, relative density (RD) <25.00); **sELR** (small SSR extremely low density region, 3 Kbp ≤ size <100 Kbp, RD<25.00; **ZD** (SSR desert region with $pD_1RD = 0$, size ≤2 Kbp)

Supplementary Figure 1.267. The SSR position related D_1-relative density (pD_1RD) map of position at 13549001-13600000 bp of human reference Y-DNA (NC_000024.10) at resolution of 1 Kbp.

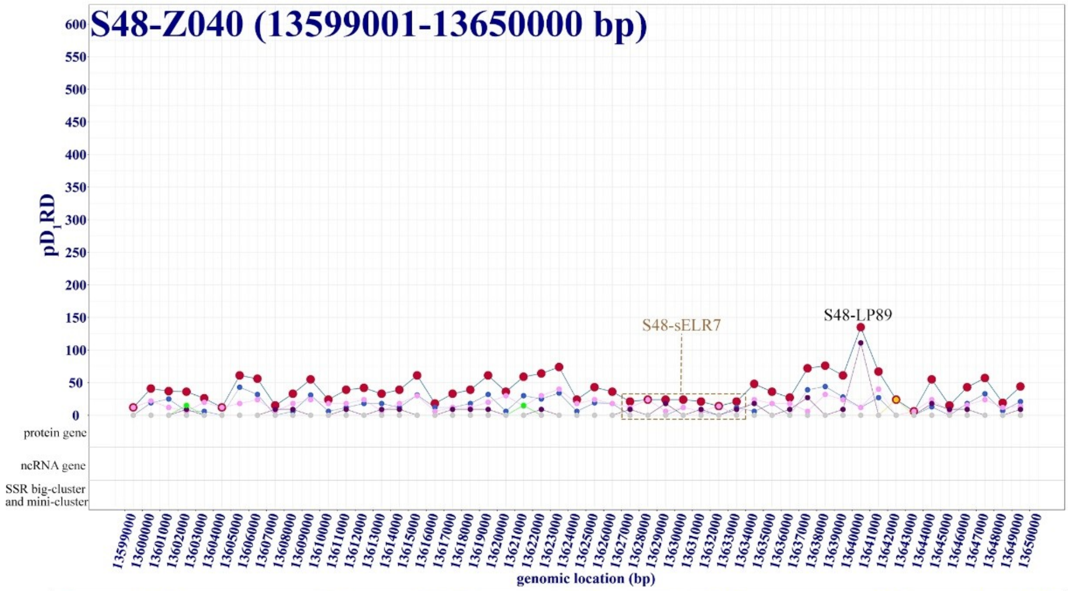

overall SSRs | mono-SSRs | di-SSRs | tri-SSRs | tetra-SSRs | penta-SSRs | hexa-SSRs

protein gene non-coding region (including intron and untranslated region) | protein gene coding exon | ncRNA gene intron | ncRNA gene exon

SSR big-clusters | SSR mini-clusters

sHP (super high peak, $pD_1RD \geq 425.00$); **HP** (high peak, $300.00 \leq pD_1RD < 425.00$); **MP** (middle peak ($150.00 \leq pD_1RD < 300.00$); **LP** (low peak, $90.00 \leq pD_1RD < 150.00$).

bELR (big SSR extremely low density region, size ≥100 Kbp, relative density (RD) <25.00); **sELR** (small SSR extremely low density region, 3 Kbp ≤ size <100 Kbp, RD<25.00; **ZD** (SSR desert region with $pD_1RD = 0$, size ≤2 Kbp)

Supplementary Figure 1.268. The SSR position related D_1-relative density (pD_1RD) map of position at 13599001-13650000 bp of human reference Y-DNA (NC_000024.10) at resolution of 1 Kbp.

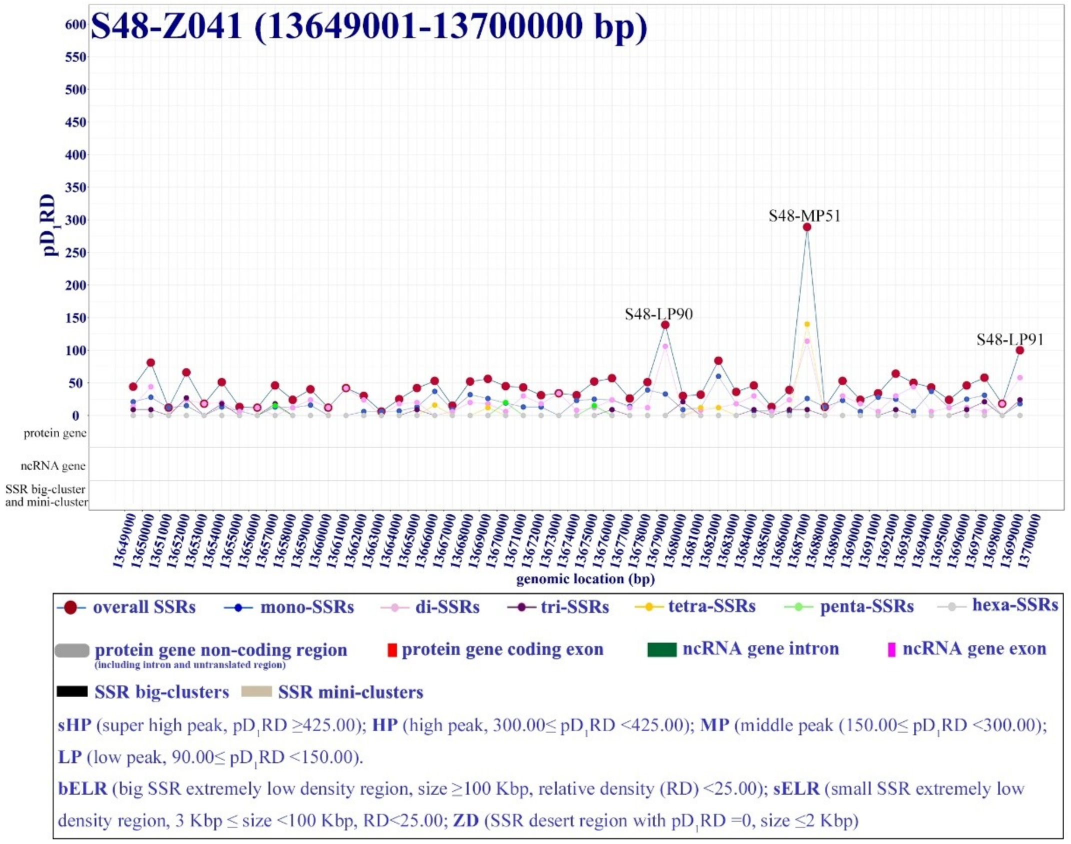

Supplementary Figure 1.269. The SSR position related D_1-relative density (pD_1RD) map of position at 13649001-13700000 bp of human reference Y-DNA (NC_000024.10) at resolution of 1 Kbp.

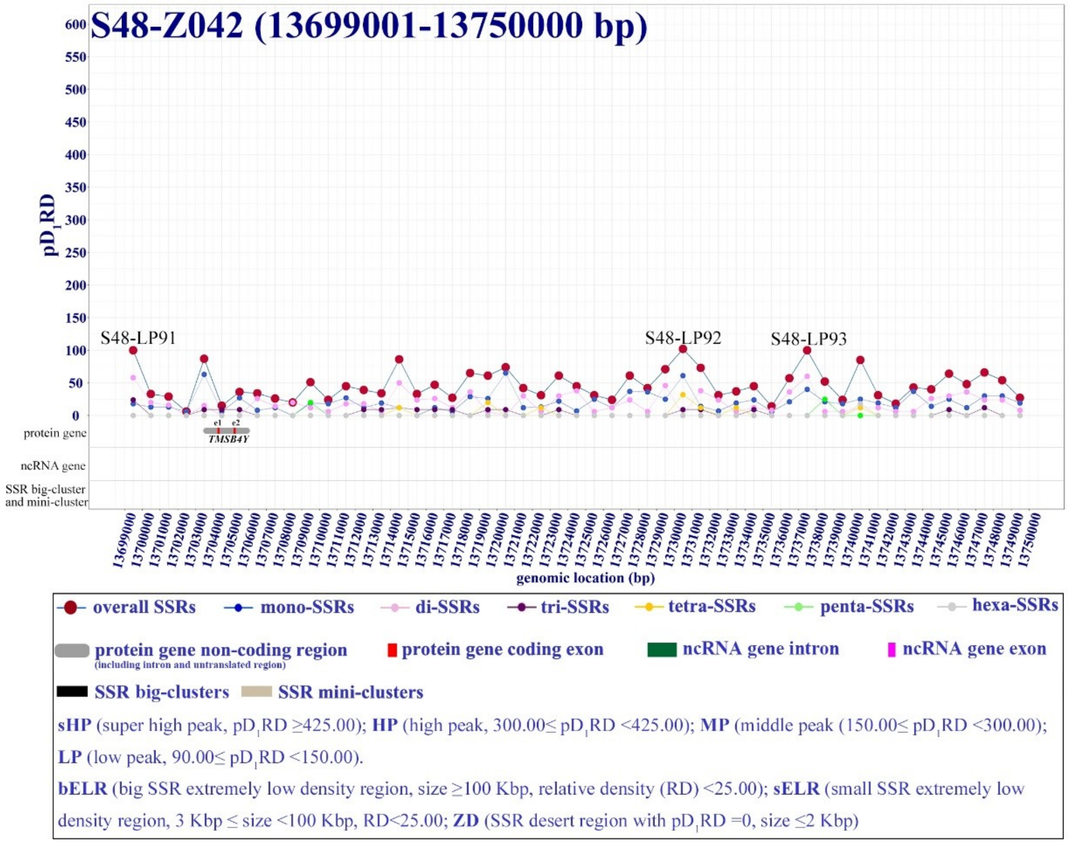

Supplementary Figure 1.270. The SSR position related D_1-relative density (pD_1RD) map of position at 13699001-13750000 bp of human reference Y-DNA (NC_000024.10) at resolution of 1 Kbp.

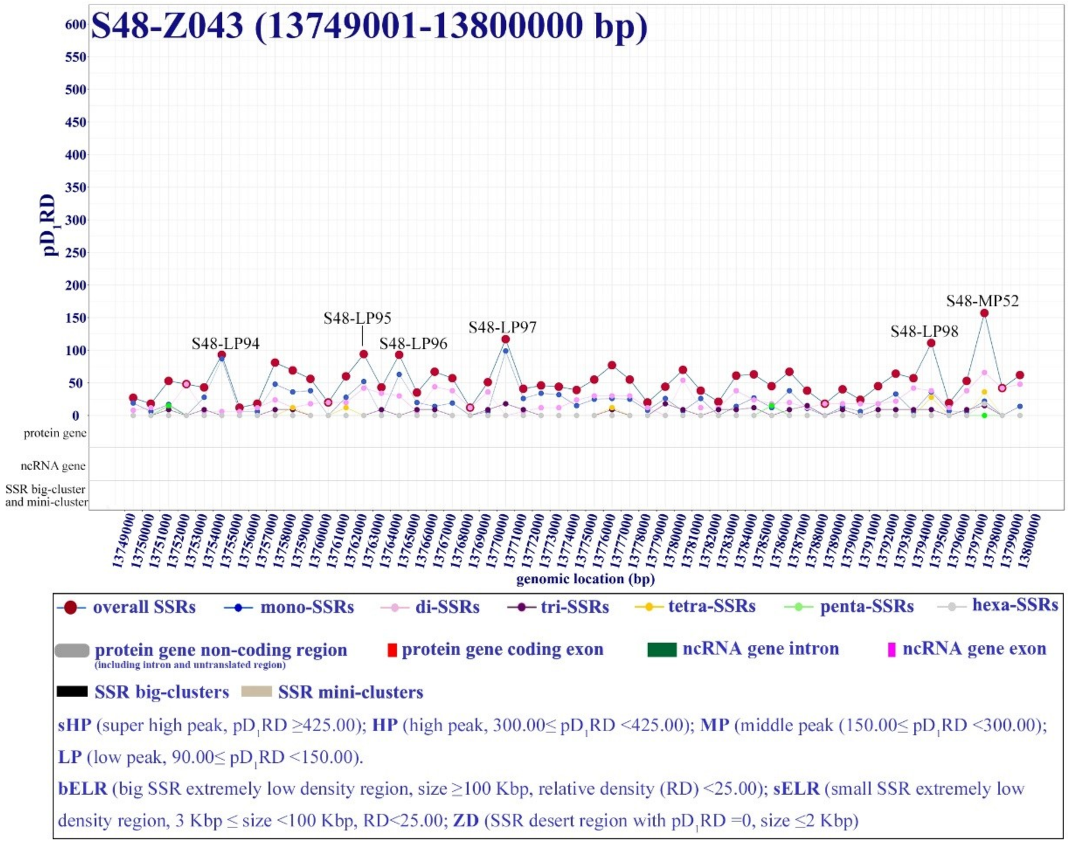

Supplementary Figure 1.271. The SSR position related D_1-relative density (pD_1RD) map of position at 13749001-13800000 bp of human reference Y-DNA (NC_000024.10) at resolution of 1 Kbp.

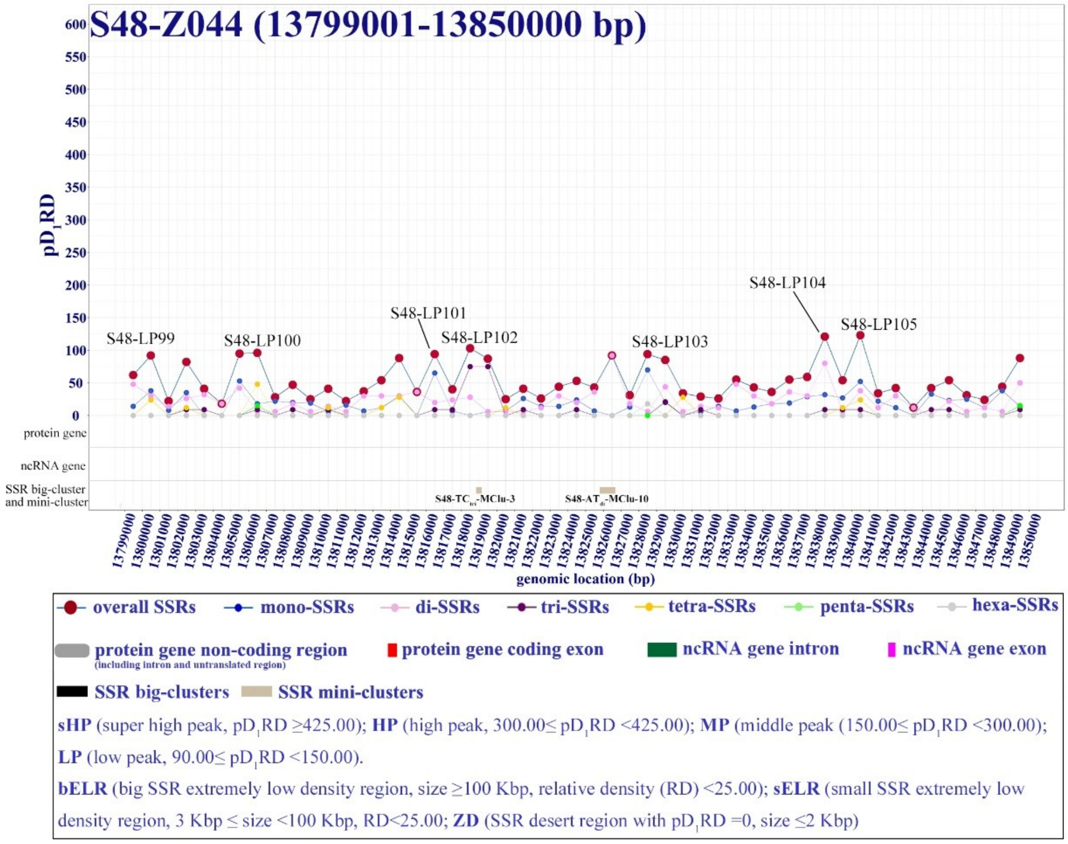

Supplementary Figure 1.272. The SSR position related D_1-relative density (pD_1RD) map of position at 13799001-13850000 bp of human reference Y-DNA (NC_000024.10) at resolution of 1 Kbp.

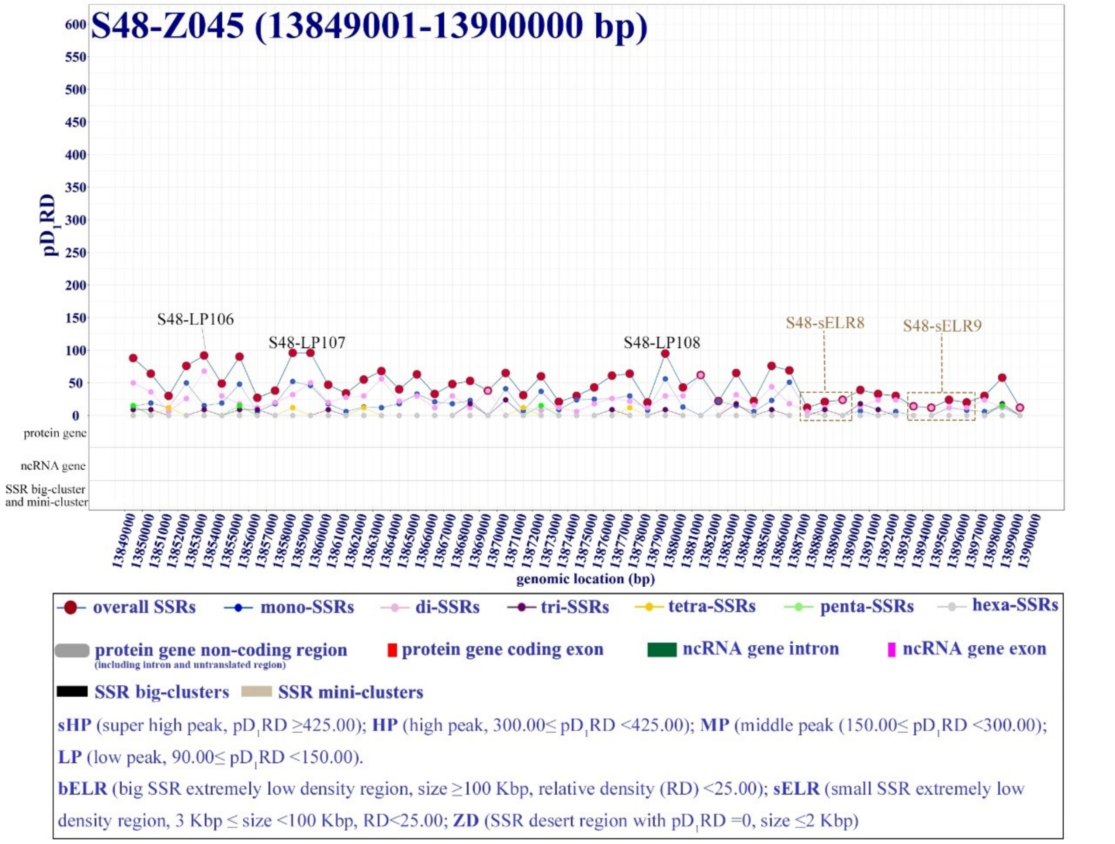

Supplementary Figure 1.273. The SSR position related D_1-relative density (pD_1RD) map of position at 13849001-13900000 bp of human reference Y-DNA (NC_000024.10) at resolution of 1 Kbp.

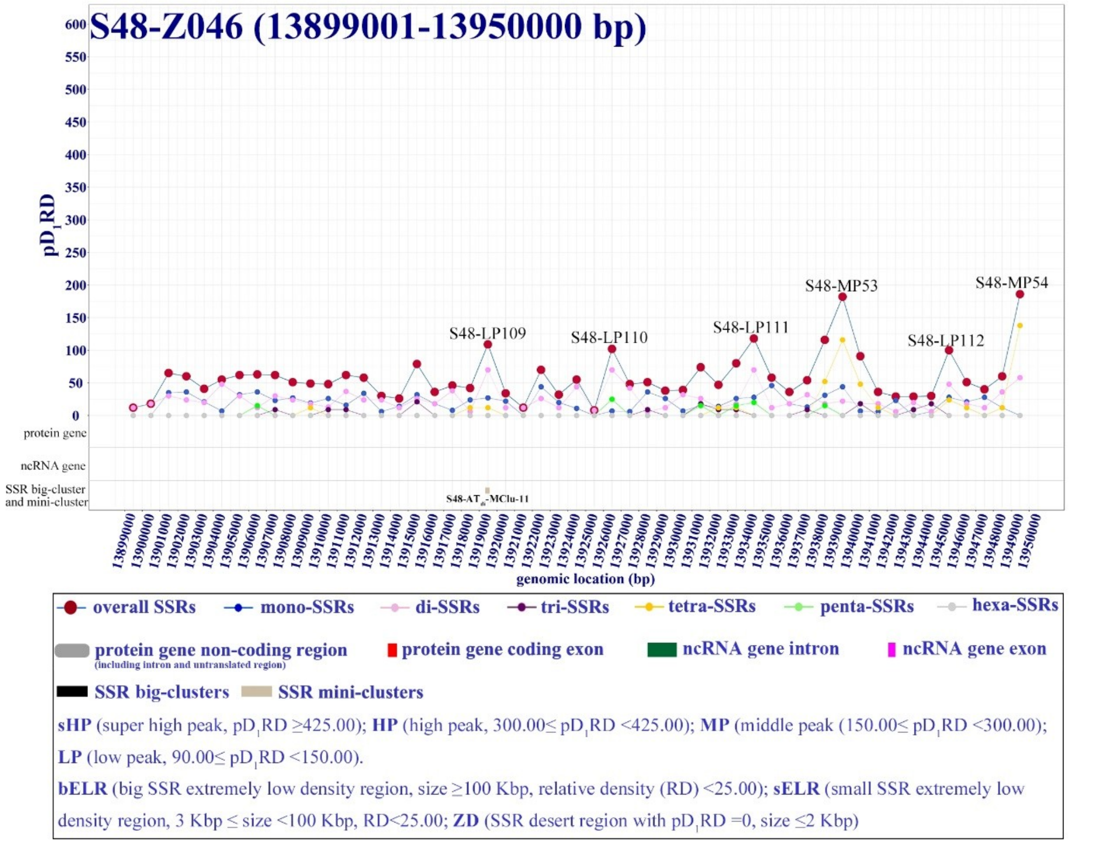

Supplementary Figure 1.274. The SSR position related D_1-relative density (pD_1RD) map of position at 13899001-13950000 bp of human reference Y-DNA (NC_000024.10) at resolution of 1 Kbp.

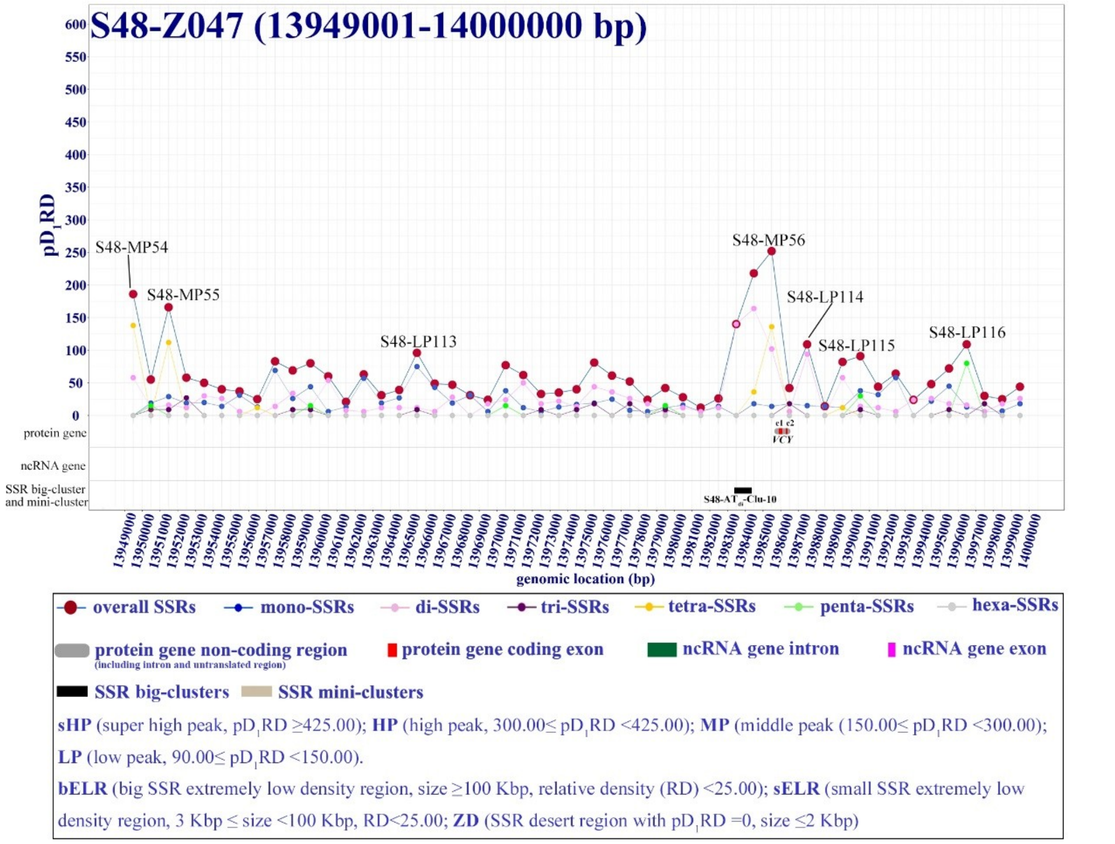

Supplementary Figure 1.275. The SSR position related D_1-relative density (pD_1RD) map of position at 13949001-14000000 bp of human reference Y-DNA (NC_000024.10) at resolution of 1 Kbp.

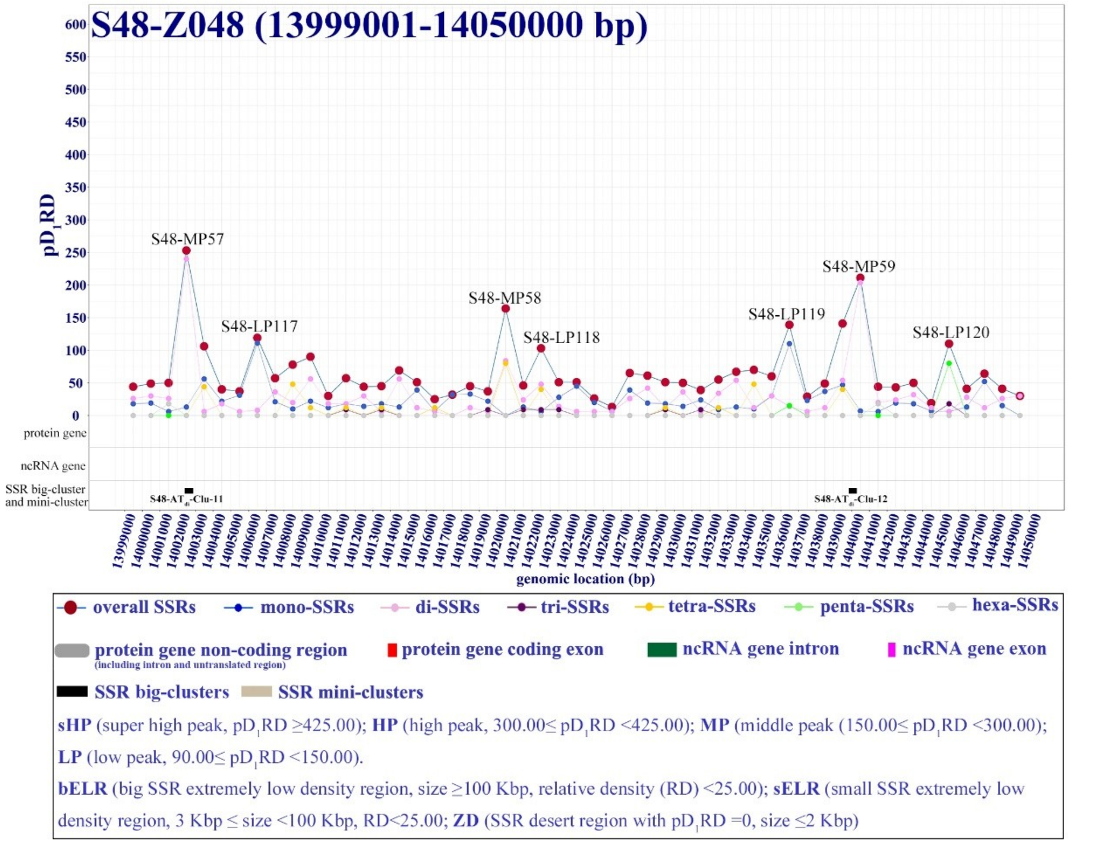

Supplementary Figure 1.276. The SSR position related D_1-relative density (pD_1RD) map of position at 13999001-14050000 bp of human reference Y-DNA (NC_000024.10) at resolution of 1 Kbp.

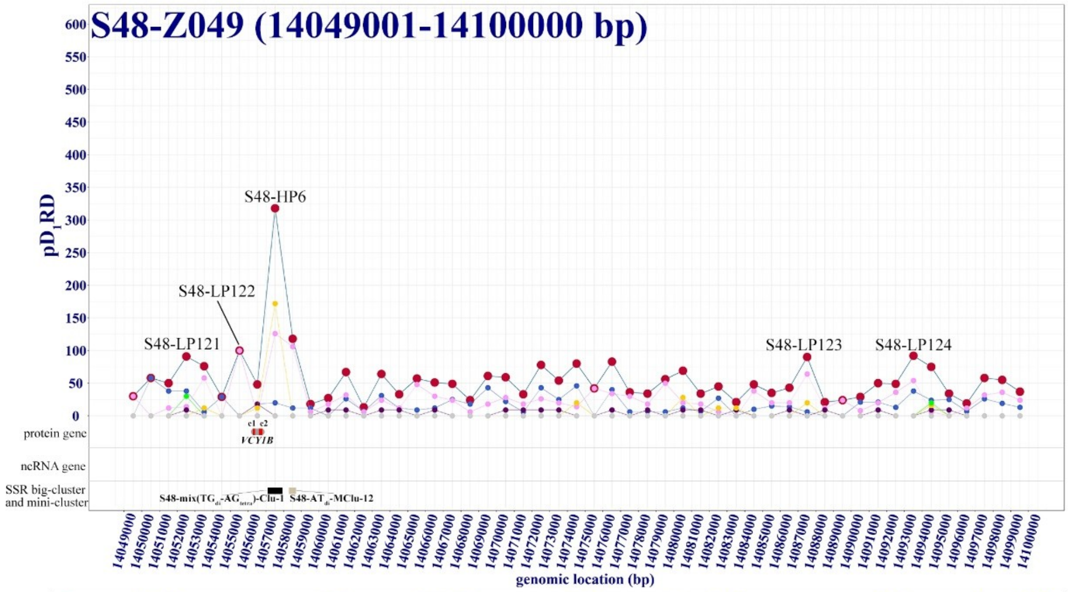

overall SSRs | mono-SSRs | di-SSRs | tri-SSRs | tetra-SSRs | penta-SSRs | hexa-SSRs

protein gene non-coding region (including intron and untranslated region) | protein gene coding exon | ncRNA gene intron | ncRNA gene exon

SSR big-clusters | SSR mini-clusters

sHP (super high peak, $pD_1RD \geq 425.00$); **HP** (high peak, $300.00 \leq pD_1RD < 425.00$); **MP** (middle peak ($150.00 \leq pD_1RD < 300.00$); **LP** (low peak, $90.00 \leq pD_1RD < 150.00$).

bELR (big SSR extremely low density region, size ≥100 Kbp, relative density (RD) <25.00); **sELR** (small SSR extremely low density region, 3 Kbp ≤ size <100 Kbp, RD<25.00; **ZD** (SSR desert region with $pD_1RD = 0$, size ≤2 Kbp)

Supplementary Figure 1.277. The SSR position related D_1-relative density (pD_1RD) map of position at 14049001-14100000 bp of human reference Y-DNA (NC_000024.10) at resolution of 1 Kbp.

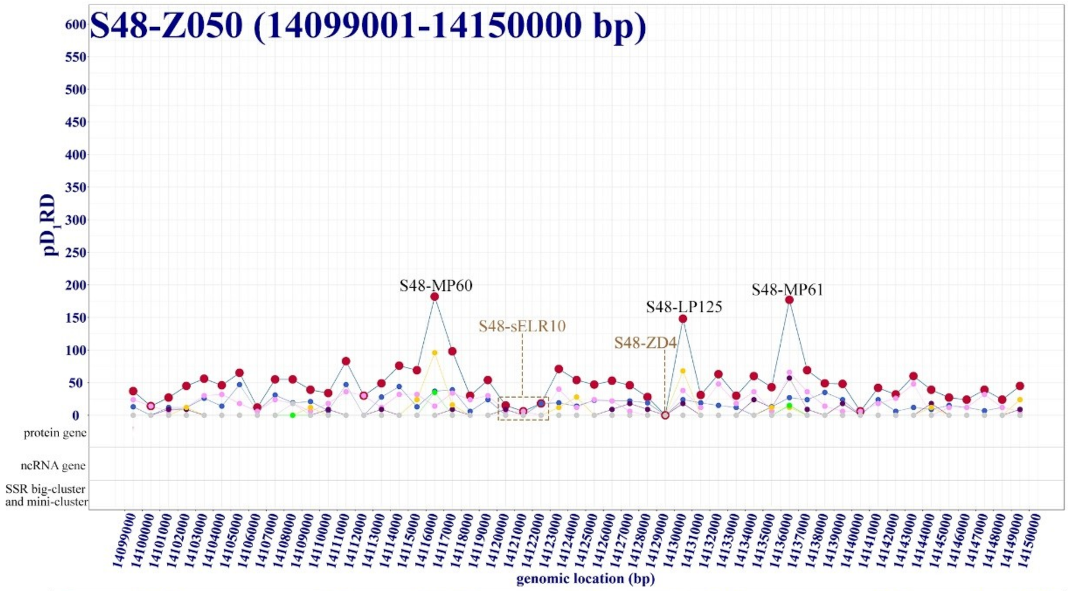

overall SSRs | mono-SSRs | di-SSRs | tri-SSRs | tetra-SSRs | penta-SSRs | hexa-SSRs

protein gene non-coding region (including intron and untranslated region) | protein gene coding exon | ncRNA gene intron | ncRNA gene exon

SSR big-clusters | SSR mini-clusters

sHP (super high peak, $pD_1RD \geq 425.00$); **HP** (high peak, $300.00 \leq pD_1RD < 425.00$); **MP** (middle peak ($150.00 \leq pD_1RD < 300.00$); **LP** (low peak, $90.00 \leq pD_1RD < 150.00$).

bELR (big SSR extremely low density region, size ≥100 Kbp, relative density (RD) <25.00); **sELR** (small SSR extremely low density region, 3 Kbp ≤ size <100 Kbp, RD<25.00; **ZD** (SSR desert region with $pD_1RD = 0$, size ≤2 Kbp)

Supplementary Figure 1.278. The SSR position related D_1-relative density (pD_1RD) map of position at 14099001-14150000 bp of human reference Y-DNA (NC_000024.10) at resolution of 1 Kbp.

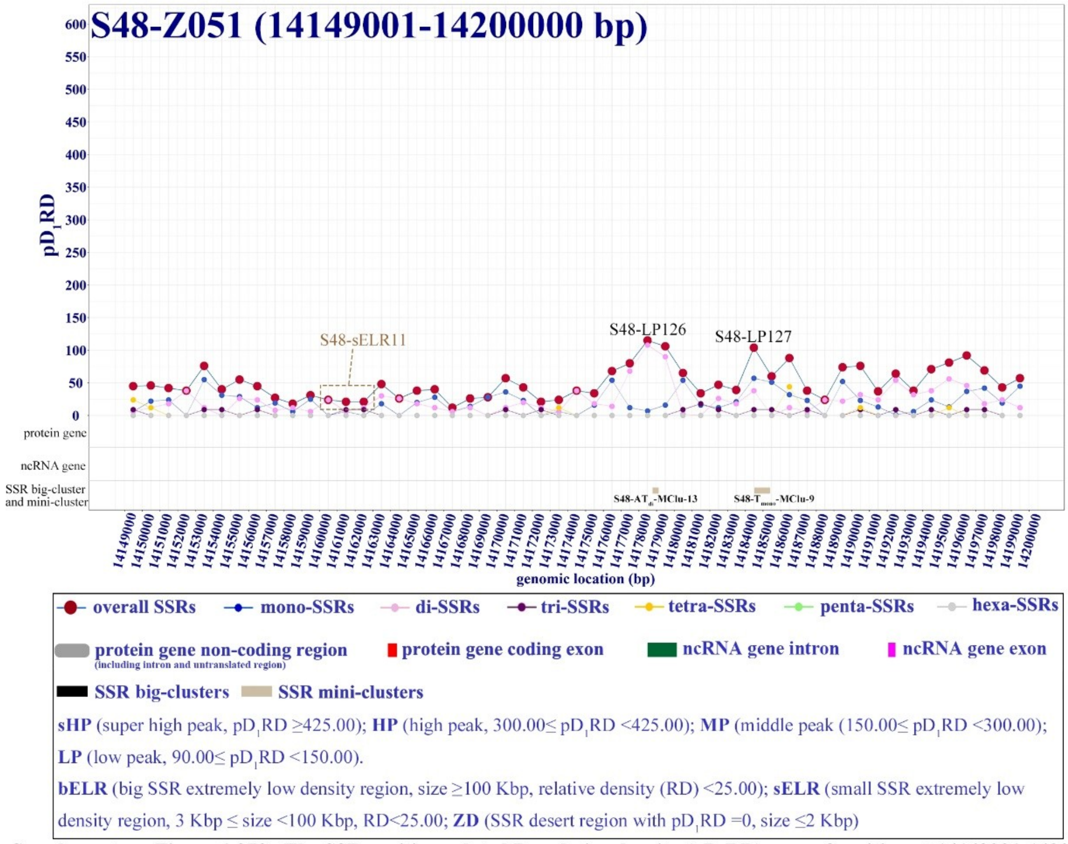

Supplementary Figure 1.279. The SSR position related D_1-relative density (pD_1RD) map of position at 14149001-14200000 bp of human reference Y-DNA (NC_000024.10) at resolution of 1 Kbp.

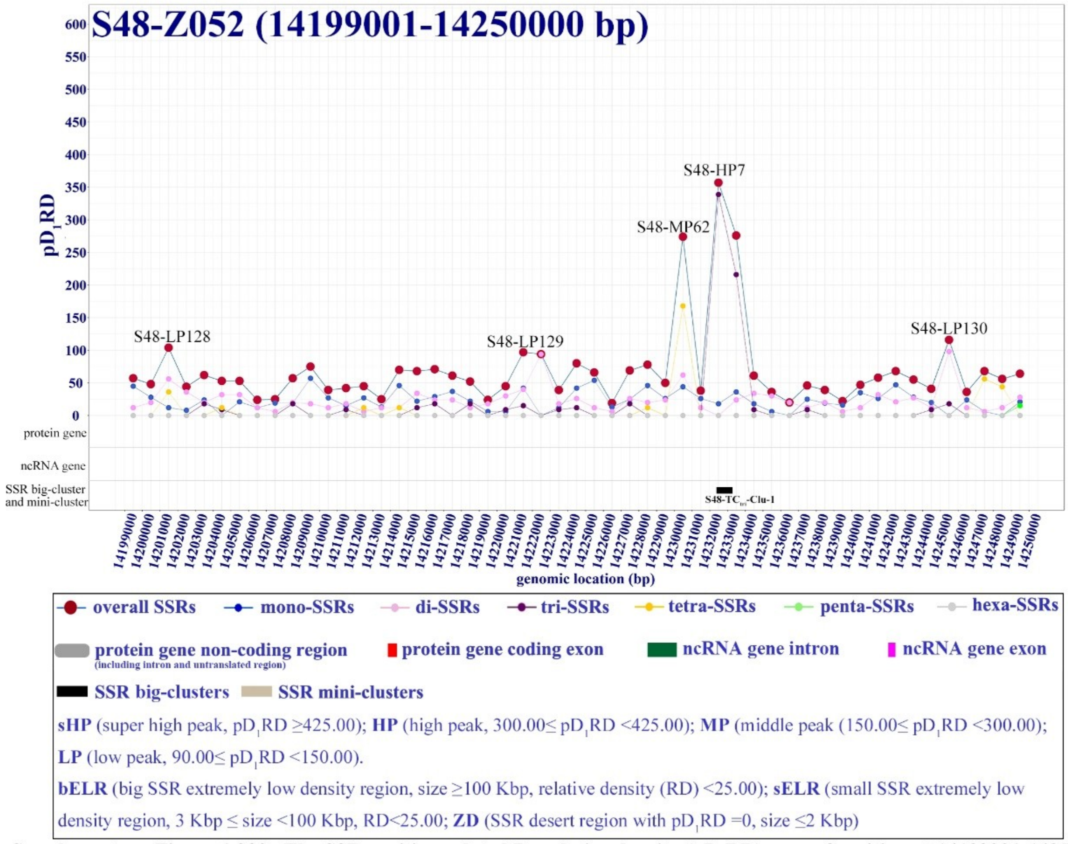

Supplementary Figure 1.280. The SSR position related D_1-relative density (pD_1RD) map of position at 14199001-14250000 bp of human reference Y-DNA (NC_000024.10) at resolution of 1 Kbp.

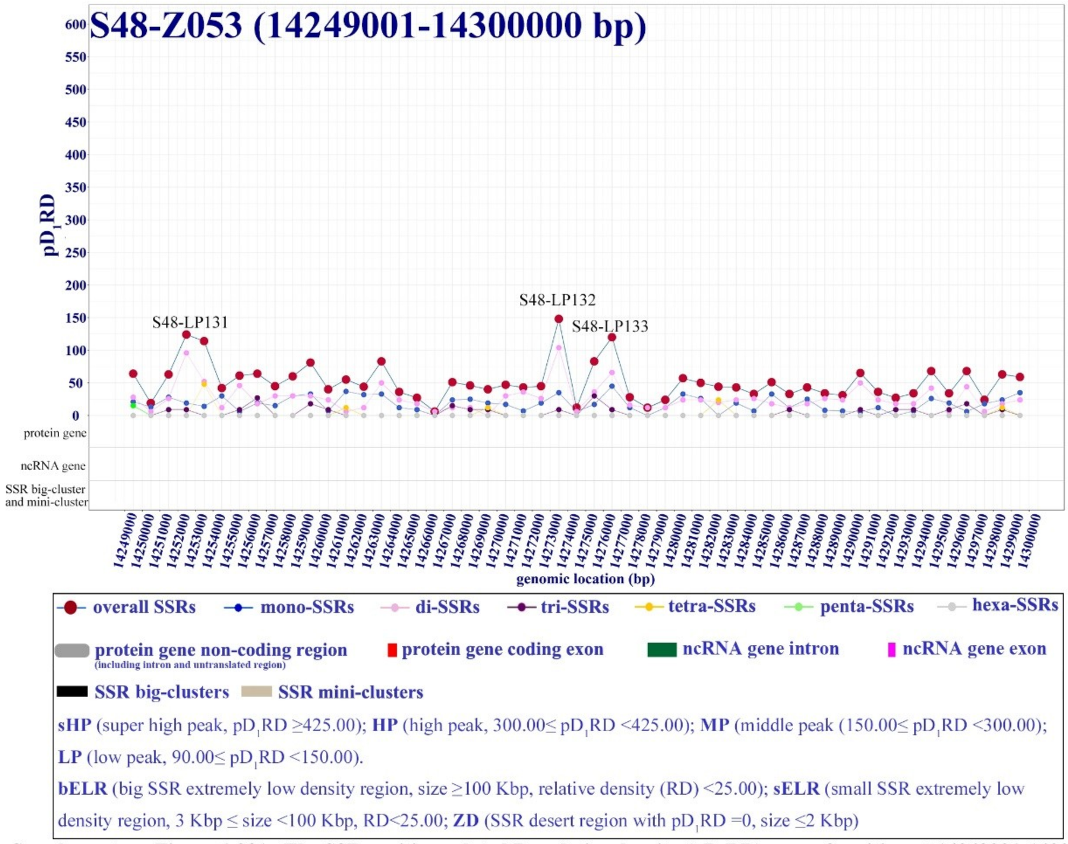

Supplementary Figure 1.281. The SSR position related D_1-relative density (pD_1RD) map of position at 14249001-14300000 bp of human reference Y-DNA (NC_000024.10) at resolution of 1 Kbp.

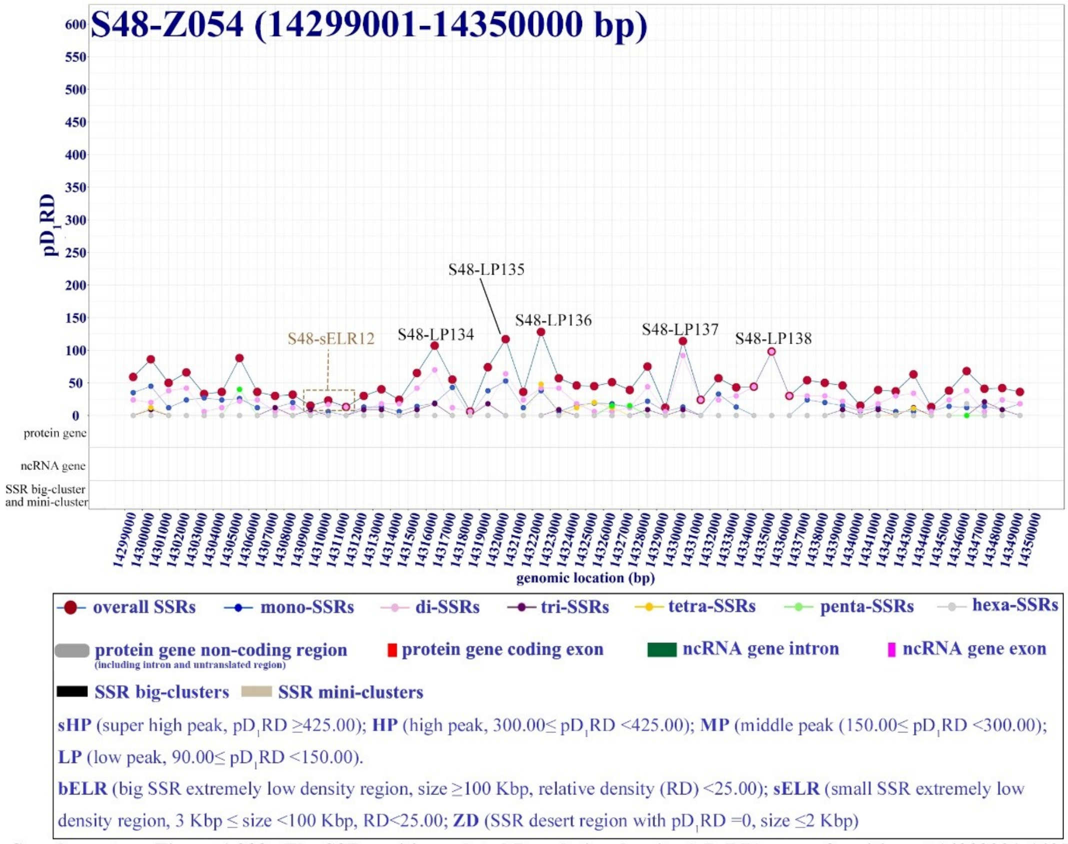

Supplementary Figure 1.282. The SSR position related D_1-relative density (pD_1RD) map of position at 14299001-14350000 bp of human reference Y-DNA (NC_000024.10) at resolution of 1 Kbp.

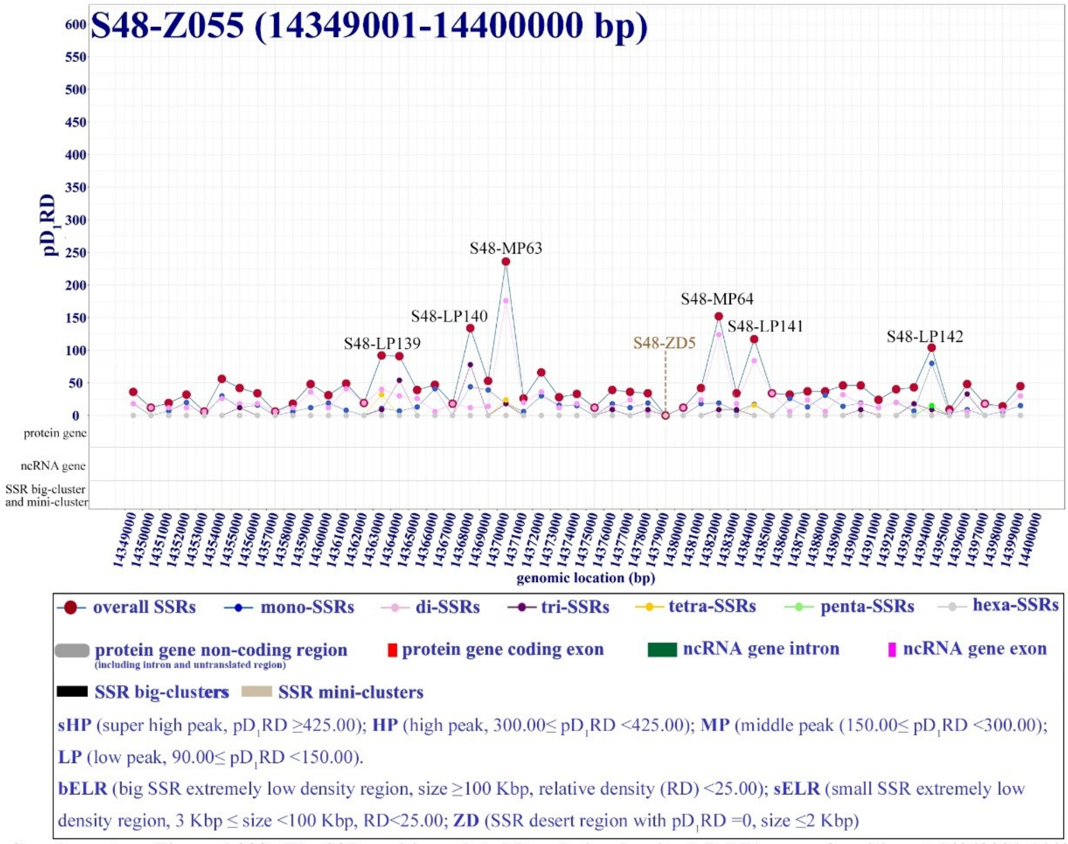

Supplementary Figure 1.283. The SSR position related D_1-relative density (pD_1RD) map of position at 14349001-14400000 bp of human reference Y-DNA (NC_000024.10) at resolution of 1 Kbp.

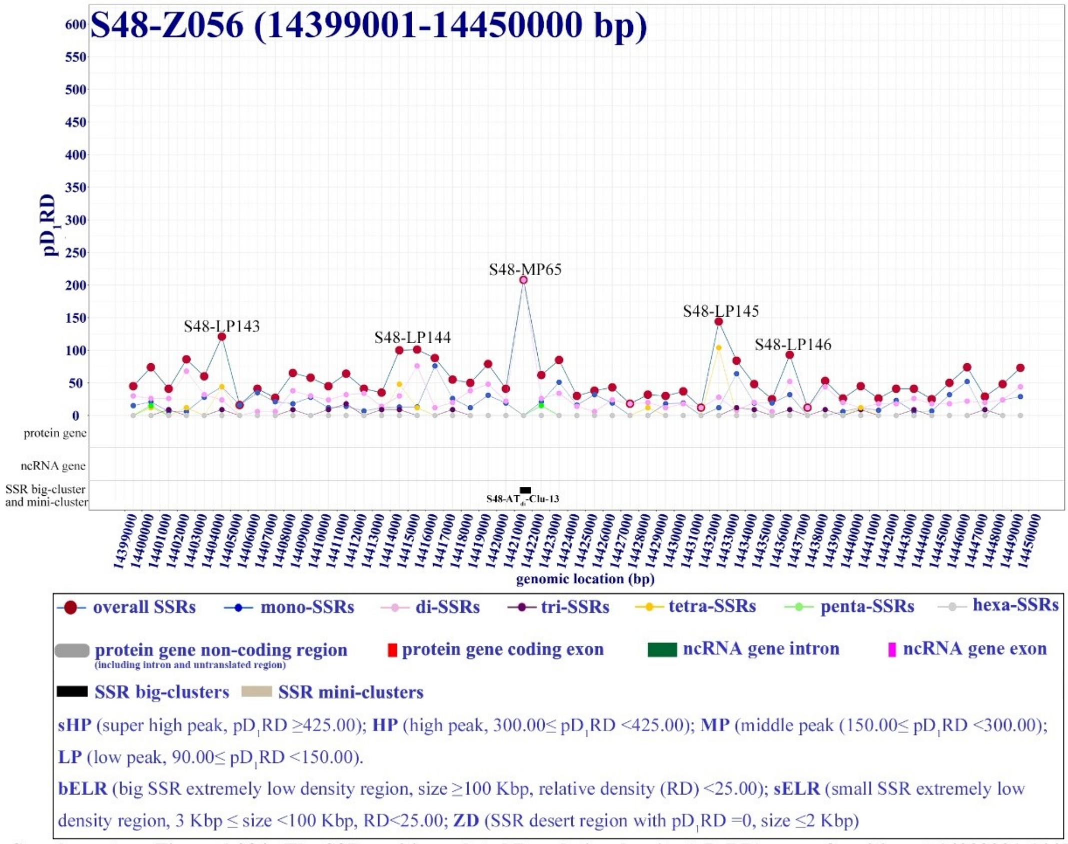

Supplementary Figure 1.284. The SSR position related D_1-relative density (pD_1RD) map of position at 14399001-14450000 bp of human reference Y-DNA (NC_000024.10) at resolution of 1 Kbp.

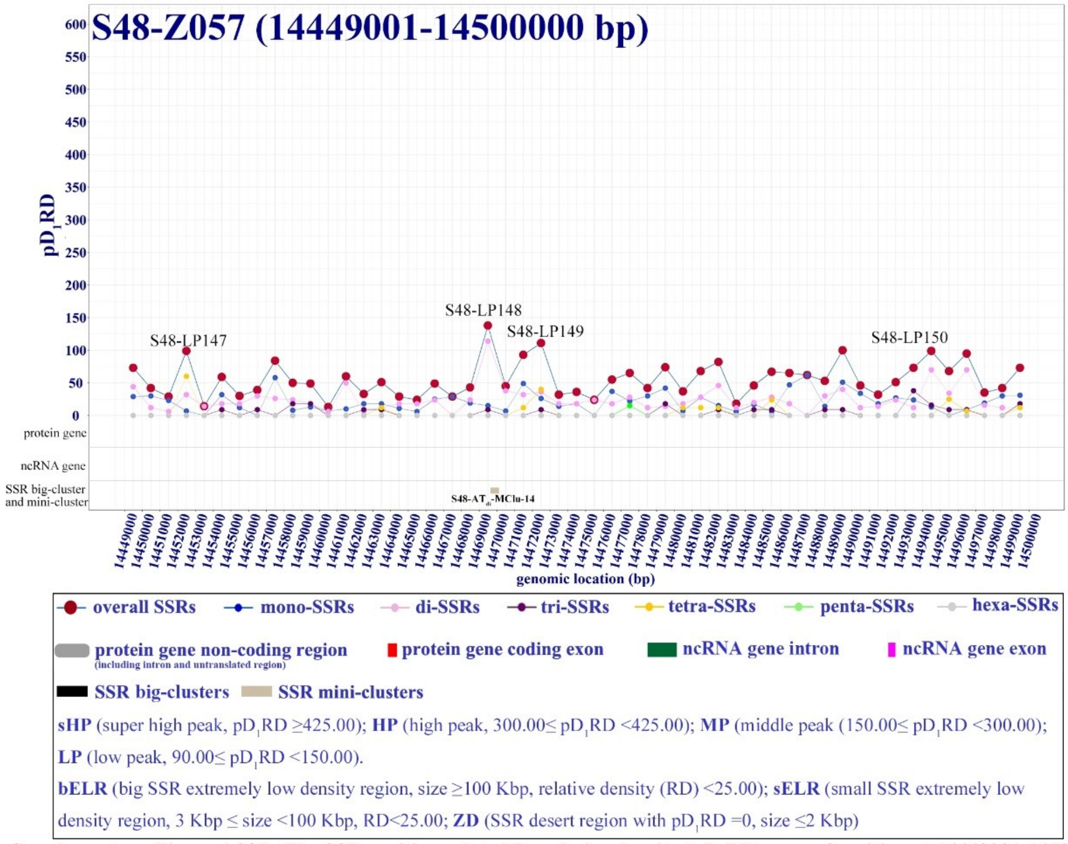

Supplementary Figure 1.285. The SSR position related D_1-relative density (pD_1RD) map of position at 14449001-14500000 bp of human reference Y-DNA (NC_000024.10) at resolution of 1 Kbp.

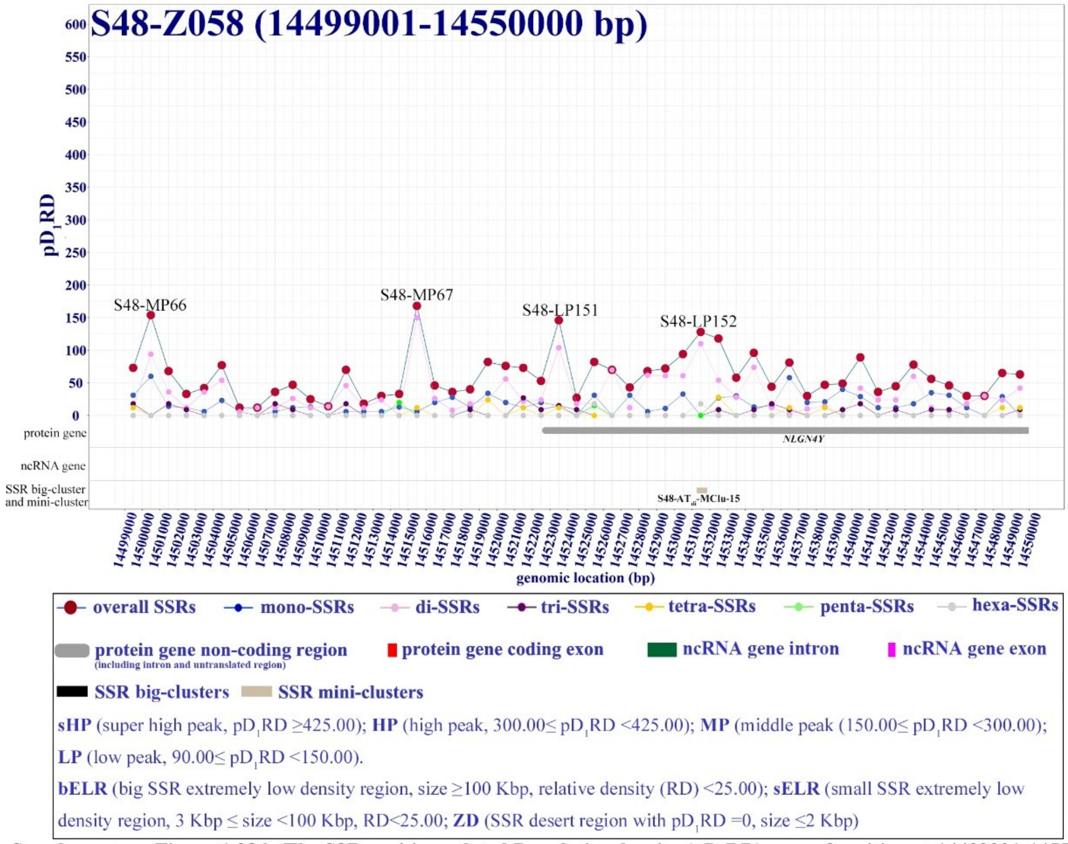

Supplementary Figure 1.286. The SSR position related D_1-relative density (pD_1RD) map of position at 14499001-14550000 bp of human reference Y-DNA (NC_000024.10) at resolution of 1 Kbp.

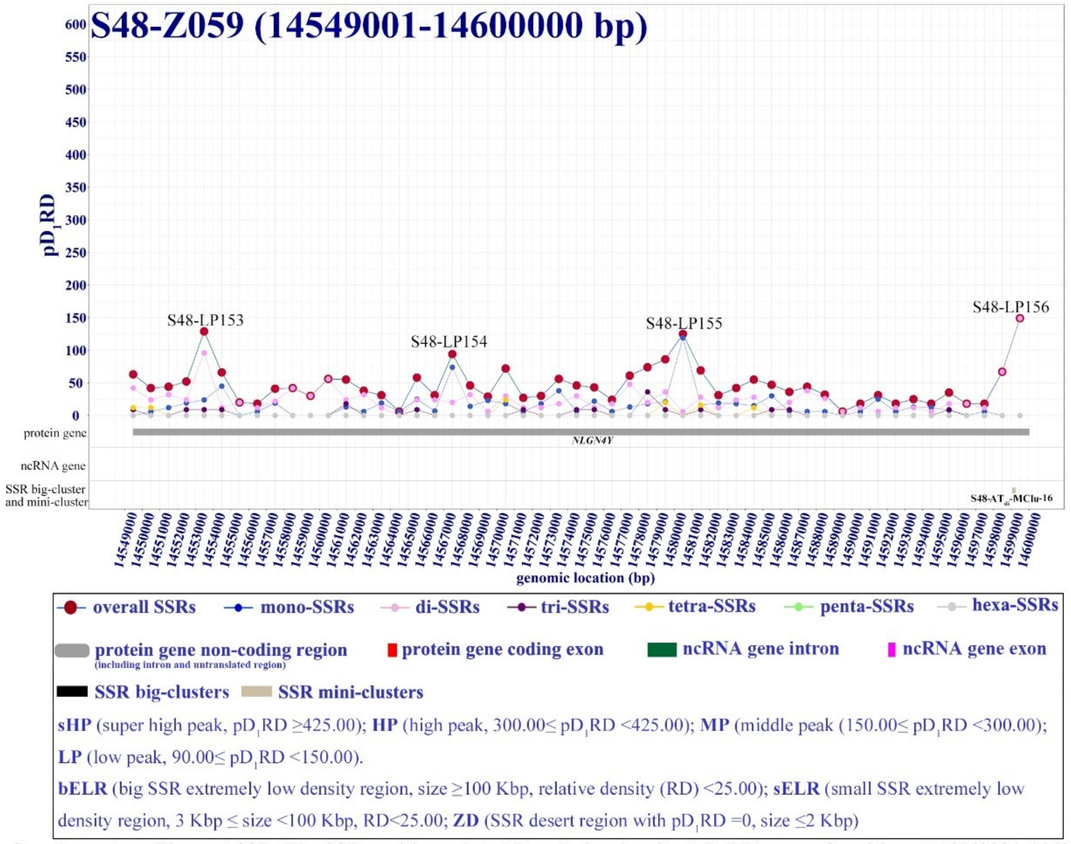

Supplementary Figure 1.287. The SSR position related D_1-relative density (pD_1RD) map of position at 14549001-14600000 bp of human reference Y-DNA (NC_000024.10) at resolution of 1 Kbp.

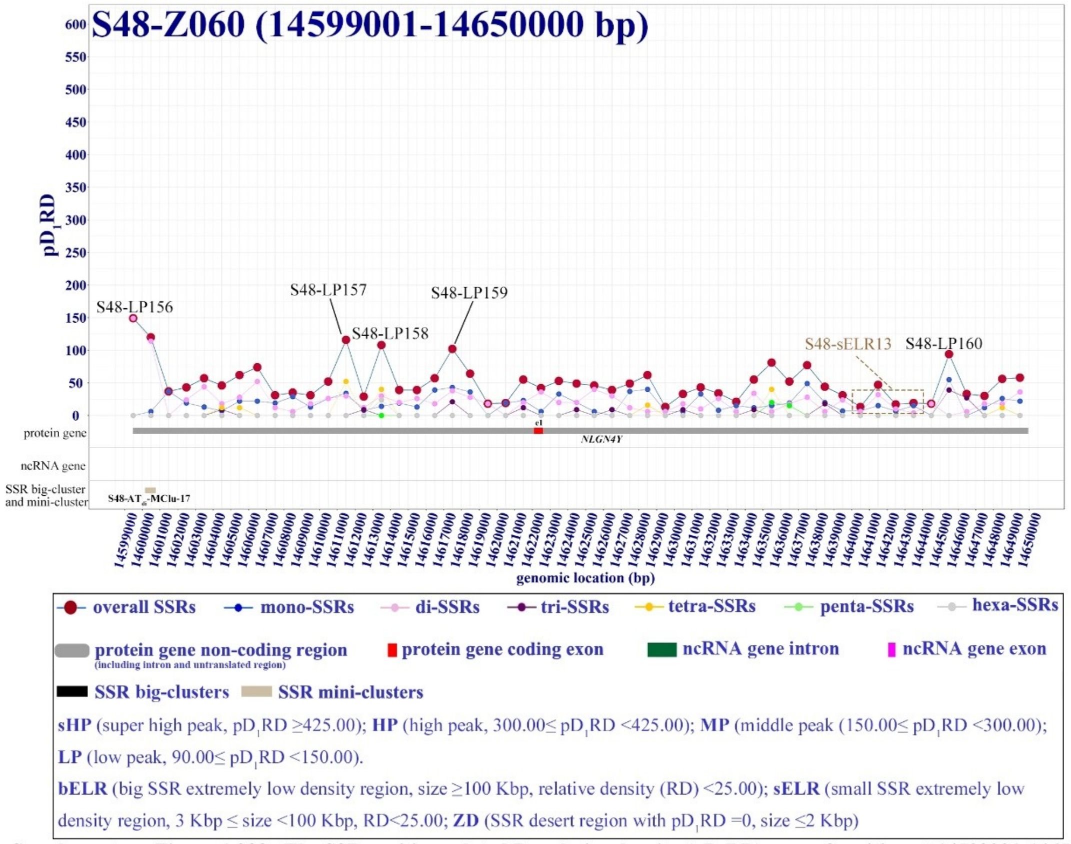

Supplementary Figure 1.288. The SSR position related D_1-relative density (pD_1RD) map of position at 14599001-14650000 bp of human reference Y-DNA (NC_000024.10) at resolution of 1 Kbp.

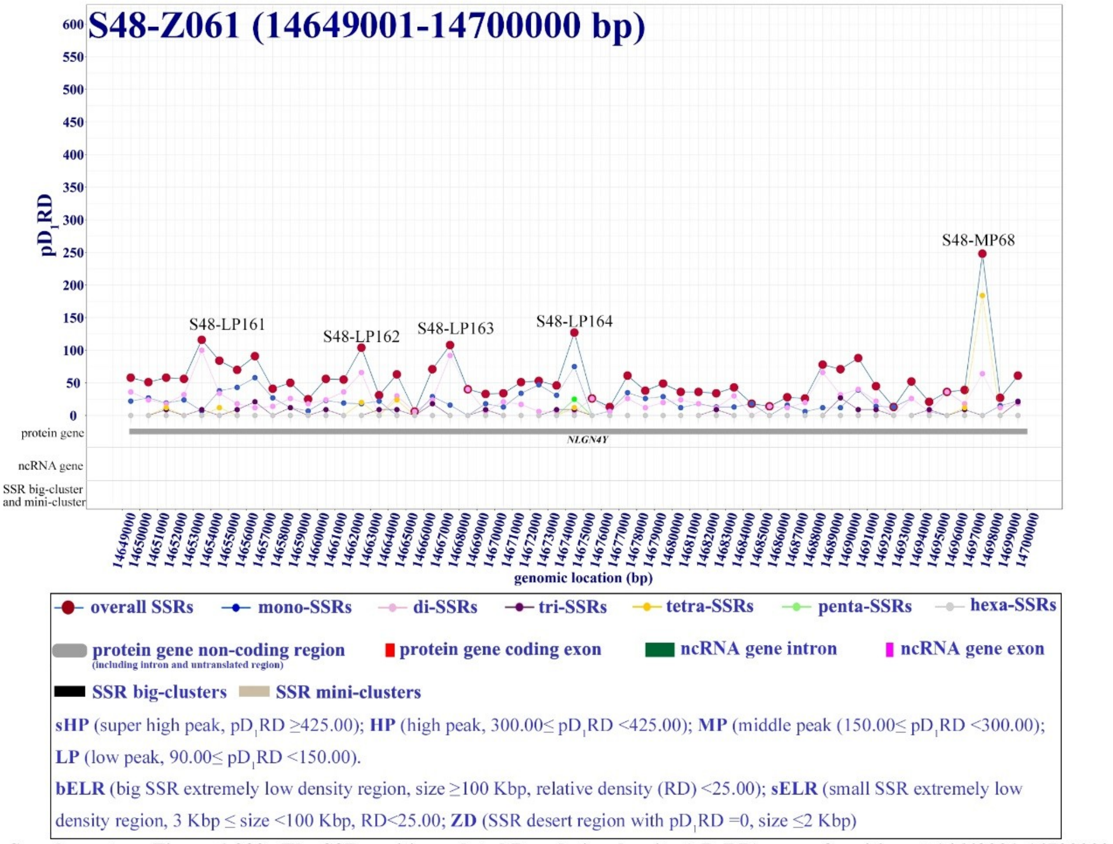

Supplementary Figure 1.289. The SSR position related D_1-relative density (pD_1RD) map of position at 14649001-14700000 bp of human reference Y-DNA (NC_000024.10) at resolution of 1 Kbp.

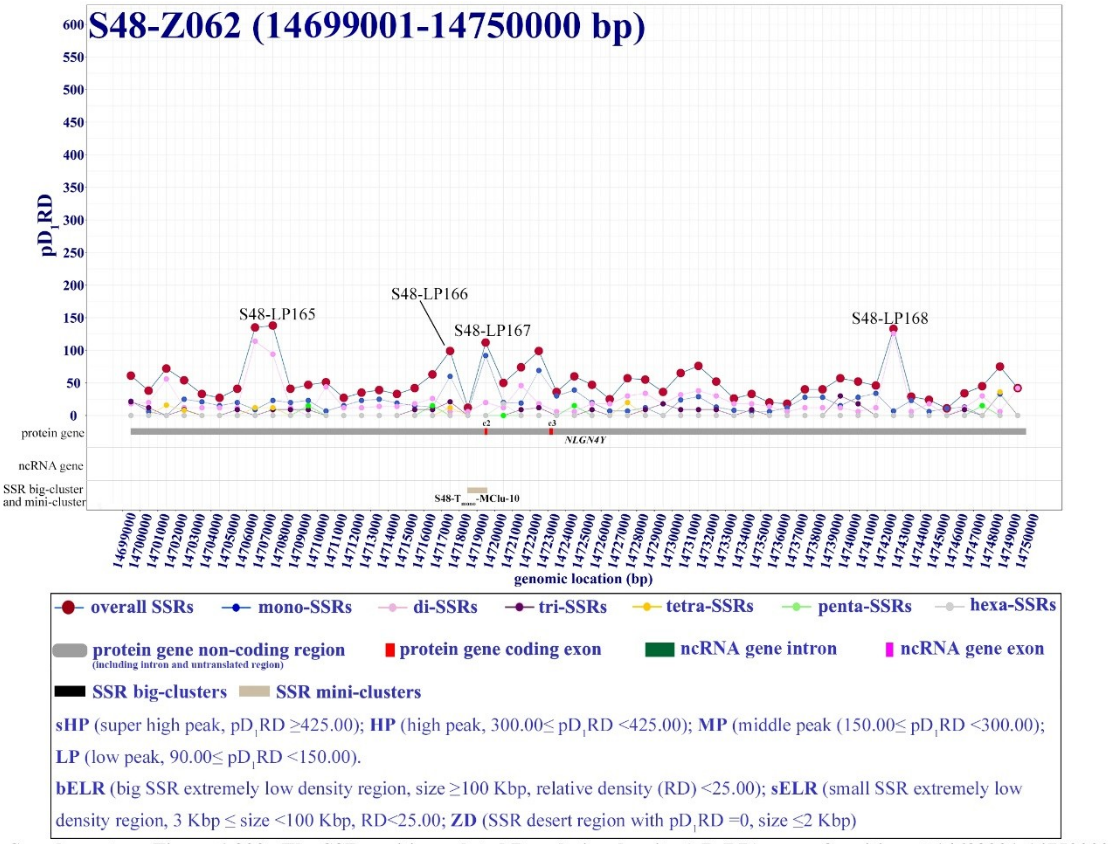

Supplementary Figure 1.290. The SSR position related D_1-relative density (pD_1RD) map of position at 14699001-14750000 bp of human reference Y-DNA (NC_000024.10) at resolution of 1 Kbp.

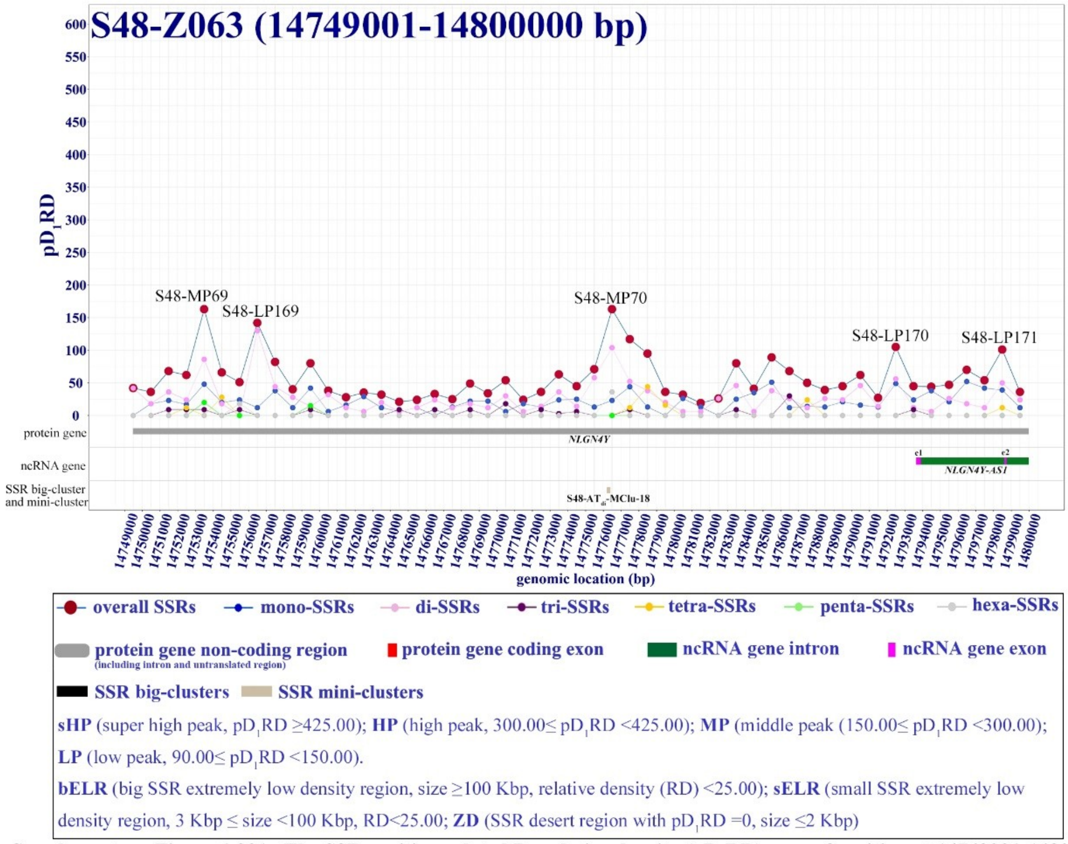

Supplementary Figure 1.291. The SSR position related D_1-relative density (pD_1RD) map of position at 14749001-14800000 bp of human reference Y-DNA (NC_000024.10) at resolution of 1 Kbp.

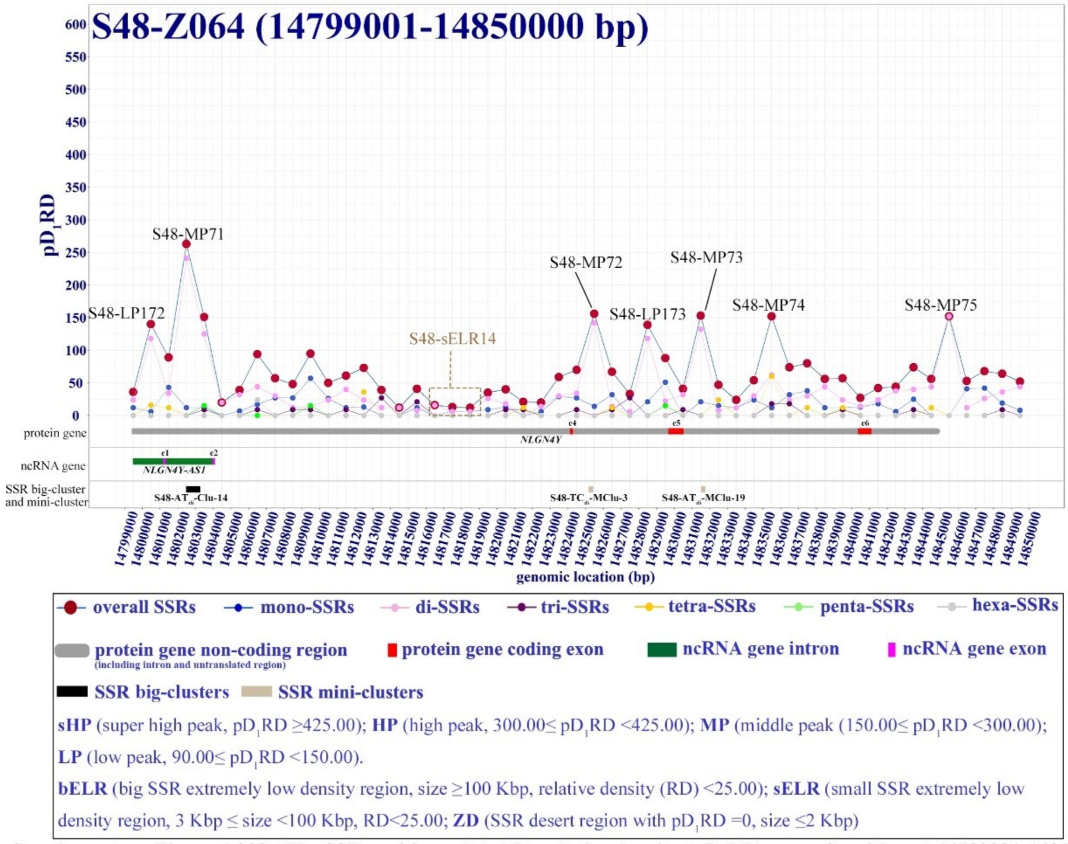

Supplementary Figure 1.292. The SSR position related D_1-relative density (pD_1RD) map of position at 14799001-14850000 bp of human reference Y-DNA (NC_000024.10) at resolution of 1 Kbp.

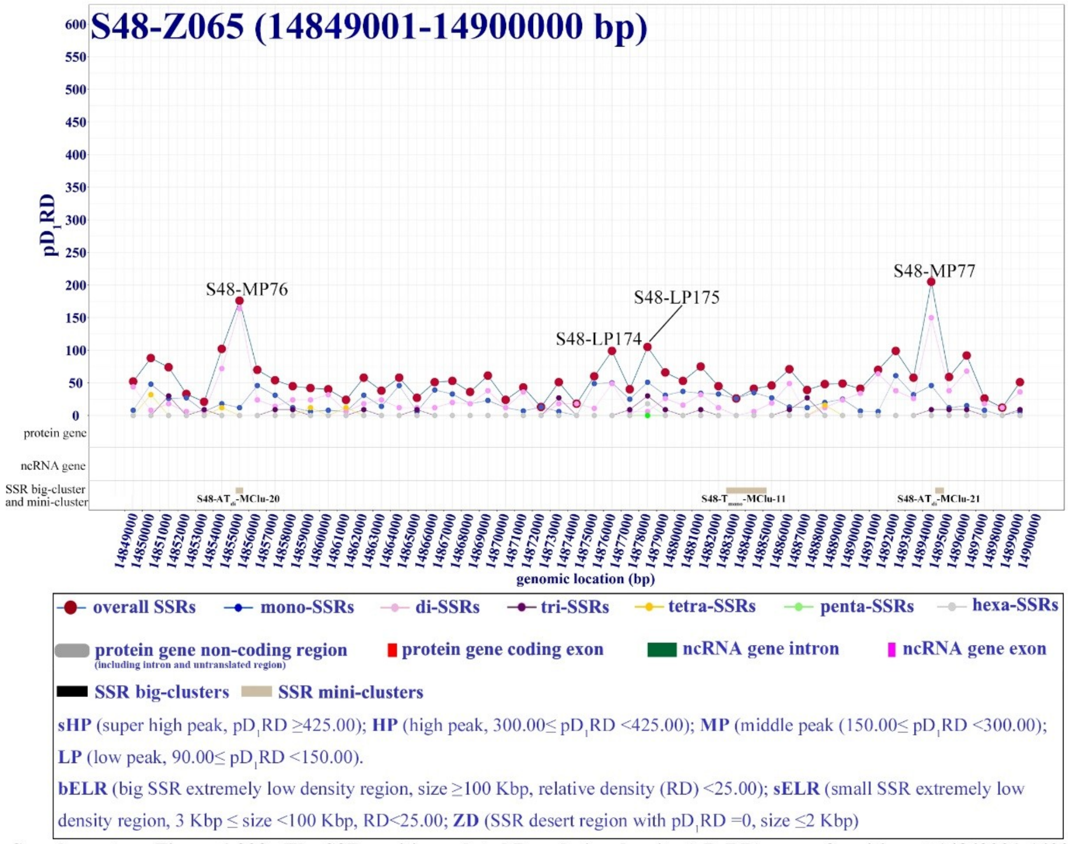

Supplementary Figure 1.293. The SSR position related D_1-relative density (pD_1RD) map of position at 14849001-14900000 bp of human reference Y-DNA (NC_000024.10) at resolution of 1 Kbp.

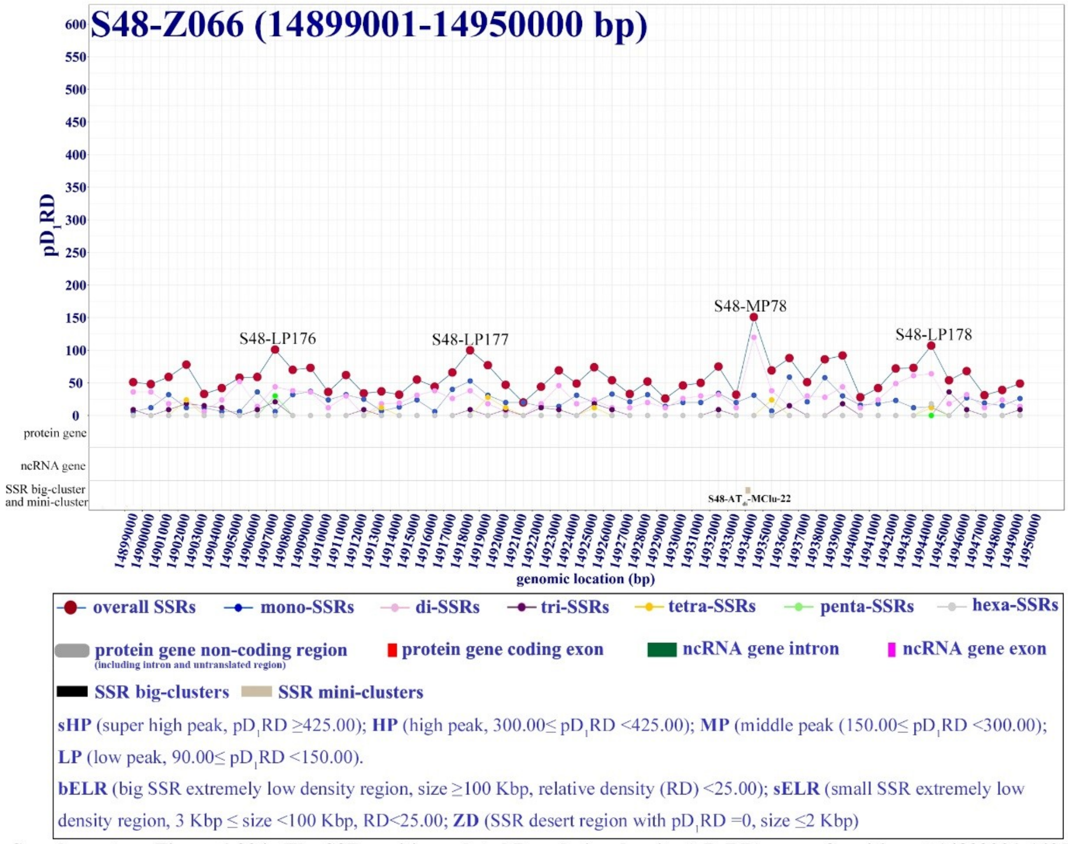

Supplementary Figure 1.294. The SSR position related D_1-relative density (pD_1RD) map of position at 14899001-14950000 bp of human reference Y-DNA (NC_000024.10) at resolution of 1 Kbp.

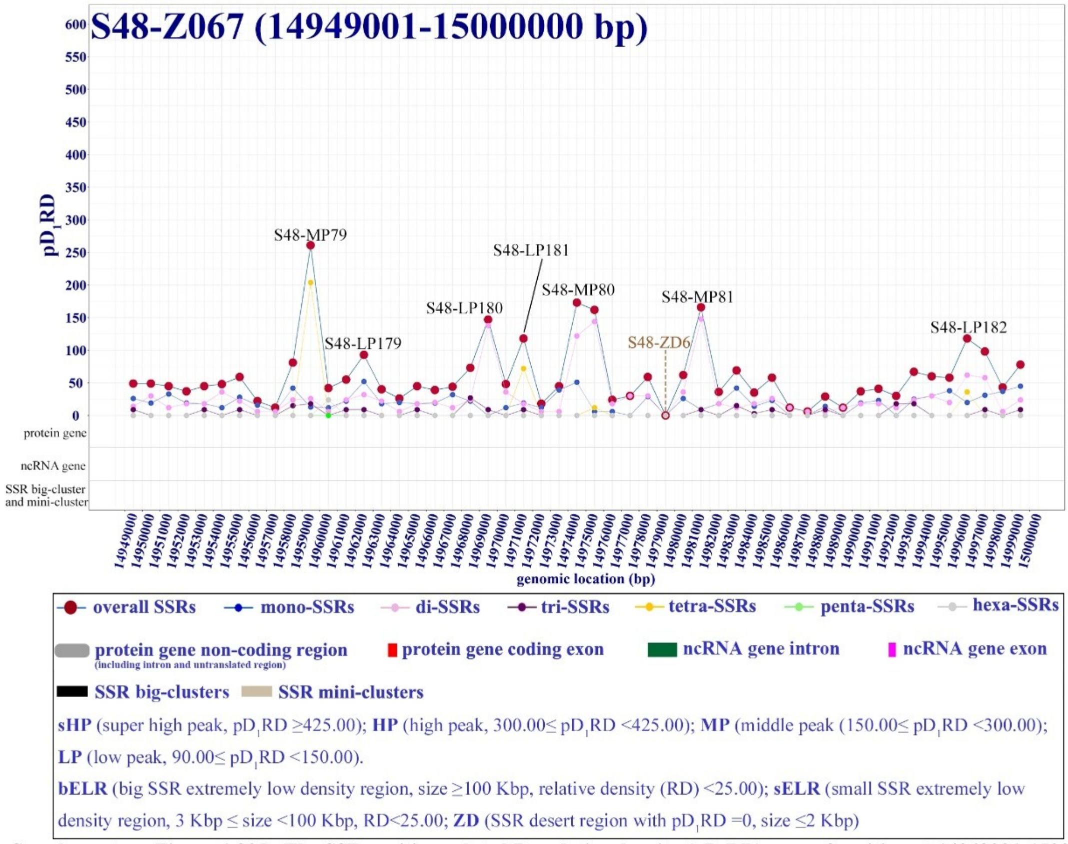

Supplementary Figure 1.295. The SSR position related D_1-relative density (pD_1RD) map of position at 14949001-15000000 bp of human reference Y-DNA (NC_000024.10) at resolution of 1 Kbp.

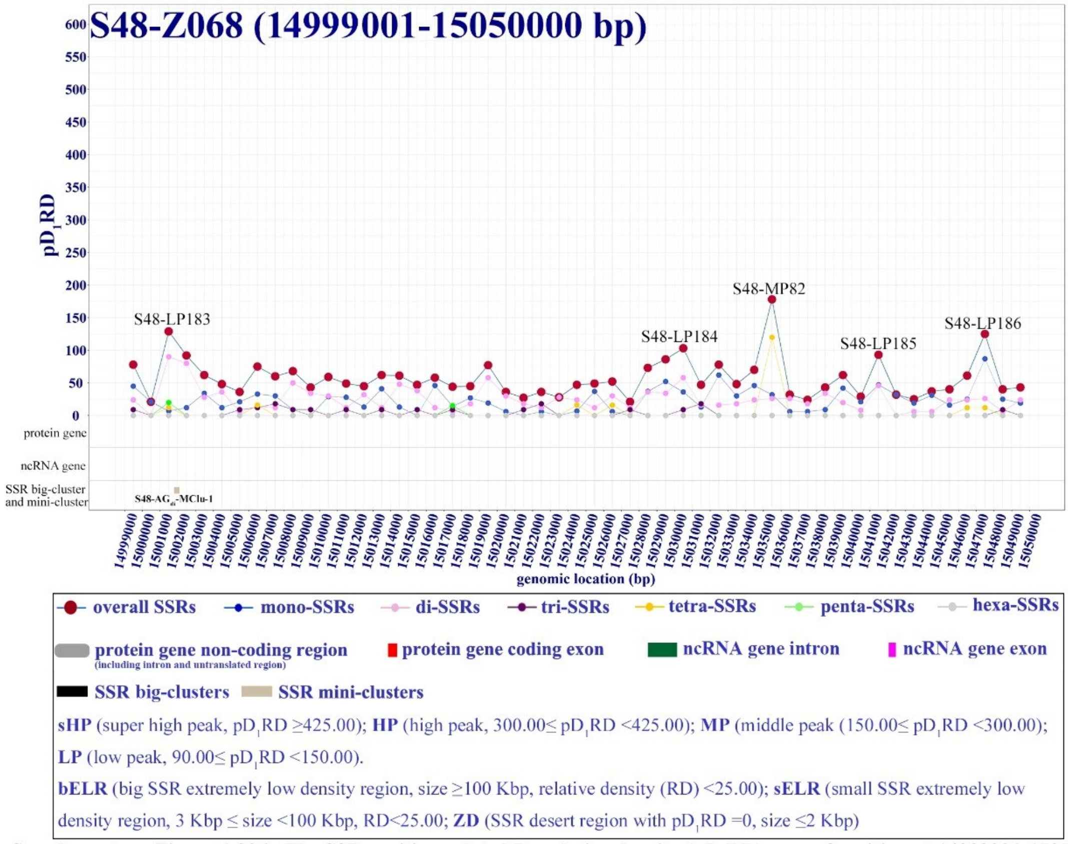

Supplementary Figure 1.296. The SSR position related D_1-relative density (pD_1RD) map of position at 14999001-15050000 bp of human reference Y-DNA (NC_000024.10) at resolution of 1 Kbp.

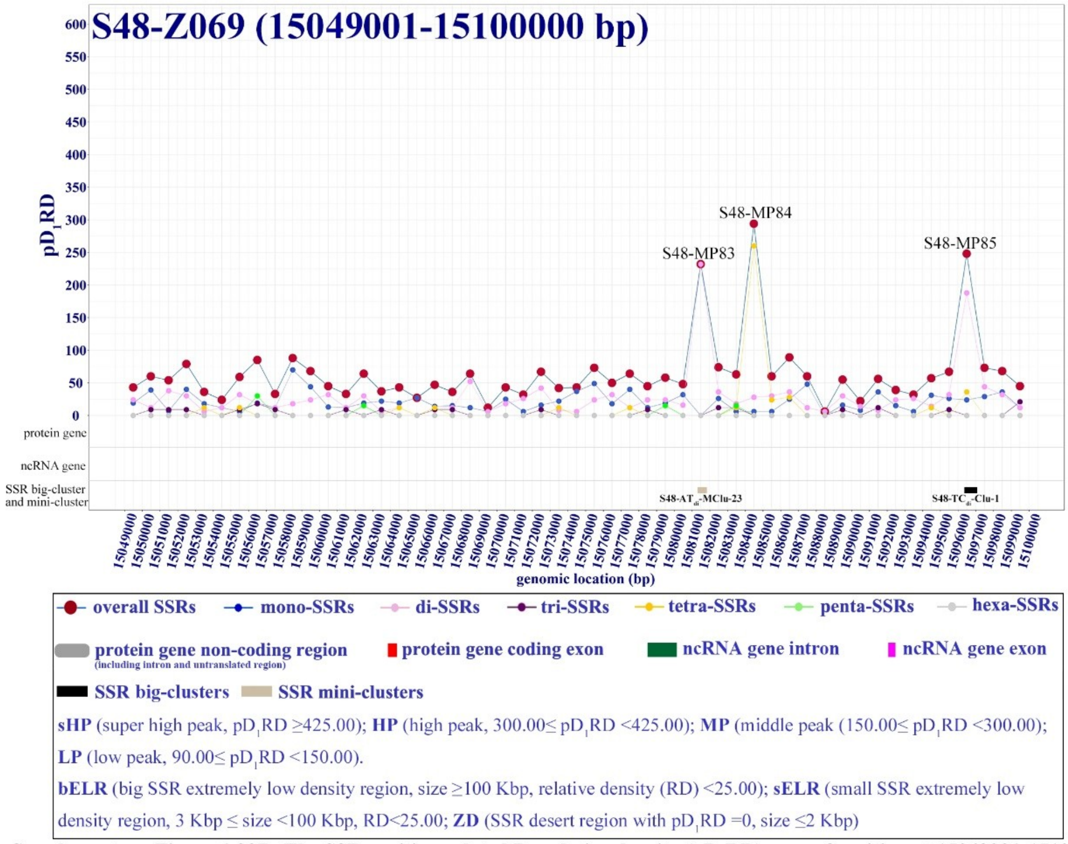

Supplementary Figure 1.297. The SSR position related D_1-relative density (pD_1RD) map of position at 15049001-15100000 bp of human reference Y-DNA (NC_000024.10) at resolution of 1 Kbp.

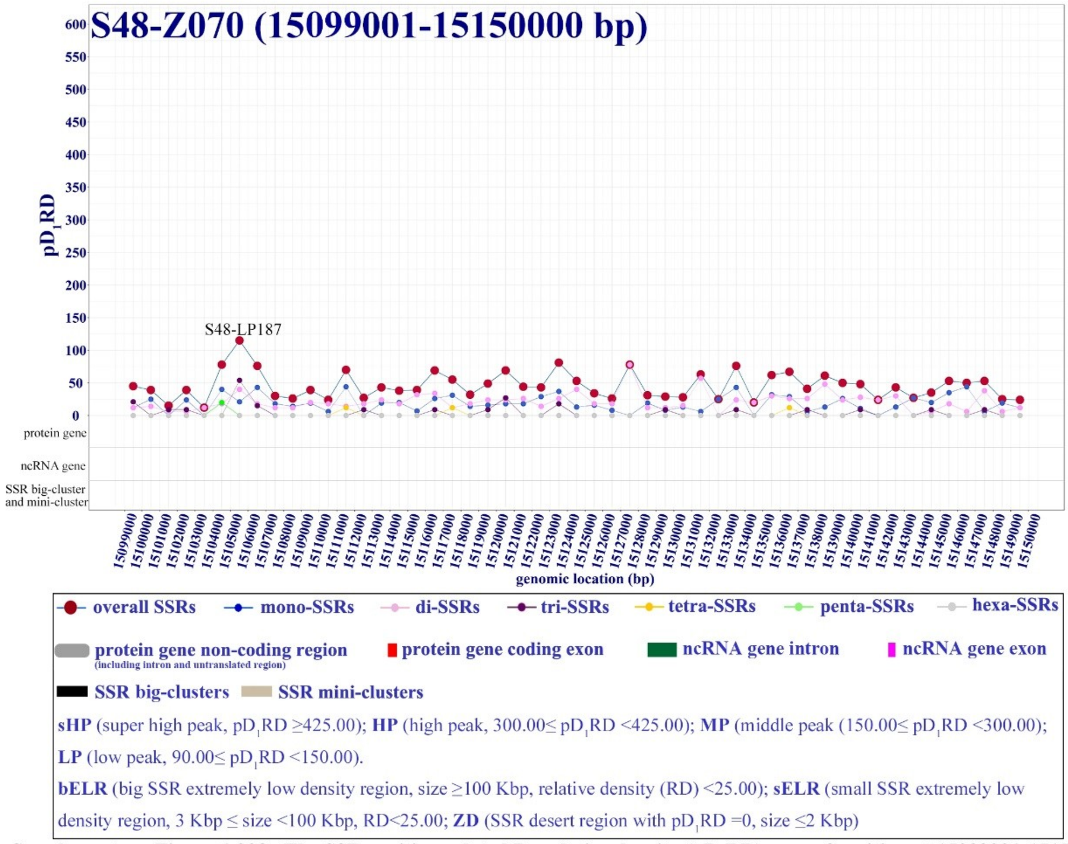

Supplementary Figure 1.298. The SSR position related D_1-relative density (pD_1RD) map of position at 15099001-15150000 bp of human reference Y-DNA (NC_000024.10) at resolution of 1 Kbp.

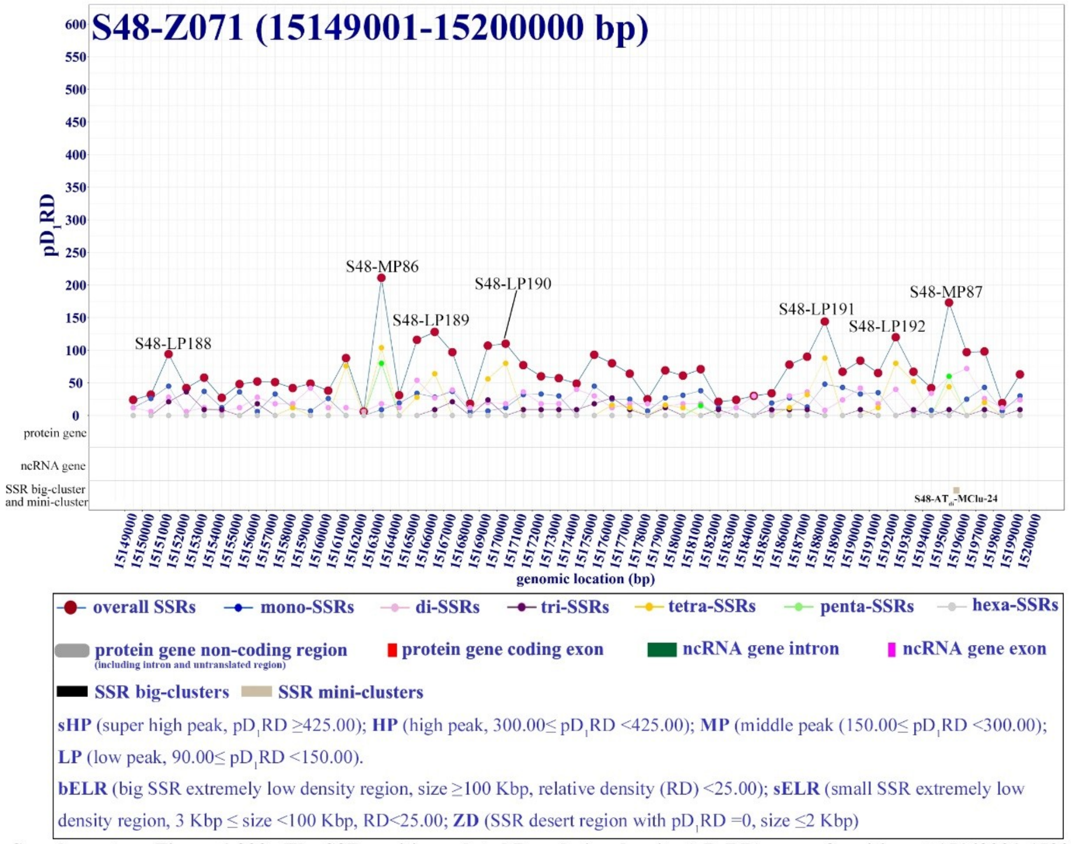

Supplementary Figure 1.299. The SSR position related D_1-relative density (pD_1RD) map of position at 15149001-15200000 bp of human reference Y-DNA (NC_000024.10) at resolution of 1 Kbp.

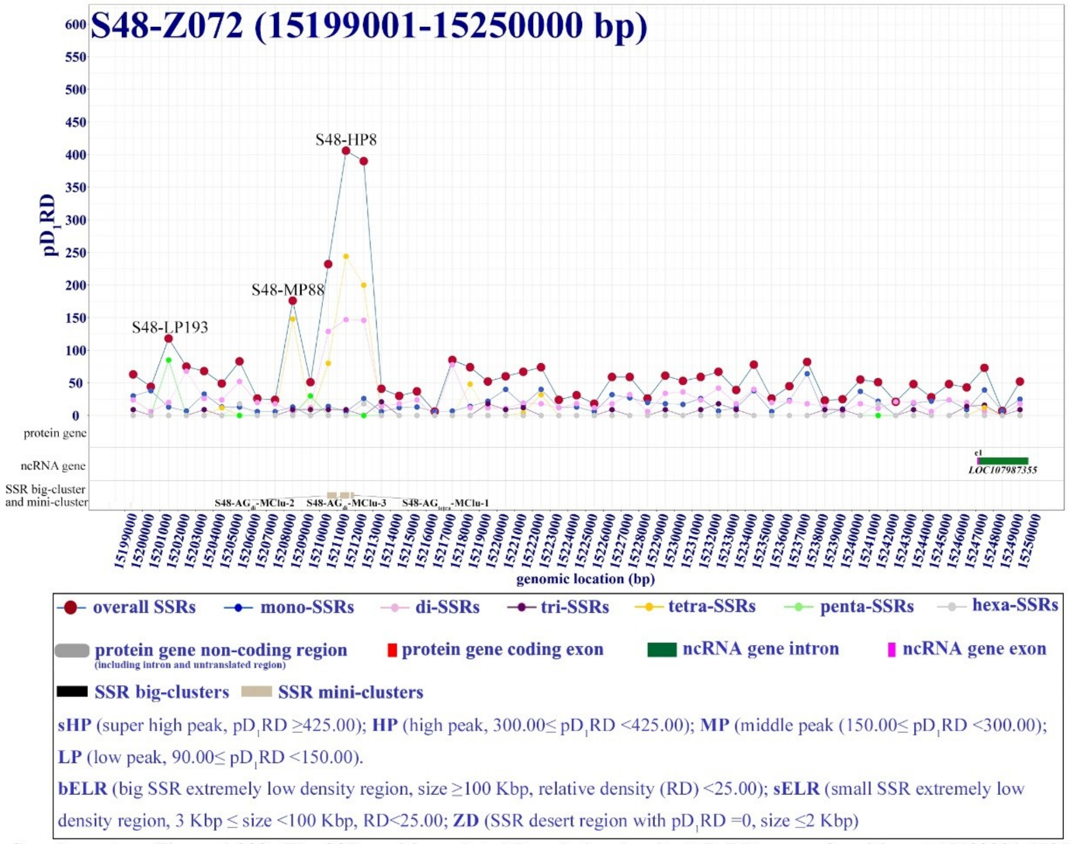

Supplementary Figure 1.300. The SSR position related D_1-relative density (pD_1RD) map of position at 15199001-15250000 bp of human reference Y-DNA (NC_000024.10) at resolution of 1 Kbp.

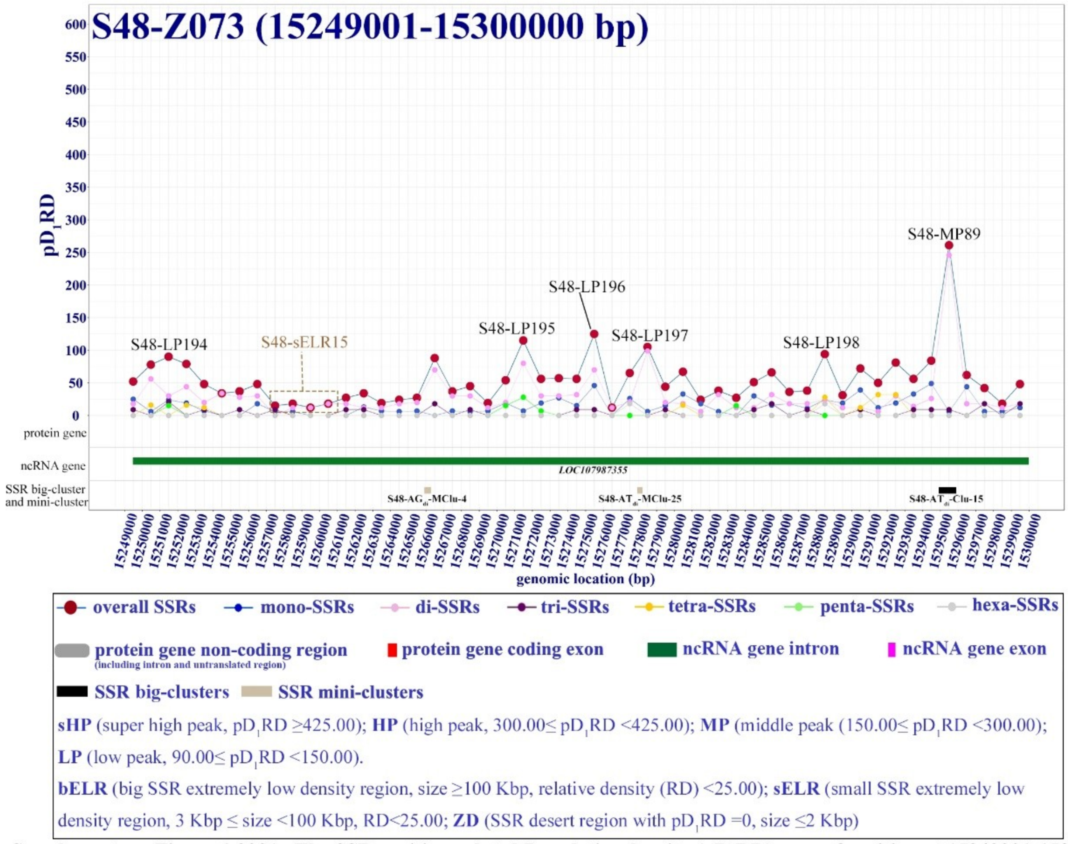

Supplementary Figure 1.3001. The SSR position related D_1-relative density (pD_1RD) map of position at 15249001-1530000 bp of human reference Y-DNA (NC_000024.10) at resolution of 1 Kbp.

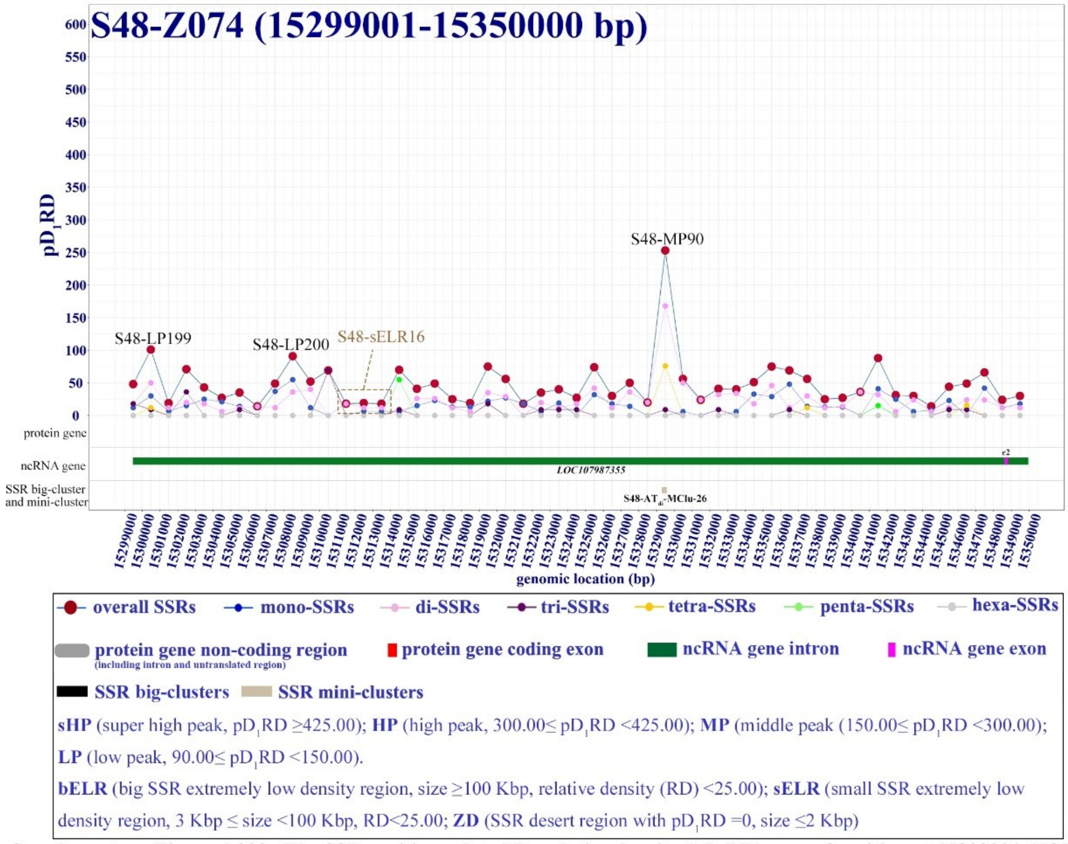

Supplementary Figure 1.302. The SSR position related D_1-relative density (pD_1RD) map of position at 15299001-15350000 bp of human reference Y-DNA (NC_000024.10) at resolution of 1 Kbp.

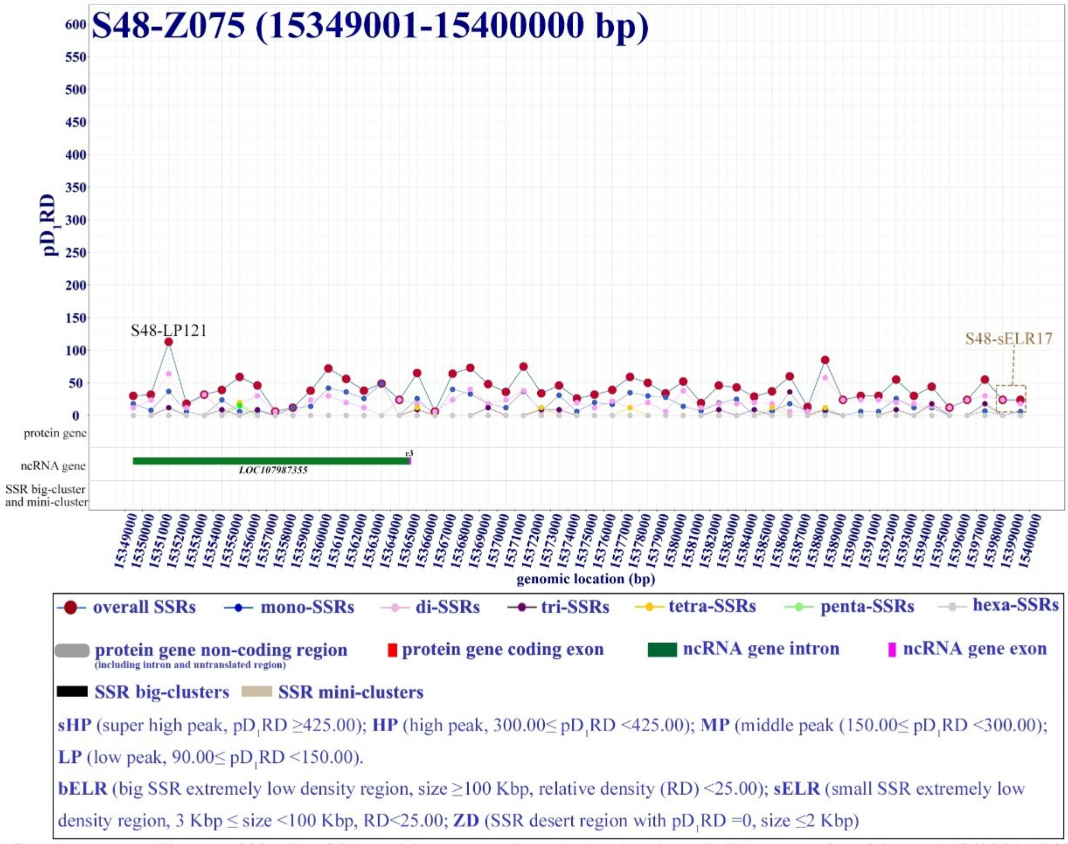

Supplementary Figure 1.303. The SSR position related D_1-relative density (pD_1RD) map of position at 15349001-15400000 bp of human reference Y-DNA (NC_000024.10) at resolution of 1 Kbp.

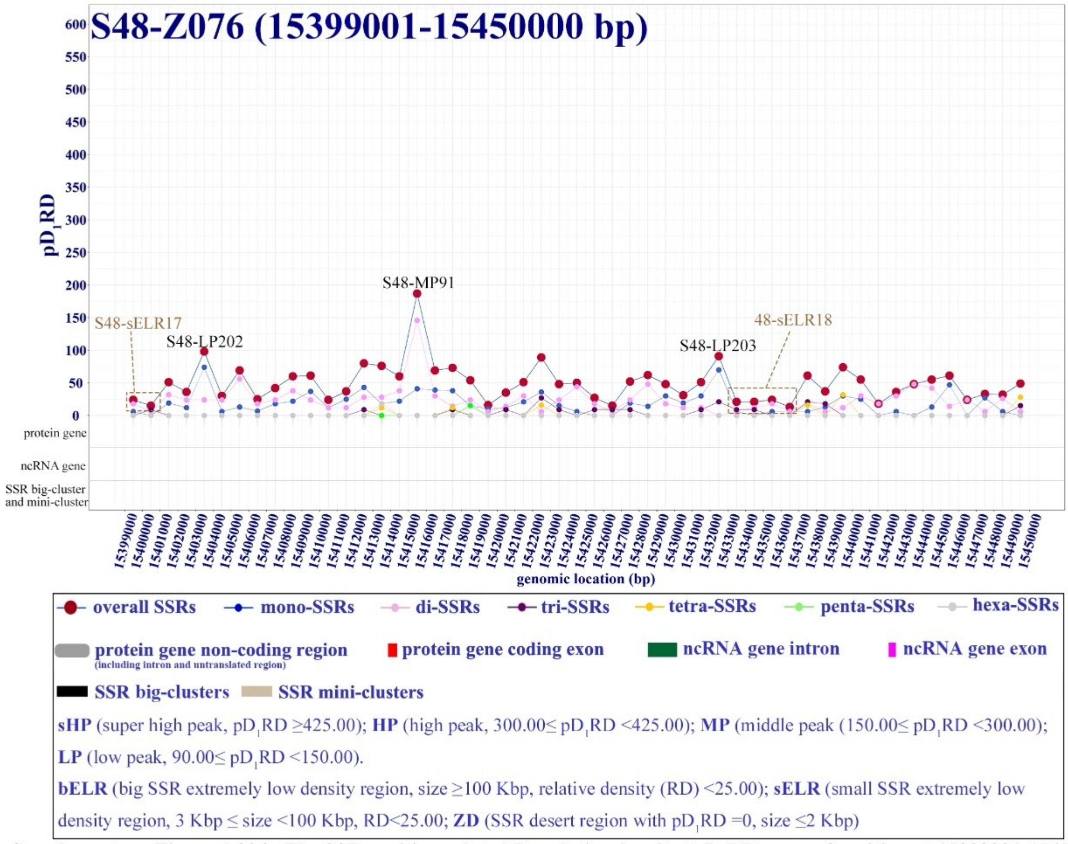

Supplementary Figure 1.304. The SSR position related D_1-relative density (pD_1RD) map of position at 15399001-15450000 bp of human reference Y-DNA (NC_000024.10) at resolution of 1 Kbp.

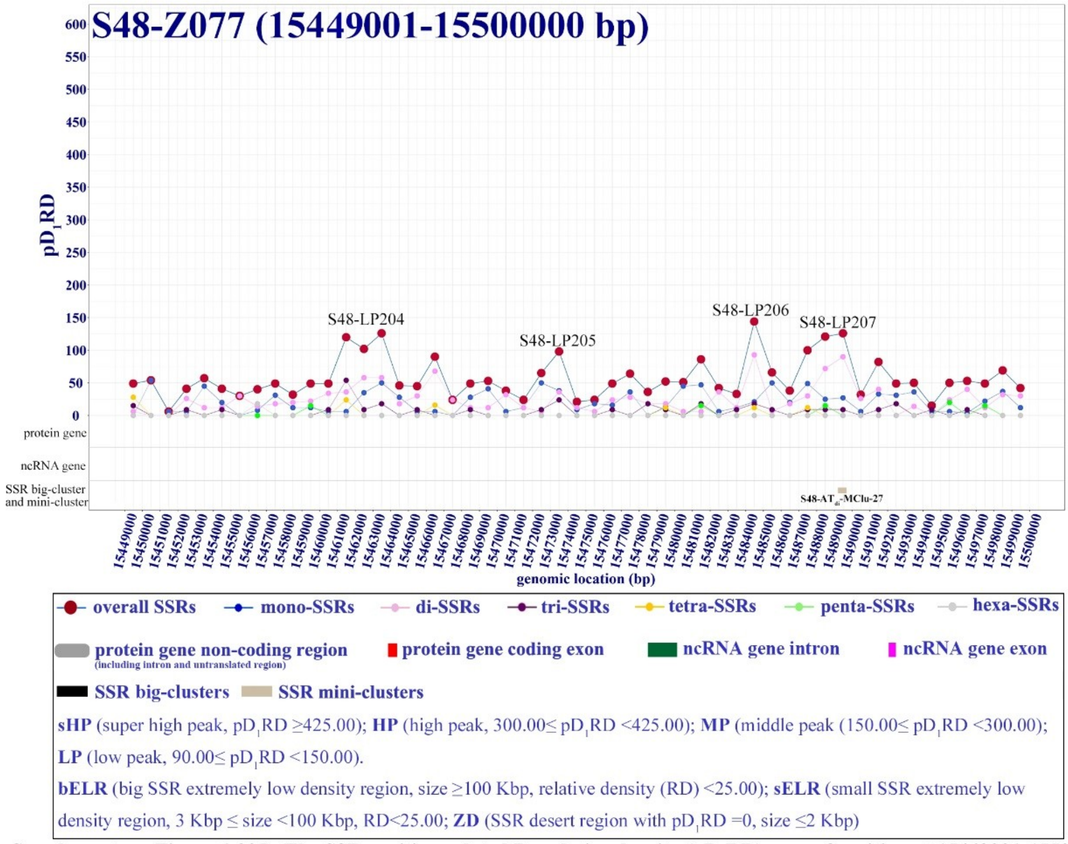

Supplementary Figure 1.305. The SSR position related D_1-relative density (pD_1RD) map of position at 15449001-15500000 bp of human reference Y-DNA (NC_000024.10) at resolution of 1 Kbp.

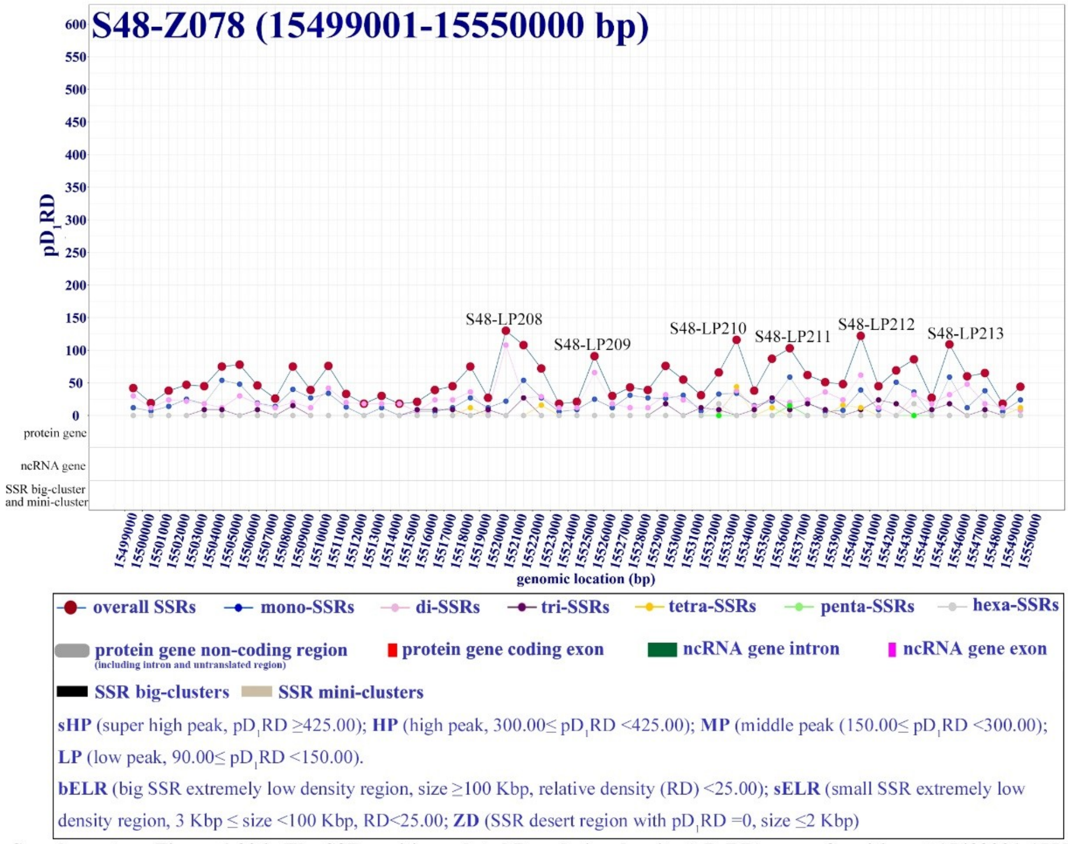

Supplementary Figure 1.306. The SSR position related D_1-relative density (pD_1RD) map of position at 15499001-15550000 bp of human reference Y-DNA (NC_000024.10) at resolution of 1 Kbp.

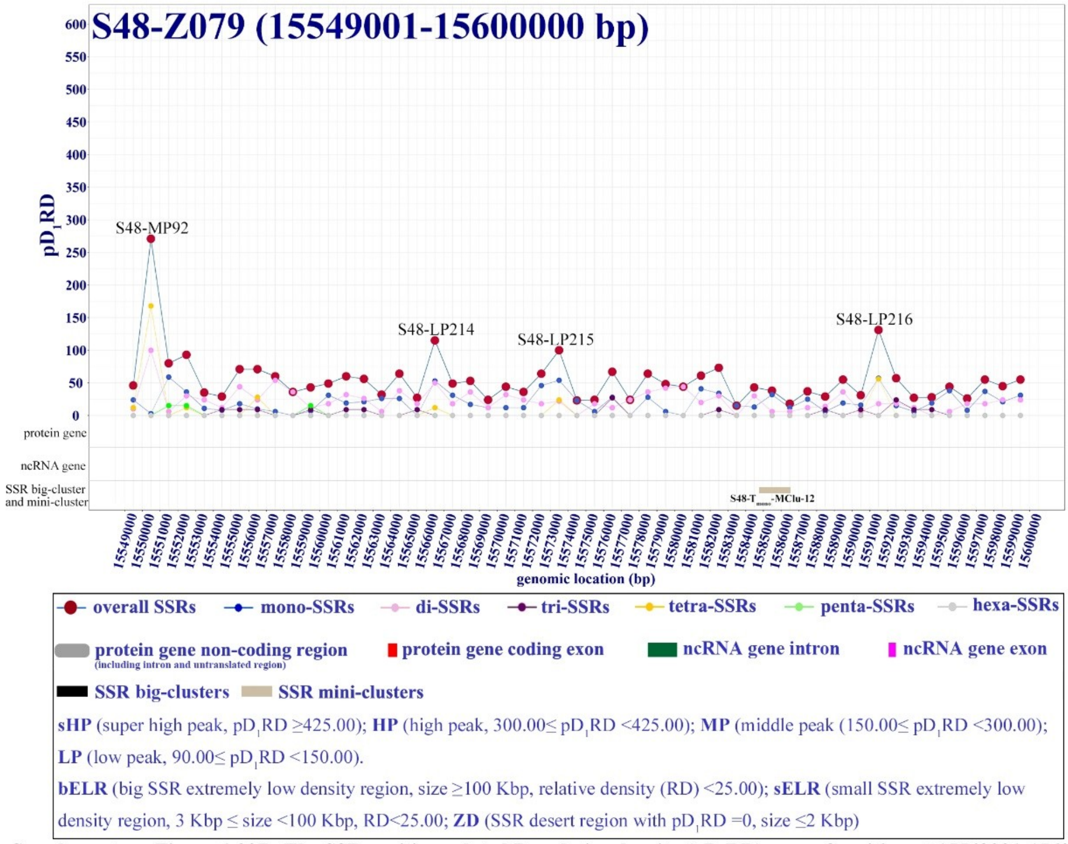

Supplementary Figure 1.307. The SSR position related D_1-relative density (pD_1RD) map of position at 15549001-15600000 bp of human reference Y-DNA (NC_000024.10) at resolution of 1 Kbp.

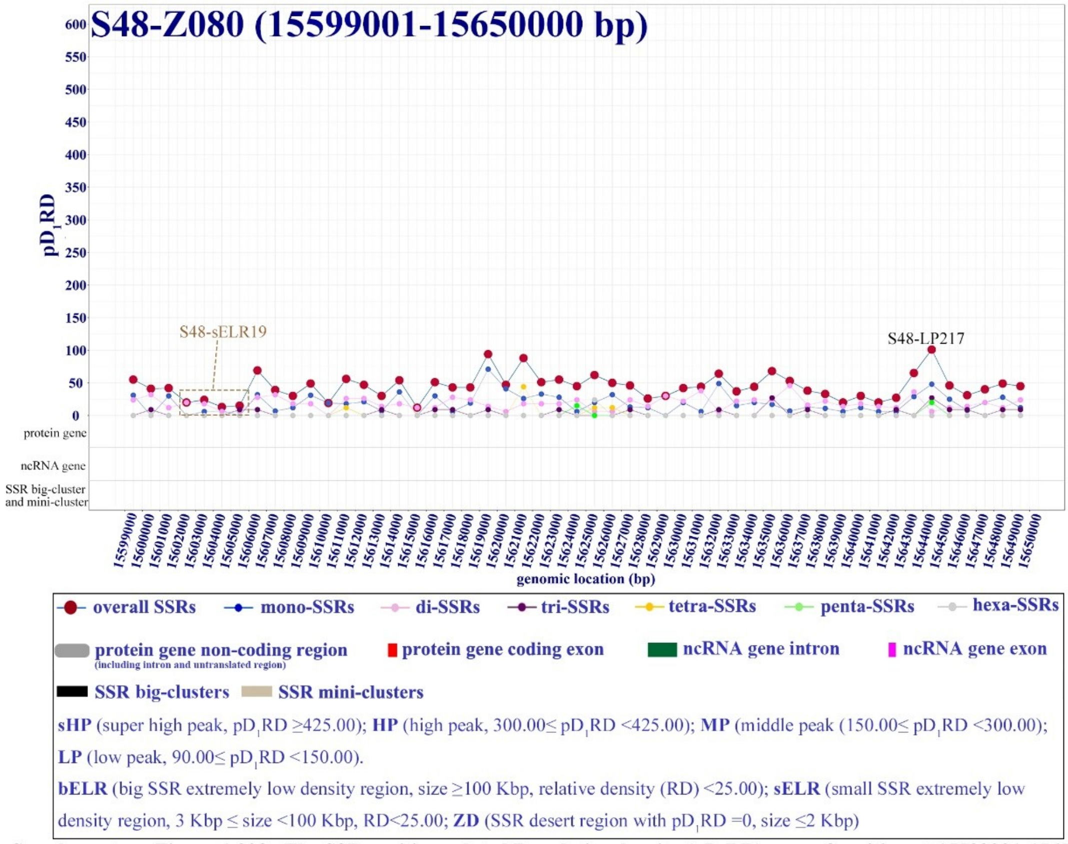

Supplementary Figure 1.308. The SSR position related D_1-relative density (pD_1RD) map of position at 15599001-15650000 bp of human reference Y-DNA (NC_000024.10) at resolution of 1 Kbp.

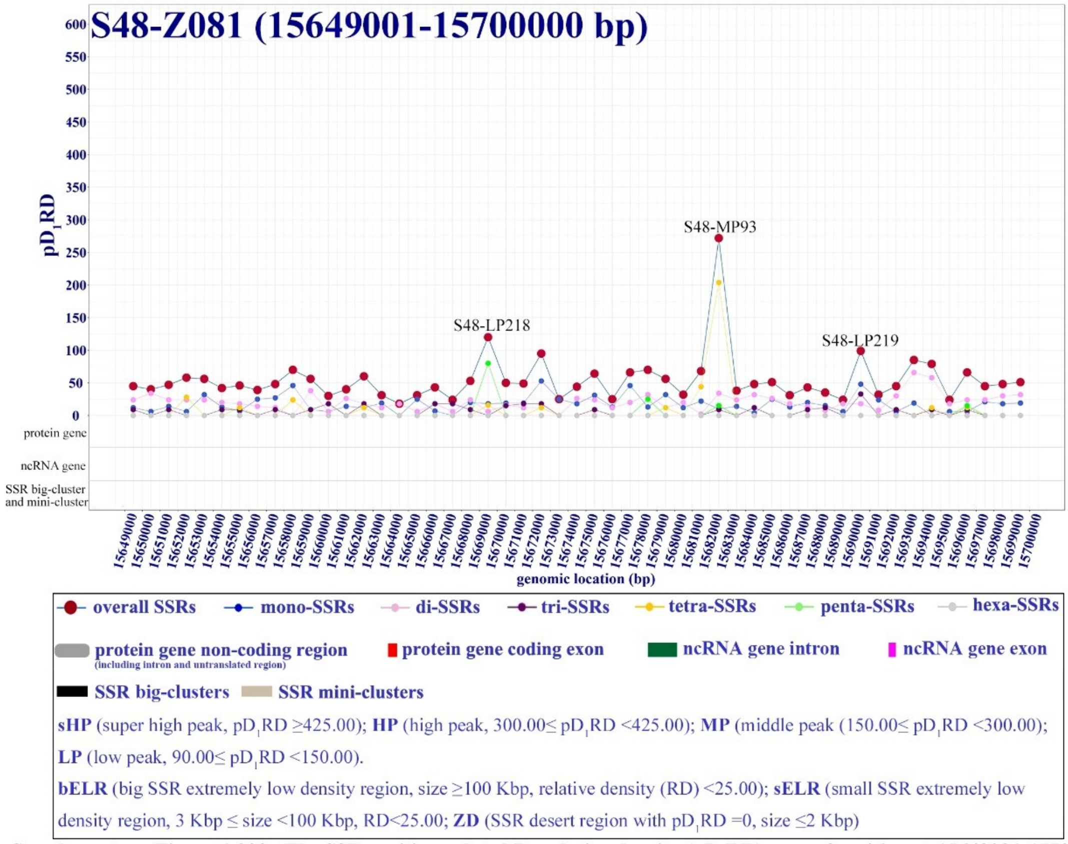

Supplementary Figure 1.309. The SSR position related D_1-relative density (pD_1RD) map of position at 15649001-15700000 bp of human reference Y-DNA (NC_000024.10) at resolution of 1 Kbp.

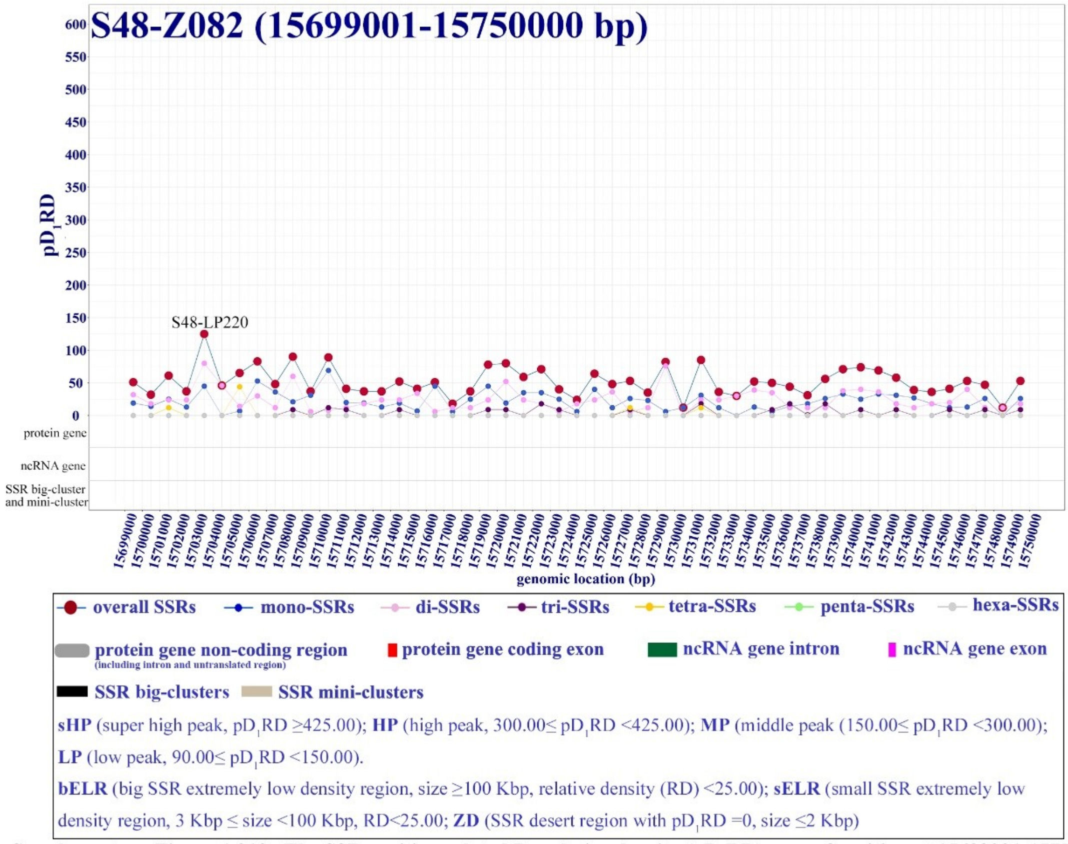

Supplementary Figure 1.310. The SSR position related D_1-relative density (pD_1RD) map of position at 15699001-15750000 bp of human reference Y-DNA (NC_000024.10) at resolution of 1 Kbp.

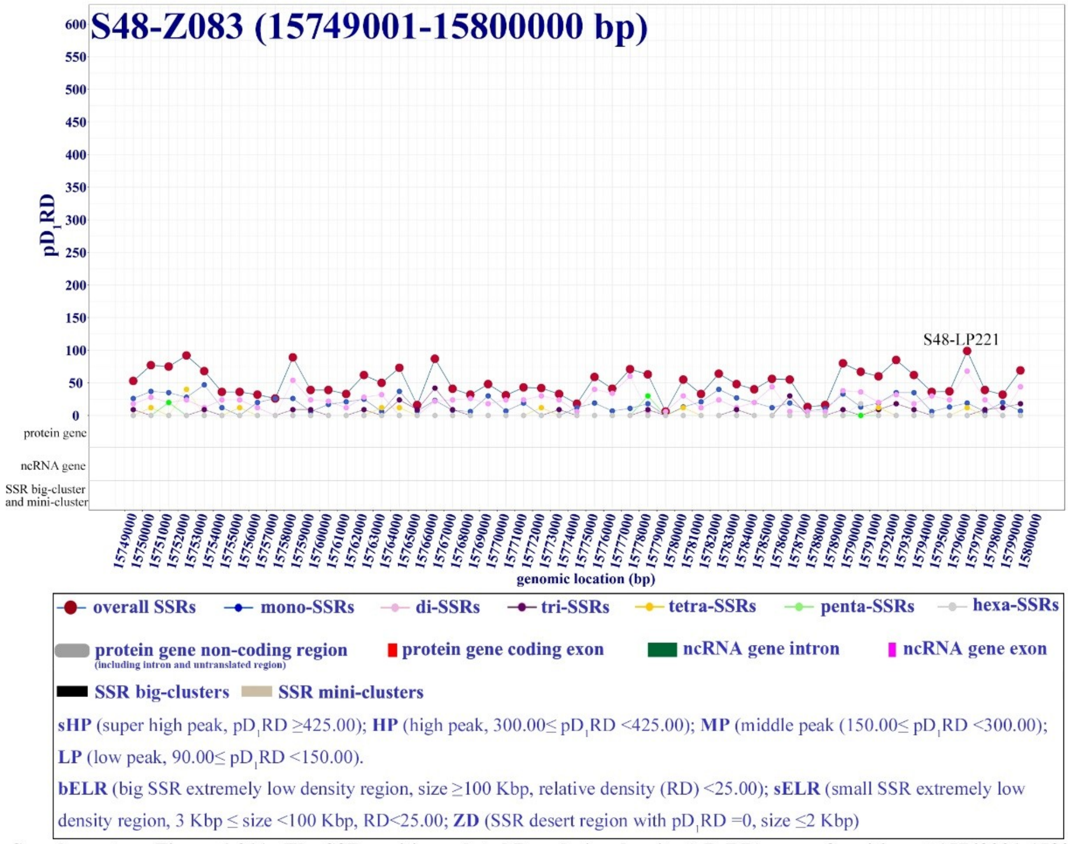

Supplementary Figure 1.311. The SSR position related D_1-relative density (pD_1RD) map of position at 15749001-15800000 bp of human reference Y-DNA (NC_000024.10) at resolution of 1 Kbp.

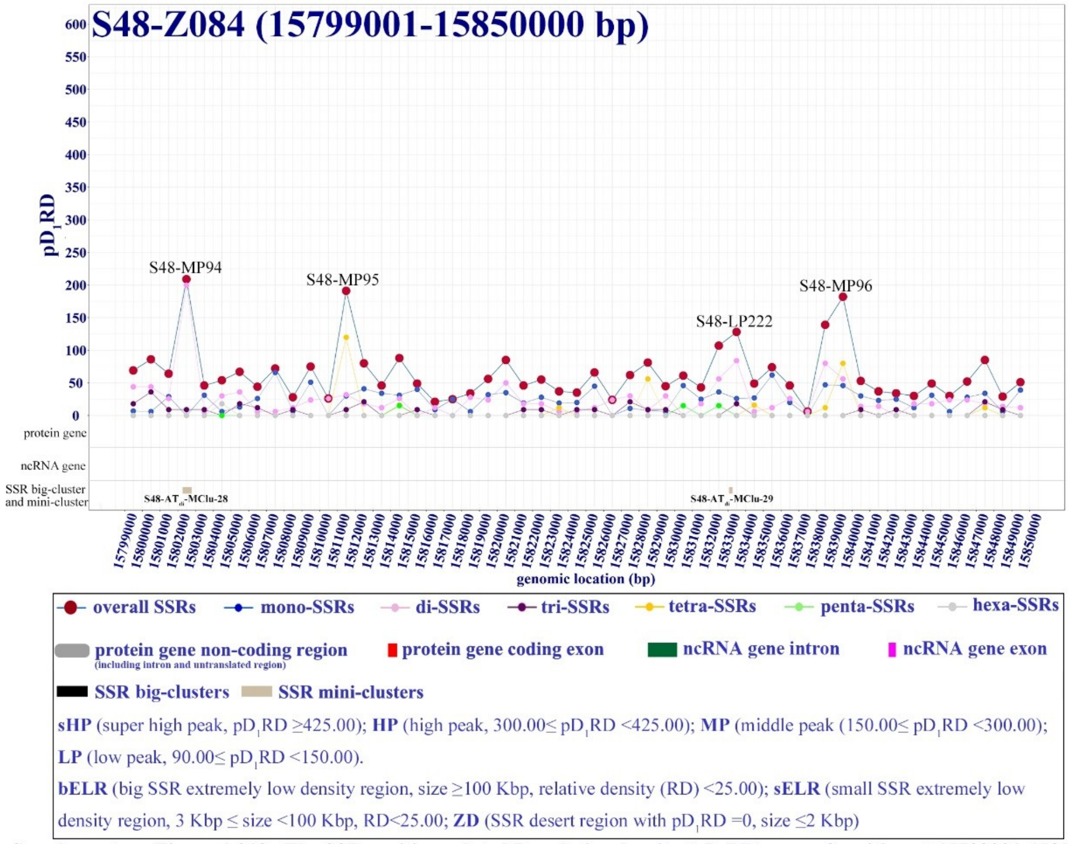

Supplementary Figure 1.312. The SSR position related D_1-relative density (pD_1RD) map of position at 15799001-15850000 bp of human reference Y-DNA (NC_000024.10) at resolution of 1 Kbp.

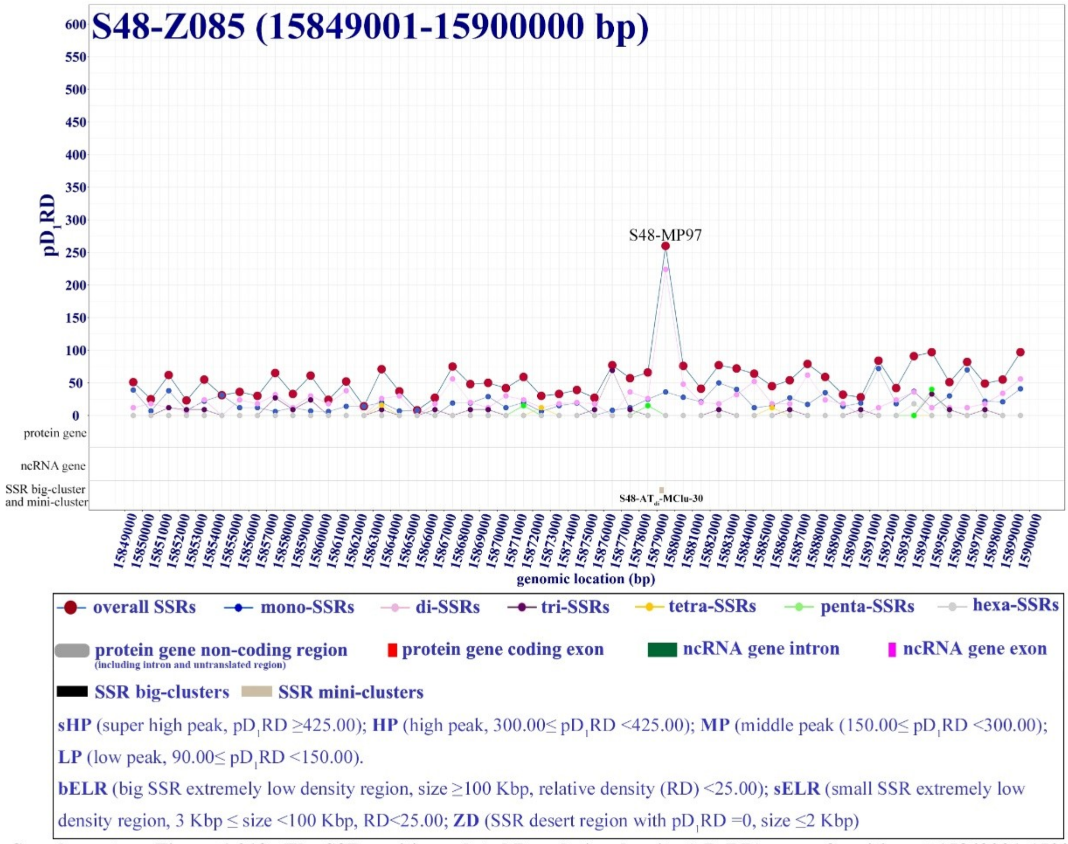

Supplementary Figure 1.313. The SSR position related D_1-relative density (pD_1RD) map of position at 15849001-15900000 bp of human reference Y-DNA (NC_000024.10) at resolution of 1 Kbp.

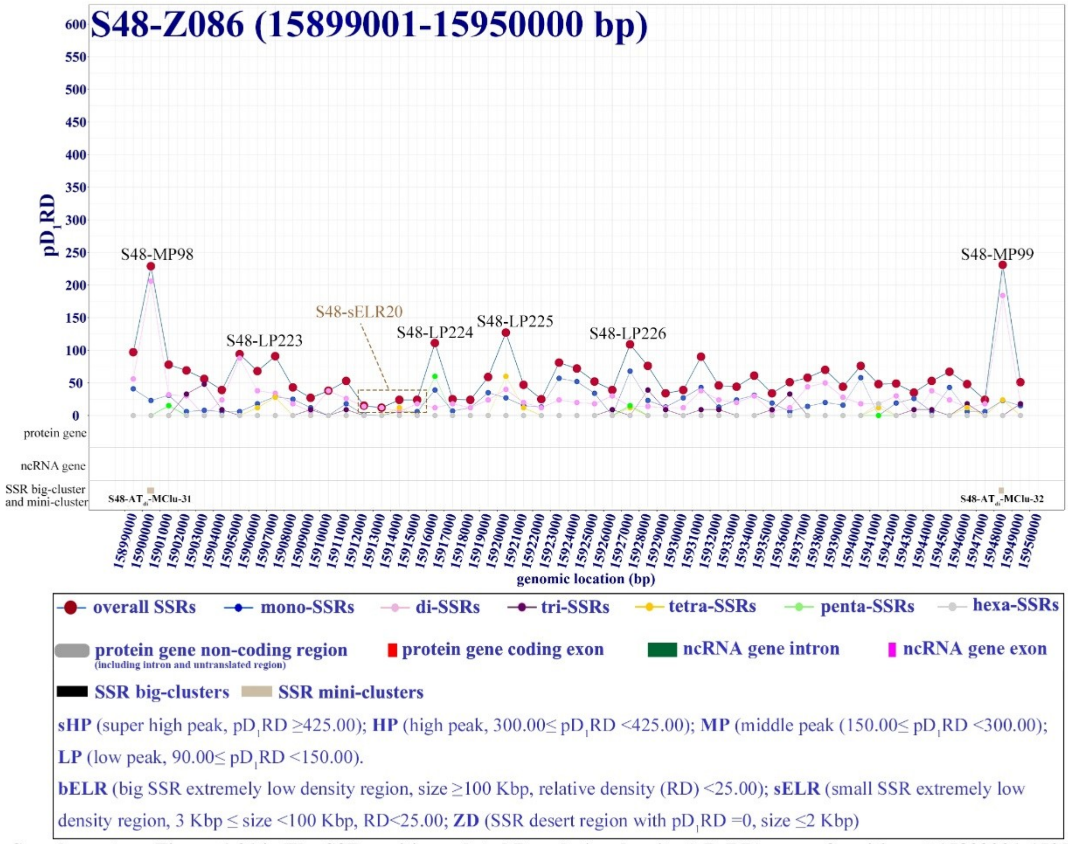

Supplementary Figure 1.314. The SSR position related D_1-relative density (pD_1RD) map of position at 15899001-15950000 bp of human reference Y-DNA (NC_000024.10) at resolution of 1 Kbp.

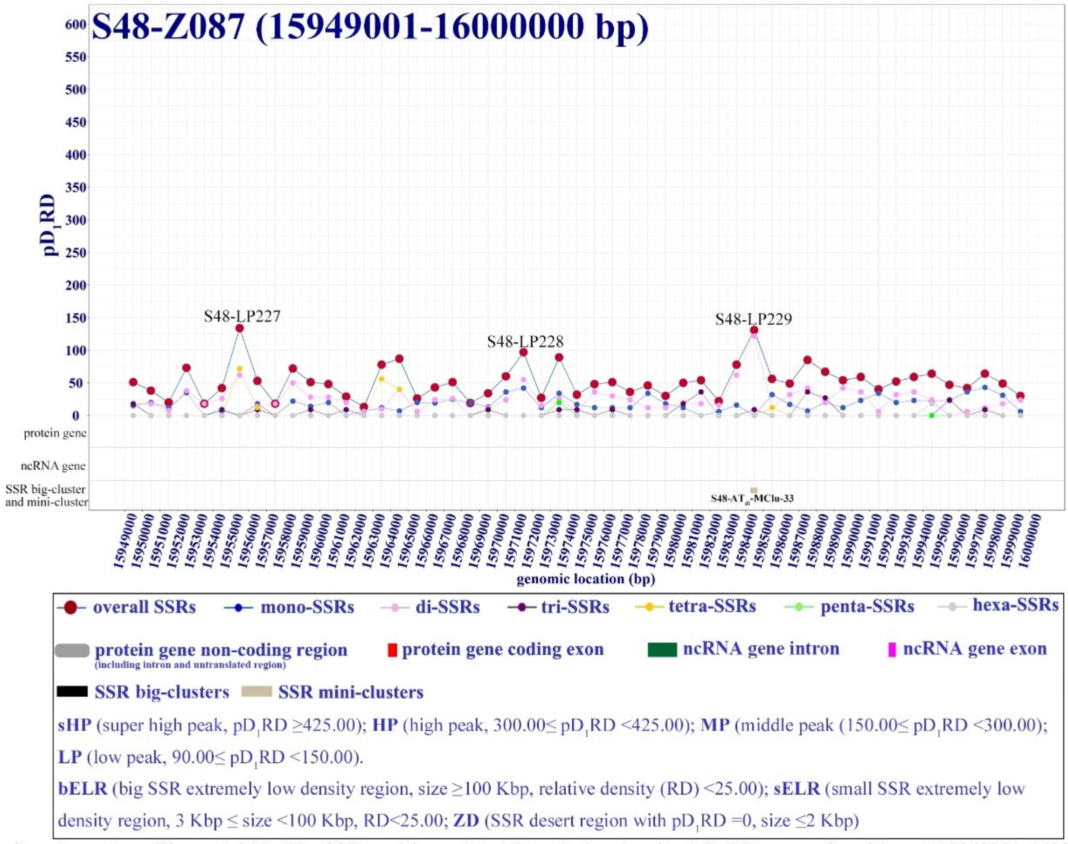

Supplementary Figure 1.315. The SSR position related D_1-relative density (pD_1RD) map of position at 15949001-16000000 bp of human reference Y-DNA (NC_000024.10) at resolution of 1 Kbp.

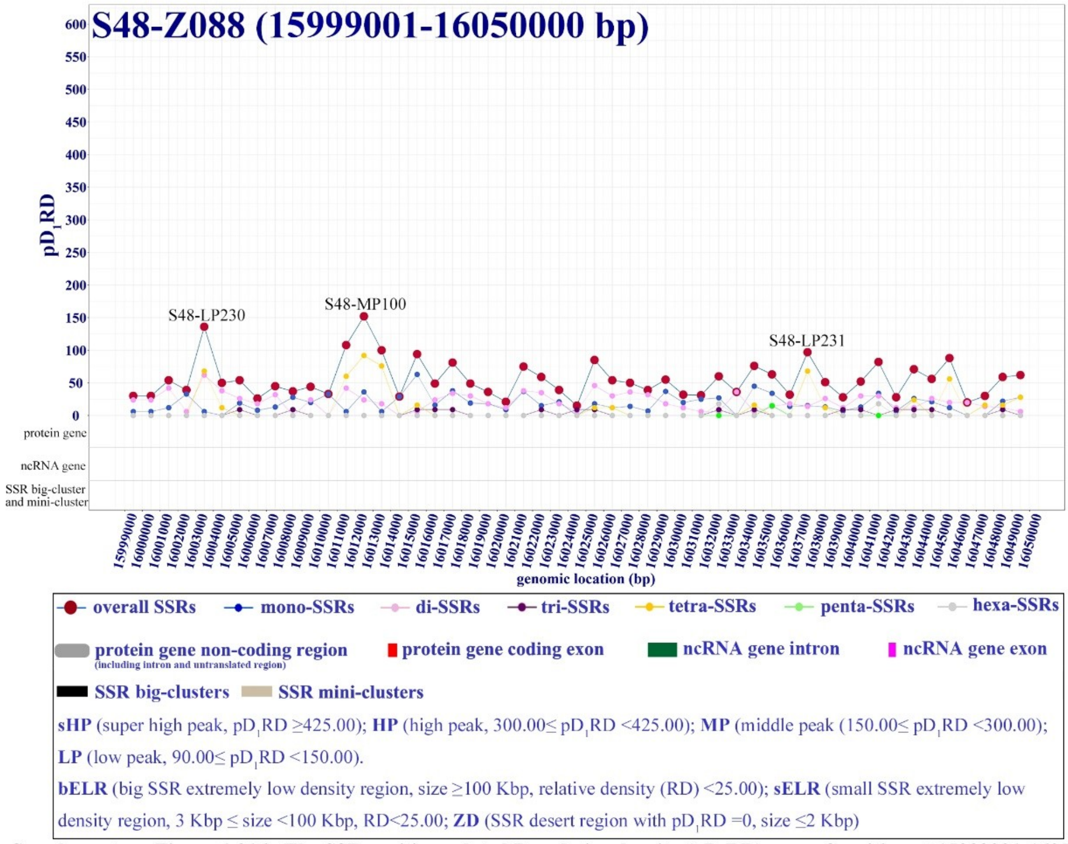

Supplementary Figure 1.316. The SSR position related D_1-relative density (pD_1RD) map of position at 15999001-16050000 bp of human reference Y-DNA (NC_000024.10) at resolution of 1 Kbp.

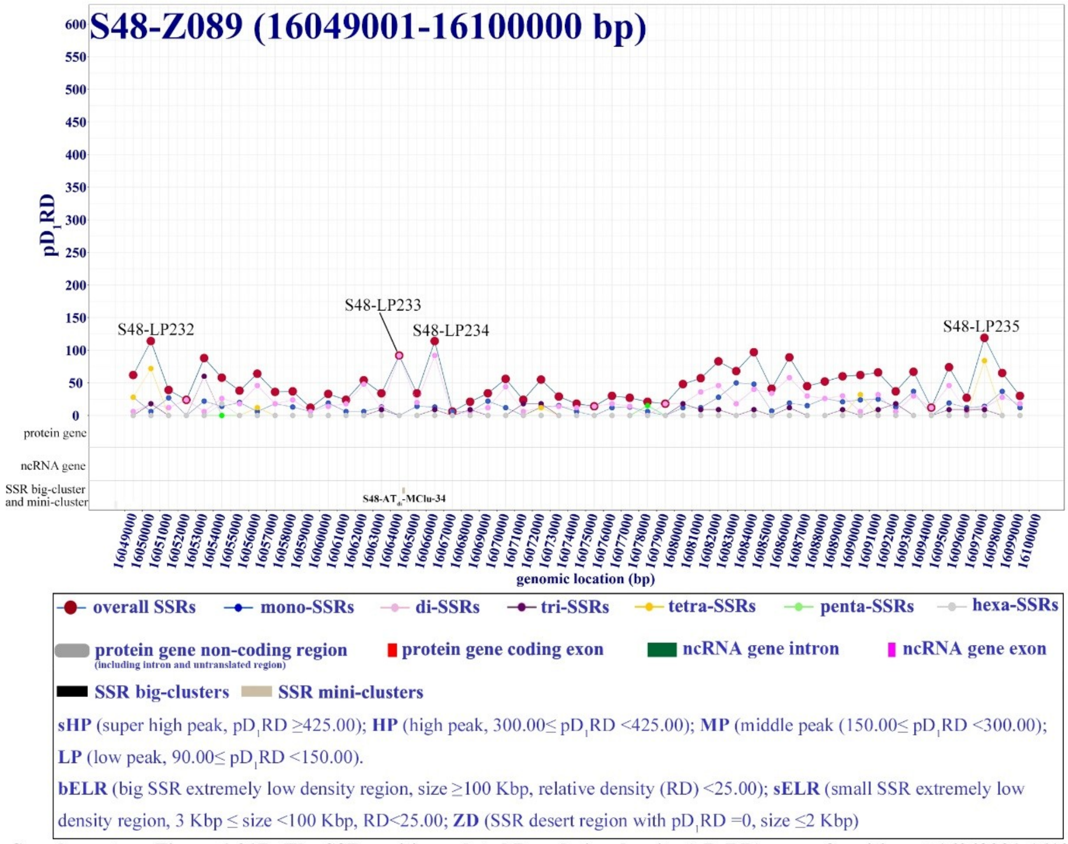

Supplementary Figure 1.317. The SSR position related D_1-relative density (pD_1RD) map of position at 16049001-16100000 bp of human reference Y-DNA (NC_000024.10) at resolution of 1 Kbp.

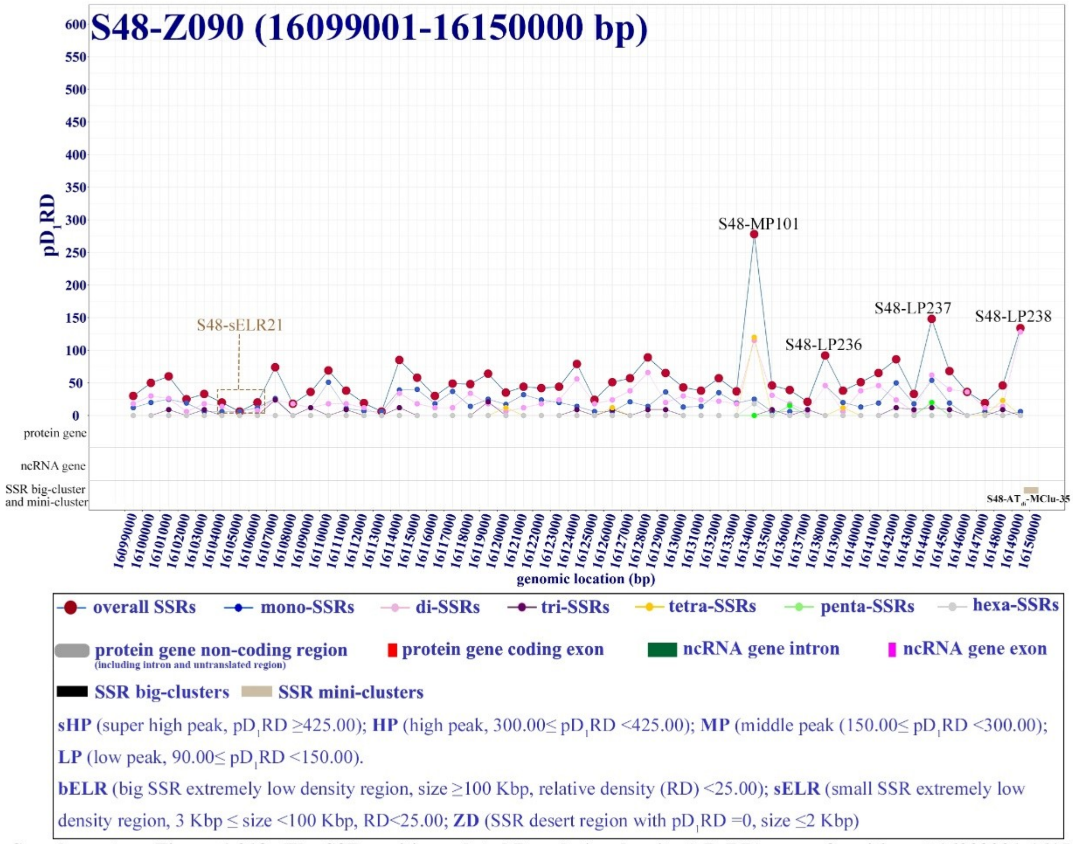

Supplementary Figure 1.318. The SSR position related D_1-relative density (pD_1RD) map of position at 16099001-16150000 bp of human reference Y-DNA (NC_000024.10) at resolution of 1 Kbp.

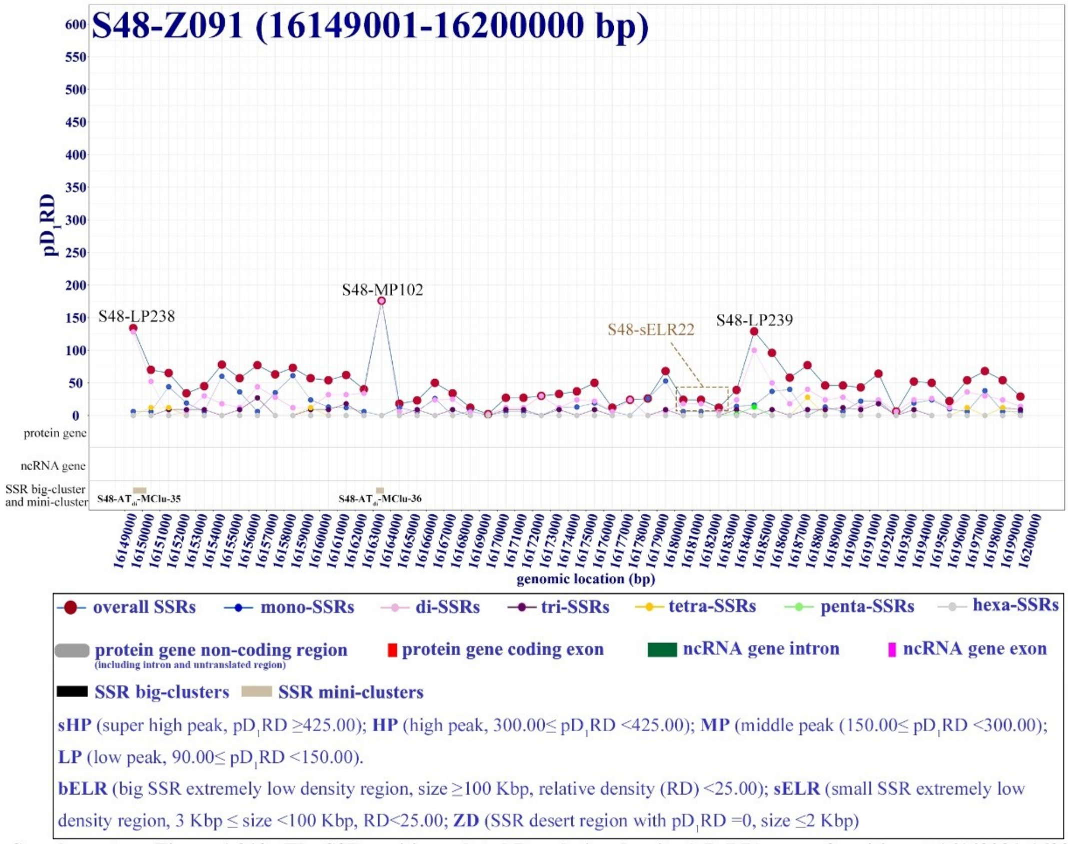

Supplementary Figure 1.319. The SSR position related D_1-relative density (pD_1RD) map of position at 16149001-16200000 bp of human reference Y-DNA (NC_000024.10) at resolution of 1 Kbp.

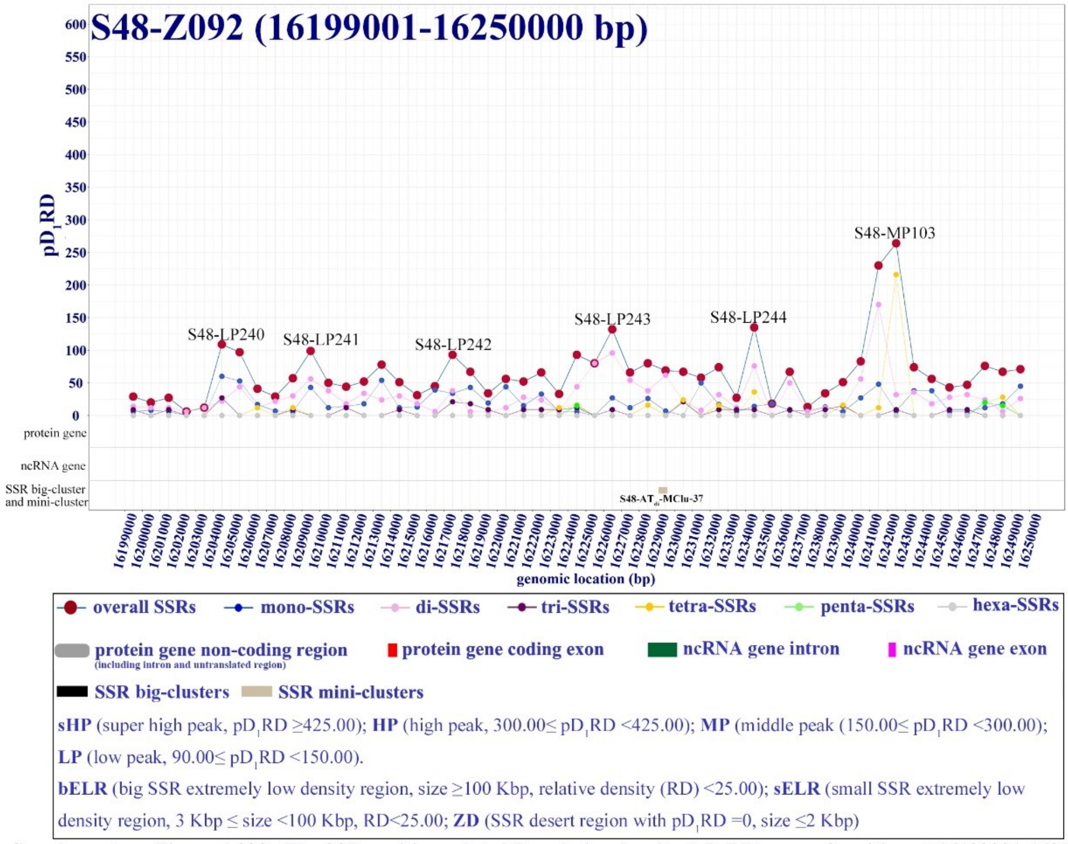

Supplementary Figure 1.320. The SSR position related D_1-relative density (pD_1RD) map of position at 16199001-16250000 bp of human reference Y-DNA (NC_000024.10) at resolution of 1 Kbp.

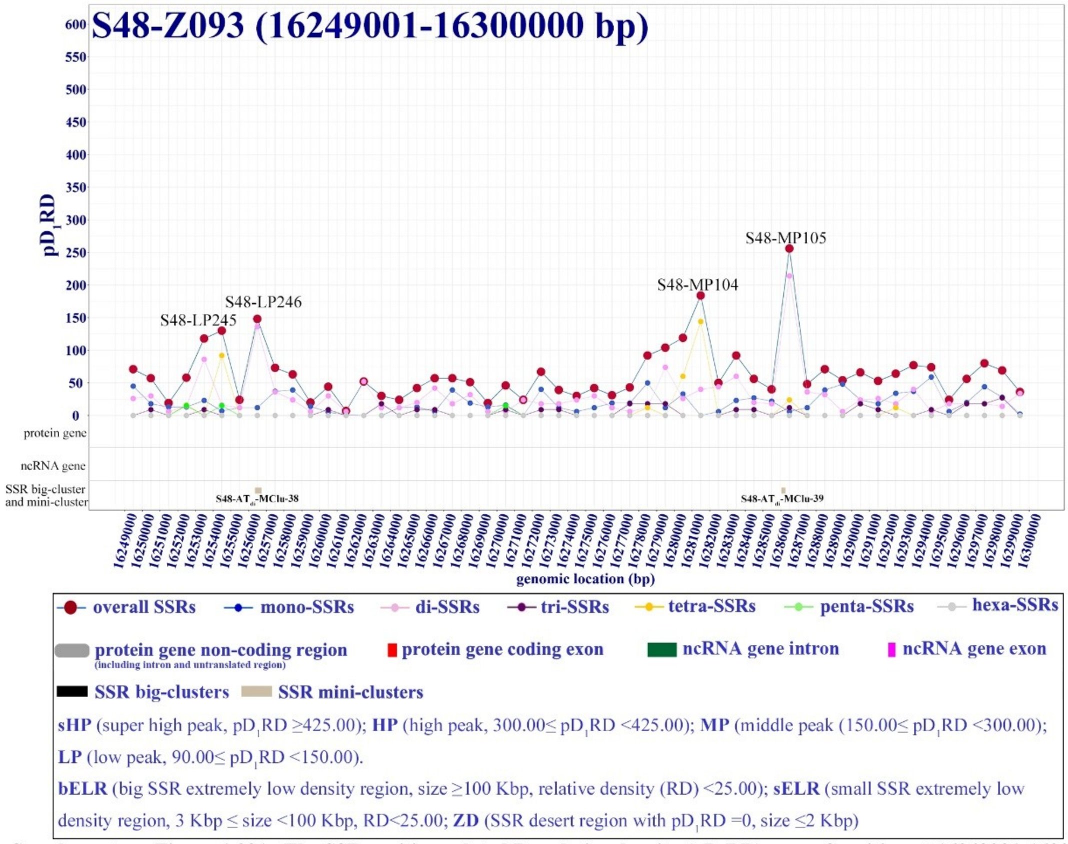

Supplementary Figure 1.321. The SSR position related D_1-relative density (pD_1RD) map of position at 16249001-16300000 bp of human reference Y-DNA (NC_000024.10) at resolution of 1 Kbp.

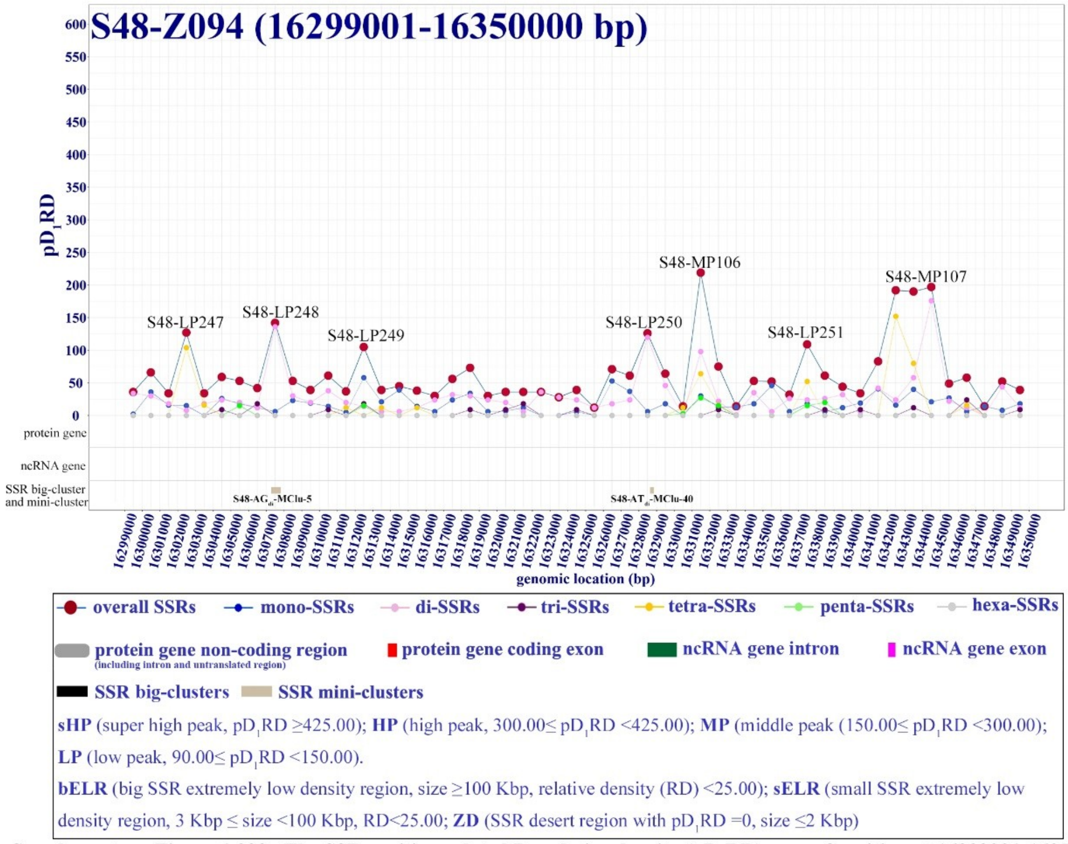

Supplementary Figure 1.322. The SSR position related D_1-relative density (pD_1RD) map of position at 16299001-16350000 bp of human reference Y-DNA (NC_000024.10) at resolution of 1 Kbp.

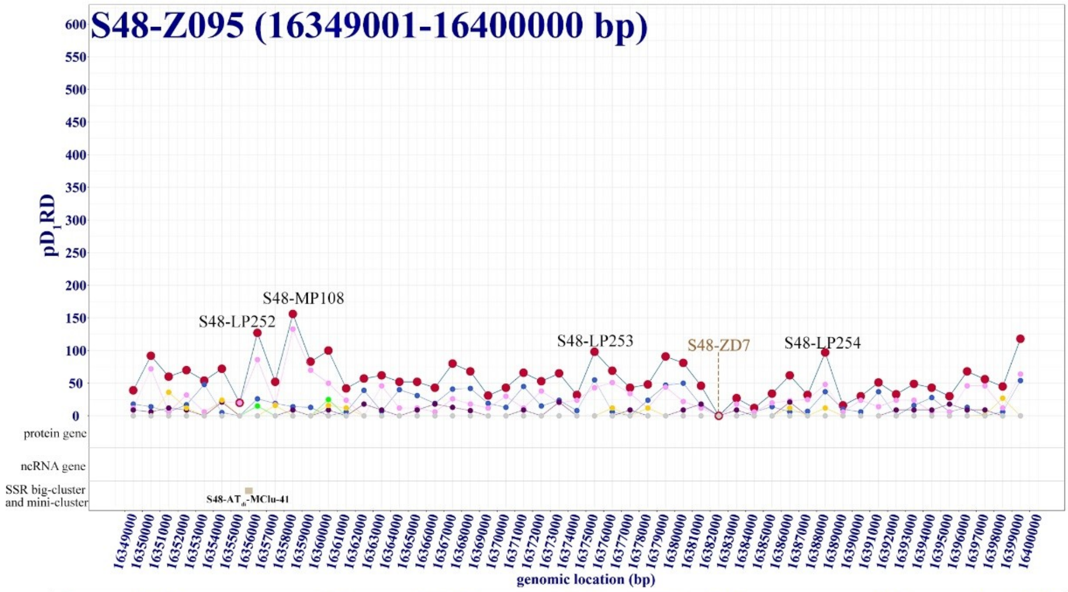

overall SSRs · mono-SSRs · di-SSRs · tri-SSRs · tetra-SSRs · penta-SSRs · hexa-SSRs

protein gene non-coding region (including intron and untranslated region) · protein gene coding exon · ncRNA gene intron · ncRNA gene exon

SSR big-clusters · SSR mini-clusters

sHP (super high peak, $pD_1RD \geq 425.00$); **HP** (high peak, $300.00 \leq pD_1RD < 425.00$); **MP** (middle peak ($150.00 \leq pD_1RD < 300.00$); **LP** (low peak, $90.00 \leq pD_1RD < 150.00$).

bELR (big SSR extremely low density region, size ≥100 Kbp, relative density (RD) <25.00); **sELR** (small SSR extremely low density region, 3 Kbp ≤ size <100 Kbp, RD<25.00; **ZD** (SSR desert region with $pD_1RD = 0$, size ≤2 Kbp)

Supplementary Figure 1.323. The SSR position related D_1-relative density (pD_1RD) map of position at 16349001-16400000 bp of human reference Y-DNA (NC_000024.10) at resolution of 1 Kbp.

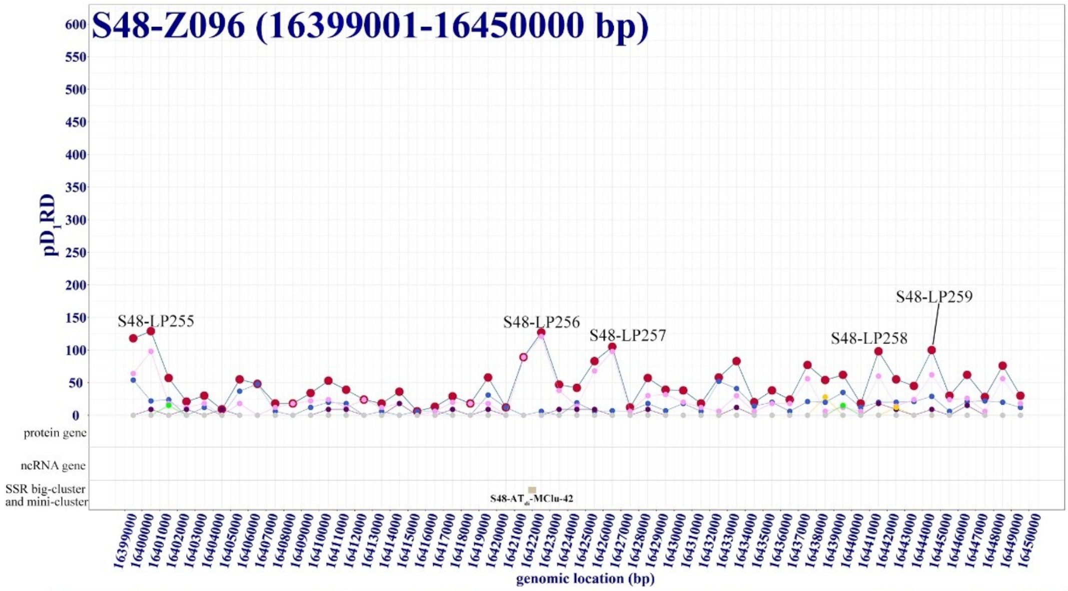

overall SSRs · mono-SSRs · di-SSRs · tri-SSRs · tetra-SSRs · penta-SSRs · hexa-SSRs

protein gene non-coding region (including intron and untranslated region) · protein gene coding exon · ncRNA gene intron · ncRNA gene exon

SSR big-clusters · SSR mini-clusters

sHP (super high peak, $pD_1RD \geq 425.00$); **HP** (high peak, $300.00 \leq pD_1RD < 425.00$); **MP** (middle peak ($150.00 \leq pD_1RD < 300.00$); **LP** (low peak, $90.00 \leq pD_1RD < 150.00$).

bELR (big SSR extremely low density region, size ≥100 Kbp, relative density (RD) <25.00); **sELR** (small SSR extremely low density region, 3 Kbp ≤ size <100 Kbp, RD<25.00; **ZD** (SSR desert region with $pD_1RD = 0$, size ≤2 Kbp)

Supplementary Figure 1.324. The SSR position related D_1-relative density (pD_1RD) map of position at 16399001-16450000 bp of human reference Y-DNA (NC_000024.10) at resolution of 1 Kbp.

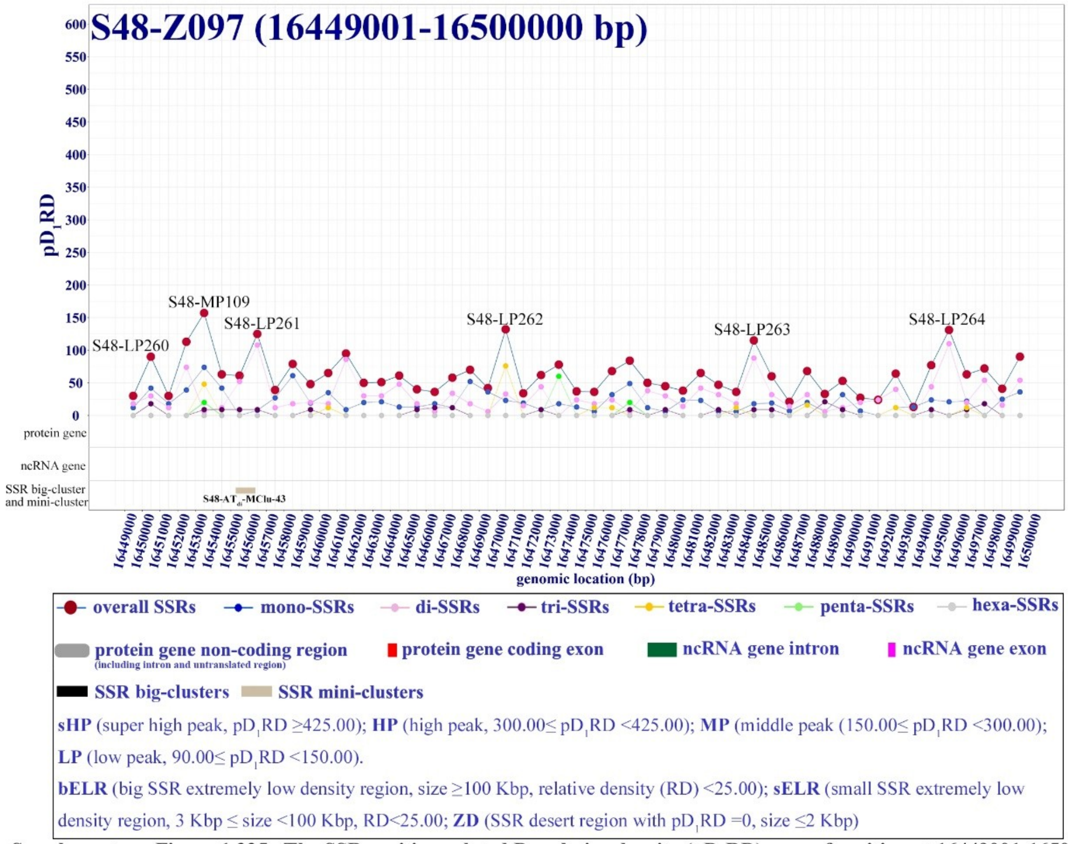

Supplementary Figure 1.325. The SSR position related D_1-relative density (pD_1RD) map of position at 16449001-16500000 bp of human reference Y-DNA (NC_000024.10) at resolution of 1 Kbp.

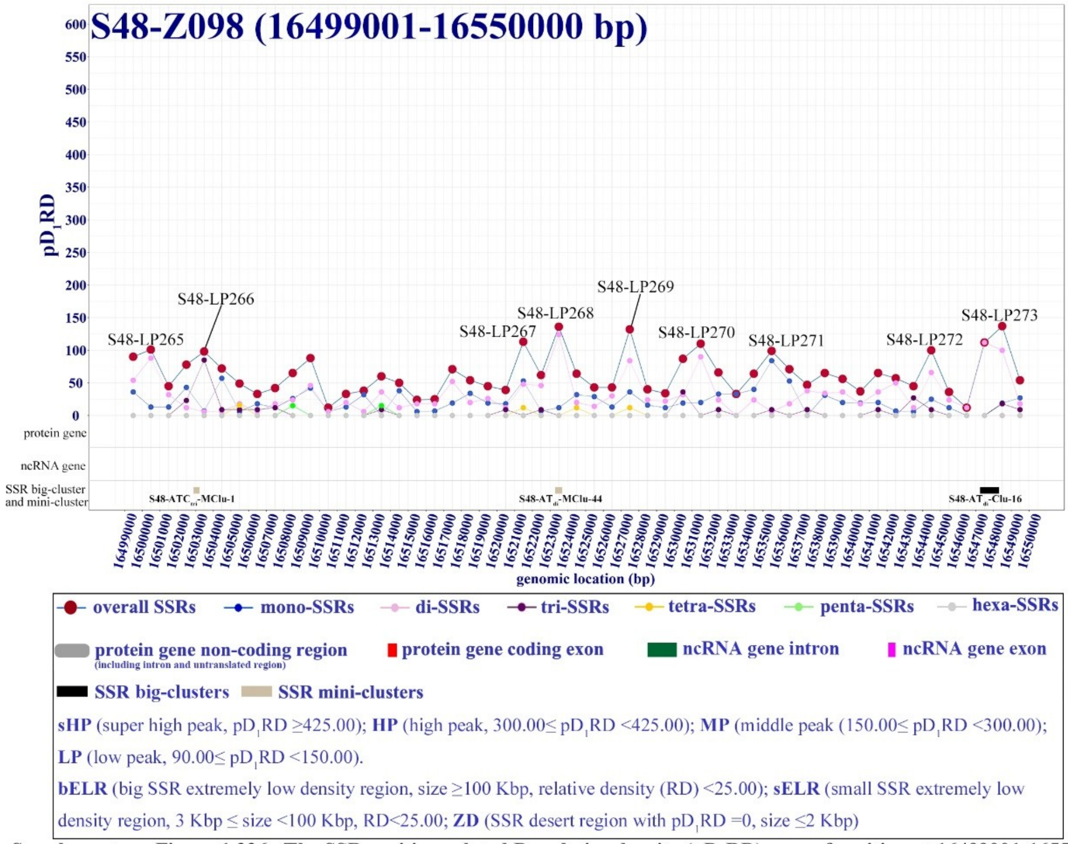

Supplementary Figure 1.326. The SSR position related D_1-relative density (pD_1RD) map of position at 16499001-16550000 bp of human reference Y-DNA (NC_000024.10) at resolution of 1 Kbp.

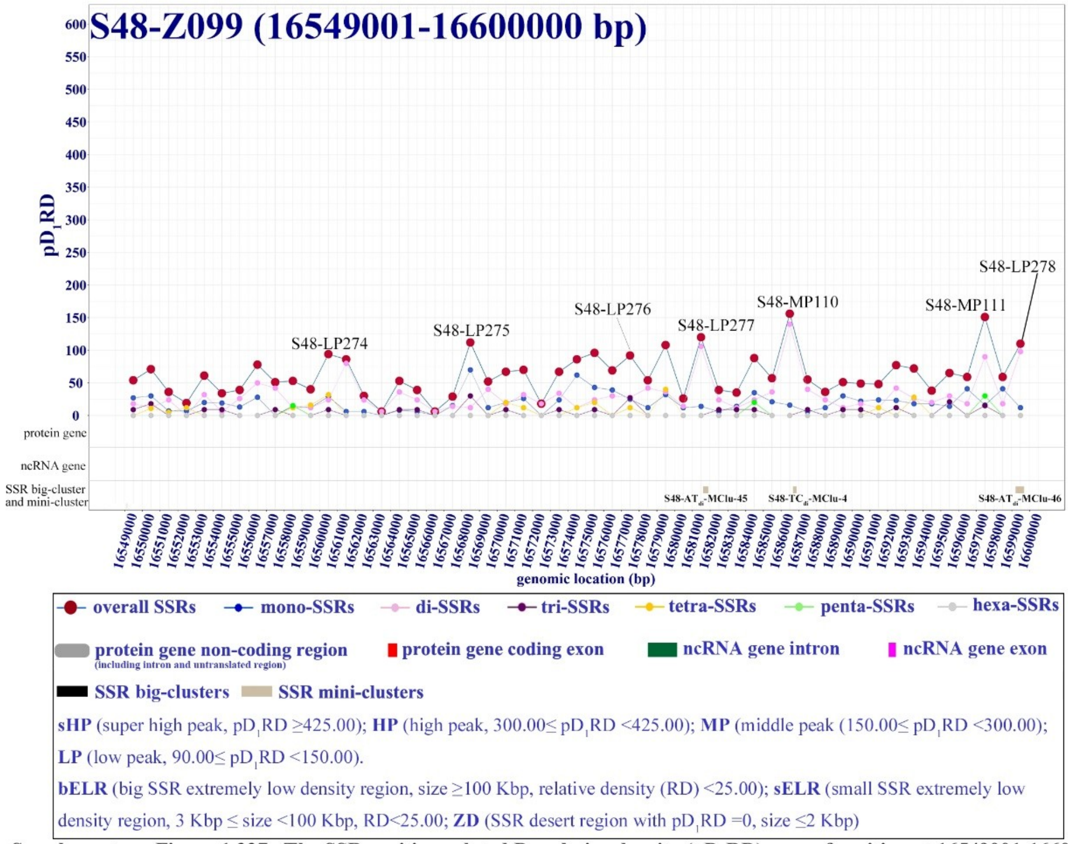

Supplementary Figure 1.327. The SSR position related D_1-relative density (pD_1RD) map of position at 16549001-16600000 bp of human reference Y-DNA (NC_000024.10) at resolution of 1 Kbp.

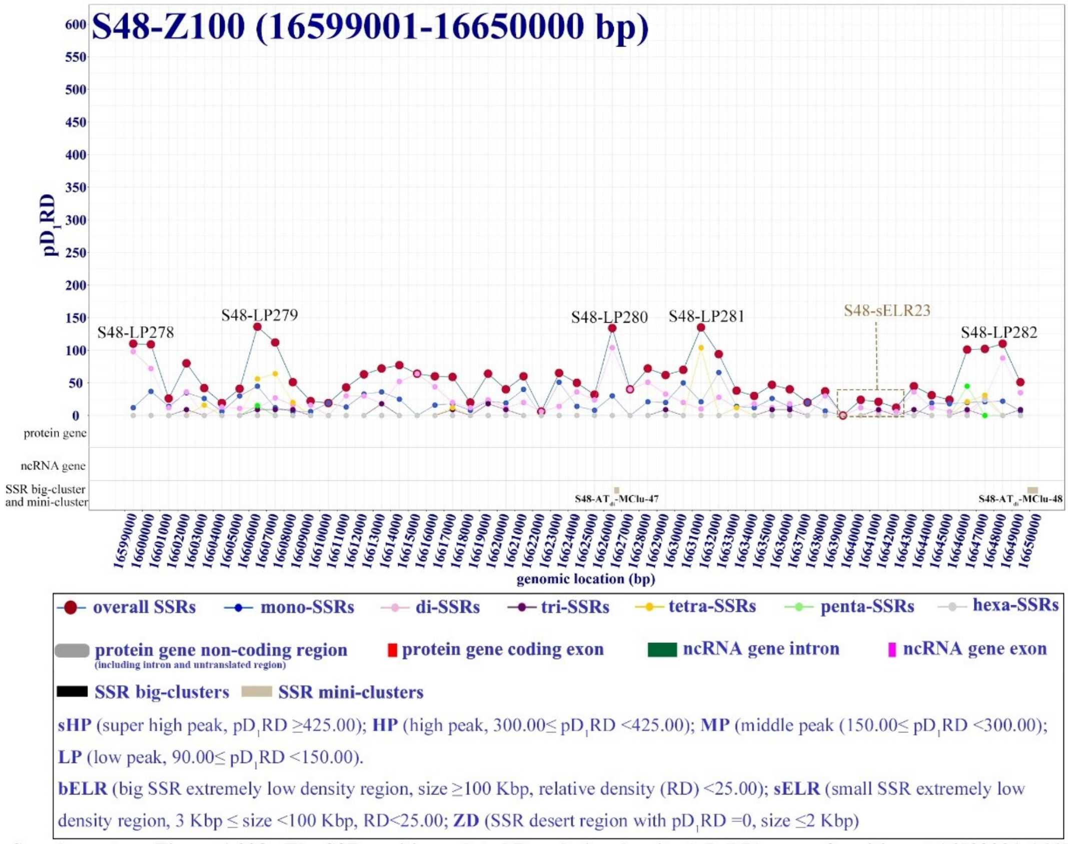

Supplementary Figure 1.328. The SSR position related D_1-relative density (pD_1RD) map of position at 16599001-16650000 bp of human reference Y-DNA (NC_000024.10) at resolution of 1 Kbp.

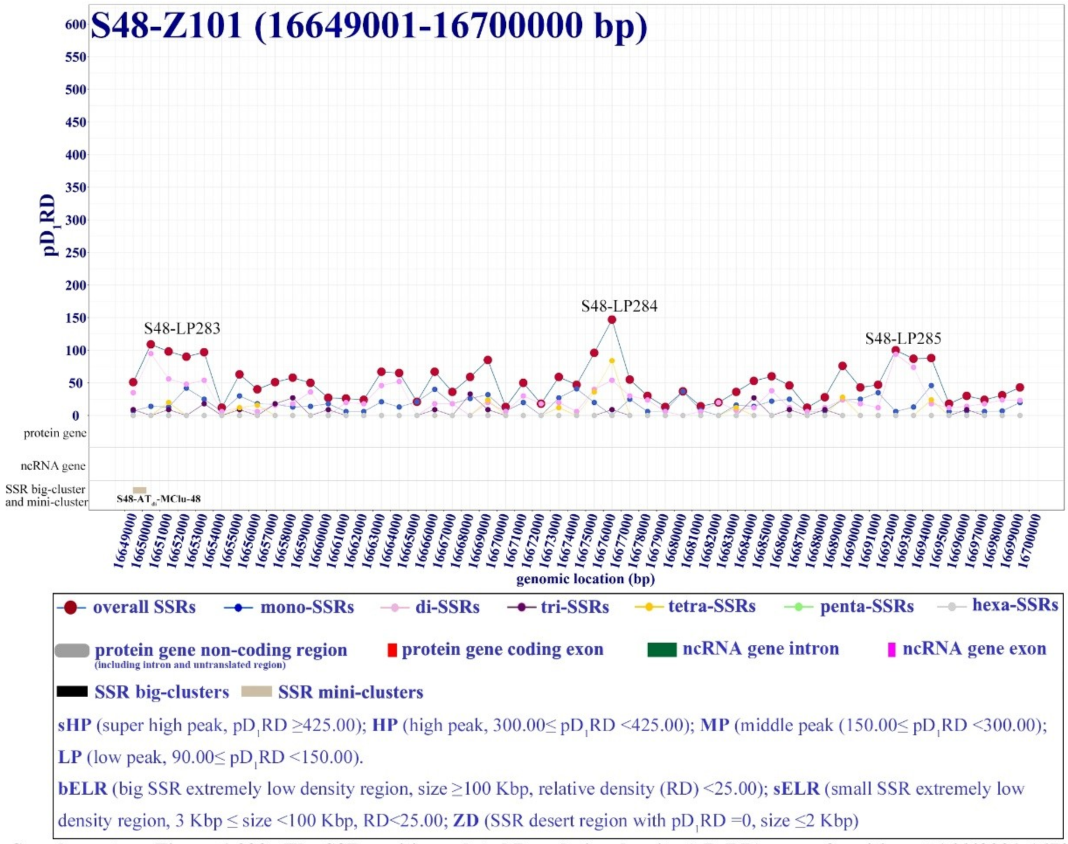

Supplementary Figure 1.329. The SSR position related D_1-relative density (pD_1RD) map of position at 16649001-16700000 bp of human reference Y-DNA (NC_000024.10) at resolution of 1 Kbp.

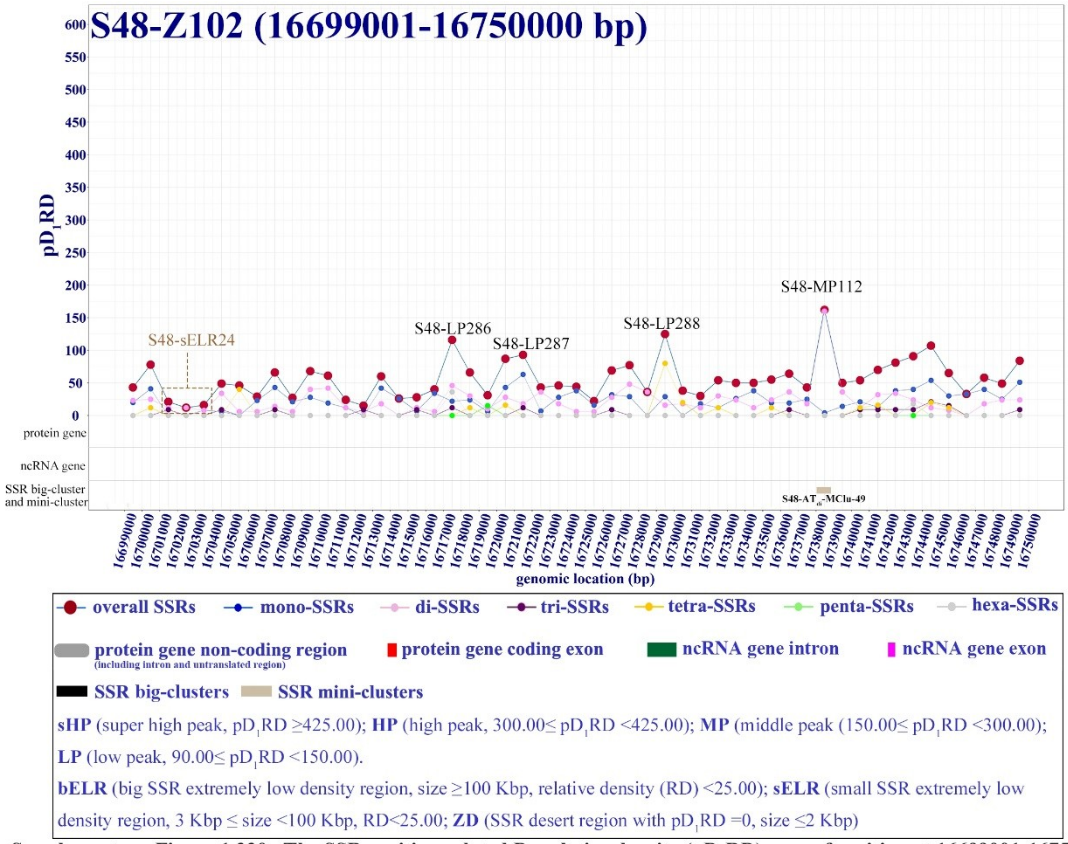

Supplementary Figure 1.330. The SSR position related D_1-relative density (pD_1RD) map of position at 16699001-16750000 bp of human reference Y-DNA (NC_000024.10) at resolution of 1 Kbp.

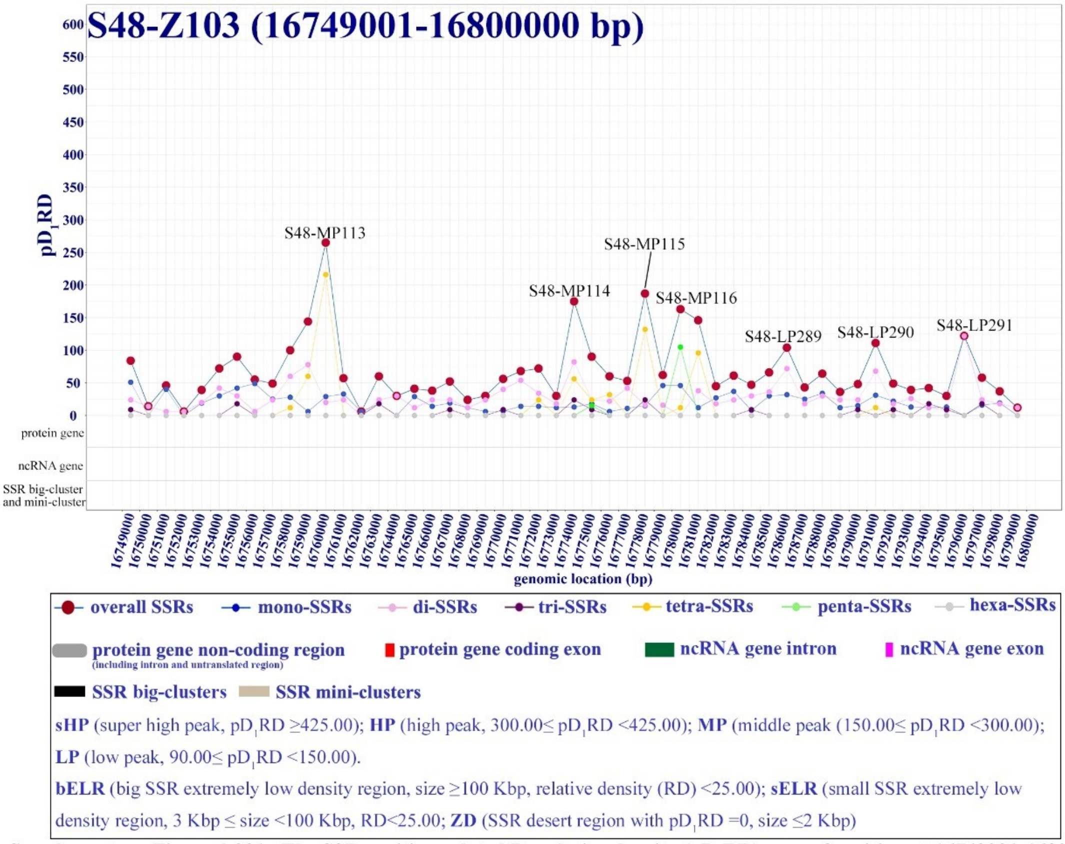

Supplementary Figure 1.331. The SSR position related D_1-relative density (pD_1RD) map of position at 16749001-16800000 bp of human reference Y-DNA (NC_000024.10) at resolution of 1 Kbp.

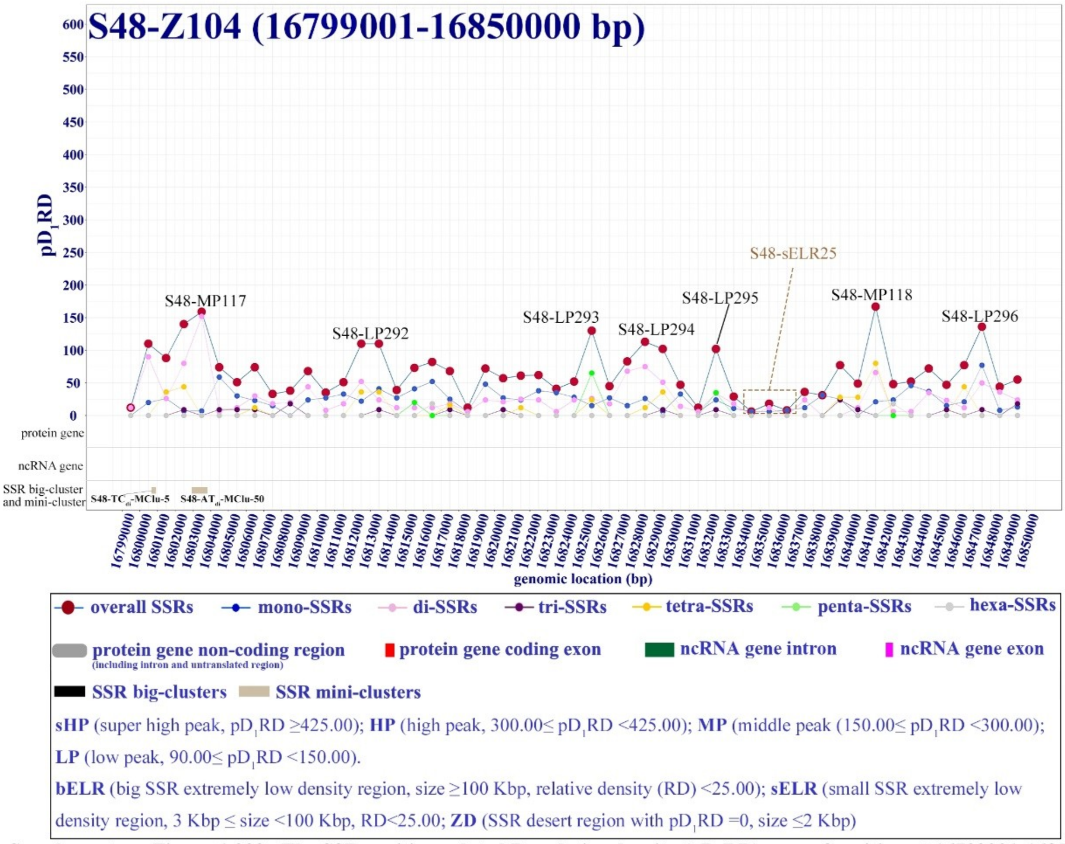

Supplementary Figure 1.332. The SSR position related D_1-relative density (pD_1RD) map of position at 16799001-16850000 bp of human reference Y-DNA (NC_000024.10) at resolution of 1 Kbp.

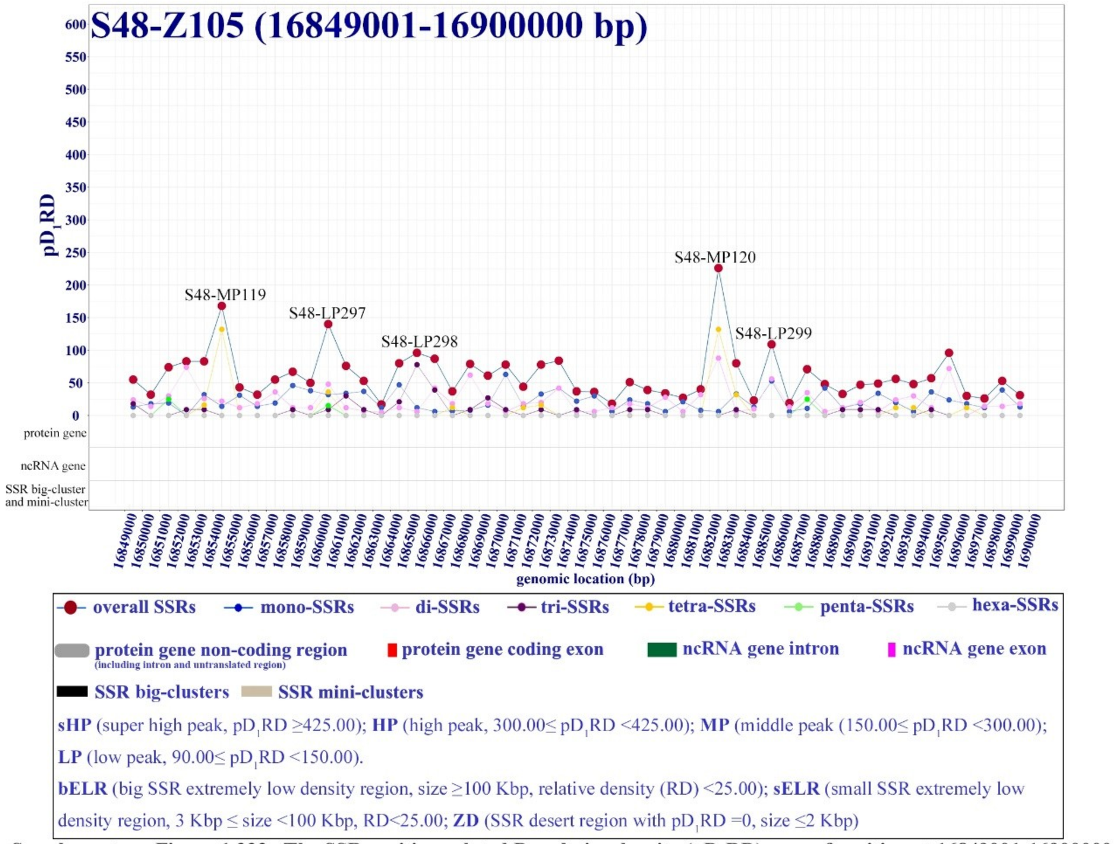

Supplementary Figure 1.333. The SSR position related D_1-relative density (pD_1RD) map of position at 16849001-16900000 bp of human reference Y-DNA (NC_000024.10) at resolution of 1 Kbp.

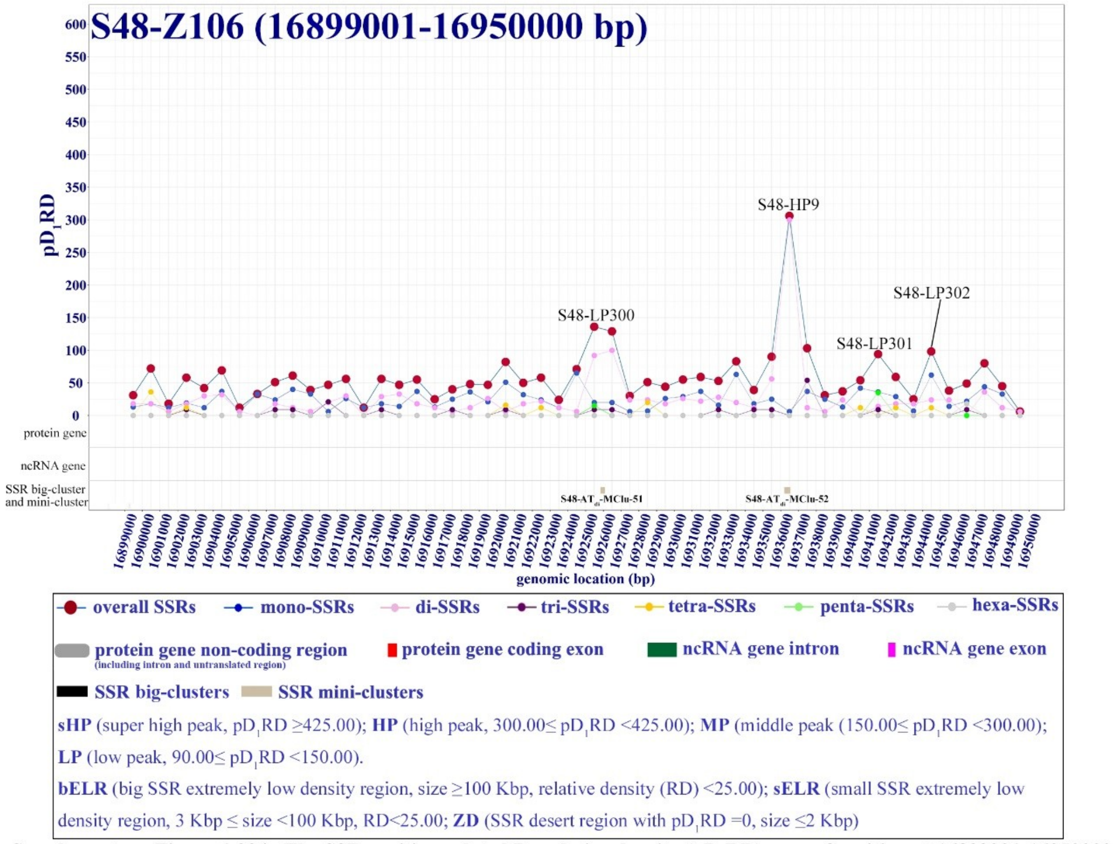

Supplementary Figure 1.334. The SSR position related D_1-relative density (pD_1RD) map of position at 16899001-16950000 bp of human reference Y-DNA (NC_000024.10) at resolution of 1 Kbp.

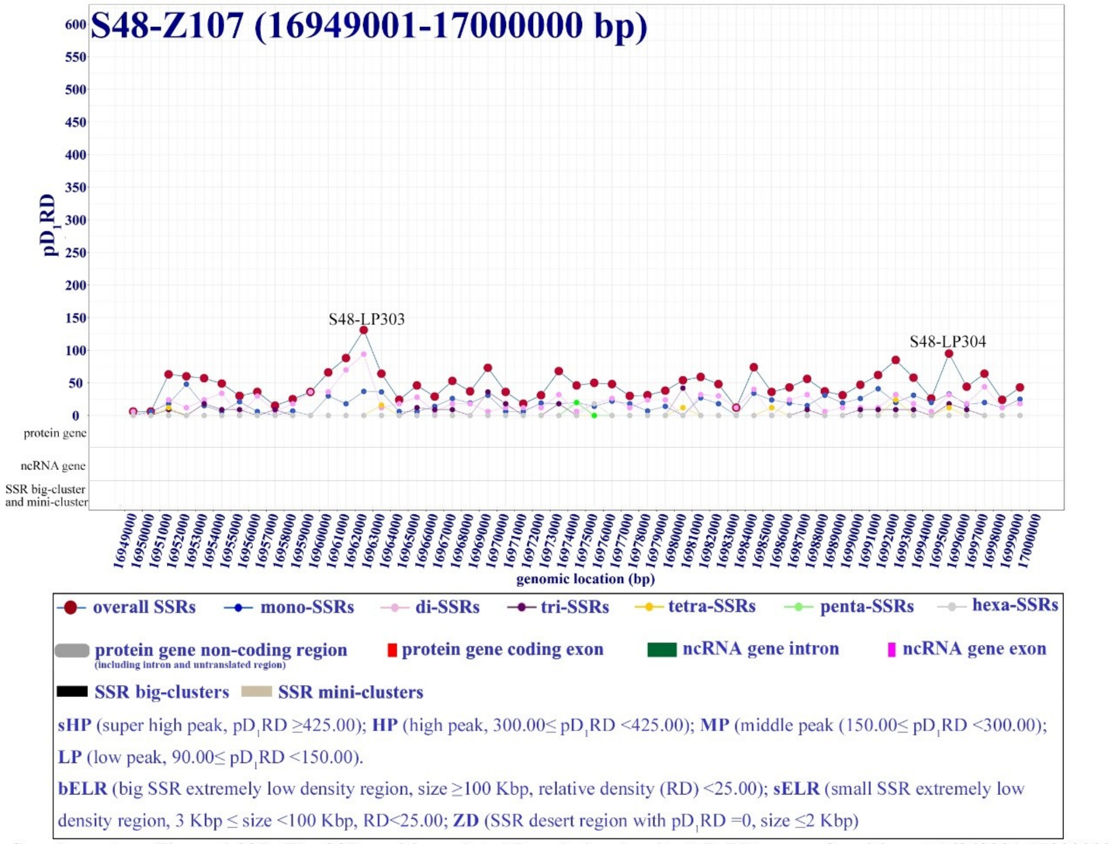

Supplementary Figure 1.335. The SSR position related D_1-relative density (pD_1RD) map of position at 16949001-17000000 bp of human reference Y-DNA (NC_000024.10) at resolution of 1 Kbp.

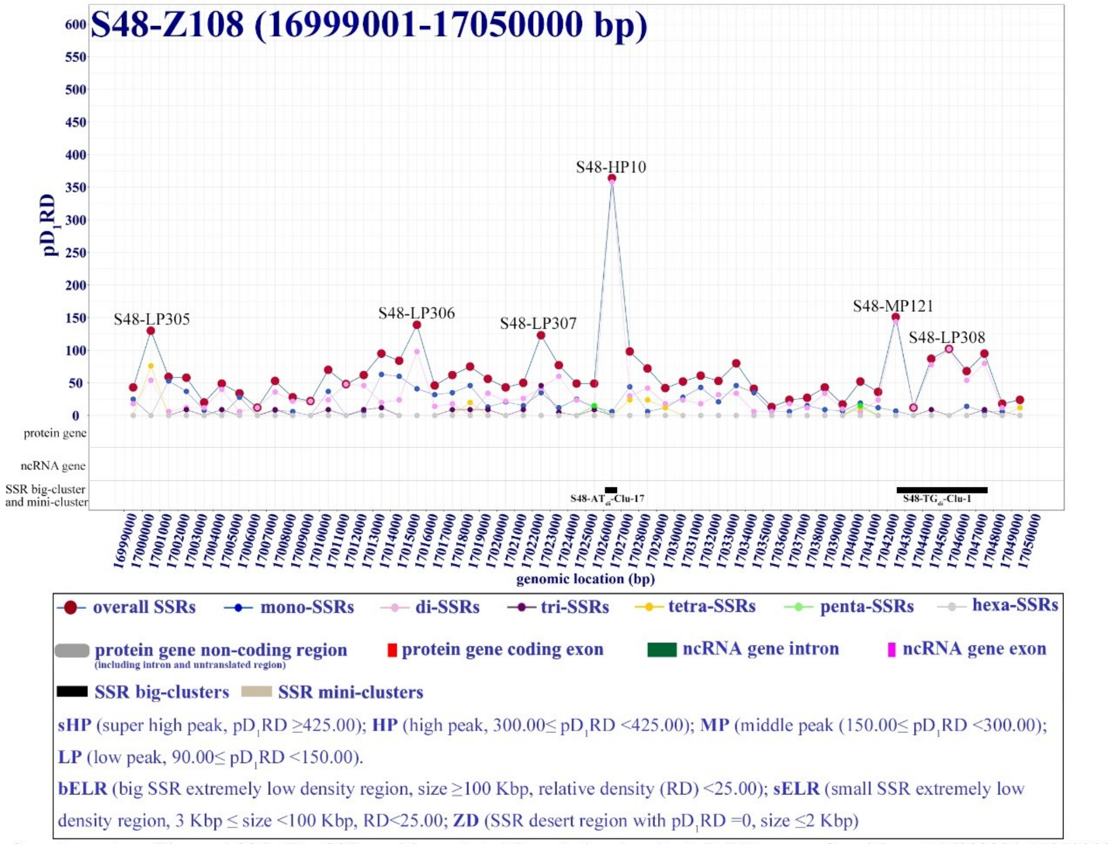

Supplementary Figure 1.336. The SSR position related D_1-relative density (pD_1RD) map of position at 16999001-17050000 bp of human reference Y-DNA (NC_000024.10) at resolution of 1 Kbp.

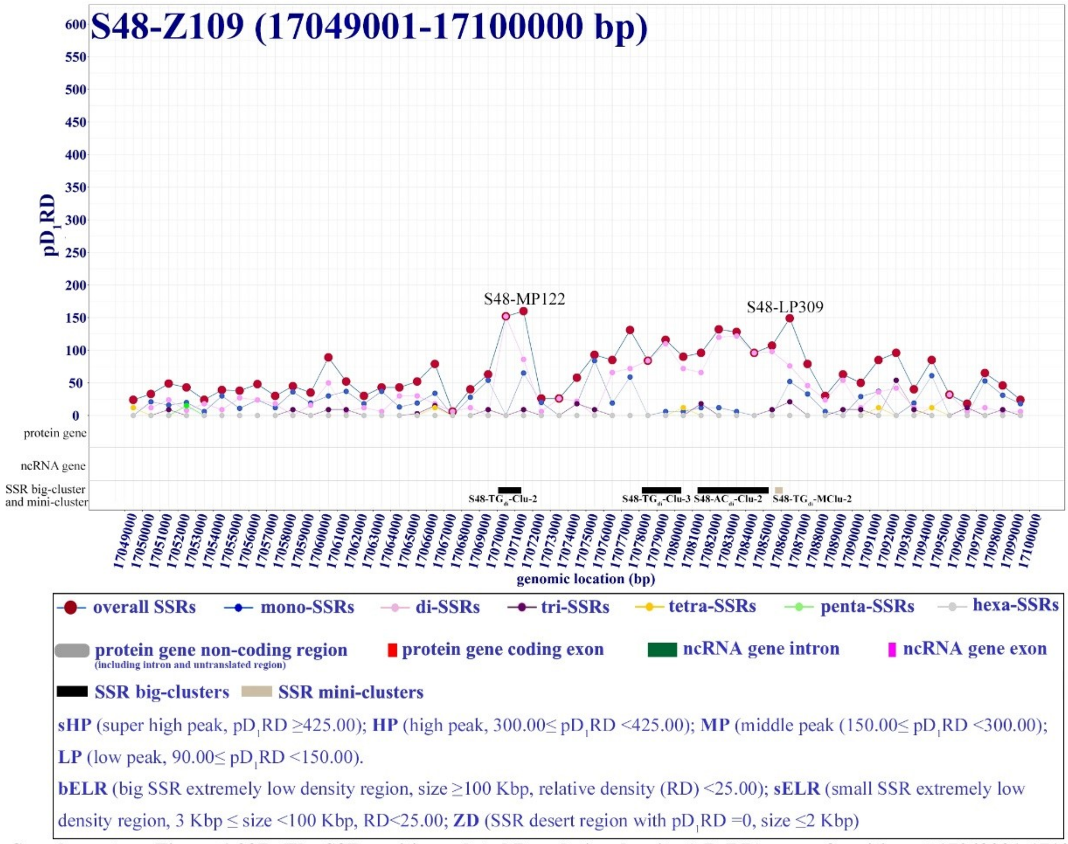

Supplementary Figure 1.337. The SSR position related D_1-relative density (pD_1RD) map of position at 17049001-17100000 bp of human reference Y-DNA (NC_000024.10) at resolution of 1 Kbp.

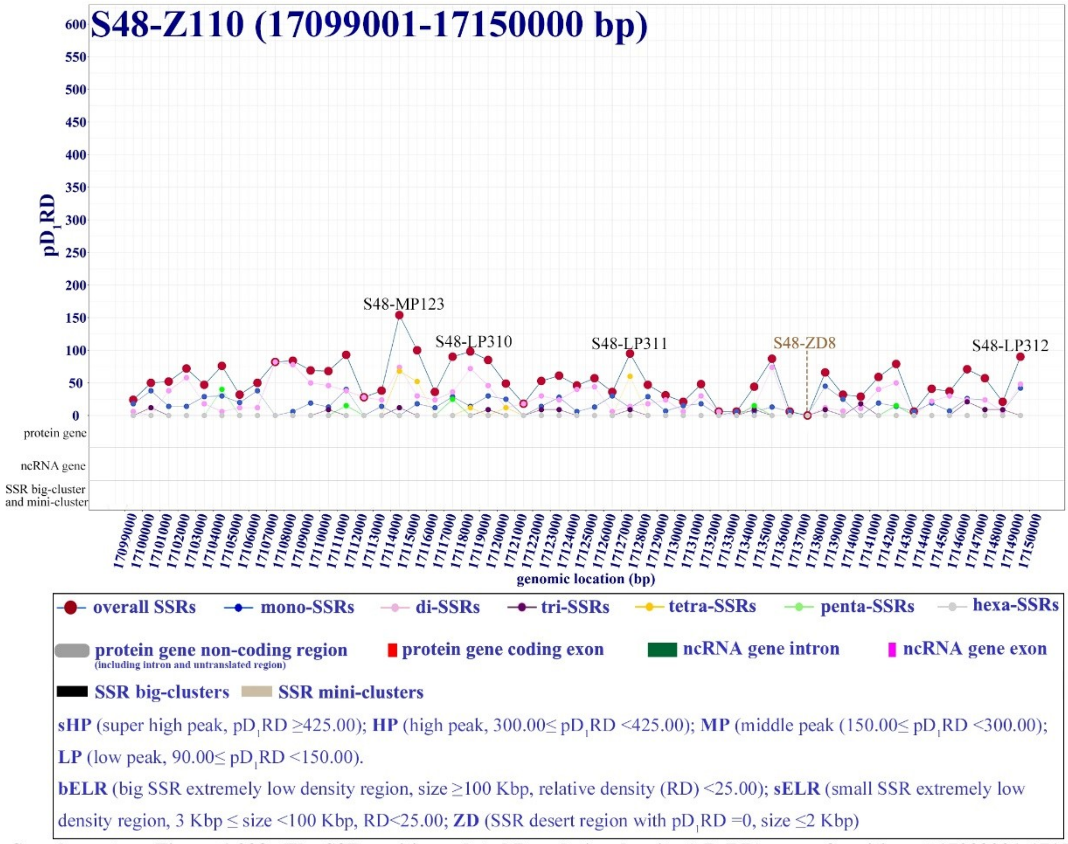

Supplementary Figure 1.338. The SSR position related D_1-relative density (pD_1RD) map of position at 17099001-17150000 bp of human reference Y-DNA (NC_000024.10) at resolution of 1 Kbp.

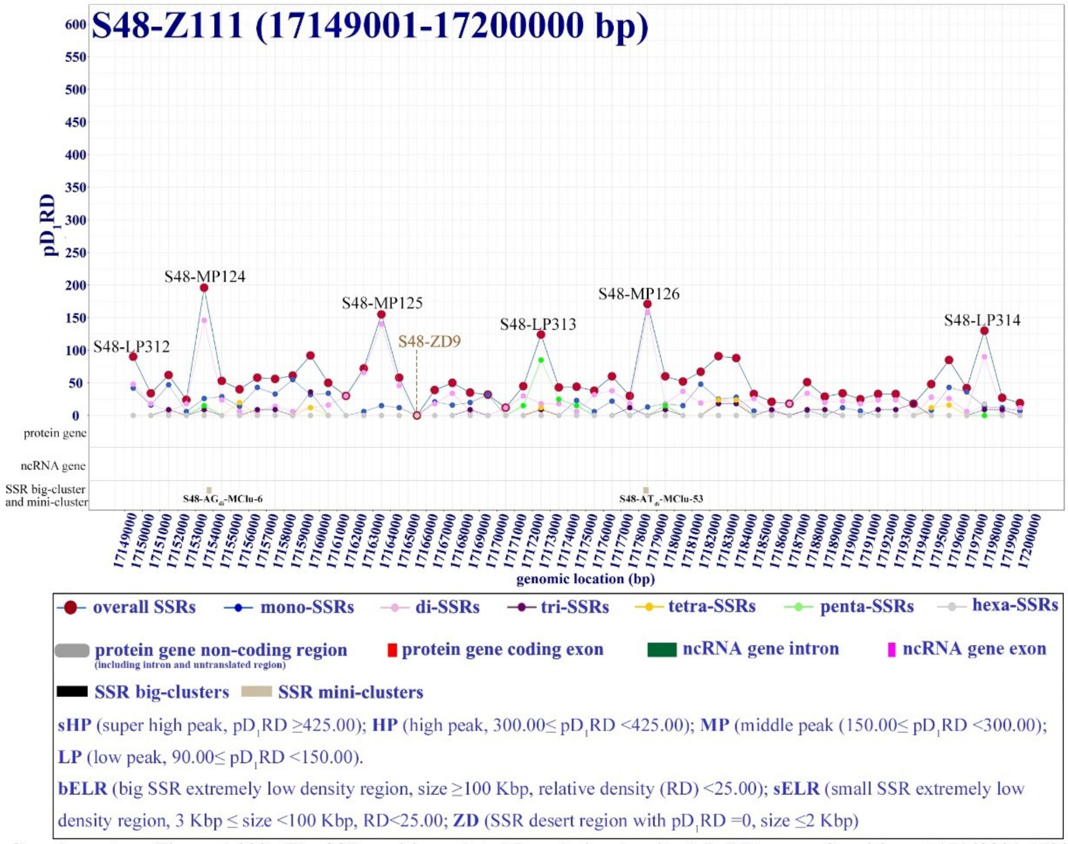

Supplementary Figure 1.339. The SSR position related D_1-relative density (pD_1RD) map of position at 17149001-17200000 bp of human reference Y-DNA (NC_000024.10) at resolution of 1 Kbp.

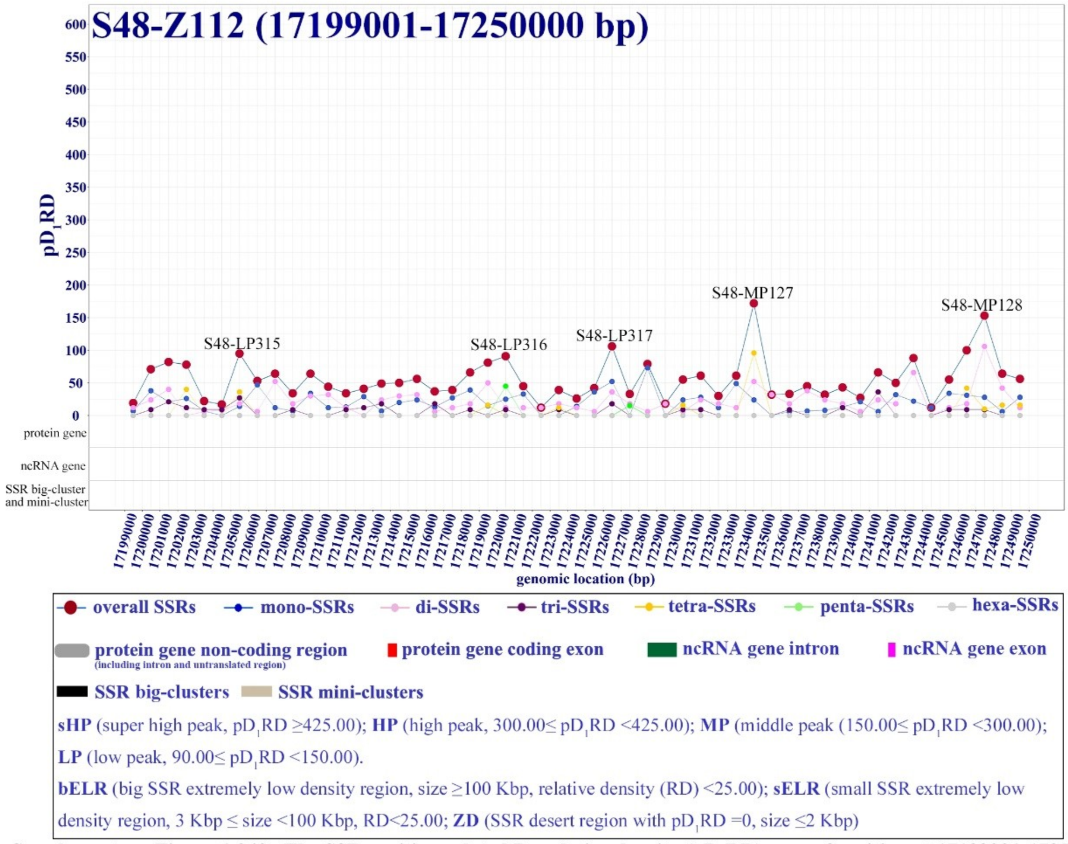

Supplementary Figure 1.340. The SSR position related D_1-relative density (pD_1RD) map of position at 17199001-17250000 bp of human reference Y-DNA (NC_000024.10) at resolution of 1 Kbp.

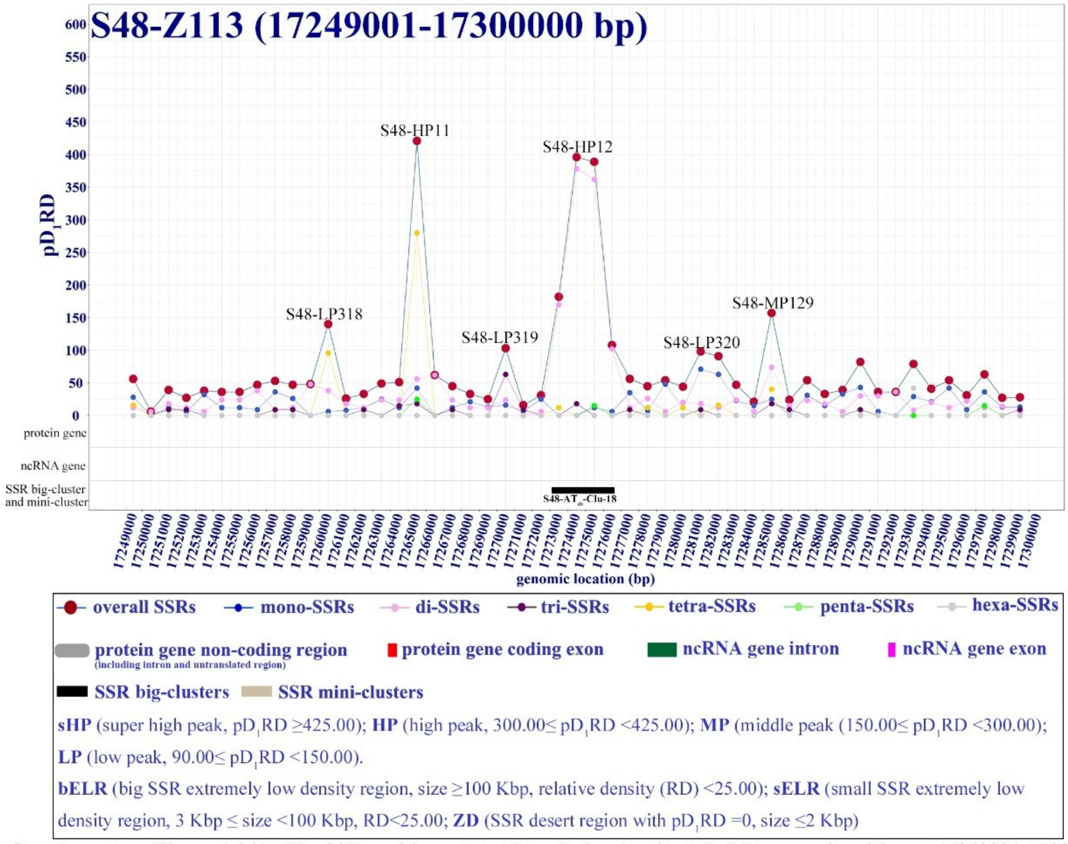

Supplementary Figure 1.341. The SSR position related D_1-relative density (pD_1RD) map of position at 17249001-17300000 bp of human reference Y-DNA (NC_000024.10) at resolution of 1 Kbp.

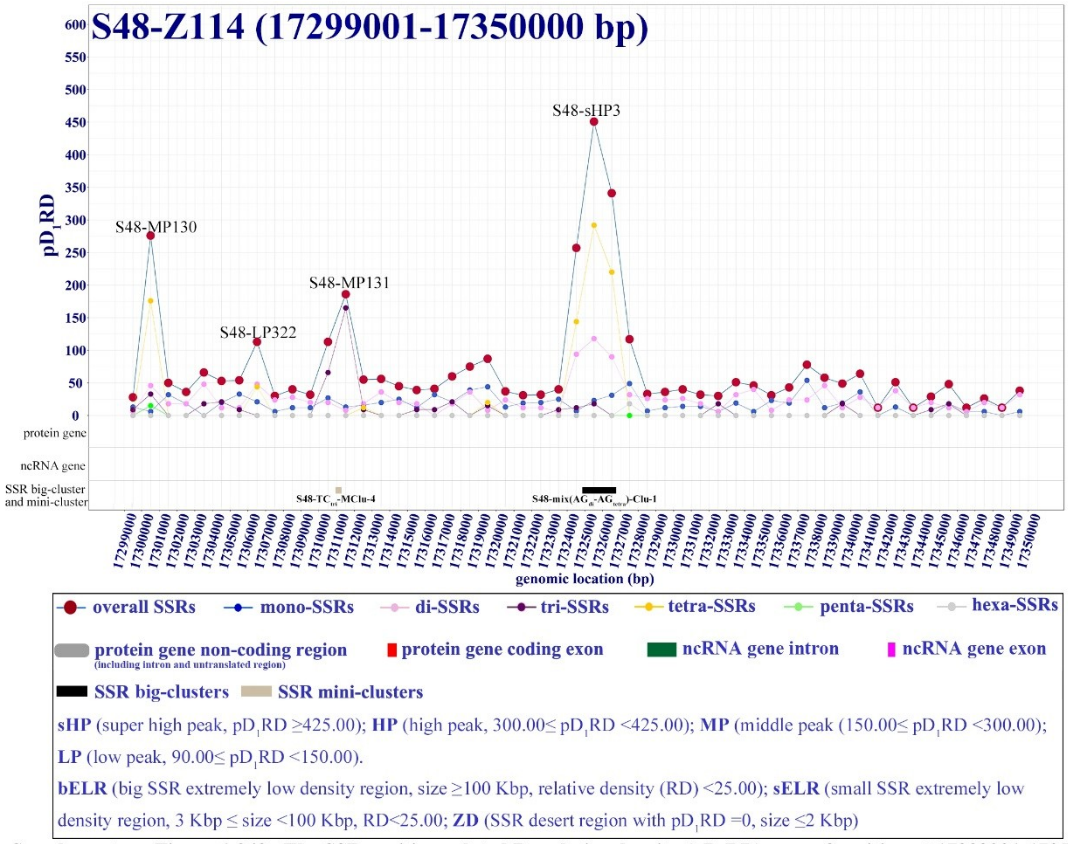

Supplementary Figure 1.342. The SSR position related D_1-relative density (pD_1RD) map of position at 17299001-17350000 bp of human reference Y-DNA (NC_000024.10) at resolution of 1 Kbp.

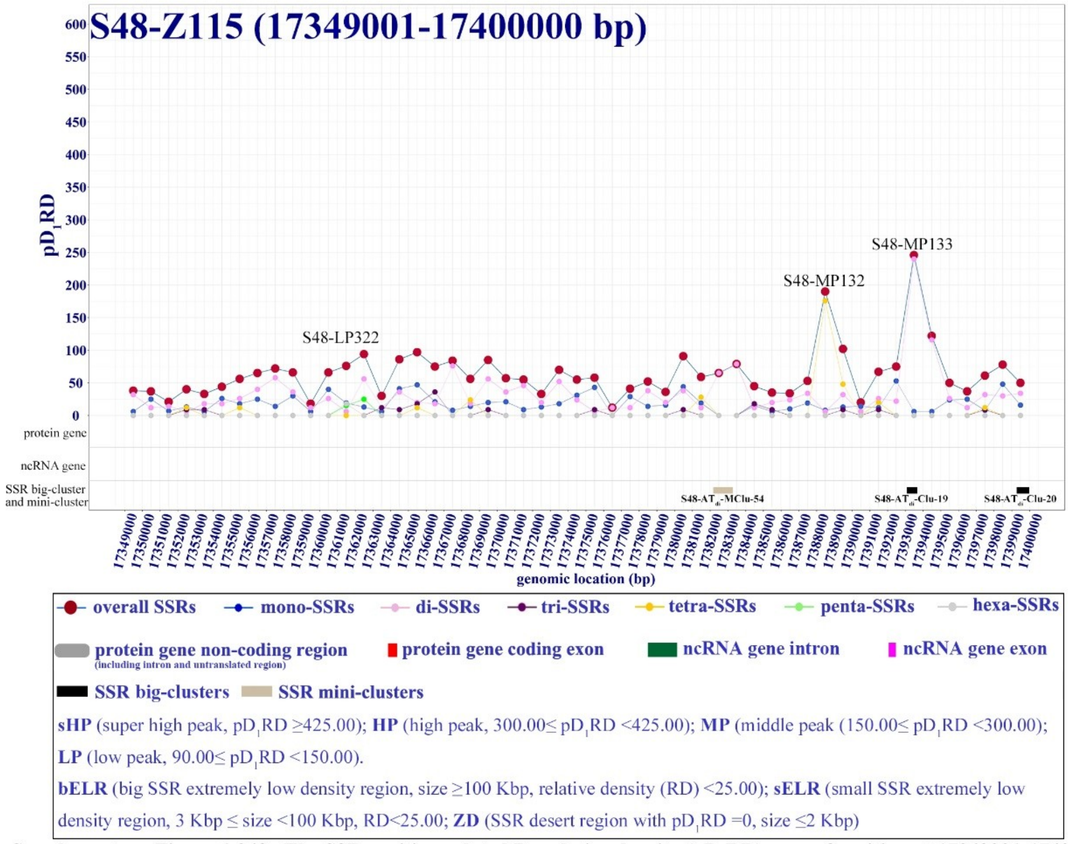

Supplementary Figure 1.343. The SSR position related D_1-relative density (pD_1RD) map of position at 17349001-17400000 bp of human reference Y-DNA (NC_000024.10) at resolution of 1 Kbp.

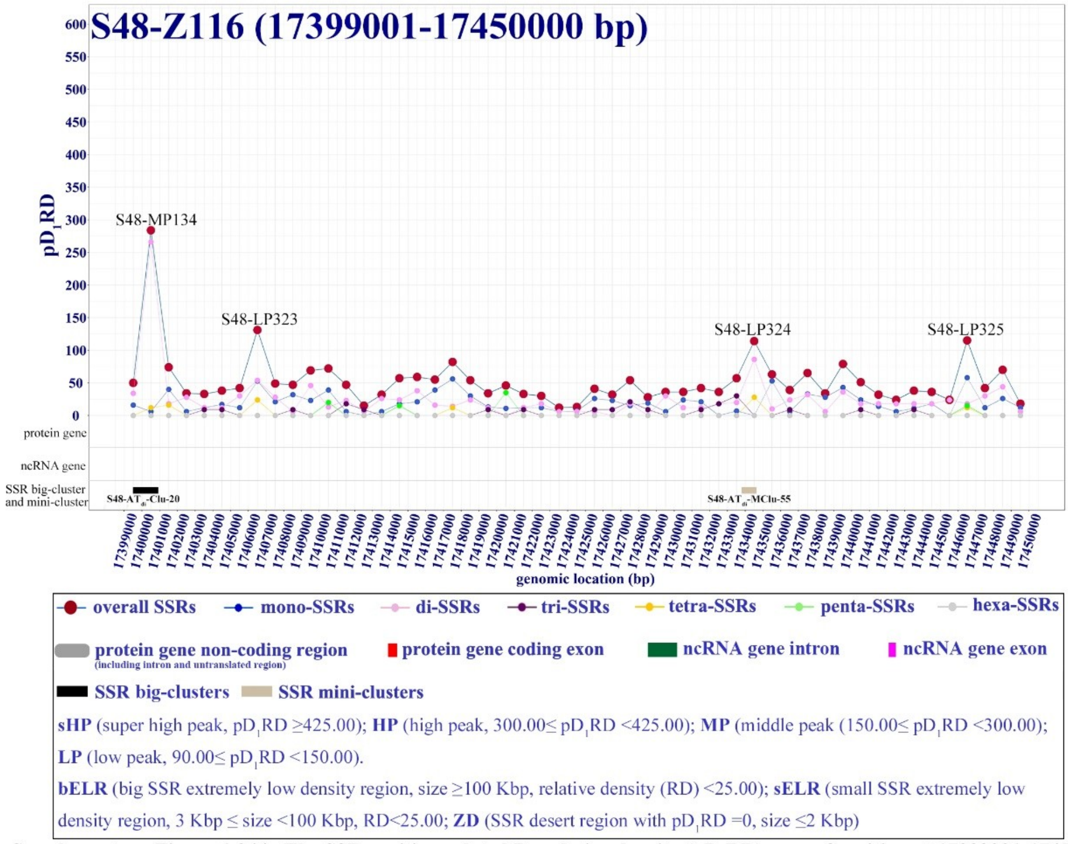

Supplementary Figure 1.344. The SSR position related D_1-relative density (pD_1RD) map of position at 17399001-17450000 bp of human reference Y-DNA (NC_000024.10) at resolution of 1 Kbp.

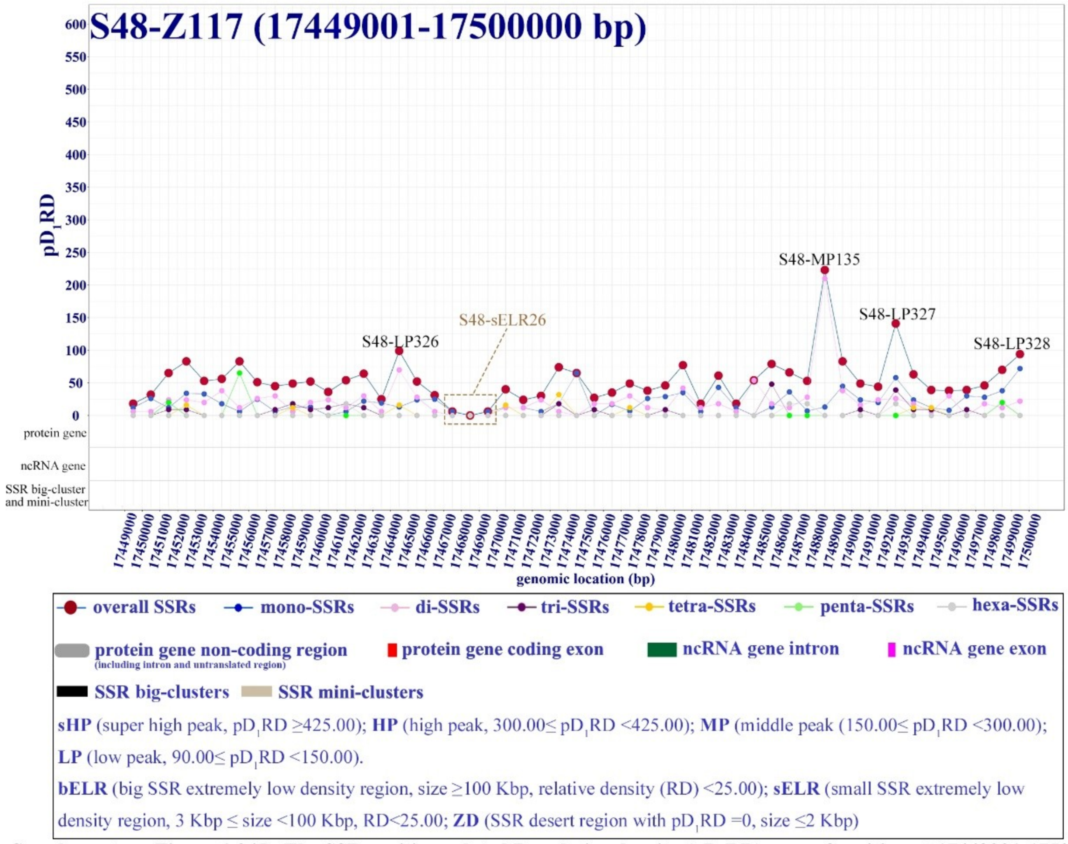

Supplementary Figure 1.345. The SSR position related D_1-relative density (pD_1RD) map of position at 17449001-17500000 bp of human reference Y-DNA (NC_000024.10) at resolution of 1 Kbp.

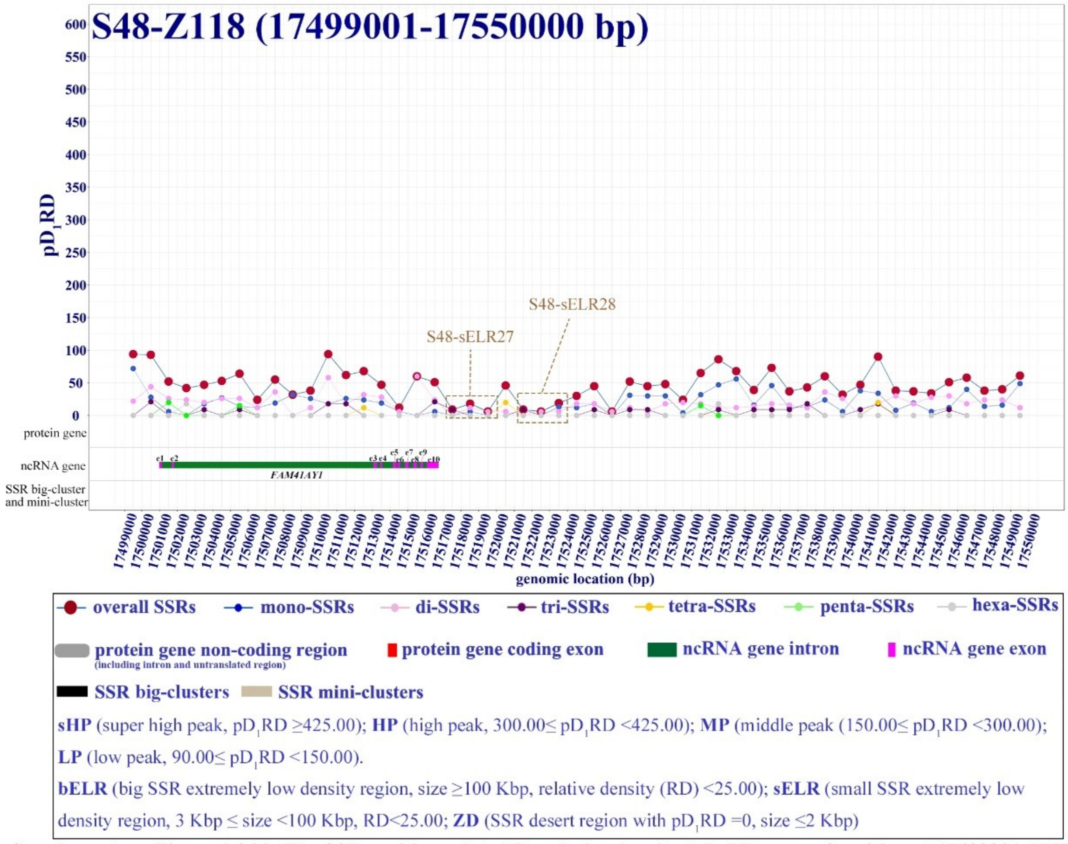

Supplementary Figure 1.346. The SSR position related D_1-relative density (pD_1RD) map of position at 17499001-17550000 bp of human reference Y-DNA (NC_000024.10) at resolution of 1 Kbp.

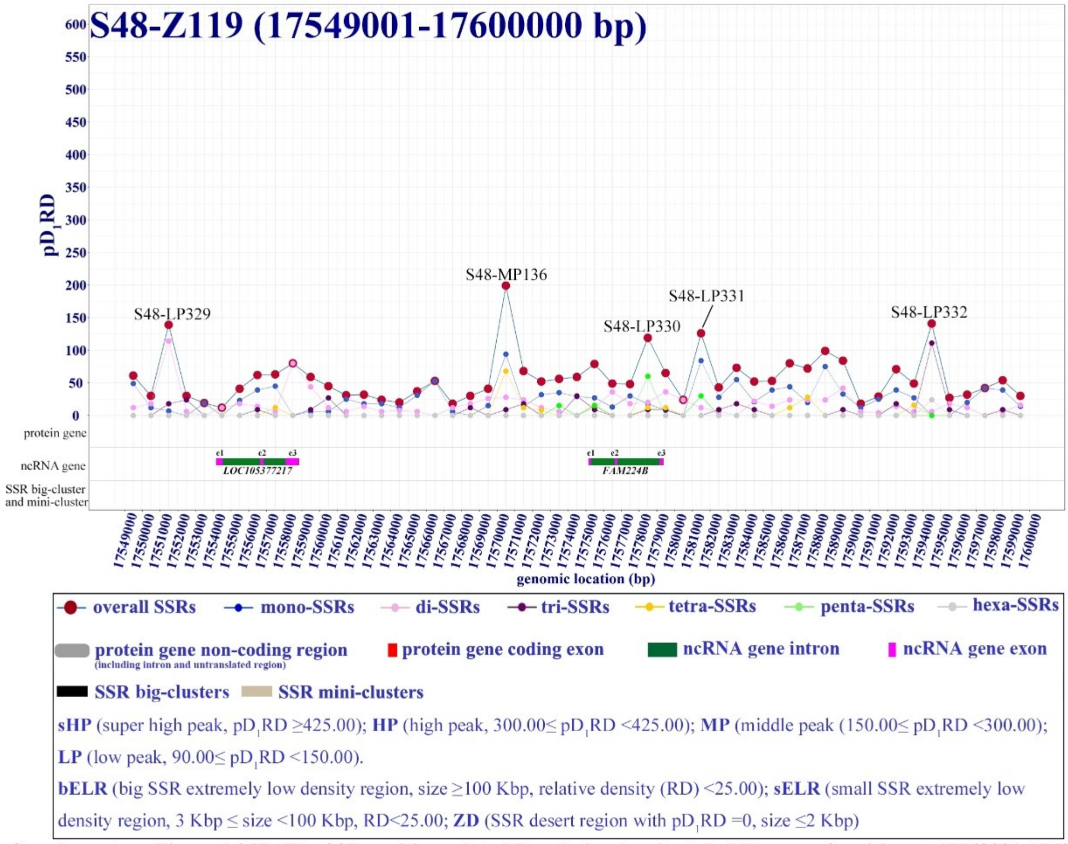

Supplementary Figure 1.347. The SSR position related D_1-relative density (pD_1RD) map of position at 17549001-17600000 bp of human reference Y-DNA (NC_000024.10) at resolution of 1 Kbp.

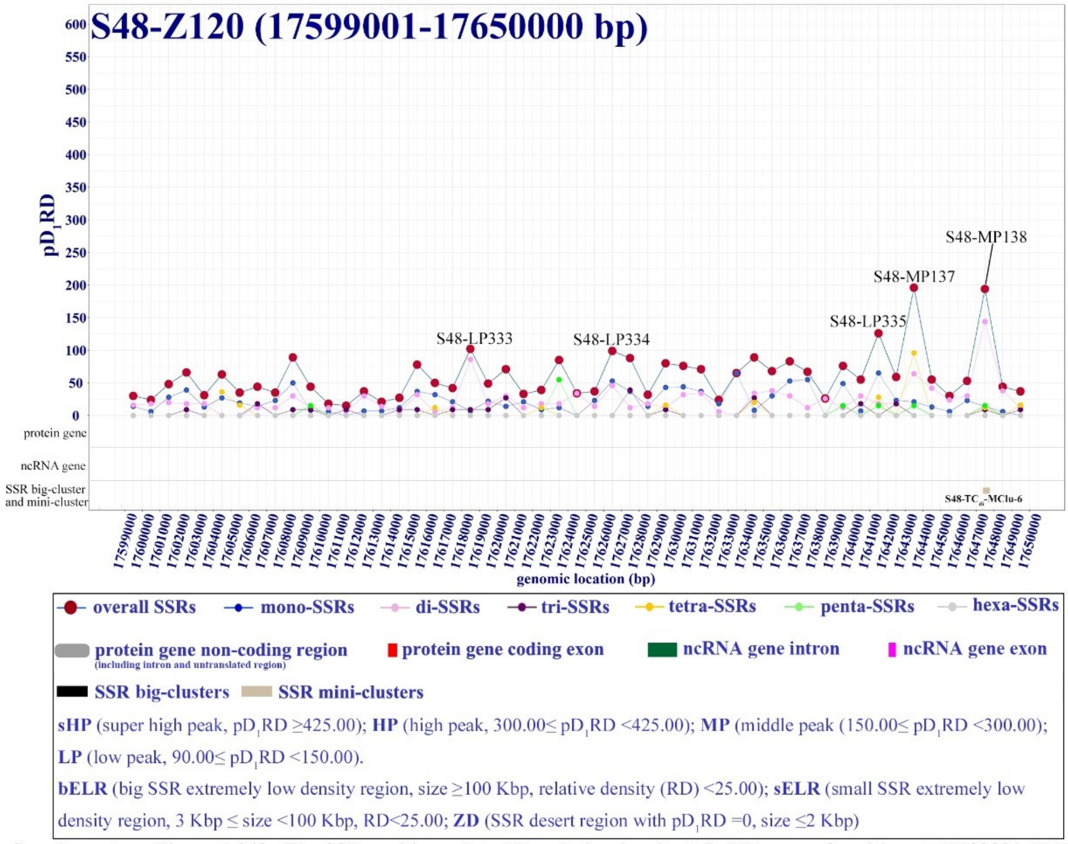

Supplementary Figure 1.348. The SSR position related D_1-relative density (pD_1RD) map of position at 17599001-17650000 bp of human reference Y-DNA (NC_000024.10) at resolution of 1 Kbp.

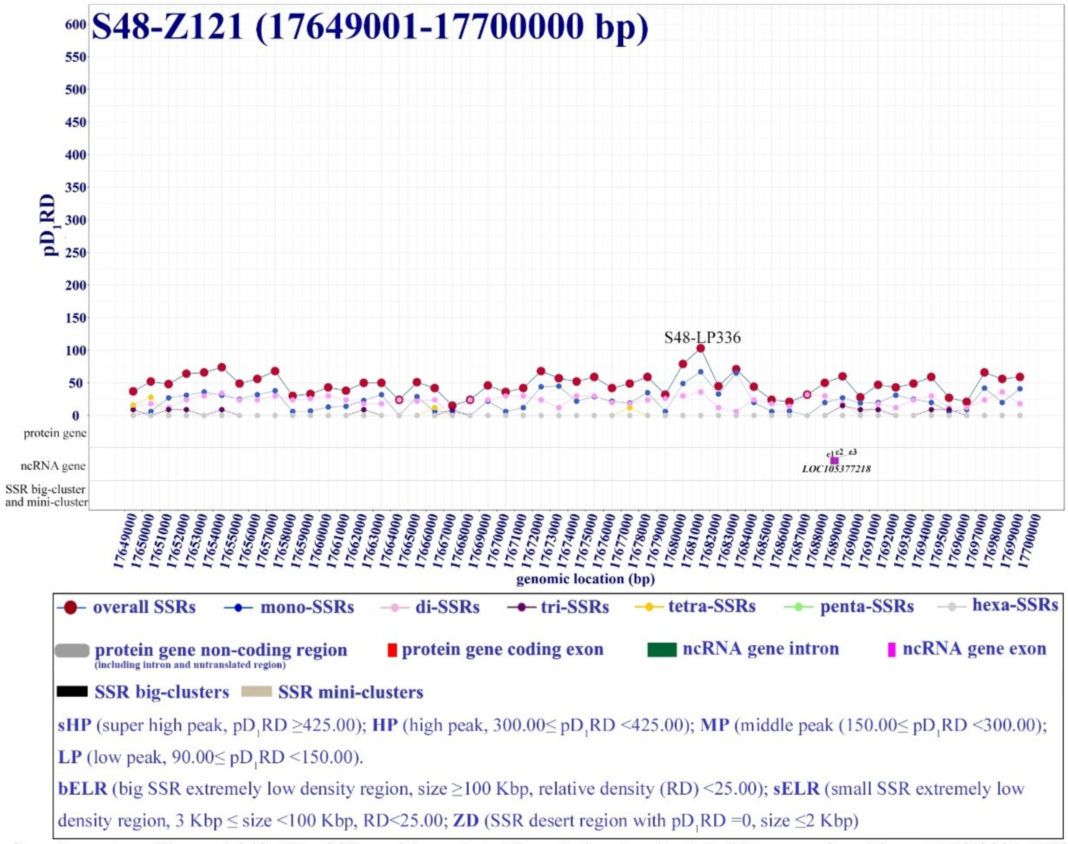

Supplementary Figure 1.349. The SSR position related D_1-relative density (pD_1RD) map of position at 17649001-17700000 bp of human reference Y-DNA (NC_000024.10) at resolution of 1 Kbp.

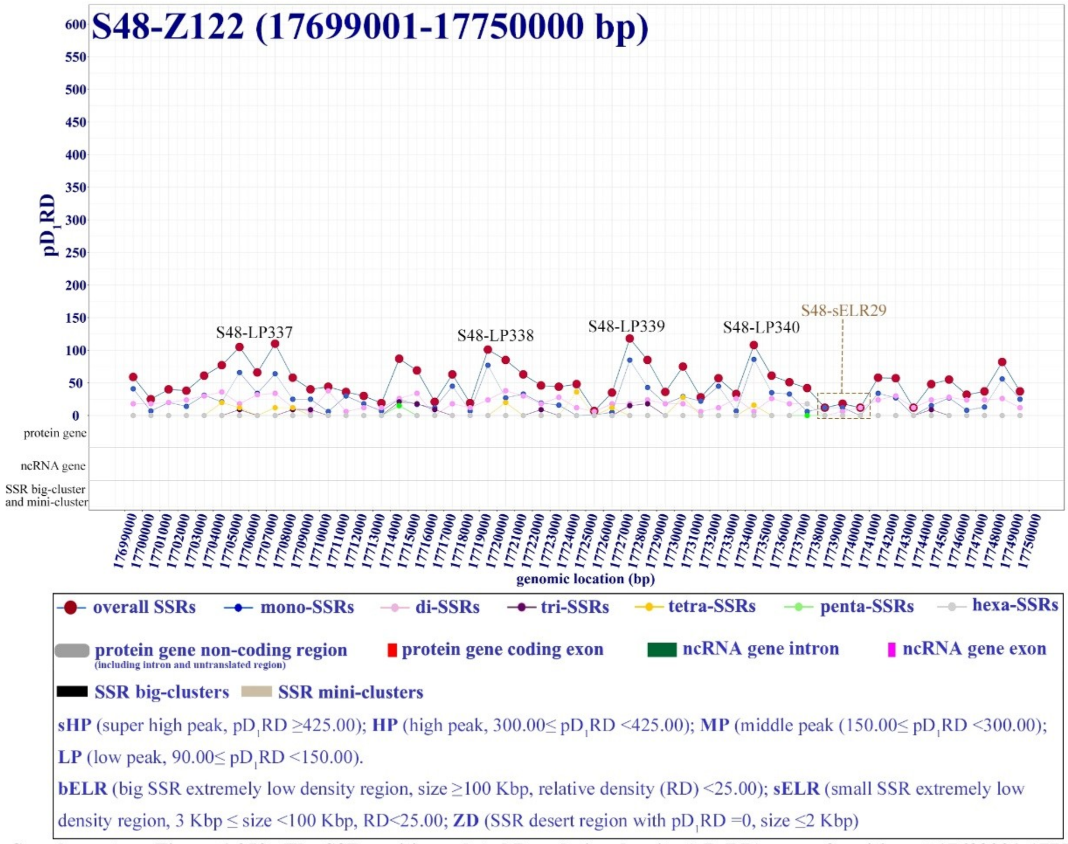

Supplementary Figure 1.350. The SSR position related D_1-relative density (pD_1RD) map of position at 17699001-17750000 bp of human reference Y-DNA (NC_000024.10) at resolution of 1 Kbp.

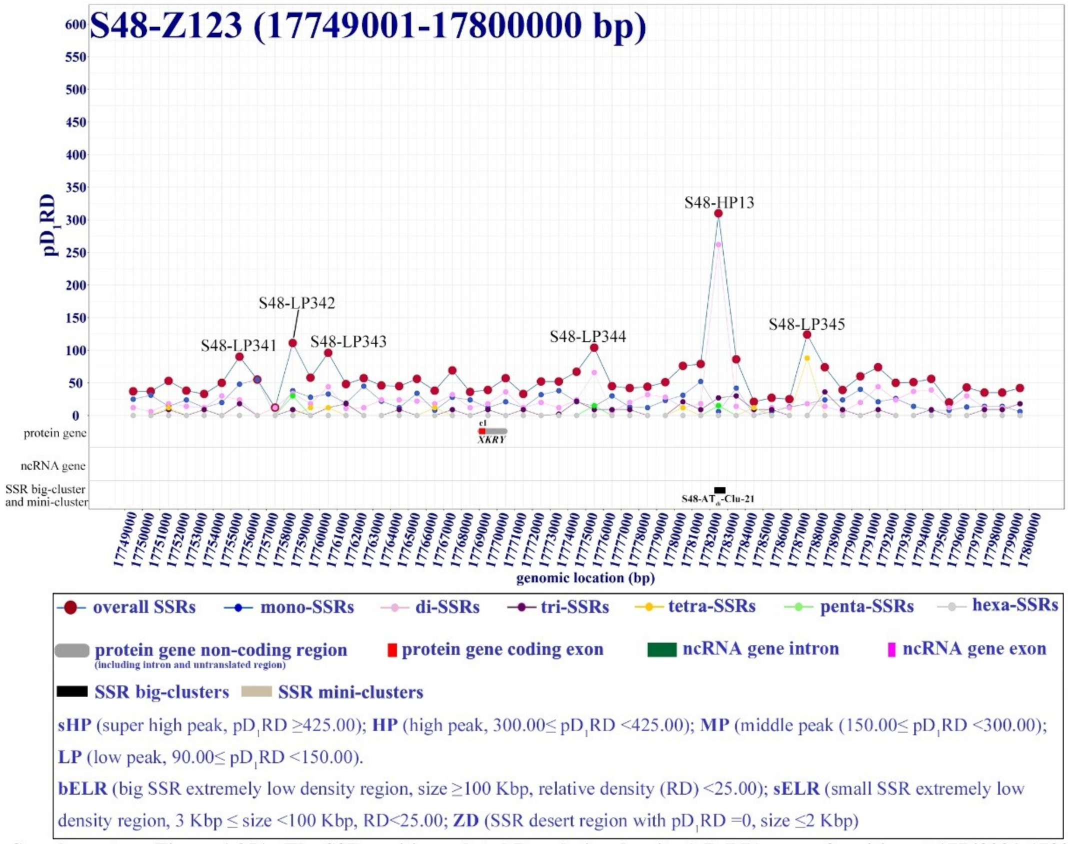

Supplementary Figure 1.351. The SSR position related D$_1$-relative density (pD$_1$RD) map of position at 17749001-17800000 bp of human reference Y-DNA (NC_000024.10) at resolution of 1 Kbp.

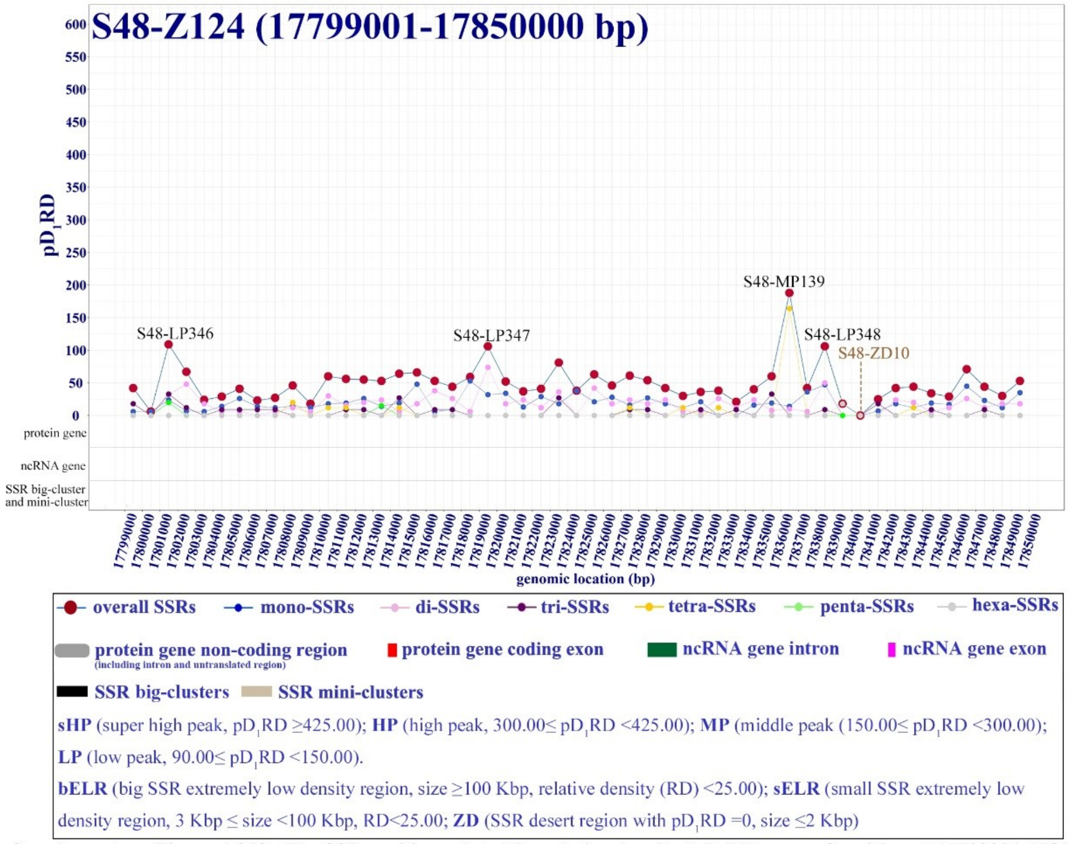

Supplementary Figure 1.352. The SSR position related D$_1$-relative density (pD$_1$RD) map of position at 17799001-17850000 bp of human reference Y-DNA (NC_000024.10) at resolution of 1 Kbp.

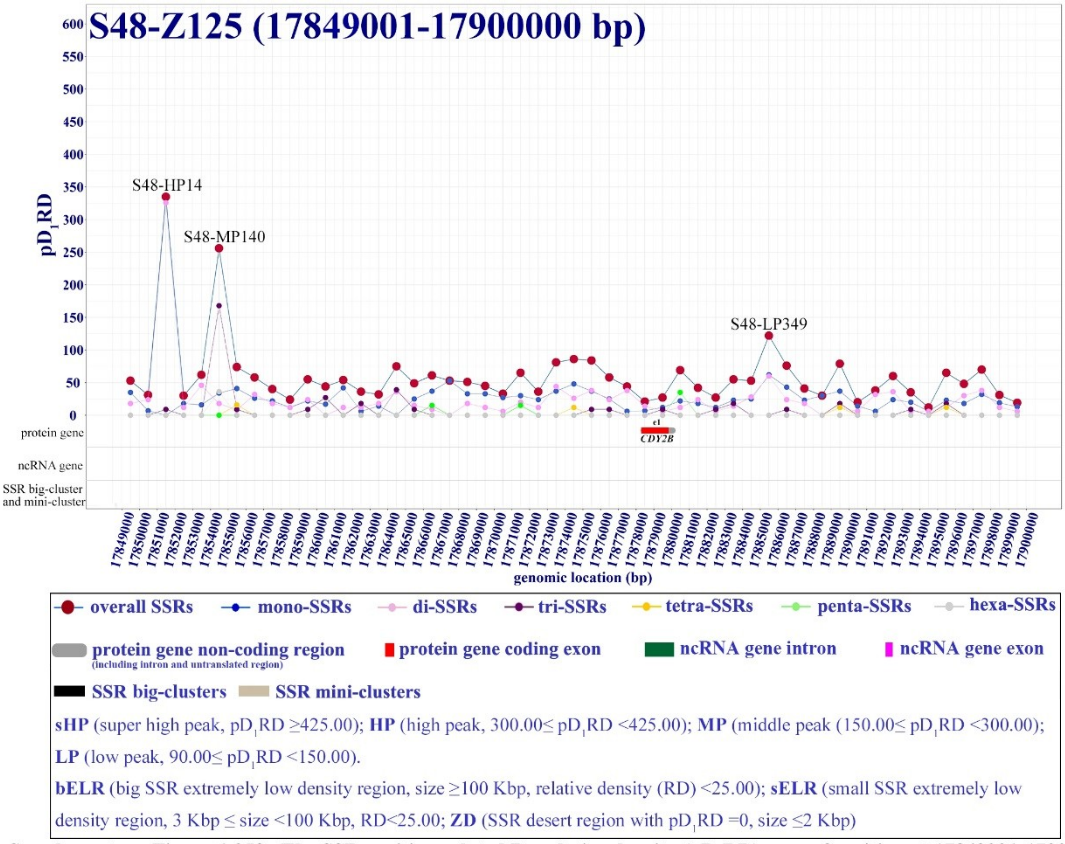

Supplementary Figure 1.353. The SSR position related D_1-relative density (pD_1RD) map of position at 17849001-17900000 bp of human reference Y-DNA (NC_000024.10) at resolution of 1 Kbp.

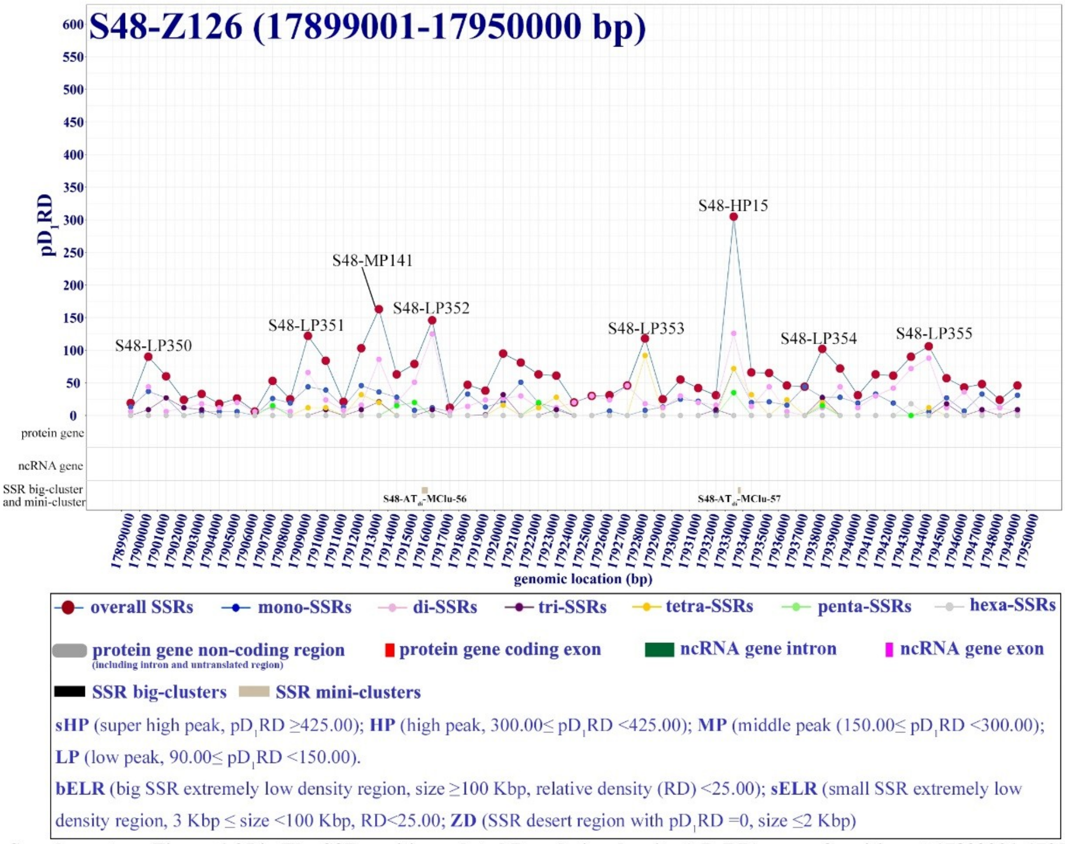

Supplementary Figure 1.354. The SSR position related D_1-relative density (pD_1RD) map of position at 17899001-17950000 bp of human reference Y-DNA (NC_000024.10) at resolution of 1 Kbp.

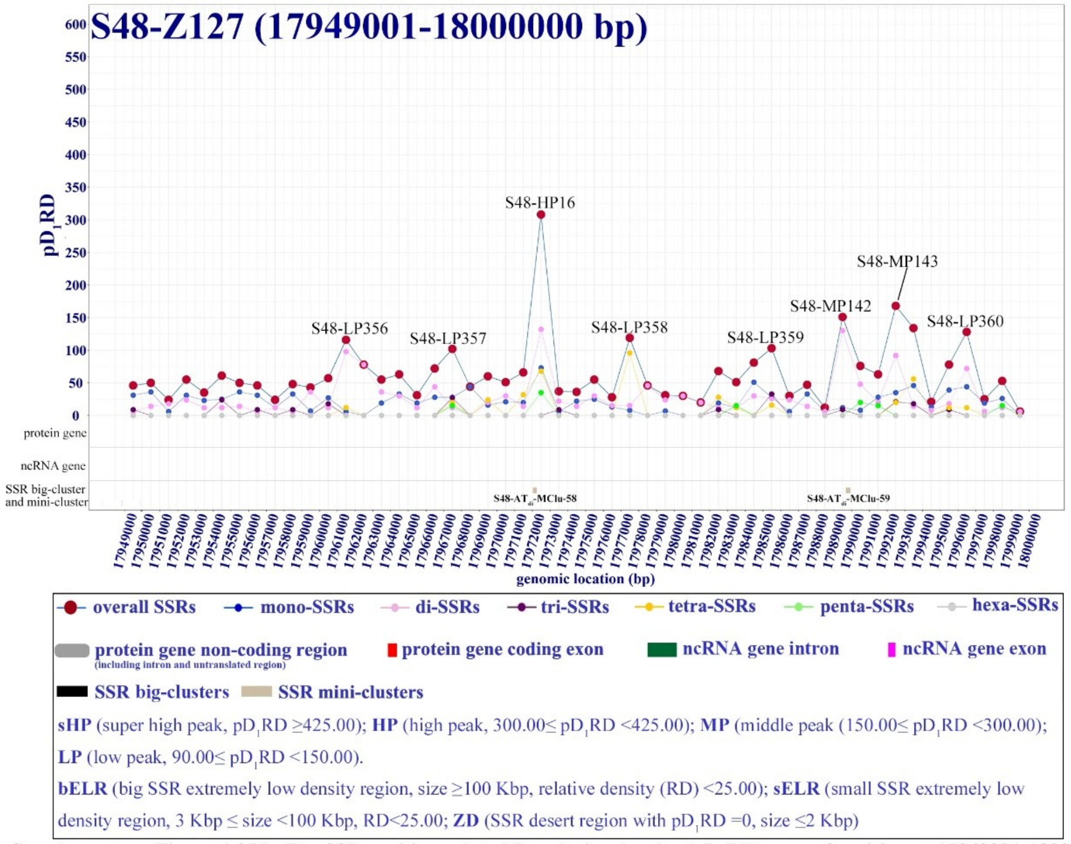

Supplementary Figure 1.355. The SSR position related D_1-relative density (pD_1RD) map of position at 17949001-18000000 bp of human reference Y-DNA (NC_000024.10) at resolution of 1 Kbp.

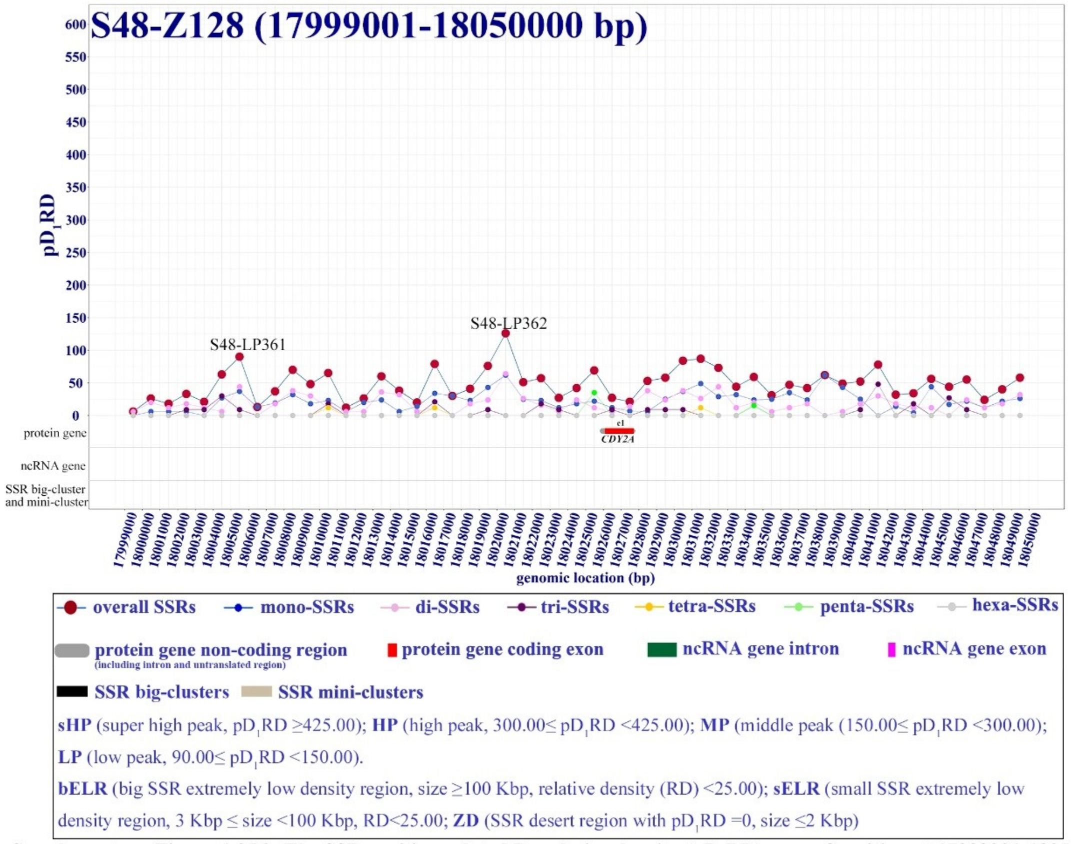

Supplementary Figure 1.356. The SSR position related D_1-relative density (pD_1RD) map of position at 17999001-18050000 bp of human reference Y-DNA (NC_000024.10) at resolution of 1 Kbp.

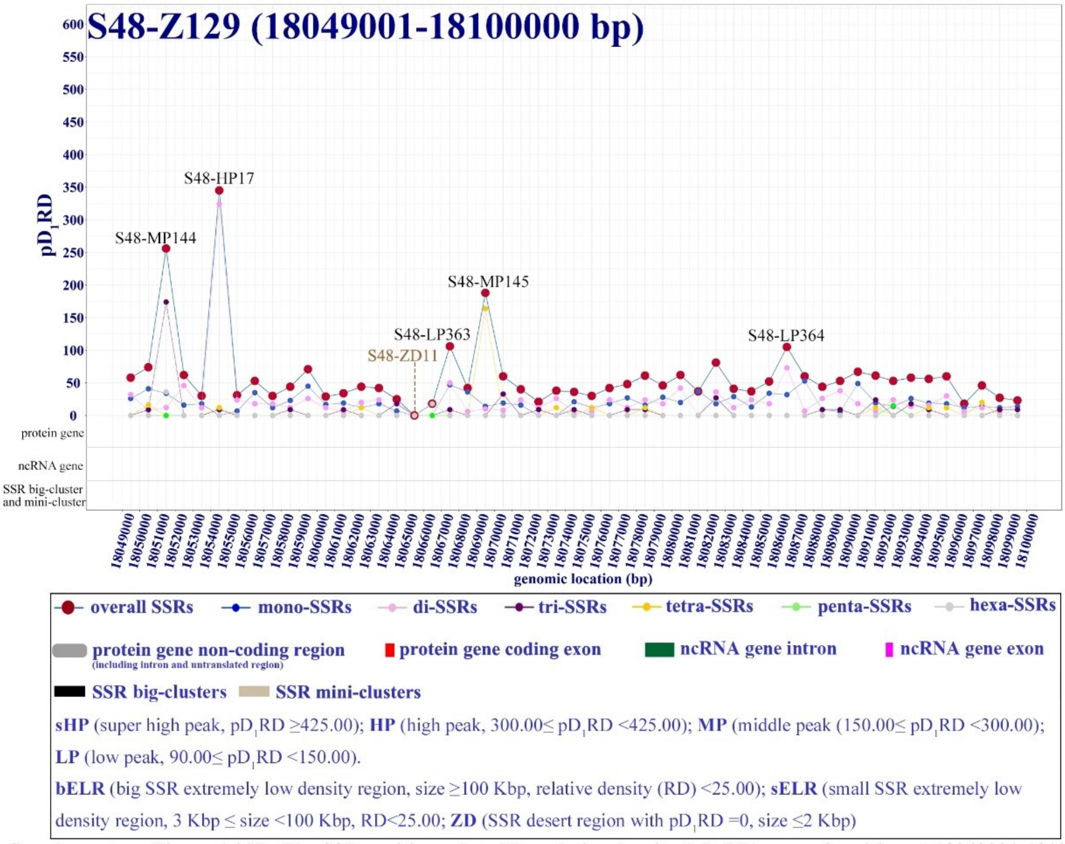

Supplementary Figure 1.357. The SSR position related D_1-relative density (pD_1RD) map of position at 18049001-18100000 bp of human reference Y-DNA (NC_000024.10) at resolution of 1 Kbp.

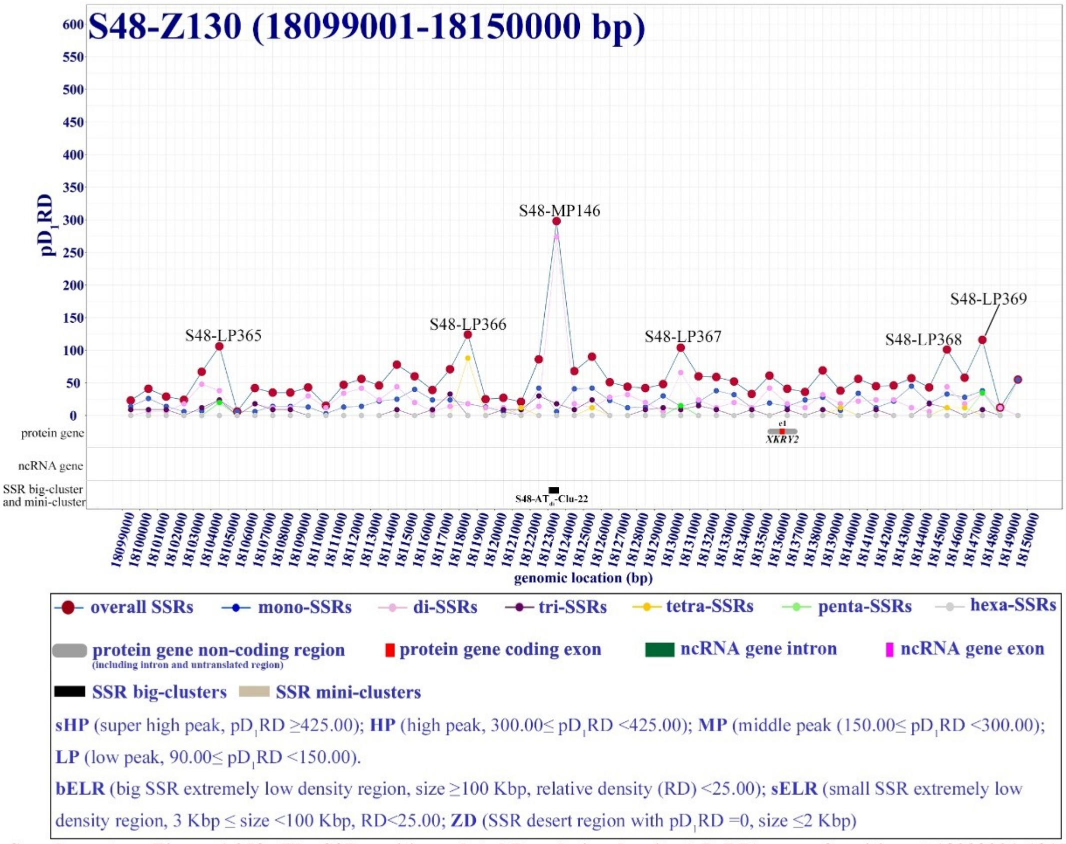

Supplementary Figure 1.358. The SSR position related D_1-relative density (pD_1RD) map of position at 18099001-18150000 bp of human reference Y-DNA (NC_000024.10) at resolution of 1 Kbp.

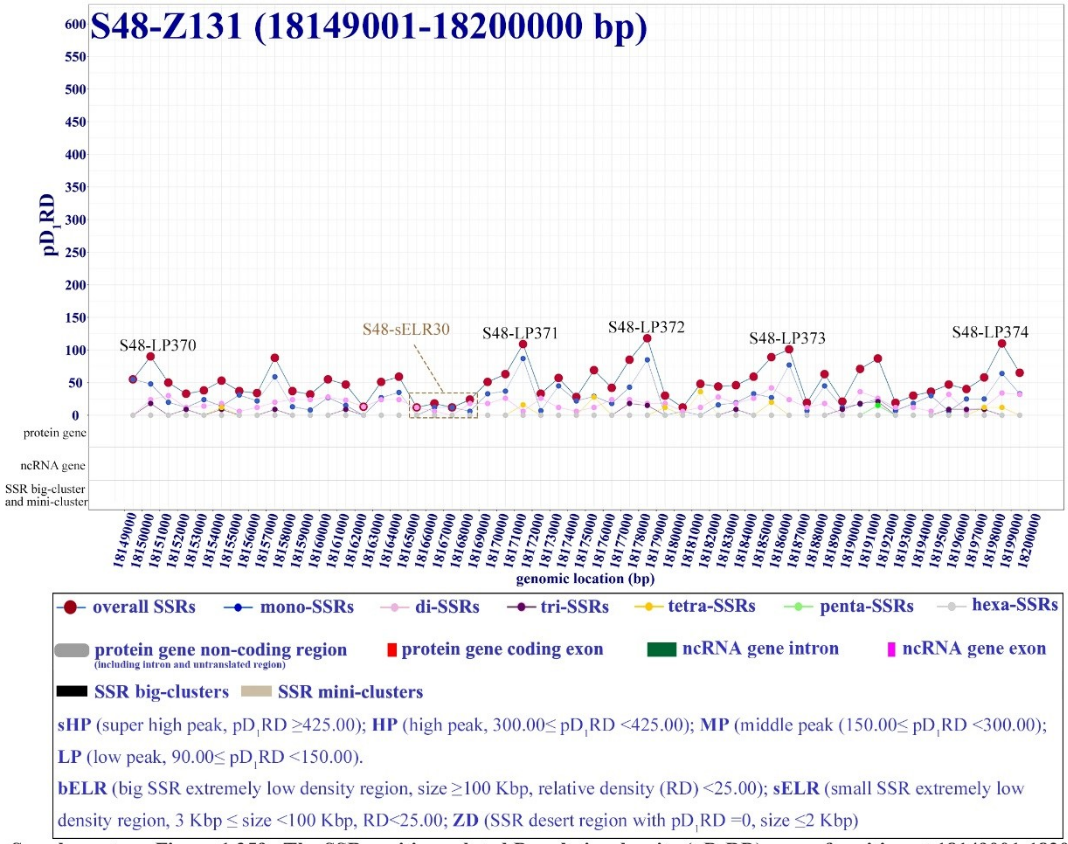

Supplementary Figure 1.359. The SSR position related D_1-relative density (pD_1RD) map of position at 18149001-18200000 bp of human reference Y-DNA (NC_000024.10) at resolution of 1 Kbp.

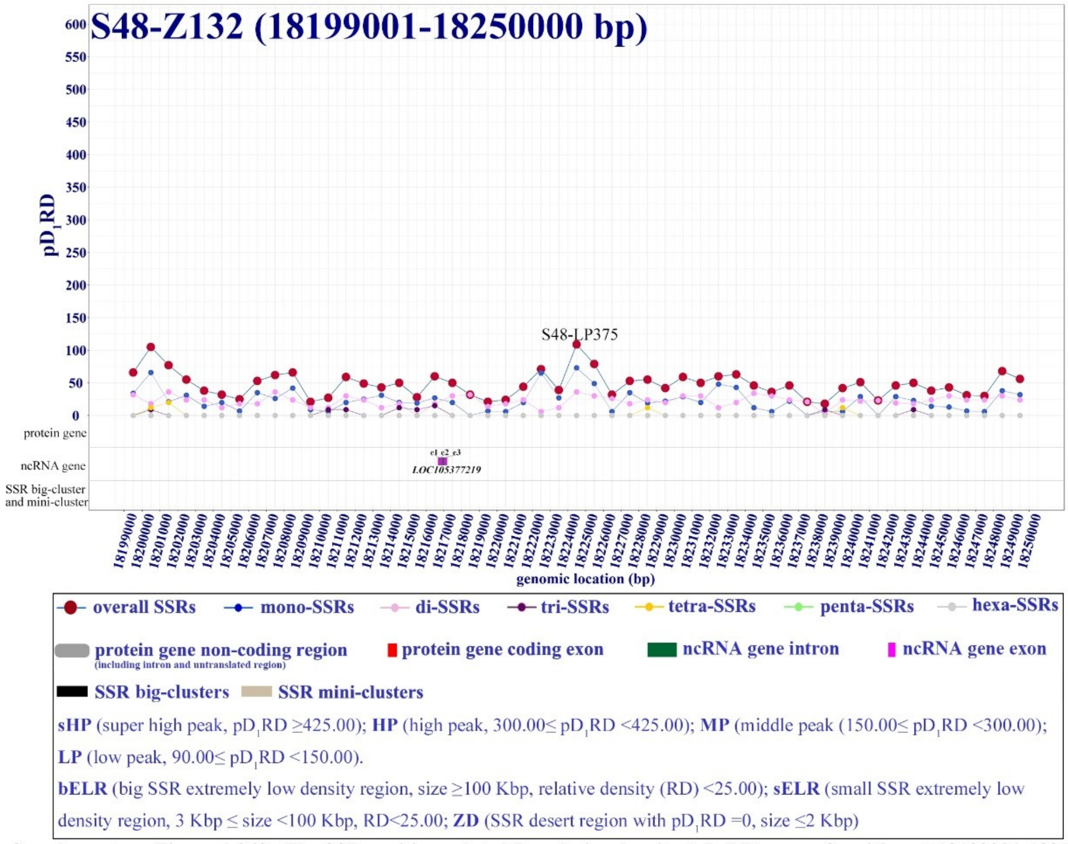

Supplementary Figure 1.360. The SSR position related D_1-relative density (pD_1RD) map of position at 18199001-18250000 bp of human reference Y-DNA (NC_000024.10) at resolution of 1 Kbp.

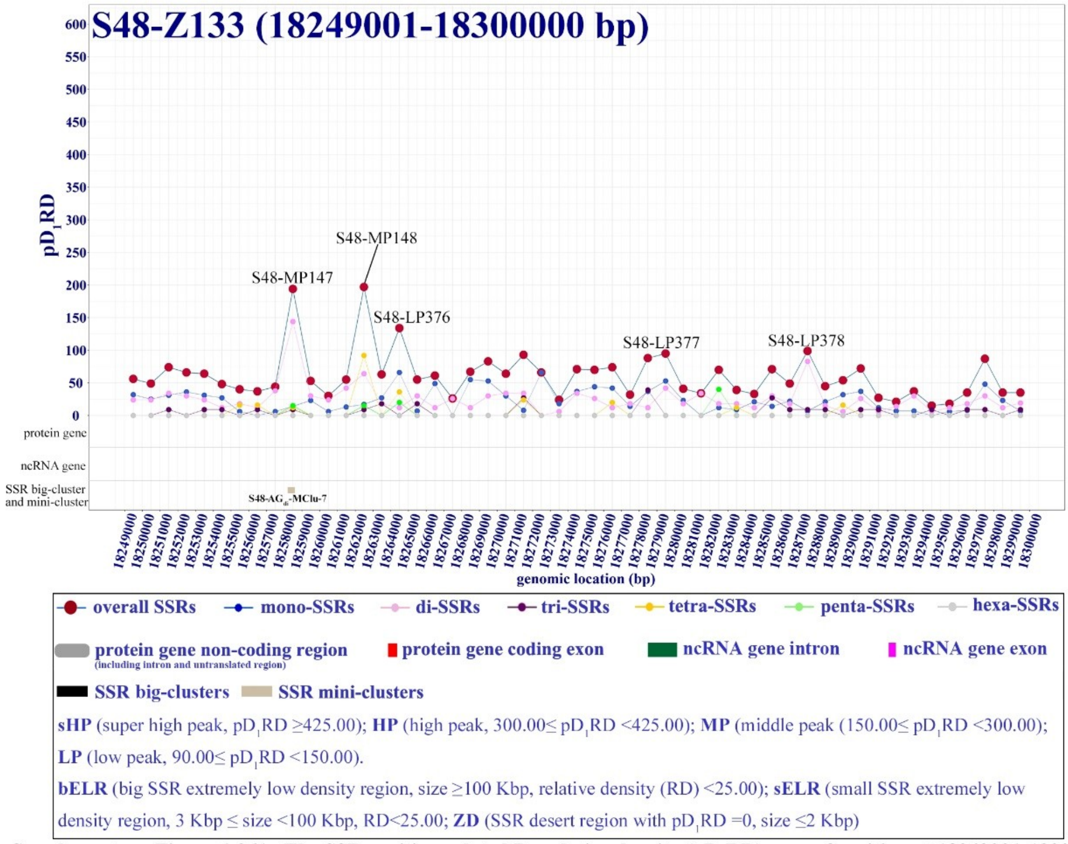

Supplementary Figure 1.361. The SSR position related D_1-relative density (pD_1RD) map of position at 18249001-18300000 bp of human reference Y-DNA (NC_000024.10) at resolution of 1 Kbp.

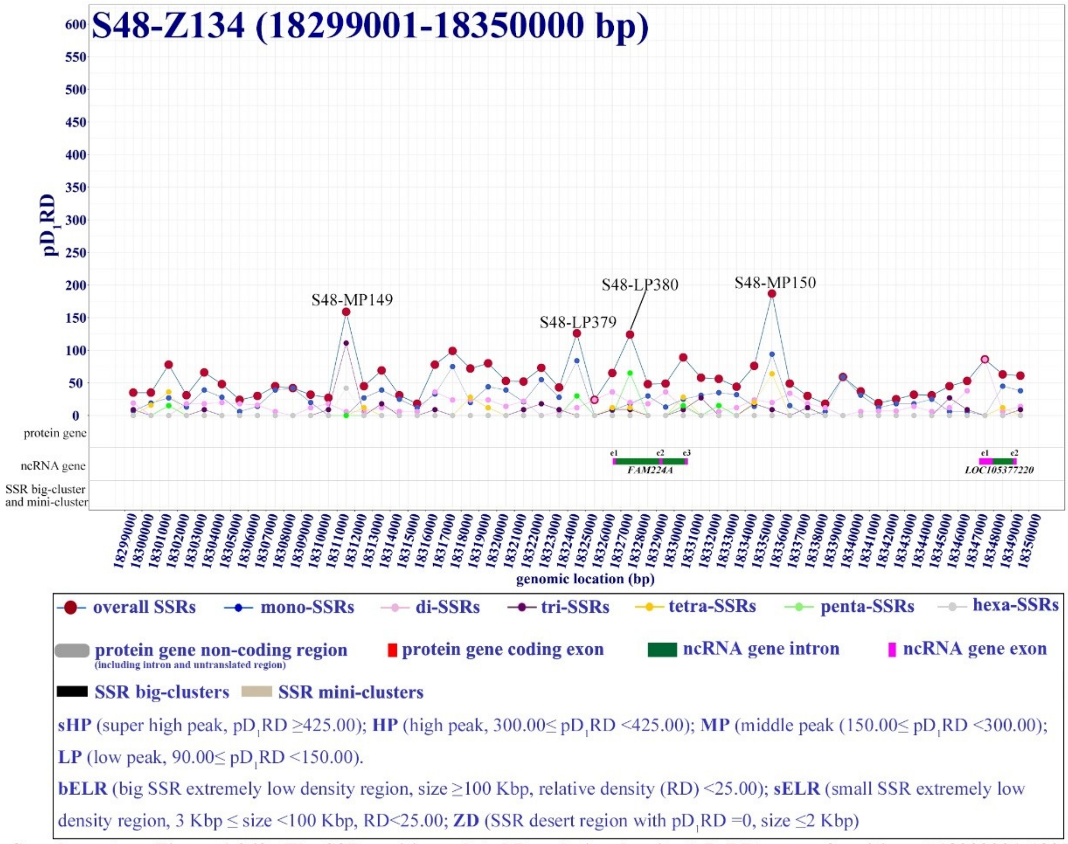

Supplementary Figure 1.362. The SSR position related D_1-relative density (pD_1RD) map of position at 18299001-18350000 bp of human reference Y-DNA (NC_000024.10) at resolution of 1 Kbp.

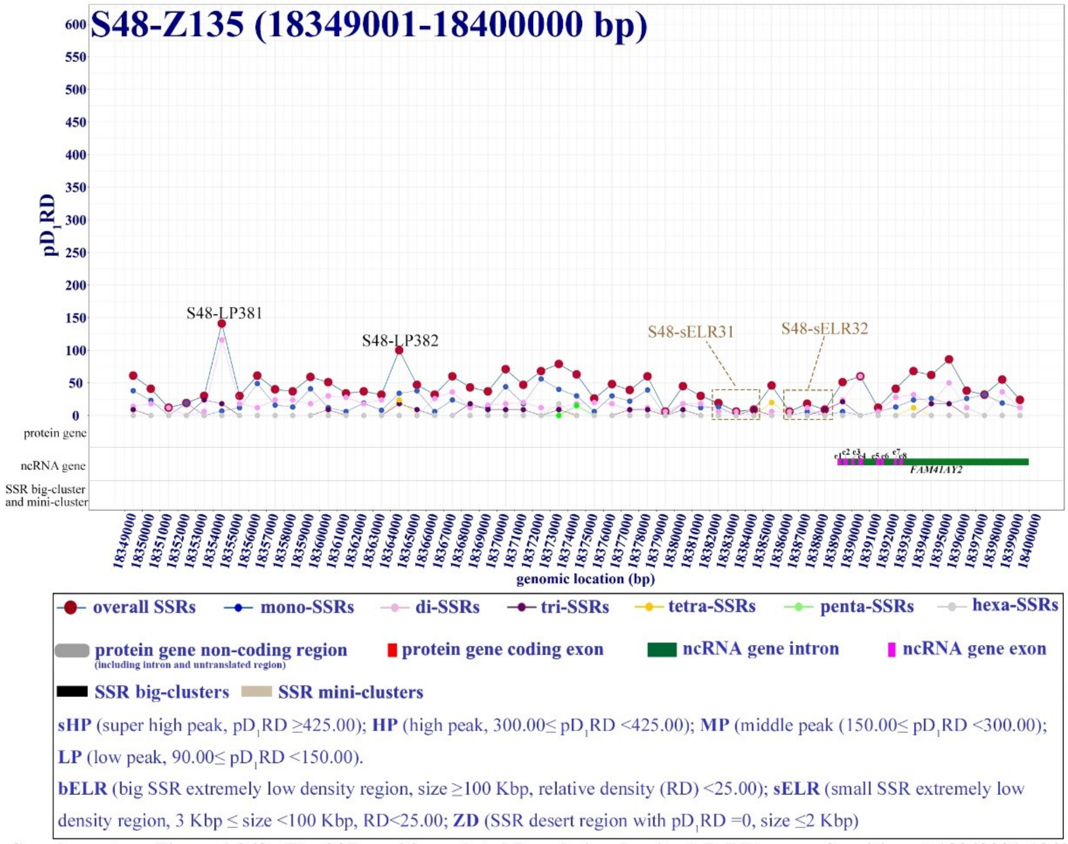

Supplementary Figure 1.363. The SSR position related D_1-relative density (pD_1RD) map of position at 18349001-18400000 bp of human reference Y-DNA (NC_000024.10) at resolution of 1 Kbp.

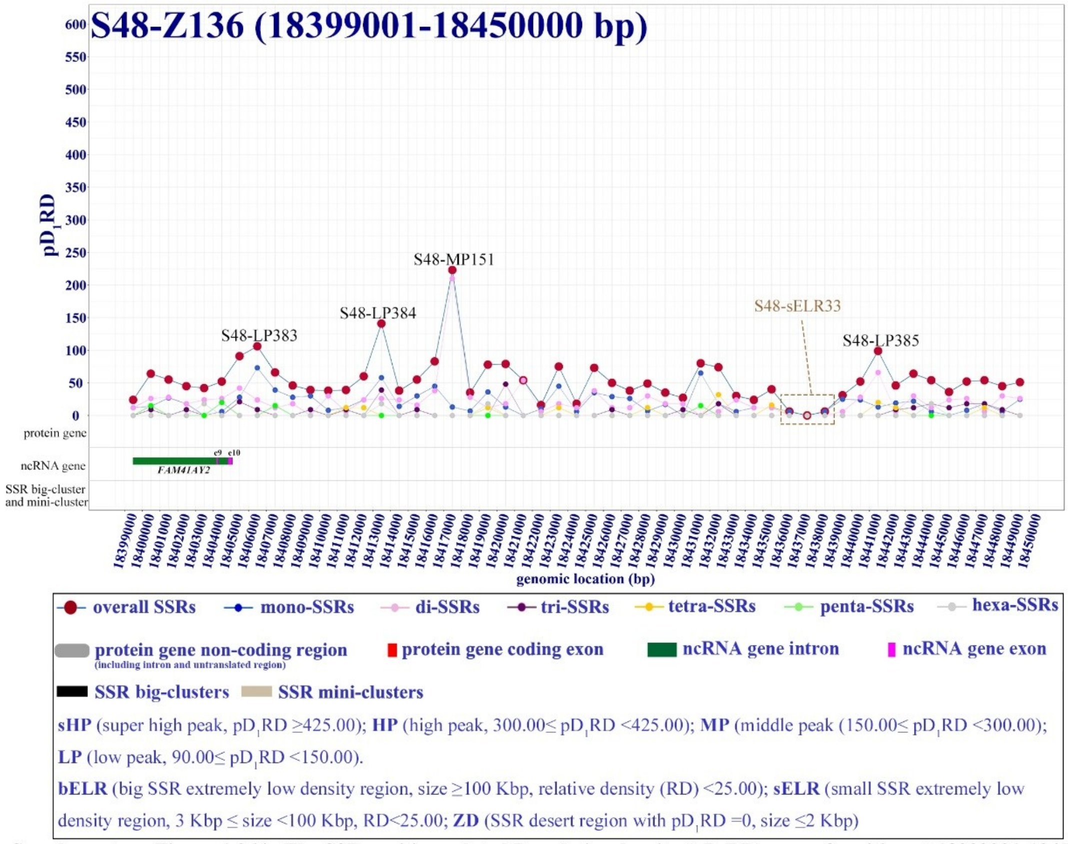

Supplementary Figure 1.364. The SSR position related D_1-relative density (pD_1RD) map of position at 18399001-18450000 bp of human reference Y-DNA (NC_000024.10) at resolution of 1 Kbp.

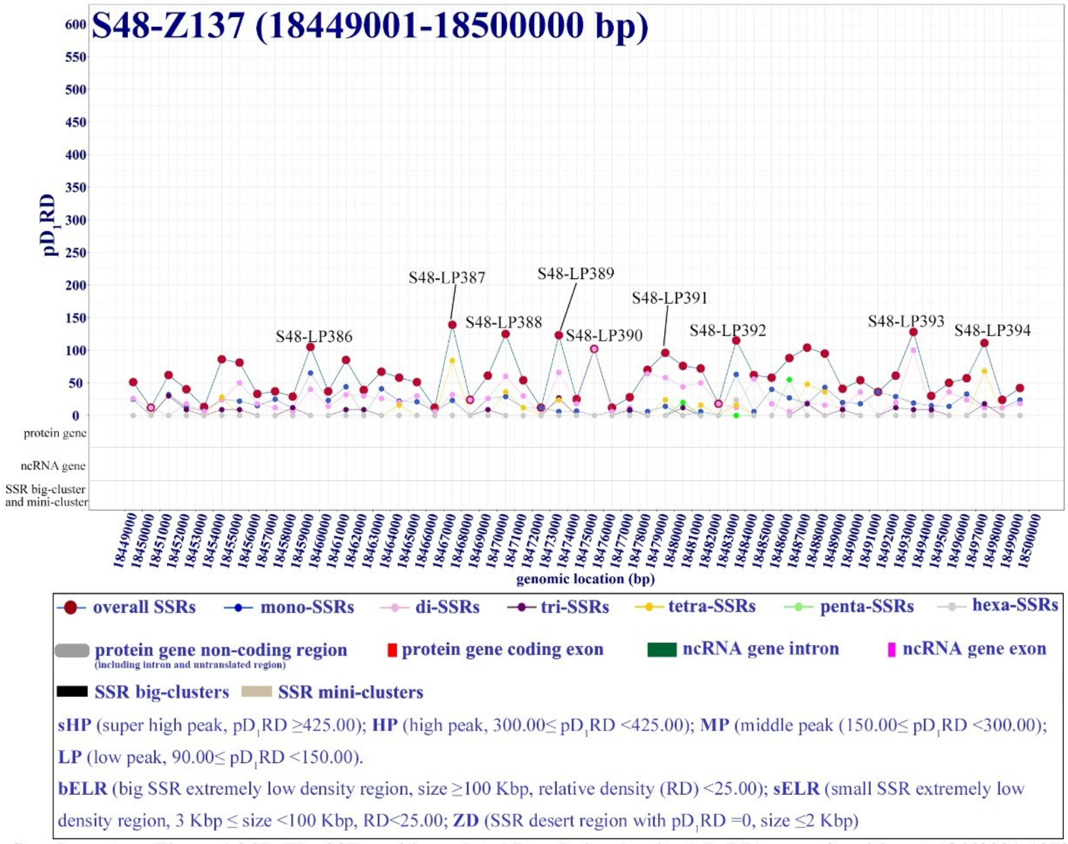

Supplementary Figure 1.365. The SSR position related D_1-relative density (pD_1RD) map of position at 18449001-18500000 bp of human reference Y-DNA (NC_000024.10) at resolution of 1 Kbp.

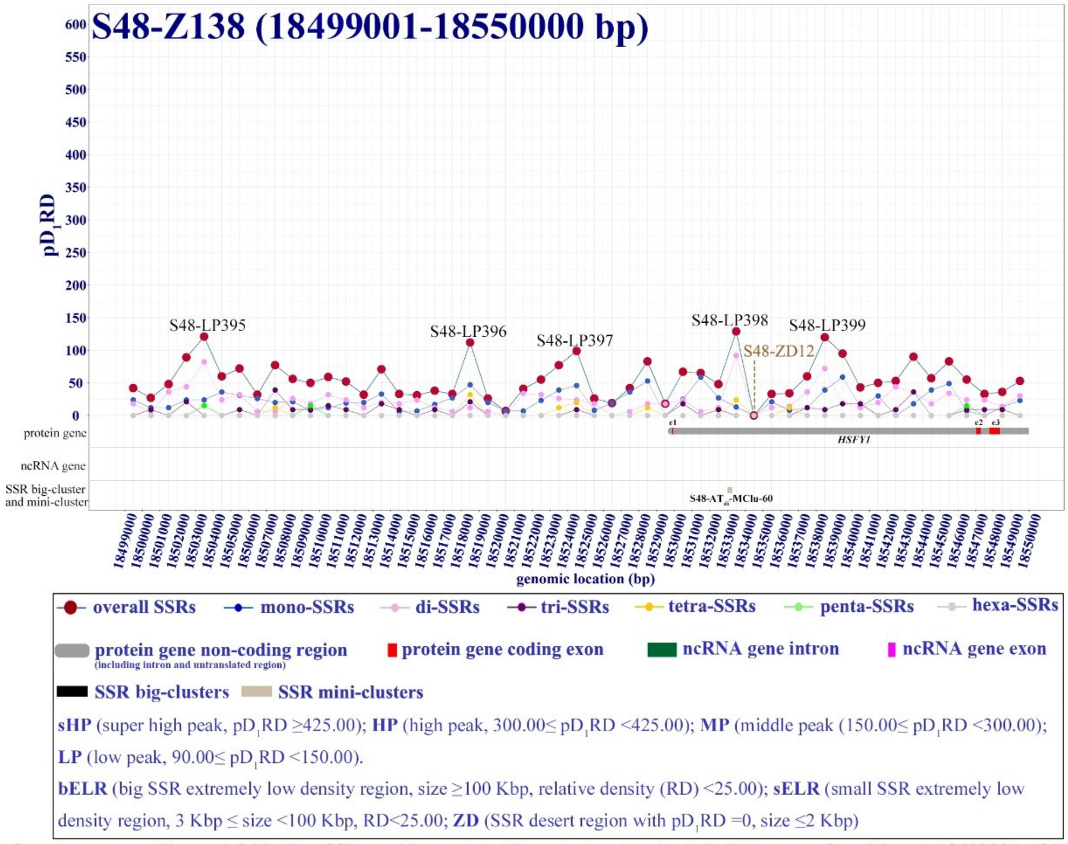

Supplementary Figure 1.366. The SSR position related D_1-relative density (pD_1RD) map of position at 18499001-18550000 bp of human reference Y-DNA (NC_000024.10) at resolution of 1 Kbp.

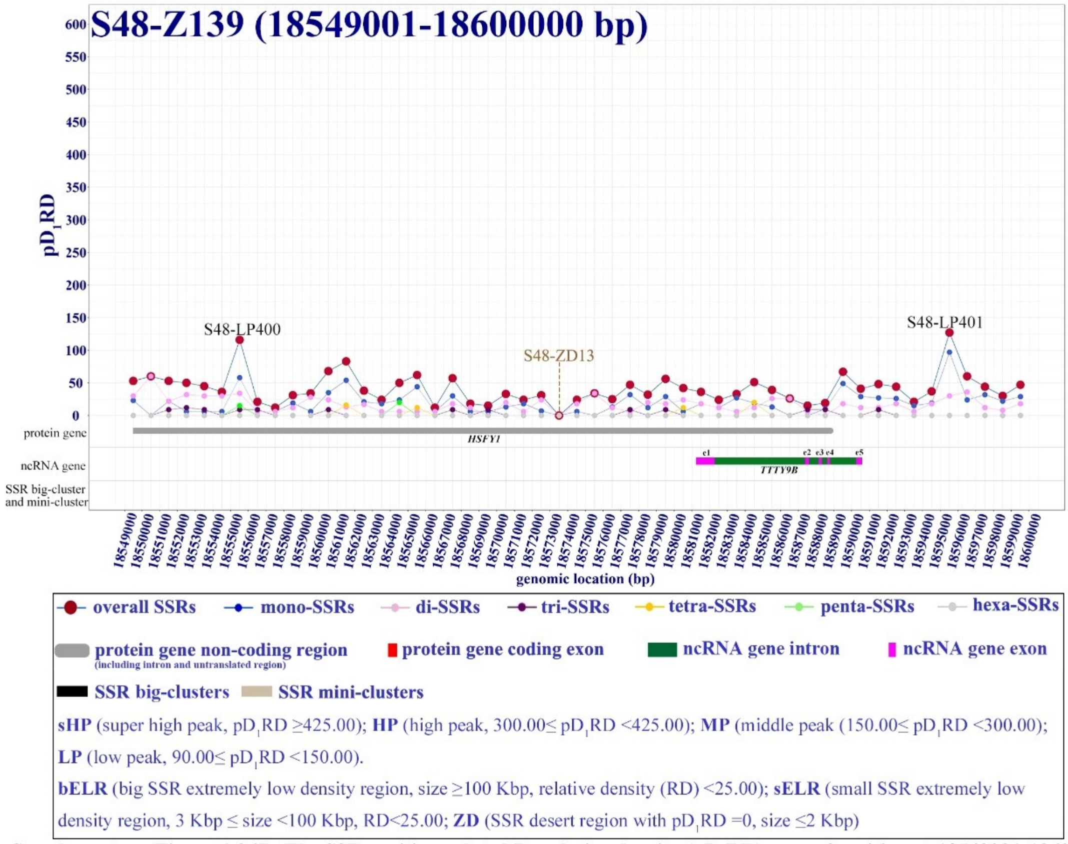

Supplementary Figure 1.367. The SSR position related D_1-relative density (pD_1RD) map of position at 18549001-18600000 bp of human reference Y-DNA (NC_000024.10) at resolution of 1 Kbp.

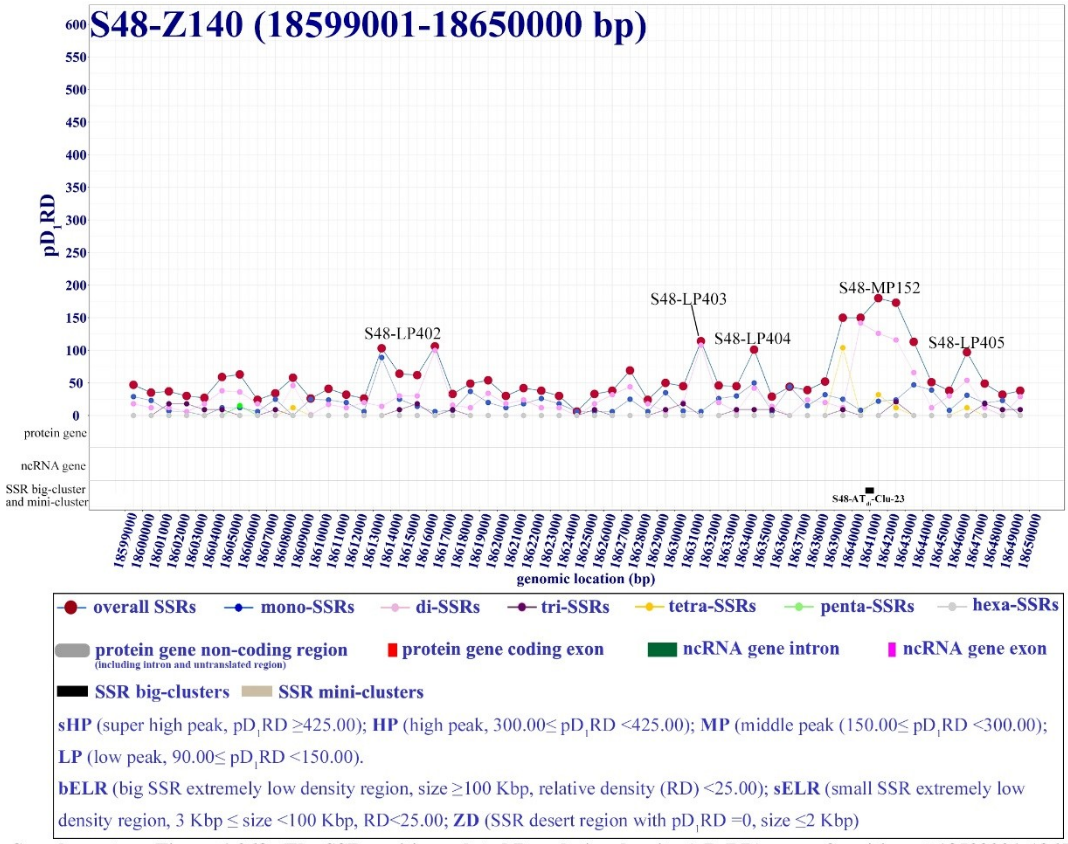

Supplementary Figure 1.368. The SSR position related D_1-relative density (pD_1RD) map of position at 18599001-18650000 bp of human reference Y-DNA (NC_000024.10) at resolution of 1 Kbp.

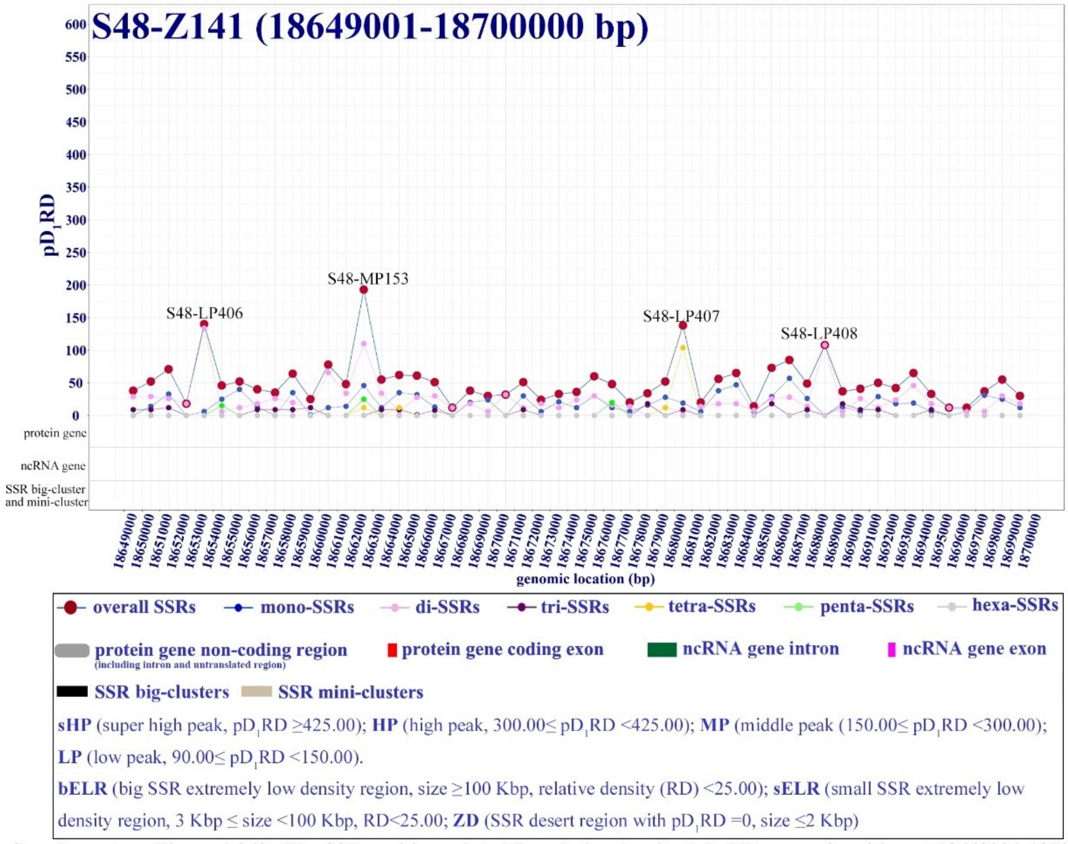

Supplementary Figure 1.369. The SSR position related D_1-relative density (pD_1RD) map of position at 18649001-18700000 bp of human reference Y-DNA (NC_000024.10) at resolution of 1 Kbp.

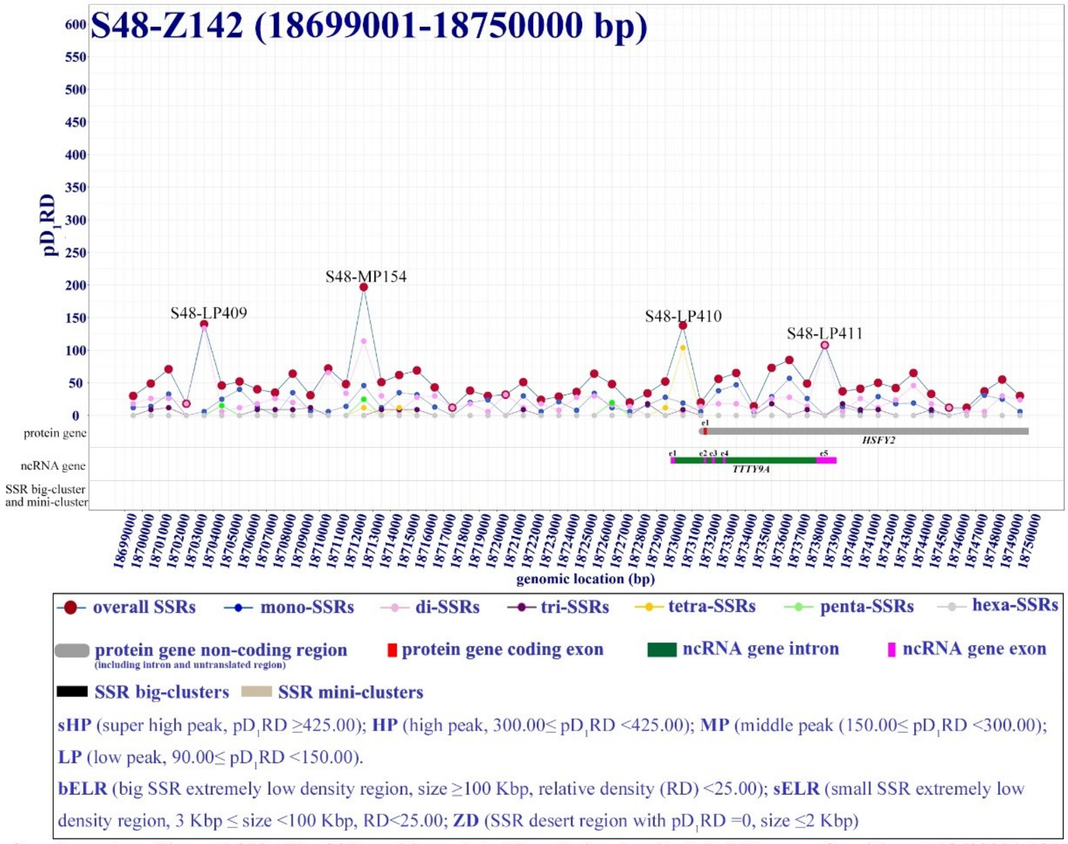

Supplementary Figure 1.370. The SSR position related D_1-relative density (pD_1RD) map of position at 18699001-18750000 bp of human reference Y-DNA (NC_000024.10) at resolution of 1 Kbp.

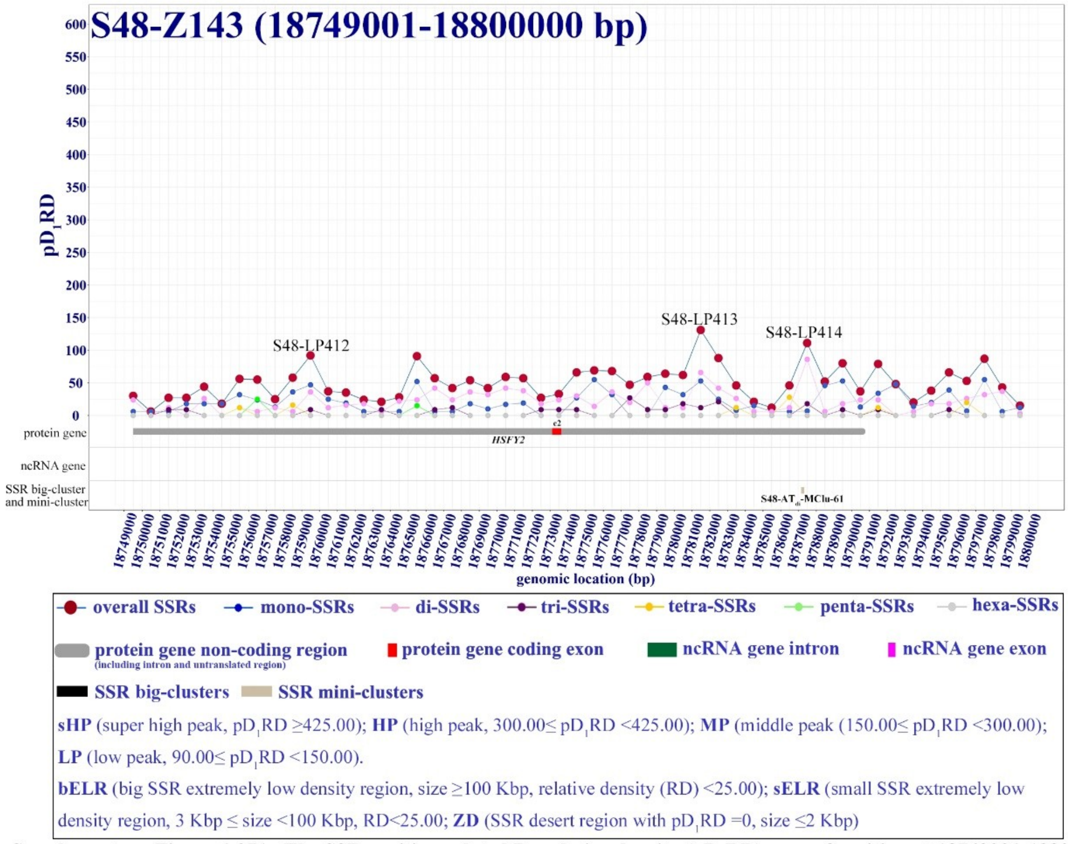

Supplementary Figure 1.371. The SSR position related D_1-relative density (pD_1RD) map of position at 18749001-18800000 bp of human reference Y-DNA (NC_000024.10) at resolution of 1 Kbp.

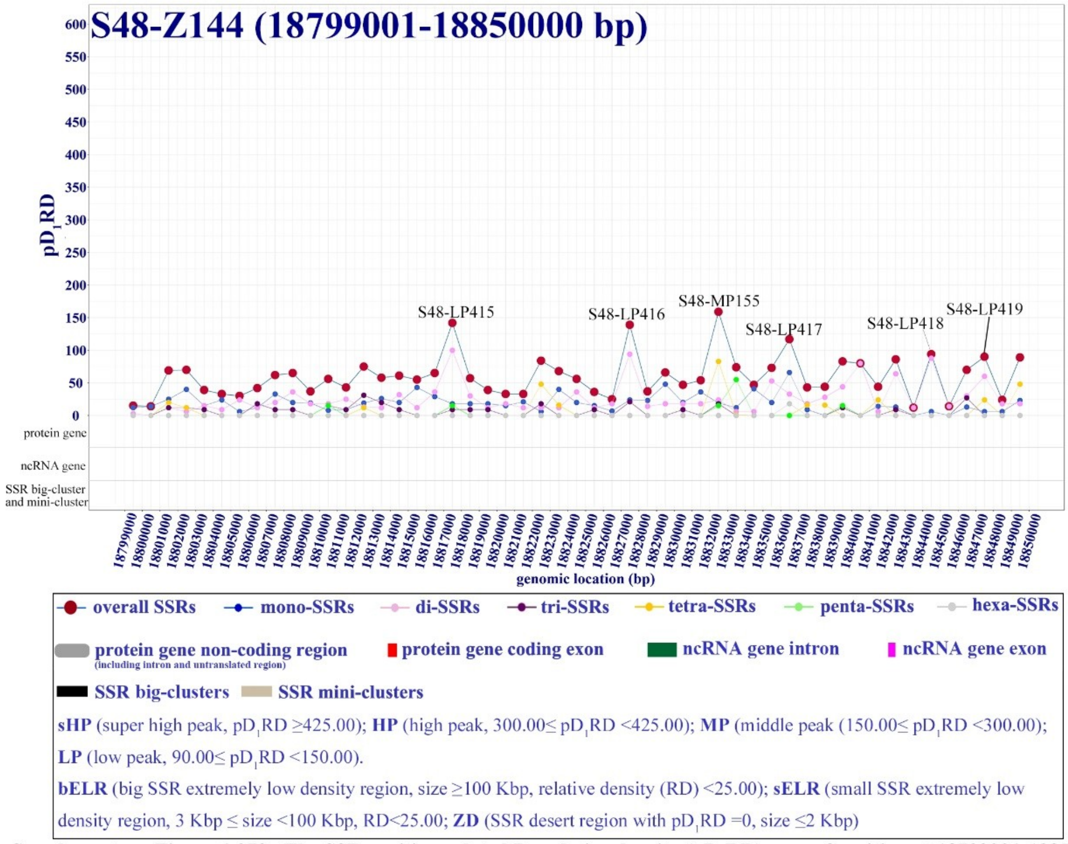

Supplementary Figure 1.372. The SSR position related D_1-relative density (pD_1RD) map of position at 18799001-18850000 bp of human reference Y-DNA (NC_000024.10) at resolution of 1 Kbp.

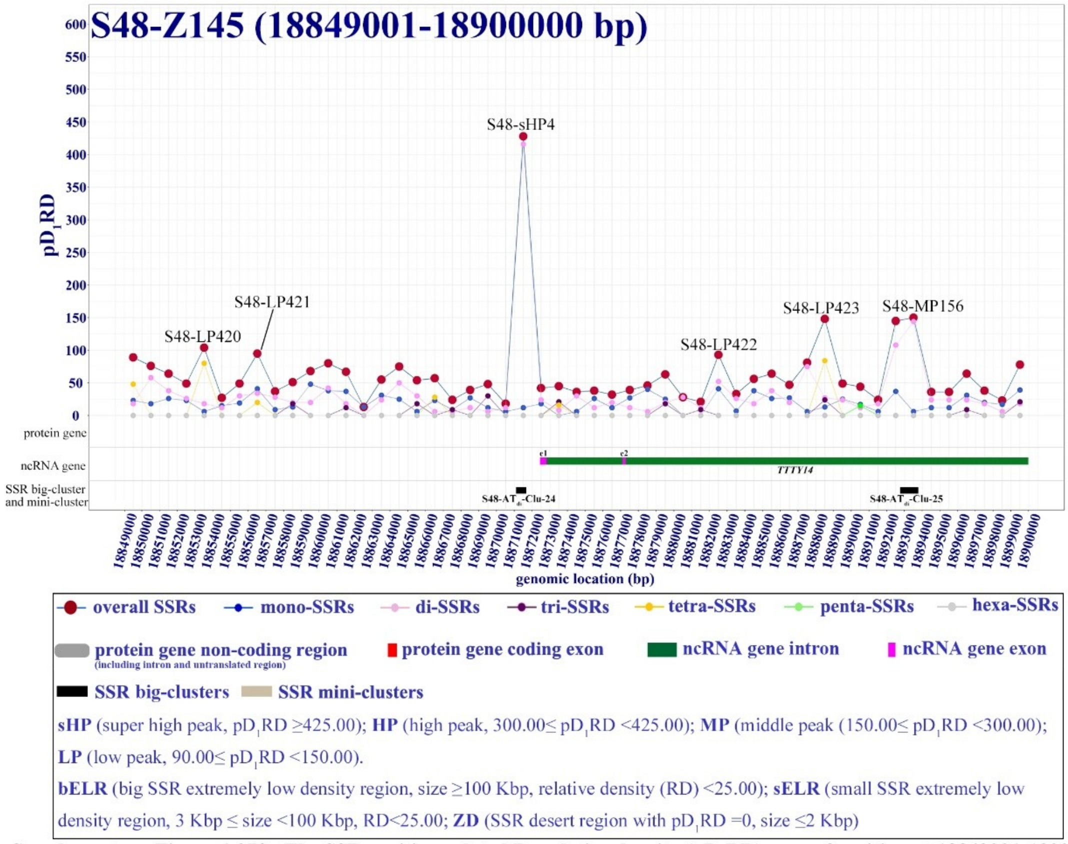

Supplementary Figure 1.373. The SSR position related D_1-relative density (pD_1RD) map of position at 18849001-18900000 bp of human reference Y-DNA (NC_000024.10) at resolution of 1 Kbp.

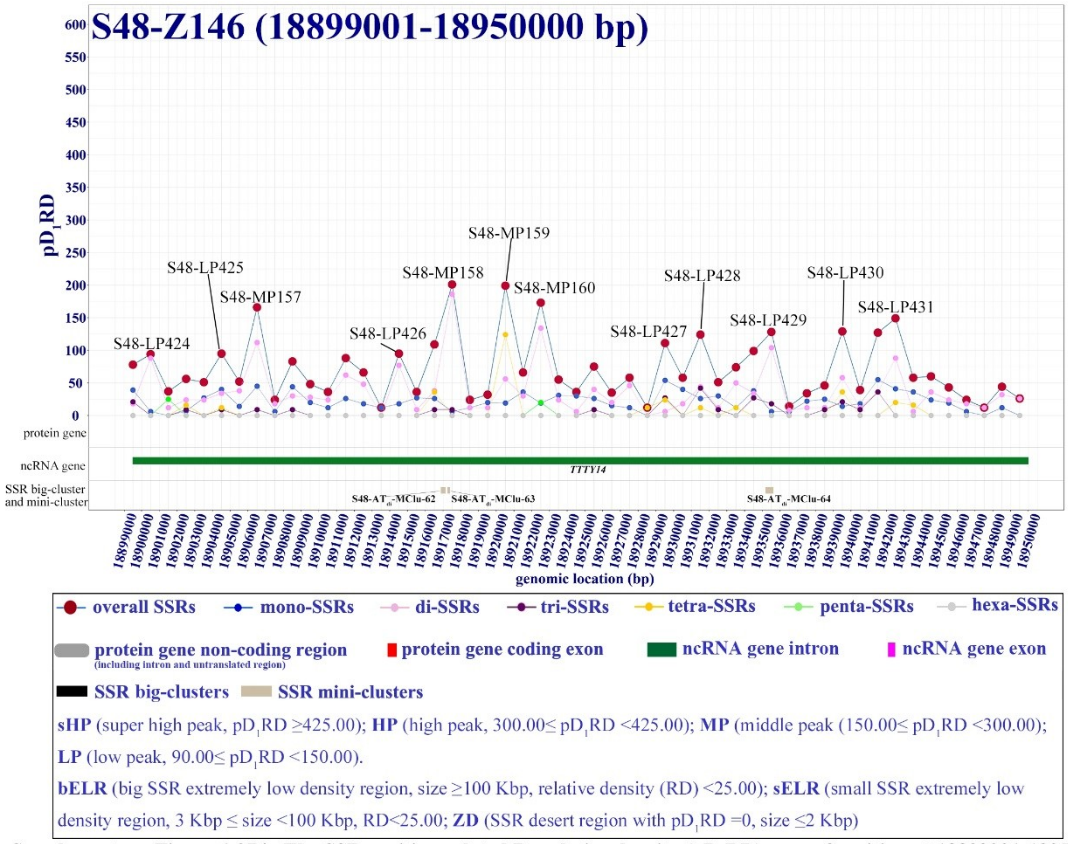

Supplementary Figure 1.374. The SSR position related D_1-relative density (pD_1RD) map of position at 18899001-18950000 bp of human reference Y-DNA (NC_000024.10) at resolution of 1 Kbp.

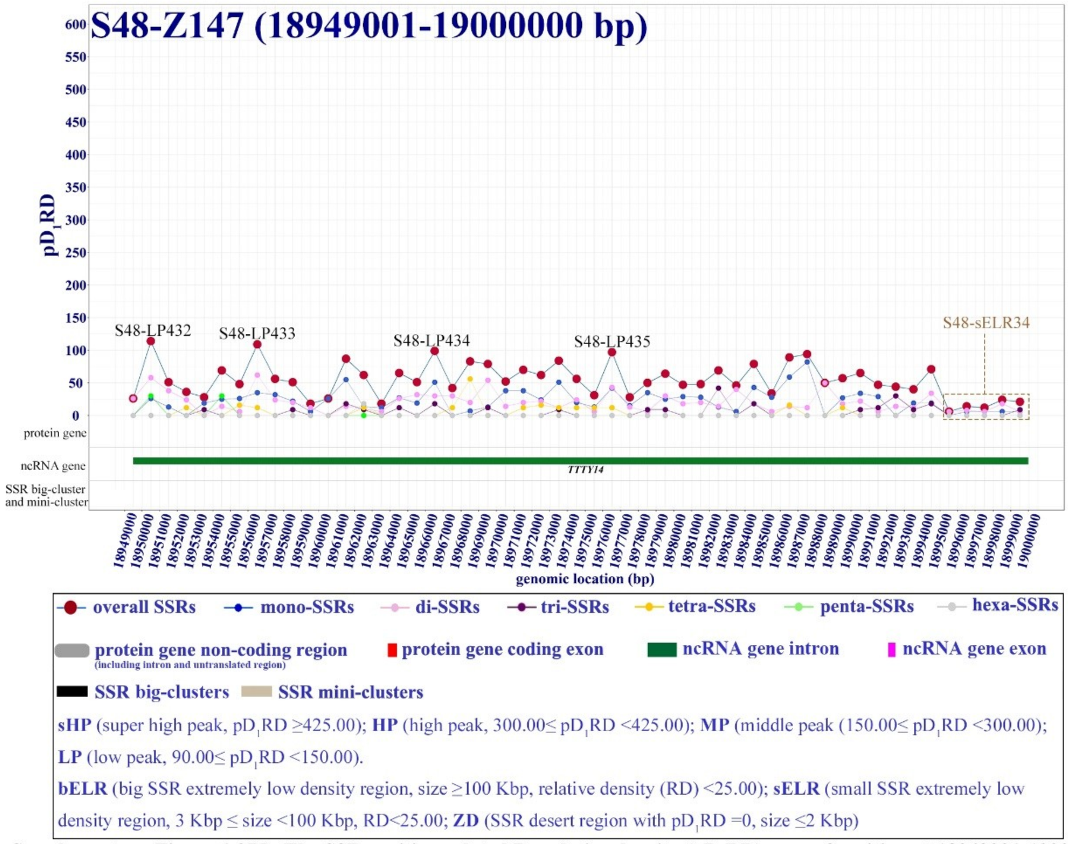

Supplementary Figure 1.375. The SSR position related D_1-relative density (pD_1RD) map of position at 18949001-19000000 bp of human reference Y-DNA (NC_000024.10) at resolution of 1 Kbp.

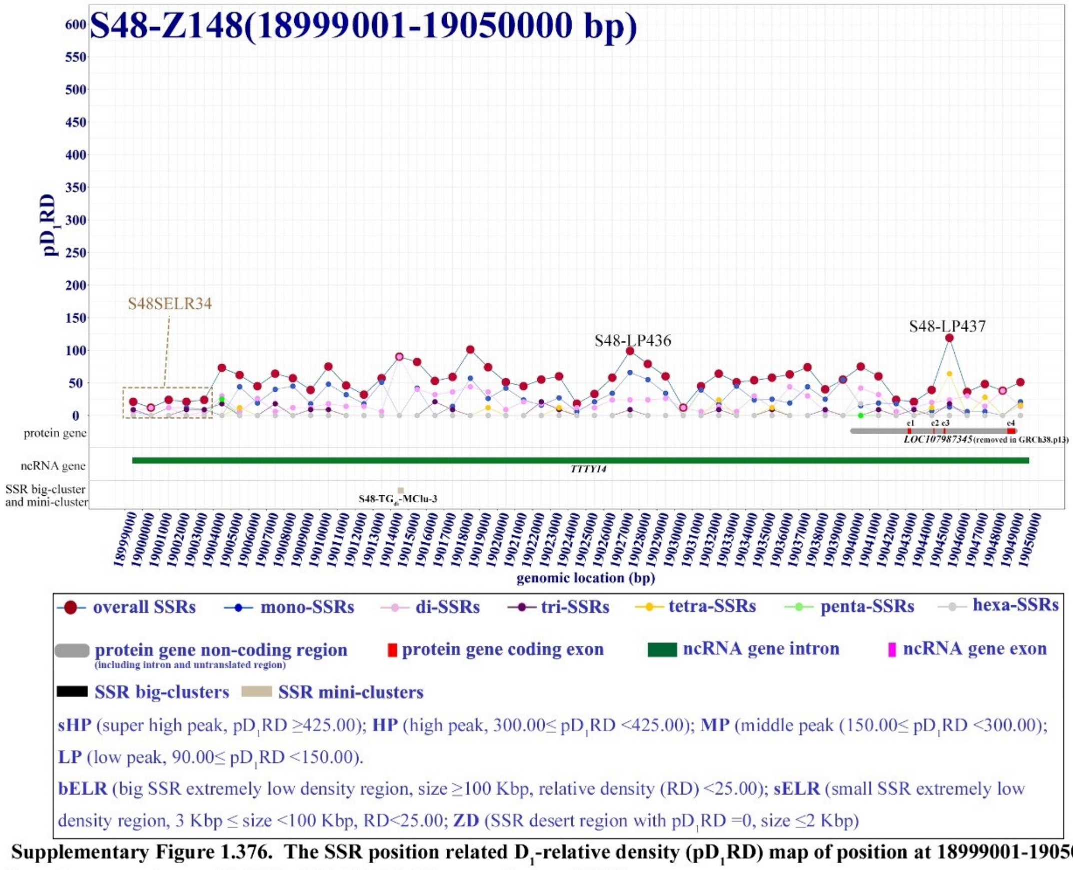

Supplementary Figure 1.376. The SSR position related D_1-relative density (pD_1RD) map of position at 18999001-19050000 bp of human reference Y-DNA (NC_000024.10) at resolution of 1 Kbp.

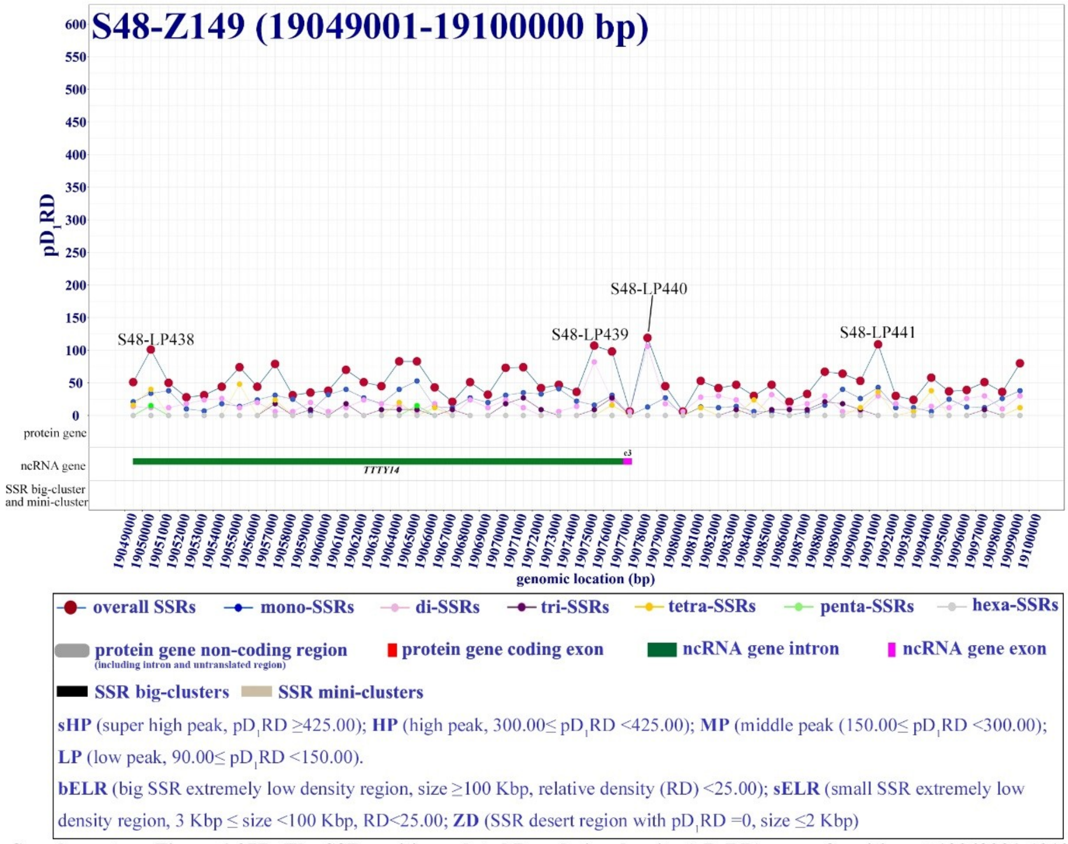

Supplementary Figure 1.377. The SSR position related D_1-relative density (pD_1RD) map of position at 19049001-19100000 bp of human reference Y-DNA (NC_000024.10) at resolution of 1 Kbp.

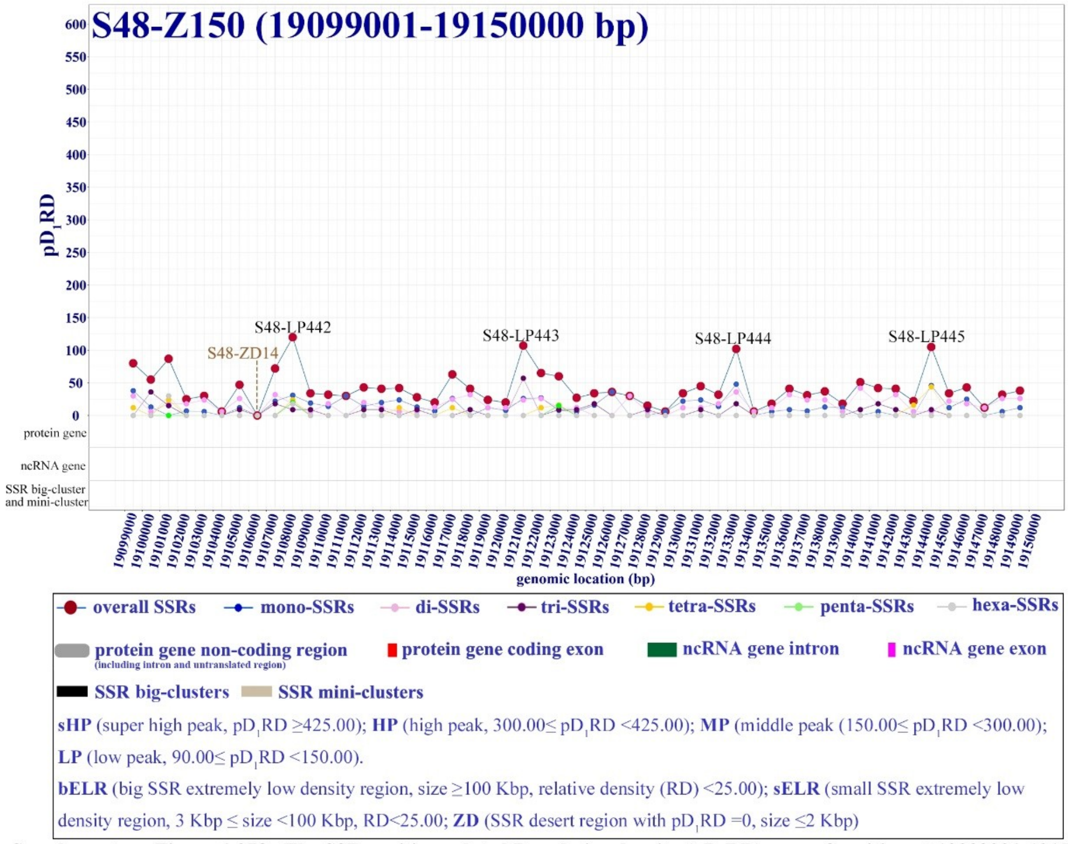

Supplementary Figure 1.378. The SSR position related D_1-relative density (pD_1RD) map of position at 19099001-19150000 bp of human reference Y-DNA (NC_000024.10) at resolution of 1 Kbp.

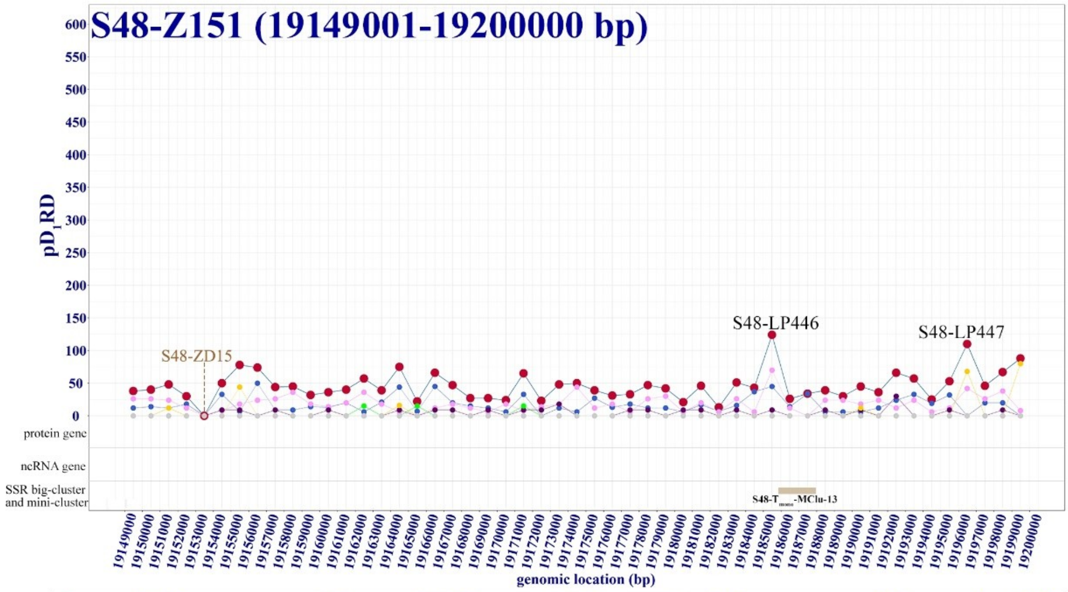

overall SSRs — mono-SSRs — di-SSRs — tri-SSRs — tetra-SSRs — penta-SSRs — hexa-SSRs

protein gene non-coding region (including intron and untranslated region) — protein gene coding exon — ncRNA gene intron — ncRNA gene exon

SSR big-clusters — SSR mini-clusters

sHP (super high peak, $pD_1RD \geq 425.00$); **HP** (high peak, $300.00 \leq pD_1RD < 425.00$); **MP** (middle peak ($150.00 \leq pD_1RD < 300.00$); **LP** (low peak, $90.00 \leq pD_1RD < 150.00$).

bELR (big SSR extremely low density region, size ≥100 Kbp, relative density (RD) <25.00); **sELR** (small SSR extremely low density region, 3 Kbp ≤ size <100 Kbp, RD<25.00; **ZD** (SSR desert region with $pD_1RD = 0$, size ≤2 Kbp)

Supplementary Figure 1.379. The SSR position related D_1-relative density (pD_1RD) map of position at 19149001-19200000 bp of human reference Y-DNA (NC_000024.10) at resolution of 1 Kbp.

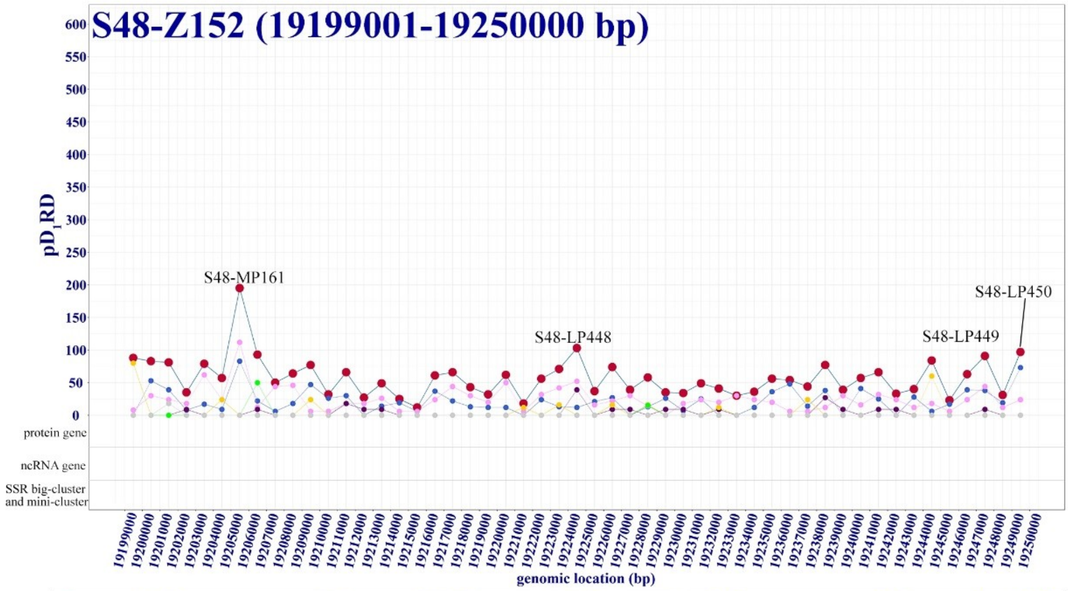

overall SSRs — mono-SSRs — di-SSRs — tri-SSRs — tetra-SSRs — penta-SSRs — hexa-SSRs

protein gene non-coding region (including intron and untranslated region) — protein gene coding exon — ncRNA gene intron — ncRNA gene exon

SSR big-clusters — SSR mini-clusters

sHP (super high peak, $pD_1RD \geq 425.00$); **HP** (high peak, $300.00 \leq pD_1RD < 425.00$); **MP** (middle peak ($150.00 \leq pD_1RD < 300.00$); **LP** (low peak, $90.00 \leq pD_1RD < 150.00$).

bELR (big SSR extremely low density region, size ≥100 Kbp, relative density (RD) <25.00); **sELR** (small SSR extremely low density region, 3 Kbp ≤ size <100 Kbp, RD<25.00; **ZD** (SSR desert region with $pD_1RD = 0$, size ≤2 Kbp)

Supplementary Figure 1.380. The SSR position related D_1-relative density (pD_1RD) map of position at 19199001-19250000 bp of human reference Y-DNA (NC_000024.10) at resolution of 1 Kbp.

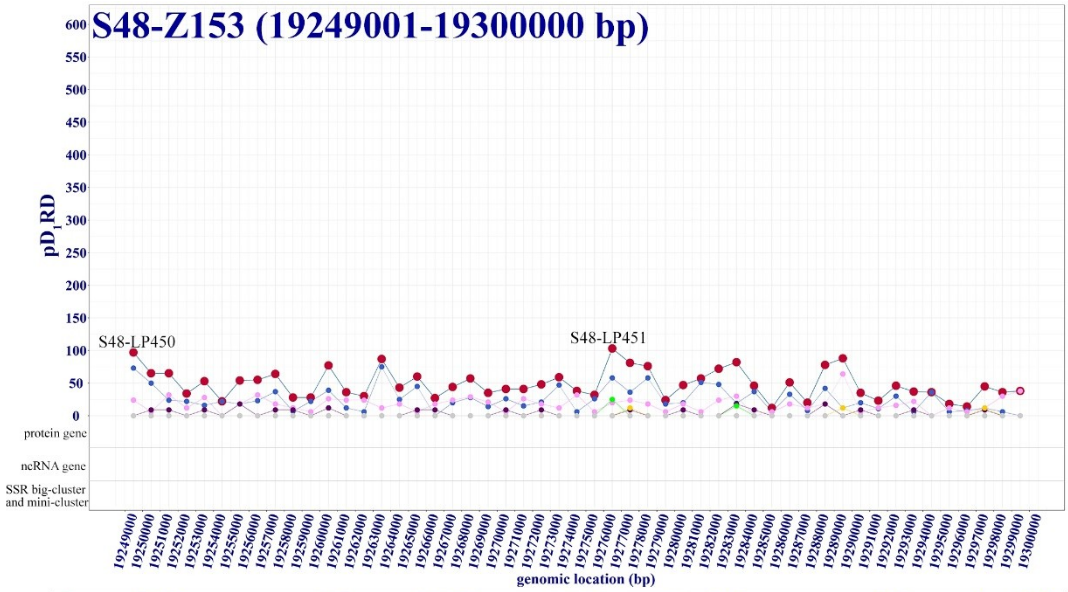

overall SSRs — mono-SSRs — di-SSRs — tri-SSRs — tetra-SSRs — penta-SSRs — hexa-SSRs

protein gene non-coding region (including intron and untranslated region) — protein gene coding exon — ncRNA gene intron — ncRNA gene exon

SSR big-clusters — SSR mini-clusters

sHP (super high peak, $pD_1RD \geq 425.00$); **HP** (high peak, $300.00 \leq pD_1RD < 425.00$); **MP** (middle peak ($150.00 \leq pD_1RD < 300.00$); **LP** (low peak, $90.00 \leq pD_1RD < 150.00$).

bELR (big SSR extremely low density region, size ≥100 Kbp, relative density (RD) <25.00); **sELR** (small SSR extremely low density region, 3 Kbp ≤ size <100 Kbp, RD<25.00; **ZD** (SSR desert region with $pD_1RD = 0$, size ≤2 Kbp)

Supplementary Figure 1.381. The SSR position related D_1-relative density (pD_1RD) map of position at 19249001-19300000 bp of human reference Y-DNA (NC_000024.10) at resolution of 1 Kbp.

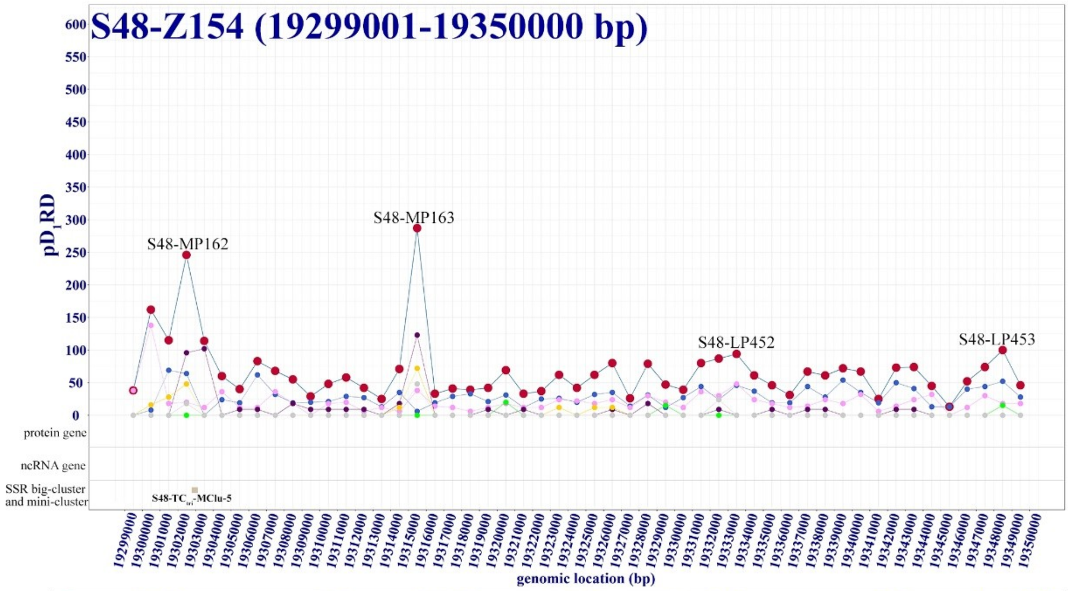

overall SSRs — mono-SSRs — di-SSRs — tri-SSRs — tetra-SSRs — penta-SSRs — hexa-SSRs

protein gene non-coding region (including intron and untranslated region) — protein gene coding exon — ncRNA gene intron — ncRNA gene exon

SSR big-clusters — SSR mini-clusters

sHP (super high peak, $pD_1RD \geq 425.00$); **HP** (high peak, $300.00 \leq pD_1RD < 425.00$); **MP** (middle peak ($150.00 \leq pD_1RD < 300.00$); **LP** (low peak, $90.00 \leq pD_1RD < 150.00$).

bELR (big SSR extremely low density region, size ≥100 Kbp, relative density (RD) <25.00); **sELR** (small SSR extremely low density region, 3 Kbp ≤ size <100 Kbp, RD<25.00; **ZD** (SSR desert region with $pD_1RD = 0$, size ≤2 Kbp)

Supplementary Figure 1.382. The SSR position related D_1-relative density (pD_1RD) map of position at 19299001-19350000 bp of human reference Y-DNA (NC_000024.10) at resolution of 1 Kbp.

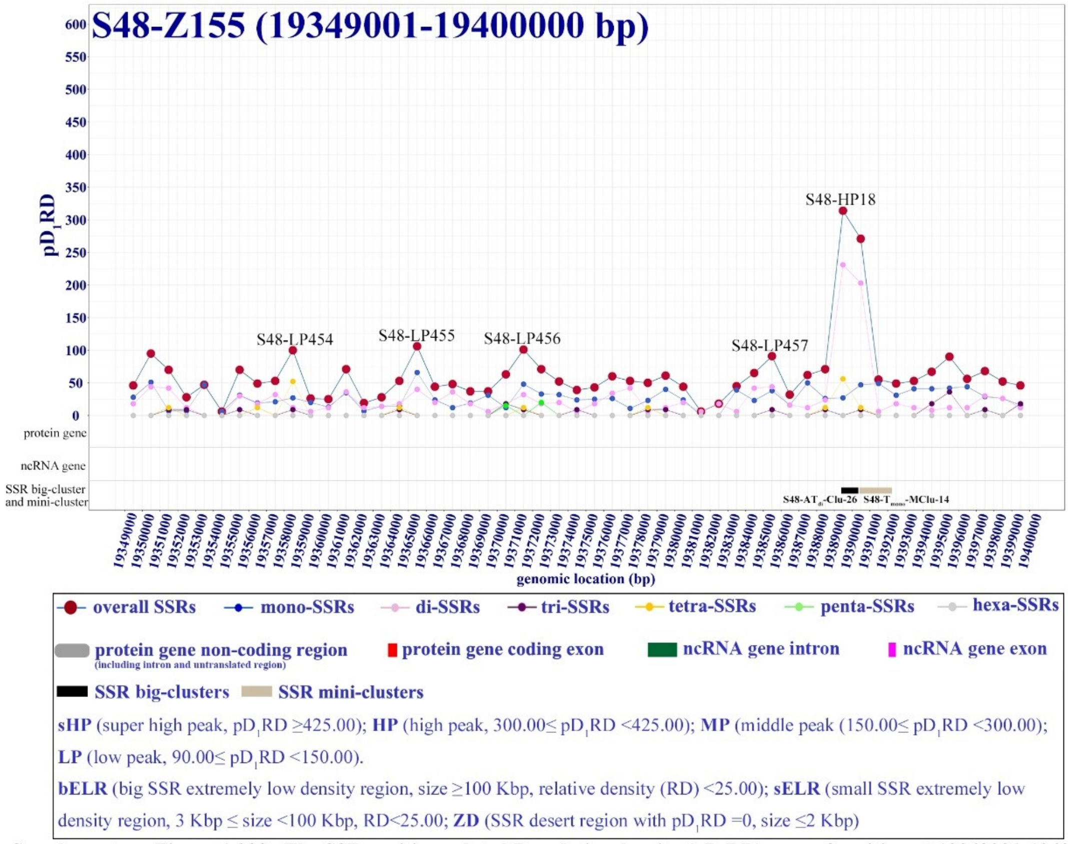

Supplementary Figure 1.383. The SSR position related D_1-relative density (pD_1RD) map of position at 19349001-19400000 bp of human reference Y-DNA (NC_000024.10) at resolution of 1 Kbp.

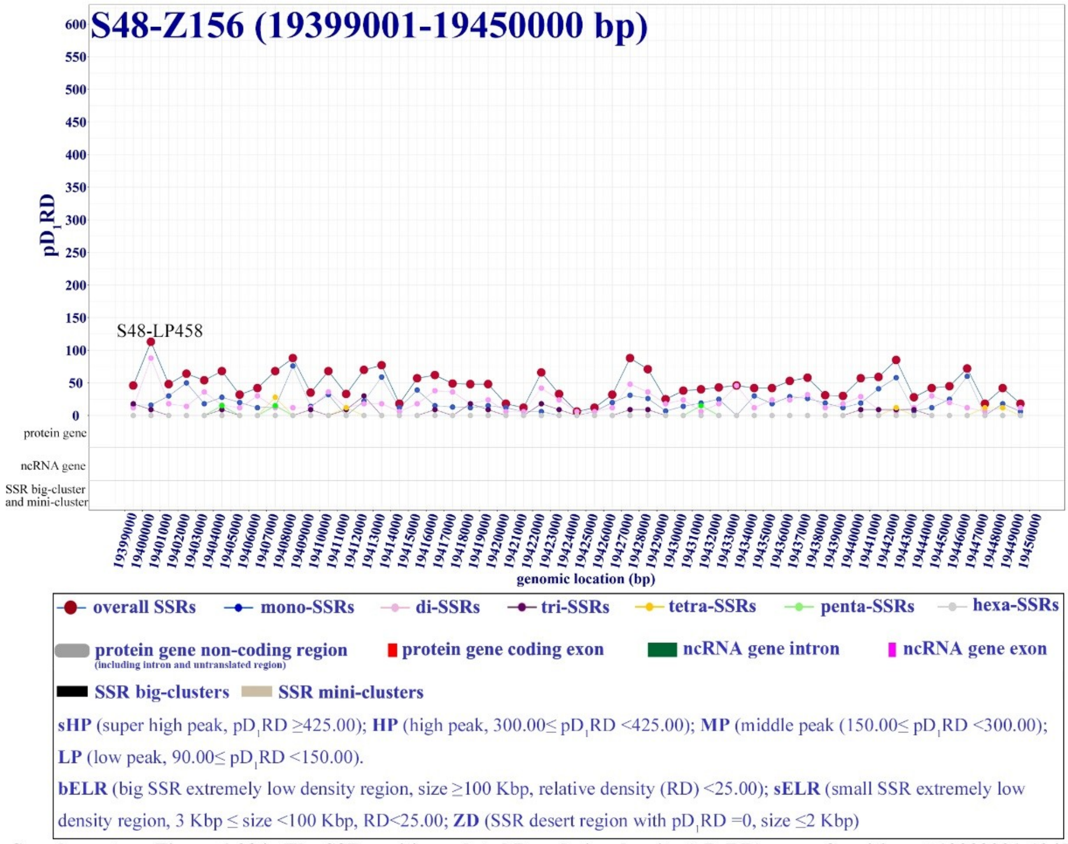

Supplementary Figure 1.384. The SSR position related D_1-relative density (pD_1RD) map of position at 19399001-19450000 bp of human reference Y-DNA (NC_000024.10) at resolution of 1 Kbp.

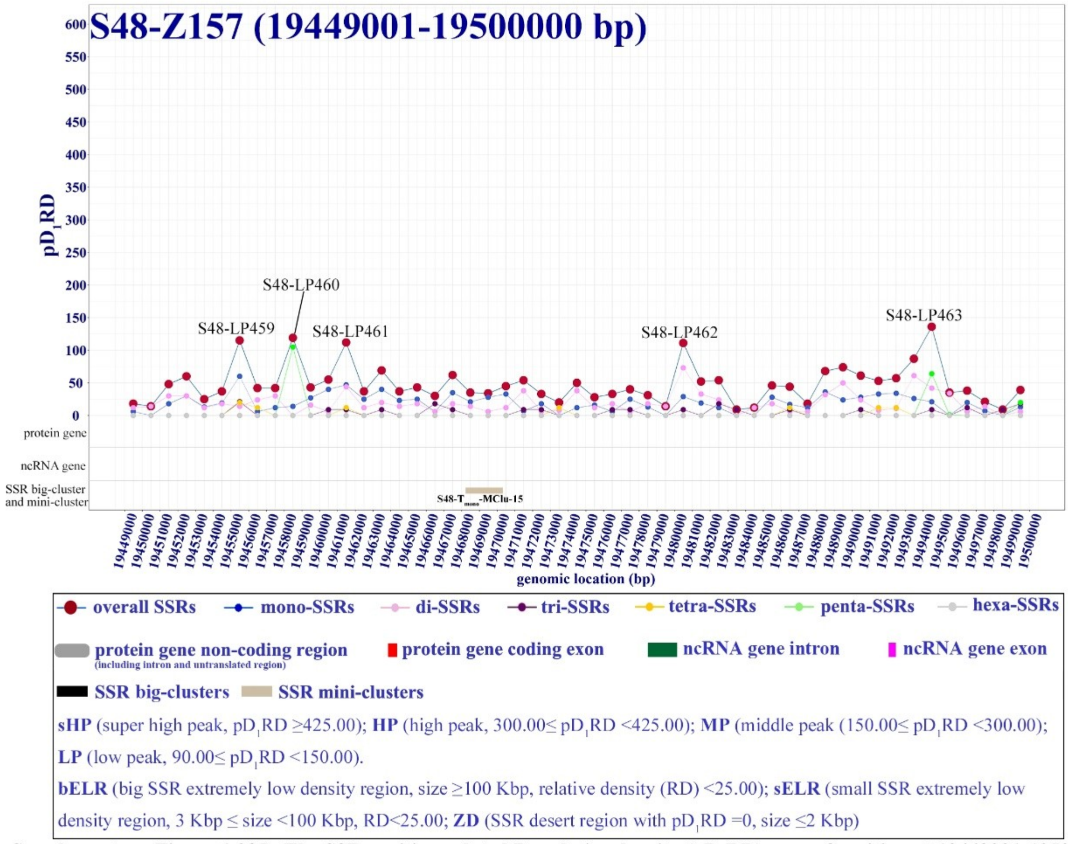

Supplementary Figure 1.385. The SSR position related D_1-relative density (pD_1RD) map of position at 19449001-19500000 bp of human reference Y-DNA (NC_000024.10) at resolution of 1 Kbp.

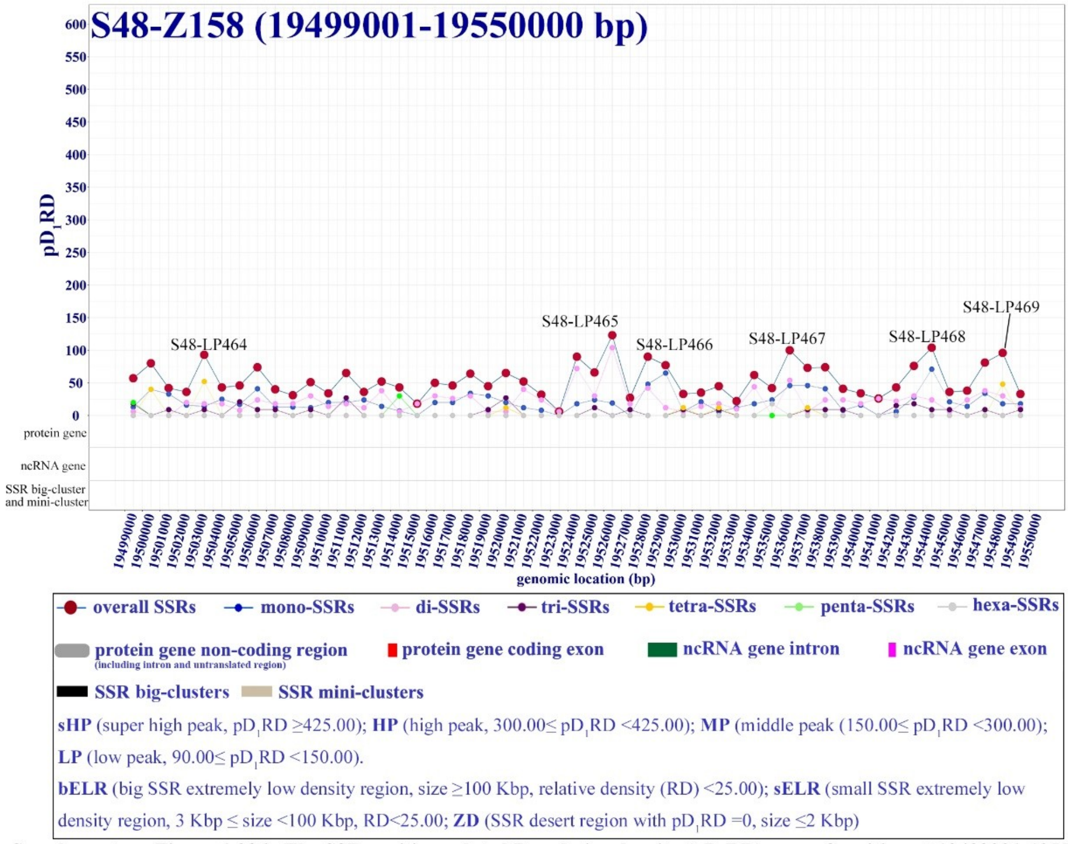

Supplementary Figure 1.386. The SSR position related D_1-relative density (pD_1RD) map of position at 19499001-19550000 bp of human reference Y-DNA (NC_000024.10) at resolution of 1 Kbp.

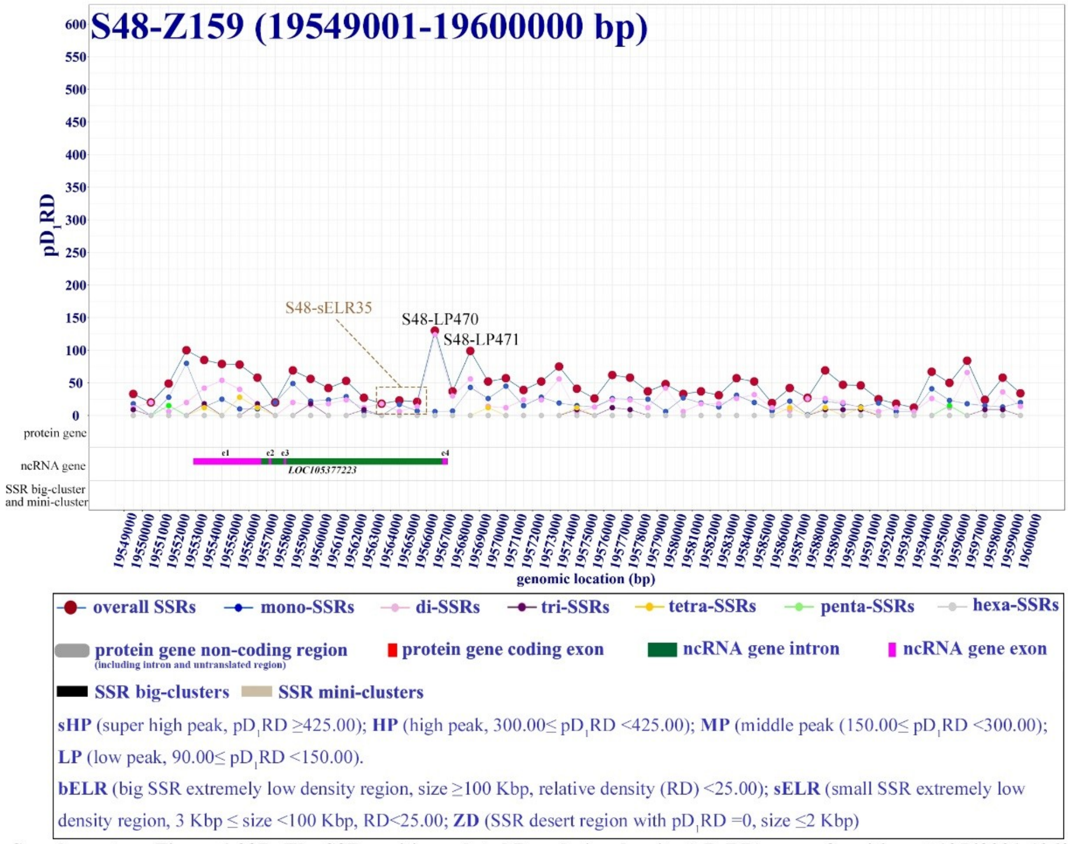

Supplementary Figure 1.387. The SSR position related D_1-relative density (pD_1RD) map of position at 19549001-19600000 bp of human reference Y-DNA (NC_000024.10) at resolution of 1 Kbp.

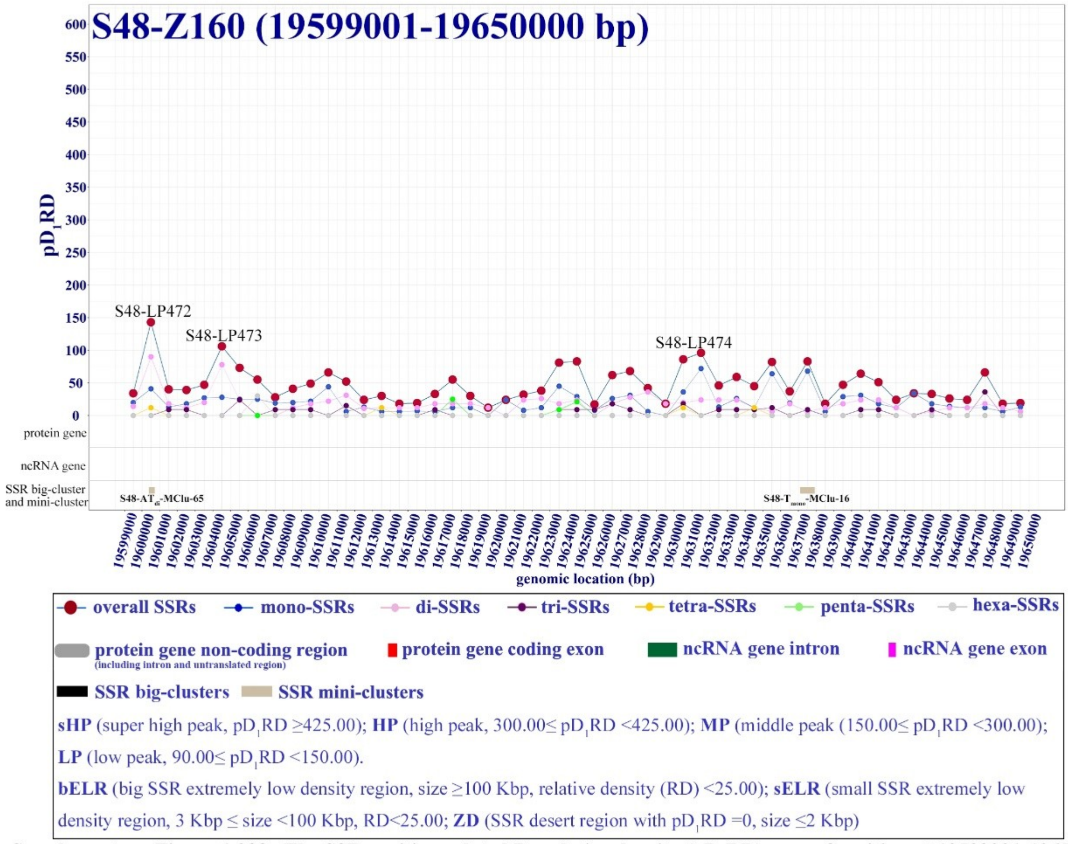

Supplementary Figure 1.388. The SSR position related D_1-relative density (pD_1RD) map of position at 19599001-19650000 bp of human reference Y-DNA (NC_000024.10) at resolution of 1 Kbp.

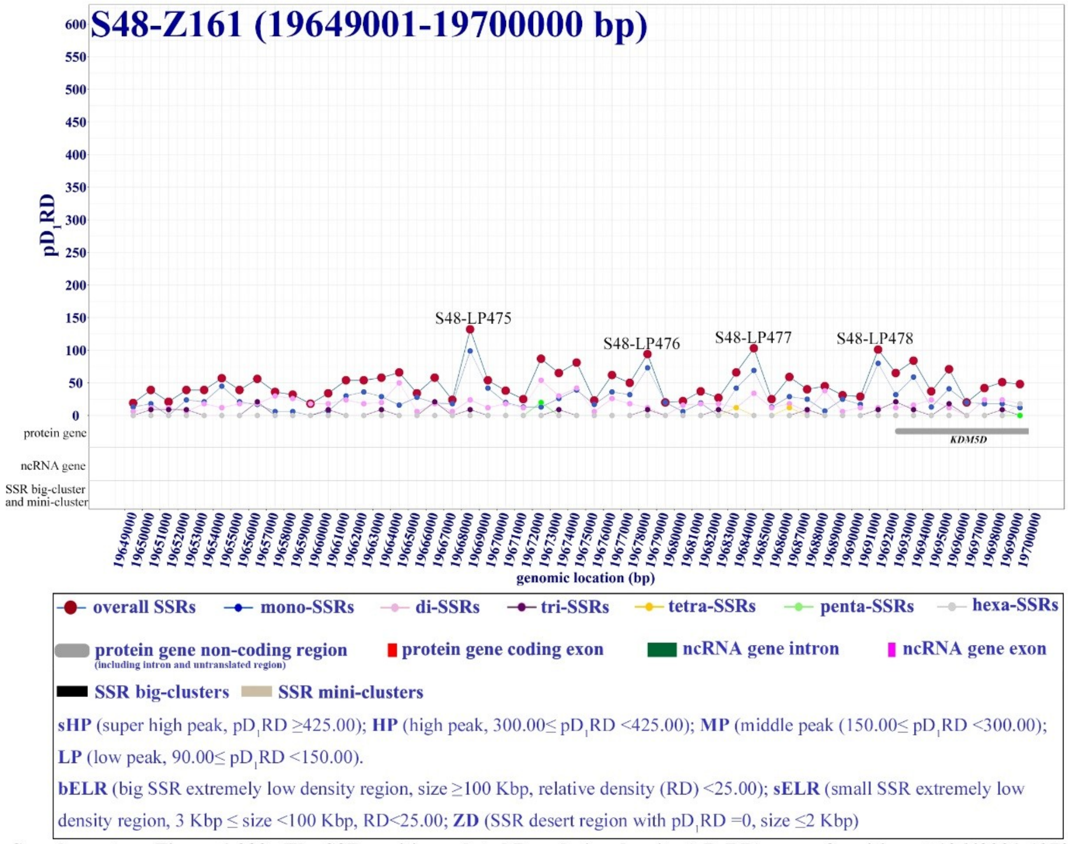

Supplementary Figure 1.389. The SSR position related D_1-relative density (pD_1RD) map of position at 19649001-19700000 bp of human reference Y-DNA (NC_000024.10) at resolution of 1 Kbp.

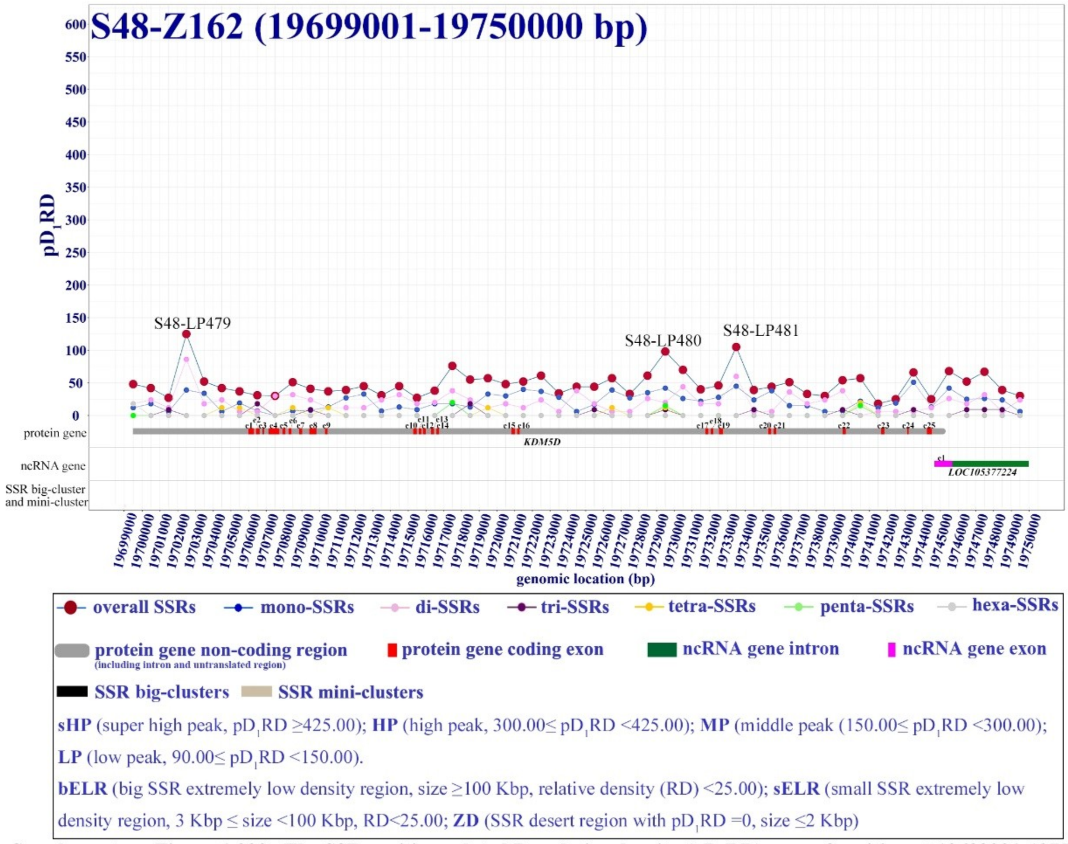

Supplementary Figure 1.390. The SSR position related D_1-relative density (pD_1RD) map of position at 19699001-19750000 bp of human reference Y-DNA (NC_000024.10) at resolution of 1 Kbp.

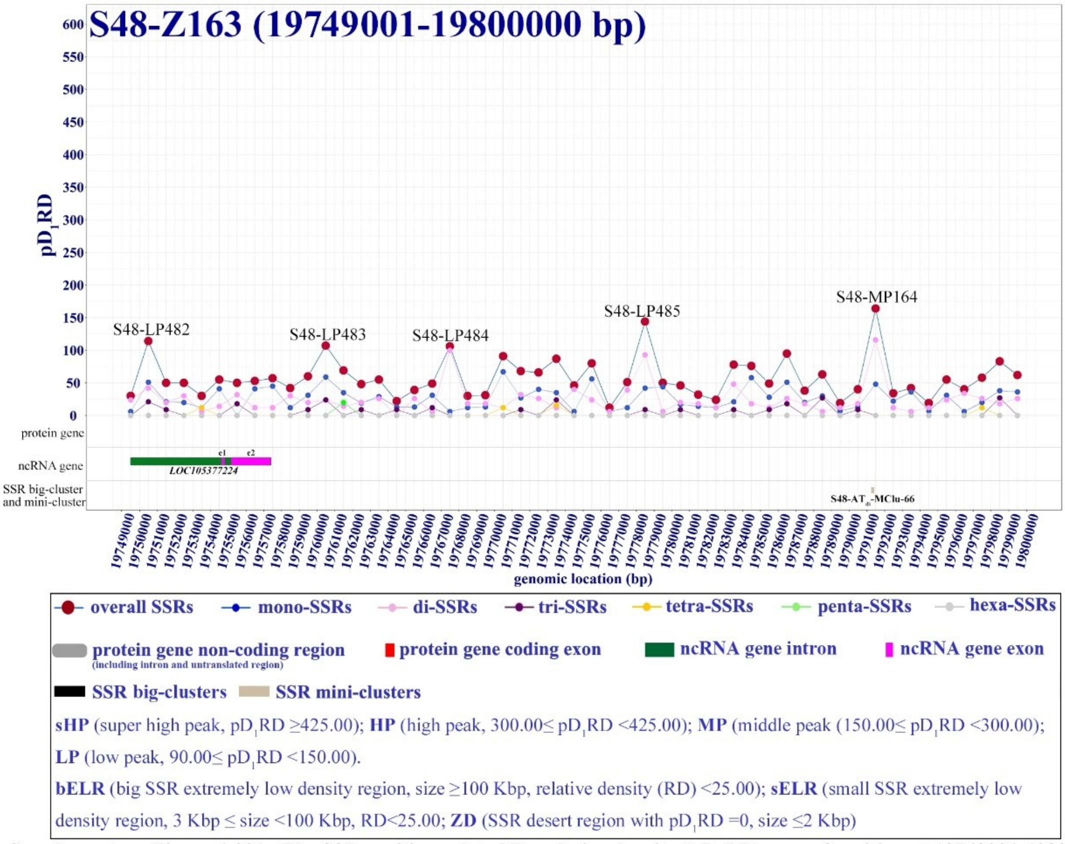

Supplementary Figure 1.391. The SSR position related D_1-relative density (pD_1RD) map of position at 19749001-19800000 bp of human reference Y-DNA (NC_000024.10) at resolution of 1 Kbp.

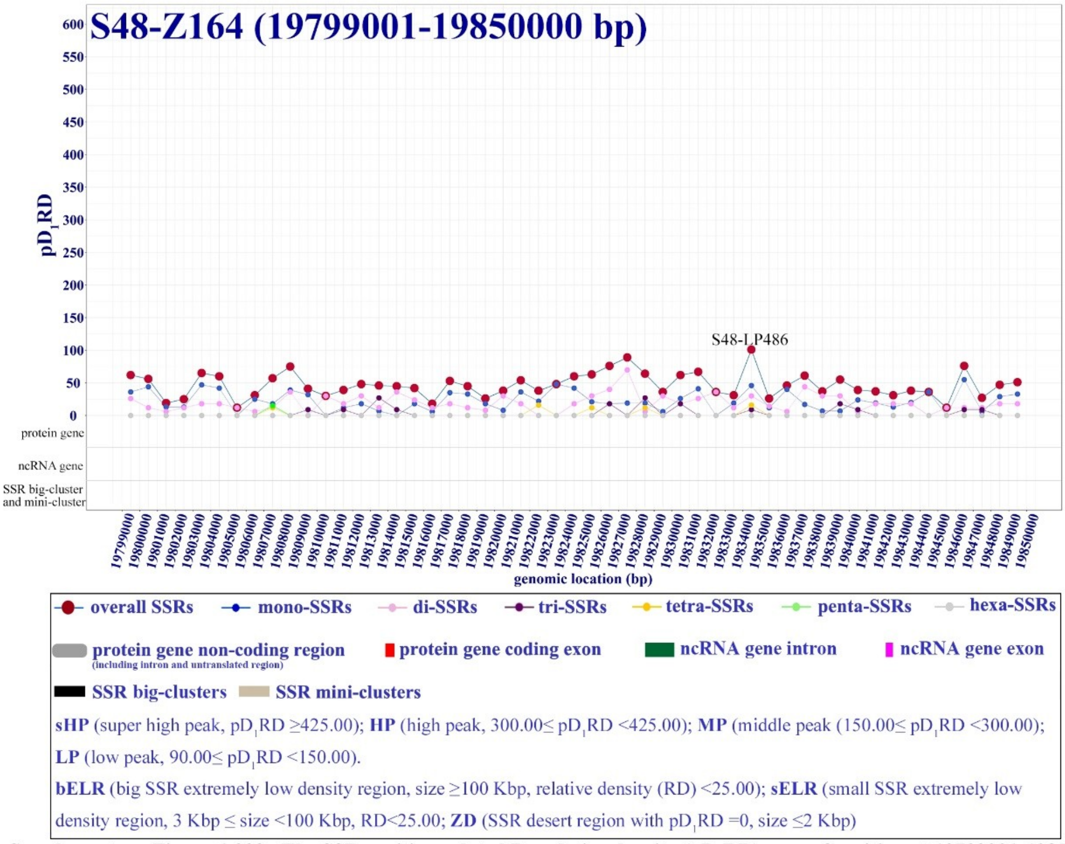

Supplementary Figure 1.392. The SSR position related D_1-relative density (pD_1RD) map of position at 19799001-19850000 bp of human reference Y-DNA (NC_000024.10) at resolution of 1 Kbp.

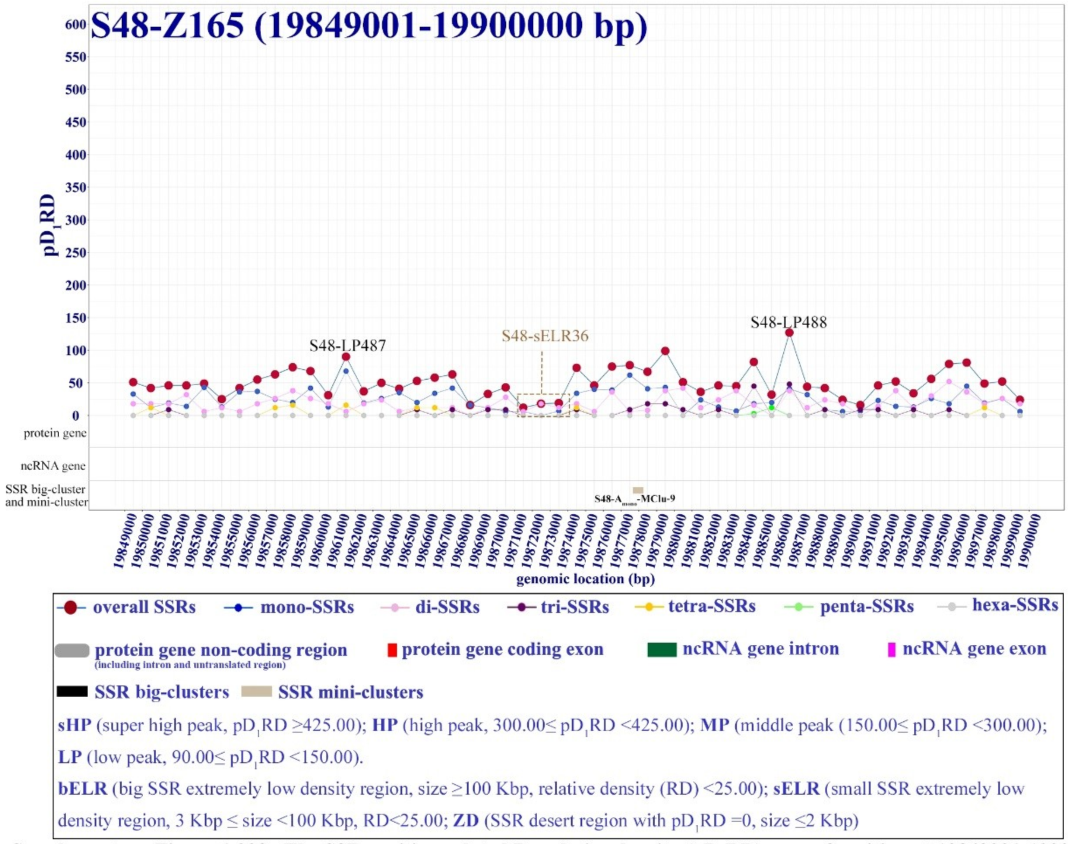

Supplementary Figure 1.393. The SSR position related D_1-relative density (pD_1RD) map of position at 19849001-19900000 bp of human reference Y-DNA (NC_000024.10) at resolution of 1 Kbp.

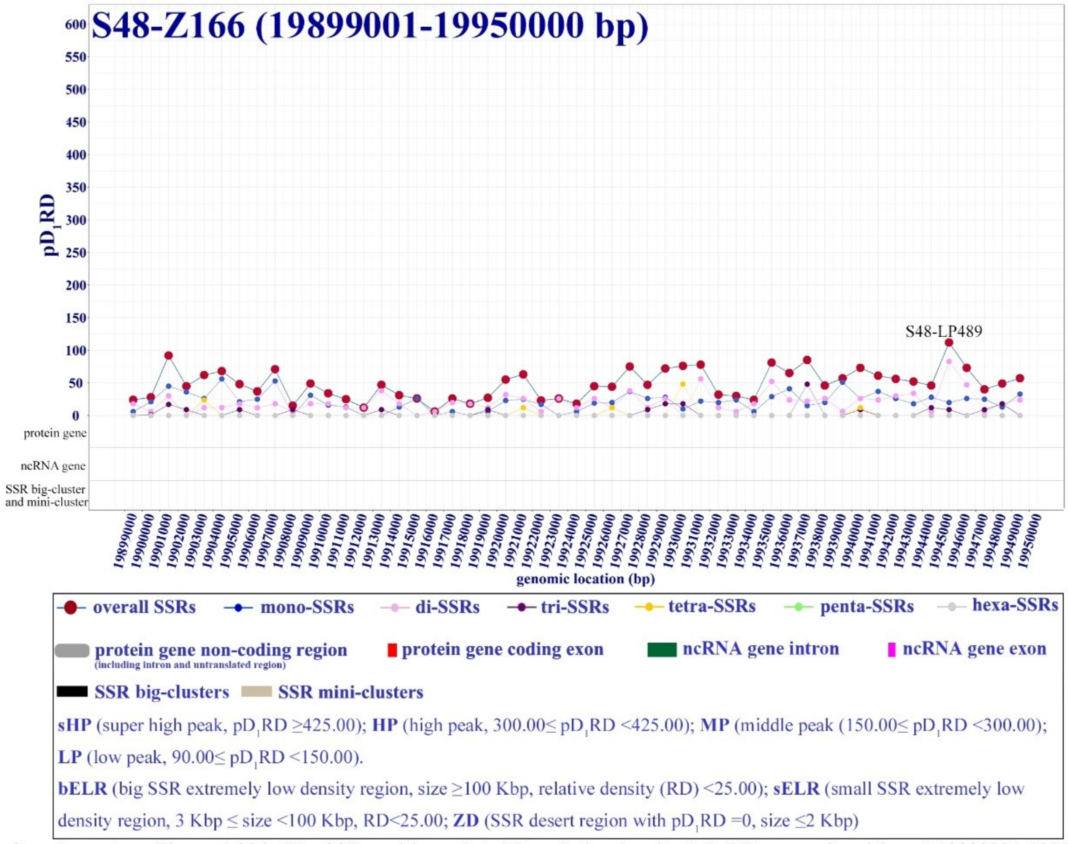

Supplementary Figure 1.394. The SSR position related D_1-relative density (pD_1RD) map of position at 19899001-19950000 bp of human reference Y-DNA (NC_000024.10) at resolution of 1 Kbp.

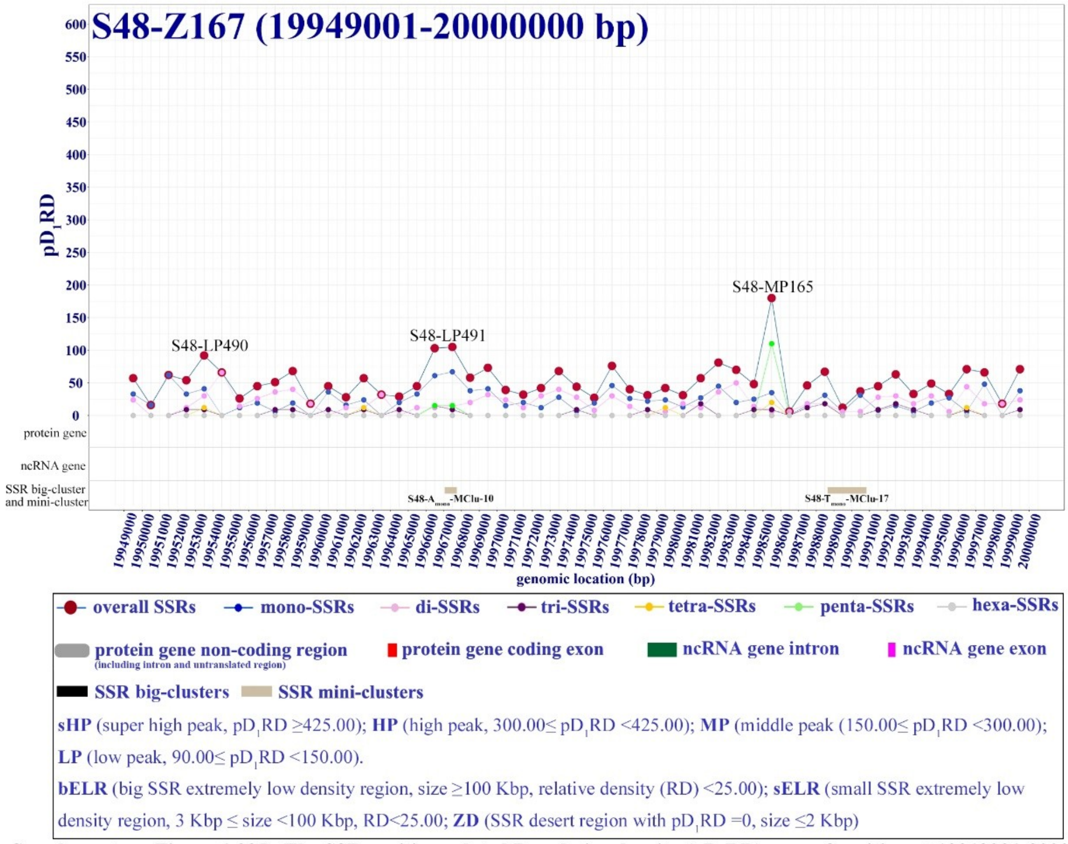

Supplementary Figure 1.395. The SSR position related D_1-relative density (pD_1RD) map of position at 19919001-20000000 bp of human reference Y-DNA (NC_000024.10) at resolution of 1 Kbp.

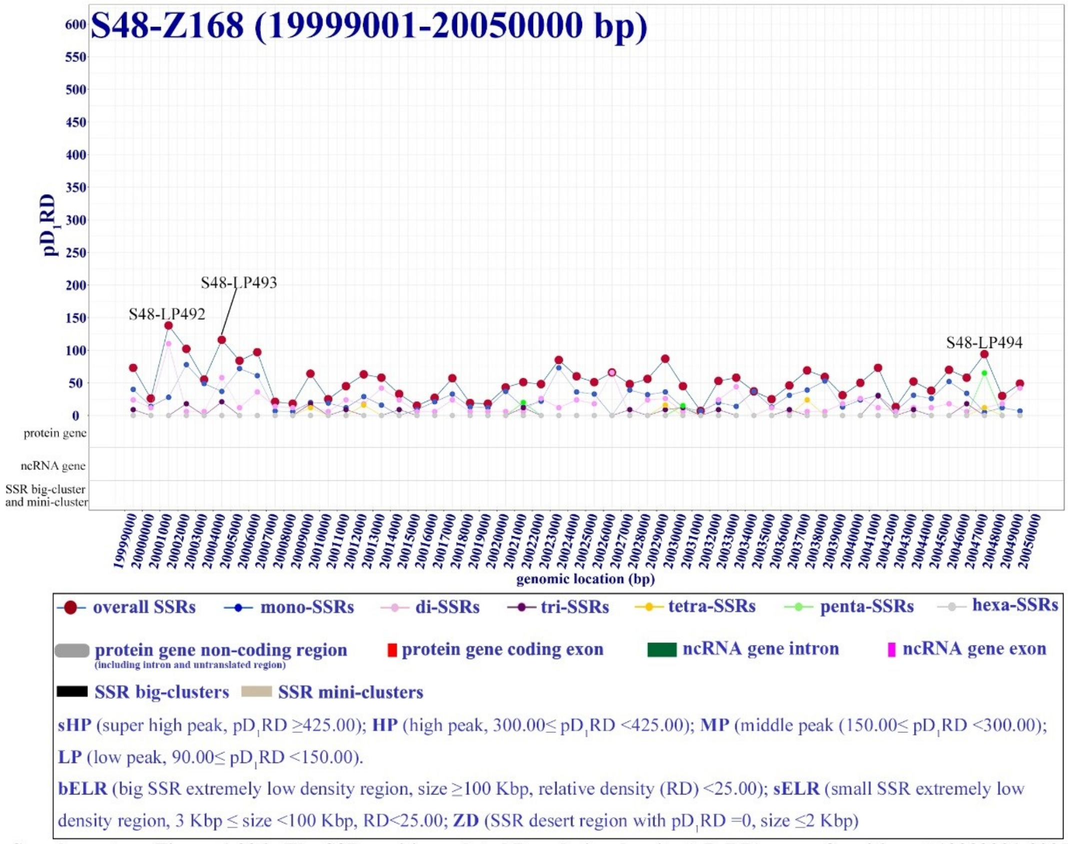

Supplementary Figure 1.396. The SSR position related D_1-relative density (pD_1RD) map of position at 19999001-20050000 bp of human reference Y-DNA (NC_000024.10) at resolution of 1 Kbp.

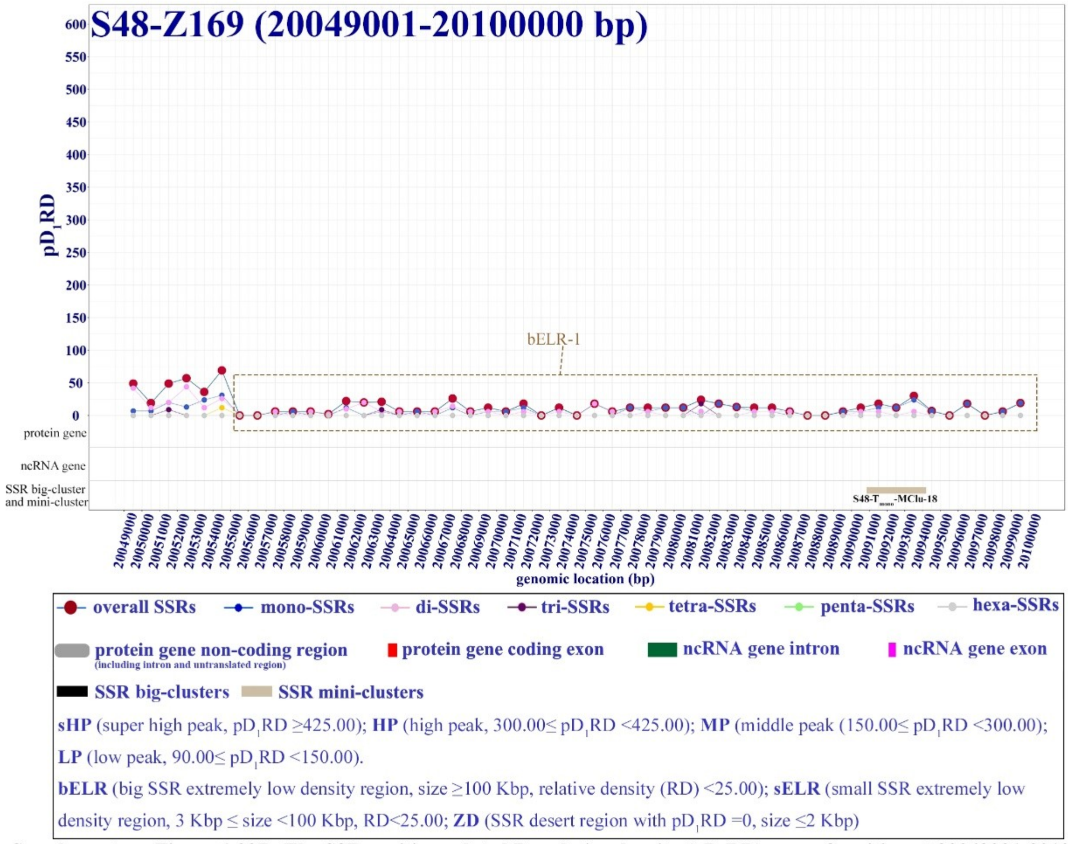

Supplementary Figure 1.397. The SSR position related D_1-relative density (pD_1RD) map of position at 20049001-20100000 bp of human reference Y-DNA (NC_000024.10) at resolution of 1 Kbp.

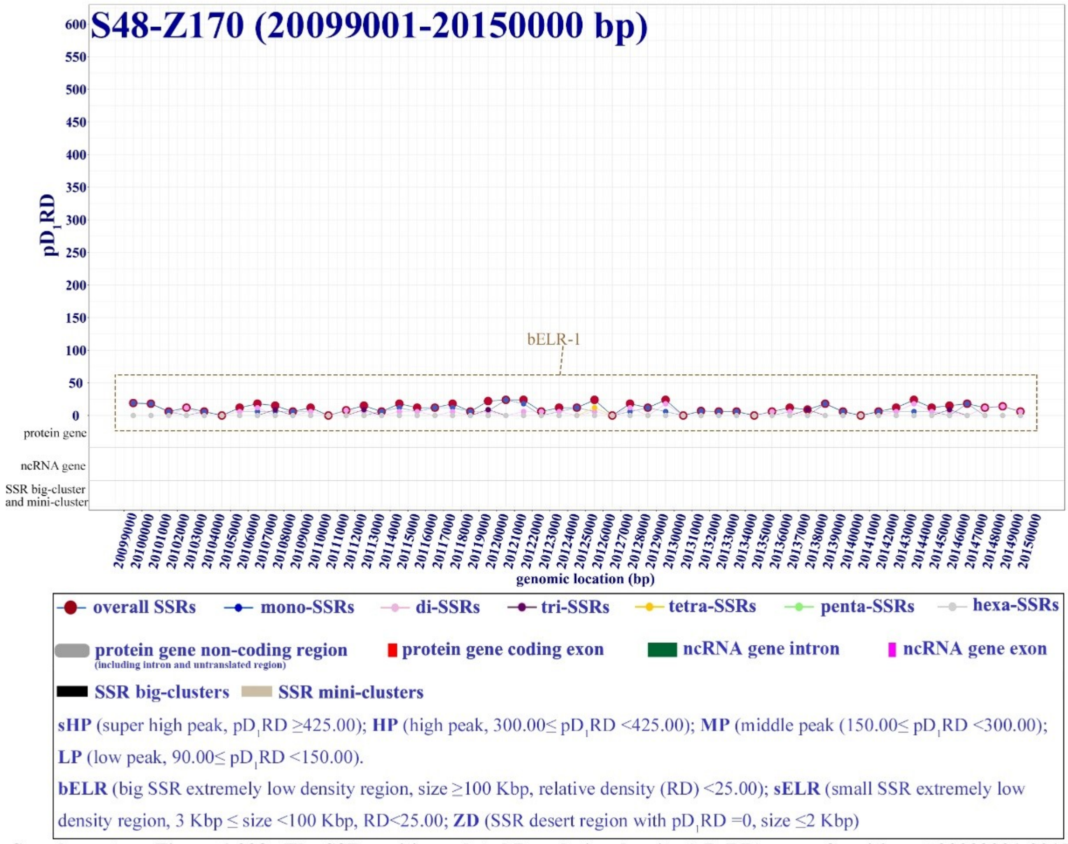

Supplementary Figure 1.398. The SSR position related D_1-relative density (pD_1RD) map of position at 20099001-20150000 bp of human reference Y-DNA (NC_000024.10) at resolution of 1 Kbp.

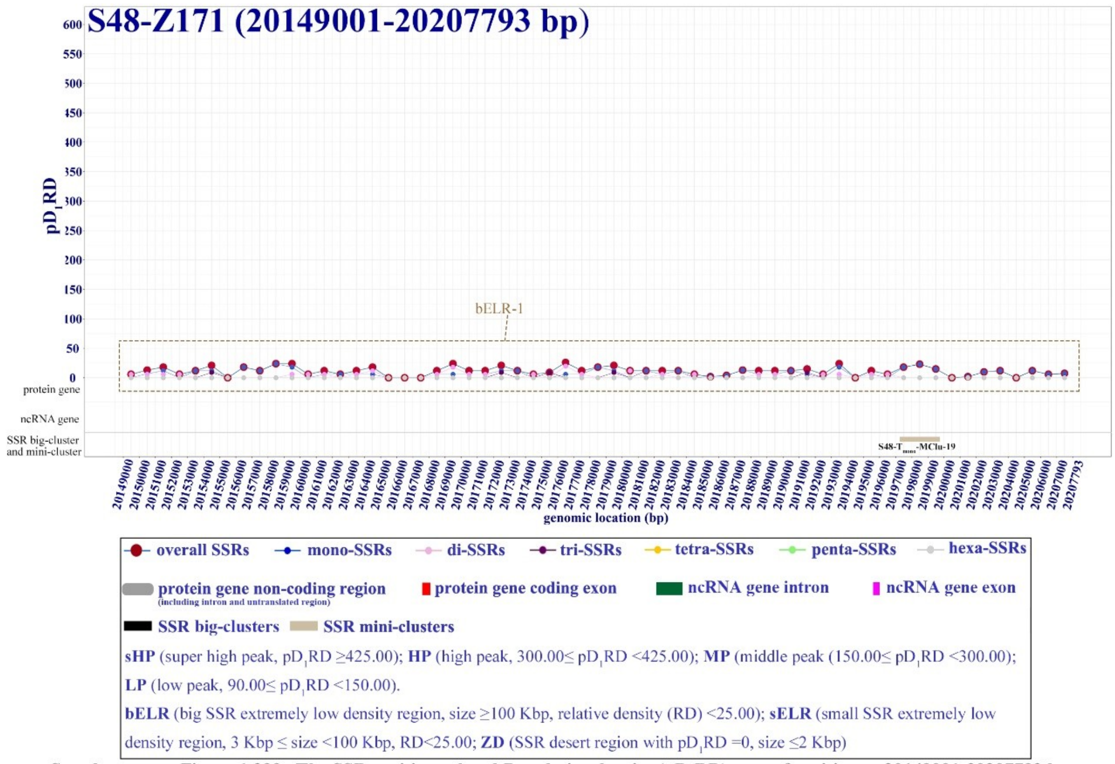

Supplementary Figure 1.399. The SSR position related D_1-relative density (pD_1RD) map of position at 20149001-20207793 bp (unnormal zone >51000 bp) of human reference Y-DNA (NC_000024.10) at resolution of 1 Kbp.

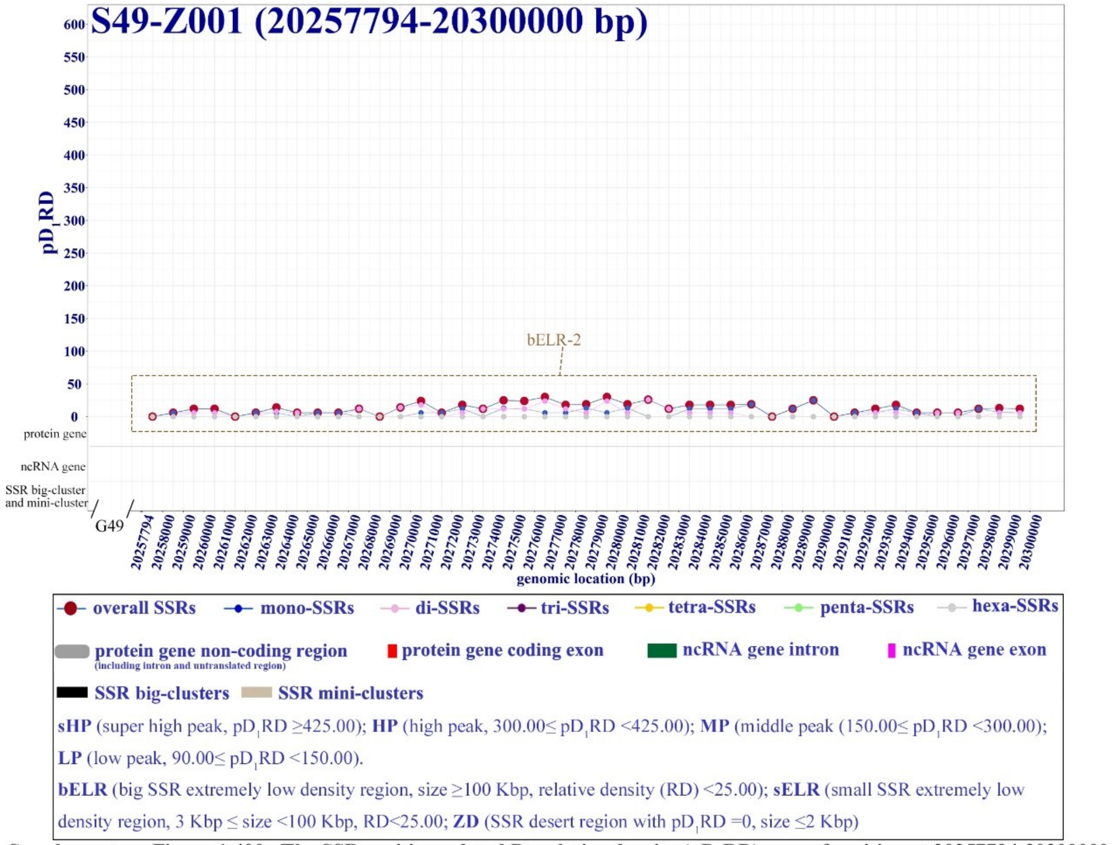

Supplementary Figure 1.400. The SSR position related D_1-relative density (pD_1RD) map of position at 20257794-20300000 bp (unnormal zone <51000 bp) of human reference Y-DNA (NC_000024.10) at resolution of 1 Kbp.

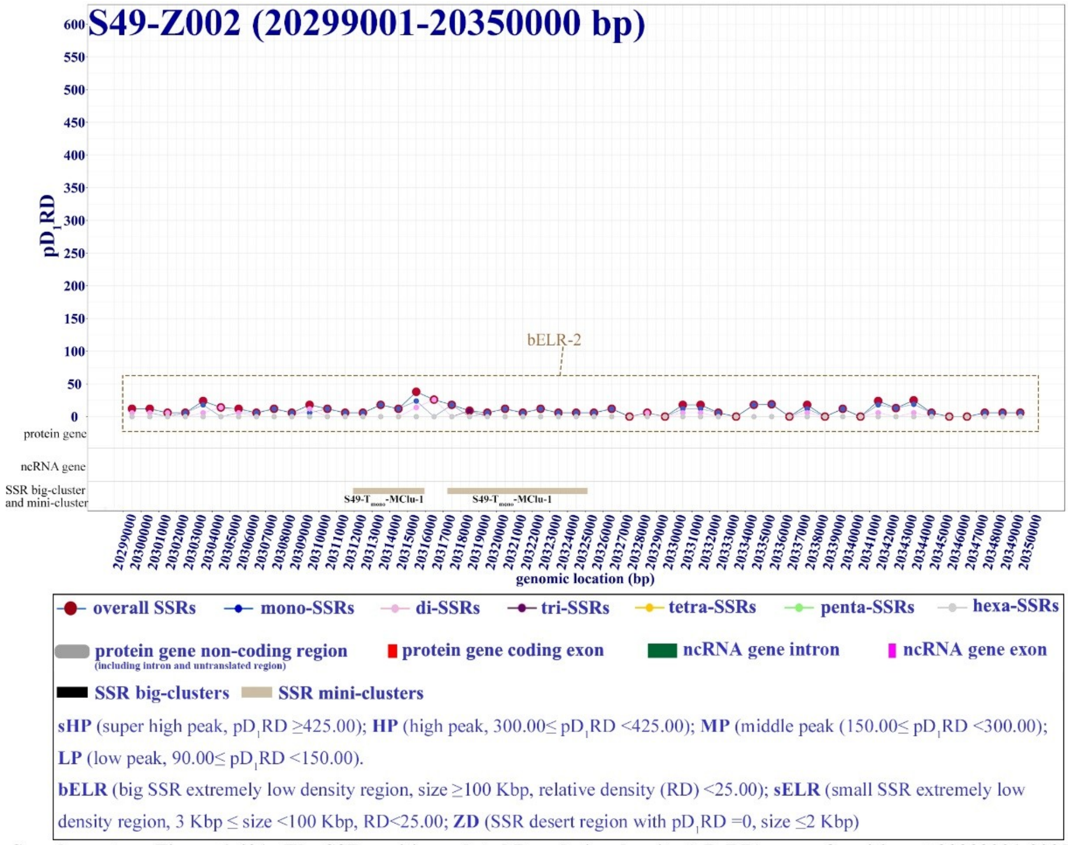

Supplementary Figure 1.401. The SSR position related D_1-relative density (pD_1RD) map of position at 20299001-20350000 bp of human reference Y-DNA (NC_000024.10) at resolution of 1 Kbp.

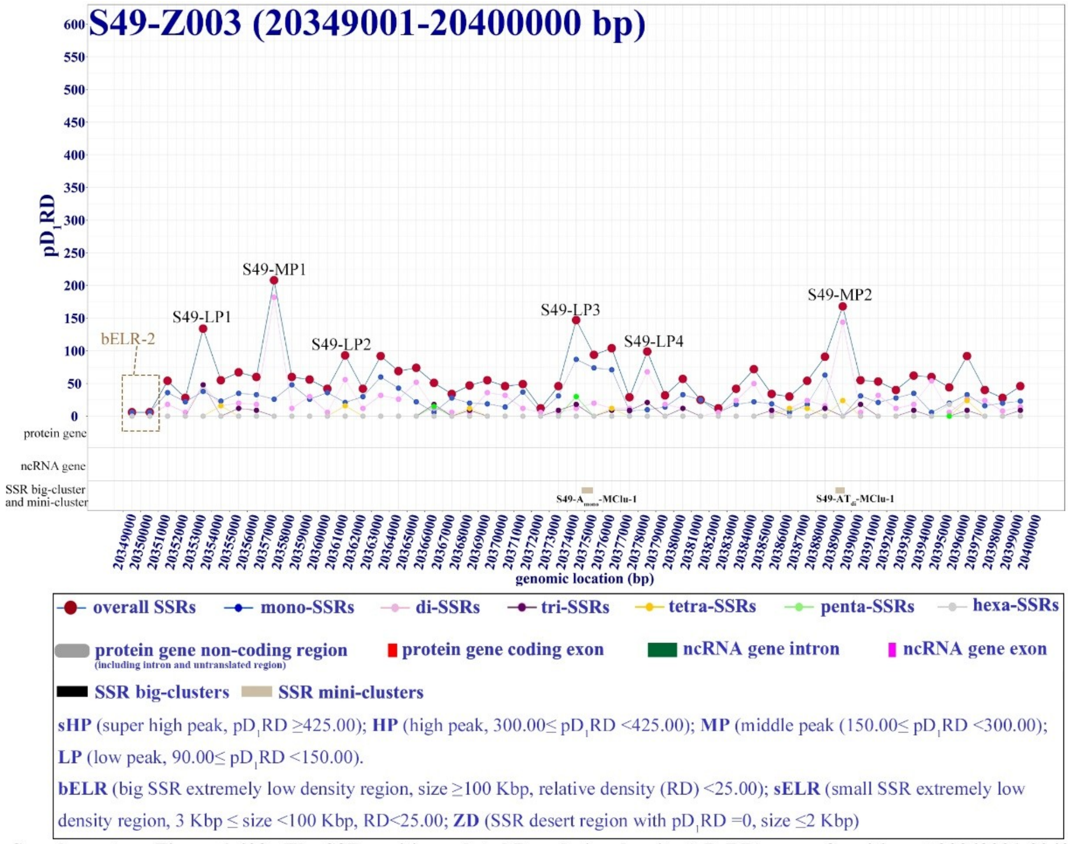

Supplementary Figure 1.402. The SSR position related D_1-relative density (pD_1RD) map of position at 20349001-20400000 bp of human reference Y-DNA (NC_000024.10) at resolution of 1 Kbp.

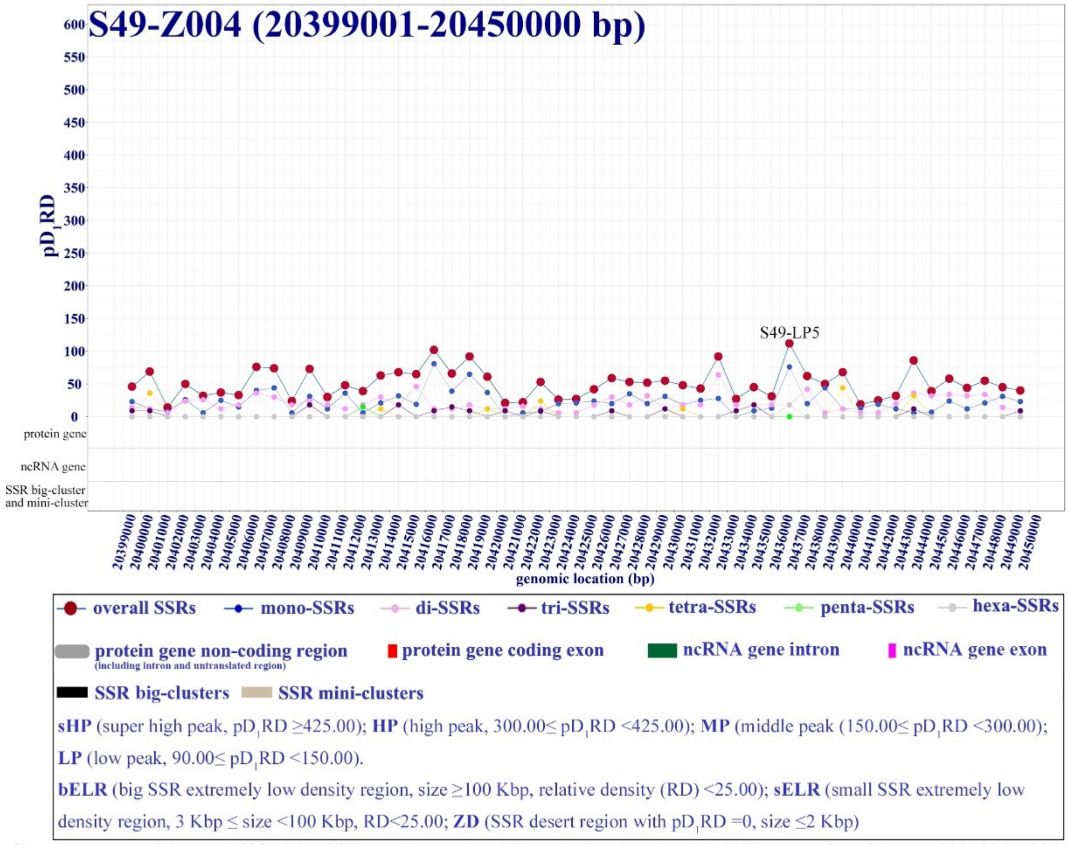

Supplementary Figure 1.403. The SSR position related D_1-relative density (pD_1RD) map of position at 20399001-20450000 bp of human reference Y-DNA (NC_000024.10) at resolution of 1 Kbp.

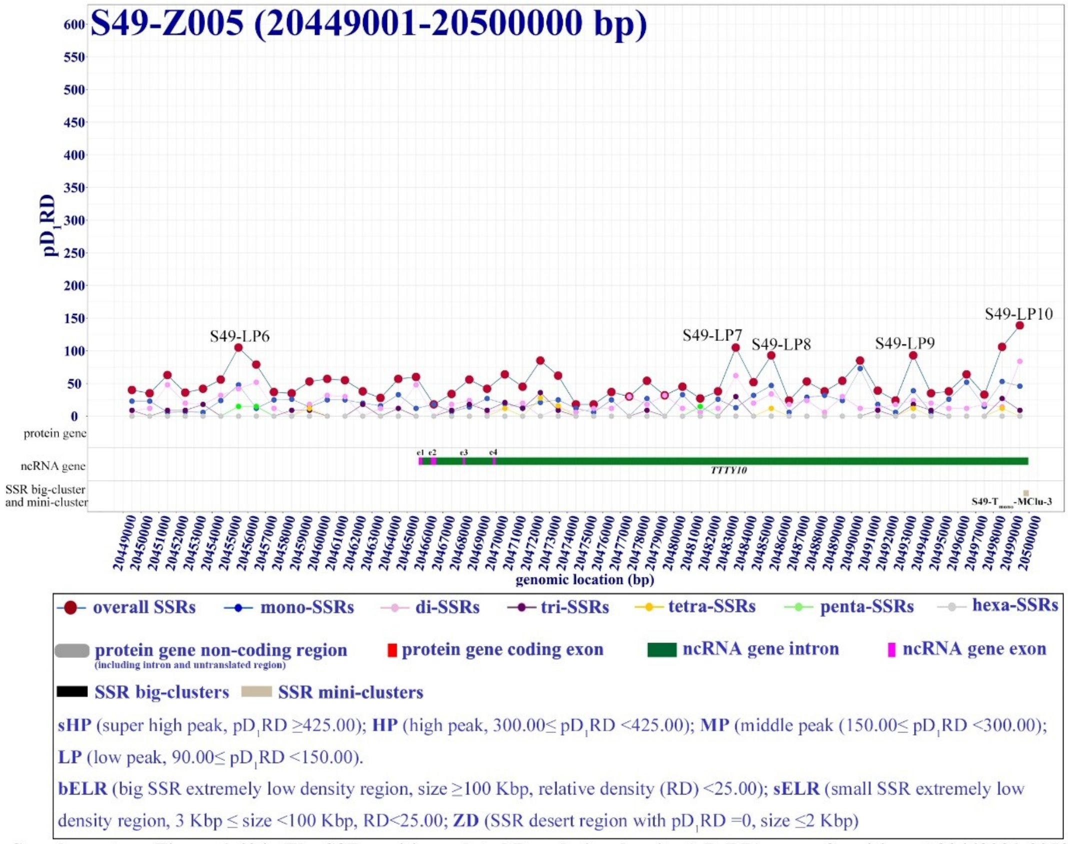

Supplementary Figure 1.404. The SSR position related D_1-relative density (pD_1RD) map of position at 20449001-20500000 bp of human reference Y-DNA (NC_000024.10) at resolution of 1 Kbp.

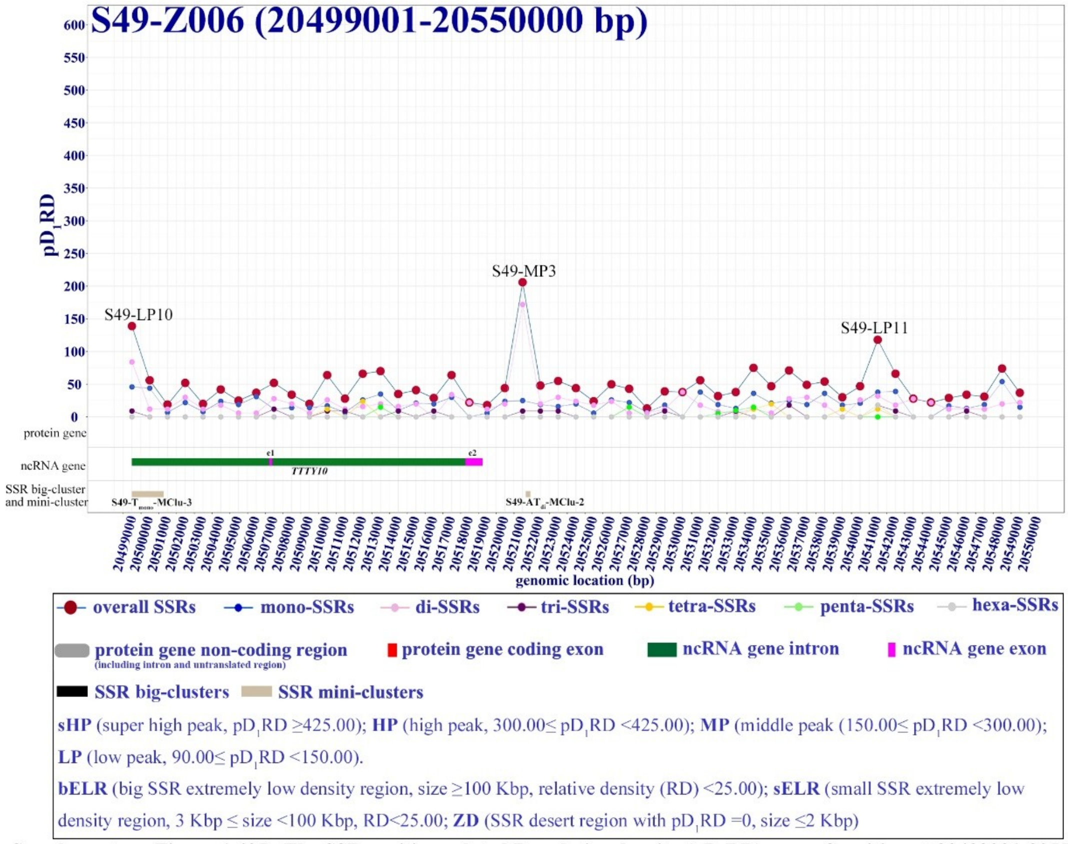

Supplementary Figure 1.405. The SSR position related D_1-relative density (pD_1RD) map of position at 20499001-20550000 bp of human reference Y-DNA (NC_000024.10) at resolution of 1 Kbp.

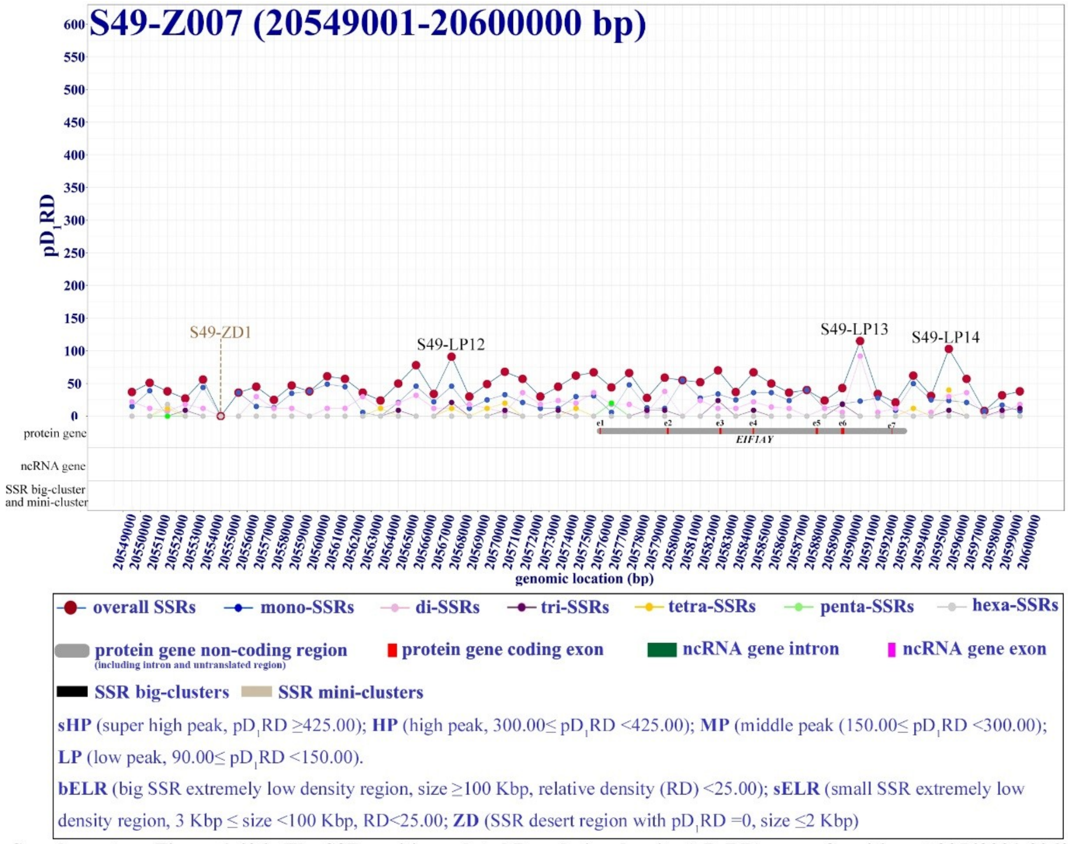

Supplementary Figure 1.406. The SSR position related D_1-relative density (pD_1RD) map of position at 20549001-20600000 bp of human reference Y-DNA (NC_000024.10) at resolution of 1 Kbp.

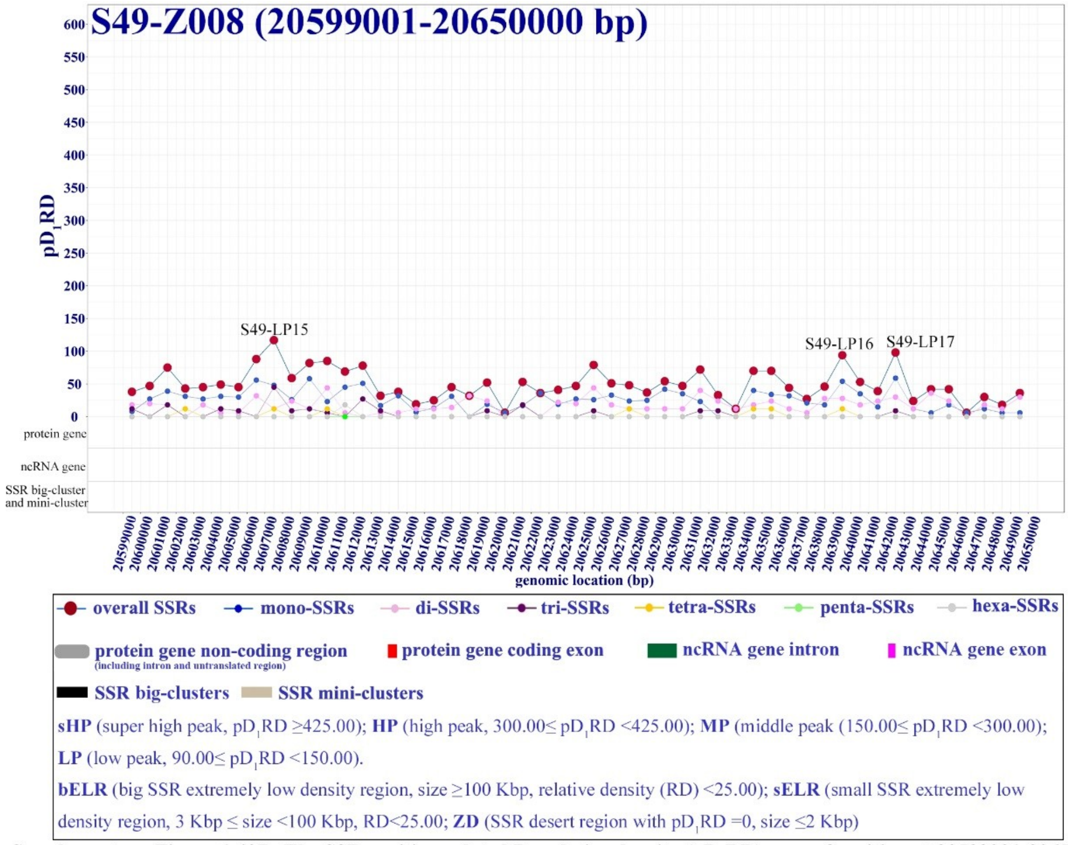

Supplementary Figure 1.407. The SSR position related D_1-relative density (pD_1RD) map of position at 20599001-20650000 bp of human reference Y-DNA (NC_000024.10) at resolution of 1 Kbp.

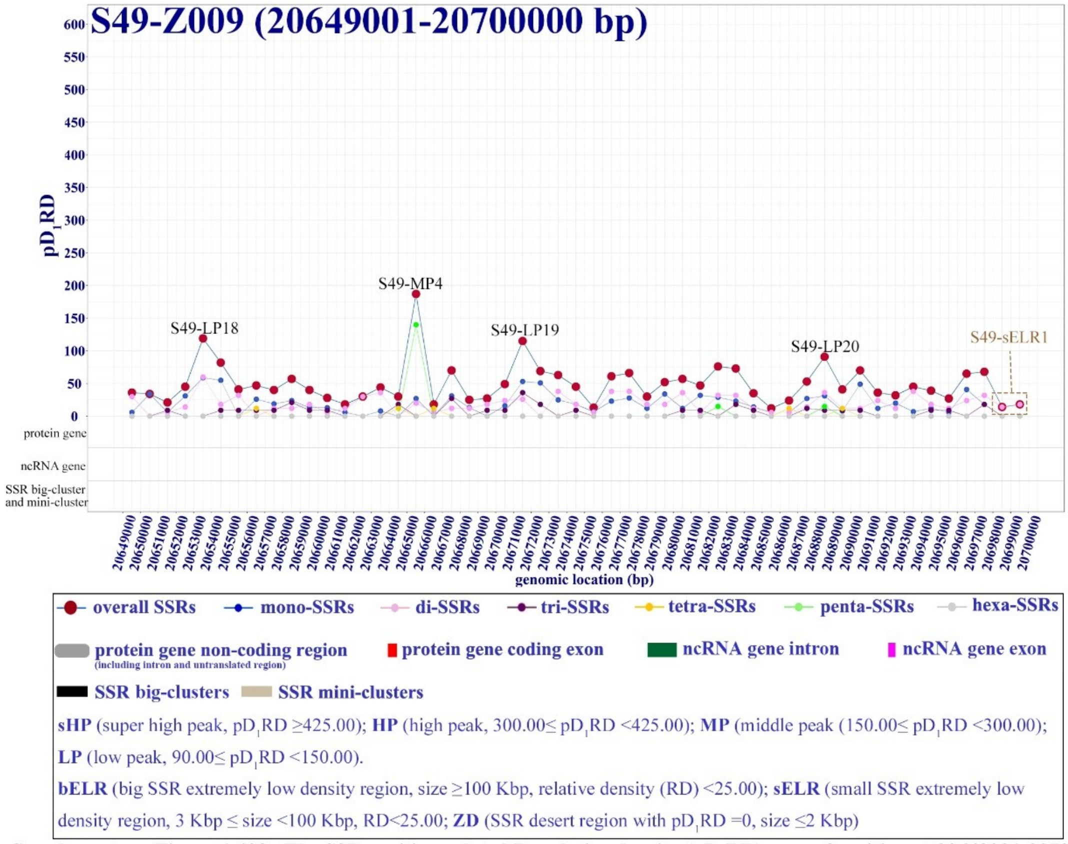

Supplementary Figure 1.408. The SSR position related D_1-relative density (pD_1RD) map of position at 20649001-20700000 bp of human reference Y-DNA (NC_000024.10) at resolution of 1 Kbp.

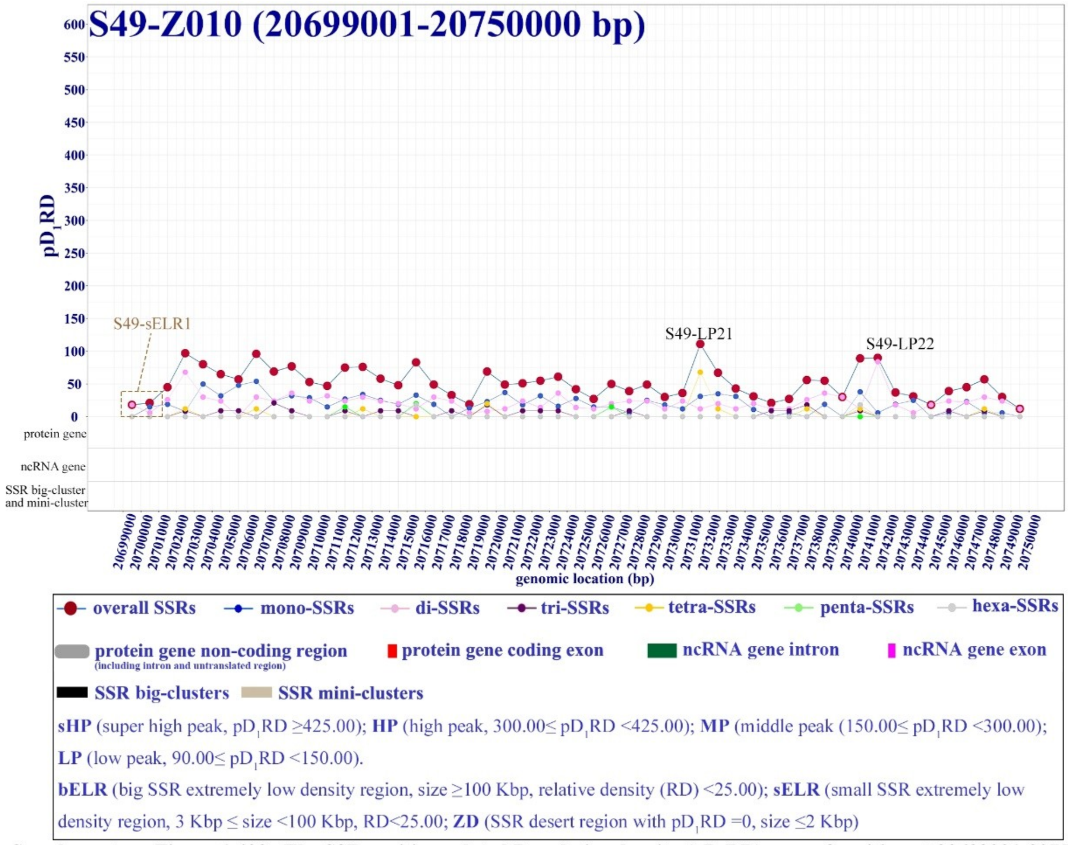

Supplementary Figure 1.409. The SSR position related D_1-relative density (pD_1RD) map of position at 20699001-20750000 bp of human reference Y-DNA (NC_000024.10) at resolution of 1 Kbp.

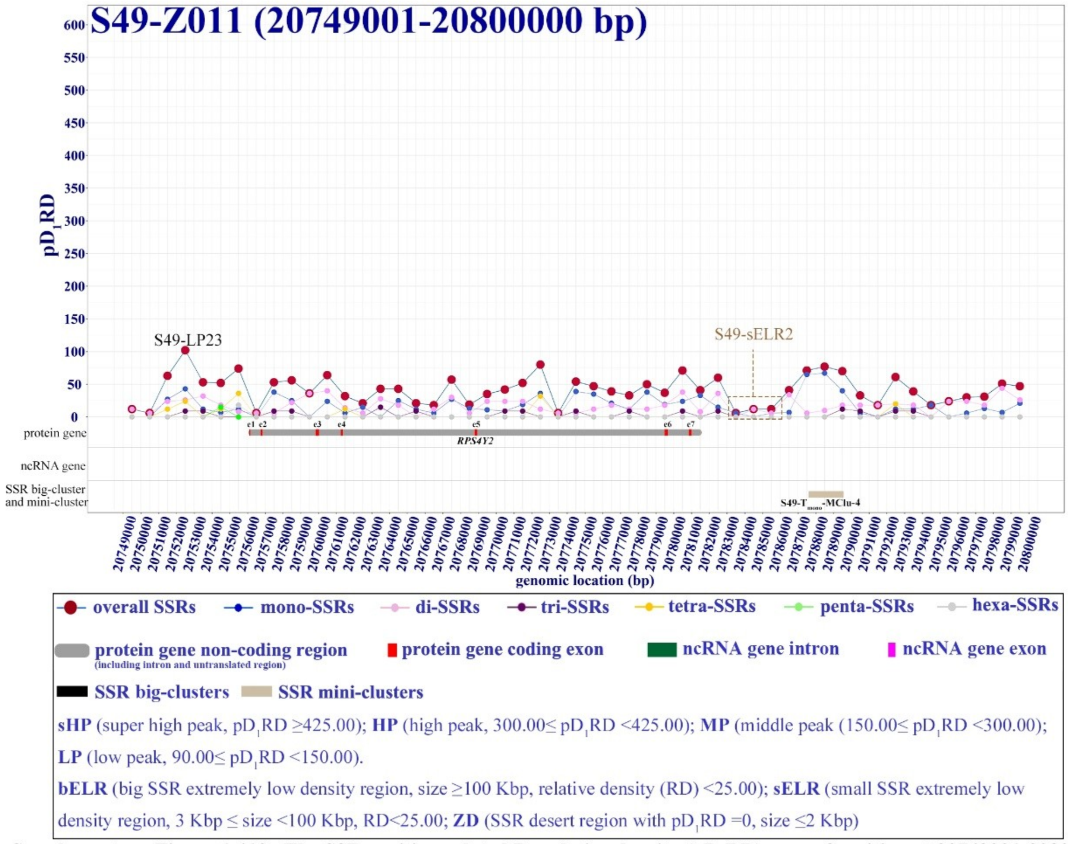

Supplementary Figure 1.410. The SSR position related D_1-relative density (pD_1RD) map of position at 20749001-20800000 bp of human reference Y-DNA (NC_000024.10) at resolution of 1 Kbp.

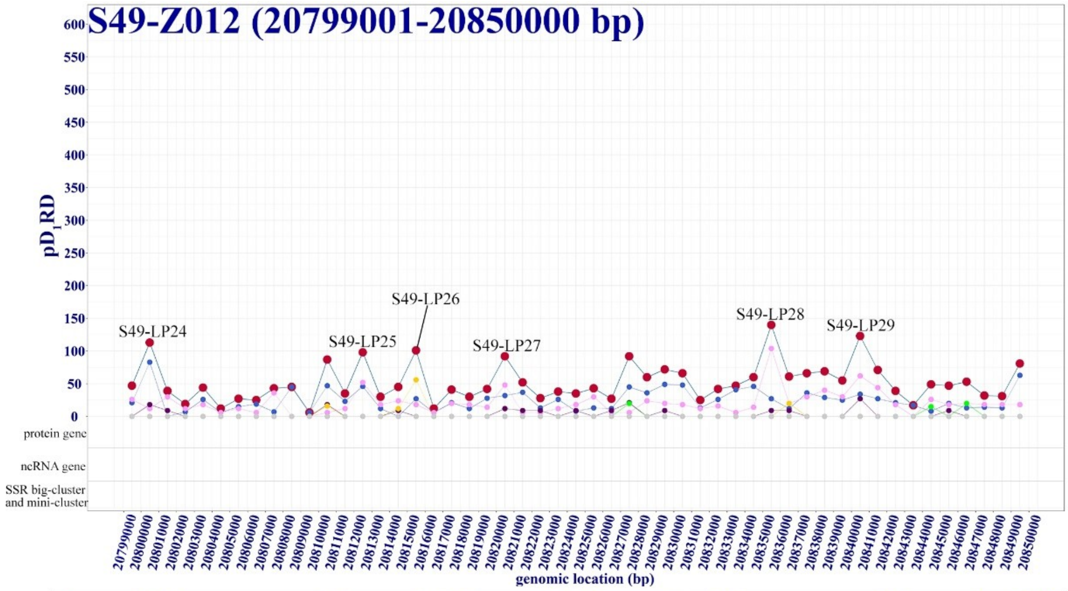

overall SSRs · mono-SSRs · di-SSRs · tri-SSRs · tetra-SSRs · penta-SSRs · hexa-SSRs

protein gene non-coding region (including intron and untranslated region) · protein gene coding exon · ncRNA gene intron · ncRNA gene exon

SSR big-clusters · SSR mini-clusters

sHP (super high peak, $pD_1RD \geq 425.00$); **HP** (high peak, $300.00 \leq pD_1RD < 425.00$); **MP** (middle peak ($150.00 \leq pD_1RD < 300.00$); **LP** (low peak, $90.00 \leq pD_1RD < 150.00$).

bELR (big SSR extremely low density region, size ≥100 Kbp, relative density (RD) <25.00); **sELR** (small SSR extremely low density region, 3 Kbp ≤ size <100 Kbp, RD<25.00; **ZD** (SSR desert region with pD_1RD =0, size ≤2 Kbp)

Supplementary Figure 1.411. The SSR position related D_1-relative density (pD_1RD) map of position at 20799001-20850000 bp of human reference Y-DNA (NC_000024.10) at resolution of 1 Kbp.

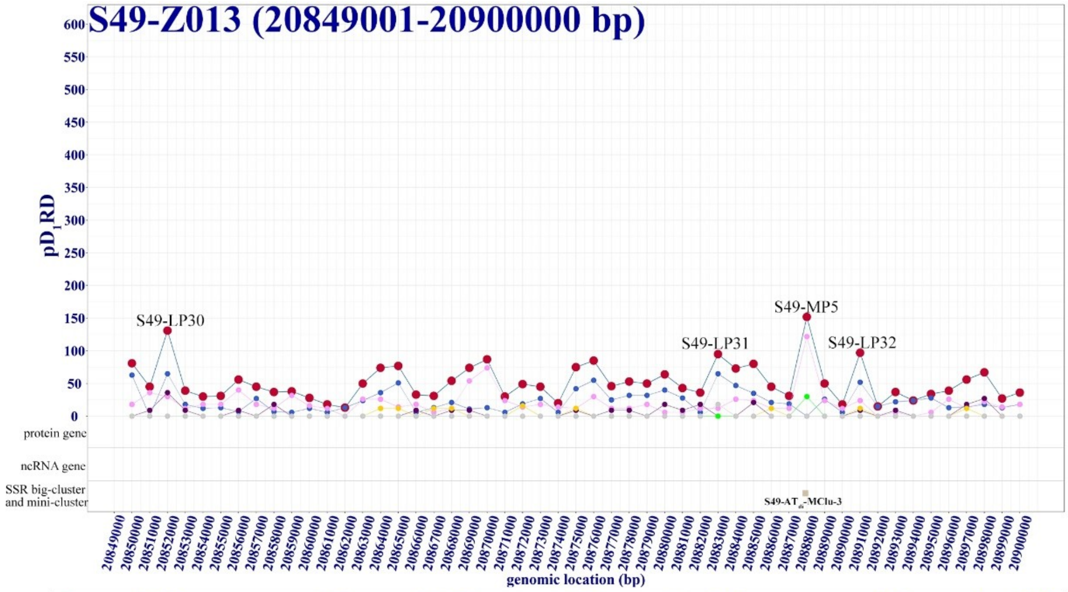

overall SSRs · mono-SSRs · di-SSRs · tri-SSRs · tetra-SSRs · penta-SSRs · hexa-SSRs

protein gene non-coding region (including intron and untranslated region) · protein gene coding exon · ncRNA gene intron · ncRNA gene exon

SSR big-clusters · SSR mini-clusters

sHP (super high peak, $pD_1RD \geq 425.00$); **HP** (high peak, $300.00 \leq pD_1RD < 425.00$); **MP** (middle peak ($150.00 \leq pD_1RD < 300.00$); **LP** (low peak, $90.00 \leq pD_1RD < 150.00$).

bELR (big SSR extremely low density region, size ≥100 Kbp, relative density (RD) <25.00); **sELR** (small SSR extremely low density region, 3 Kbp ≤ size <100 Kbp, RD<25.00; **ZD** (SSR desert region with pD_1RD =0, size ≤2 Kbp)

Supplementary Figure 1.412. The SSR position related D_1-relative density (pD_1RD) map of position at 20849001-20900000 bp of human reference Y-DNA (NC_000024.10) at resolution of 1 Kbp.

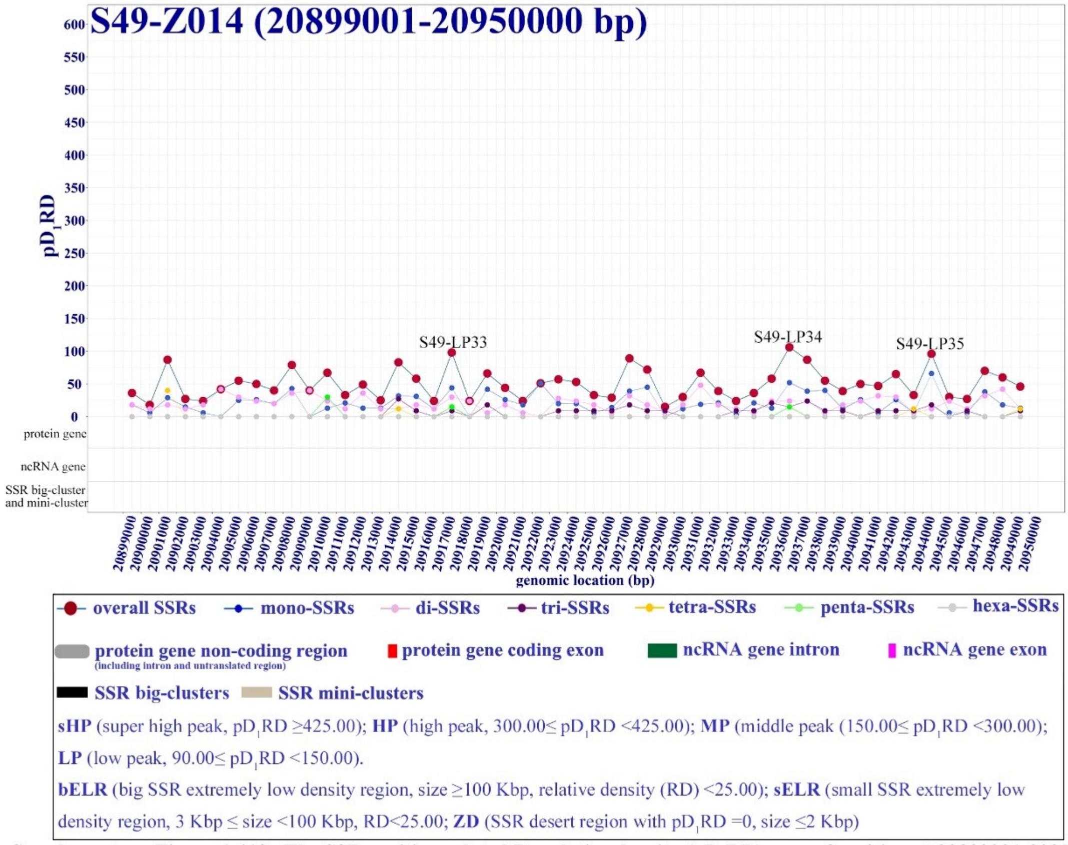

Supplementary Figure 1.413. The SSR position related D_1-relative density (pD_1RD) map of position at 20899001-20950000 bp of human reference Y-DNA (NC_000024.10) at resolution of 1 Kbp.

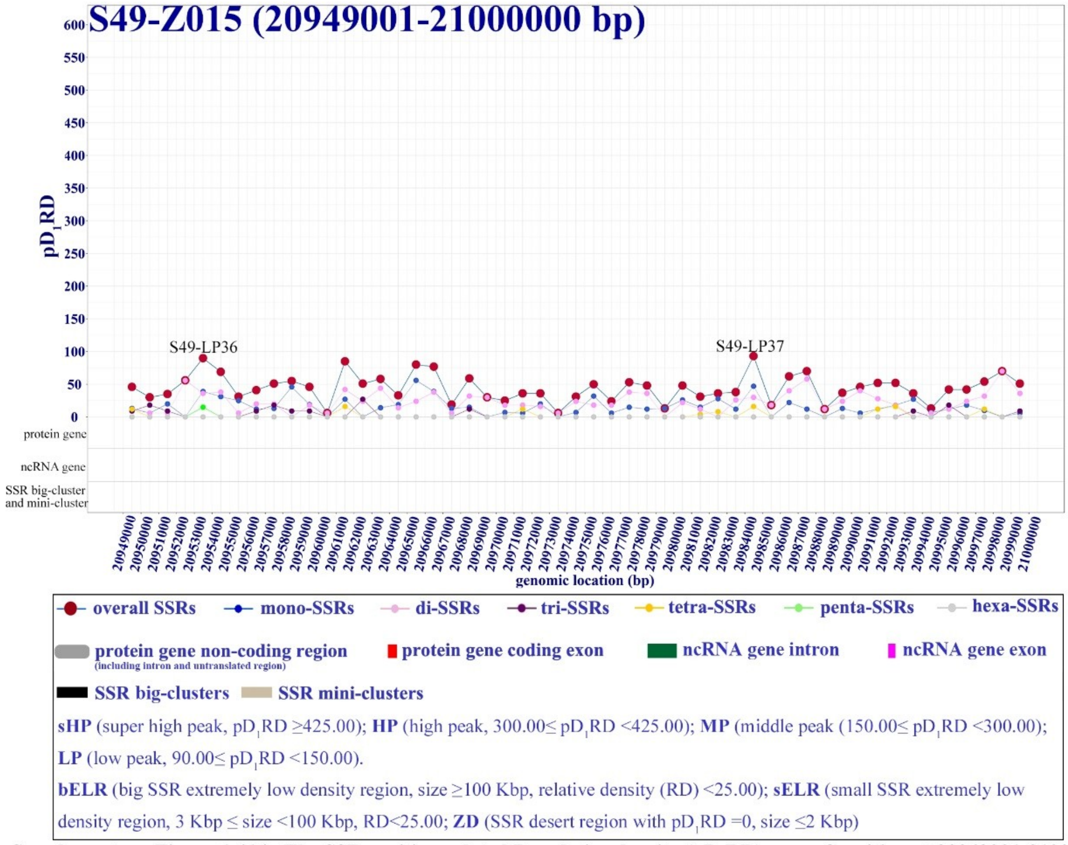

Supplementary Figure 1.414. The SSR position related D_1-relative density (pD_1RD) map of position at 20949001-21000000 bp of human reference Y-DNA (NC_000024.10) at resolution of 1 Kbp.

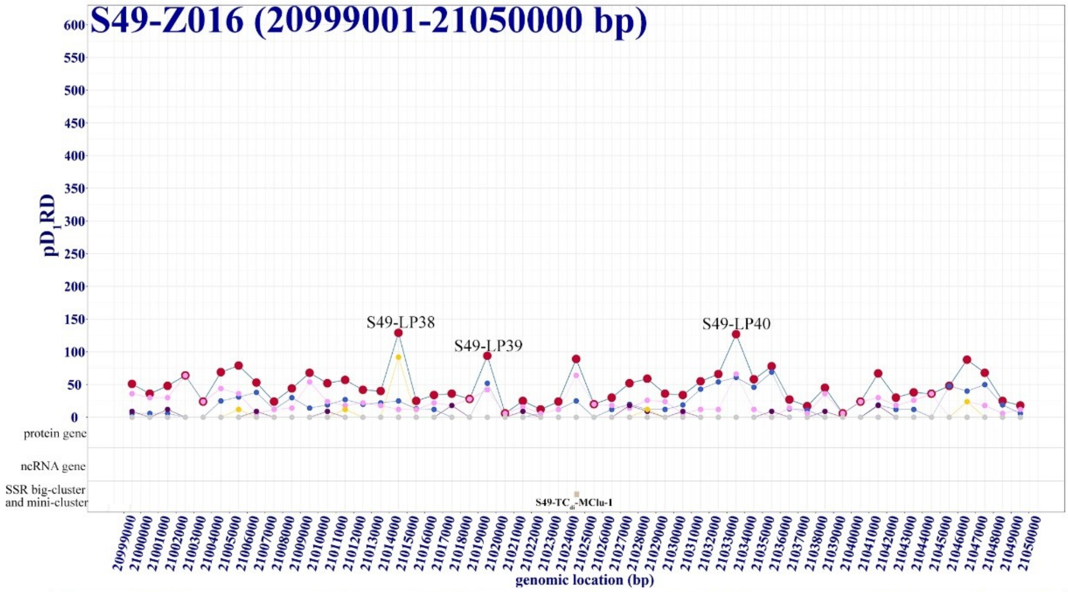

overall SSRs · mono-SSRs · di-SSRs · tri-SSRs · tetra-SSRs · penta-SSRs · hexa-SSRs

protein gene non-coding region (including intron and untranslated region) · protein gene coding exon · ncRNA gene intron · ncRNA gene exon

SSR big-clusters · SSR mini-clusters

sHP (super high peak, $pD_1RD \geq 425.00$); **HP** (high peak, $300.00 \leq pD_1RD < 425.00$); **MP** (middle peak ($150.00 \leq pD_1RD < 300.00$); **LP** (low peak, $90.00 \leq pD_1RD < 150.00$).

bELR (big SSR extremely low density region, size ≥100 Kbp, relative density (RD) <25.00); **sELR** (small SSR extremely low density region, 3 Kbp ≤ size <100 Kbp, RD<25.00; **ZD** (SSR desert region with $pD_1RD = 0$, size ≤2 Kbp)

Supplementary Figure 1.415. The SSR position related D_1-relative density (pD_1RD) map of position at 20999001-21050000 bp of human reference Y-DNA (NC_000024.10) at resolution of 1 Kbp.

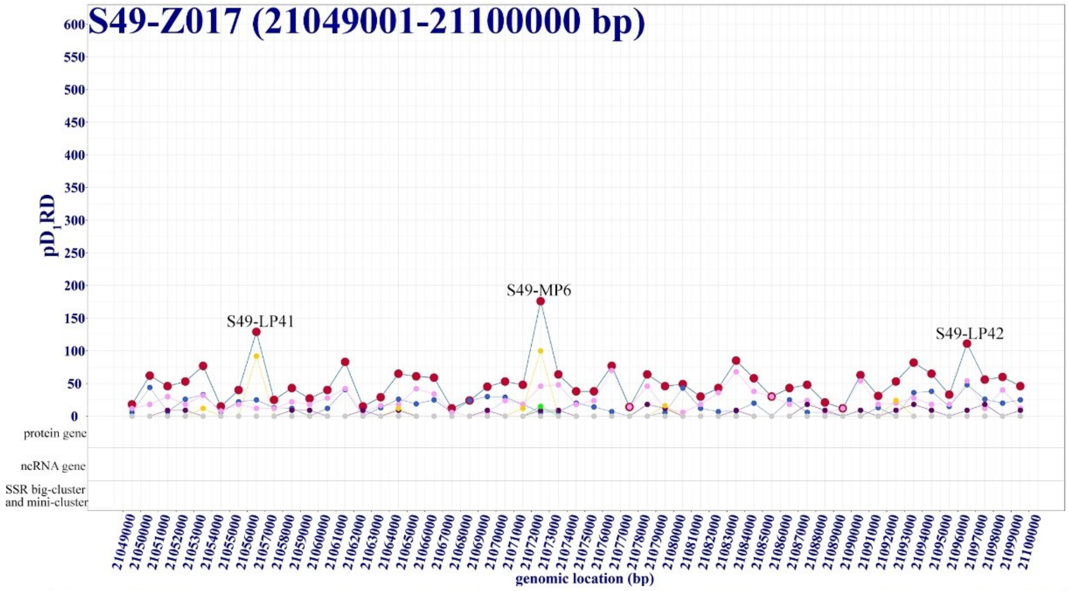

overall SSRs · mono-SSRs · di-SSRs · tri-SSRs · tetra-SSRs · penta-SSRs · hexa-SSRs

protein gene non-coding region (including intron and untranslated region) · protein gene coding exon · ncRNA gene intron · ncRNA gene exon

SSR big-clusters · SSR mini-clusters

sHP (super high peak, $pD_1RD \geq 425.00$); **HP** (high peak, $300.00 \leq pD_1RD < 425.00$); **MP** (middle peak ($150.00 \leq pD_1RD < 300.00$); **LP** (low peak, $90.00 \leq pD_1RD < 150.00$).

bELR (big SSR extremely low density region, size ≥100 Kbp, relative density (RD) <25.00); **sELR** (small SSR extremely low density region, 3 Kbp ≤ size <100 Kbp, RD<25.00; **ZD** (SSR desert region with $pD_1RD = 0$, size ≤2 Kbp)

Supplementary Figure 1.416. The SSR position related D_1-relative density (pD_1RD) map of position at 21049001-21100000 bp of human reference Y-DNA (NC_000024.10) at resolution of 1 Kbp.

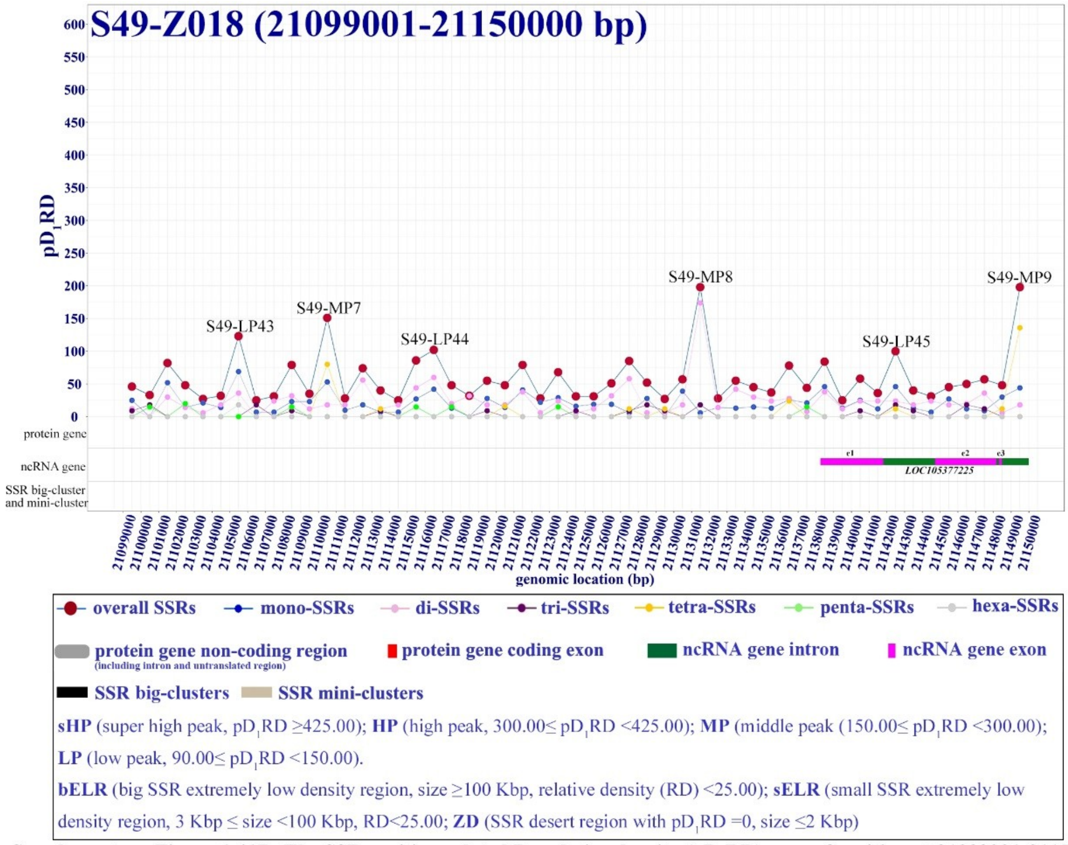

Supplementary Figure 1.417. The SSR position related D_1-relative density (pD_1RD) map of position at 21099001-21150000 bp of human reference Y-DNA (NC_000024.10) at resolution of 1 Kbp.

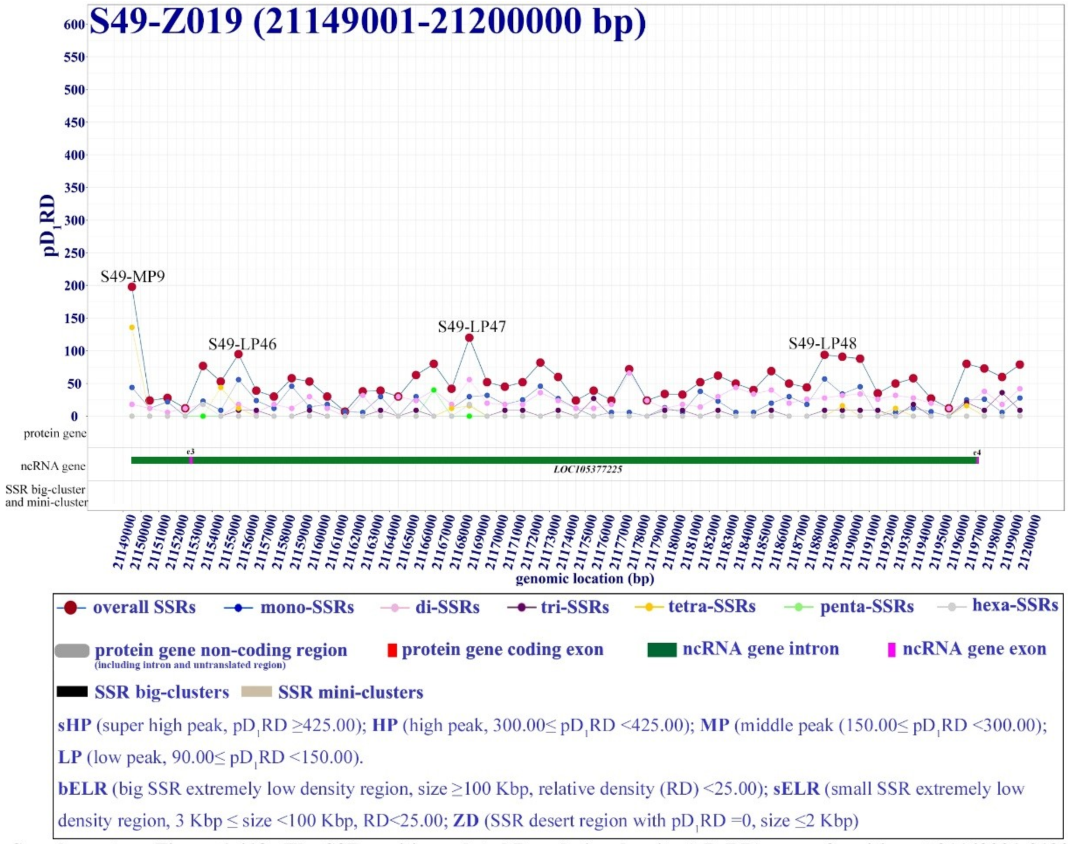

Supplementary Figure 1.418. The SSR position related D_1-relative density (pD_1RD) map of position at 21149001-21200000 bp of human reference Y-DNA (NC_000024.10) at resolution of 1 Kbp.

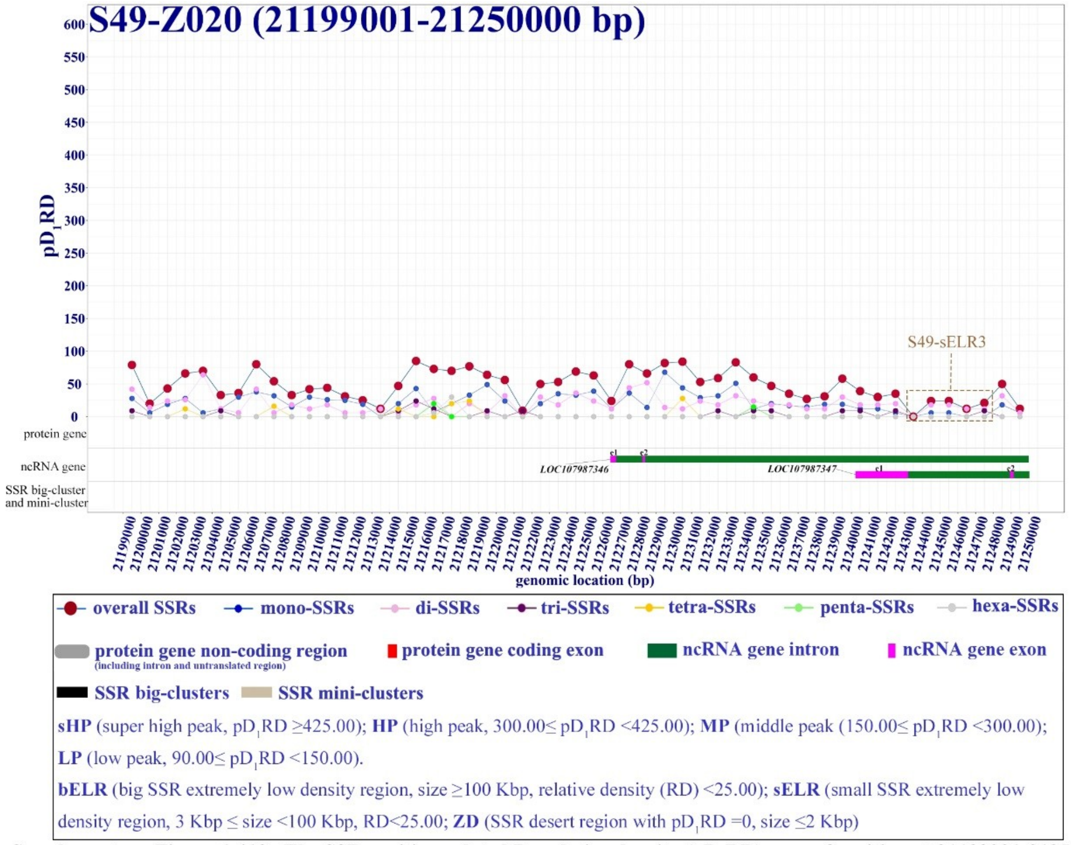

Supplementary Figure 1.419. The SSR position related D_1-relative density (pD_1RD) map of position at 21199001-21250000 bp of human reference Y-DNA (NC_000024.10) at resolution of 1 Kbp.

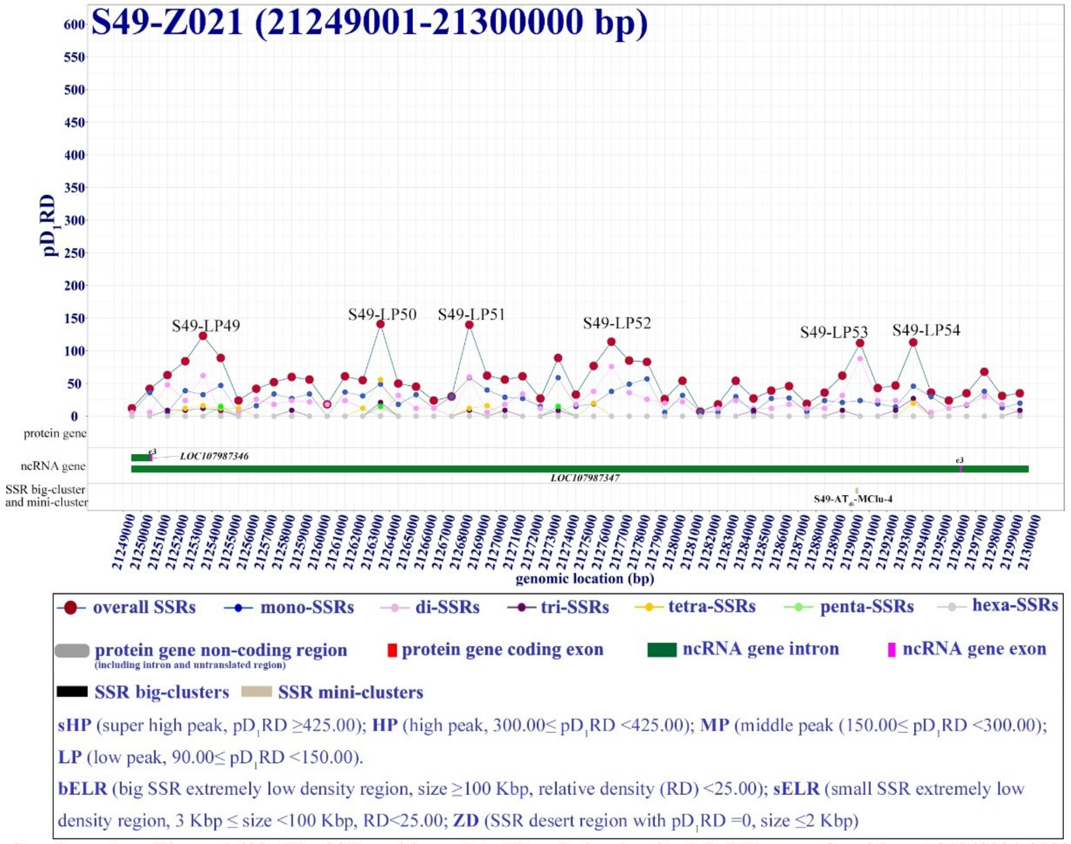

Supplementary Figure 1.420. The SSR position related D_1-relative density (pD_1RD) map of position at 21249001-21300000 bp of human reference Y-DNA (NC_000024.10) at resolution of 1 Kbp.

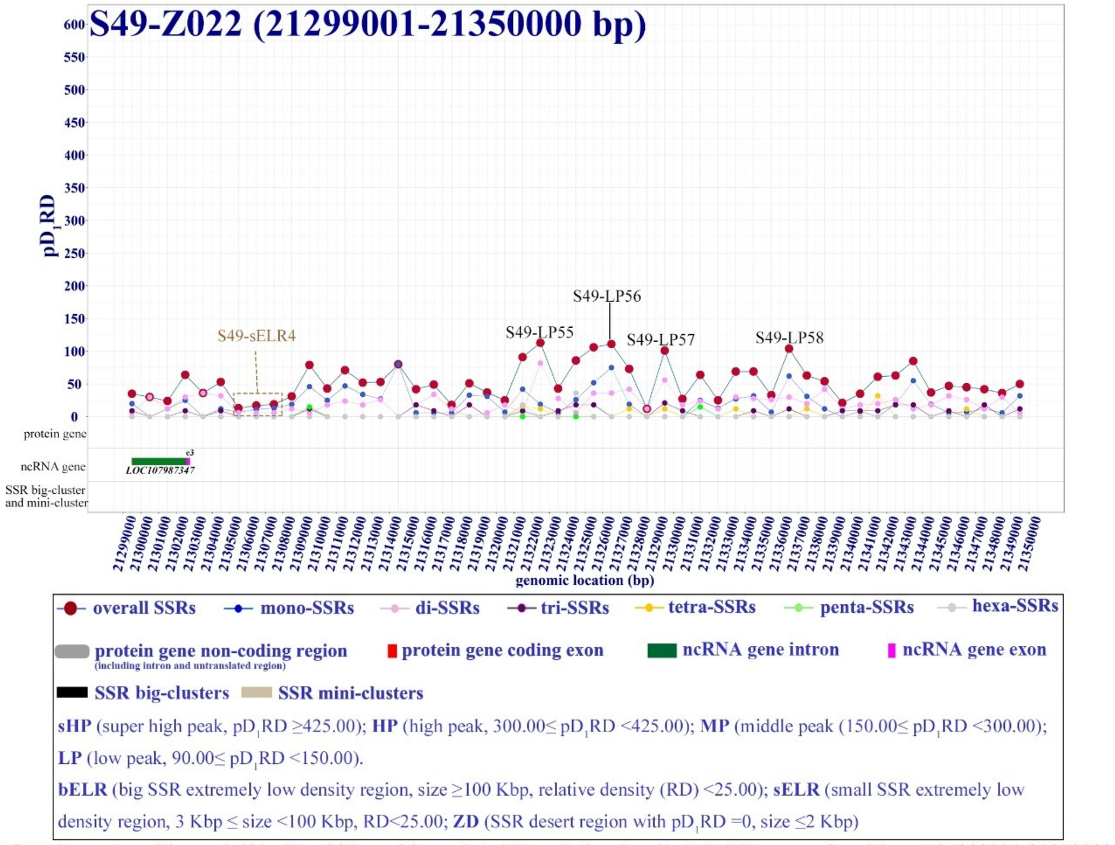

Supplementary Figure 1.421. The SSR position related D_1-relative density (pD_1RD) map of position at 21299001-21350000 bp of human reference Y-DNA (NC_000024.10) at resolution of 1 Kbp.

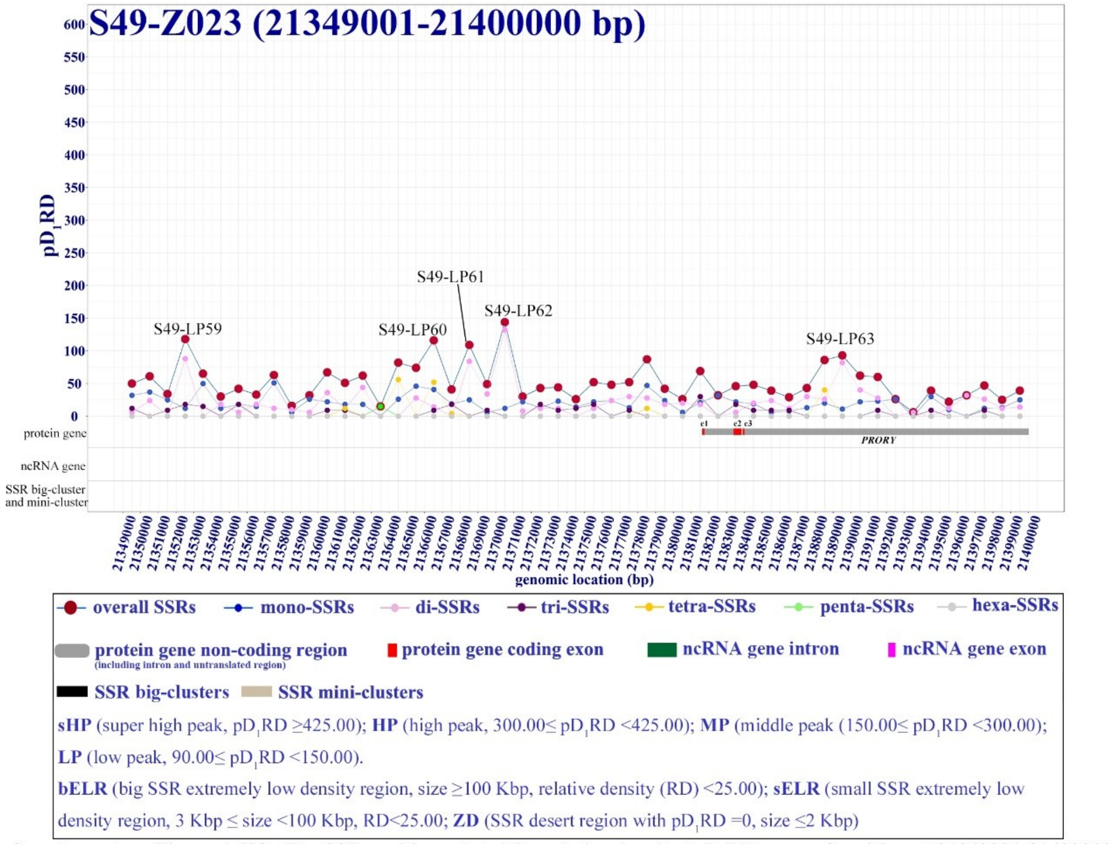

Supplementary Figure 1.422. The SSR position related D_1-relative density (pD_1RD) map of position at 21349001-21400000 bp of human reference Y-DNA (NC_000024.10) at resolution of 1 Kbp.

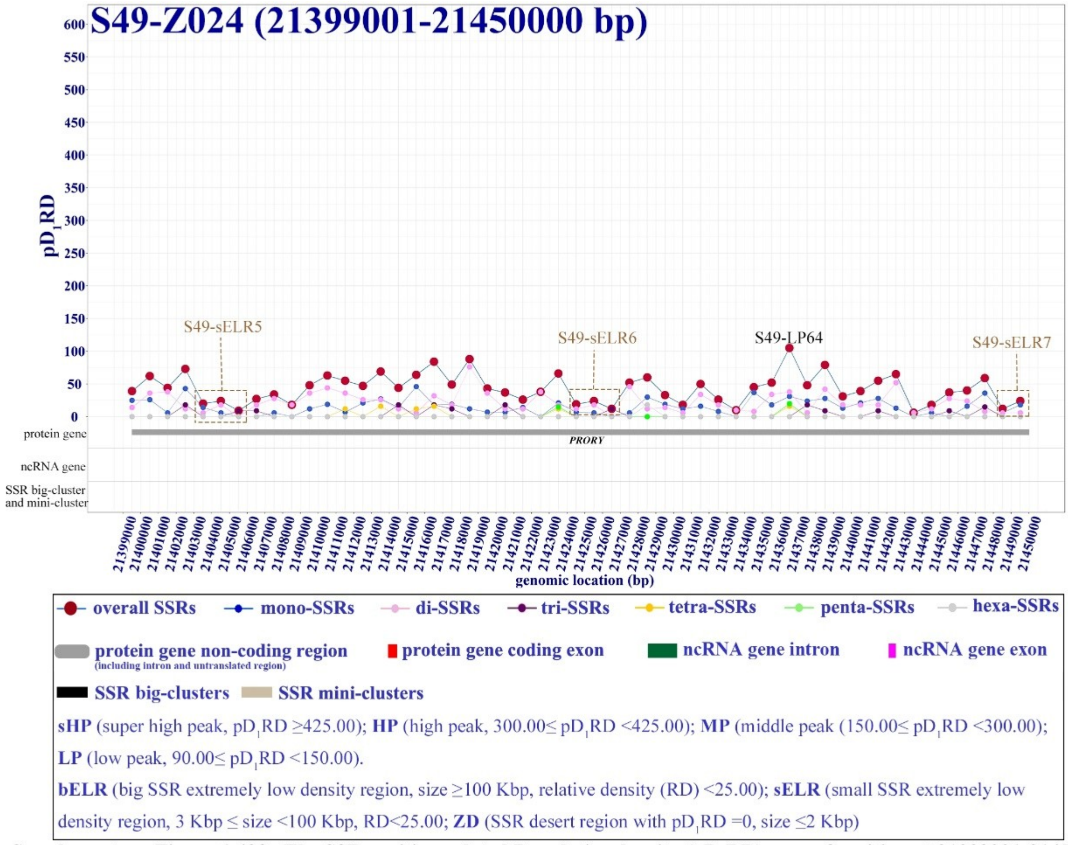

Supplementary Figure 1.423. The SSR position related D_1-relative density (pD_1RD) map of position at 21399001-21450000 bp of human reference Y-DNA (NC_000024.10) at resolution of 1 Kbp.

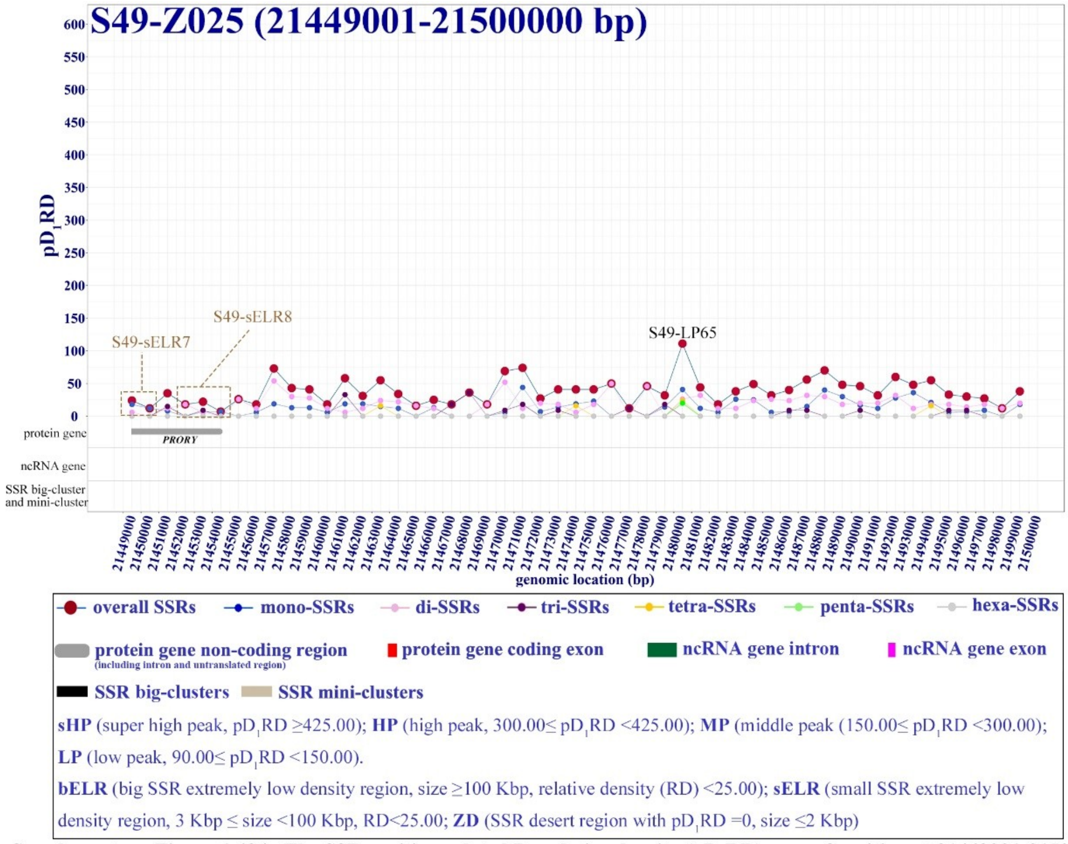

Supplementary Figure 1.424. The SSR position related D_1-relative density (pD_1RD) map of position at 21449001-21500000 bp of human reference Y-DNA (NC_000024.10) at resolution of 1 Kbp.

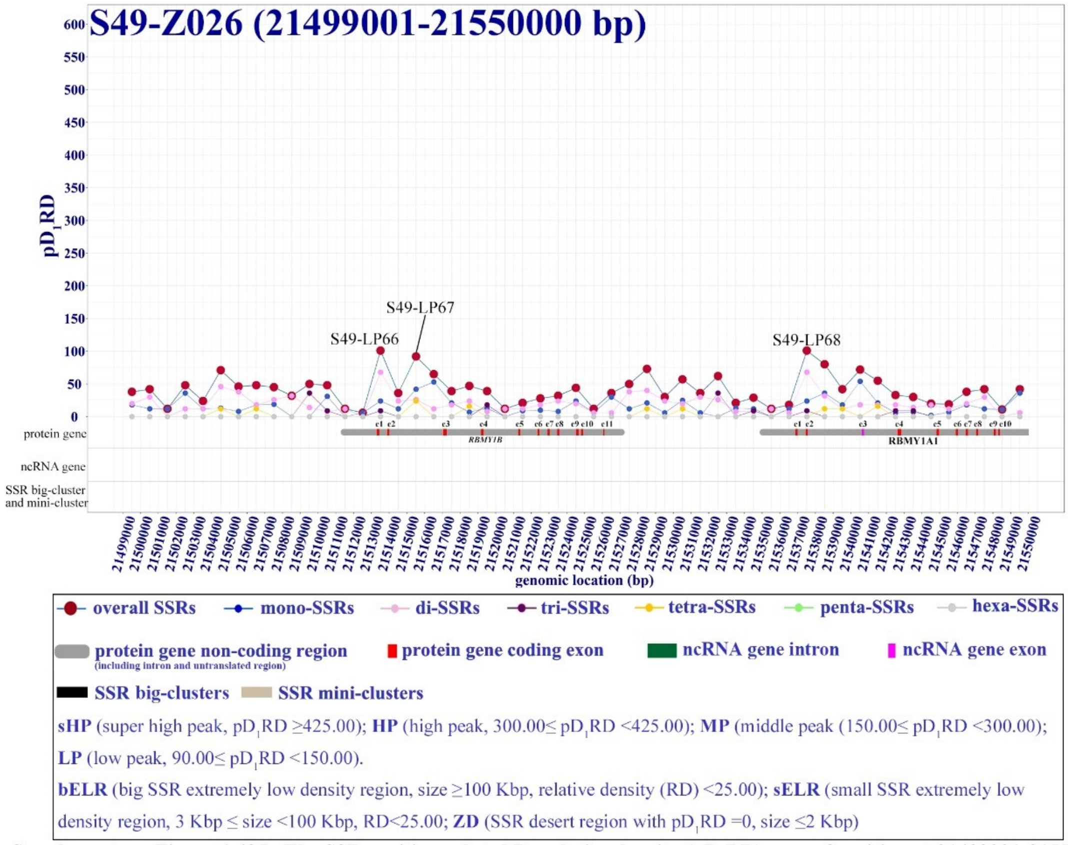

Supplementary Figure 1.425. The SSR position related D_1-relative density (pD_1RD) map of position at 21499001-21550000 bp of human reference Y-DNA (NC_000024.10) at resolution of 1 Kbp.

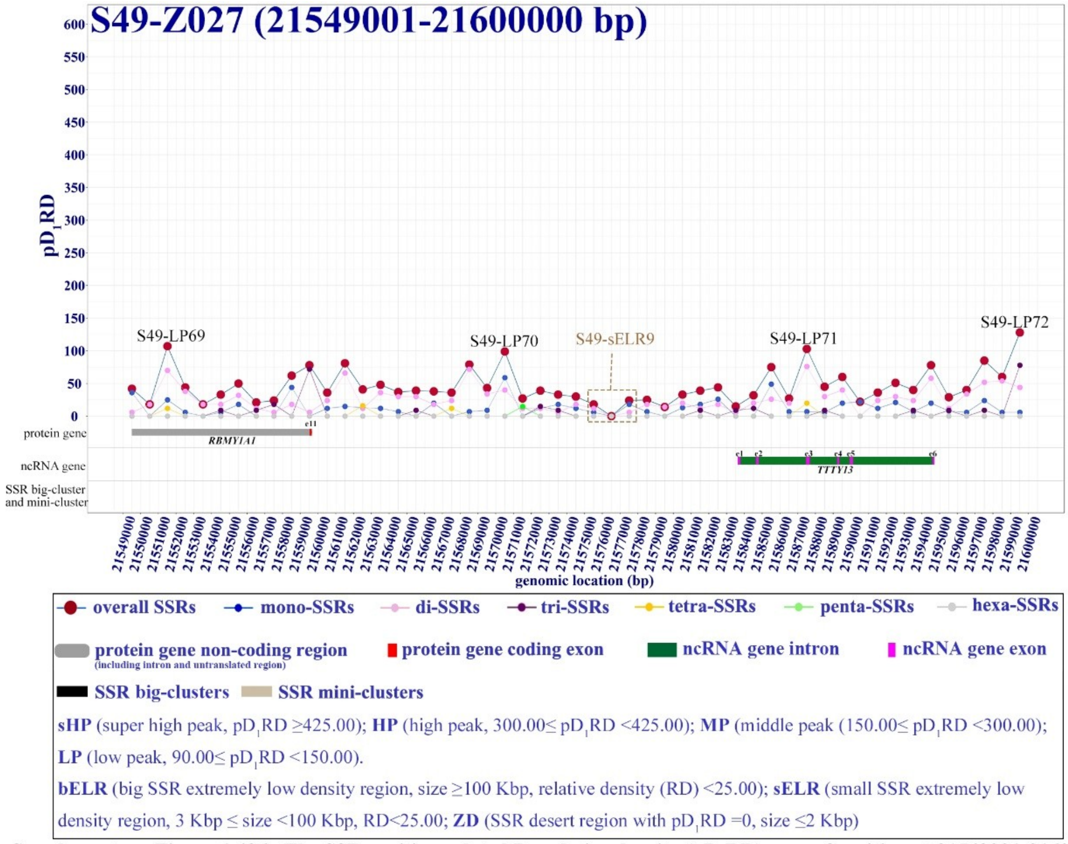

Supplementary Figure 1.426. The SSR position related D_1-relative density (pD_1RD) map of position at 21549001-21600000 bp of human reference Y-DNA (NC_000024.10) at resolution of 1 Kbp.

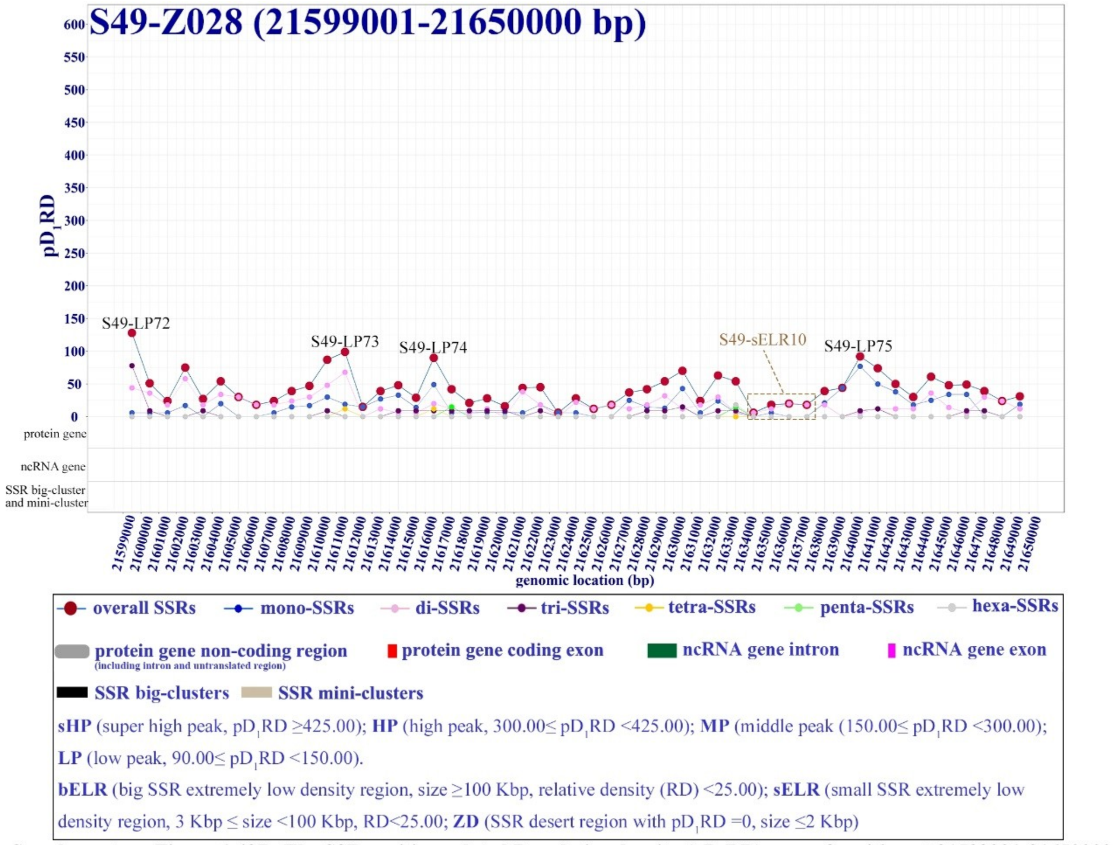

Supplementary Figure 1.427. The SSR position related D_1-relative density (pD_1RD) map of position at 21599001-21650000 bp of human reference Y-DNA (NC_000024.10) at resolution of 1 Kbp.

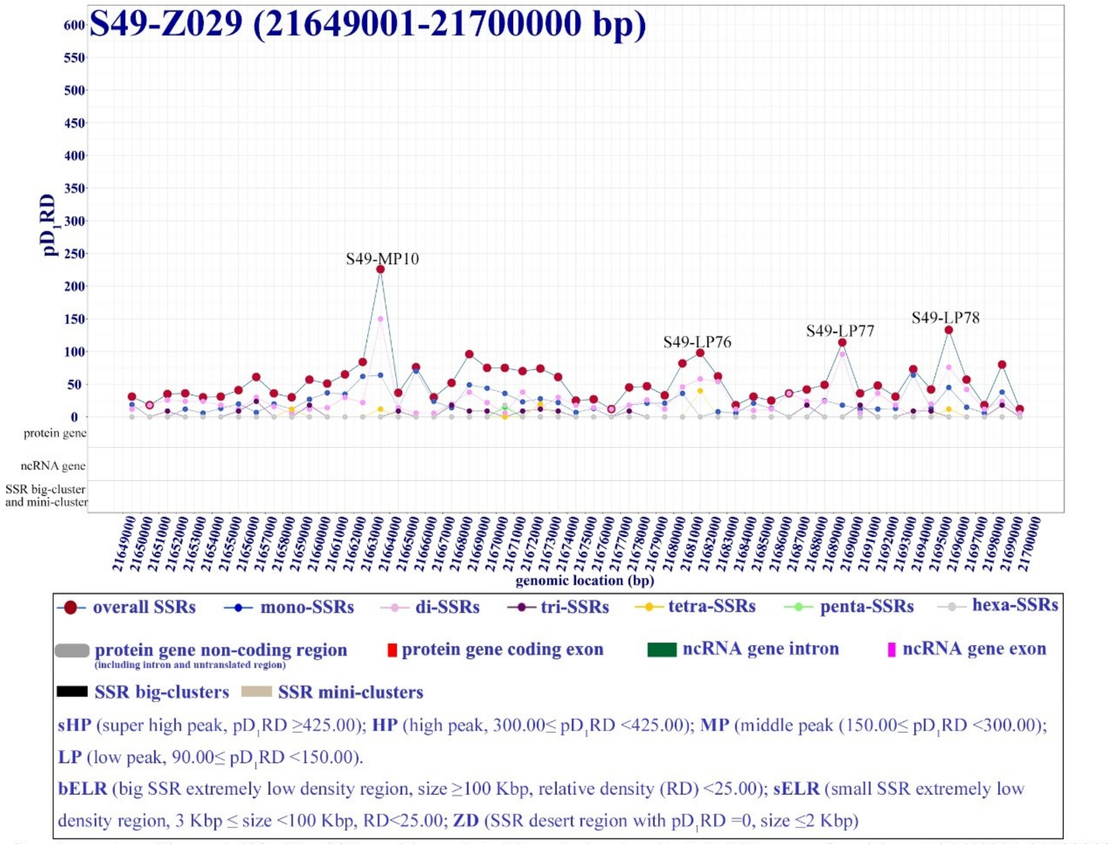

Supplementary Figure 1.428. The SSR position related D_1-relative density (pD_1RD) map of position at 21649001-21700000 bp of human reference Y-DNA (NC_000024.10) at resolution of 1 Kbp.

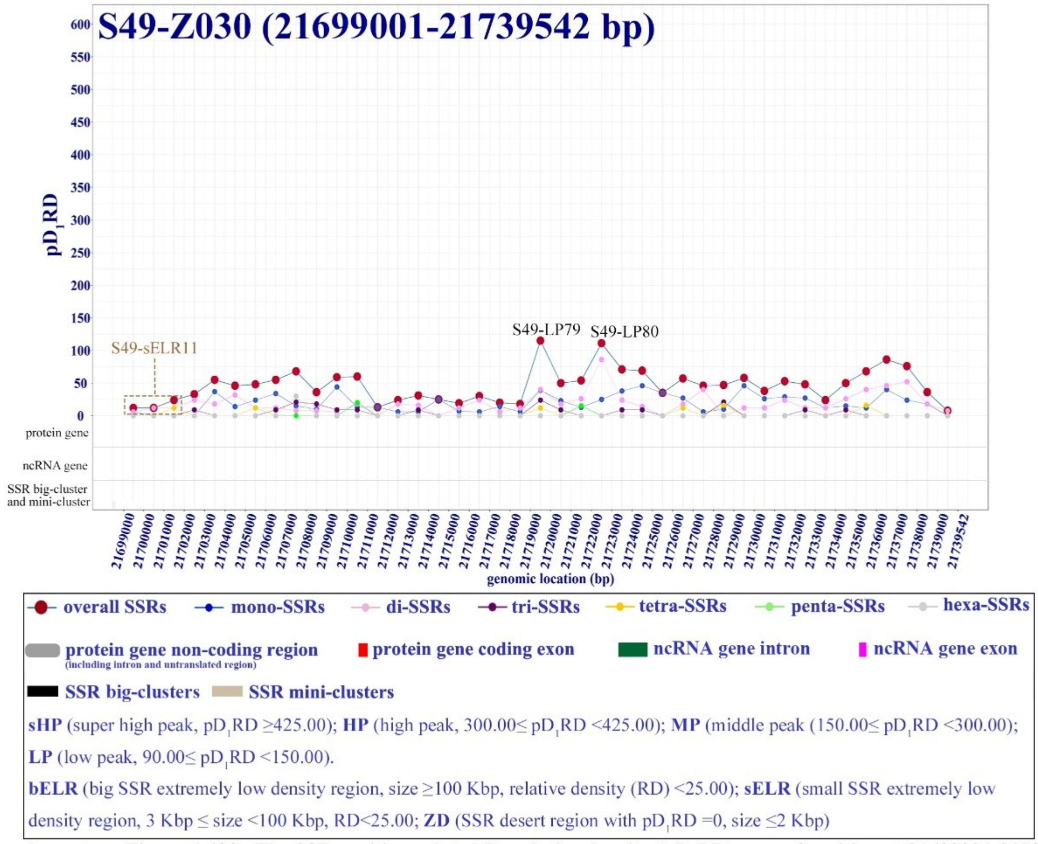

Supplementary Figure 1.429. The SSR position related D_1-relative density (pD_1RD) map of position at 21699001-21739542 bp of human reference Y-DNA (NC_000024.10) at resolution of 1 Kbp.

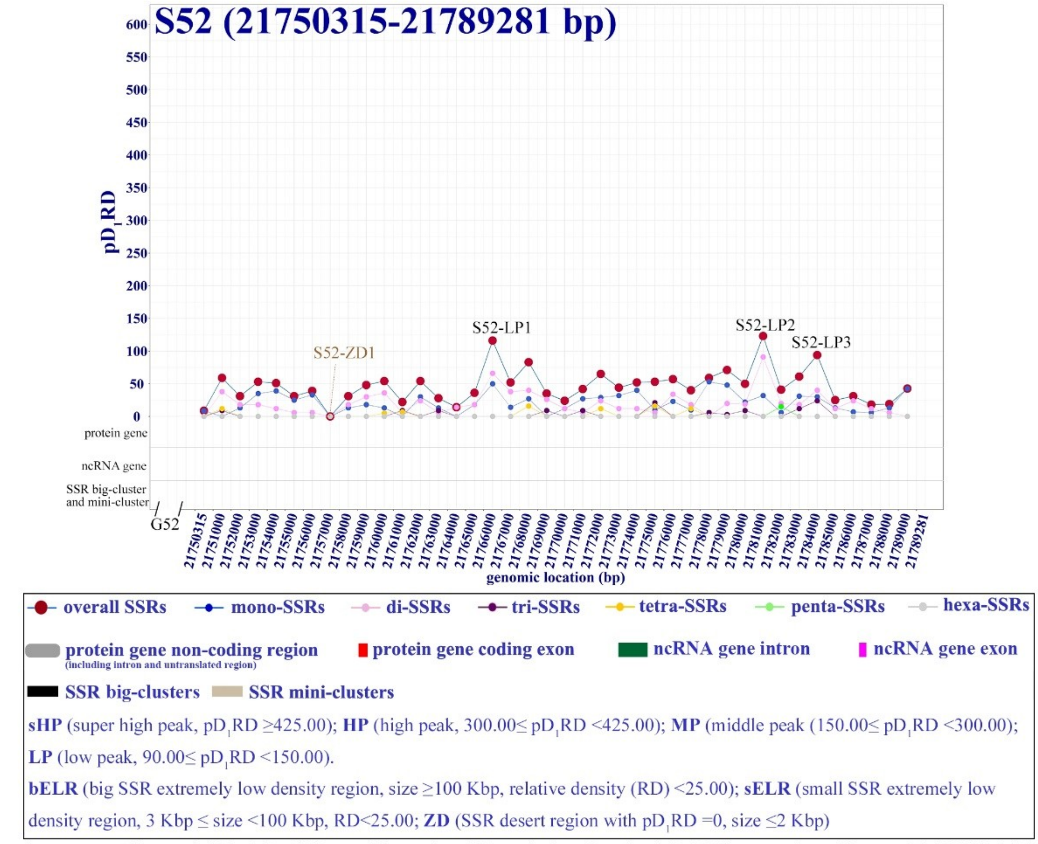

Supplementary Figure 1.430. The SSR position related D_1-relative density (pD_1RD) map of position at 21750315-21789281 bp of human reference Y-DNA (NC_000024.10) at resolution of 1 Kbp.

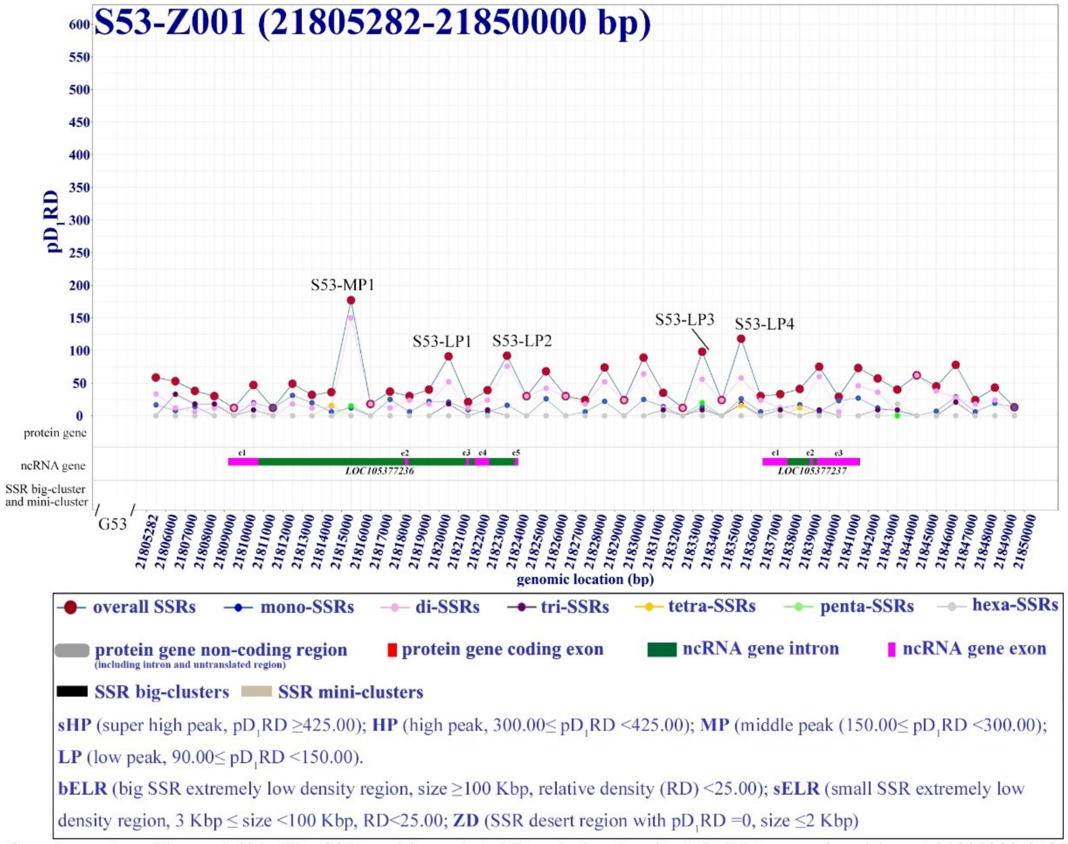

Supplementary Figure 1.431. The SSR position related D_1-relative density (pD_1RD) map of position at 21805282-21850000 bp of human reference Y-DNA (NC_000024.10) at resolution of 1 Kbp.

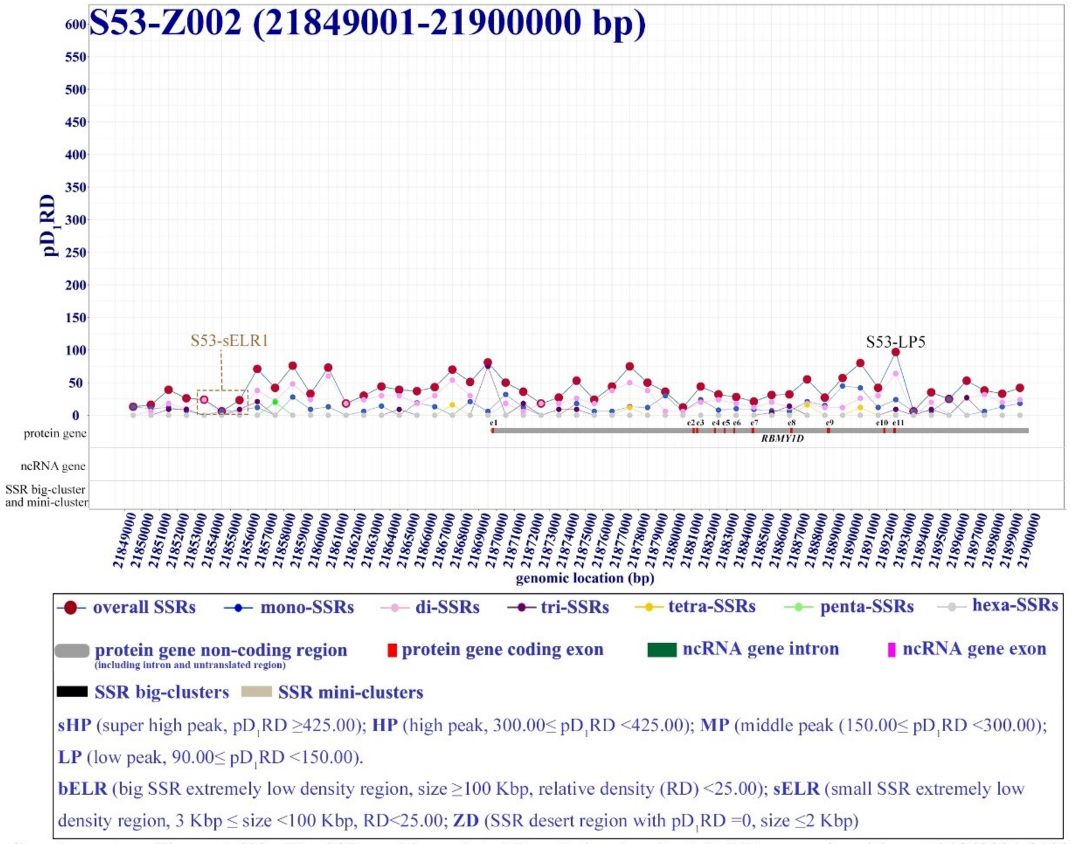

Supplementary Figure 1.432. The SSR position related D_1-relative density (pD_1RD) map of position at 21849001-21900000 bp of human reference Y-DNA (NC_000024.10) at resolution of 1 Kbp.

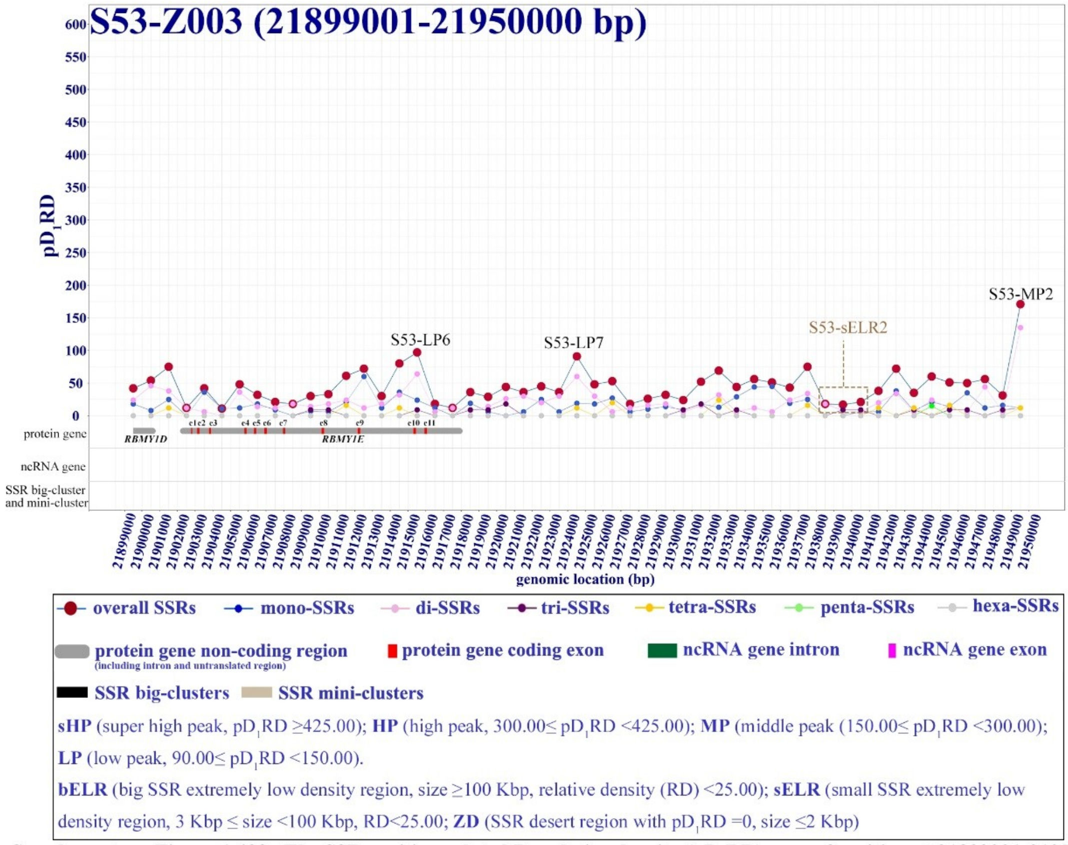

Supplementary Figure 1.433. The SSR position related D_1-relative density (pD_1RD) map of position at 21899001-21950000 bp of human reference Y-DNA (NC_000024.10) at resolution of 1 Kbp.

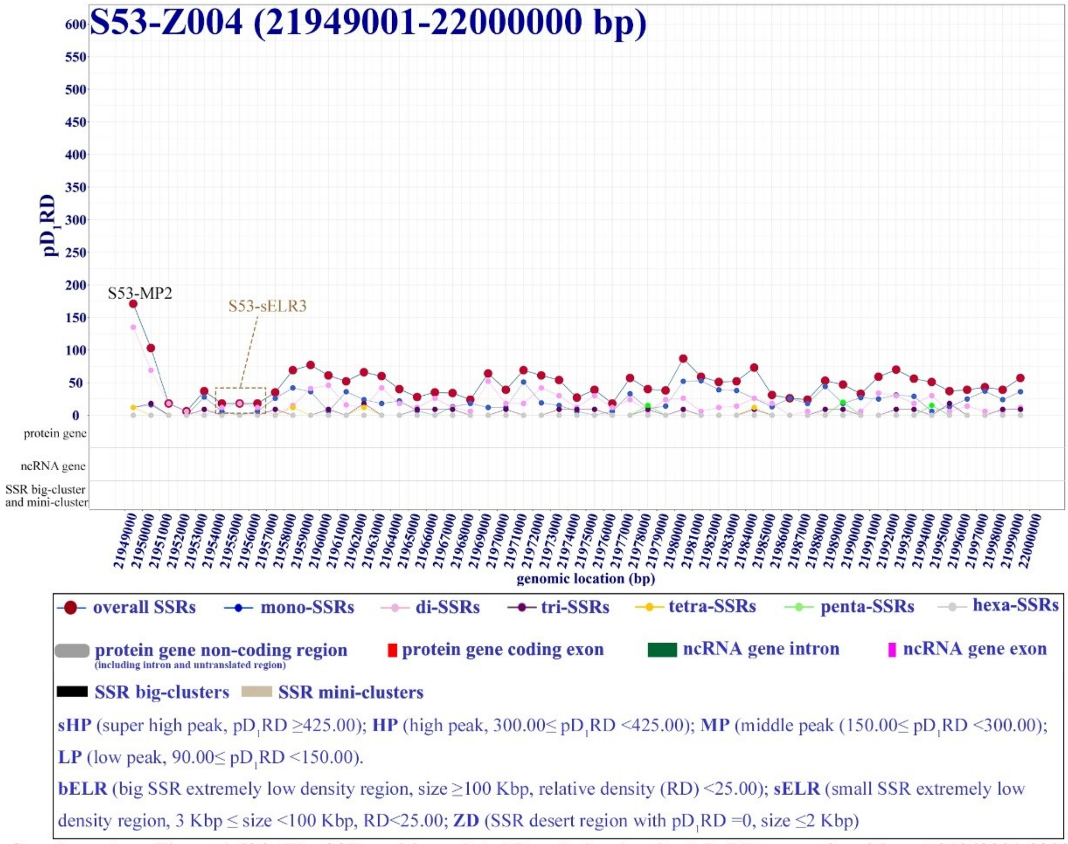

Supplementary Figure 1.434. The SSR position related D_1-relative density (pD_1RD) map of position at 21949001-22000000 bp of human reference Y-DNA (NC_000024.10) at resolution of 1 Kbp.

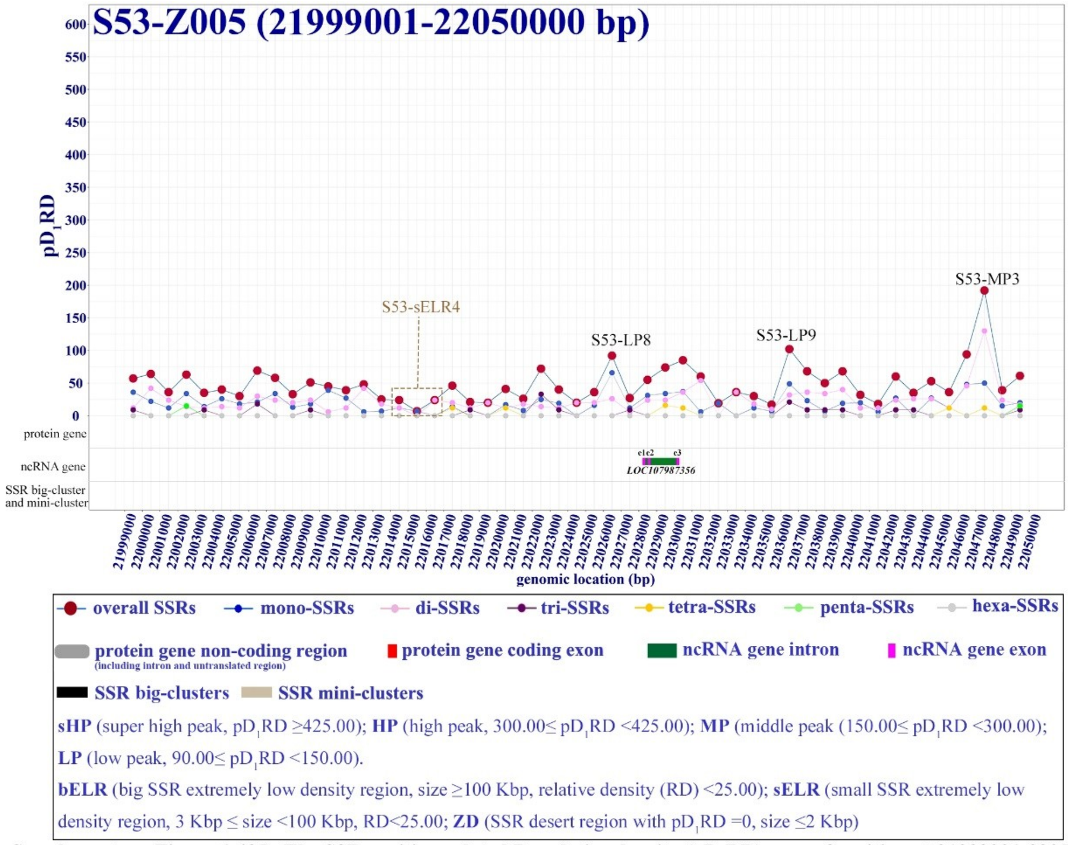

Supplementary Figure 1.435. The SSR position related D_1-relative density (pD_1RD) map of position at 21999001-22050000 bp of human reference Y-DNA (NC_000024.10) at resolution of 1 Kbp.

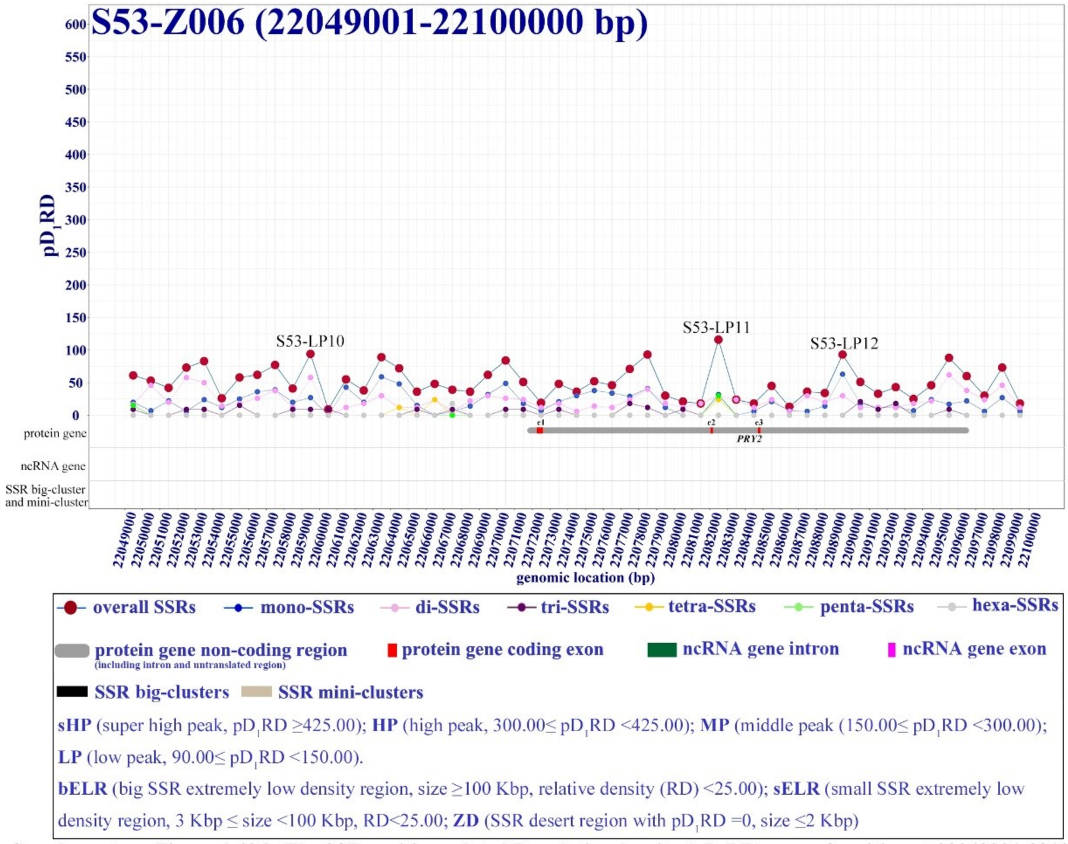

Supplementary Figure 1.436. The SSR position related D_1-relative density (pD_1RD) map of position at 22049001-22100000 bp of human reference Y-DNA (NC_000024.10) at resolution of 1 Kbp.

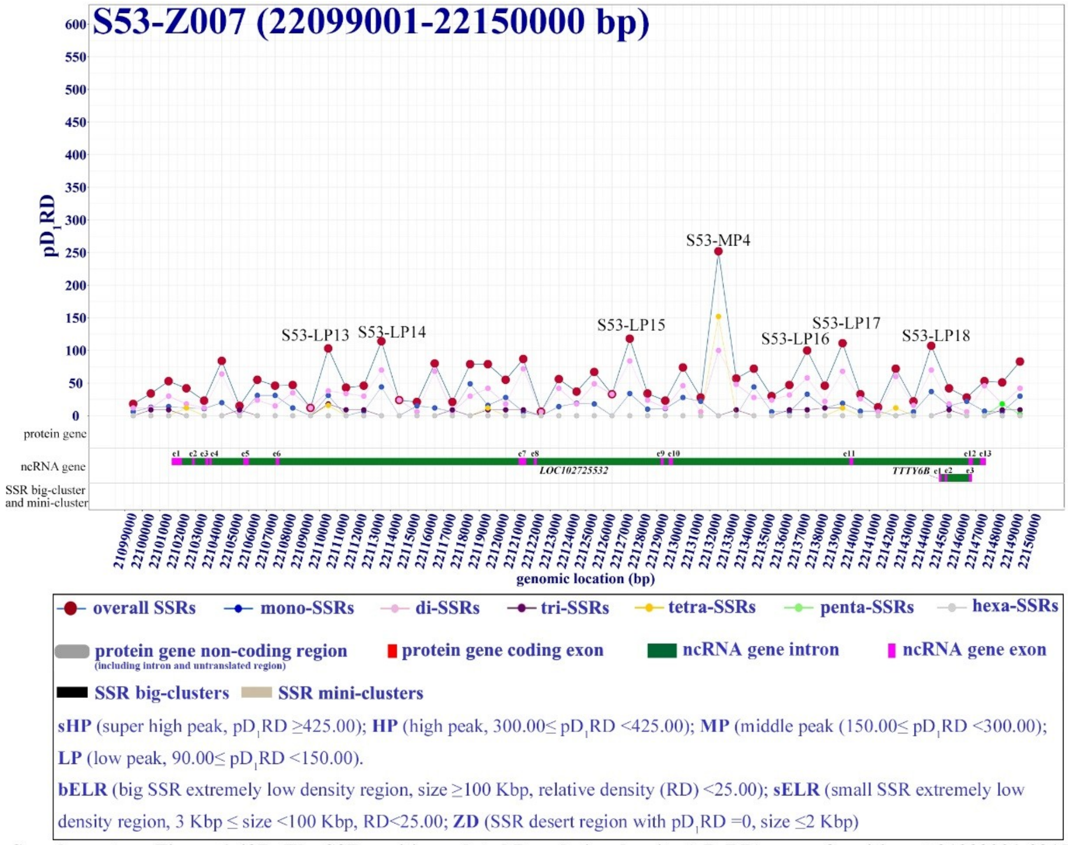

Supplementary Figure 1.437. The SSR position related D_1-relative density (pD_1RD) map of position at 21099001-22150000 bp of human reference Y-DNA (NC_000024.10) at resolution of 1 Kbp.

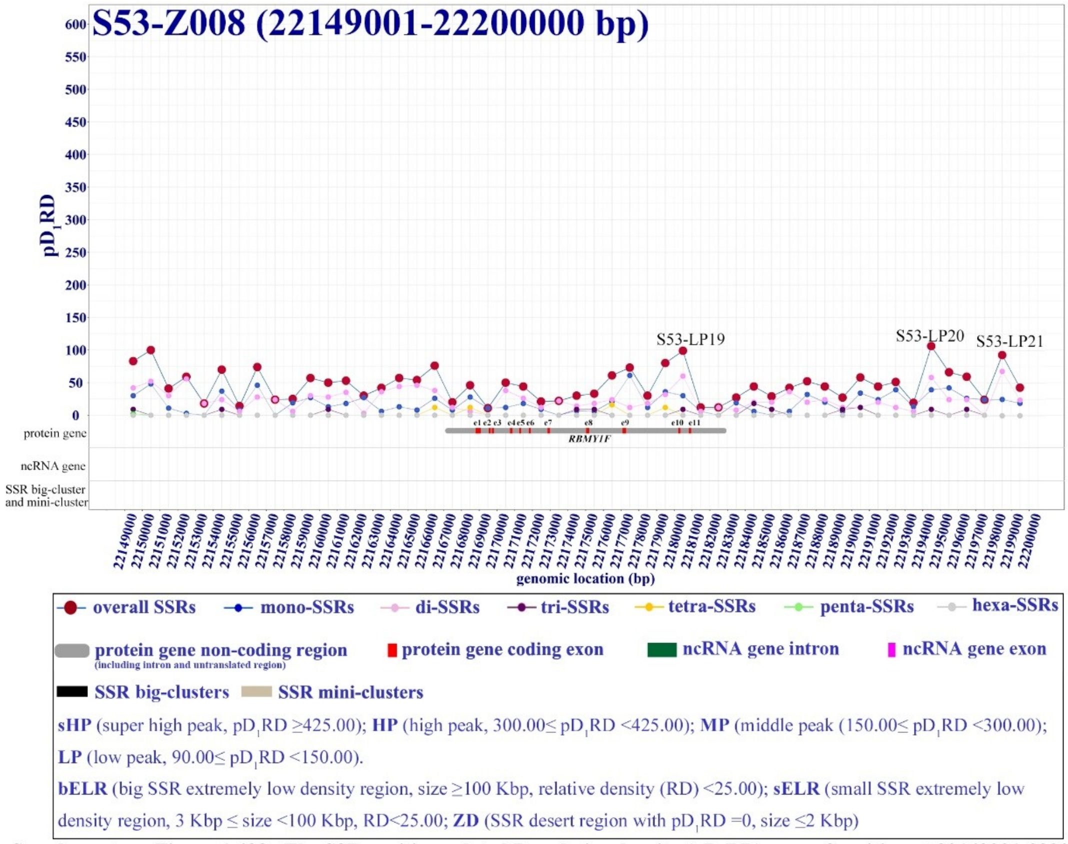

Supplementary Figure 1.438. The SSR position related D_1-relative density (pD_1RD) map of position at 22149001-22200000 bp of human reference Y-DNA (NC_000024.10) at resolution of 1 Kbp.

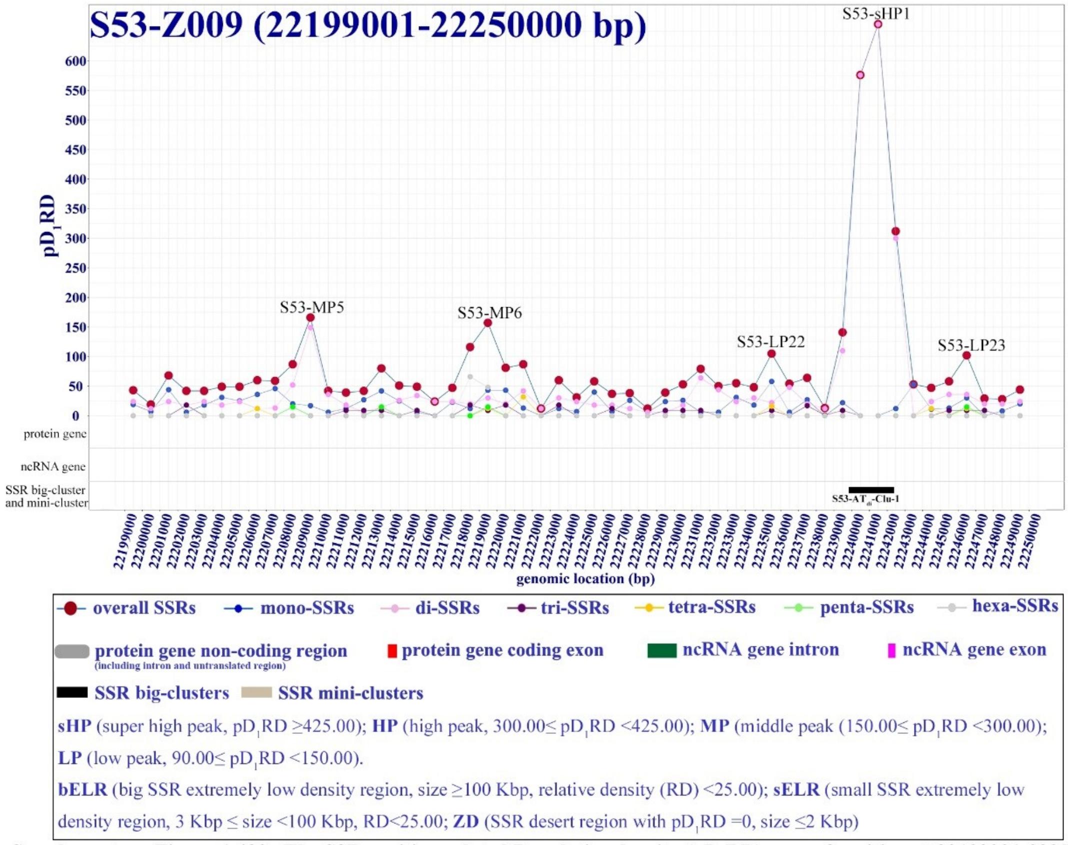

Supplementary Figure 1.439. The SSR position related D_1-relative density (pD_1RD) map of position at 22199001-22250000 bp of human reference Y-DNA (NC_000024.10) at resolution of 1 Kbp.

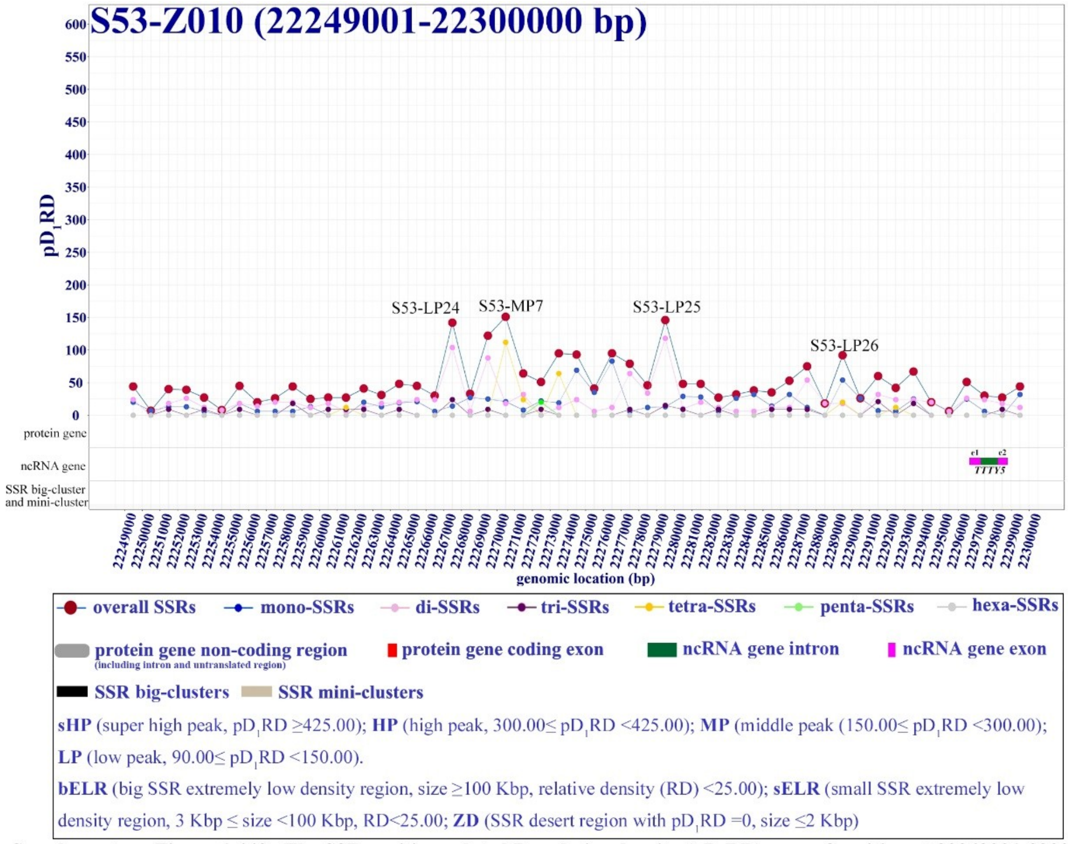

Supplementary Figure 1.440. The SSR position related D_1-relative density (pD_1RD) map of position at 22249001-22300000 bp of human reference Y-DNA (NC_000024.10) at resolution of 1 Kbp.

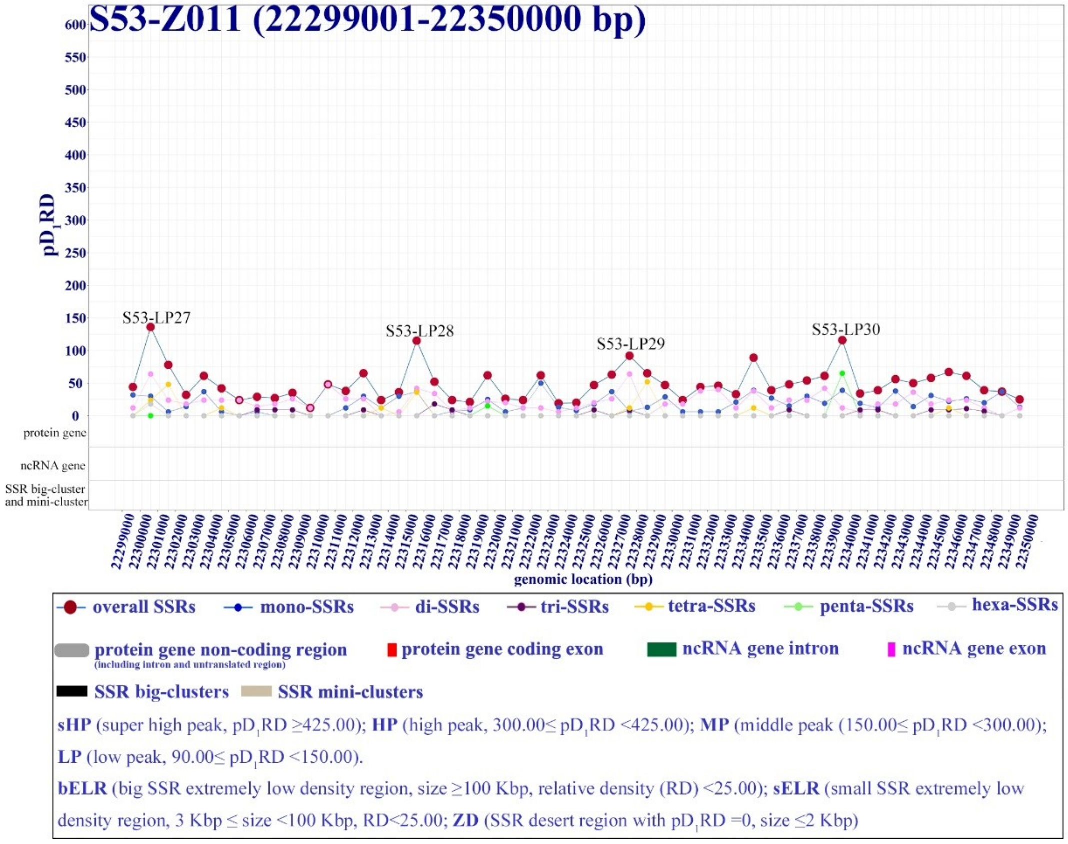

Supplementary Figure 1.441. The SSR position related D_1-relative density (pD_1RD) map of position at 22299001-22350000 bp of human reference Y-DNA (NC_000024.10) at resolution of 1 Kbp.

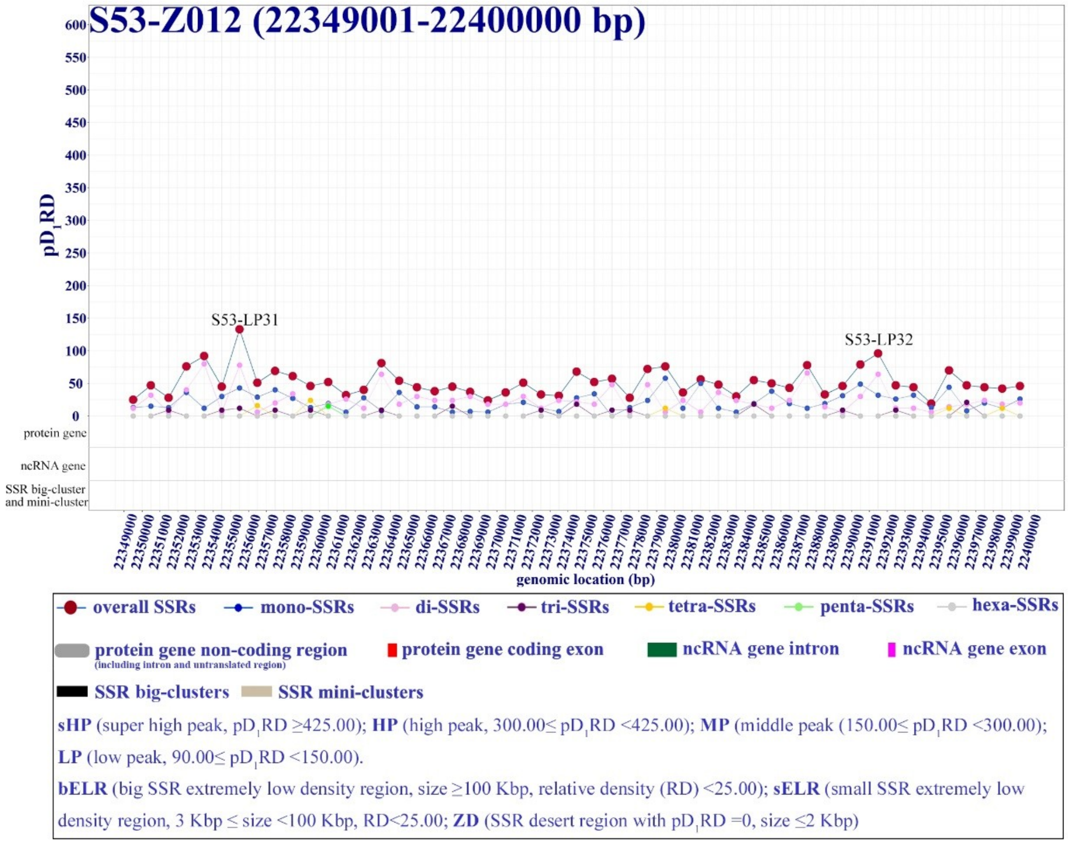

Supplementary Figure 1.442. The SSR position related D_1-relative density (pD_1RD) map of position at 22349001-22400000 bp of human reference Y-DNA (NC_000024.10) at resolution of 1 Kbp.

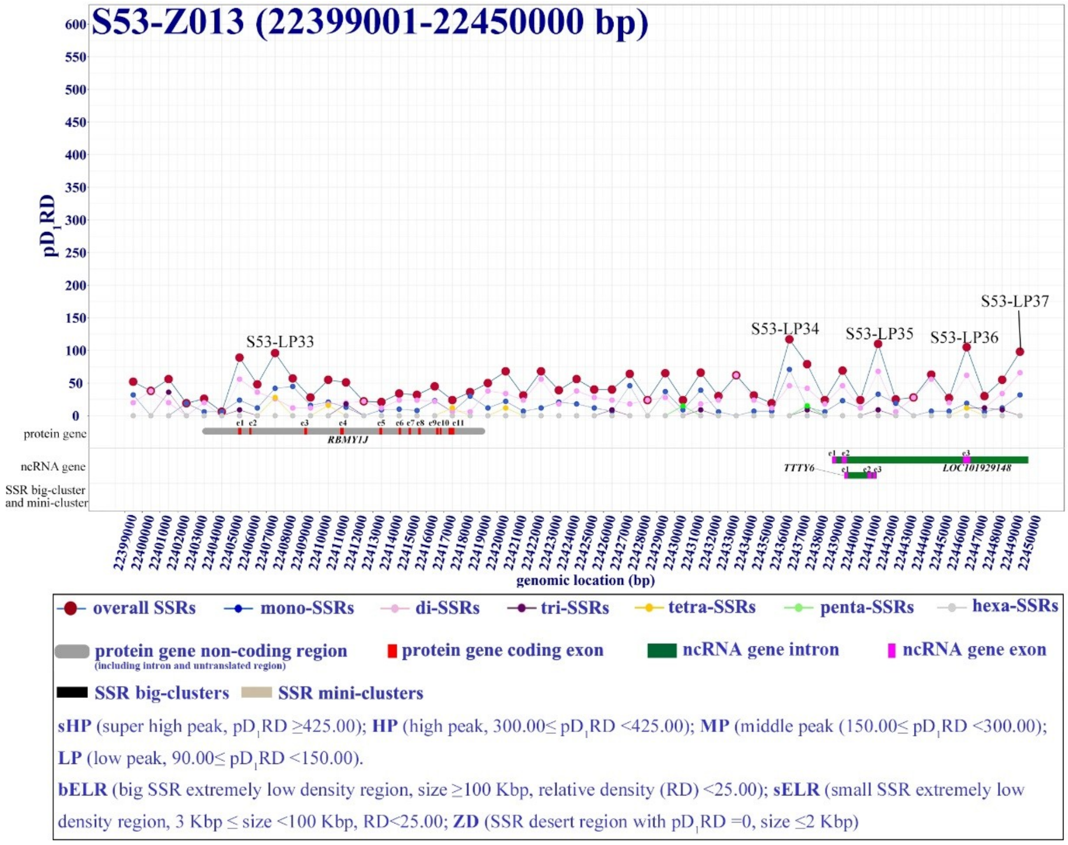

Supplementary Figure 1.443. The SSR position related D_1-relative density (pD_1RD) map of position at 22399001-22450000 bp of human reference Y-DNA (NC_000024.10) at resolution of 1 Kbp.

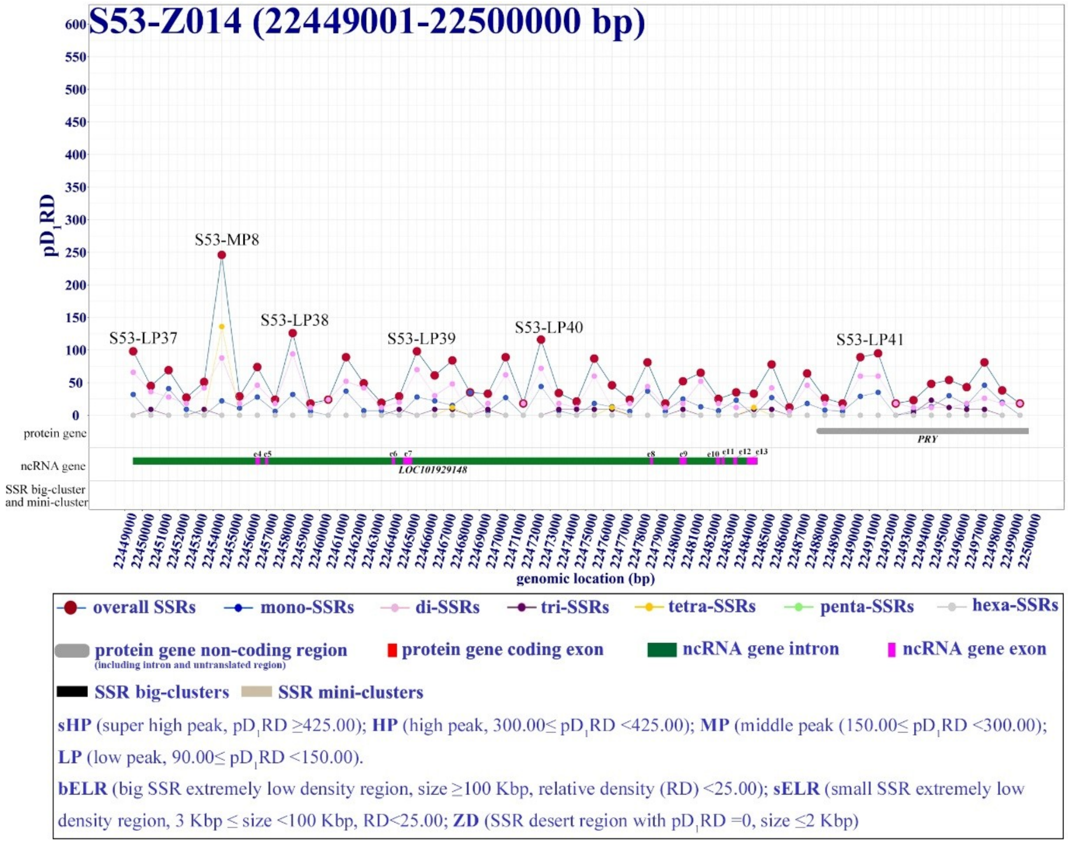

Supplementary Figure 1.444. The SSR position related D_1-relative density (pD_1RD) map of position at 22449001-22500000 bp of human reference Y-DNA (NC_000024.10) at resolution of 1 Kbp.

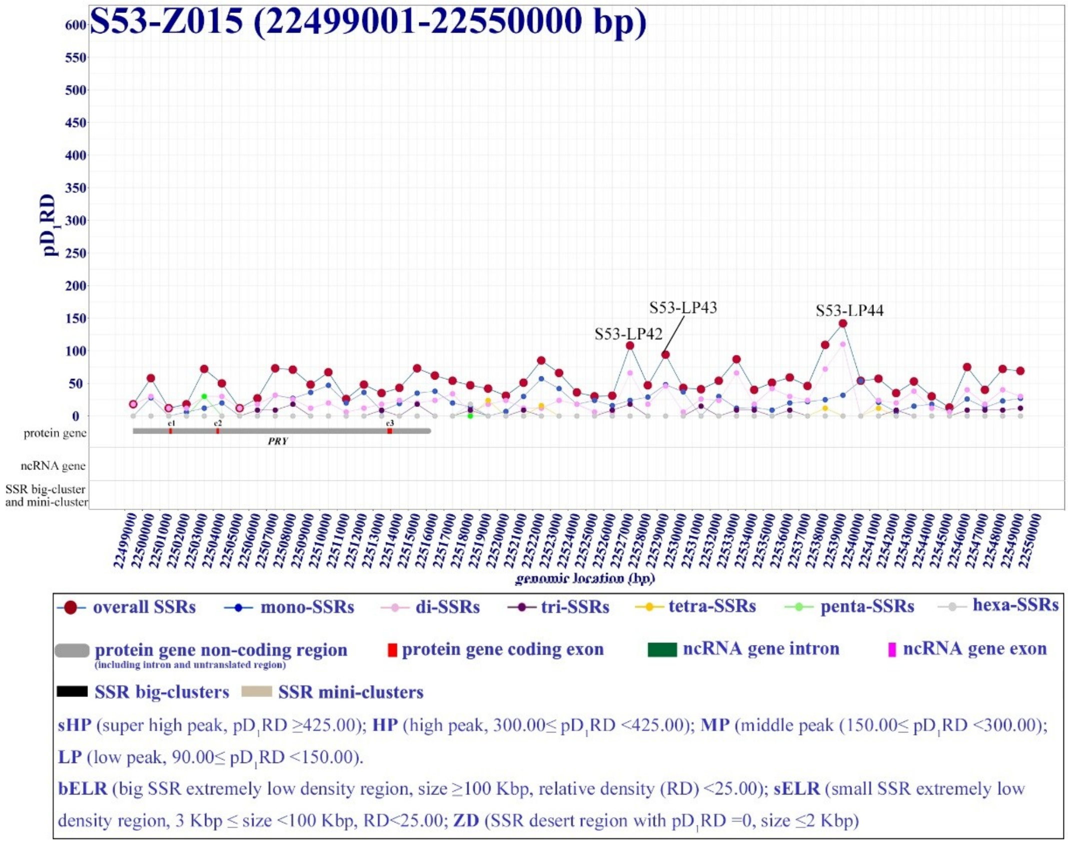

Supplementary Figure 1.445. The SSR position related D_1-relative density (pD_1RD) map of position at 22499001-22550000 bp of human reference Y-DNA (NC_000024.10) at resolution of 1 Kbp.

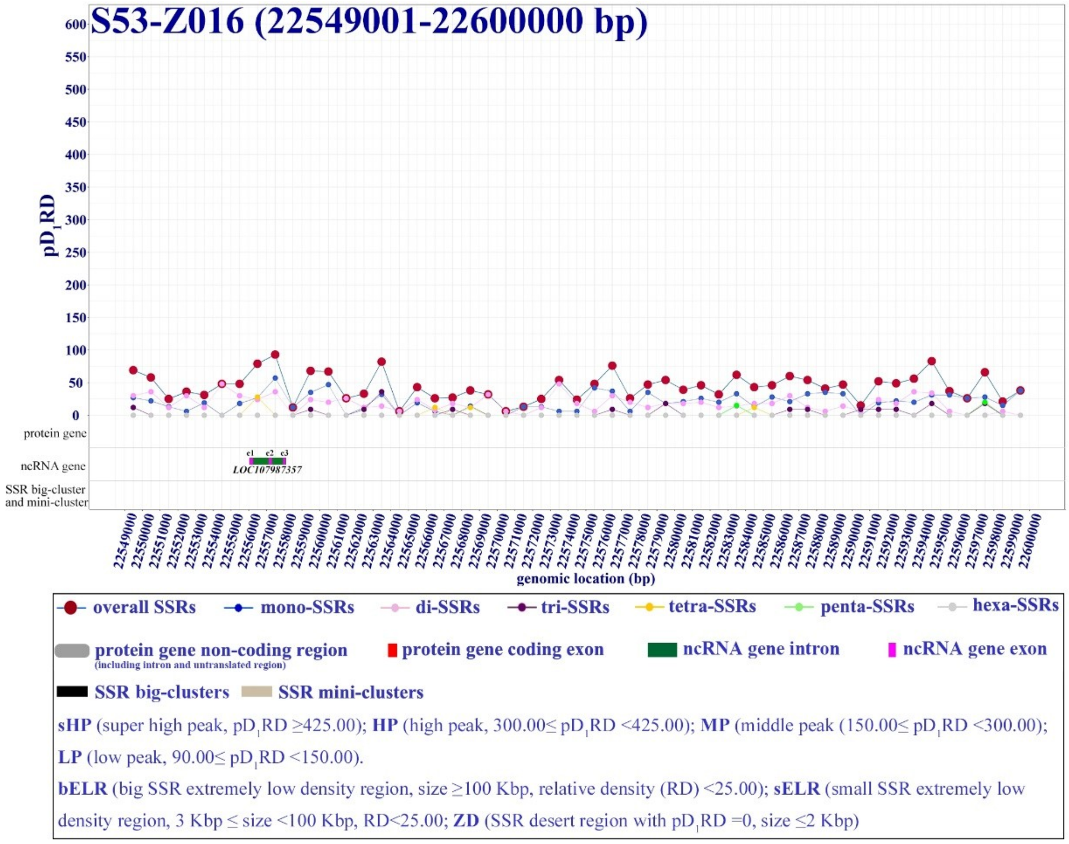

Supplementary Figure 1.446. The SSR position related D_1-relative density (pD_1RD) map of position at 22549001-22600000 bp of human reference Y-DNA (NC_000024.10) at resolution of 1 Kbp.

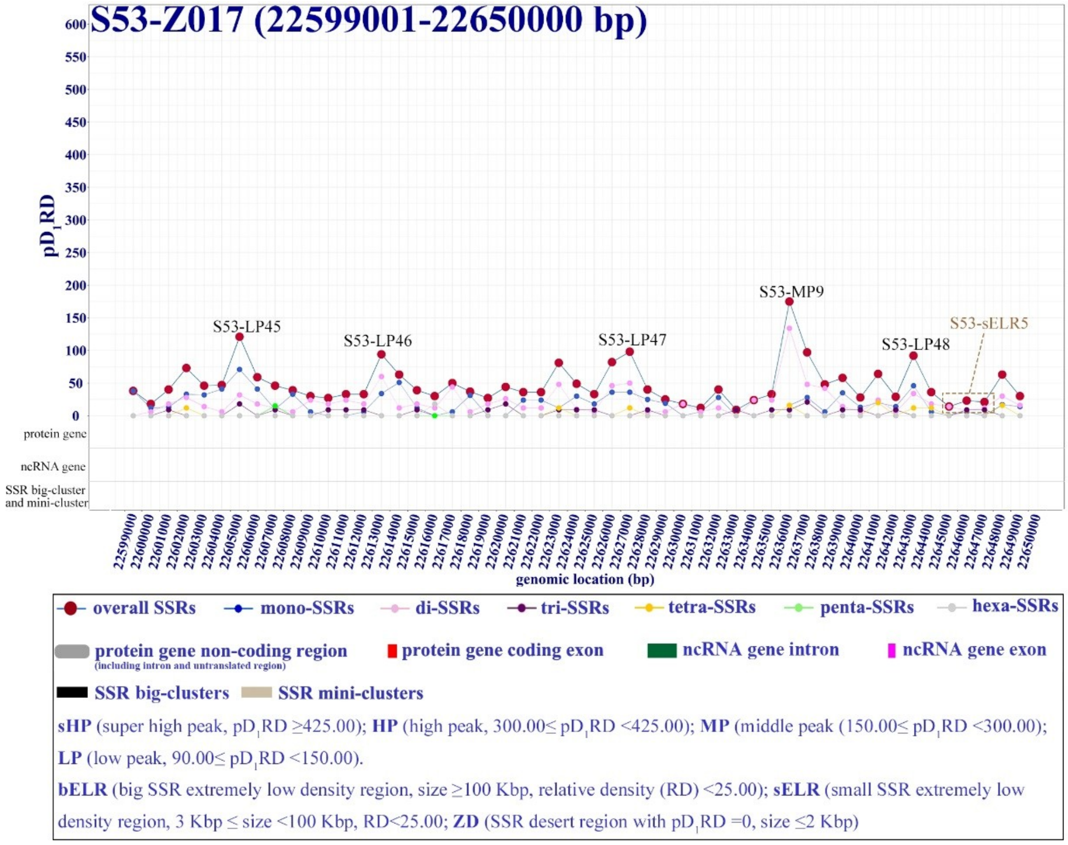

Supplementary Figure 1.447. The SSR position related D_1-relative density (pD_1RD) map of position at 22599001-22650000 bp of human reference Y-DNA (NC_000024.10) at resolution of 1 Kbp.

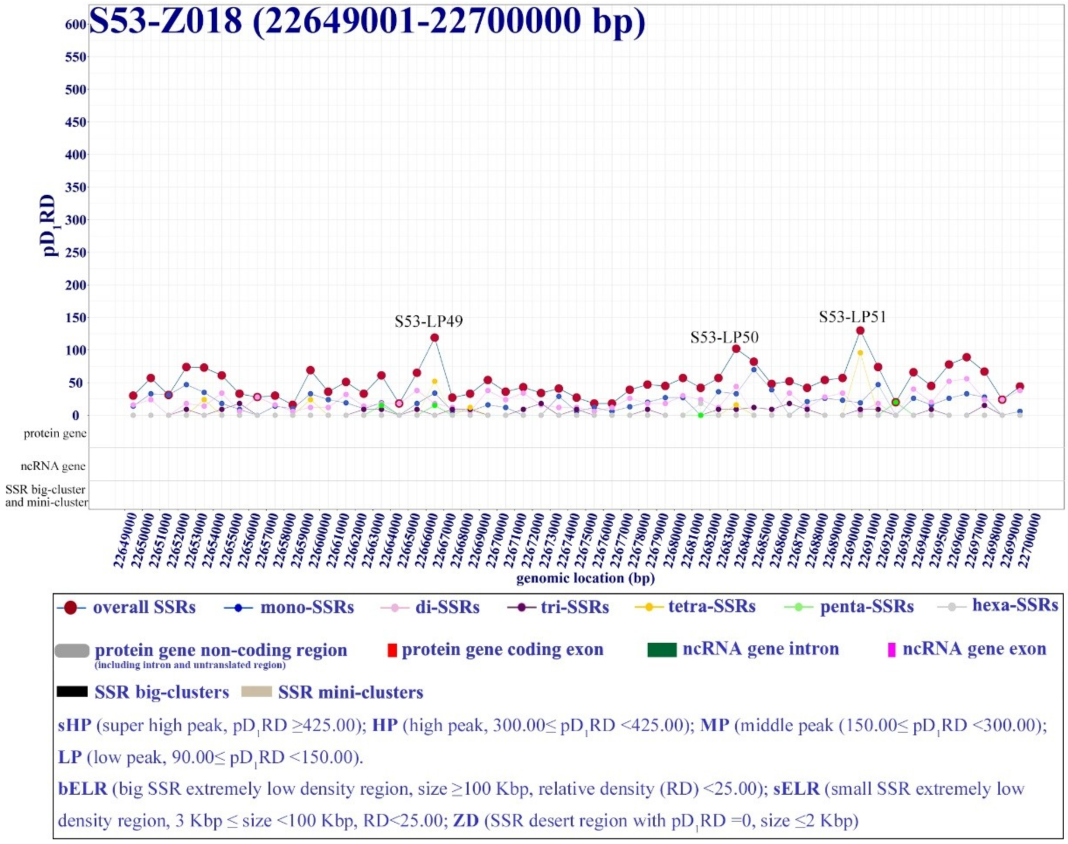

Supplementary Figure 1.448. The SSR position related D_1-relative density (pD_1RD) map of position at 22649001-22700000 bp of human reference Y-DNA (NC_000024.10) at resolution of 1 Kbp.

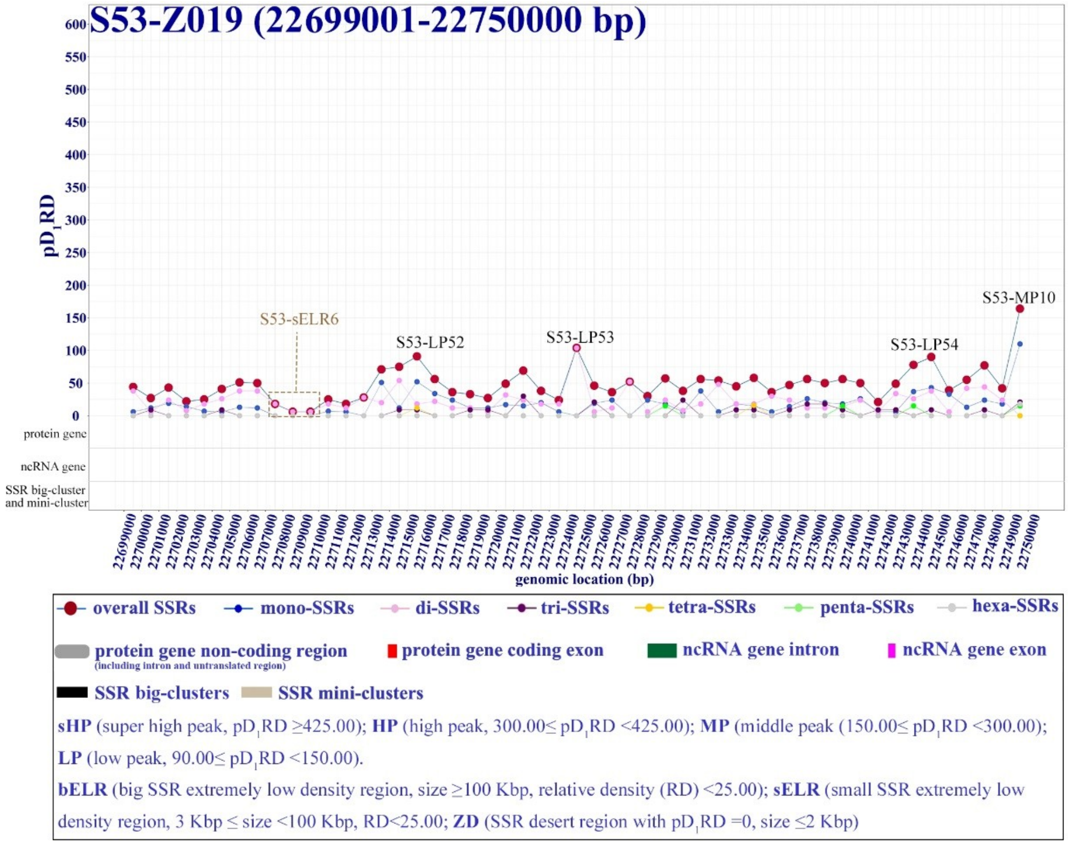

Supplementary Figure 1.449. The SSR position related D_1-relative density (pD_1RD) map of position at 22699001-22750000 bp of human reference Y-DNA (NC_000024.10) at resolution of 1 Kbp.

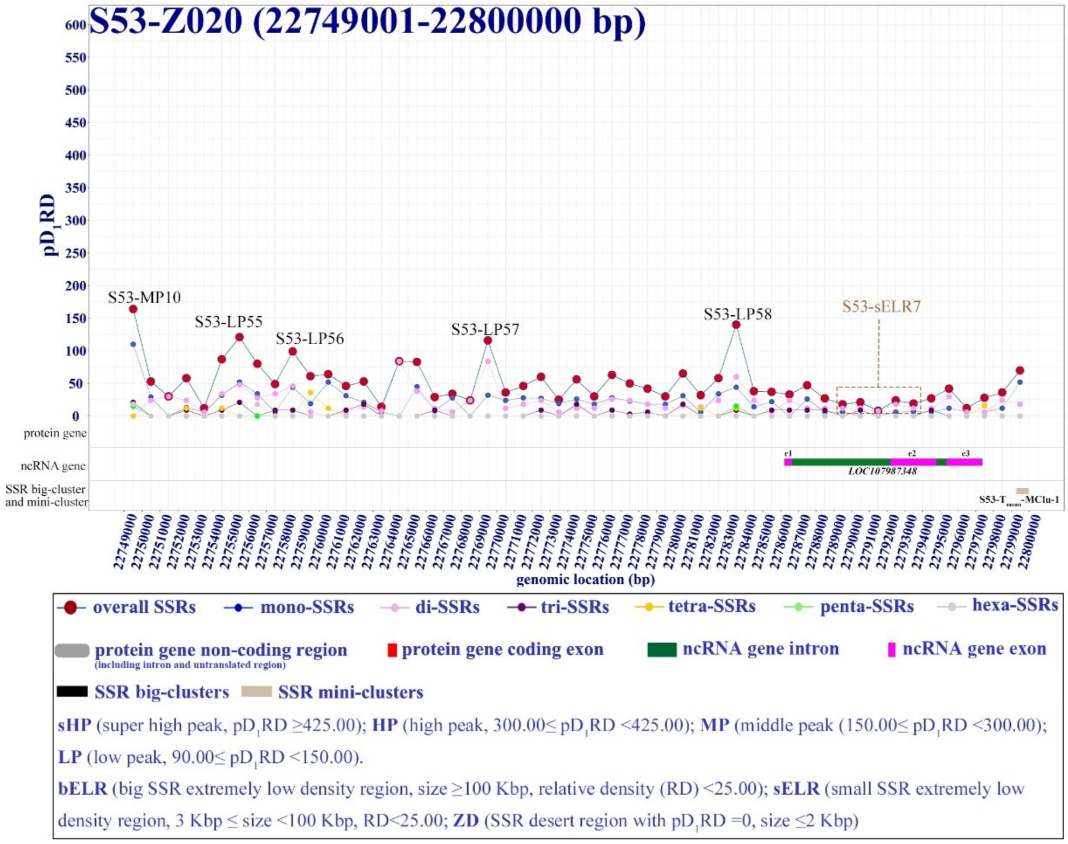

Supplementary Figure 1.450. The SSR position related D_1-relative density (pD_1RD) map of position at 22749001-22800000 bp of human reference Y-DNA (NC_000024.10) at resolution of 1 Kbp.

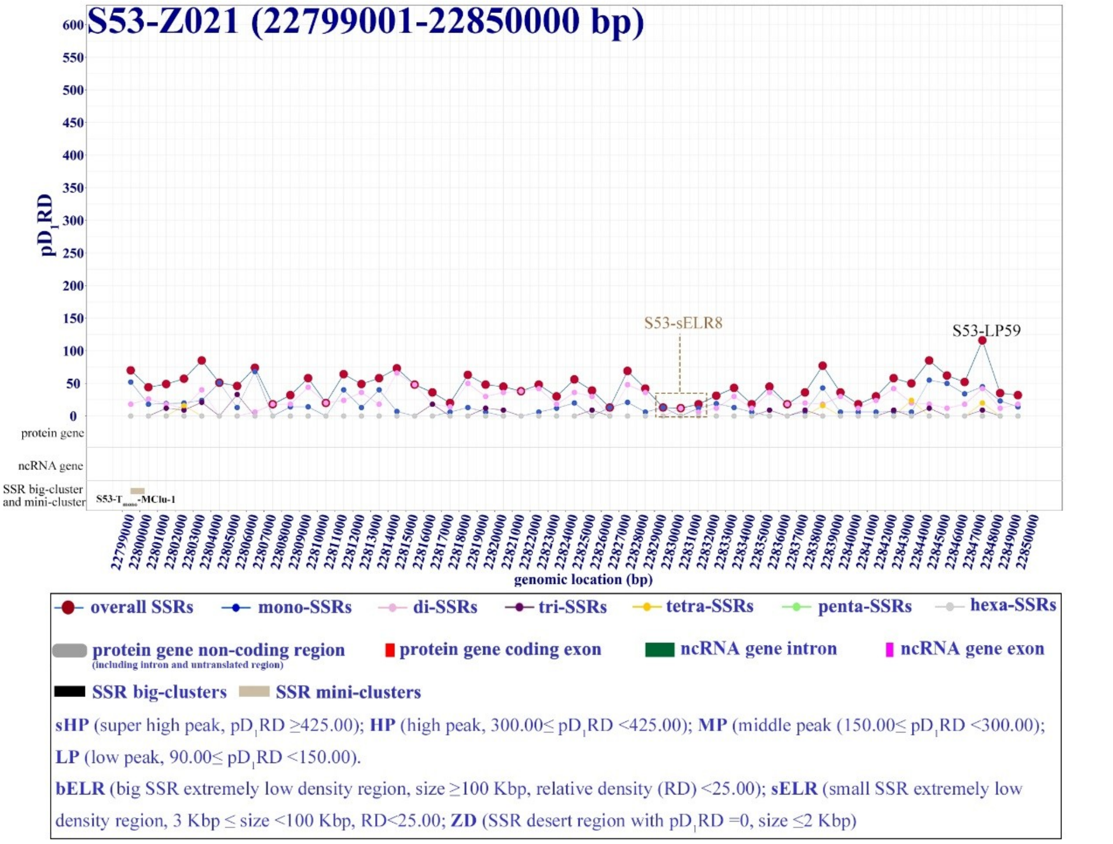

Supplementary Figure 1.451. The SSR position related D_1-relative density (pD_1RD) map of position at 22799001-22850000 bp of human reference Y-DNA (NC_000024.10) at resolution of 1 Kbp.

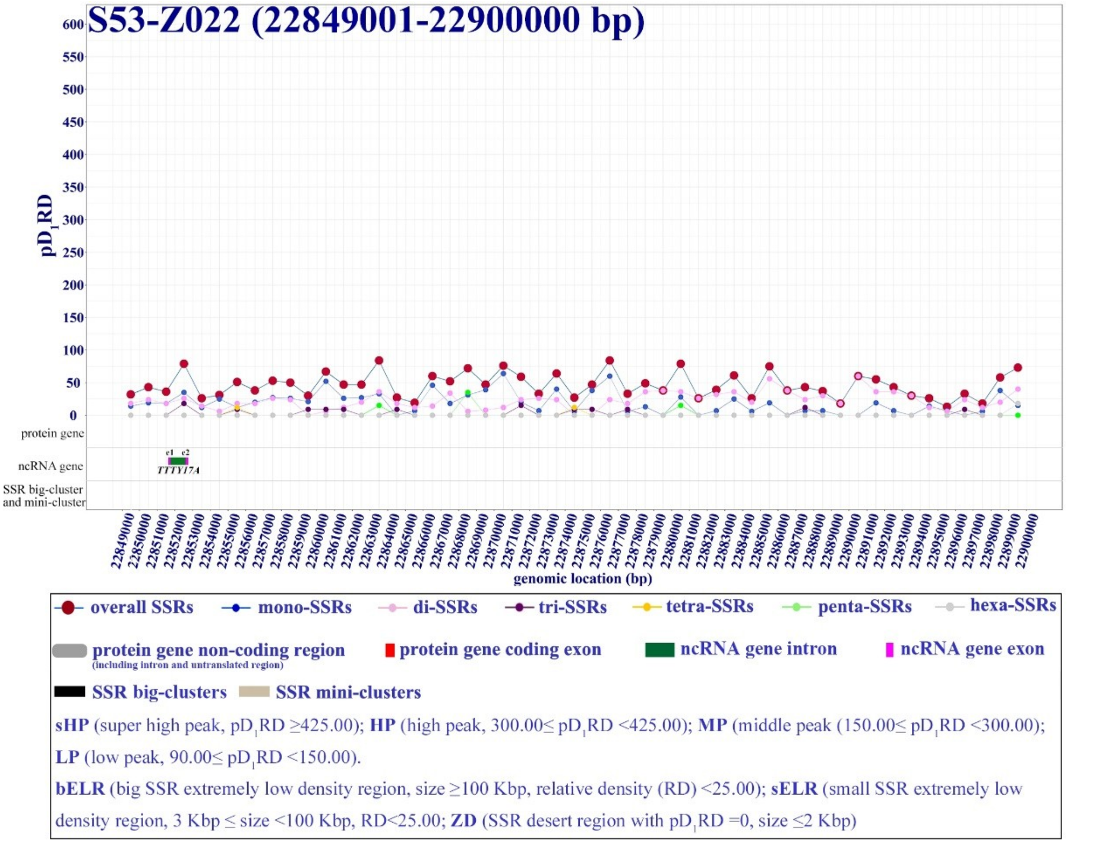

Supplementary Figure 1.452. The SSR position related D_1-relative density (pD_1RD) map of position at 22849001-22900000 bp of human reference Y-DNA (NC_000024.10) at resolution of 1 Kbp.

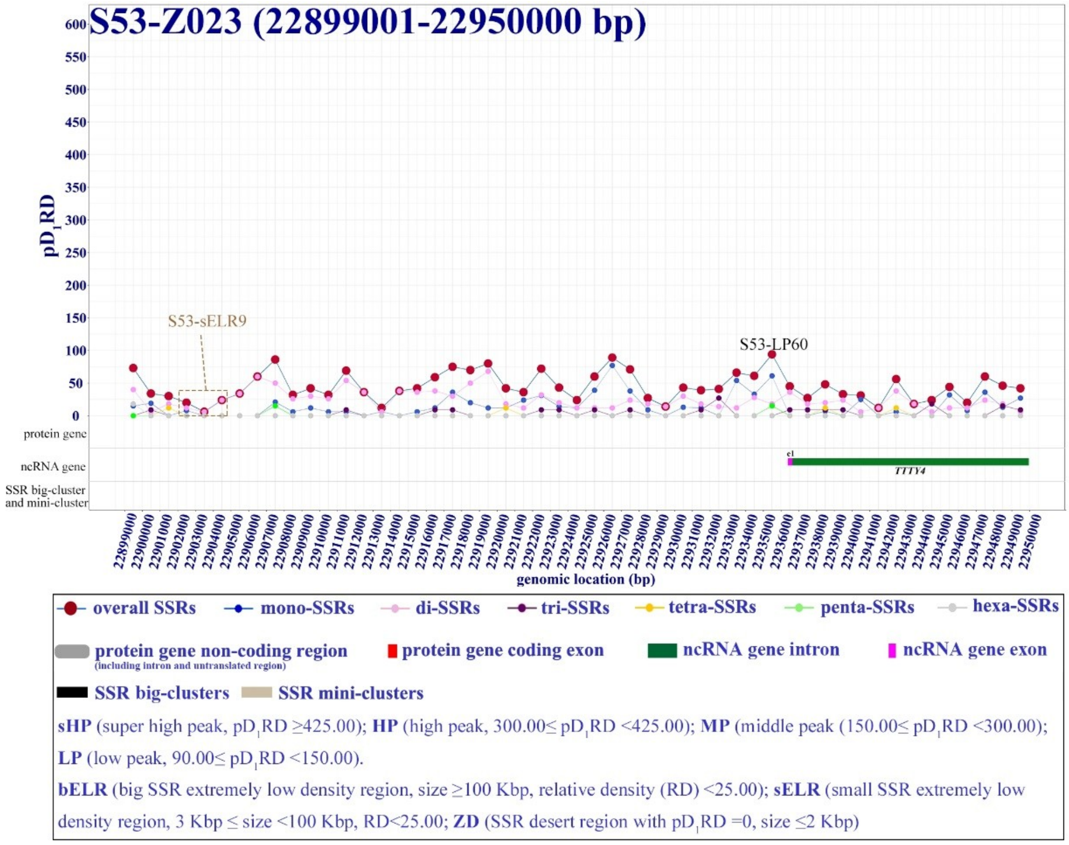

Supplementary Figure 1.453. The SSR position related D_1-relative density (pD_1RD) map of position at 22899001-22950000 bp of human reference Y-DNA (NC_000024.10) at resolution of 1 Kbp.

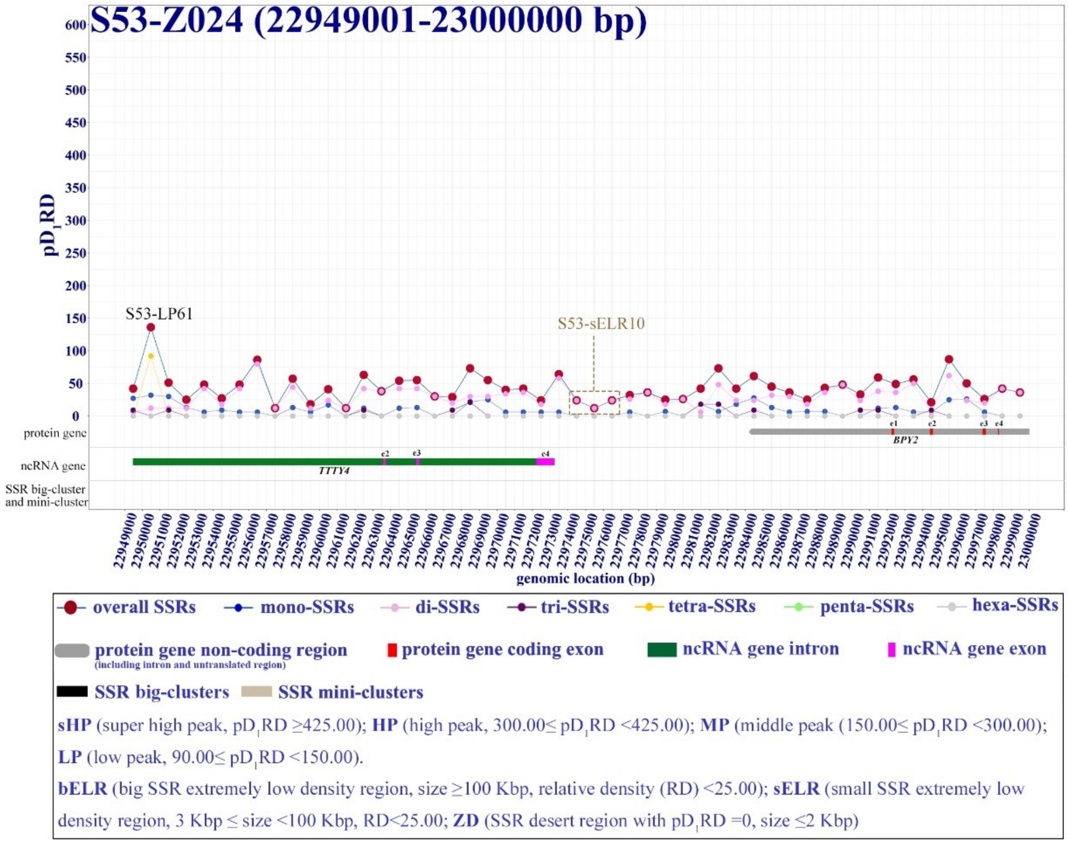

Supplementary Figure 1.454. The SSR position related D_1-relative density (pD_1RD) map of position at 22949001-23000000 bp of human reference Y-DNA (NC_000024.10) at resolution of 1 Kbp.

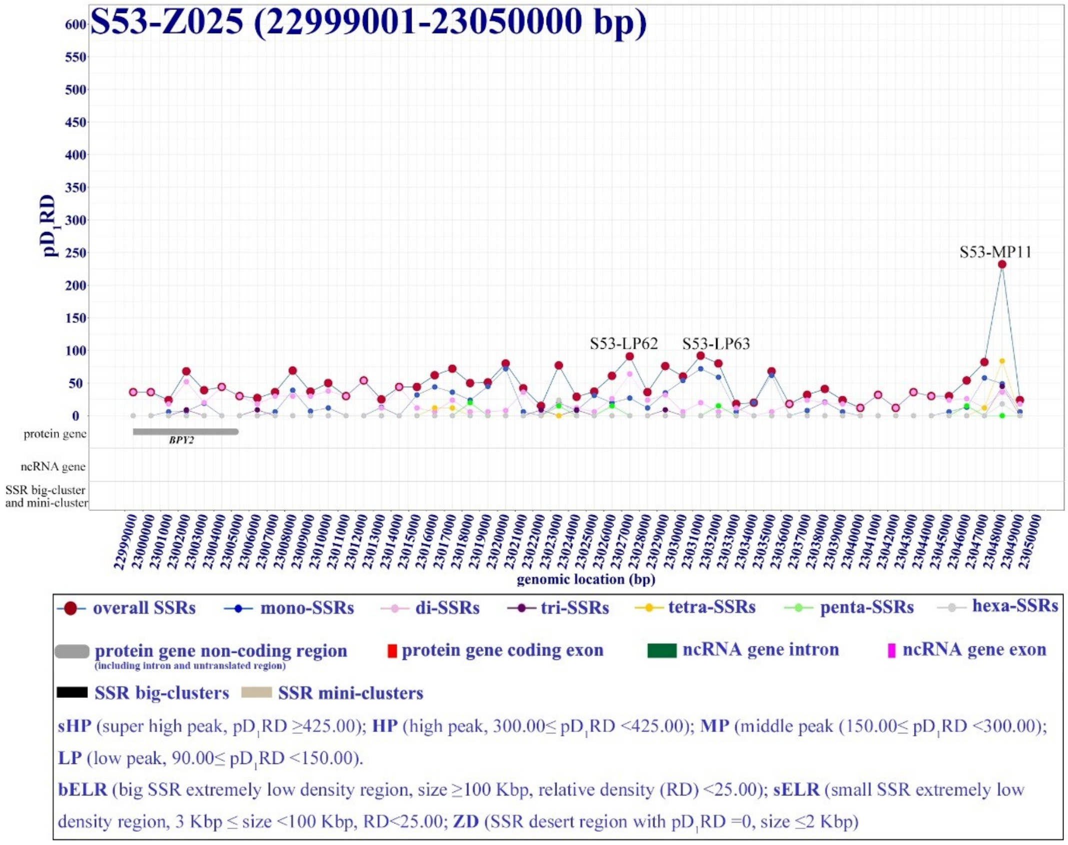

Supplementary Figure 1.455. The SSR position related D_1-relative density (pD_1RD) map of position at 22999001-23050000 bp of human reference Y-DNA (NC_000024.10) at resolution of 1 Kbp.

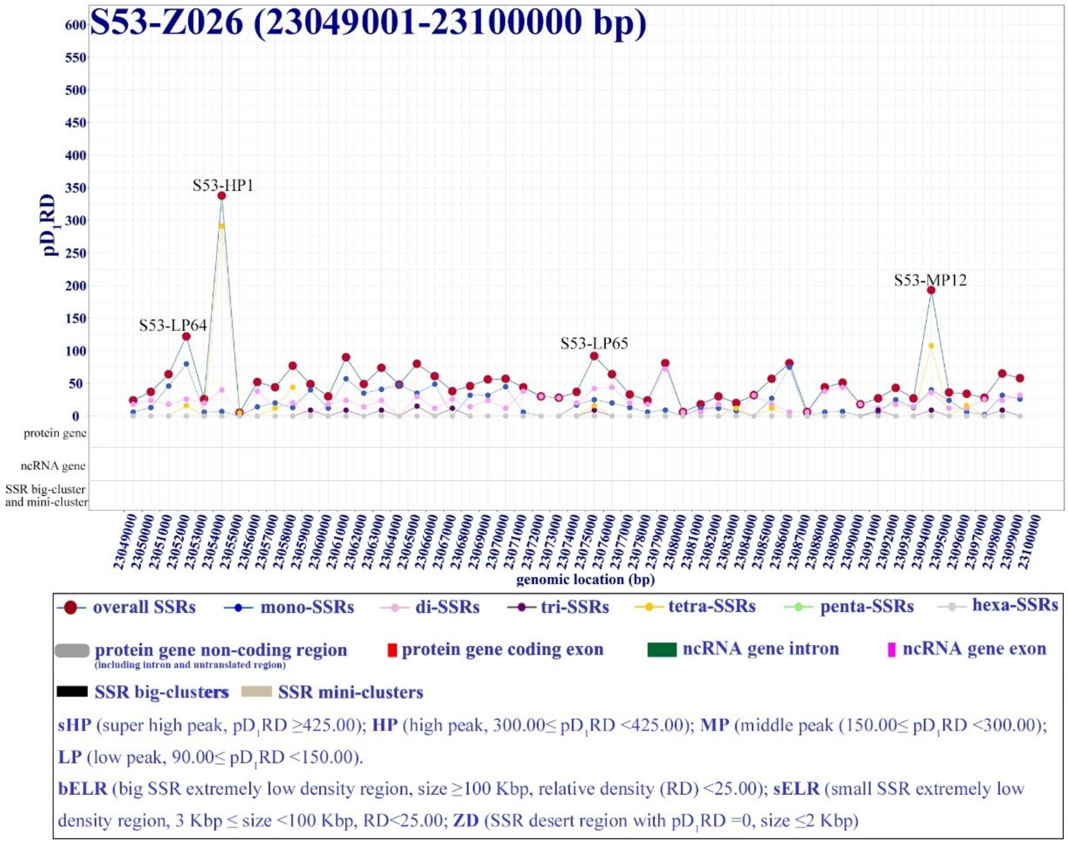

Supplementary Figure 1.456. The SSR position related D_1-relative density (pD_1RD) map of position at 23049001-23100000 bp of human reference Y-DNA (NC_000024.10) at resolution of 1 Kbp.

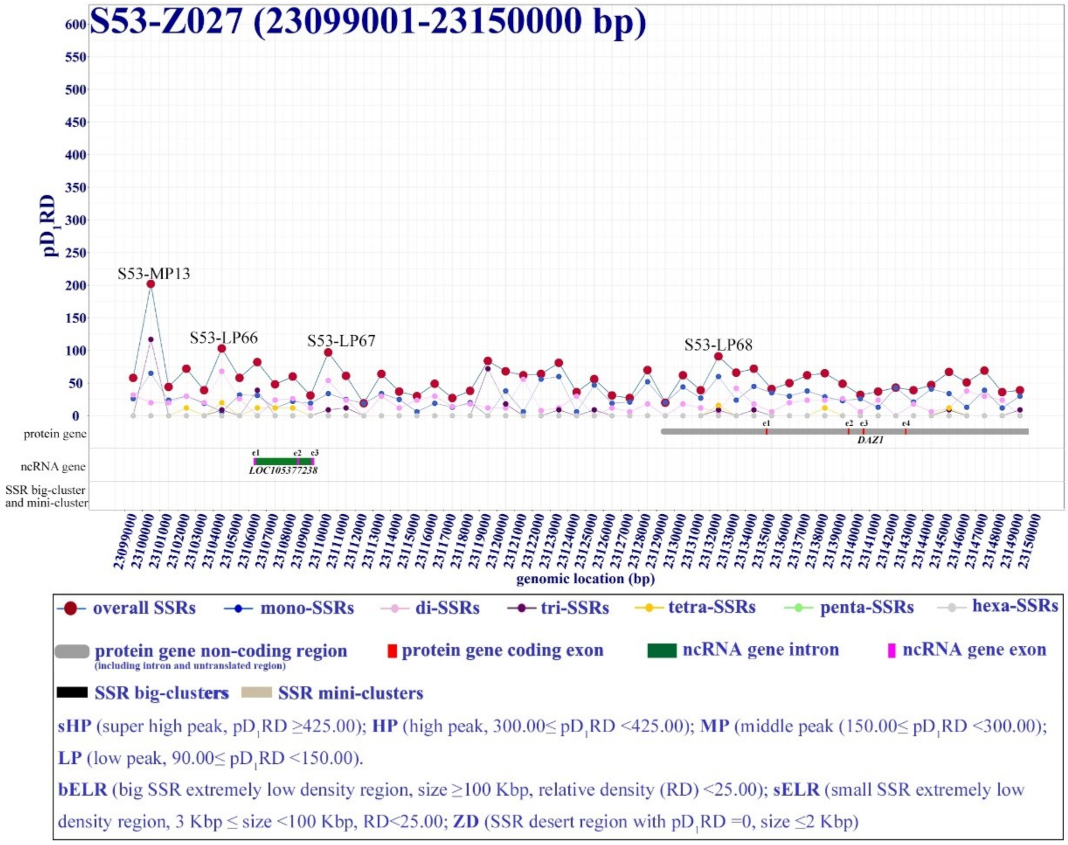

Supplementary Figure 1.457. The SSR position related D_1-relative density (pD_1RD) map of position at 23099001-23150000 bp of human reference Y-DNA (NC_000024.10) at resolution of 1 Kbp.

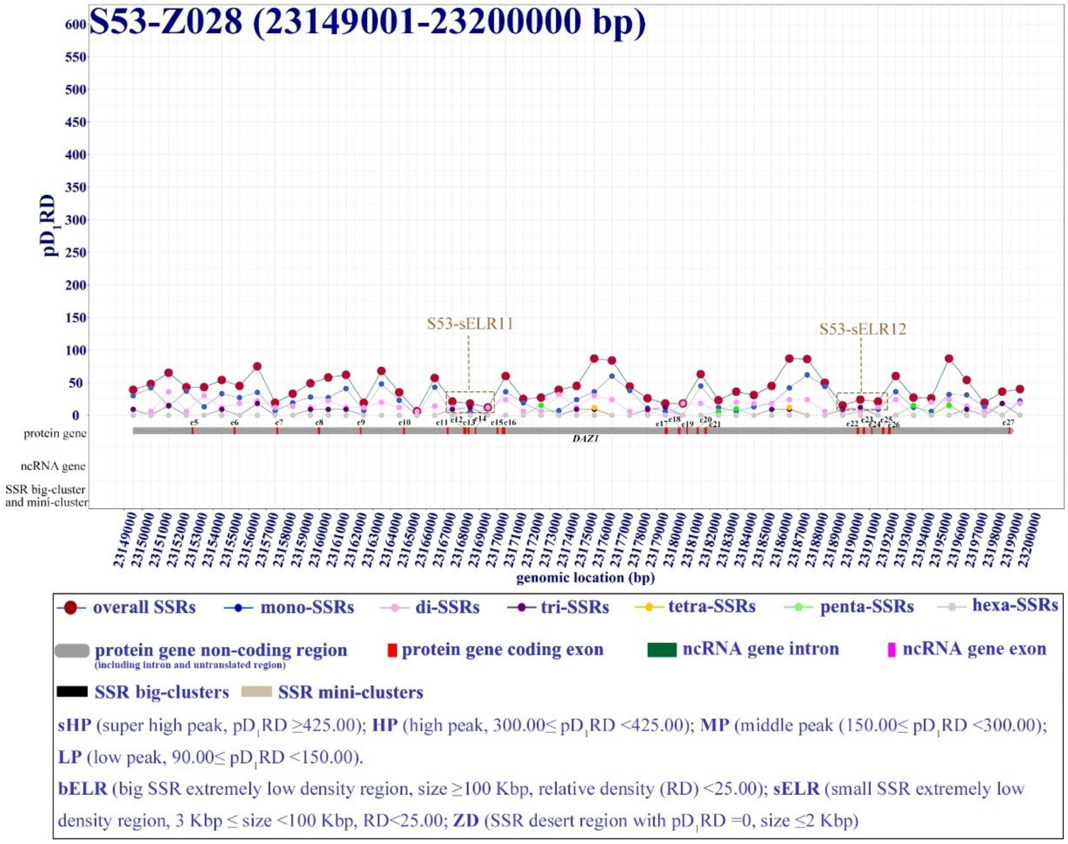

Supplementary Figure 1.458. The SSR position related D_1-relative density (pD_1RD) map of position at 23149001-23200000 bp of human reference Y-DNA (NC_000024.10) at resolution of 1 Kbp.

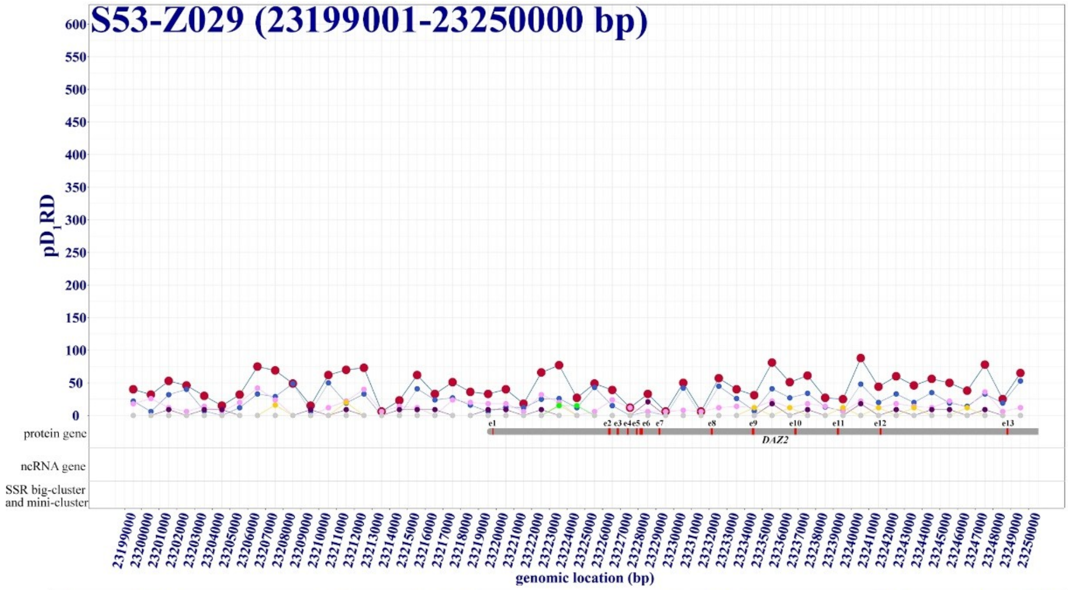

overall SSRs | mono-SSRs | di-SSRs | tri-SSRs | tetra-SSRs | penta-SSRs | hexa-SSRs

protein gene non-coding region (including intron and untranslated region) | protein gene coding exon | ncRNA gene intron | ncRNA gene exon

SSR big-clusters | SSR mini-clusters

sHP (super high peak, $pD_1RD \geq 425.00$); **HP** (high peak, $300.00 \leq pD_1RD < 425.00$); **MP** (middle peak ($150.00 \leq pD_1RD < 300.00$); **LP** (low peak, $90.00 \leq pD_1RD < 150.00$).

bELR (big SSR extremely low density region, size ≥100 Kbp, relative density (RD) <25.00); **sELR** (small SSR extremely low density region, 3 Kbp ≤ size <100 Kbp, RD<25.00; **ZD** (SSR desert region with $pD_1RD = 0$, size ≤2 Kbp)

Supplementary Figure 1.459. The SSR position related D_1-relative density (pD_1RD) map of position at 23199001-23250000 bp of human reference Y-DNA (NC_000024.10) at resolution of 1 Kbp.

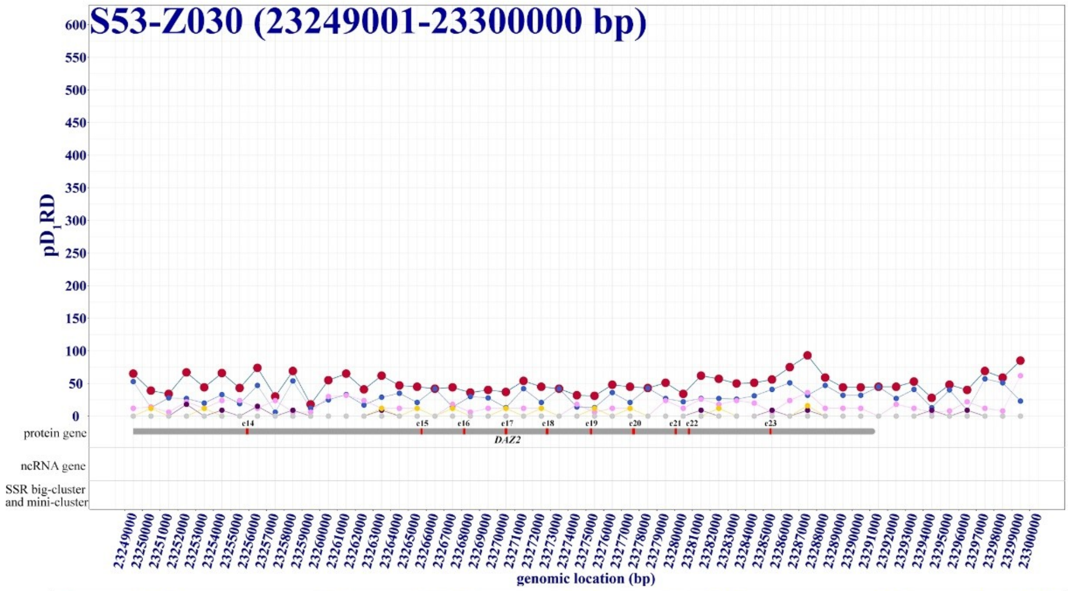

overall SSRs | mono-SSRs | di-SSRs | tri-SSRs | tetra-SSRs | penta-SSRs | hexa-SSRs

protein gene non-coding region (including intron and untranslated region) | protein gene coding exon | ncRNA gene intron | ncRNA gene exon

SSR big-clusters | SSR mini-clusters

sHP (super high peak, $pD_1RD \geq 425.00$); **HP** (high peak, $300.00 \leq pD_1RD < 425.00$); **MP** (middle peak ($150.00 \leq pD_1RD < 300.00$); **LP** (low peak, $90.00 \leq pD_1RD < 150.00$).

bELR (big SSR extremely low density region, size ≥100 Kbp, relative density (RD) <25.00); **sELR** (small SSR extremely low density region, 3 Kbp ≤ size <100 Kbp, RD<25.00; **ZD** (SSR desert region with $pD_1RD = 0$, size ≤2 Kbp)

Supplementary Figure 1.460. The SSR position related D_1-relative density (pD_1RD) map of position at 23249001-23300000 bp of human reference Y-DNA (NC_000024.10) at resolution of 1 Kbp.

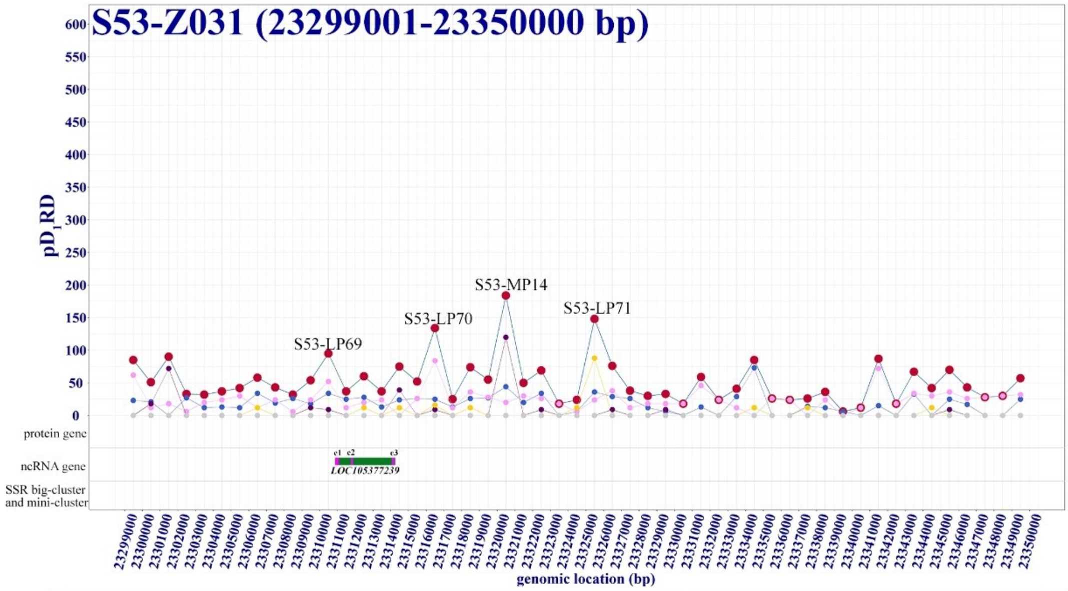

overall SSRs · mono-SSRs · di-SSRs · tri-SSRs · tetra-SSRs · penta-SSRs · hexa-SSRs

protein gene non-coding region (including intron and untranslated region) · protein gene coding exon · ncRNA gene intron · ncRNA gene exon

SSR big-clusters · SSR mini-clusters

sHP (super high peak, $pD_1RD \geq 425.00$); **HP** (high peak, $300.00 \leq pD_1RD < 425.00$); **MP** (middle peak ($150.00 \leq pD_1RD < 300.00$); **LP** (low peak, $90.00 \leq pD_1RD < 150.00$).

bELR (big SSR extremely low density region, size ≥100 Kbp, relative density (RD) <25.00); **sELR** (small SSR extremely low density region, 3 Kbp ≤ size <100 Kbp, RD<25.00; **ZD** (SSR desert region with $pD_1RD = 0$, size ≤2 Kbp)

Supplementary Figure 1.461. The SSR position related D_1-relative density (pD_1RD) map of position at 23299001-23350000 bp of human reference Y-DNA (NC_000024.10) at resolution of 1 Kbp.

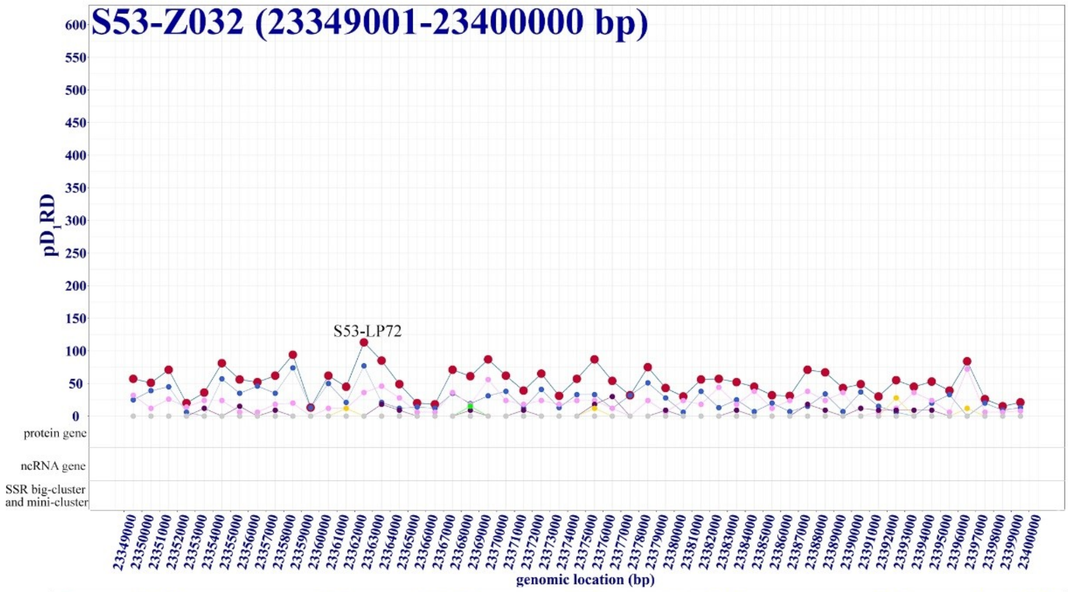

overall SSRs · mono-SSRs · di-SSRs · tri-SSRs · tetra-SSRs · penta-SSRs · hexa-SSRs

protein gene non-coding region (including intron and untranslated region) · protein gene coding exon · ncRNA gene intron · ncRNA gene exon

SSR big-clusters · SSR mini-clusters

sHP (super high peak, $pD_1RD \geq 425.00$); **HP** (high peak, $300.00 \leq pD_1RD < 425.00$); **MP** (middle peak ($150.00 \leq pD_1RD < 300.00$); **LP** (low peak, $90.00 \leq pD_1RD < 150.00$).

bELR (big SSR extremely low density region, size ≥100 Kbp, relative density (RD) <25.00); **sELR** (small SSR extremely low density region, 3 Kbp ≤ size <100 Kbp, RD<25.00; **ZD** (SSR desert region with $pD_1RD = 0$, size ≤2 Kbp)

Supplementary Figure 1.462. The SSR position related D_1-relative density (pD_1RD) map of position at 23349001-23400000 bp of human reference Y-DNA (NC_000024.10) at resolution of 1 Kbp.

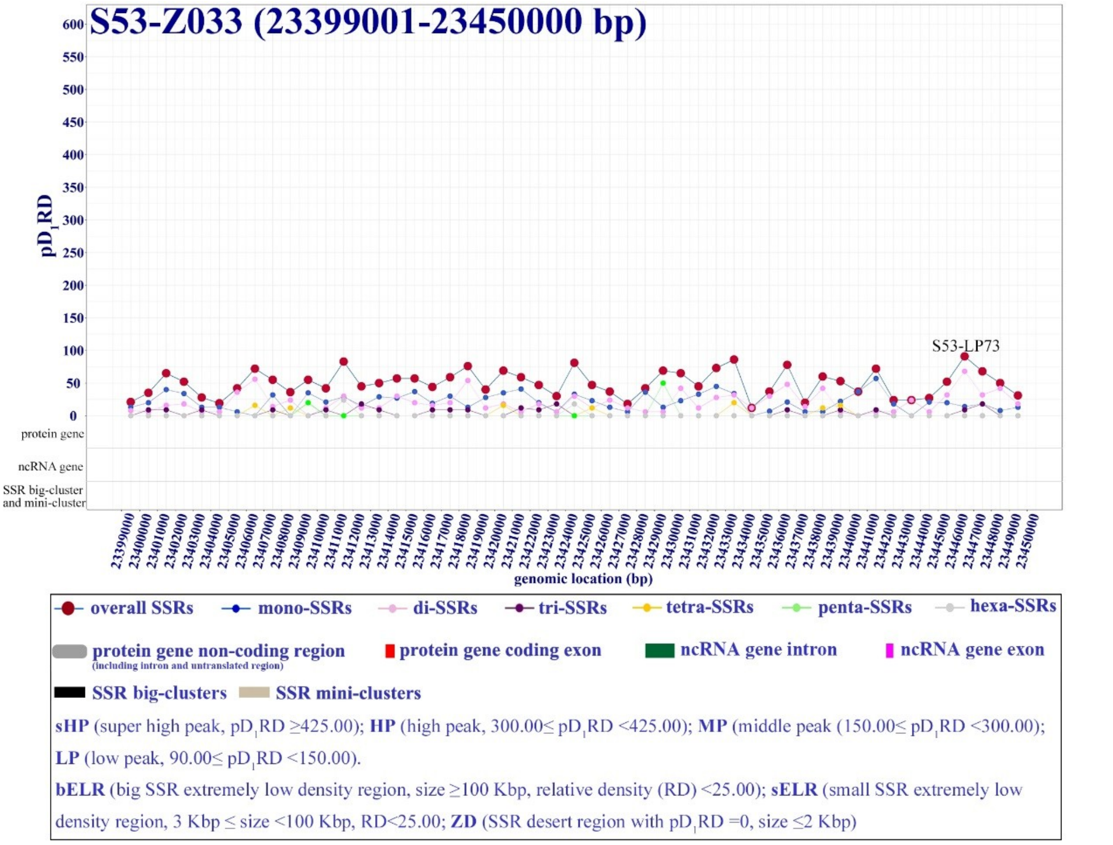

Supplementary Figure 1.463. The SSR position related D_1-relative density (pD_1RD) map of position at 23399001-23450000 bp of human reference Y-DNA (NC_000024.10) at resolution of 1 Kbp.

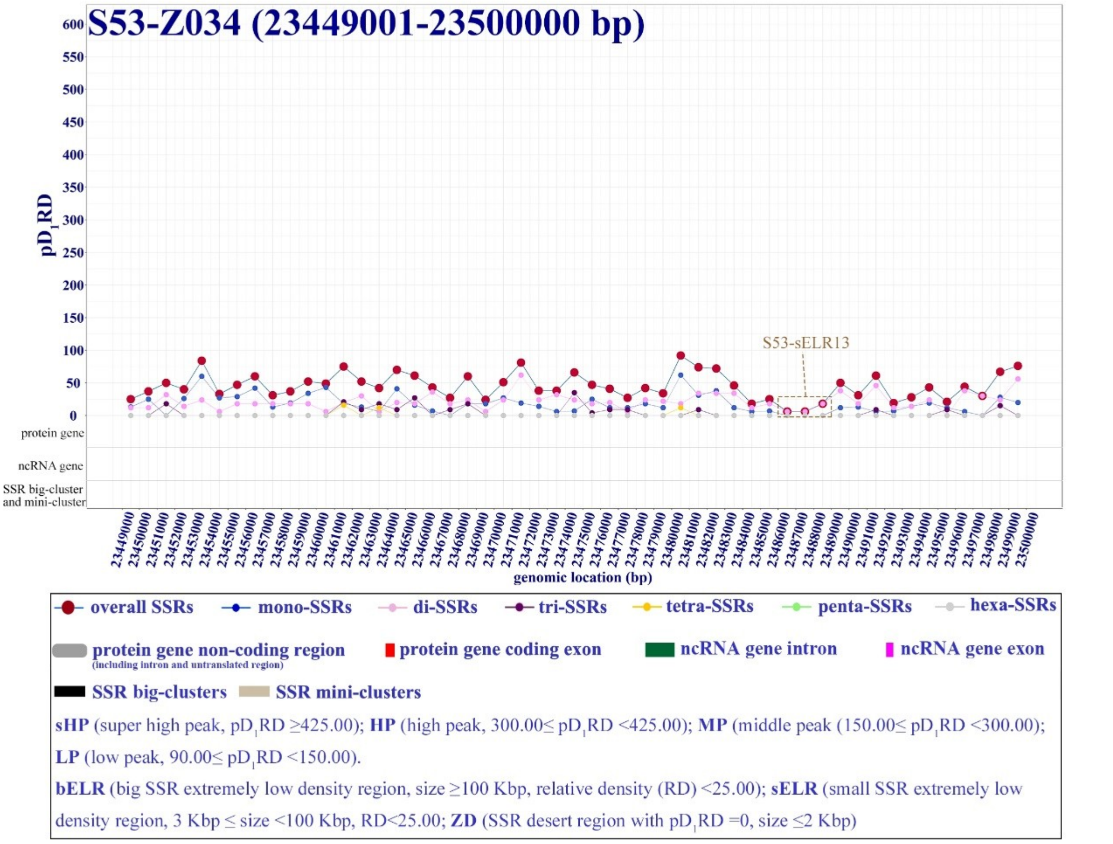

Supplementary Figure 1.464. The SSR position related D_1-relative density (pD_1RD) map of position at 23449001-23500000 bp of human reference Y-DNA (NC_000024.10) at resolution of 1 Kbp.

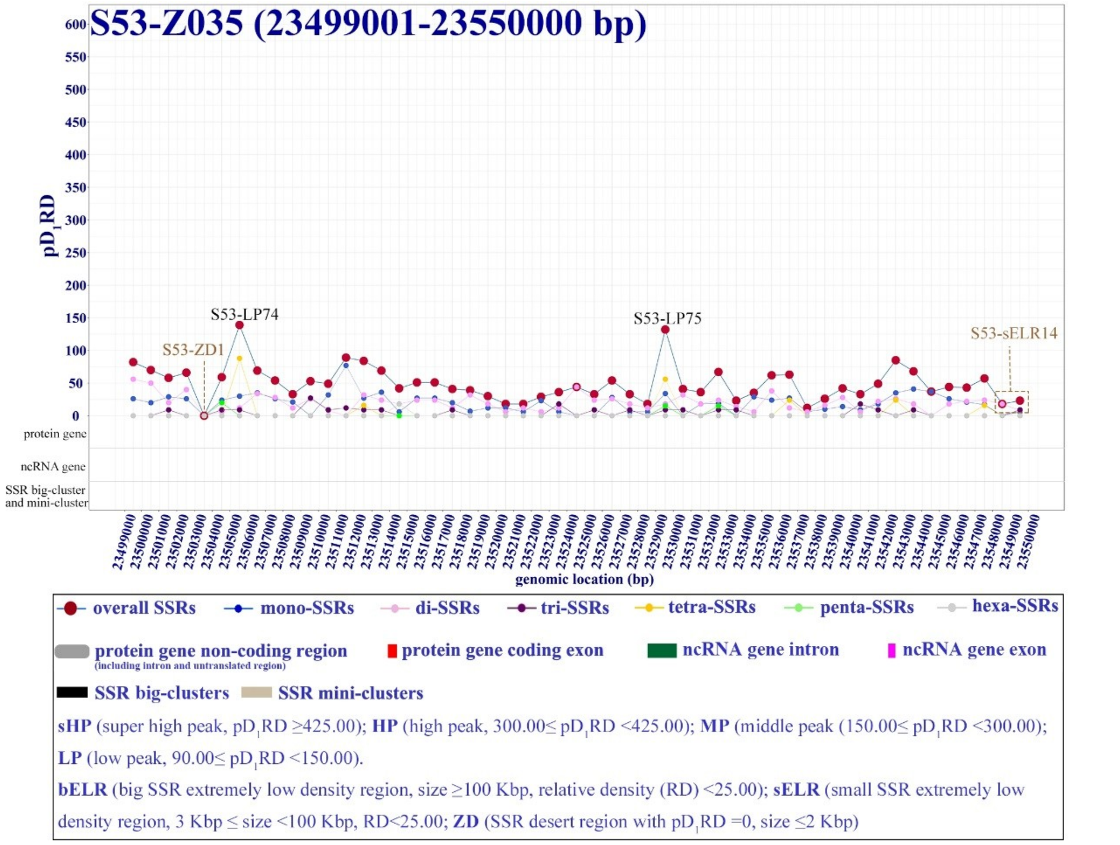

Supplementary Figure 1.465. The SSR position related D_1-relative density (pD_1RD) map of position at 23499001-23550000 bp of human reference Y-DNA (NC_000024.10) at resolution of 1 Kbp.

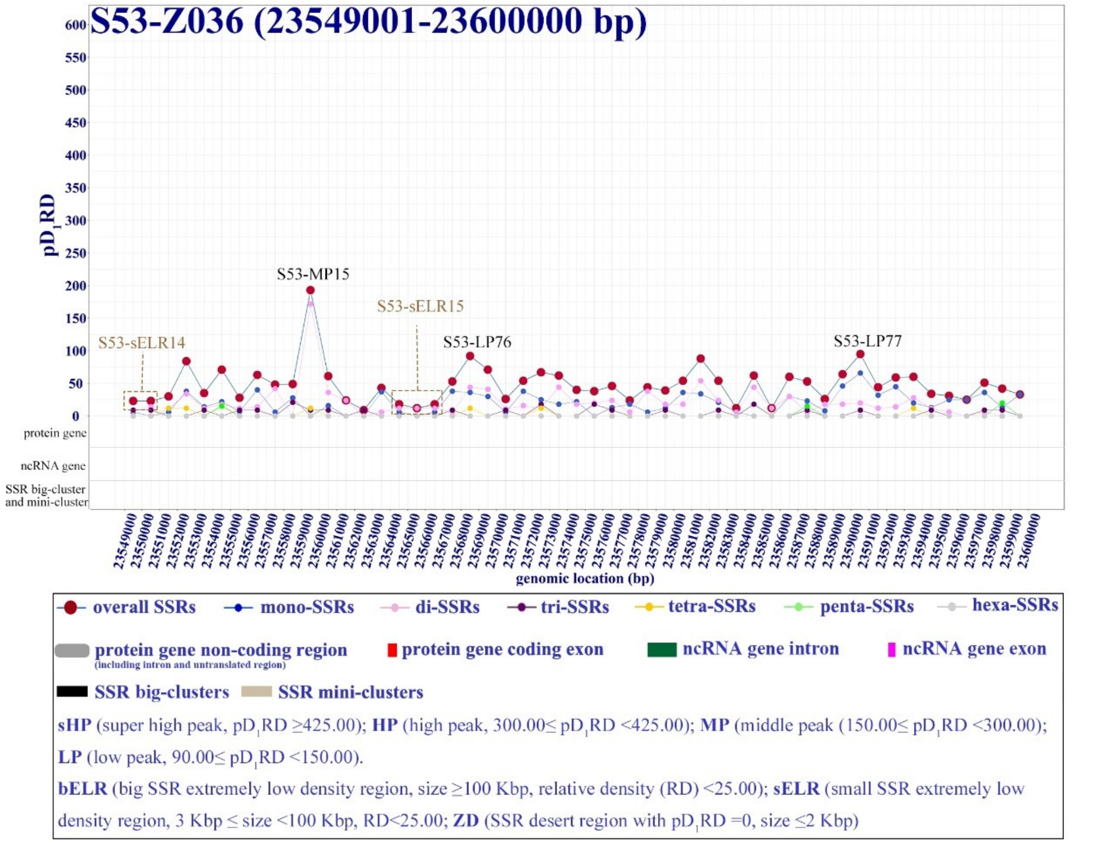

Supplementary Figure 1.466. The SSR position related D_1-relative density (pD_1RD) map of position at 23549001-23600000 bp of human reference Y-DNA (NC_000024.10) at resolution of 1 Kbp.

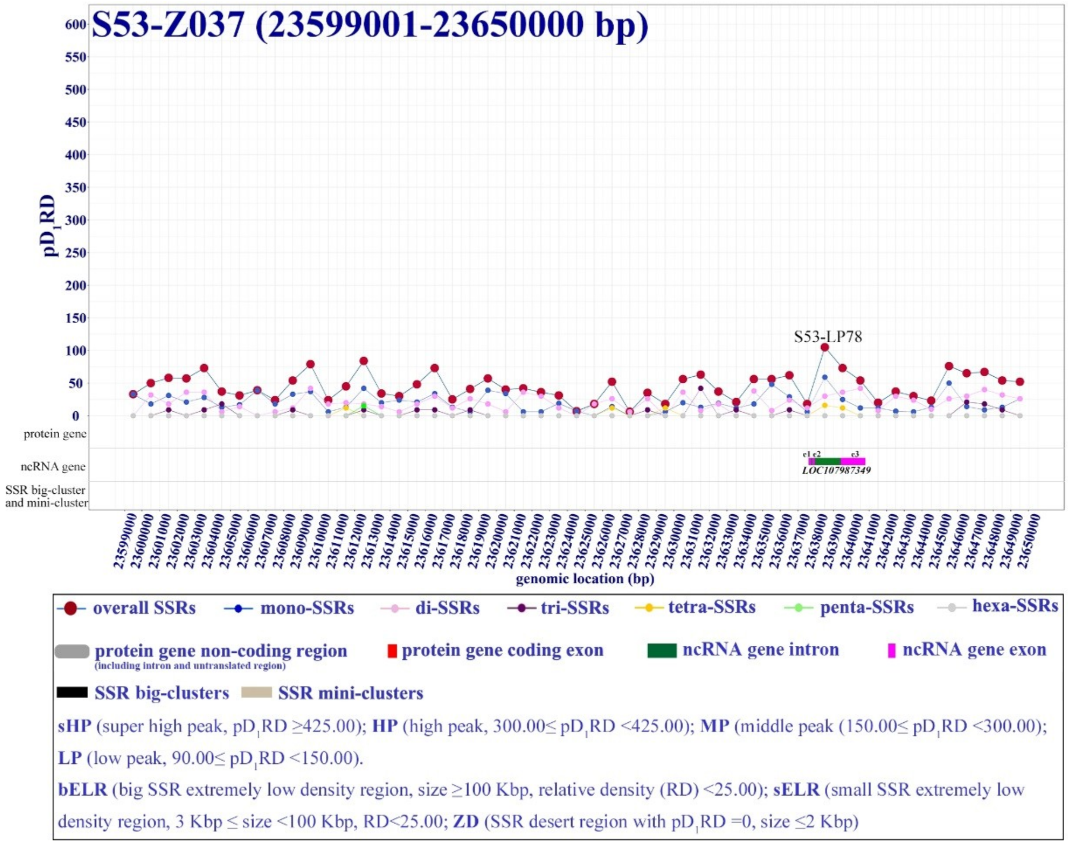

Supplementary Figure 1.467. The SSR position related D_1-relative density (pD_1RD) map of position at 23599001-23650000 bp of human reference Y-DNA (NC_000024.10) at resolution of 1 Kbp.

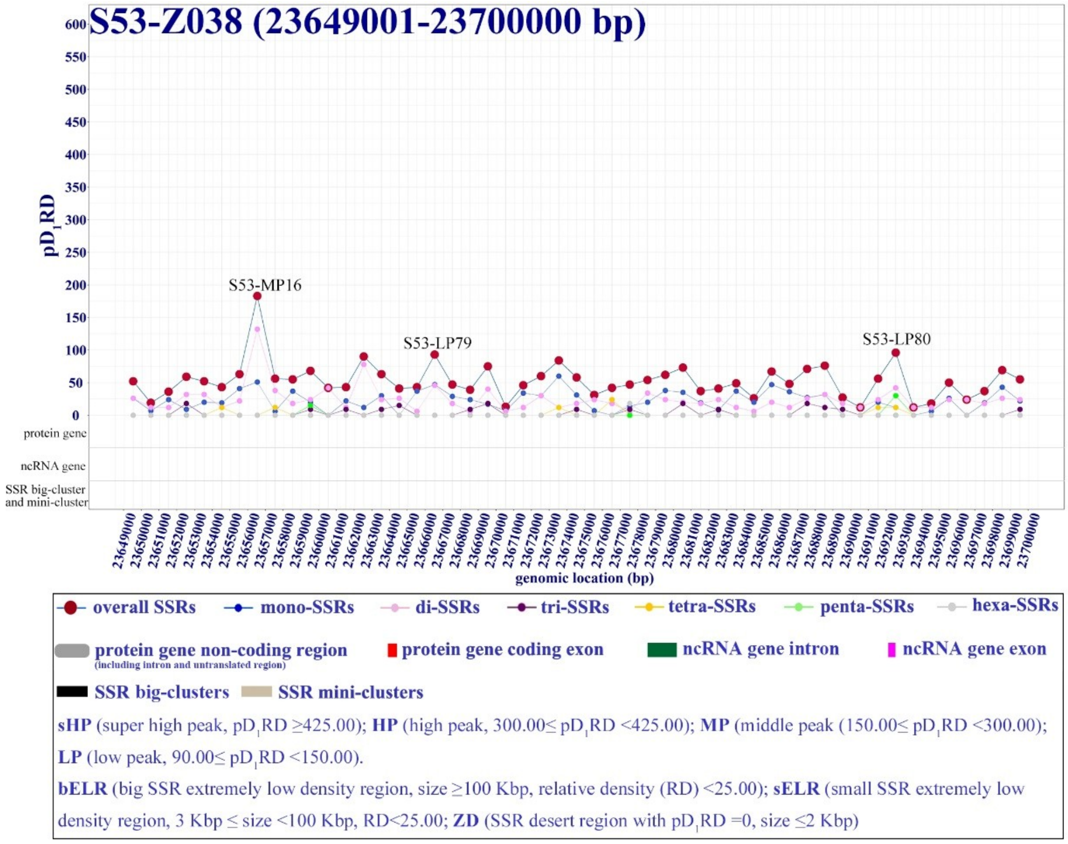

Supplementary Figure 1.468. The SSR position related D_1-relative density (pD_1RD) map of position at 23649001-23700000 bp of human reference Y-DNA (NC_000024.10) at resolution of 1 Kbp.

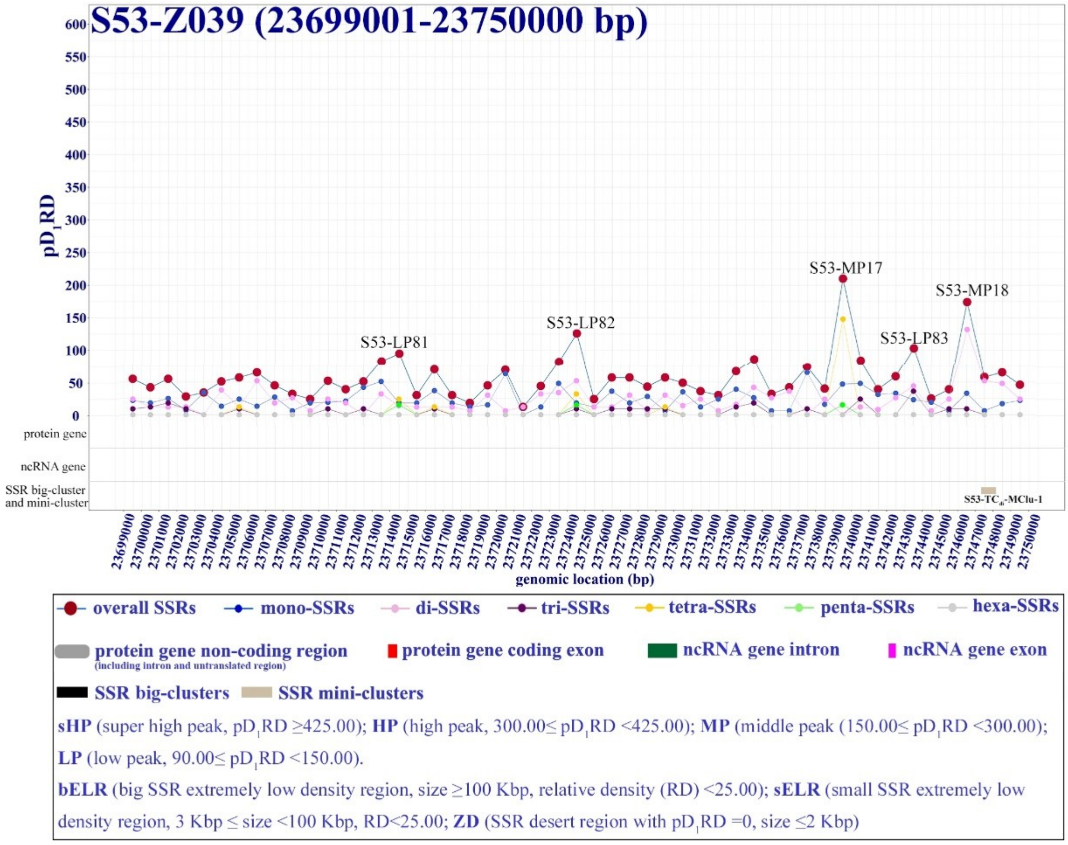

Supplementary Figure 1.469. The SSR position related D_1-relative density (pD_1RD) map of position at 23699001-23750000 bp of human reference Y-DNA (NC_000024.10) at resolution of 1 Kbp.

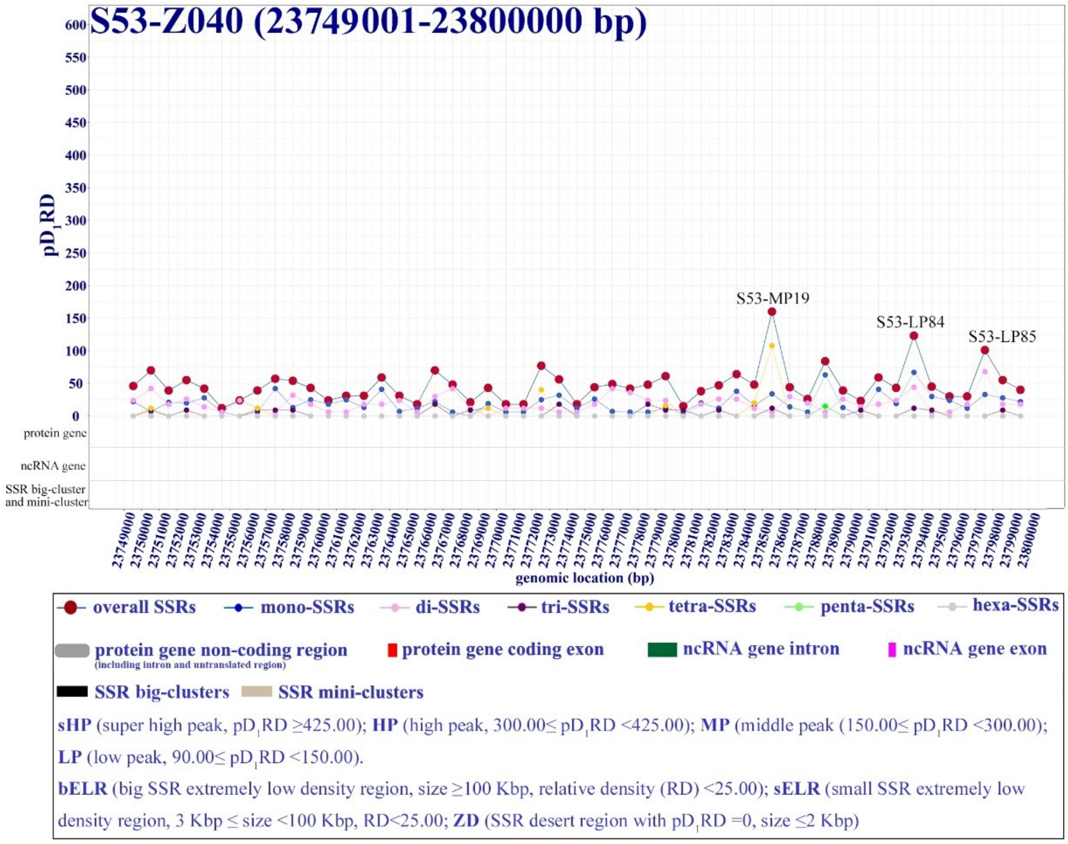

Supplementary Figure 1.470. The SSR position related D_1-relative density (pD_1RD) map of position at 23749001-23800000 bp of human reference Y-DNA (NC_000024.10) at resolution of 1 Kbp.

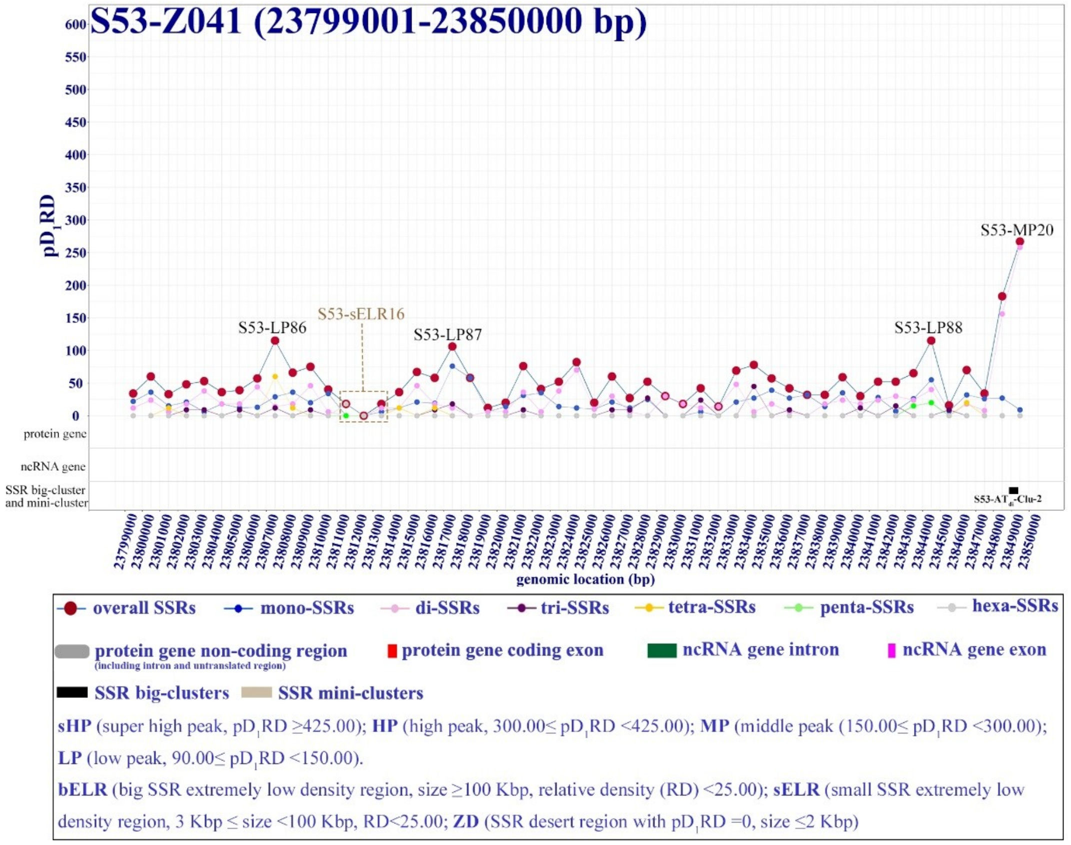

Supplementary Figure 1.471. The SSR position related D_1-relative density (pD_1RD) map of position at 23799001-23850000 bp of human reference Y-DNA (NC_000024.10) at resolution of 1 Kbp.

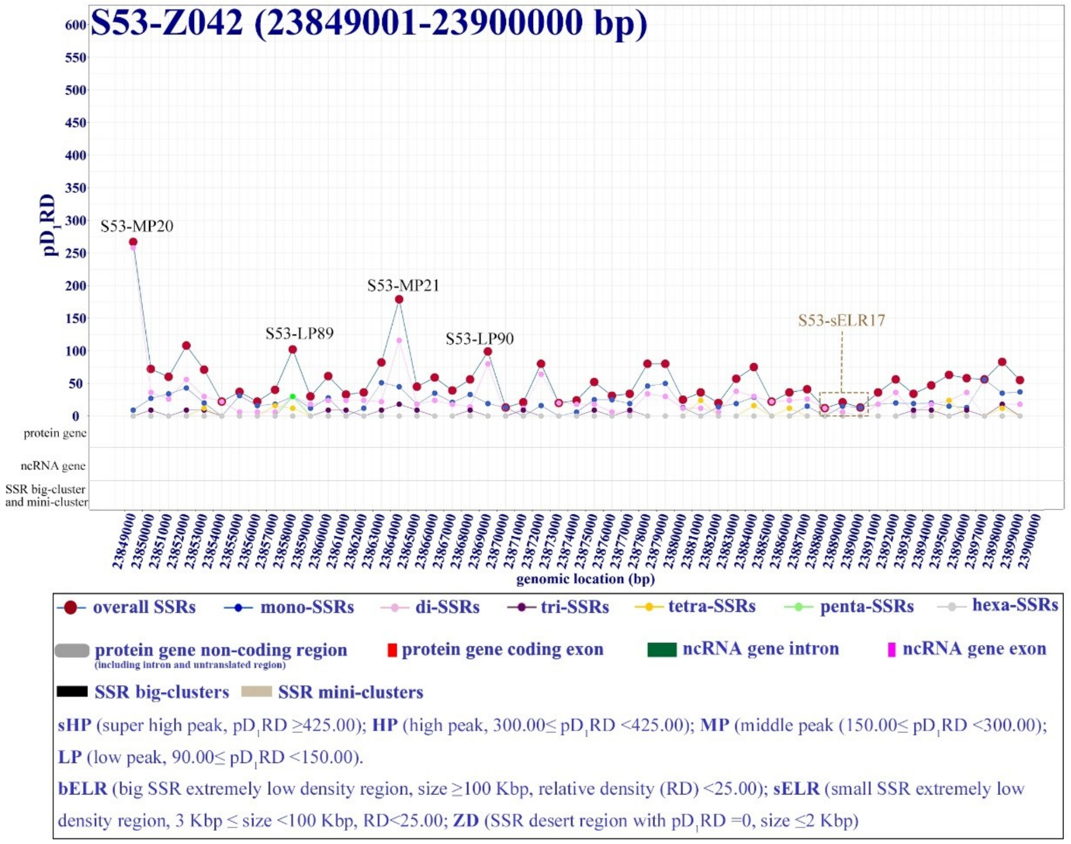

Supplementary Figure 1.472. The SSR position related D_1-relative density (pD_1RD) map of position at 23849001-23900000 bp of human reference Y-DNA (NC_000024.10) at resolution of 1 Kbp.

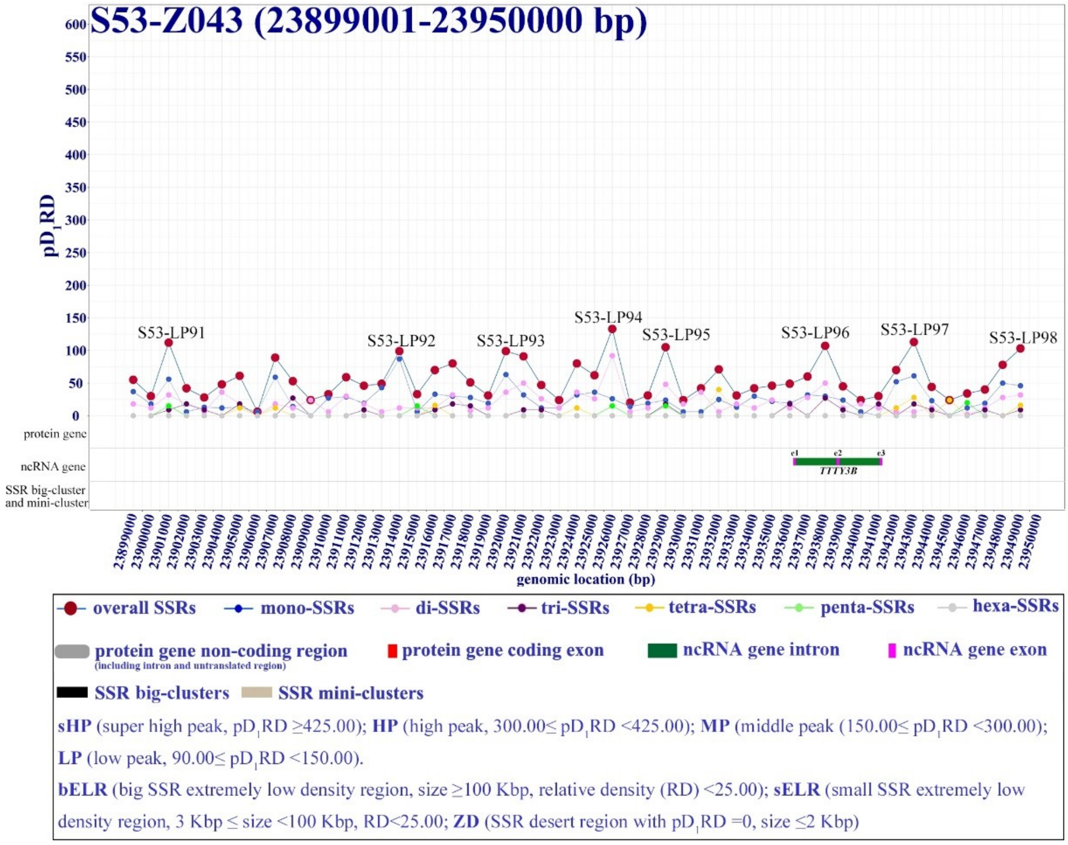

Supplementary Figure 1.473. The SSR position related D_1-relative density (pD_1RD) map of position at 23899001-23950000 bp of human reference Y-DNA (NC_000024.10) at resolution of 1 Kbp.

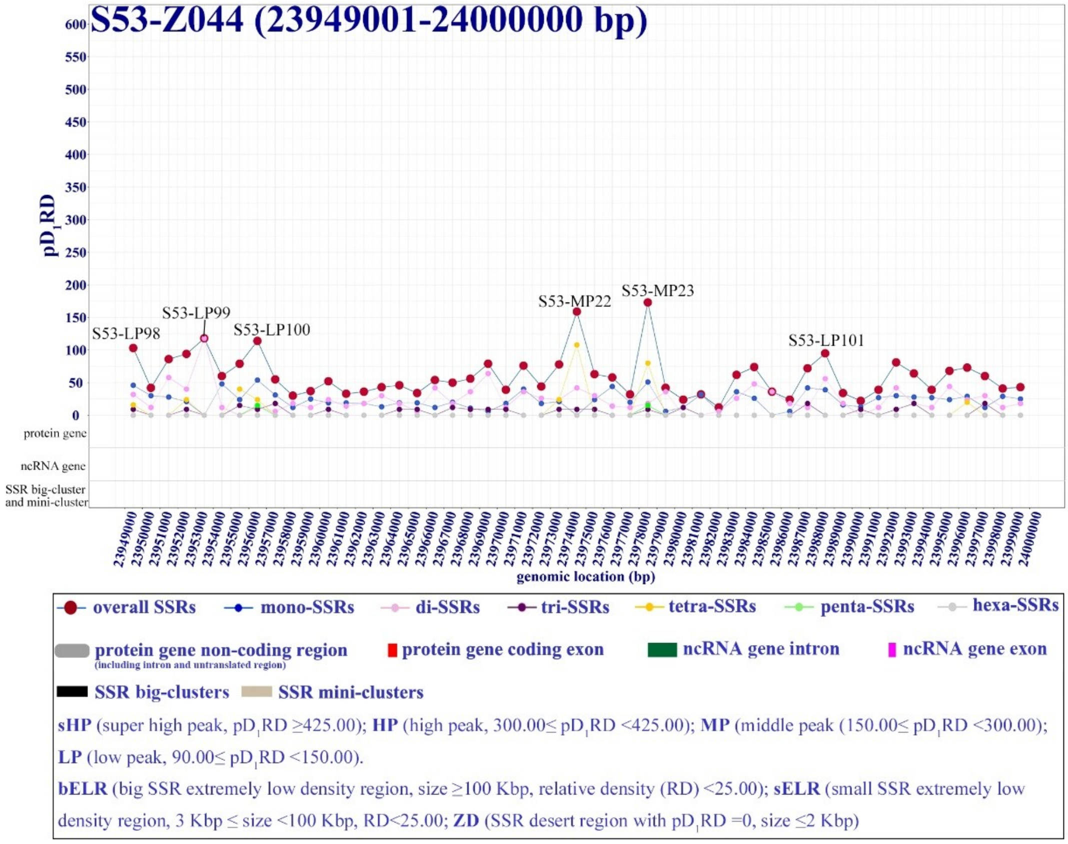

Supplementary Figure 1.474. The SSR position related D_1-relative density (pD_1RD) map of position at 23949001-24000000 bp of human reference Y-DNA (NC_000024.10) at resolution of 1 Kbp.

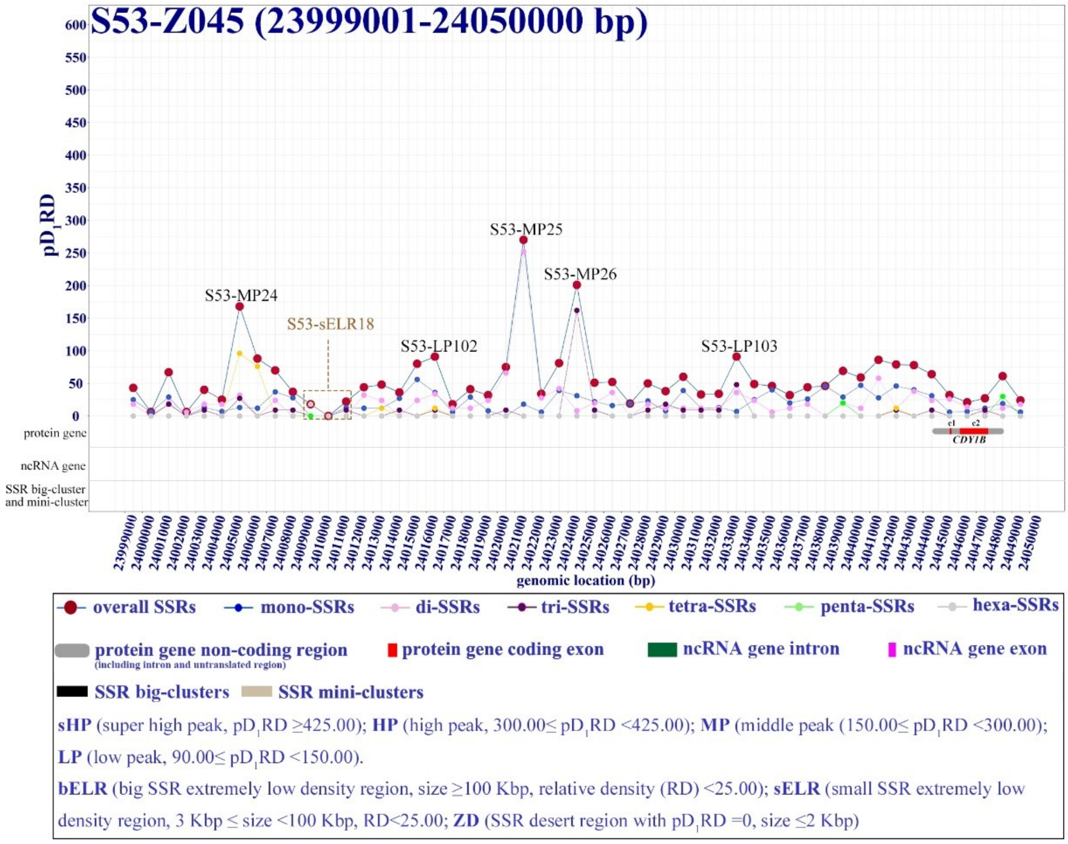

Supplementary Figure 1.475. The SSR position related D_1-relative density (pD_1RD) map of position at 23999001-24050000 bp of human reference Y-DNA (NC_000024.10) at resolution of 1 Kbp.

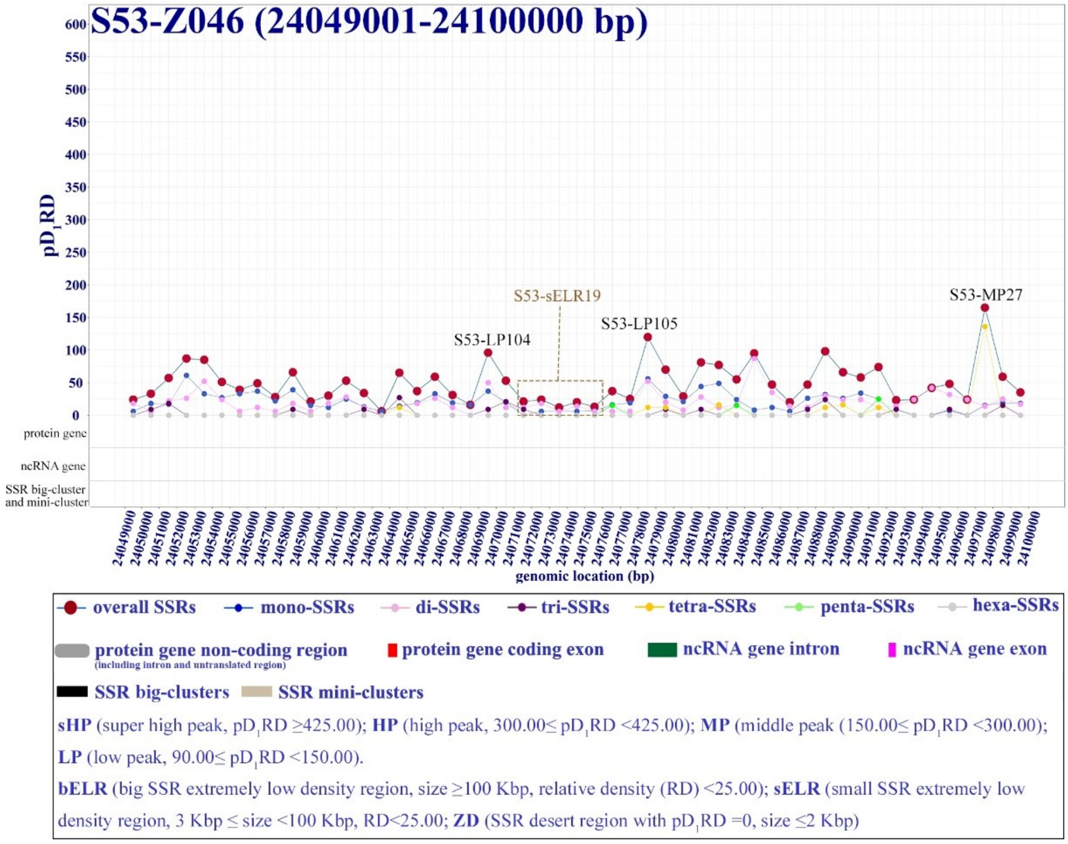

Supplementary Figure 1.476. The SSR position related D_1-relative density (pD_1RD) map of position at 24049001-24100000 bp of human reference Y-DNA (NC_000024.10) at resolution of 1 Kbp.

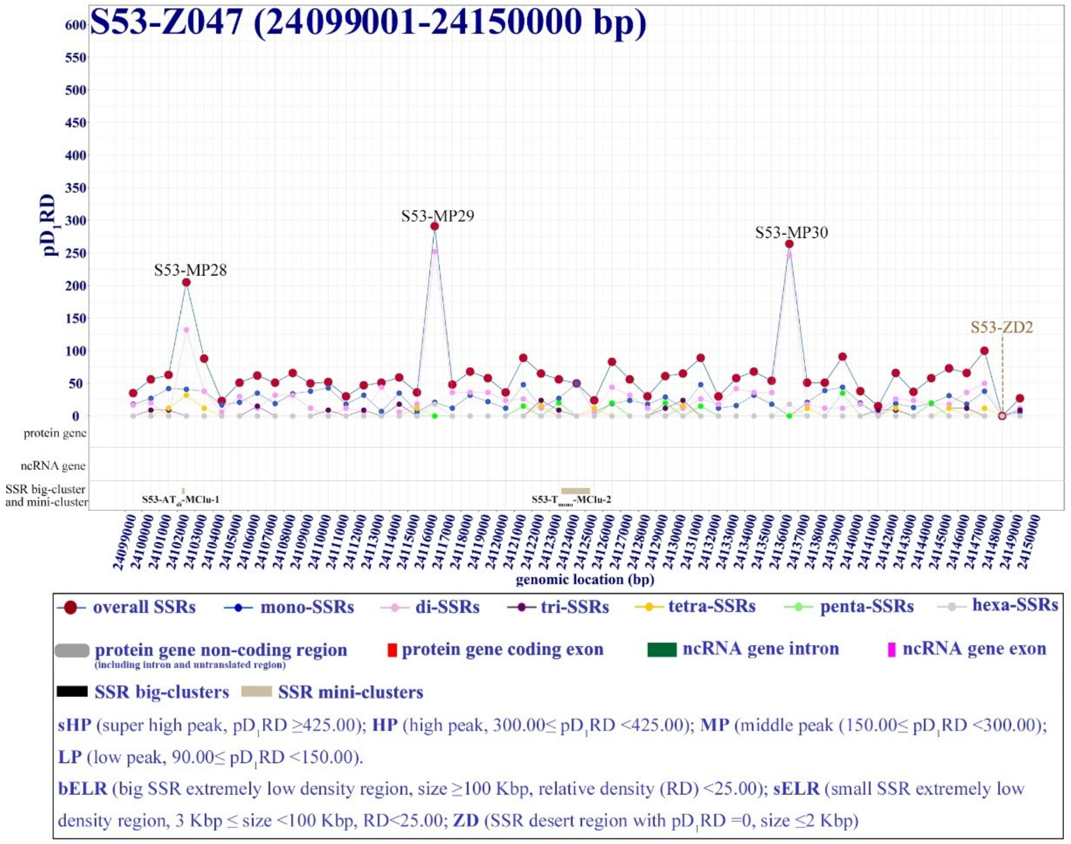

Supplementary Figure 1.477. The SSR position related D_1-relative density (pD_1RD) map of position at 24099001-24150000 bp of human reference Y-DNA (NC_000024.10) at resolution of 1 Kbp.

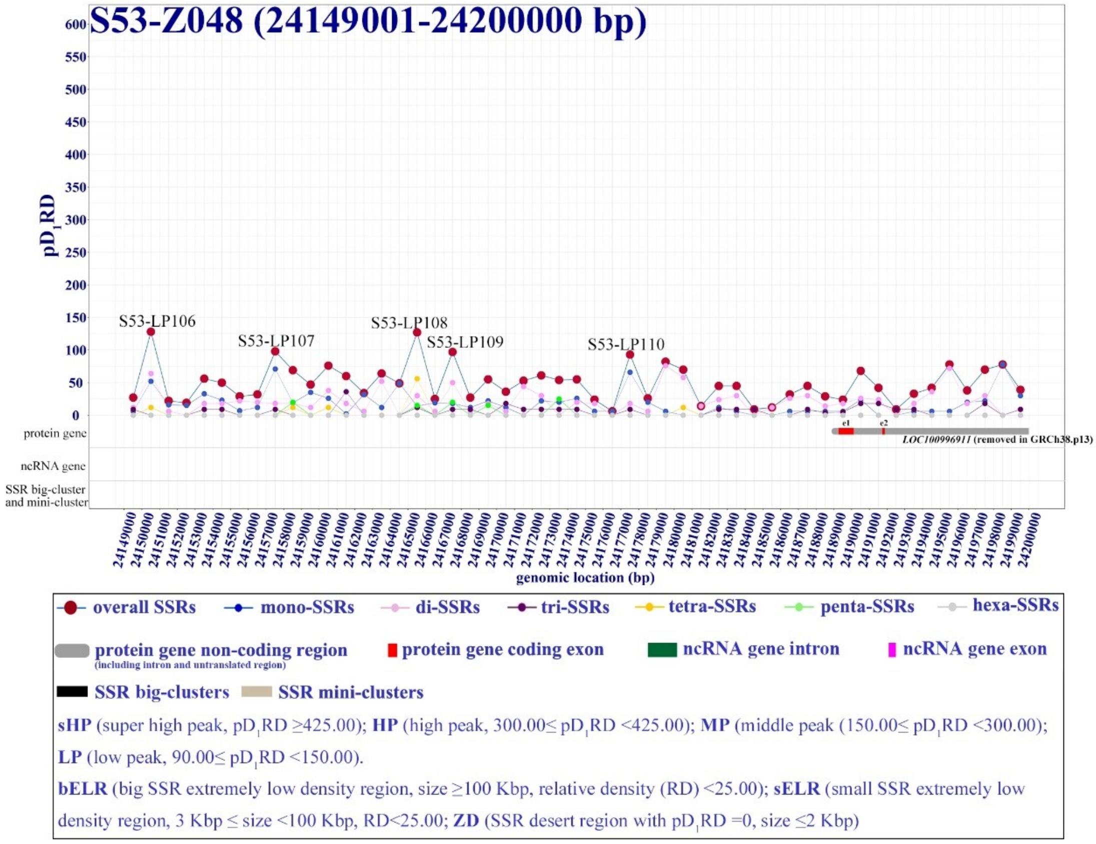

Supplementary Figure 1.478. The SSR position related D_1-relative density (pD_1RD) map of position at 24149001-24200000 bp of human reference Y-DNA (NC_000024.10) at resolution of 1 Kbp.

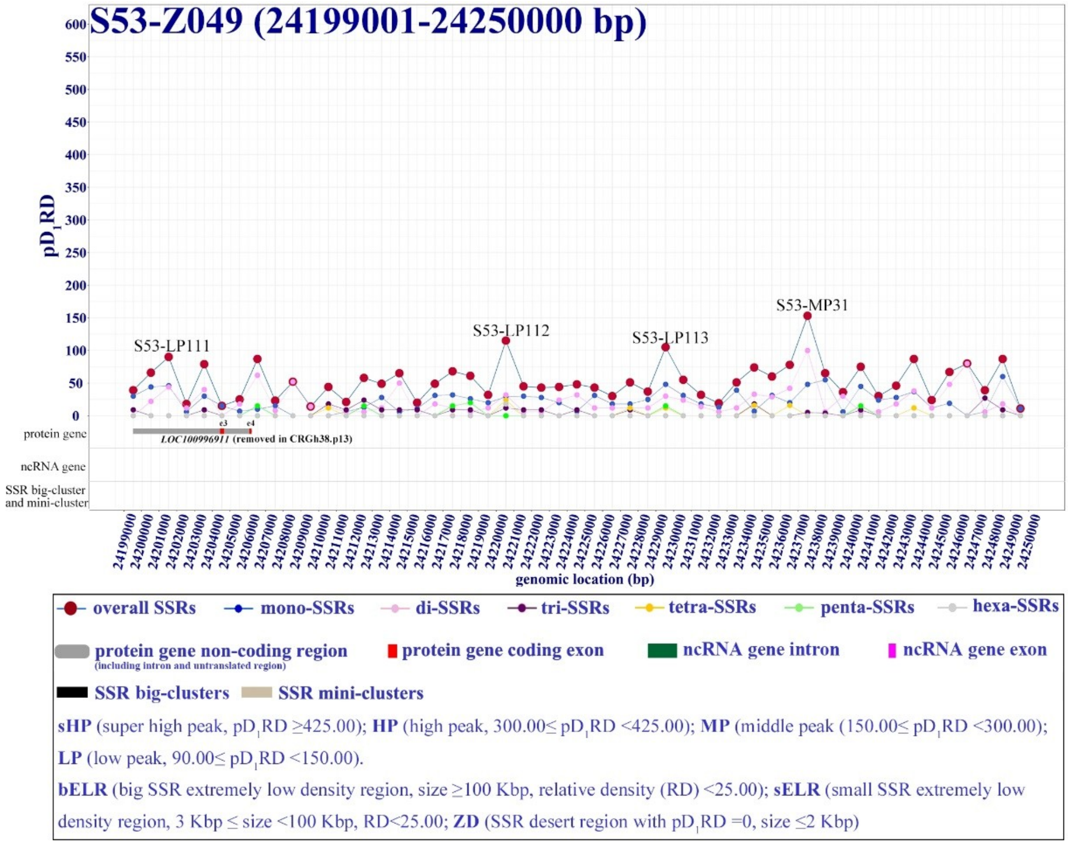

Supplementary Figure 1.479. The SSR position related D_1-relative density (pD_1RD) map of position at 24199001-24250000 bp of human reference Y-DNA (NC_000024.10) at resolution of 1 Kbp.

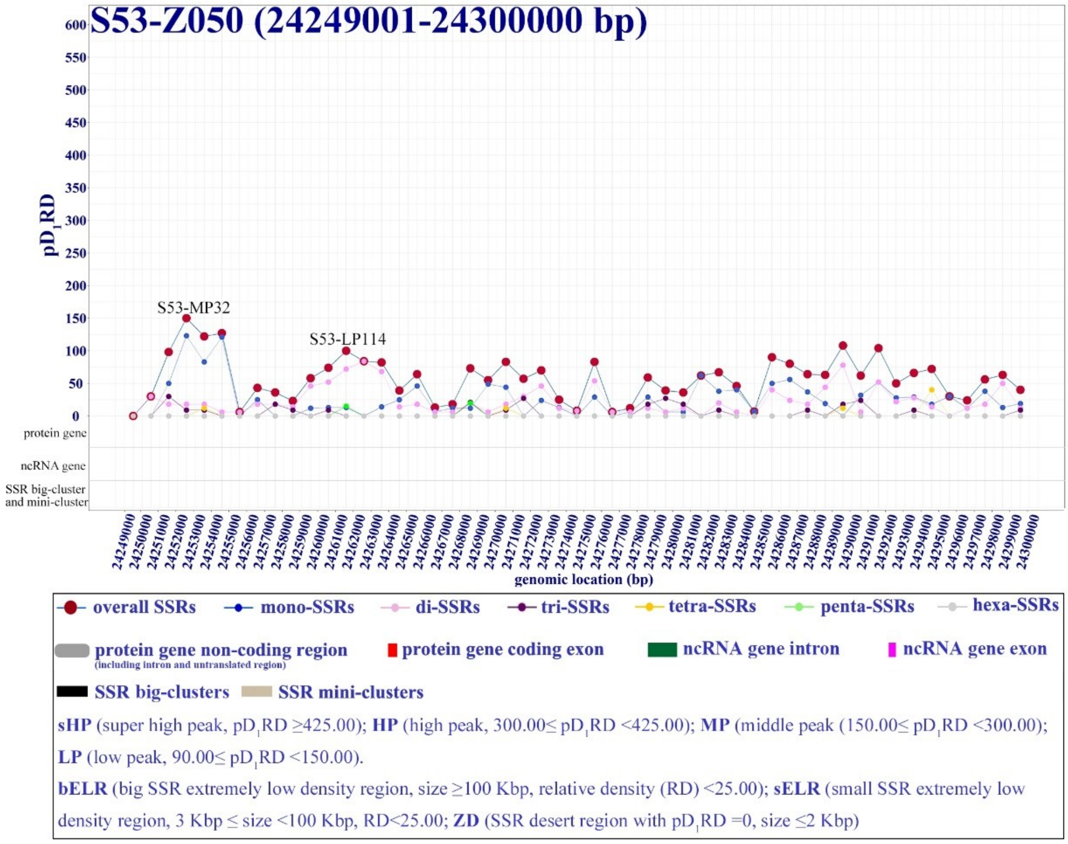

Supplementary Figure 1.480. The SSR position related D_1-relative density (pD_1RD) map of position at 24249001-24300000 bp of human reference Y-DNA (NC_000024.10) at resolution of 1 Kbp.

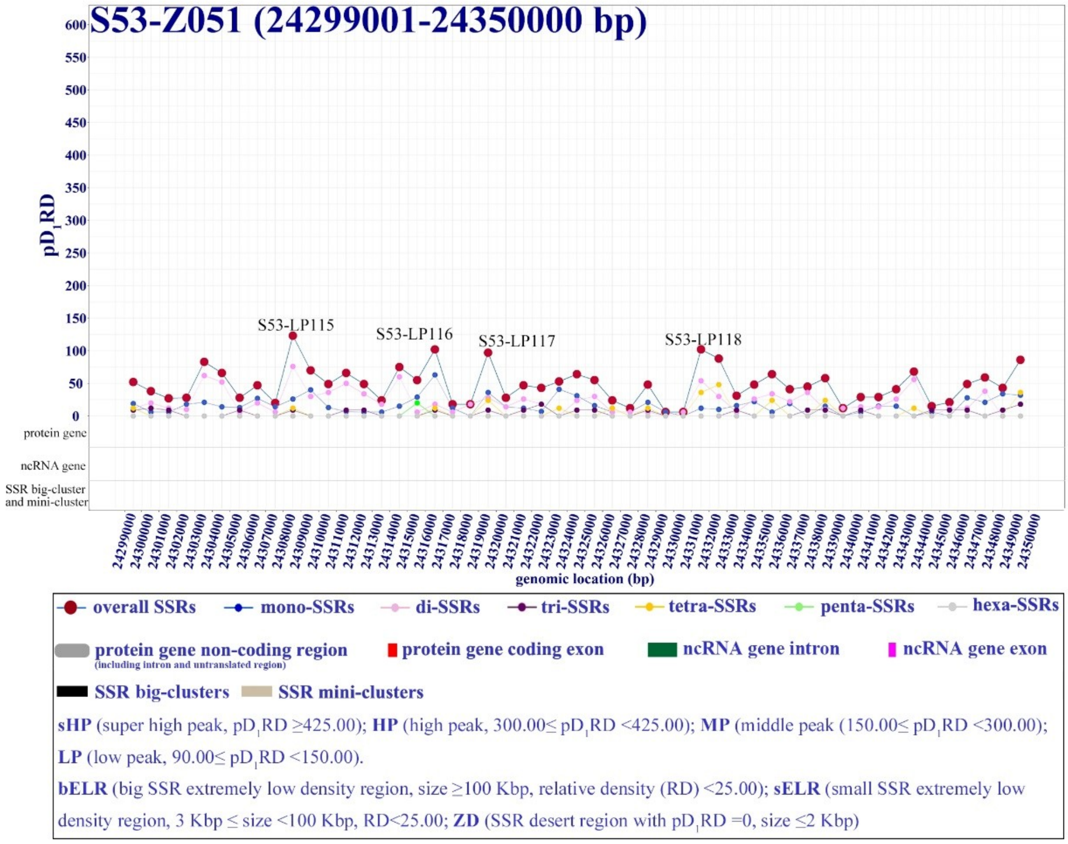

Supplementary Figure 1.481. The SSR position related D_1-relative density (pD_1RD) map of position at 24299001-24350000 bp of human reference Y-DNA (NC_000024.10) at resolution of 1 Kbp.

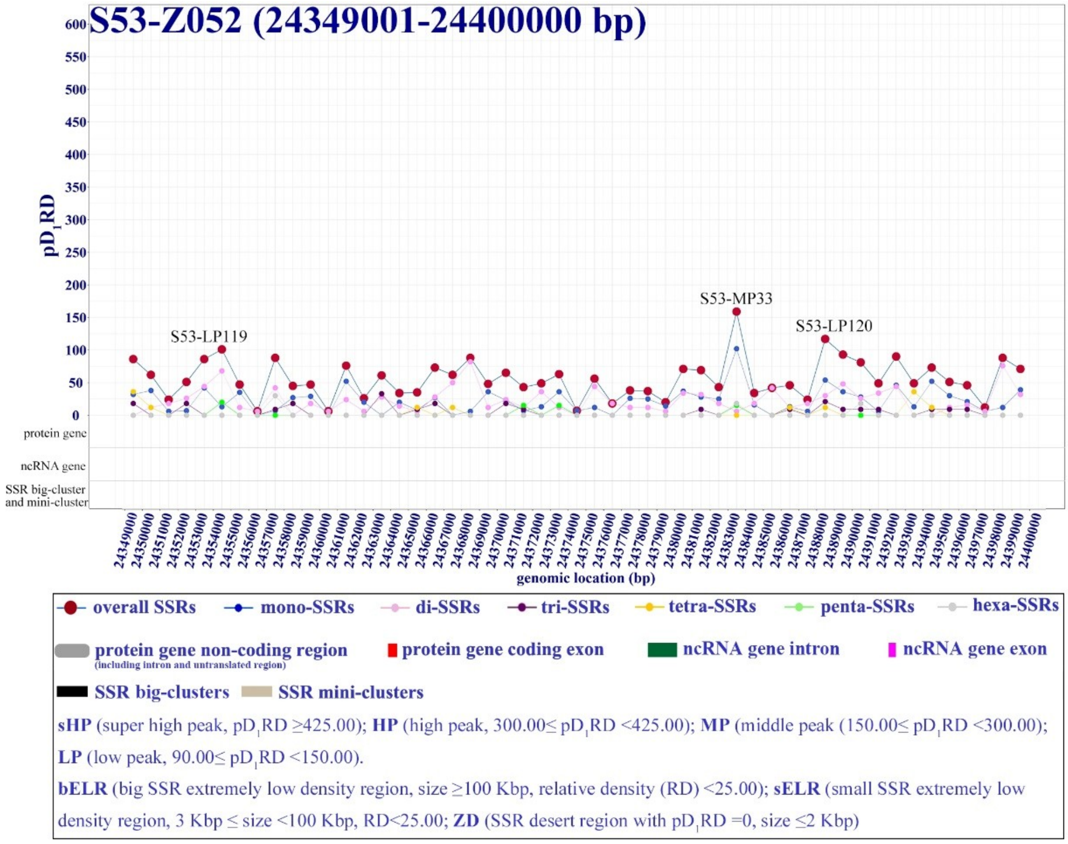

Supplementary Figure 1.482. The SSR position related D_1-relative density (pD_1RD) map of position at 24349001-24400000 bp of human reference Y-DNA (NC_000024.10) at resolution of 1 Kbp.

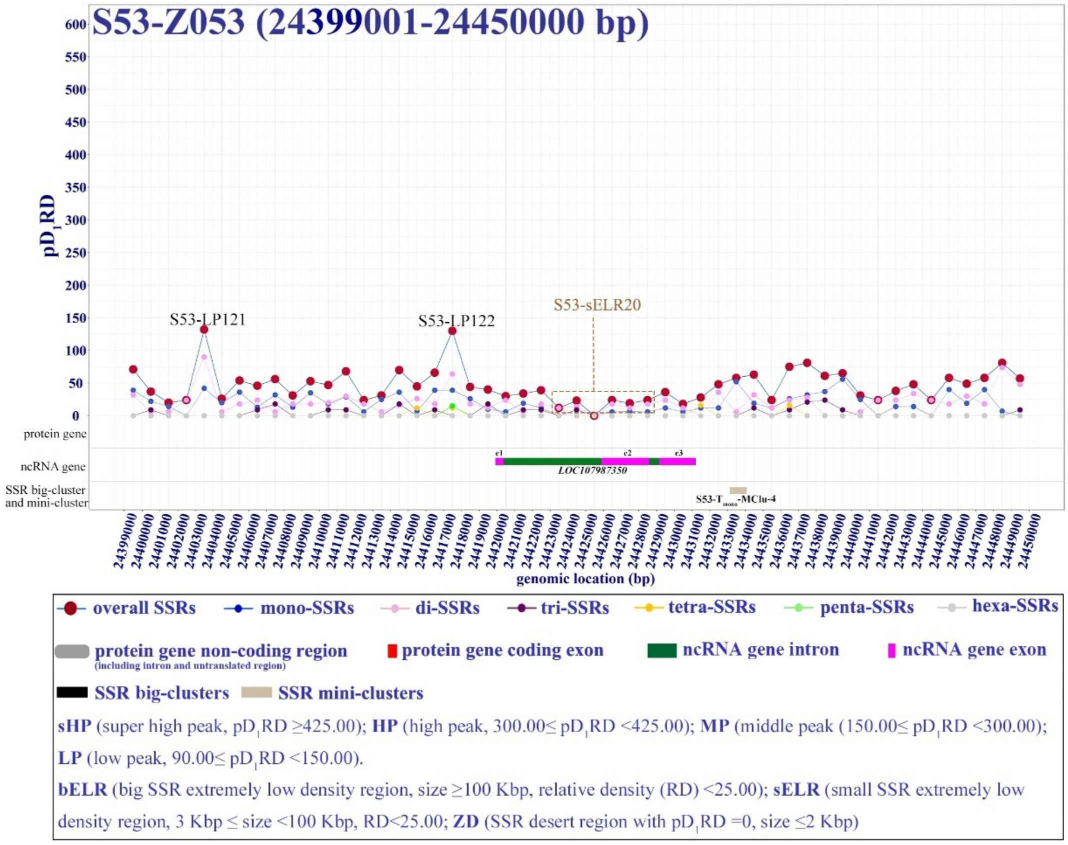

Supplementary Figure 1.483. The SSR position related D_1-relative density (pD_1RD) map of position at 24399001-24450000 bp of human reference Y-DNA (NC_000024.10) at resolution of 1 Kbp.

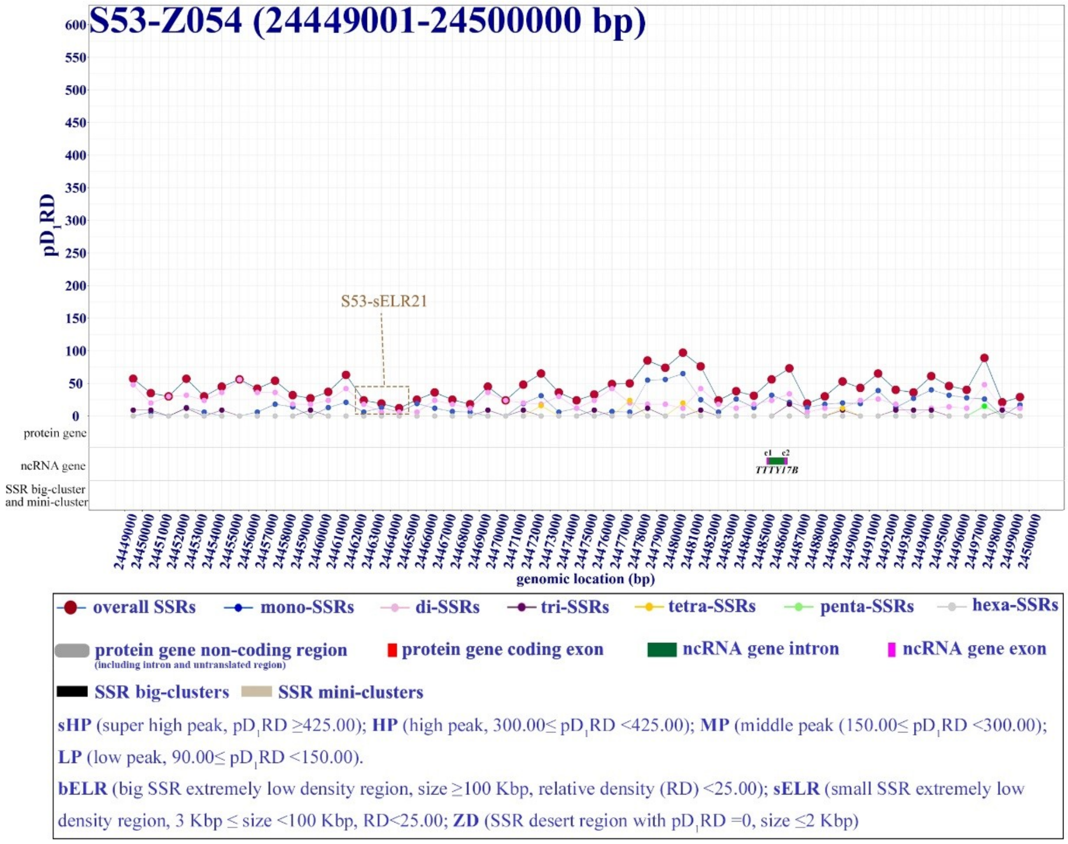

Supplementary Figure 1.484. The SSR position related D_1-relative density (pD_1RD) map of position at 24449001-24500000 bp of human reference Y-DNA (NC_000024.10) at resolution of 1 Kbp.

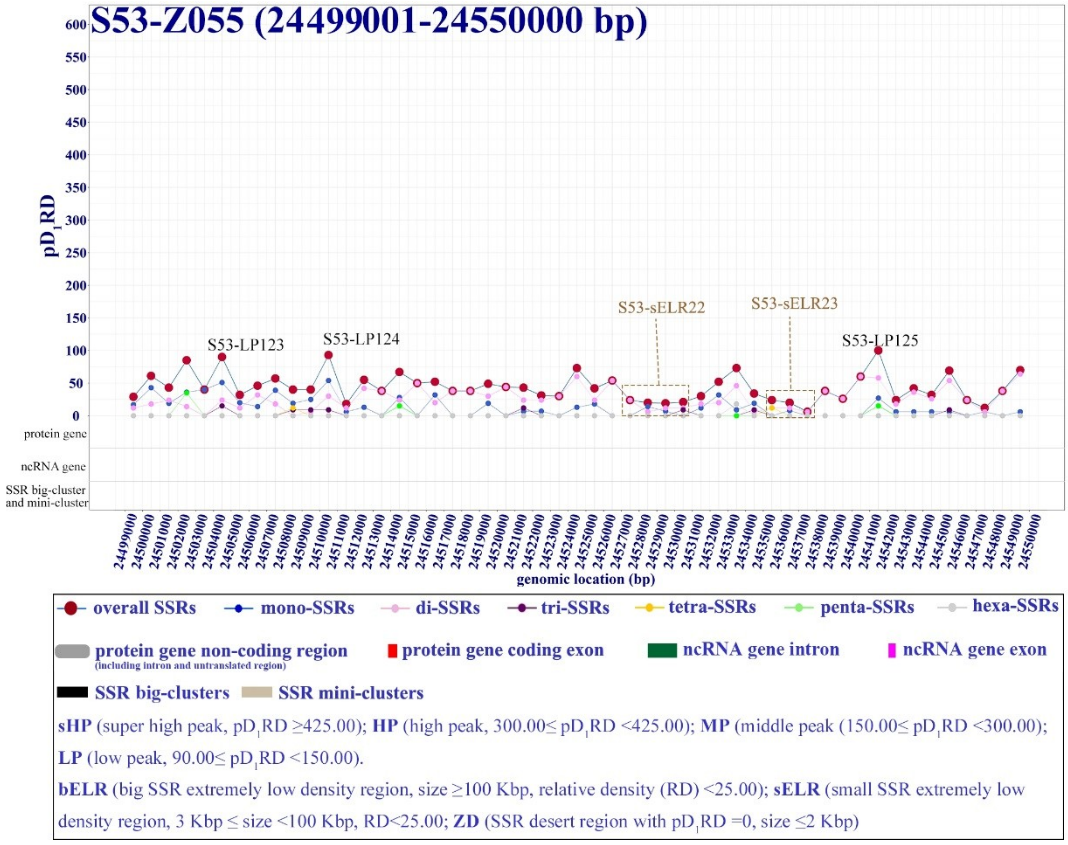

Supplementary Figure 1.485. The SSR position related D_1-relative density (pD_1RD) map of position at 24499001-24550000 bp of human reference Y-DNA (NC_000024.10) at resolution of 1 Kbp.

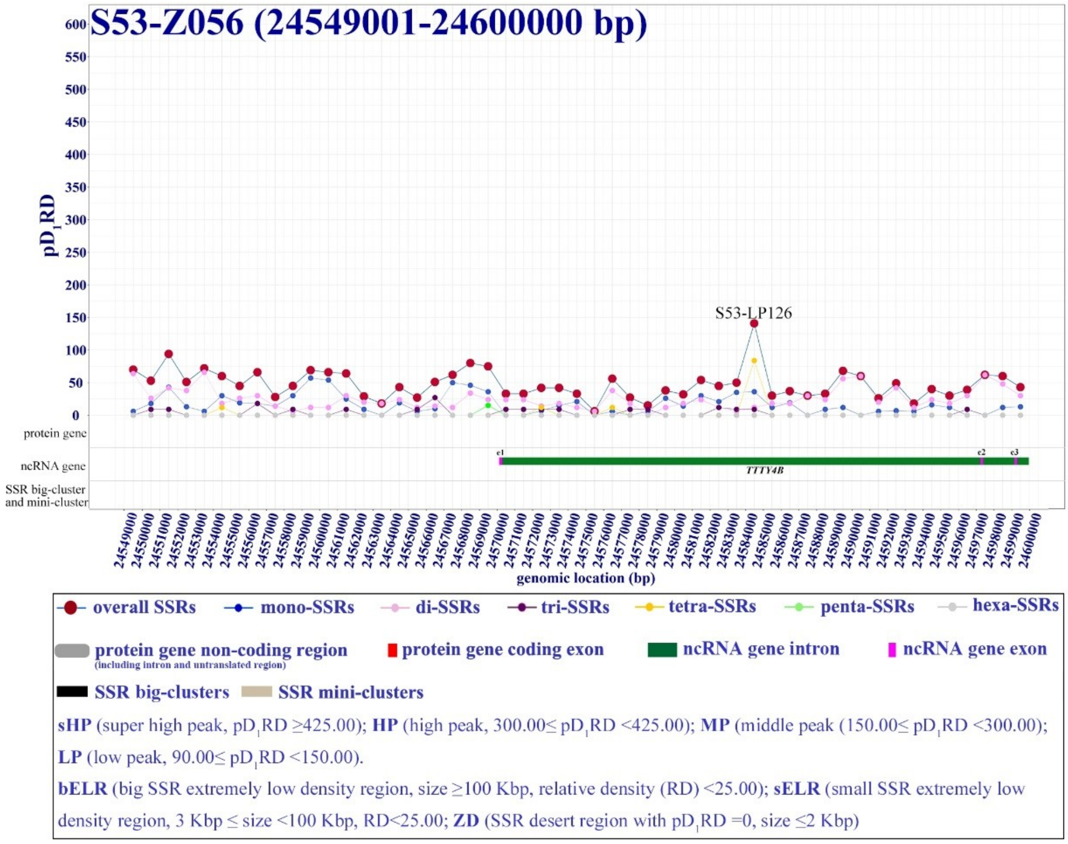

Supplementary Figure 1.486. The SSR position related D_1-relative density (pD_1RD) map of position at 24549001-24600000 bp of human reference Y-DNA (NC_000024.10) at resolution of 1 Kbp.

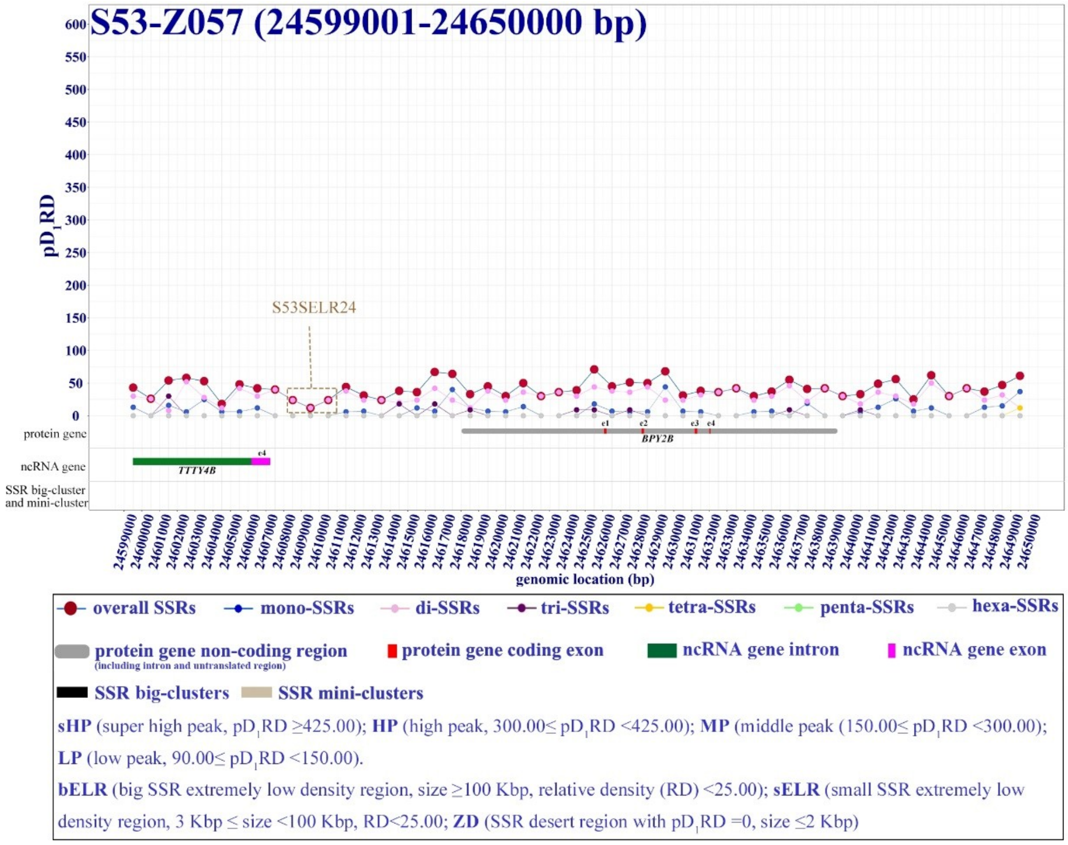

Supplementary Figure 1.487. The SSR position related D_1-relative density (pD_1RD) map of position at 24599001-24650000 bp of human reference Y-DNA (NC_000024.10) at resolution of 1 Kbp.

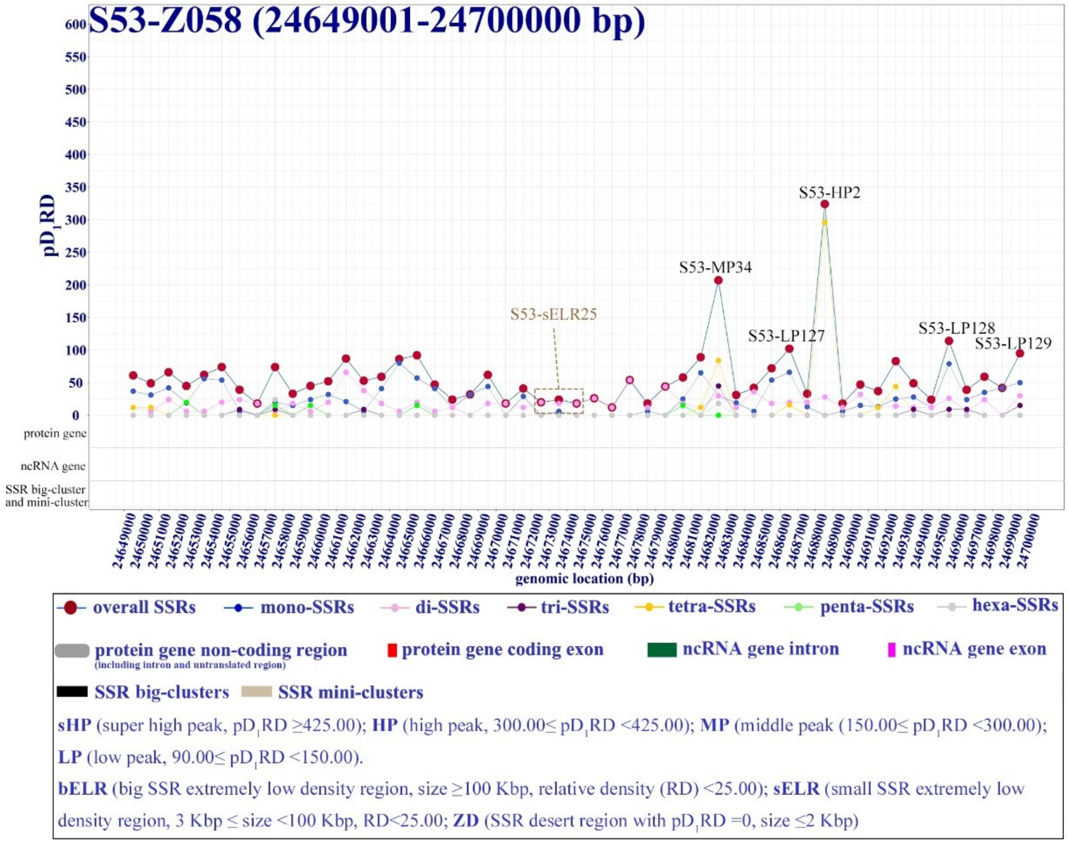

Supplementary Figure 1.488. The SSR position related D_1-relative density (pD_1RD) map of position at 24649001-24700000 bp of human reference Y-DNA (NC_000024.10) at resolution of 1 Kbp.

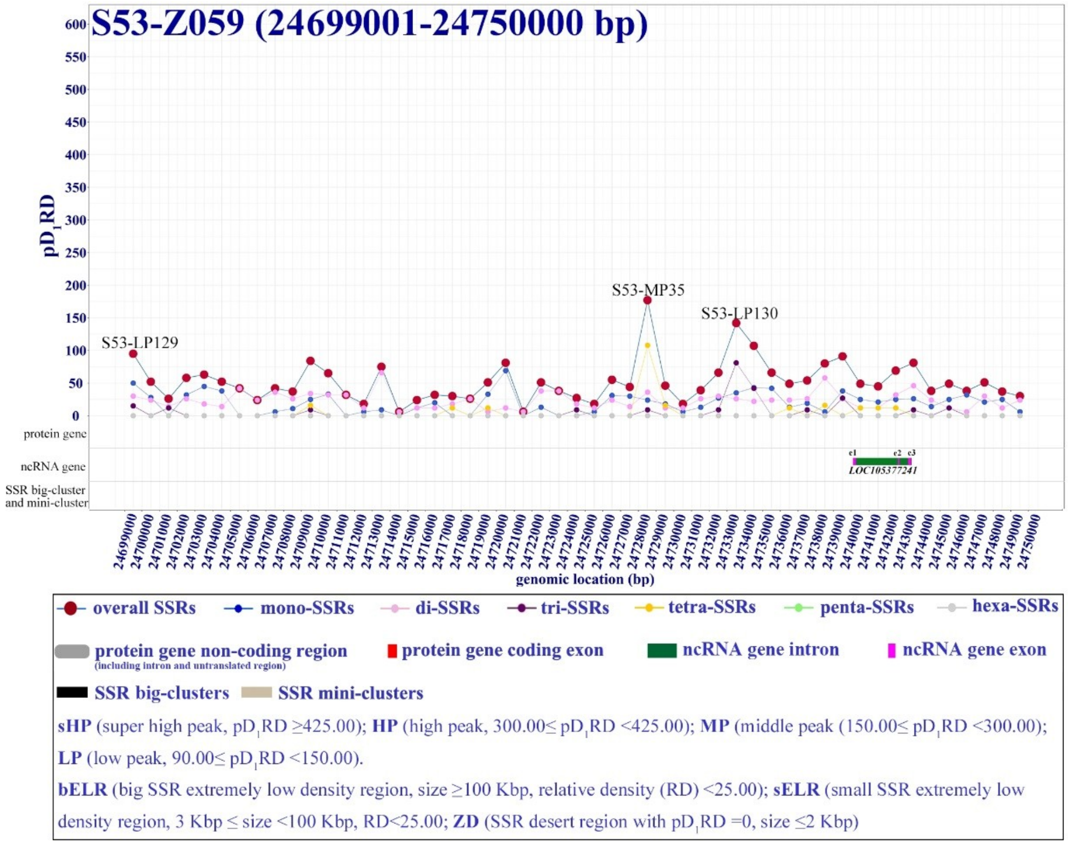

Supplementary Figure 1.489. The SSR position related D_1-relative density (pD_1RD) map of position at 24699001-24750000 bp of human reference Y-DNA (NC_000024.10) at resolution of 1 Kbp.

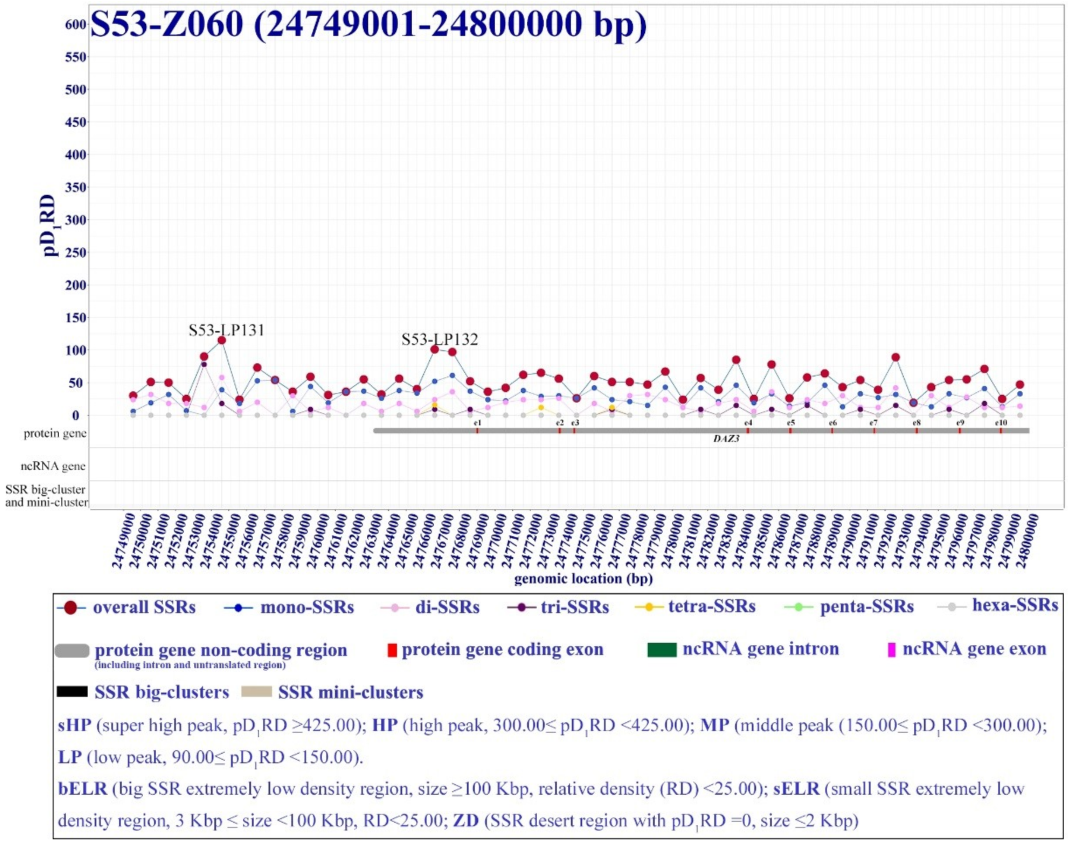

Supplementary Figure 1.490. The SSR position related D_1-relative density (pD_1RD) map of position at 24749001-24800000 bp of human reference Y-DNA (NC_000024.10) at resolution of 1 Kbp.

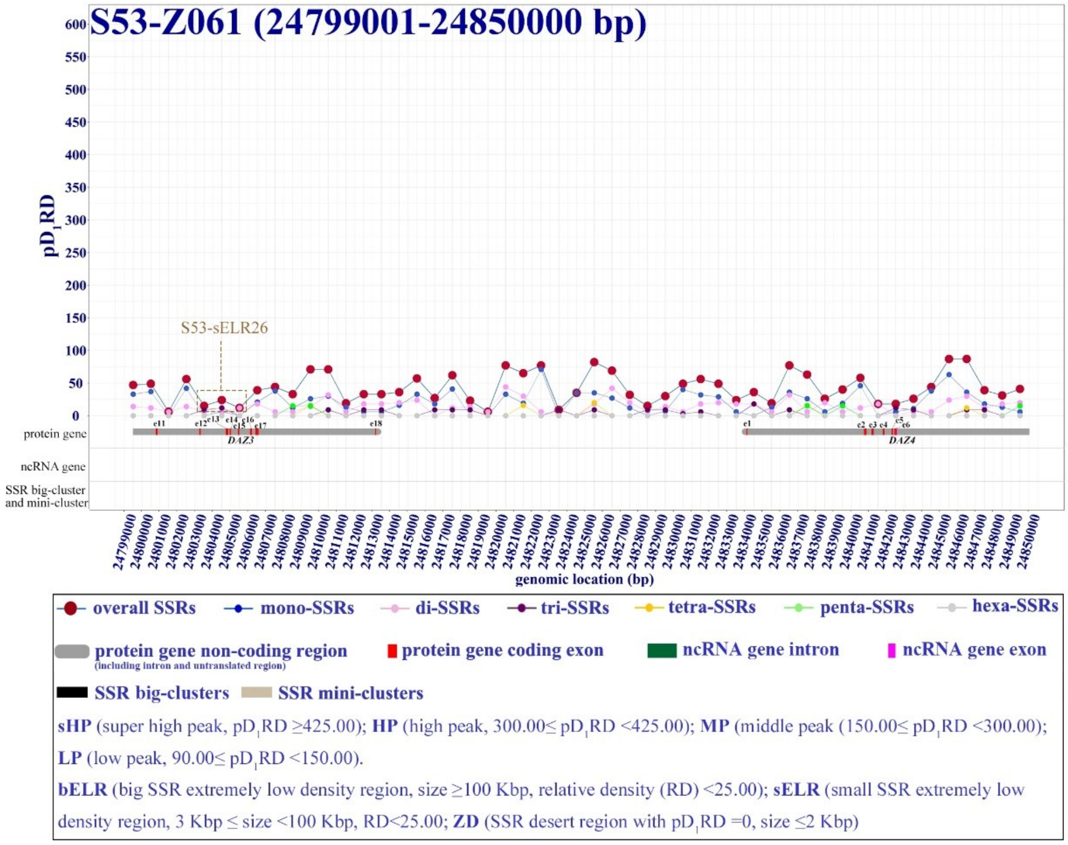

Supplementary Figure 1.491. The SSR position related D_1-relative density (pD_1RD) map of position at 24799001-24850000 bp of human reference Y-DNA (NC_000024.10) at resolution of 1 Kbp.

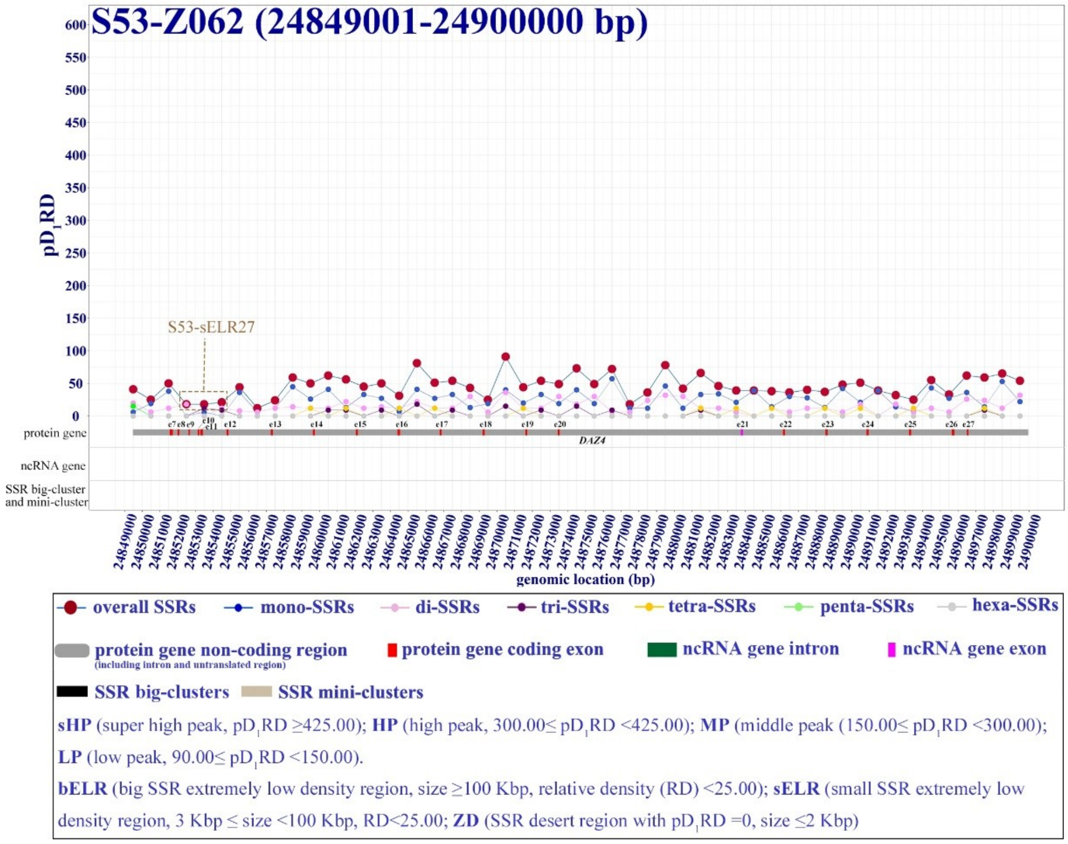

Supplementary Figure 1.492. The SSR position related D_1-relative density (pD_1RD) map of position at 24849001-24900000 bp of human reference Y-DNA (NC_000024.10) at resolution of 1 Kbp at resolution of 1 Kbp.

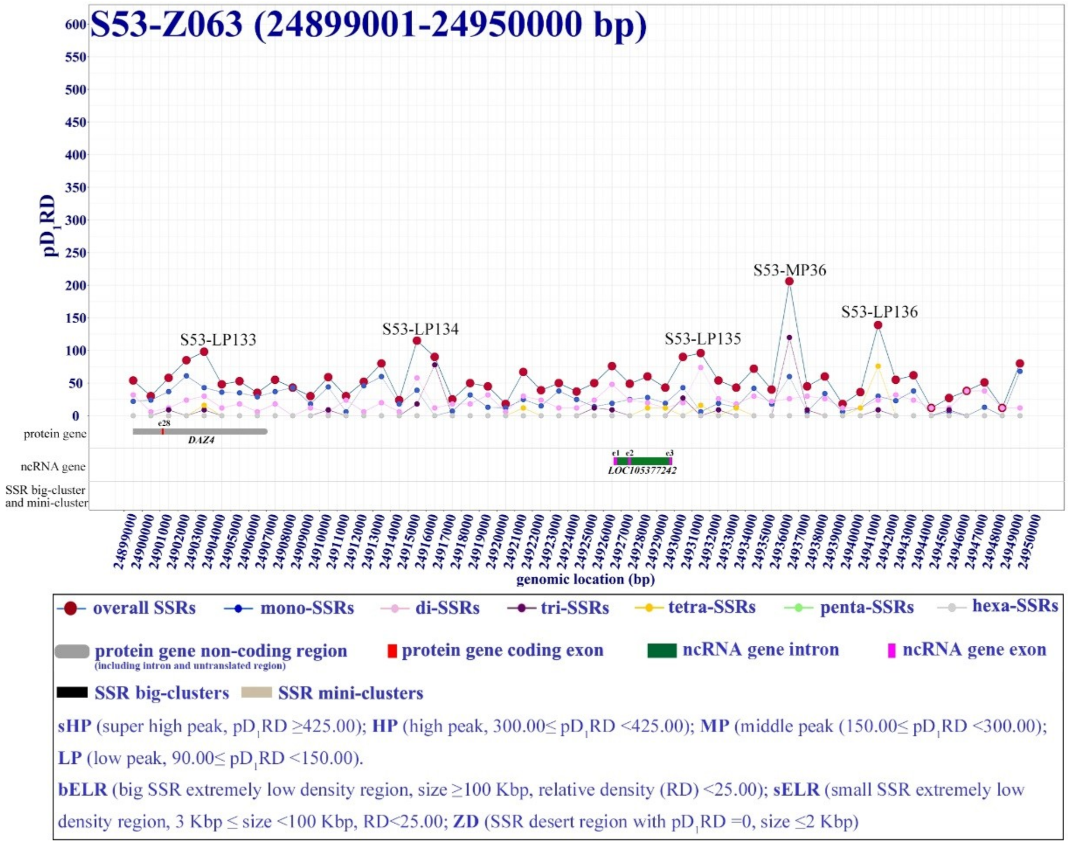

Supplementary Figure 1.493. The SSR position related D_1-relative density (pD_1RD) map of position at 24899001-24950000 bp of human reference Y-DNA (NC_000024.10) at resolution of 1 Kbp.

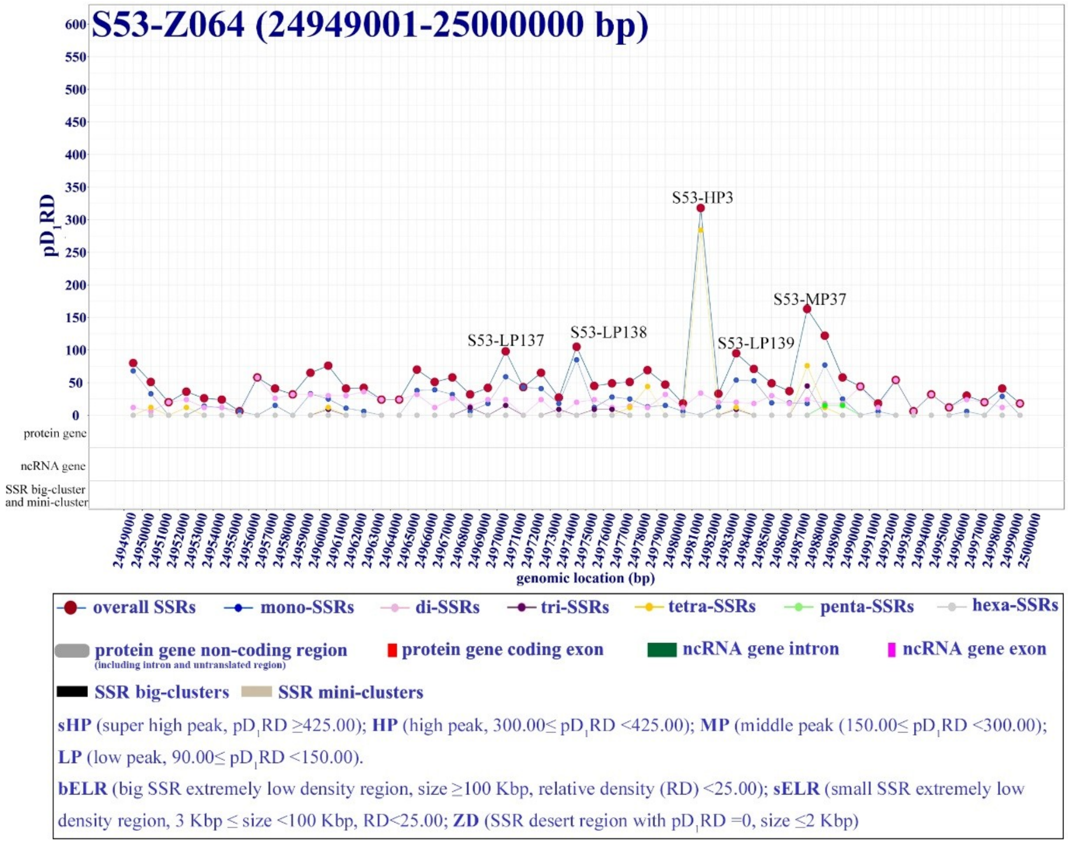

Supplementary Figure 1.494. The SSR position related D_1-relative density (pD_1RD) map of position at 24949001-25000000 bp of human reference Y-DNA (NC_000024.10) at resolution of 1 Kbp.

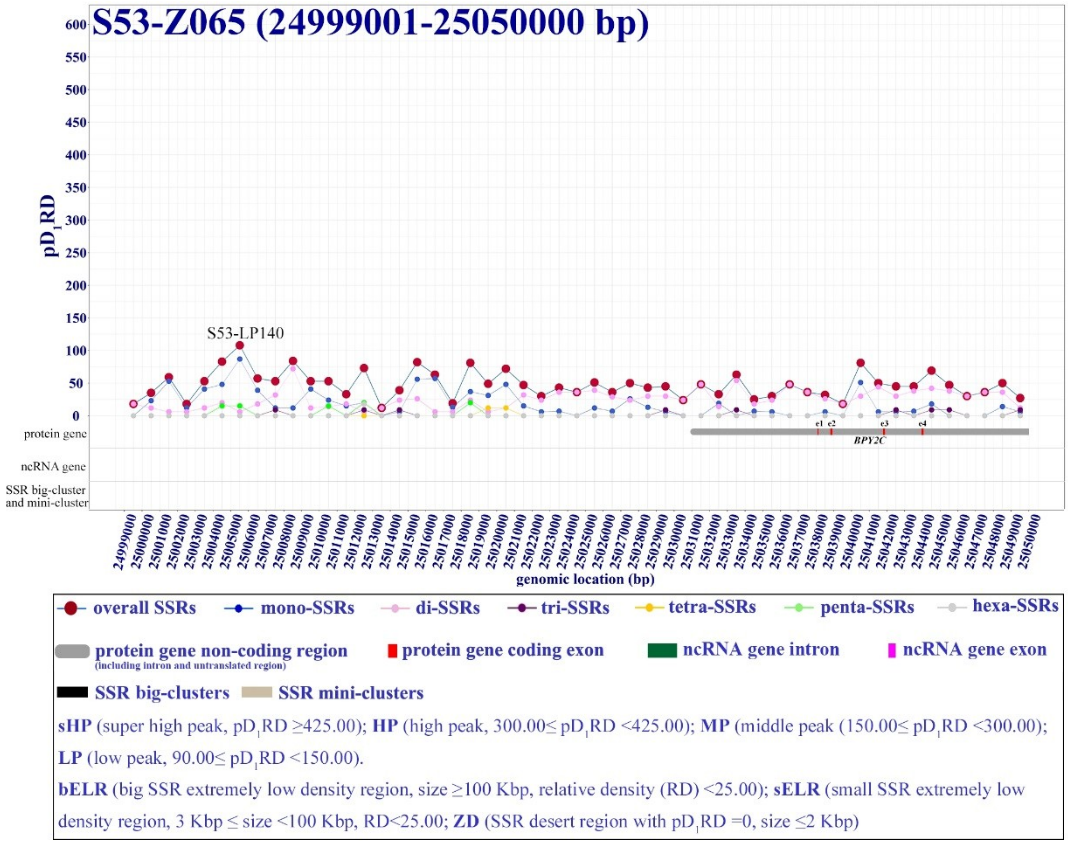

Supplementary Figure 1.495. The SSR position related D_1-relative density (pD_1RD) map of position at 24999001-25050000 bp of human reference Y-DNA (NC_000024.10) at resolution of 1 Kbp.

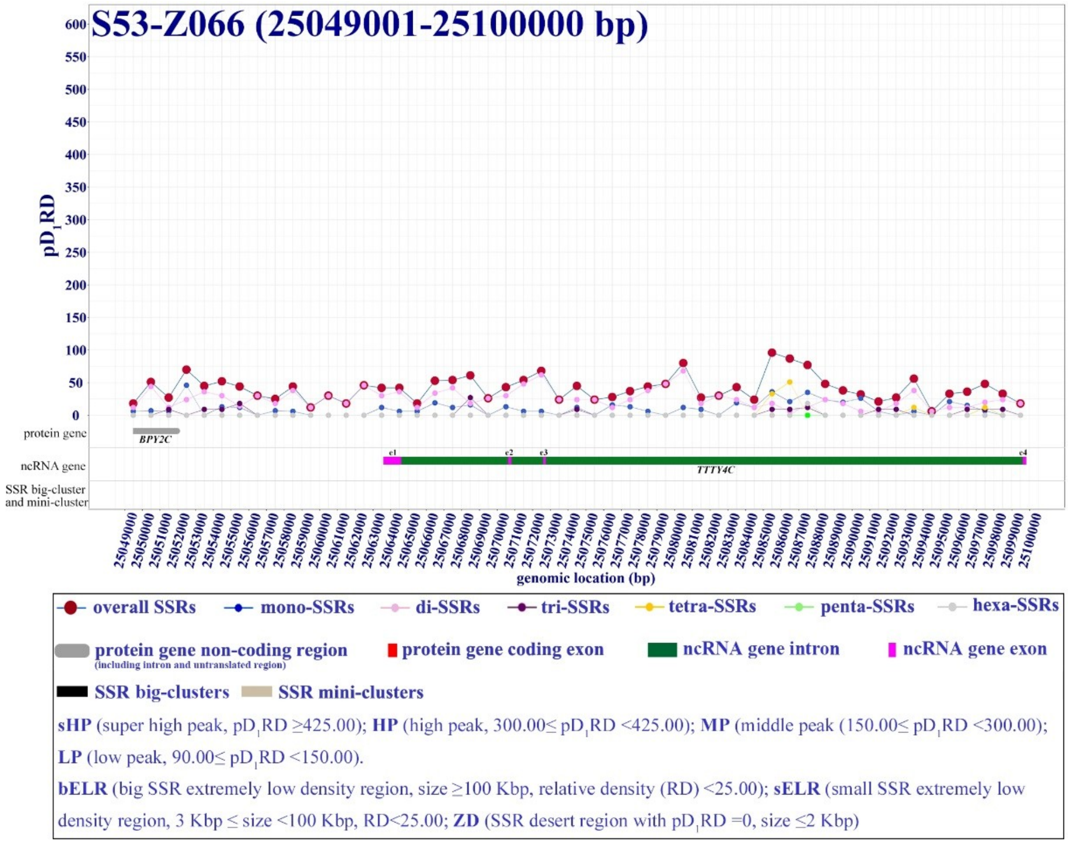

Supplementary Figure 1.496. The SSR position related D_1-relative density (pD_1RD) map of position at 25049001-25100000 bp of human reference Y-DNA (NC_000024.10) at resolution of 1 Kbp.

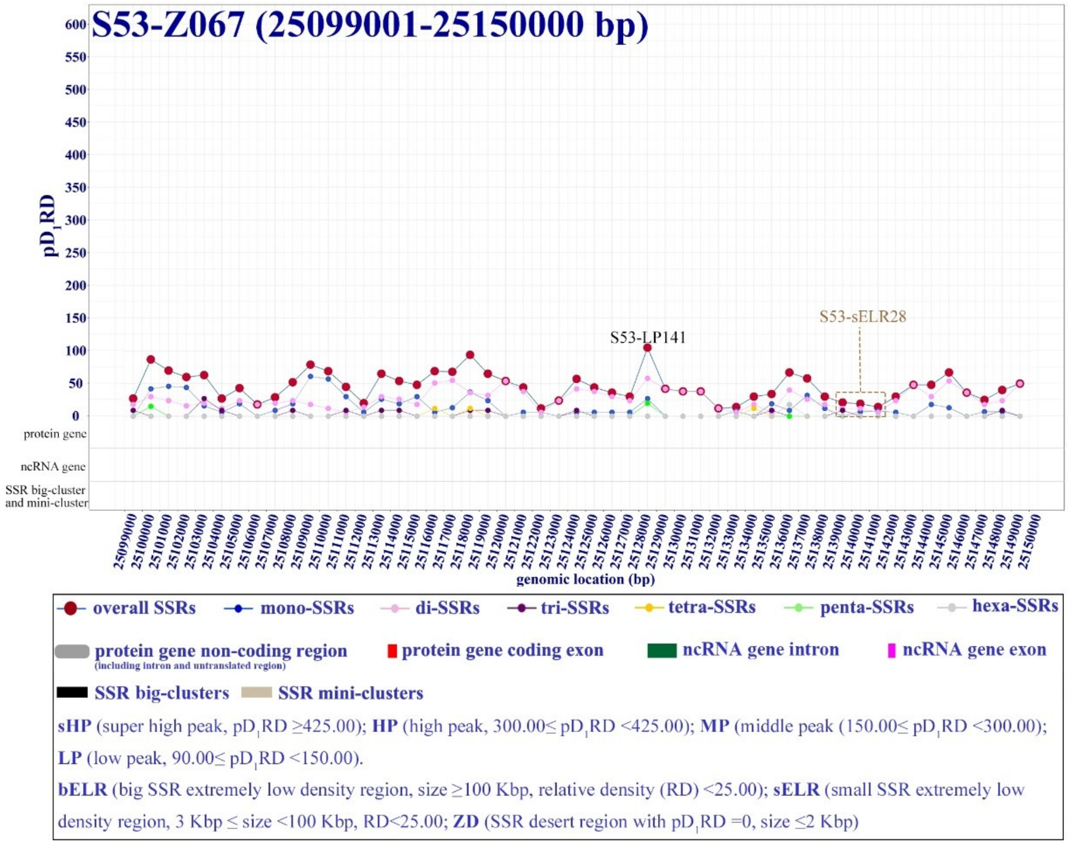

Supplementary Figure 1.497. The SSR position related D_1-relative density (pD_1RD) map of position at 25099001-25150000 bp of human reference Y-DNA (NC_000024.10) at resolution of 1 Kbp.

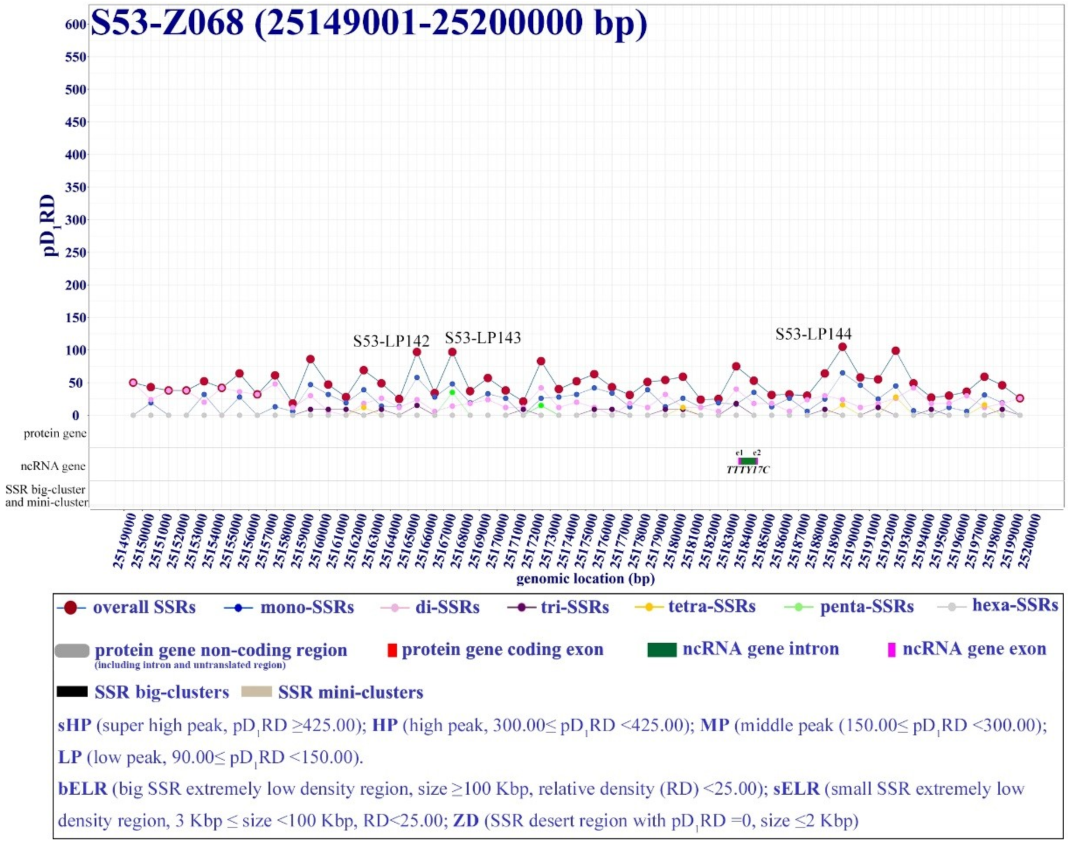

Supplementary Figure 1.498. The SSR position related D_1-relative density (pD_1RD) map of position at 25149001-25200000 bp of human reference Y-DNA (NC_000024.10) at resolution of 1 Kbp.

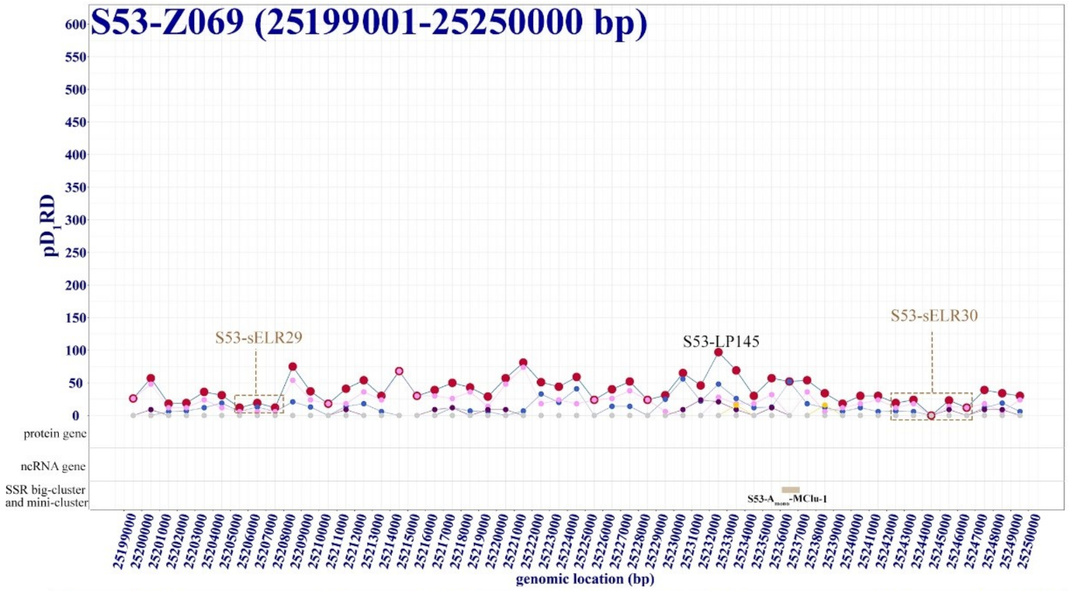

overall SSRs — mono-SSRs — di-SSRs — tri-SSRs — tetra-SSRs — penta-SSRs — hexa-SSRs

protein gene non-coding region (including intron and untranslated region) | protein gene coding exon | ncRNA gene intron | ncRNA gene exon

SSR big-clusters | SSR mini-clusters

sHP (super high peak, $pD_1RD \geq 425.00$); **HP** (high peak, $300.00 \leq pD_1RD < 425.00$); **MP** (middle peak ($150.00 \leq pD_1RD < 300.00$); **LP** (low peak, $90.00 \leq pD_1RD < 150.00$).

bELR (big SSR extremely low density region, size ≥100 Kbp, relative density (RD) <25.00); **sELR** (small SSR extremely low density region, 3 Kbp ≤ size <100 Kbp, RD<25.00; **ZD** (SSR desert region with $pD_1RD = 0$, size ≤2 Kbp)

Supplementary Figure 1.499. The SSR position related D_1-relative density (pD_1RD) map of position at 25199001-25250000 bp of human reference Y-DNA (NC_000024.10) at resolution of 1 Kbp.

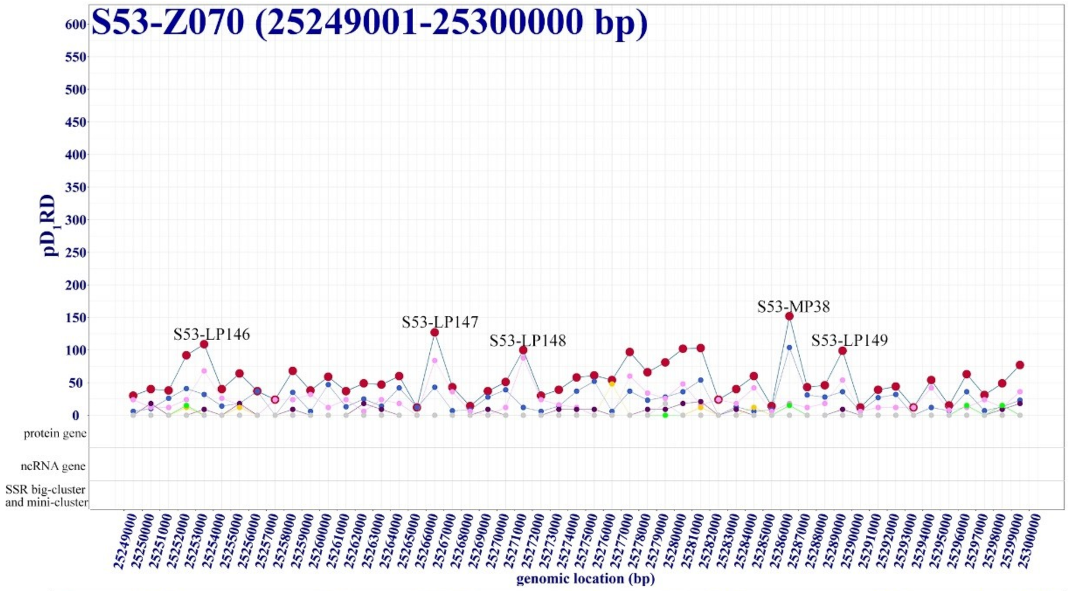

overall SSRs — mono-SSRs — di-SSRs — tri-SSRs — tetra-SSRs — penta-SSRs — hexa-SSRs

protein gene non-coding region (including intron and untranslated region) | protein gene coding exon | ncRNA gene intron | ncRNA gene exon

SSR big-clusters | SSR mini-clusters

sHP (super high peak, $pD_1RD \geq 425.00$); **HP** (high peak, $300.00 \leq pD_1RD < 425.00$); **MP** (middle peak ($150.00 \leq pD_1RD < 300.00$); **LP** (low peak, $90.00 \leq pD_1RD < 150.00$).

bELR (big SSR extremely low density region, size ≥100 Kbp, relative density (RD) <25.00); **sELR** (small SSR extremely low density region, 3 Kbp ≤ size <100 Kbp, RD<25.00; **ZD** (SSR desert region with $pD_1RD = 0$, size ≤2 Kbp)

Supplementary Figure 1.500. The SSR position related D_1-relative density (pD_1RD) map of position at 25249001-25300000 bp of human reference Y-DNA (NC_000024.10) at resolution of 1 Kbp.

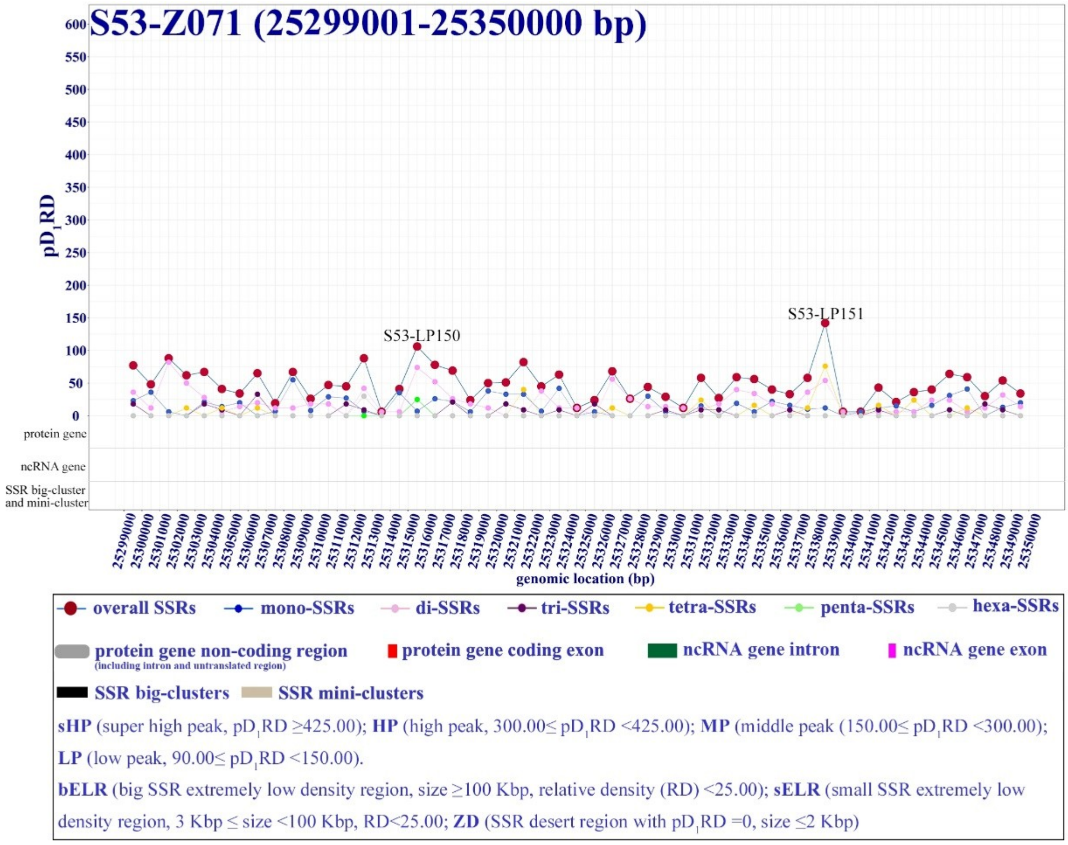

Supplementary Figure 1.501. The SSR position related D_1-relative density (pD_1RD) map of position at 25299001-25350000 bp of human reference Y-DNA (NC_000024.10) at resolution of 1 Kbp.

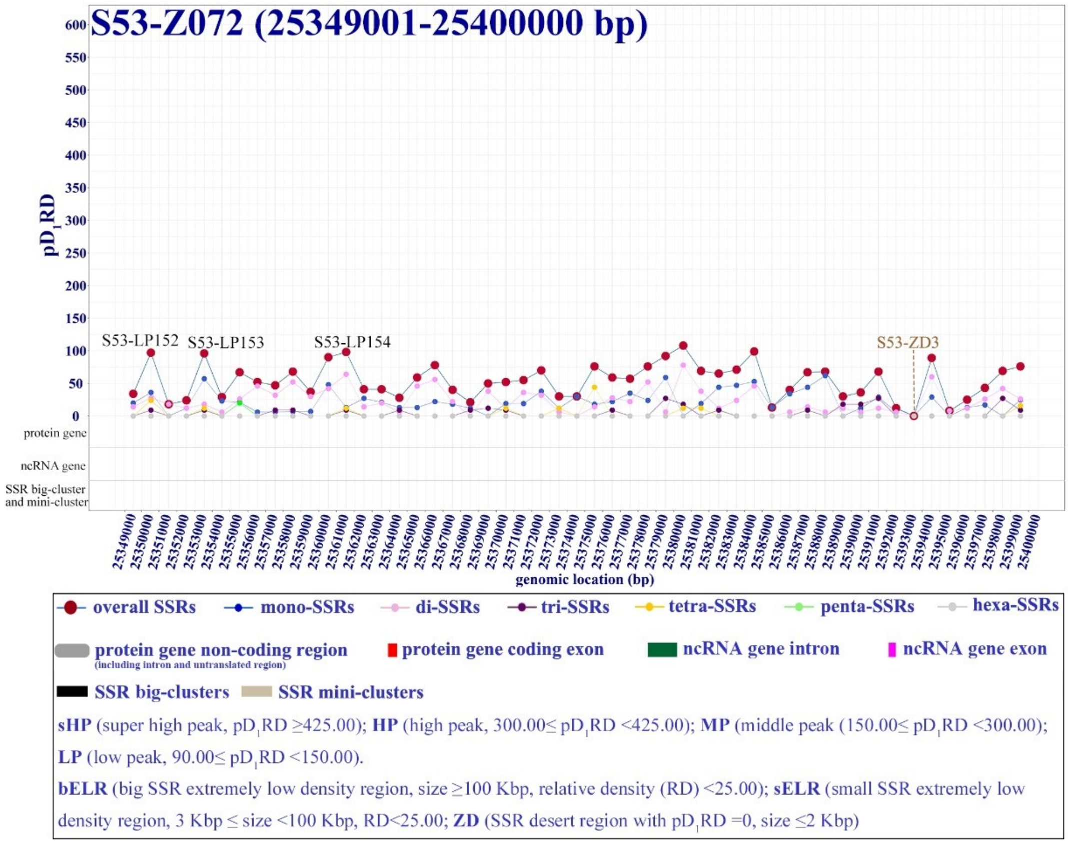

Supplementary Figure 1.502. The SSR position related D_1-relative density (pD_1RD) map of position at 25349001-25400000 bp of human reference Y-DNA (NC_000024.10) at resolution of 1 Kbp.

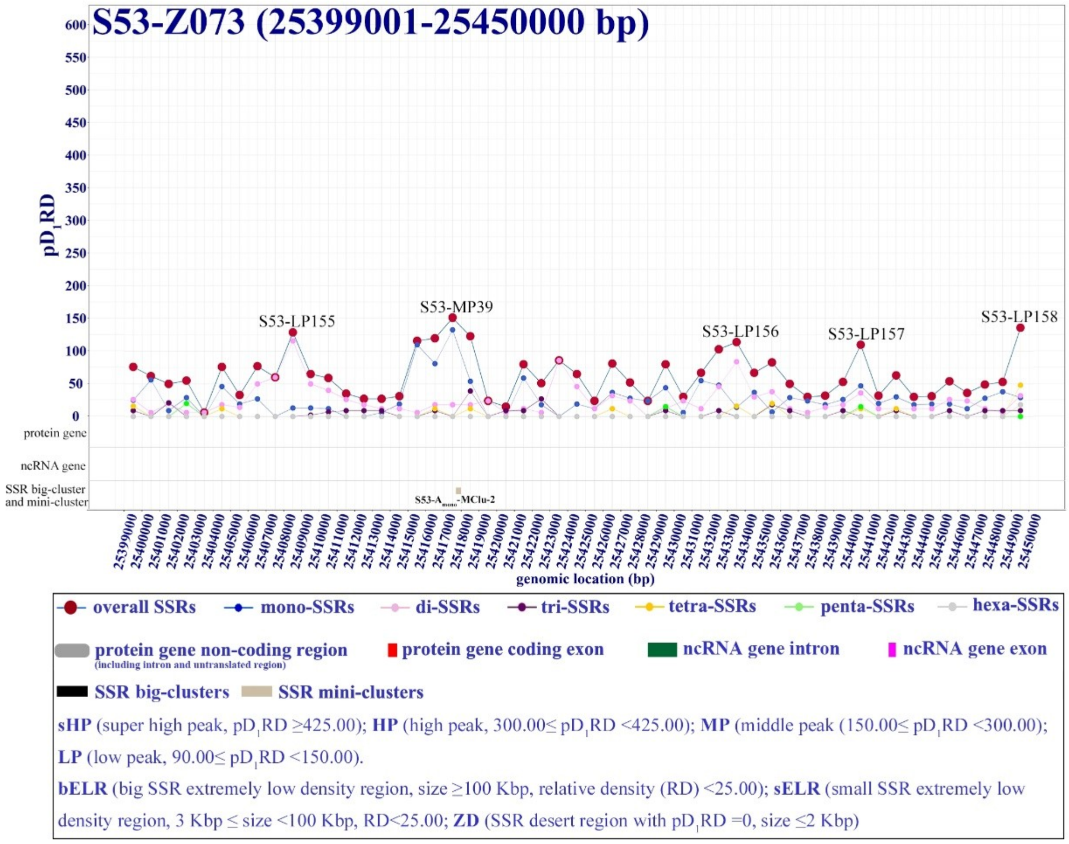

Supplementary Figure 1.503. The SSR position related D_1-relative density (pD_1RD) map of position at 25399001-25450000 bp of human reference Y-DNA (NC_000024.10) at resolution of 1 Kbp.

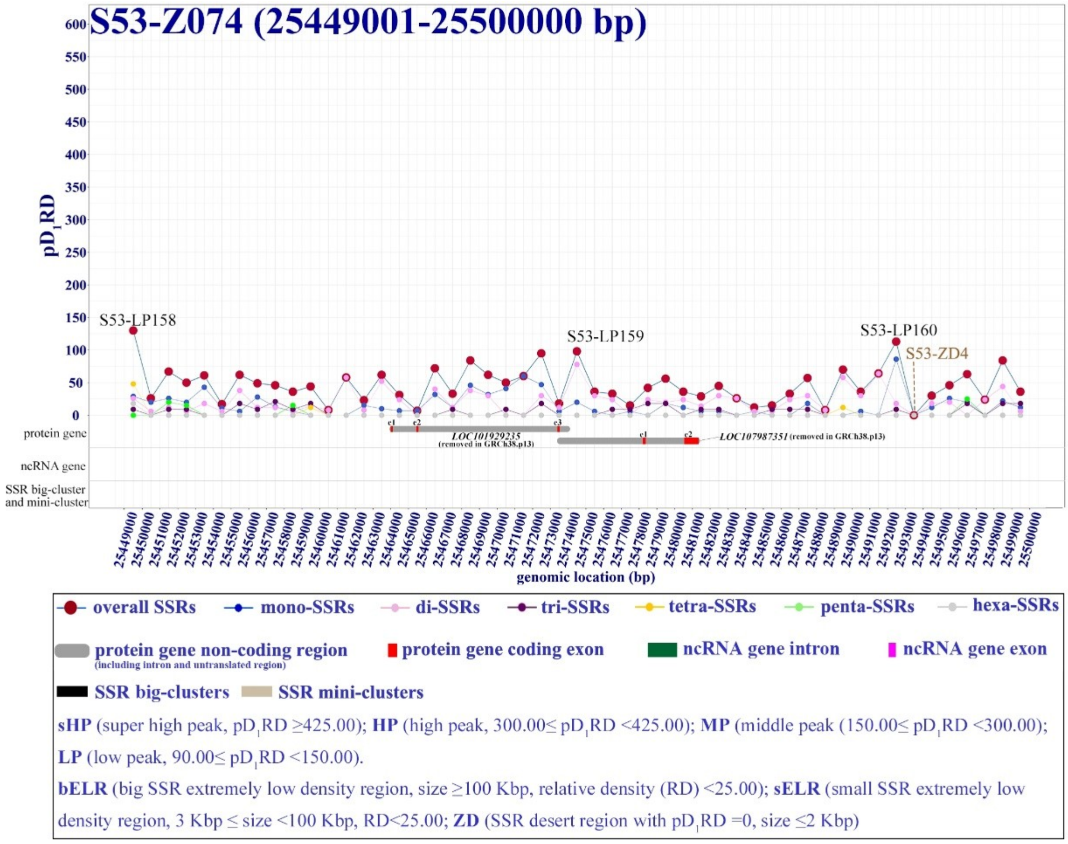

Supplementary Figure 1.504. The SSR position related D_1-relative density (pD_1RD) map of position at 25449001-25500000 bp of human reference Y-DNA (NC_000024.10) at resolution of 1 Kbp.

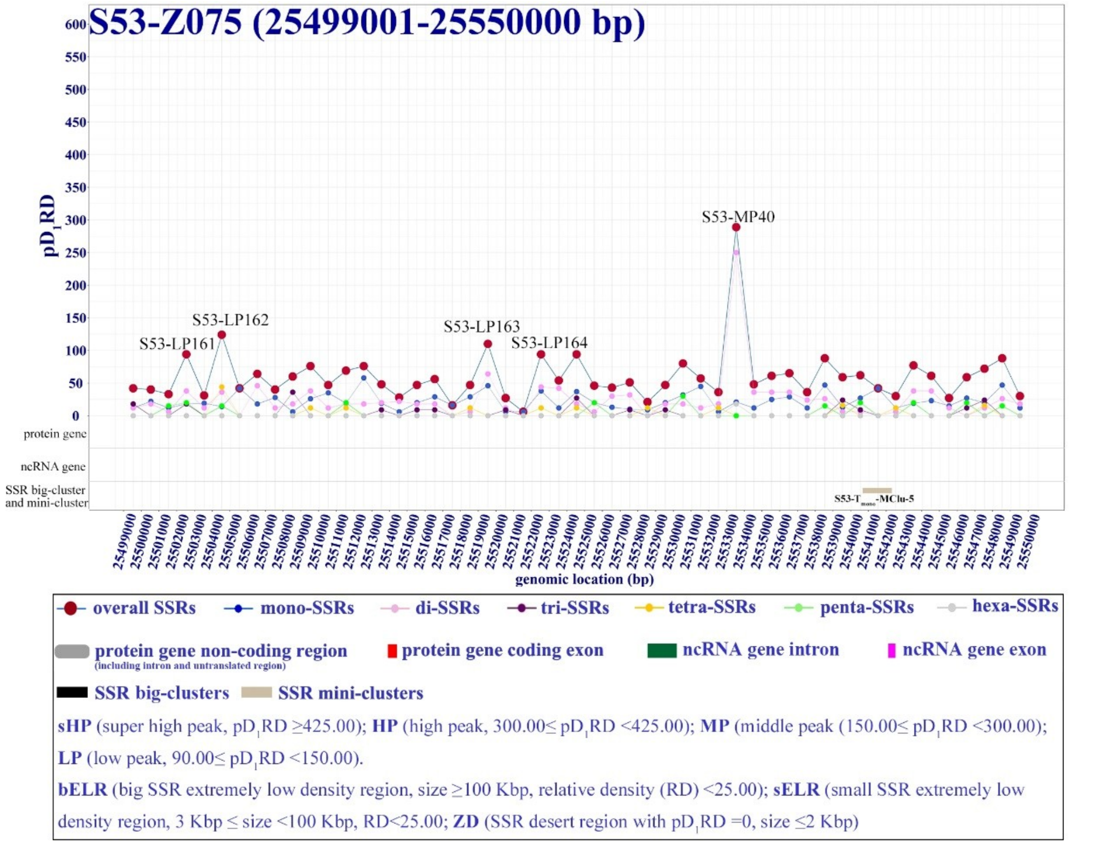

Supplementary Figure 1.505. The SSR position related D_1-relative density (pD_1RD) map of position at 25499001-25550000 bp of human reference Y-DNA (NC_000024.10) at resolution of 1 Kbp.

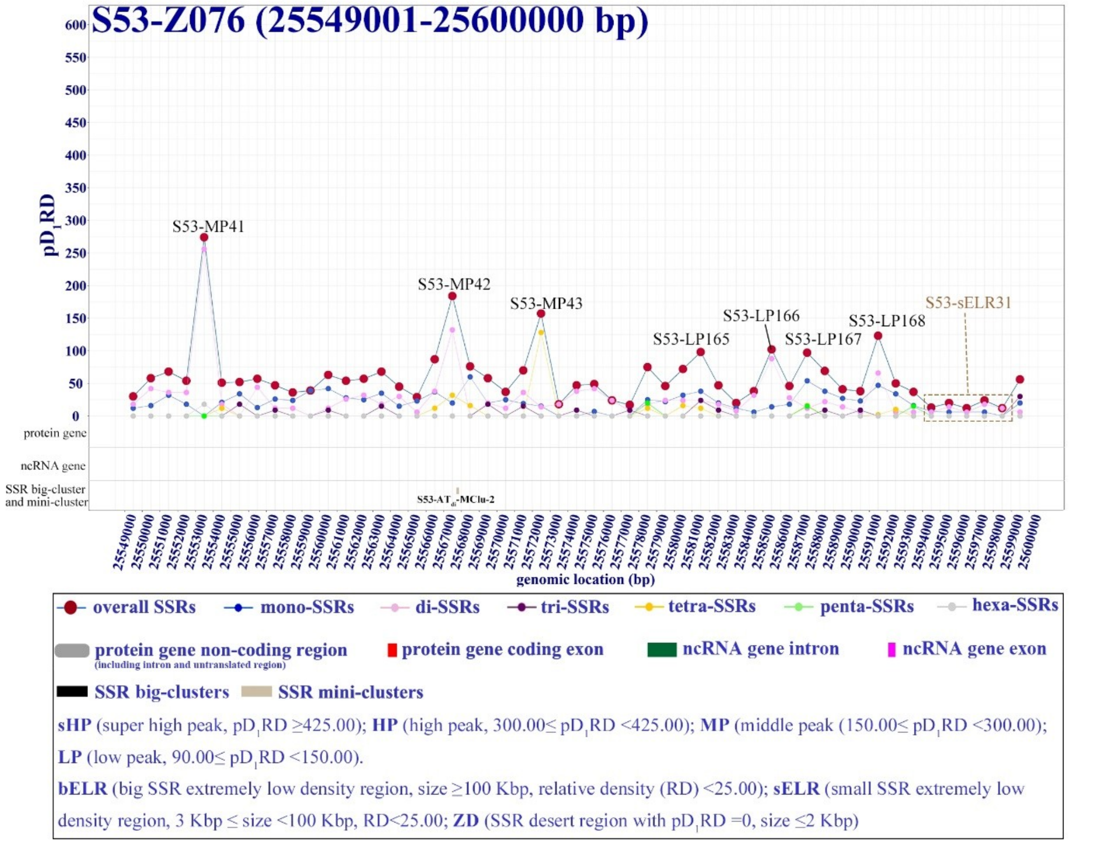

Supplementary Figure 1.506. The SSR position related D_1-relative density (pD_1RD) map of position at 25549001-25600000 bp of human reference Y-DNA (NC_000024.10) at resolution of 1 Kbp.

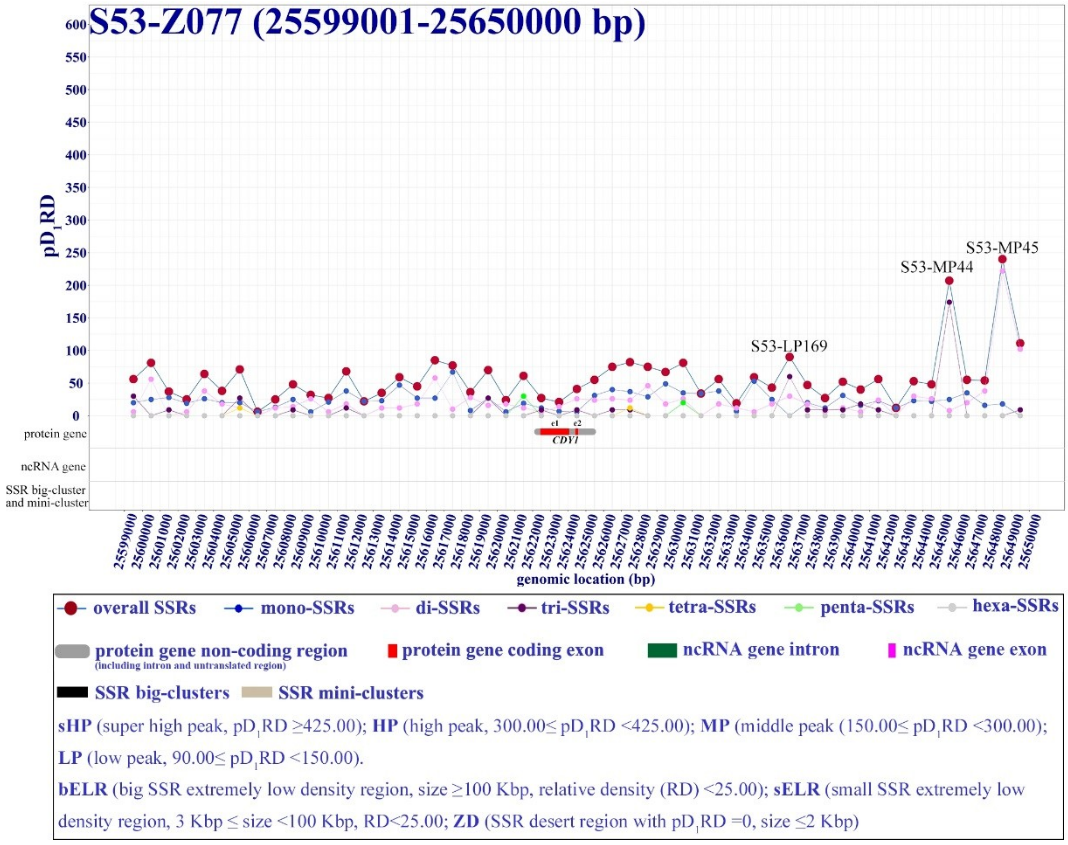

Supplementary Figure 1.507. The SSR position related D_1-relative density (pD_1RD) map of position at 25599001-25650000 bp of human reference Y-DNA (NC_000024.10) at resolution of 1 Kbp.

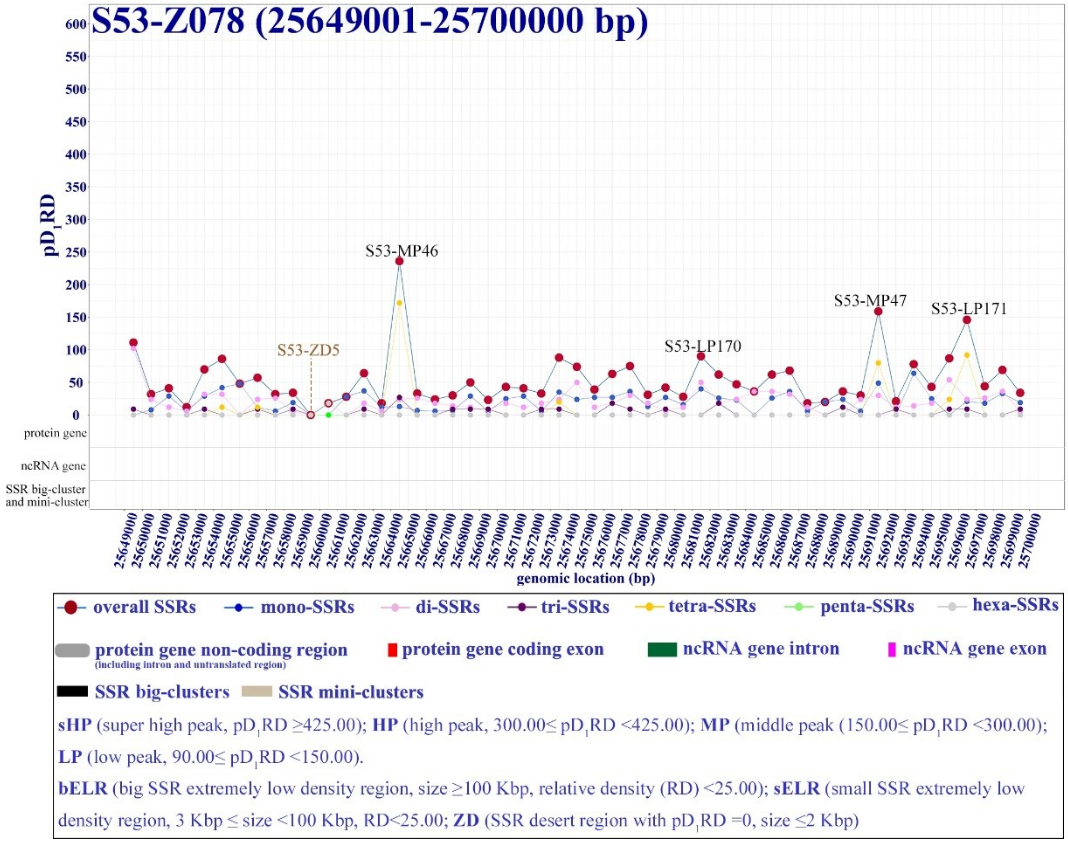

Supplementary Figure 1.508. The SSR position related D_1-relative density (pD_1RD) map of position at 25649001-25700000 bp of human reference Y-DNA (NC_000024.10) at resolution of 1 Kbp.

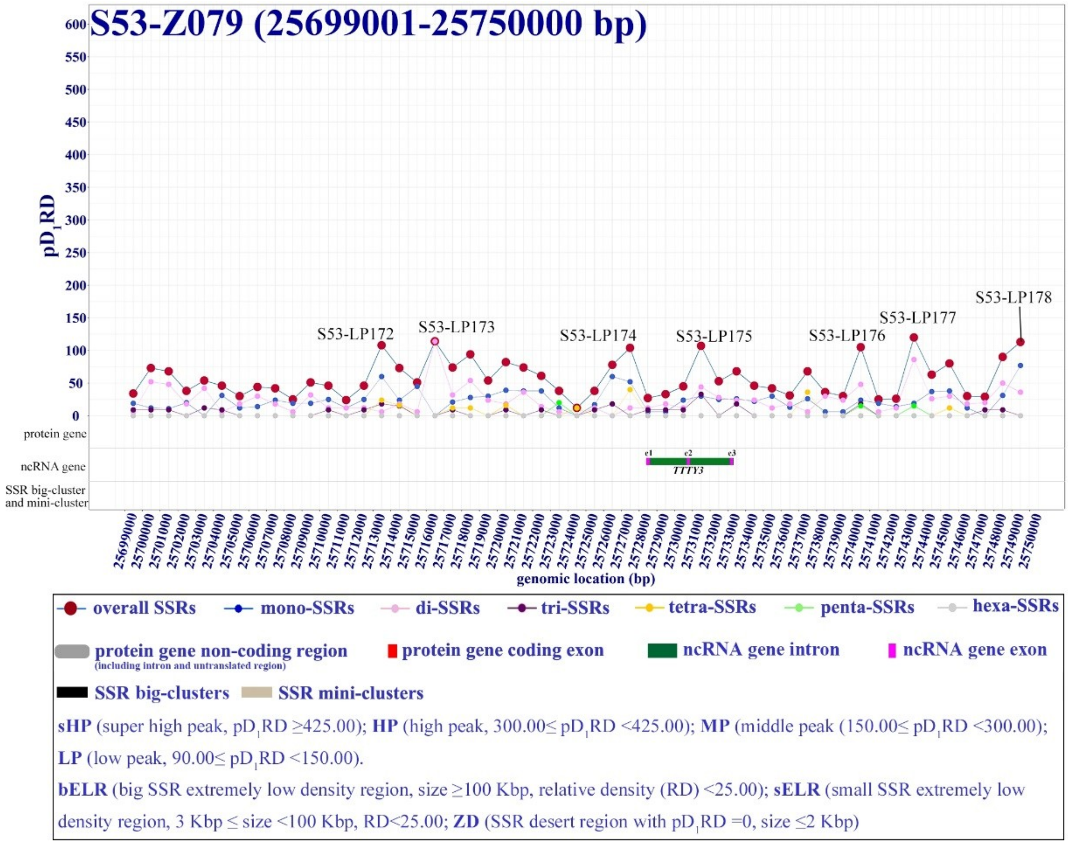

Supplementary Figure 1.509. The SSR position related D_1-relative density (pD_1RD) map of position at 25699001-25750000 bp of human reference Y-DNA (NC_000024.10) at resolution of 1 Kbp.

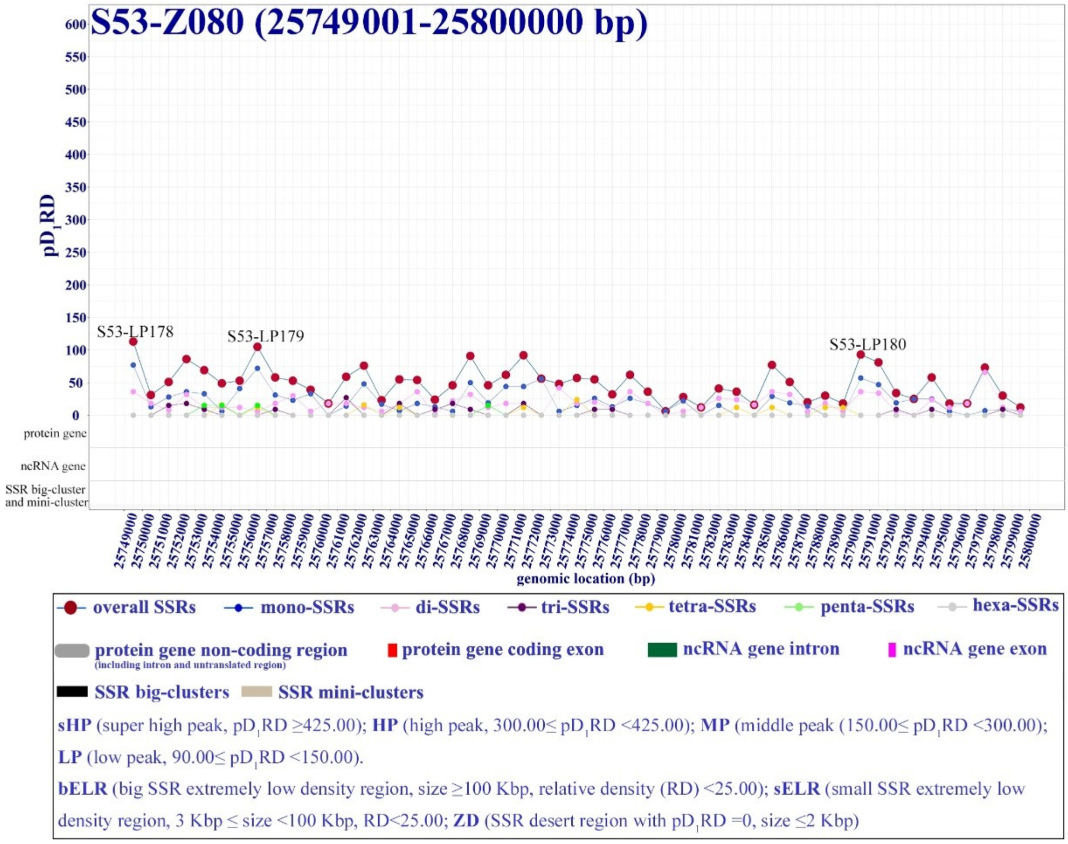

Supplementary Figure 1.510. The SSR position related D_1-relative density (pD_1RD) map of position at 25749001-25800000 bp of human reference Y-DNA (NC_000024.10) at resolution of 1 Kbp.

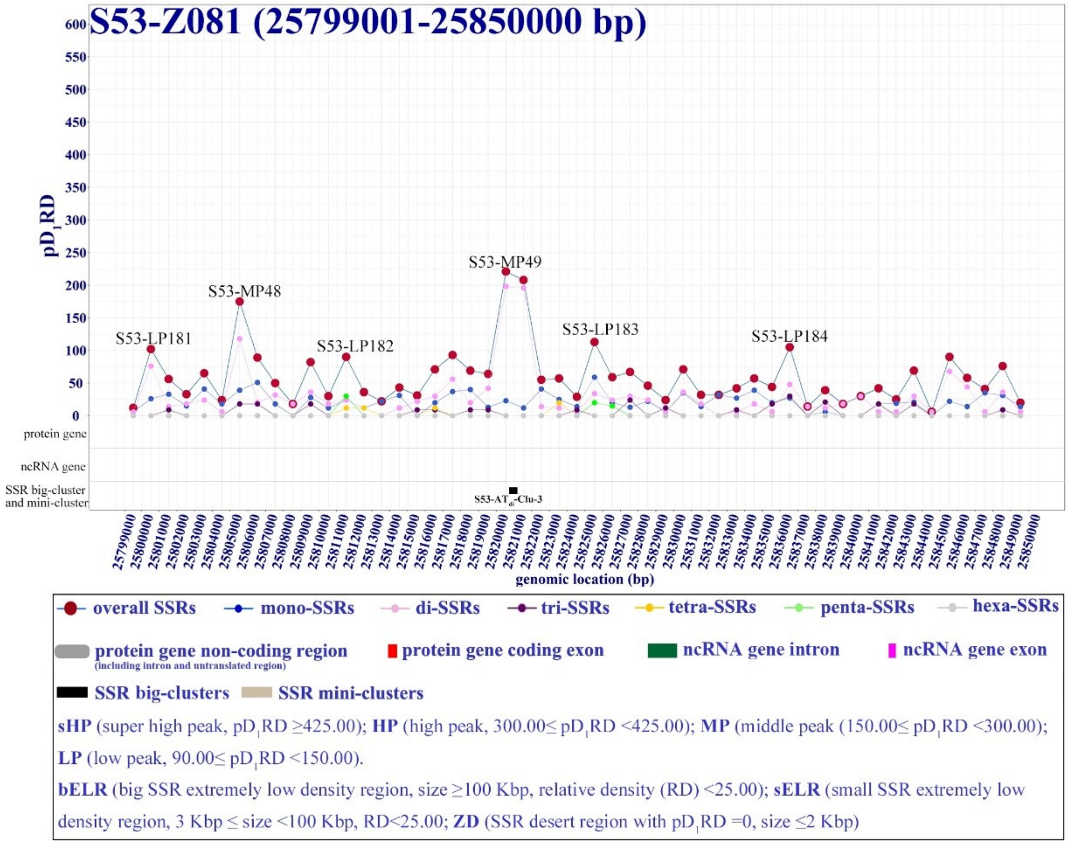

Supplementary Figure 1.511. The SSR position related D_1-relative density (pD_1RD) map of position at 25799001-25850000 bp of human reference Y-DNA (NC_000024.10) at resolution of 1 Kbp.

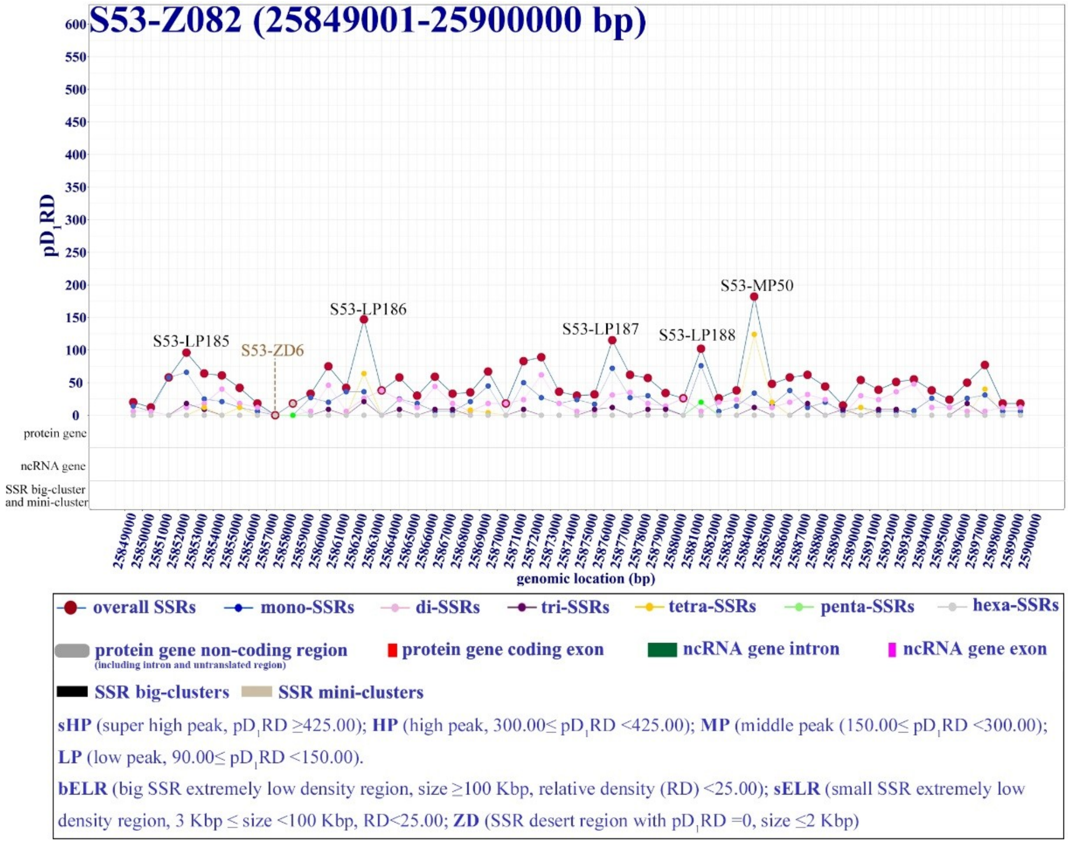

Supplementary Figure 1.512. The SSR position related D_1-relative density (pD_1RD) map of position at 25849001-25900000 bp of human reference Y-DNA (NC_000024.10) at resolution of 1 Kbp.

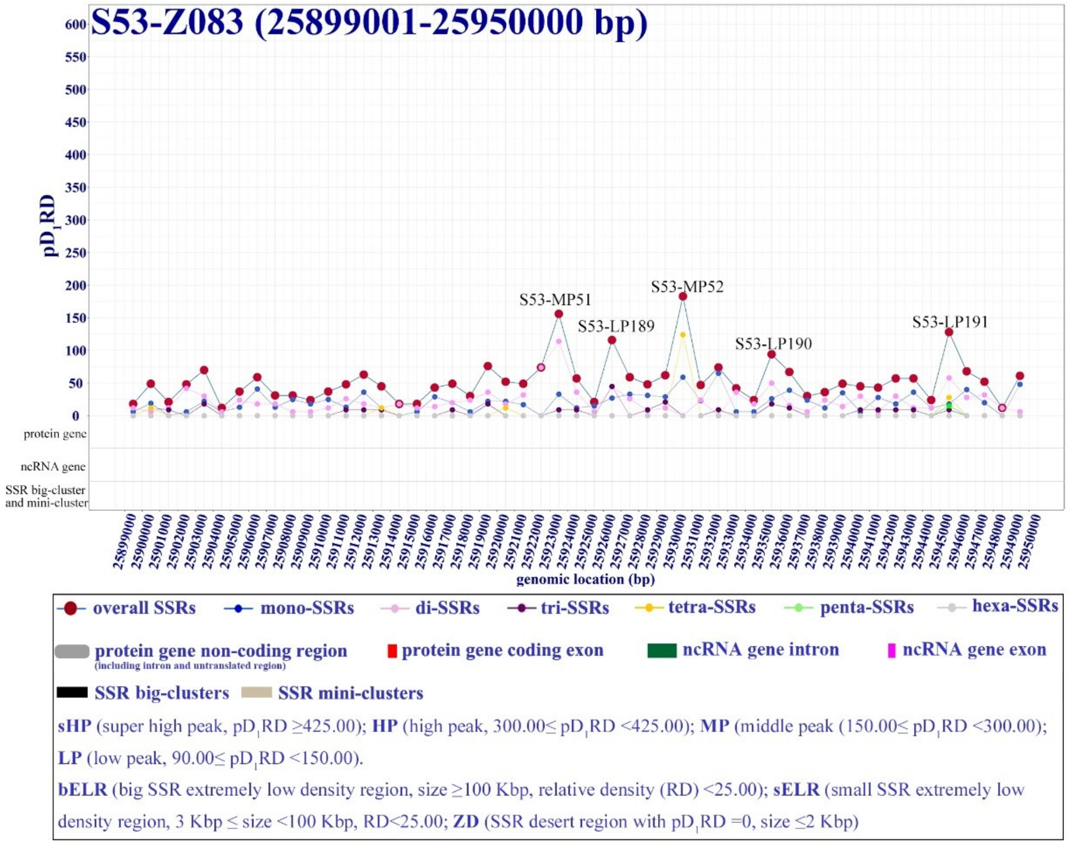

Supplementary Figure 1.513. The SSR position related D_1-relative density (pD_1RD) map of position at 25899001-25950000 bp of human reference Y-DNA (NC_000024.10) at resolution of 1 Kbp.

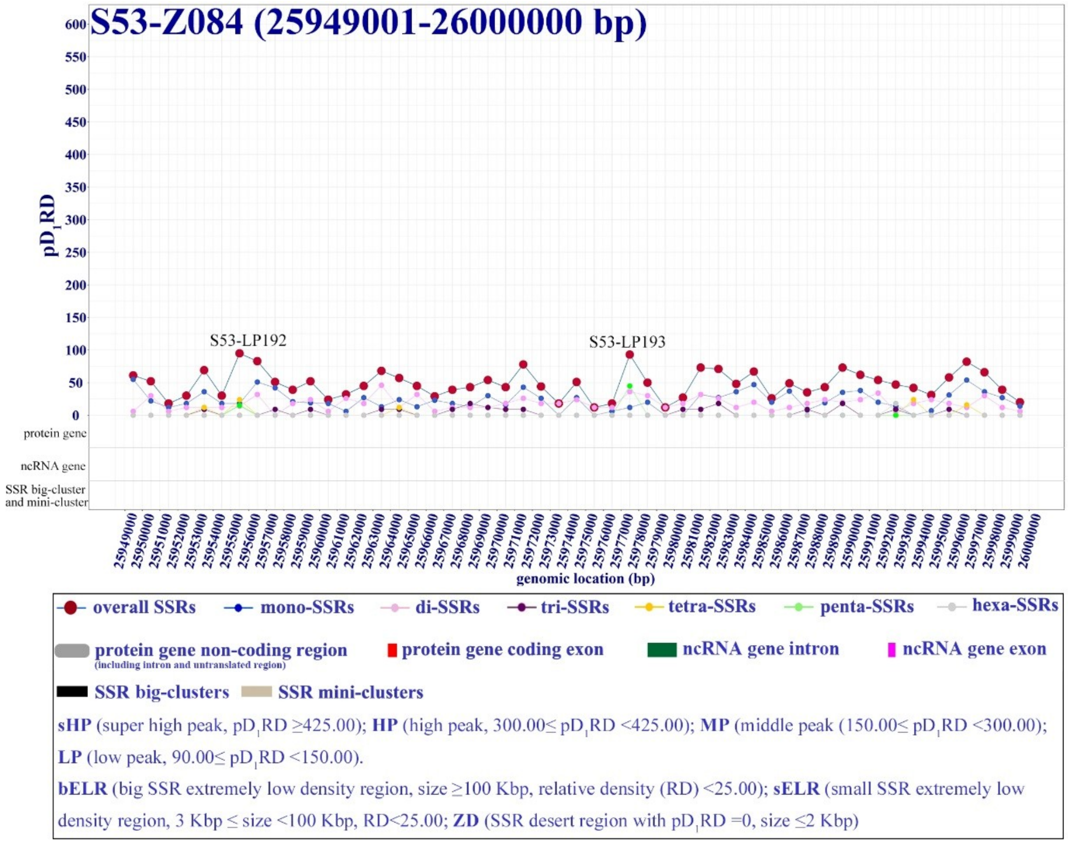

Supplementary Figure 1.514. The SSR position related D_1-relative density (pD_1RD) map of position at 25949001-26000000 bp of human reference Y-DNA (NC_000024.10) at resolution of 1 Kbp.

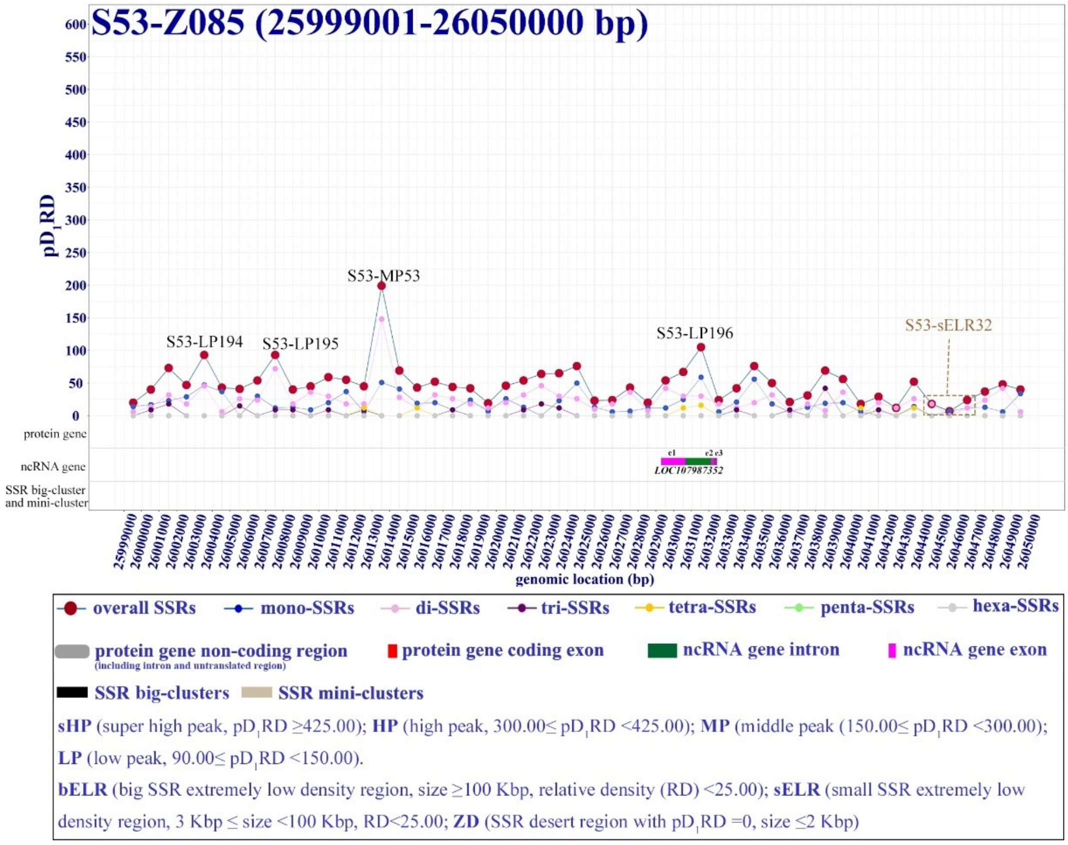

Supplementary Figure 1.515. The SSR position related D_1-relative density (pD_1RD) map of position at 25999001-26050000 bp of human reference Y-DNA (NC_000024.10) at resolution of 1 Kbp.

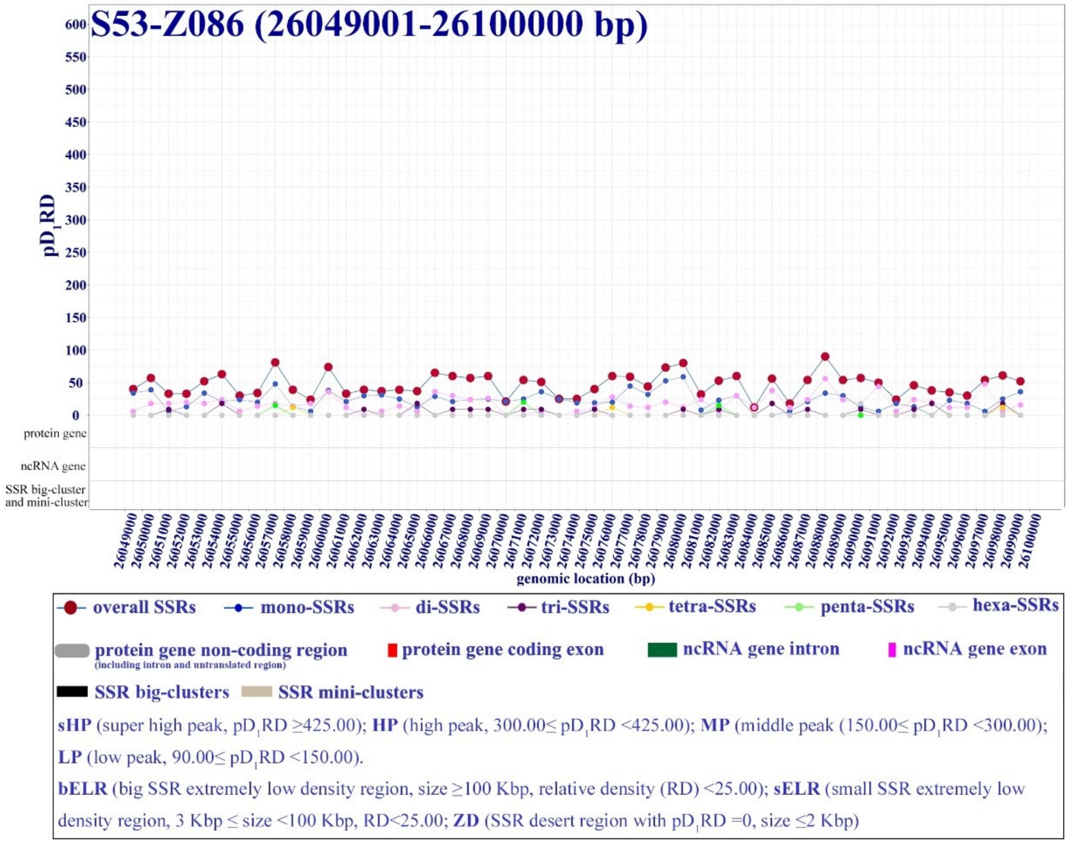

Supplementary Figure 1.516. The SSR position related D_1-relative density (pD_1RD) map of position at 26049001-26100000 bp of human reference Y-DNA (NC_000024.10) at resolution of 1 Kbp.

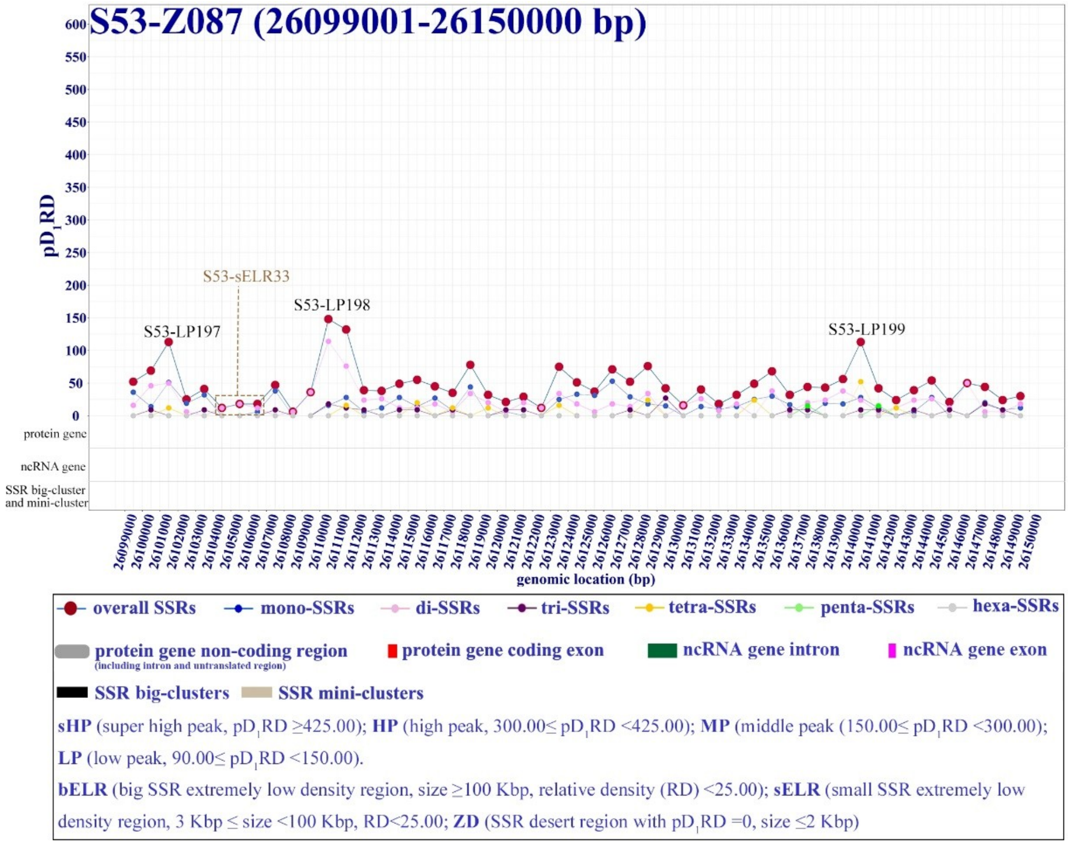

Supplementary Figure 1.517. The SSR position related D_1-relative density (pD_1RD) map of position at 26099001-26150000 bp of human reference Y-DNA (NC_000024.10) at resolution of 1 Kbp.

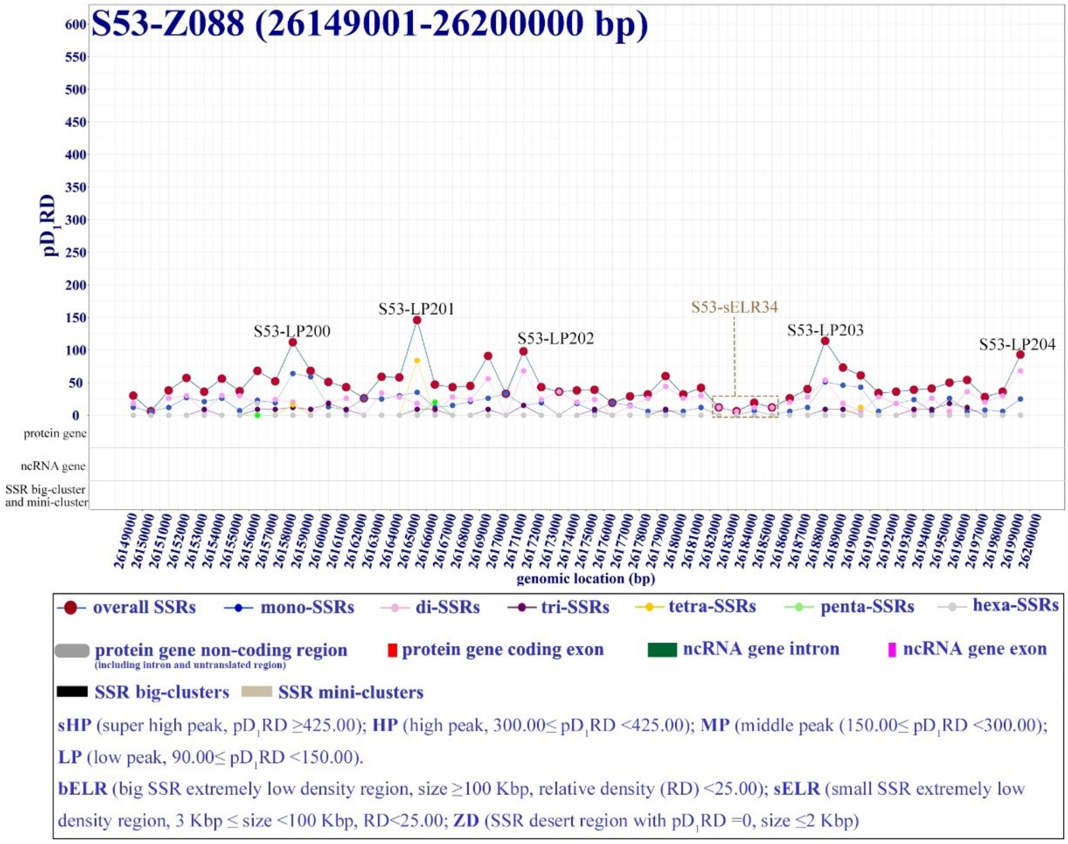

Supplementary Figure 1.518. The SSR position related D_1-relative density (pD_1RD) map of position at 26149001-26200000 bp of human reference Y-DNA (NC_000024.10) at resolution of 1 Kbp.

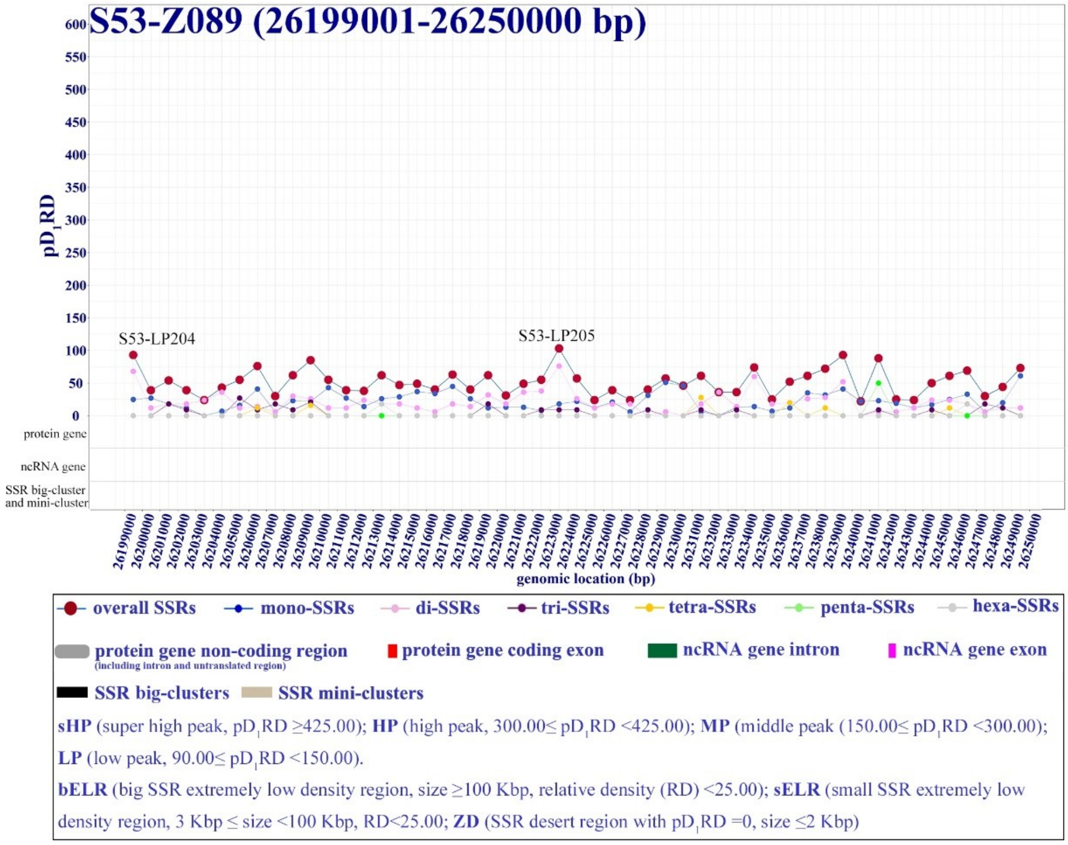

Supplementary Figure 1.519. The SSR position related D_1-relative density (pD_1RD) map of position at 26199001-26250000 bp of human reference Y-DNA (NC_000024.10) at resolution of 1 Kbp.

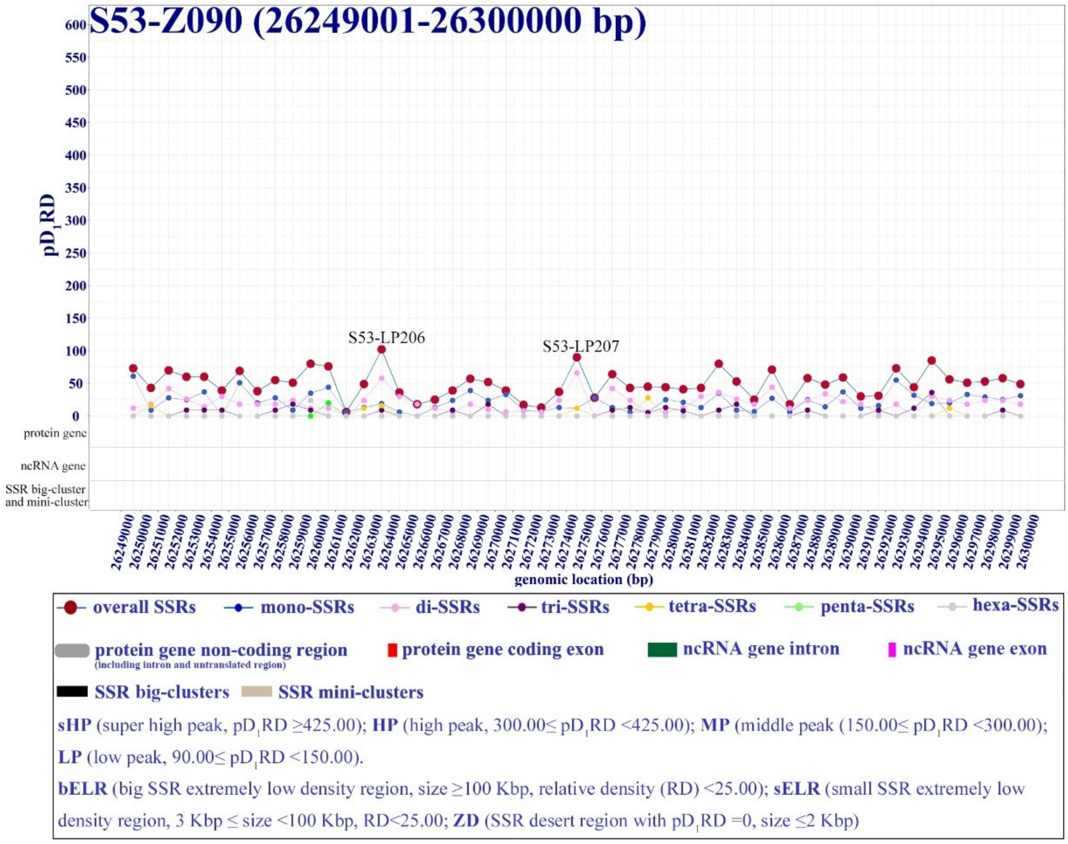

Supplementary Figure 1.520. The SSR position related D_1-relative density (pD_1RD) map of position at 26249001-26300000 bp of human reference Y-DNA (NC_000024.10) at resolution of 1 Kbp.

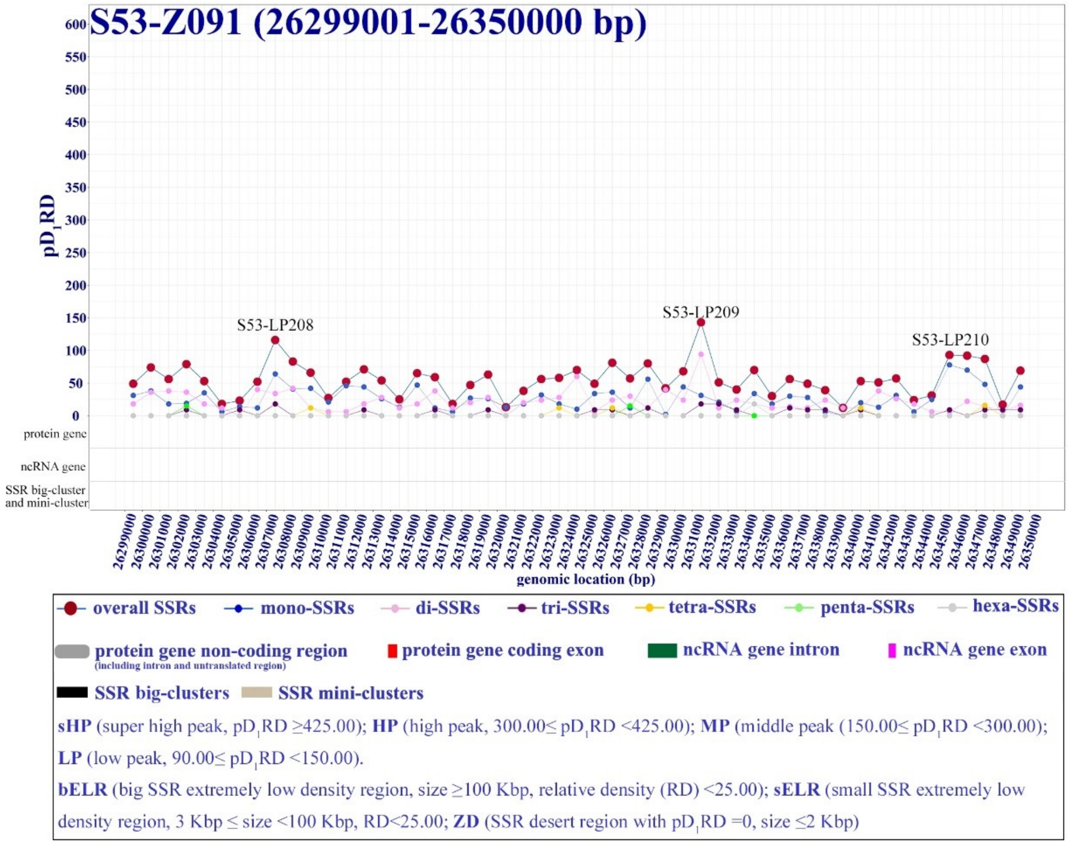

Supplementary Figure 1.521. The SSR position related D_1-relative density (pD_1RD) map of position at 26299001-26350000 bp of human reference Y-DNA (NC_000024.10) at resolution of 1 Kbp.

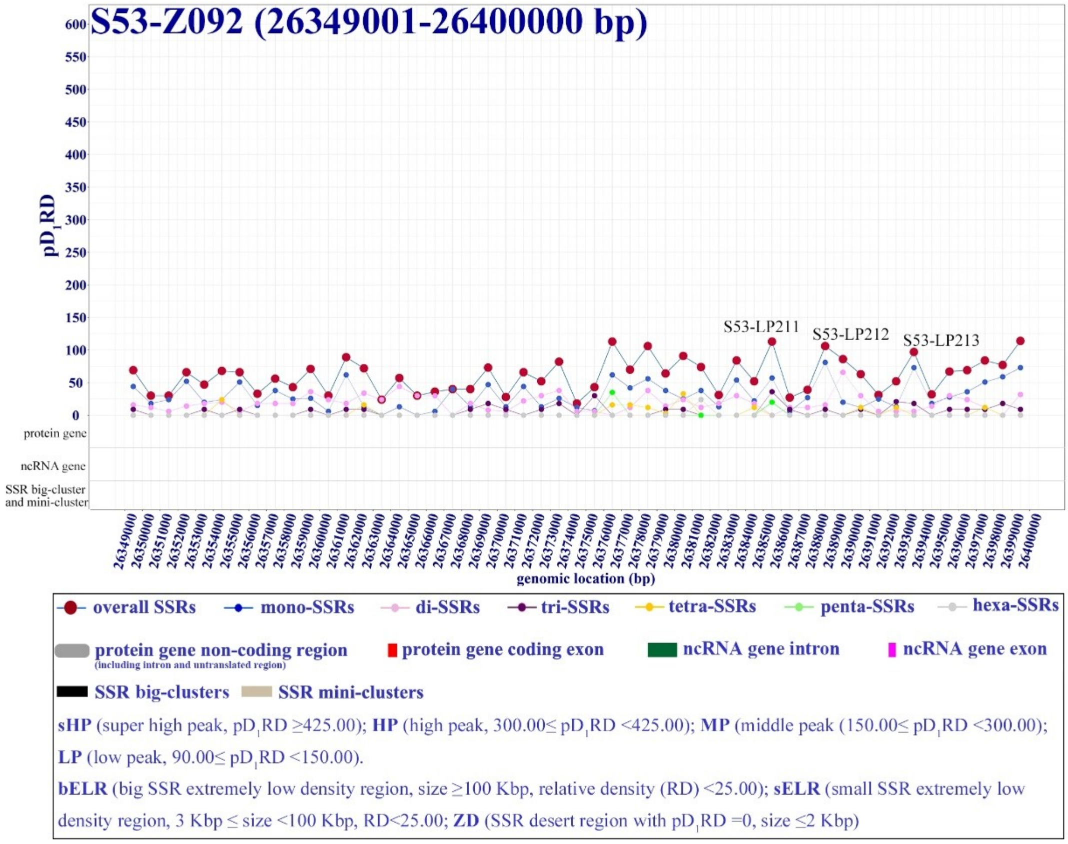

Supplementary Figure 1.522. The SSR position related D_1-relative density (pD_1RD) map of position at 26349001-26400000 bp of human reference Y-DNA (NC_000024.10) at resolution of 1 Kbp.

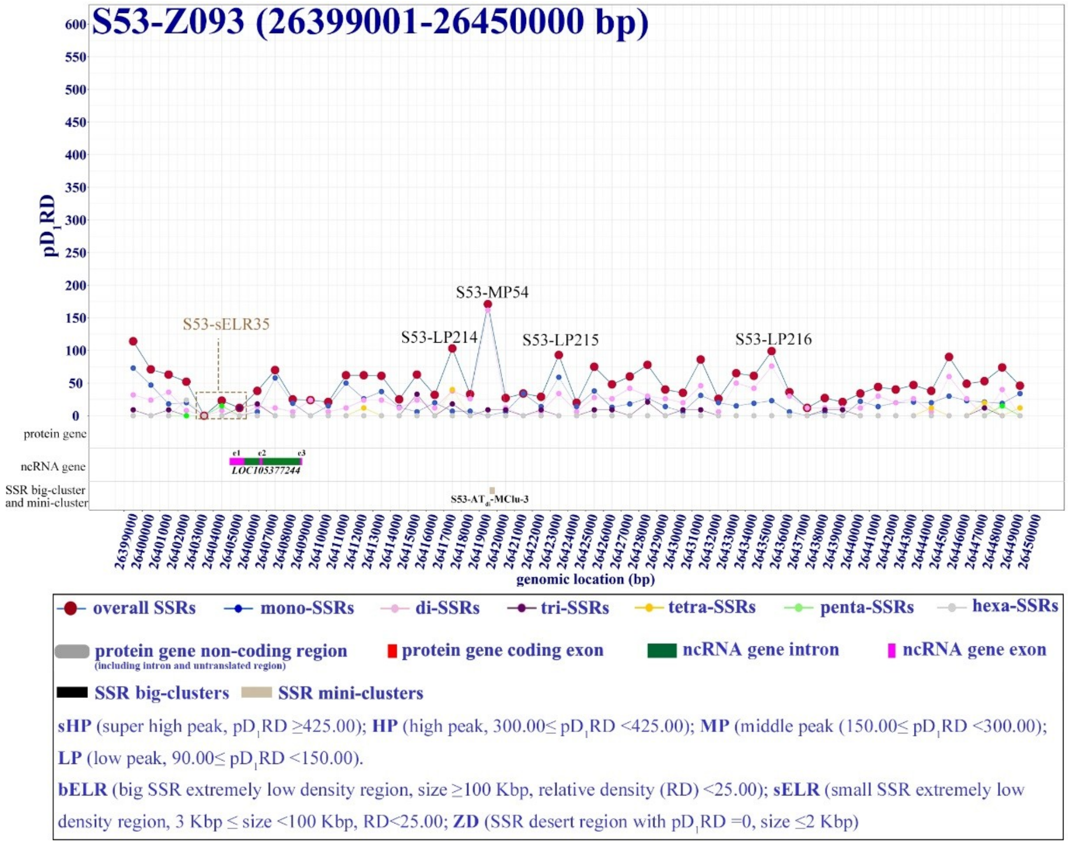

Supplementary Figure 1.523. The SSR position related D_1-relative density (pD_1RD) map of position at 26399001-26450000 bp of human reference Y-DNA (NC_000024.10) at resolution of 1 Kbp.

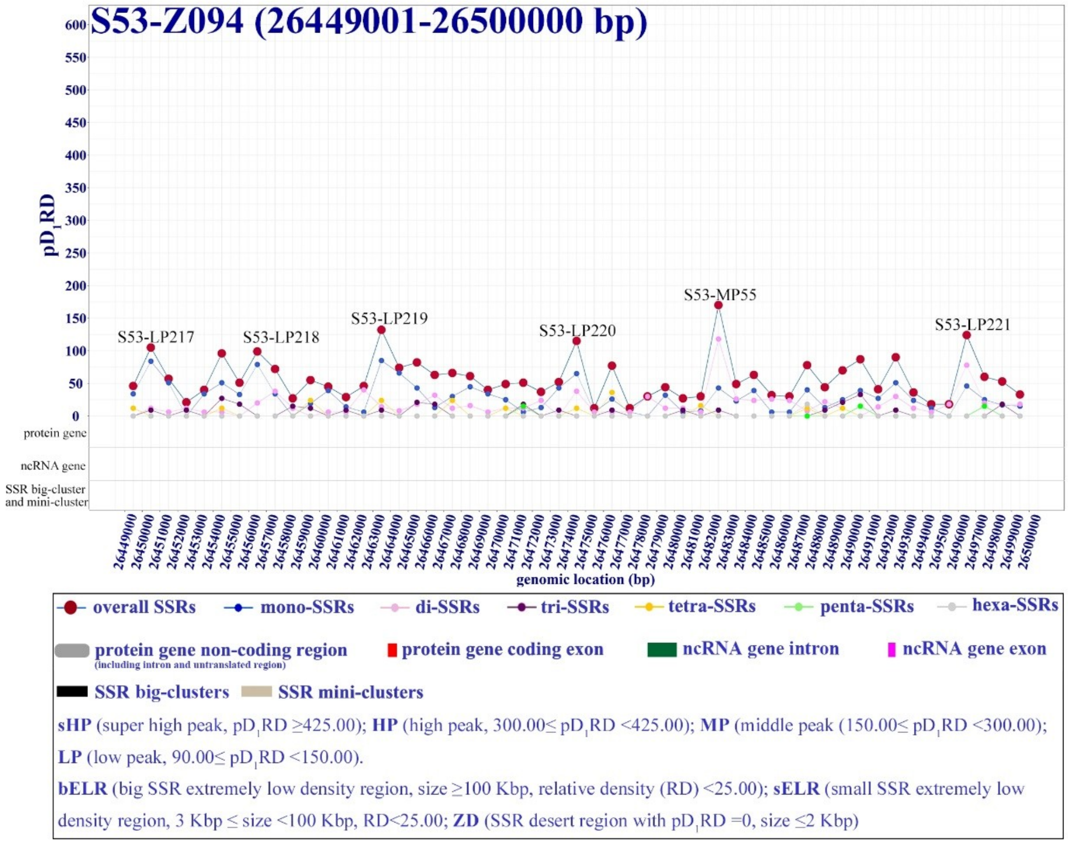

Supplementary Figure 1.524. The SSR position related D_1-relative density (pD_1RD) map of position at 26449001-26500000 bp of human reference Y-DNA (NC_000024.10) at resolution of 1 Kbp.

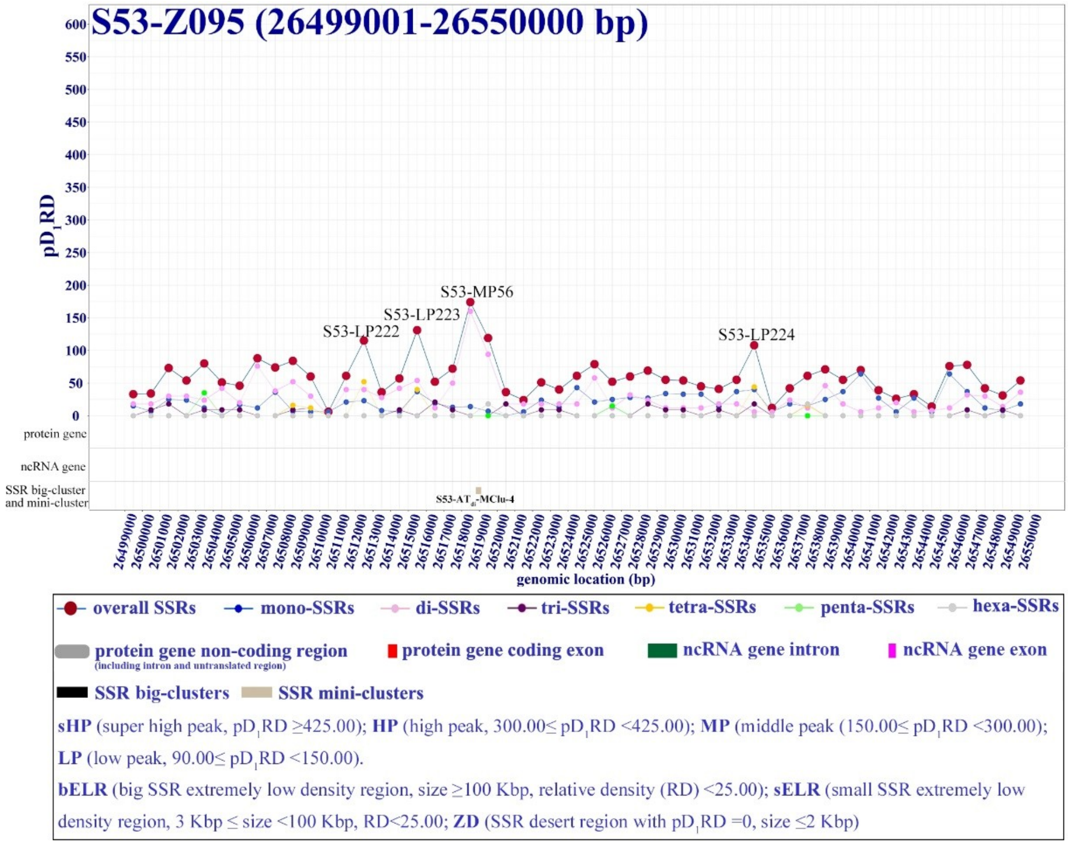

Supplementary Figure 1.525. The SSR position related D_1-relative density (pD_1RD) map of position at 26499001-26550000 bp of human reference Y-DNA (NC_000024.10) at resolution of 1 Kbp.

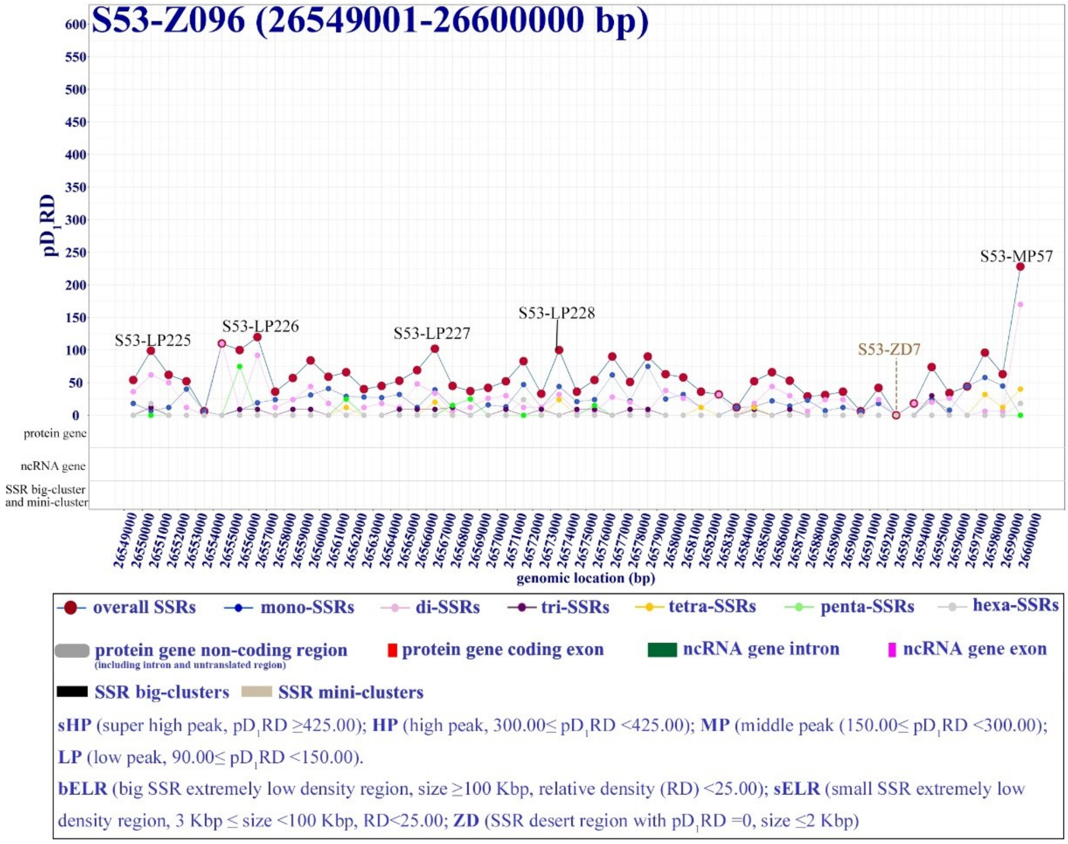

Supplementary Figure 1.526. The SSR position related D_1-relative density (pD_1RD) map of position at 26549001-26600000 bp of human reference Y-DNA (NC_000024.10) at resolution of 1 Kbp.

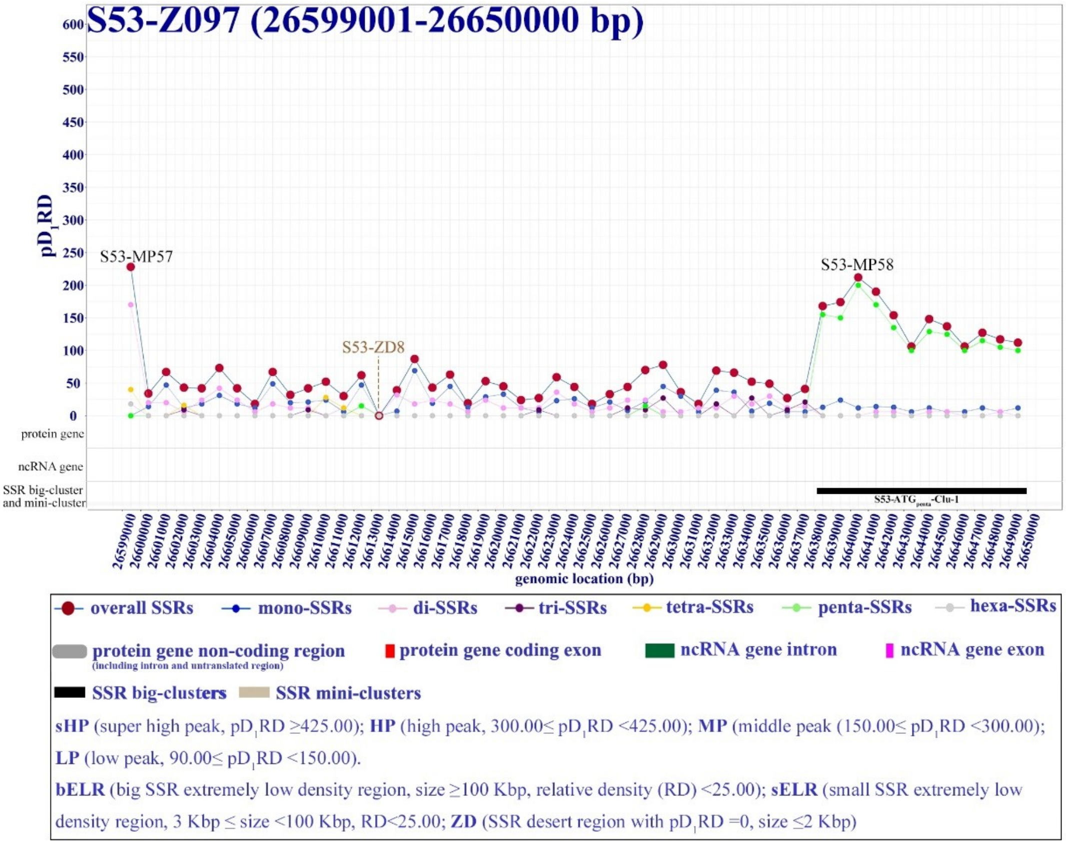

Supplementary Figure 1.527. The SSR position related D_1-relative density (pD_1RD) map of position at 26599001-26650000 bp of human reference Y-DNA (NC_000024.10) at resolution of 1 Kbp.

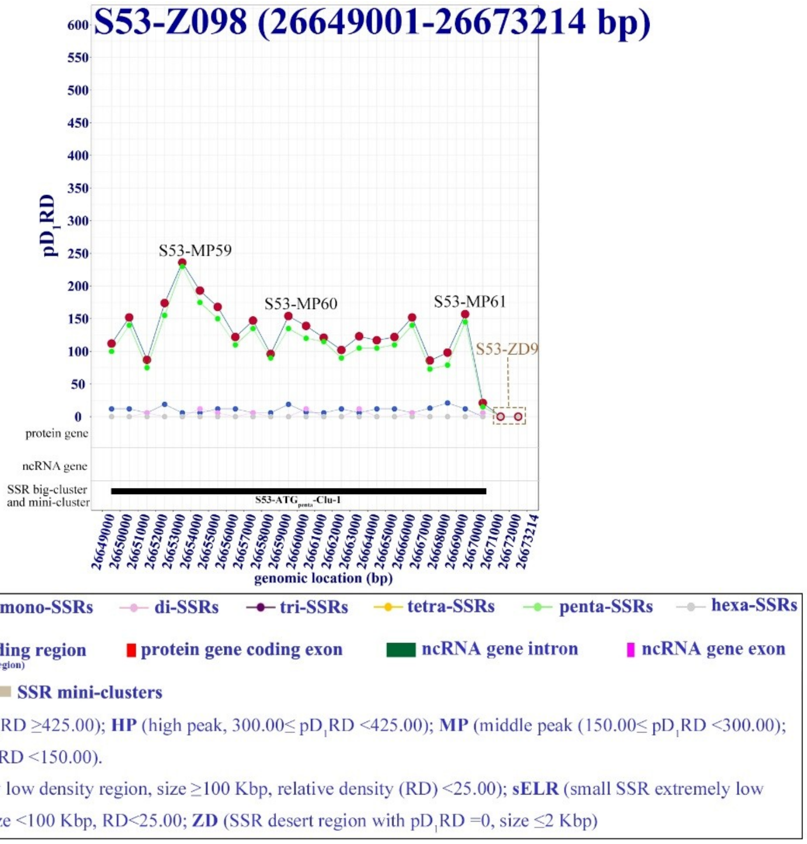

Supplementary Figure 1.528. The SSR position related D_1-relative density (pD_1RD) map of position at 26649001-26673214 bp (unnormal zone <51000 bp) of human reference Y-DNA (NC_000024.10) at resolution of 1 Kbp.

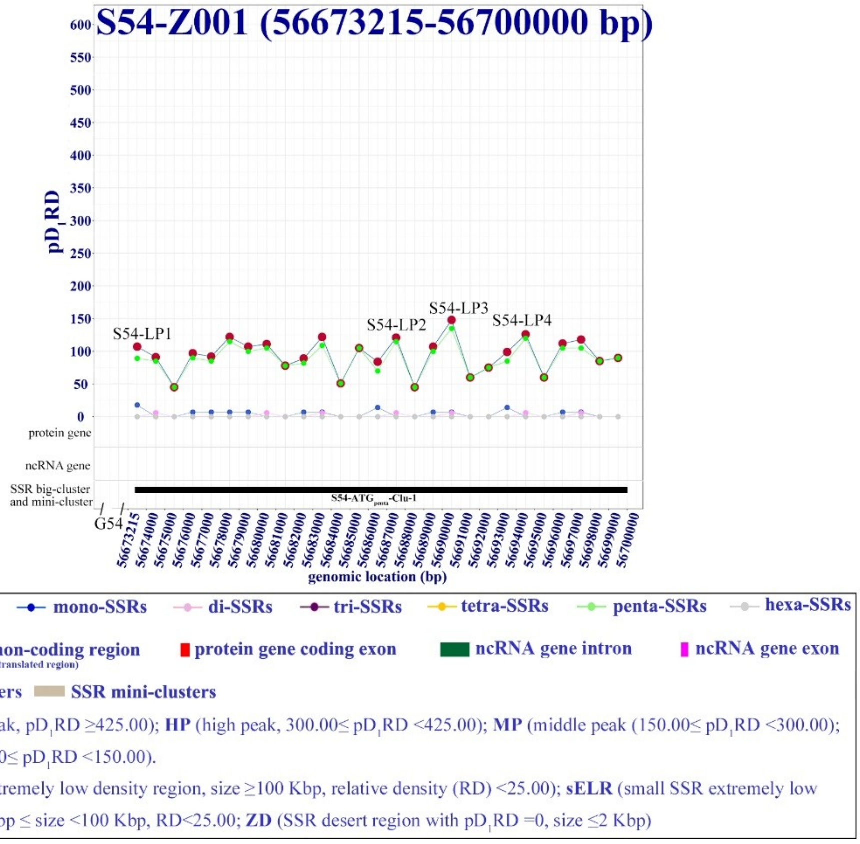

Supplementary Figure 1.529. The SSR position related D_1-relative density (pD_1RD) map of position at 56673215-56700000 bp (unnormal zone <51000 bp) of human reference Y-DNA (NC_000024.10) at resolution of 1 Kbp.

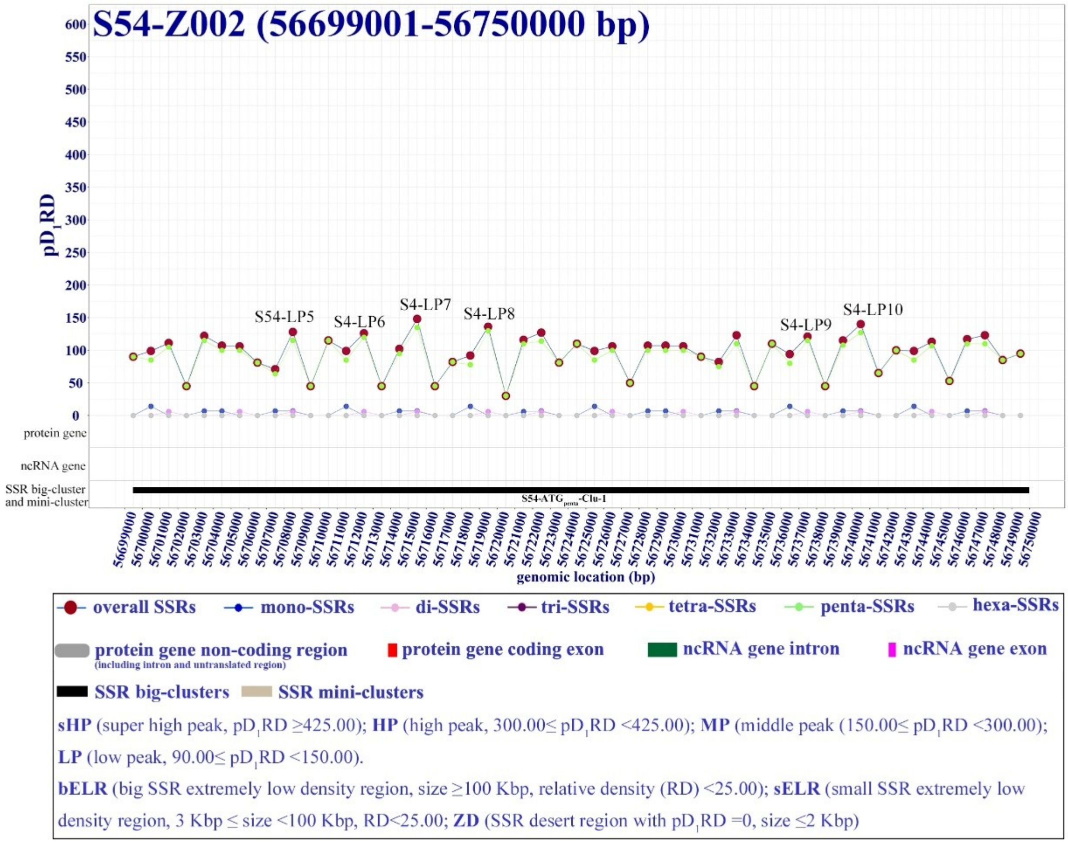

Supplementary Figure 1.530. The SSR position related D_1-relative density (pD_1RD) map of position at 56699001-56750000 bp of human reference Y-DNA (NC_000024.10) at resolution of 1 Kbp.

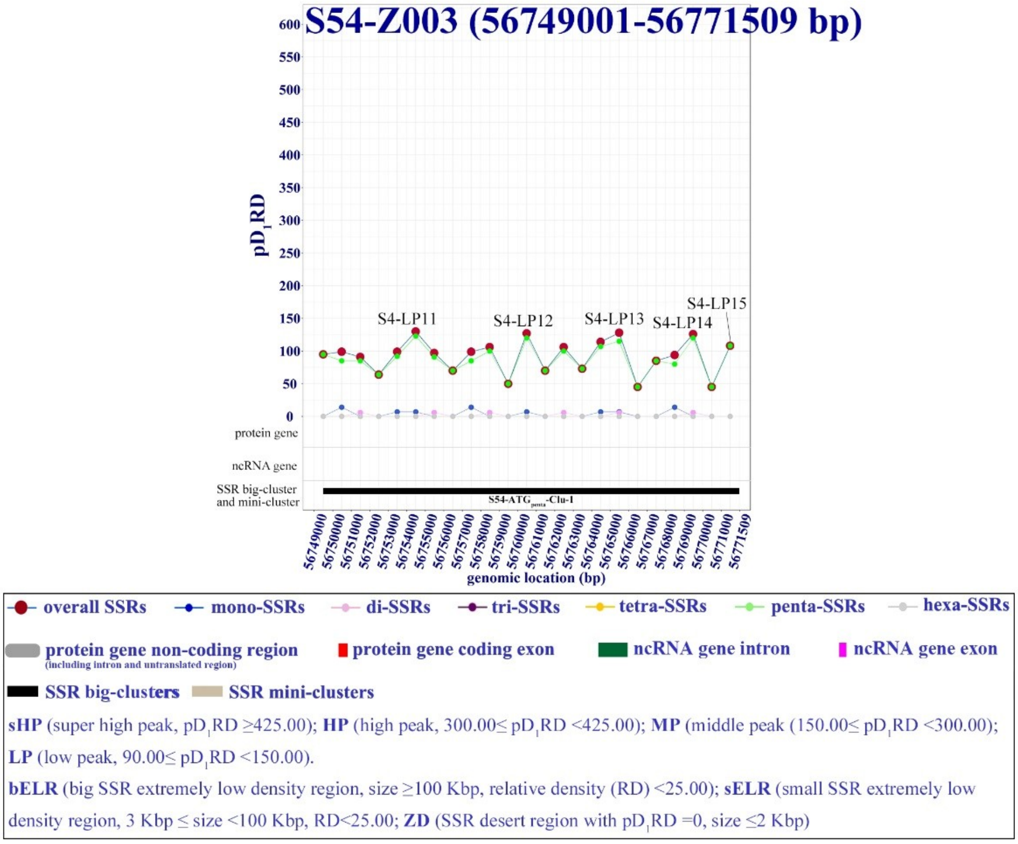

Supplementary Figure 1.531. The SSR position related D_1-relative density (pD_1RD) map of position at 56749001-56771509 bp (unnormal zone <51000 bp) of human reference Y-DNA (NC_000024.10) at resolution of 1 Kbp.

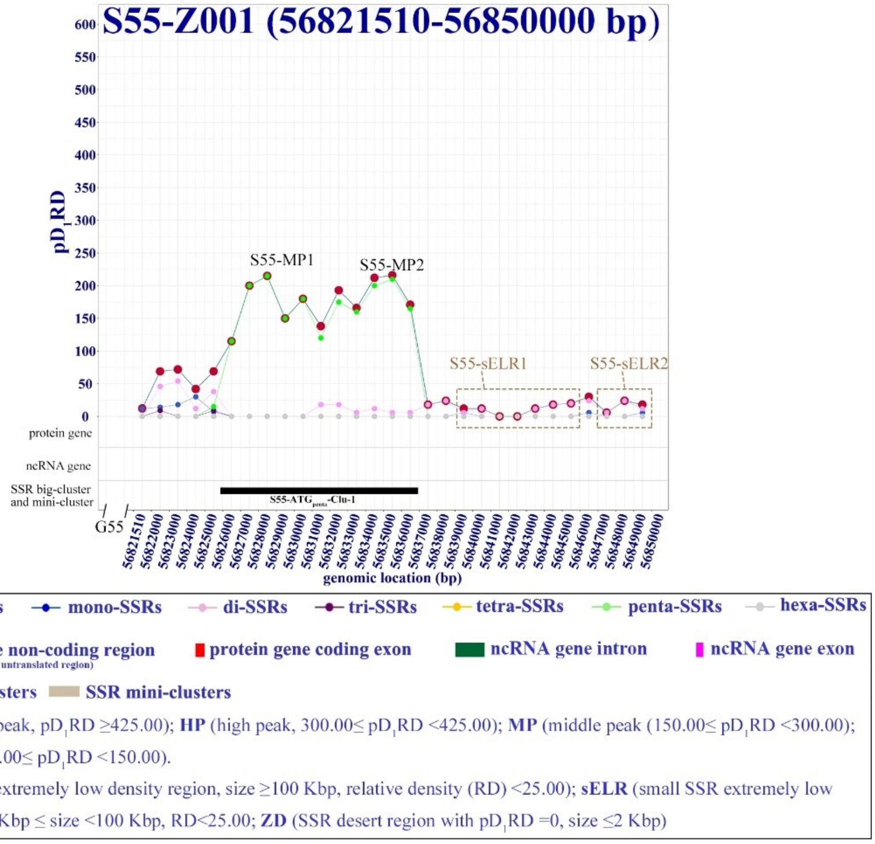

Supplementary Figure 1.532. The SSR position related D_1-relative density (pD_1RD) map of position at 56821510-56850000 bp (unnormal zone <51000 bp) of human reference Y-DNA (NC_000024.10) at resolution of 1 Kbp.

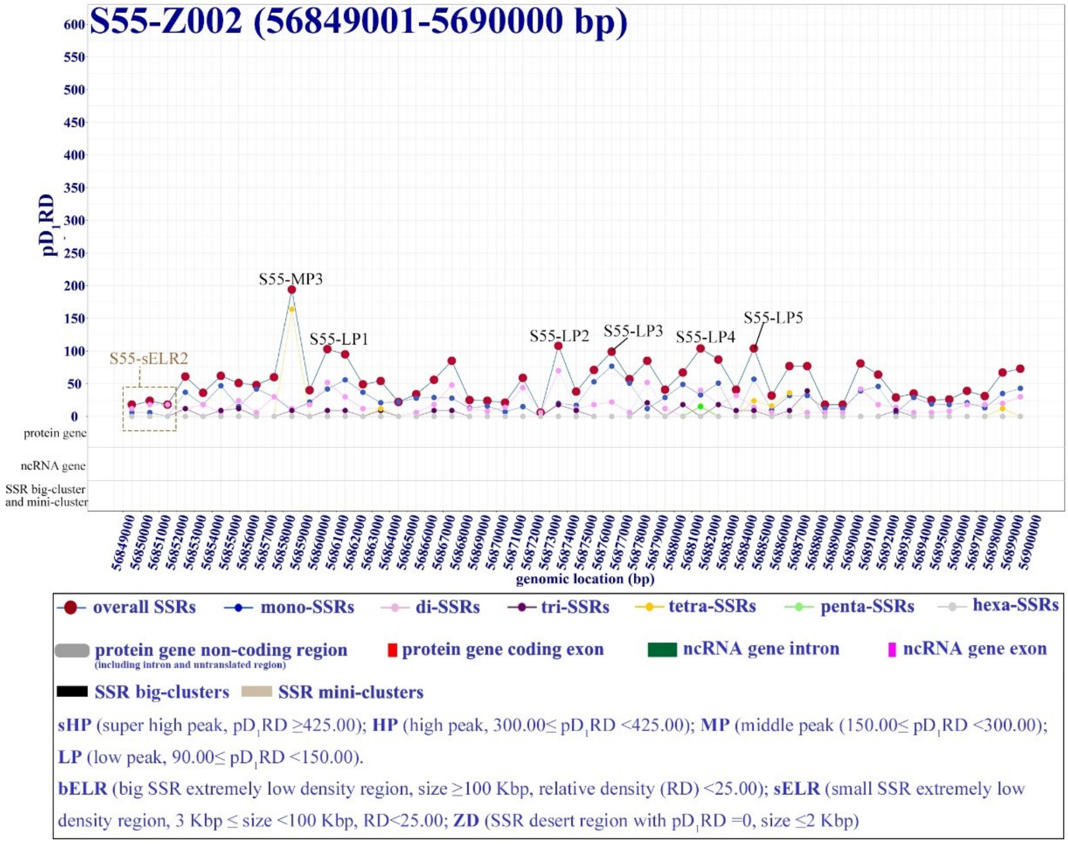

Supplementary Figure 1.533. The SSR position related D_1-relative density (pD_1RD) map of position at 56849001-56900000 bp of human reference Y-DNA (NC_000024.10) at resolution of 1 Kbp.

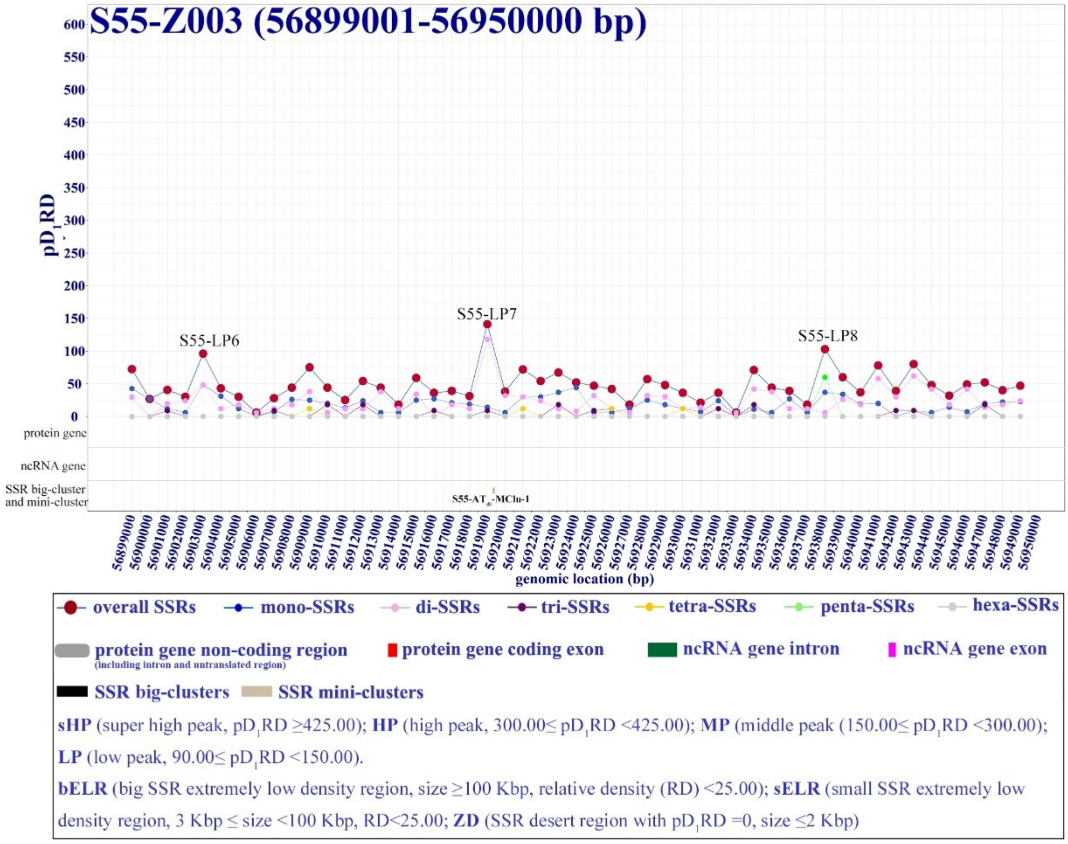

Supplementary Figure 1.534. The SSR position related D_1-relative density (pD_1RD) map of position at 56899001-56950000 bp of human reference Y-DNA (NC_000024.10) at resolution of 1 Kbp.

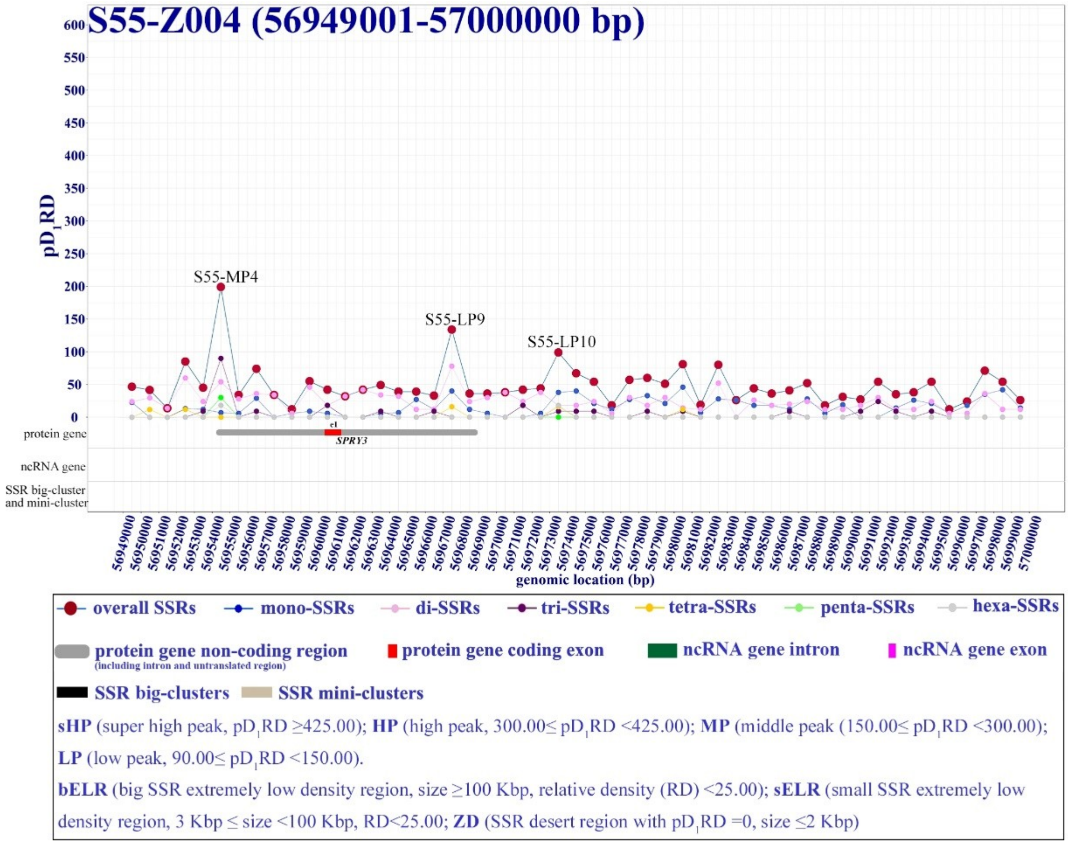

Supplementary Figure 1.535. The SSR position related D_1-relative density (pD_1RD) map of position at 56949001-57000000 bp of human reference Y-DNA (NC_000024.10) at resolution of 1 Kbp.

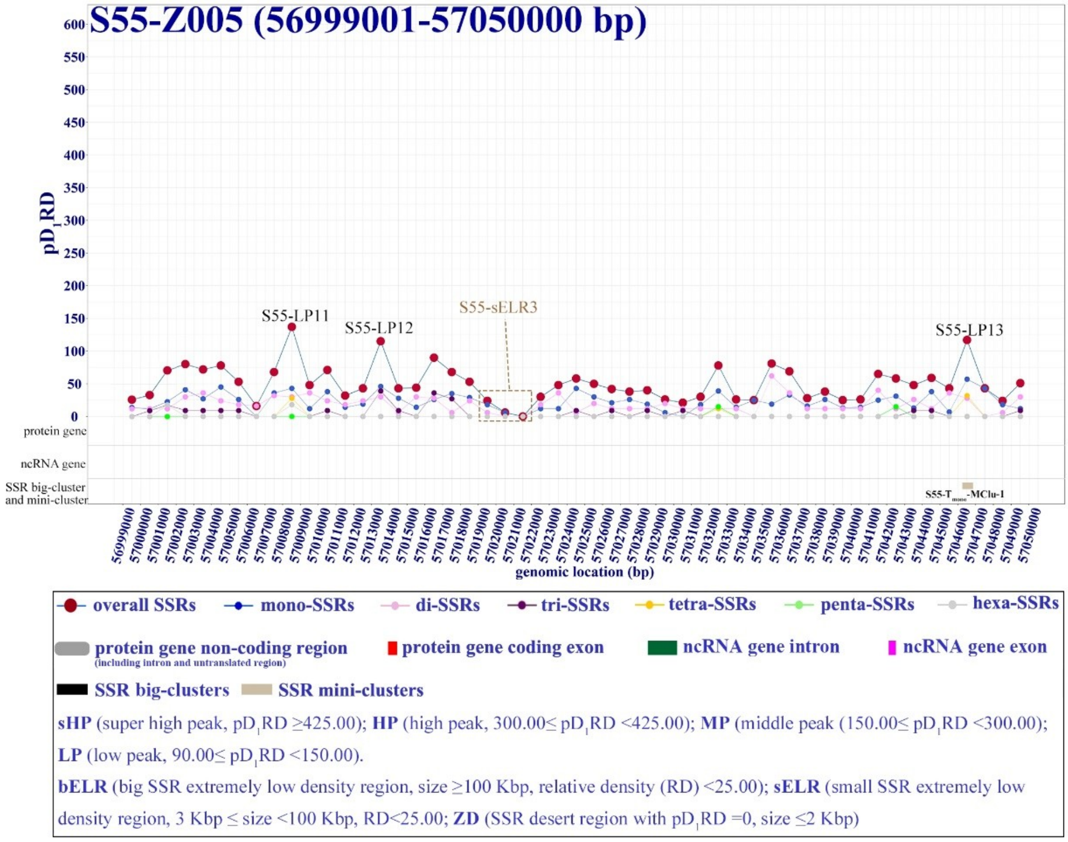

Supplementary Figure 1.536. The SSR position related D_1-relative density (pD_1RD) map of position at 56999001-57050000 bp of human reference Y-DNA (NC_000024.10) at resolution of 1 Kbp.

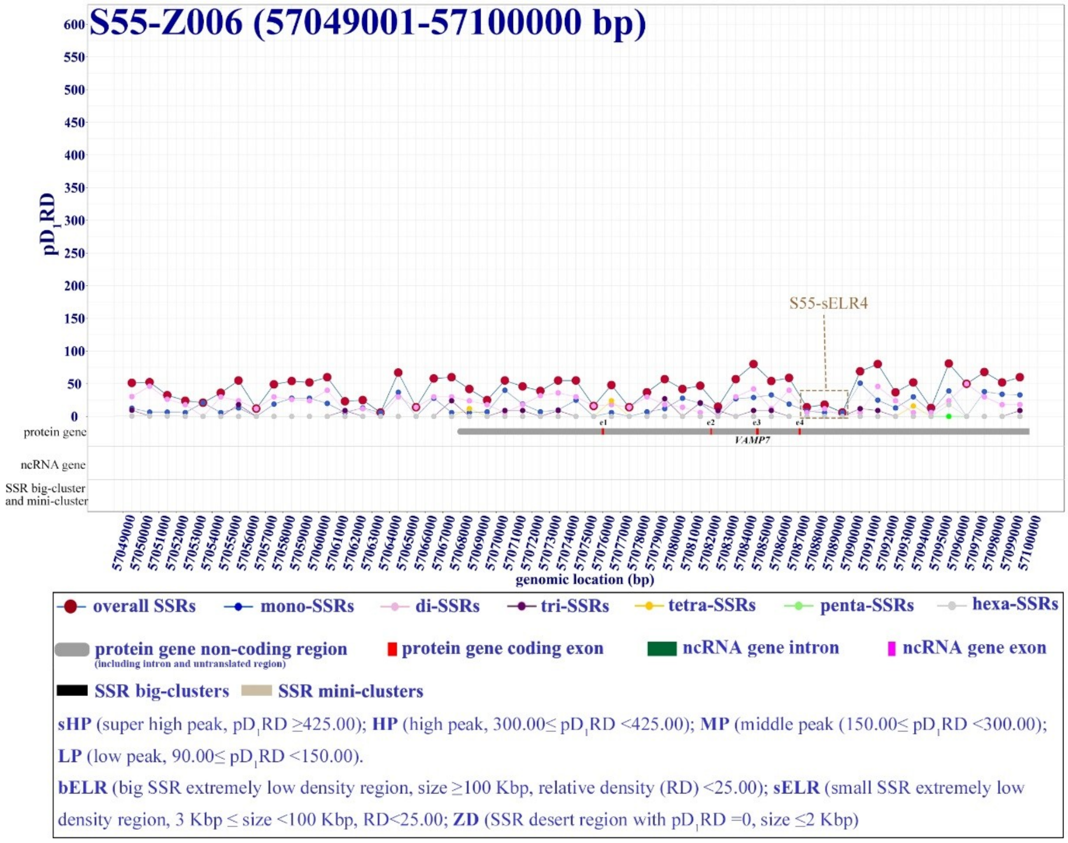

Supplementary Figure 1.537. The SSR position related D_1-relative density (pD_1RD) map of position at 57049001-57100000 bp of human reference Y-DNA (NC_000024.10) at resolution of 1 Kbp.

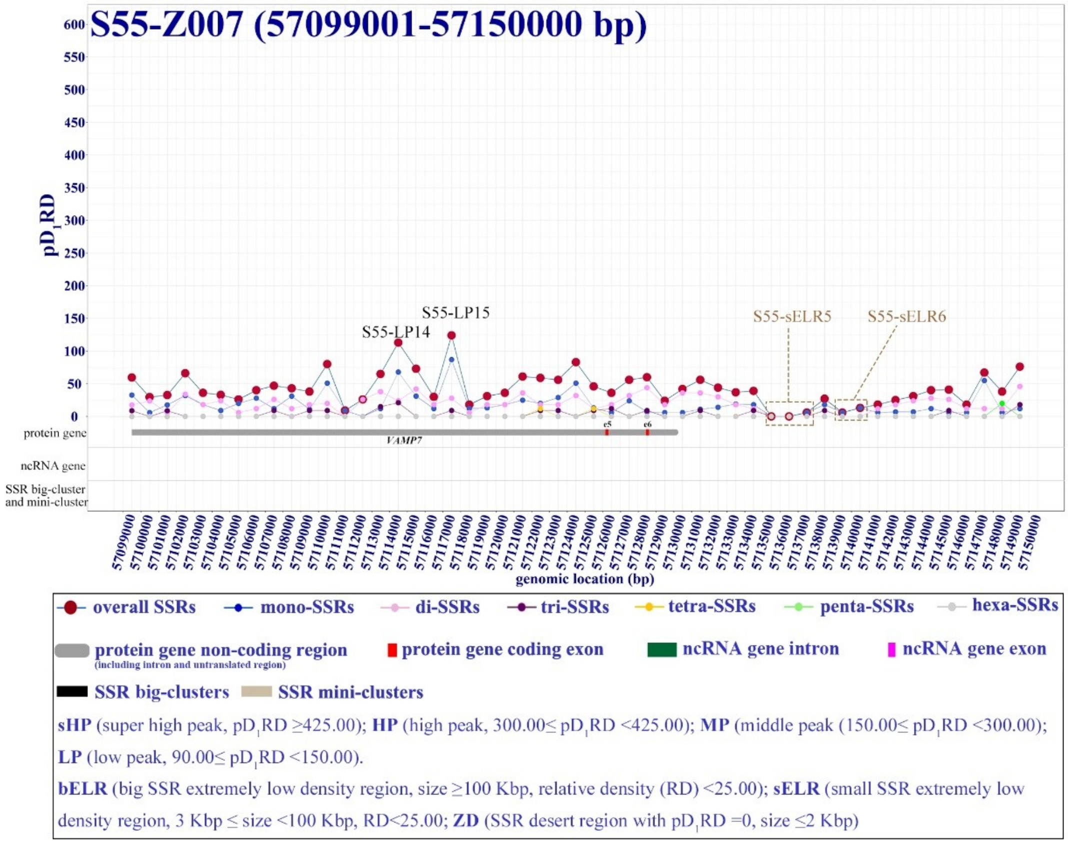

Supplementary Figure 1.538. The SSR position related D_1-relative density (pD_1RD) map of position at 57099001-57150000 bp of human reference Y-DNA (NC_000024.10) at resolution of 1 Kbp.

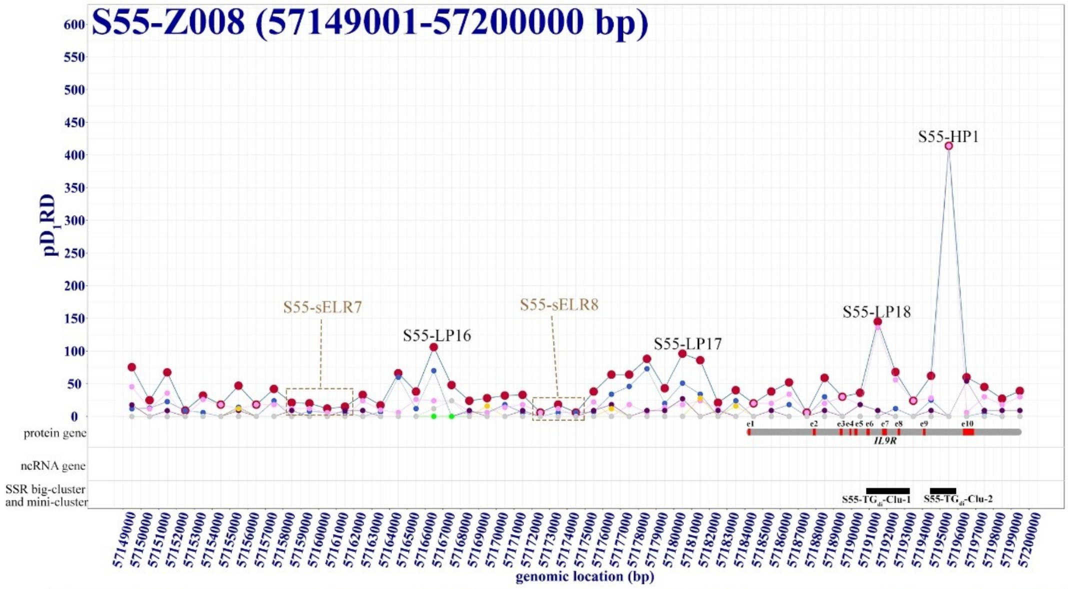

overall SSRs · mono-SSRs · di-SSRs · tri-SSRs · tetra-SSRs · penta-SSRs · hexa-SSRs

protein gene non-coding region (including intron and untranslated region) · protein gene coding exon · ncRNA gene intron · ncRNA gene exon

SSR big-clusters · SSR mini-clusters

sHP (super high peak, $pD_1RD \geq 425.00$); **HP** (high peak, $300.00 \leq pD_1RD < 425.00$); **MP** (middle peak ($150.00 \leq pD_1RD < 300.00$); **LP** (low peak, $90.00 \leq pD_1RD < 150.00$).

bELR (big SSR extremely low density region, size ≥100 Kbp, relative density (RD) <25.00); **sELR** (small SSR extremely low density region, 3 Kbp ≤ size <100 Kbp, RD<25.00; **ZD** (SSR desert region with $pD_1RD = 0$, size ≤2 Kbp)

Supplementary Figure 1.539. The SSR position related D_1-relative density (pD_1RD) map of position at 57149001-57200000 bp of human reference Y-DNA (NC_000024.10) at resolution of 1 Kbp.

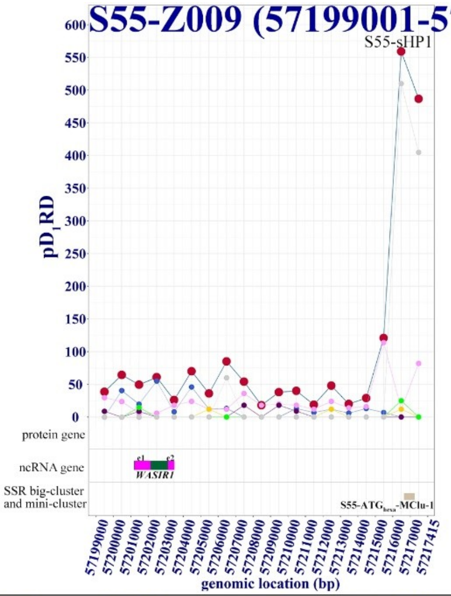

overall SSRs · mono-SSRs · di-SSRs · tri-SSRs · tetra-SSRs · penta-SSRs · hexa-SSRs

protein gene non-coding region (including intron and untranslated region) · protein gene coding exon · ncRNA gene intron · ncRNA gene exon

SSR big-clusters · SSR mini-clusters

sHP (super high peak, $pD_1RD \geq 425.00$); **HP** (high peak, $300.00 \leq pD_1RD < 425.00$); **MP** (middle peak ($150.00 \leq pD_1RD < 300.00$); **LP** (low peak, $90.00 \leq pD_1RD < 150.00$).

bELR (big SSR extremely low density region, size ≥100 Kbp, relative density (RD) <25.00); **sELR** (small SSR extremely low density region, 3 Kbp ≤ size <100 Kbp, RD<25.00; **ZD** (SSR desert region with $pD_1RD = 0$, size ≤2 Kbp)

Supplementary Figure 1.540. The SSR position related D_1-relative density (pD_1RD) map of position at 57199001-57217415 bp (unnormal zone <51000 bp) of human reference Y-DNA (NC_000024.10) at resolution of 1 Kbp.
