# Supplementary figures and images for: A comprehensive microsatellite landscape of human Y-DNA at kilobase resolution
Source: BMC Genomics. 2021 Jan 22;22:76. doi: 10.1186/s12864-021-07389-5 (PMC7821415; doi:10.1186/s12864-021-07389-5)

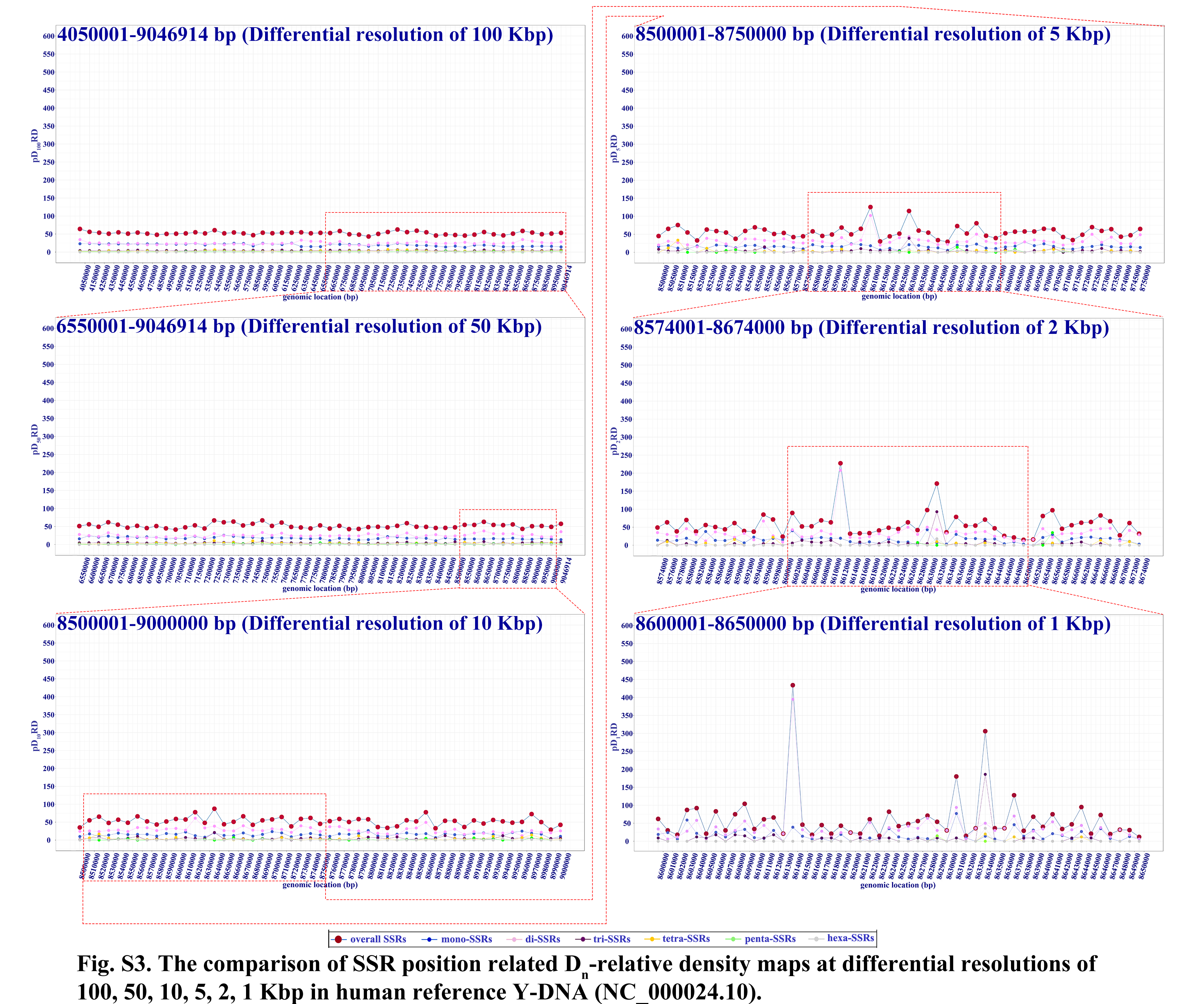

Supplement: Supplementary file 8 — Additional file 8: Figure S3. The comparison of SSR position related Dn-relative density maps at differential resolutions of 100, 50, 10, 5, 2, 1 Kbp in human reference Y-DNA (NC_000024.10). [file 12864_2021_7389_MOESM8_ESM.tiff]

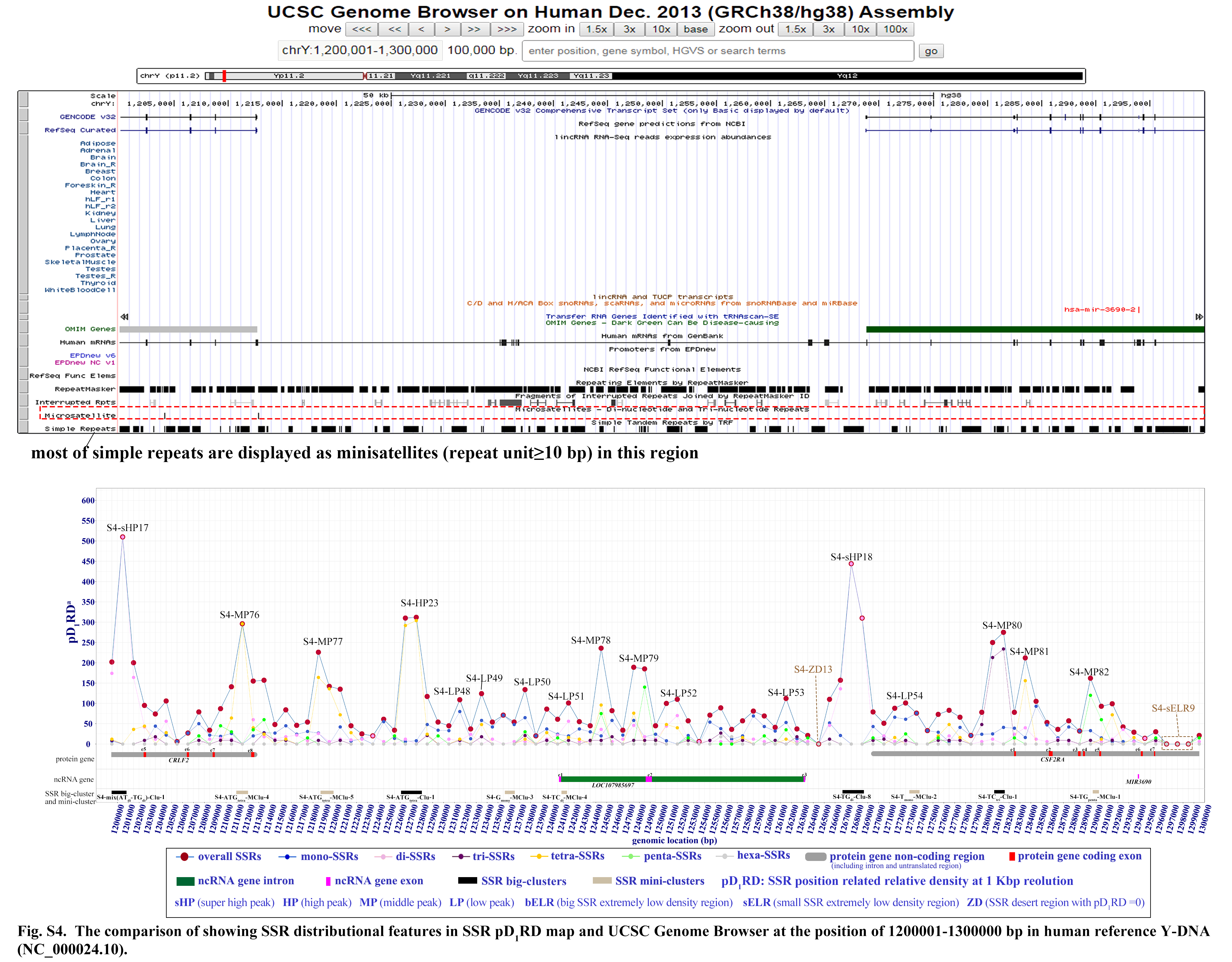

Supplement: Supplementary file 9 — Additional file 9: Figure S4. The comparison of showing SSR distributional features in SSR pD1RD map and UCSC Genome Browser at the position of 1200001-1300000 bp in human reference Y-DNA (NC_000024.10). [file 12864_2021_7389_MOESM9_ESM.tiff]

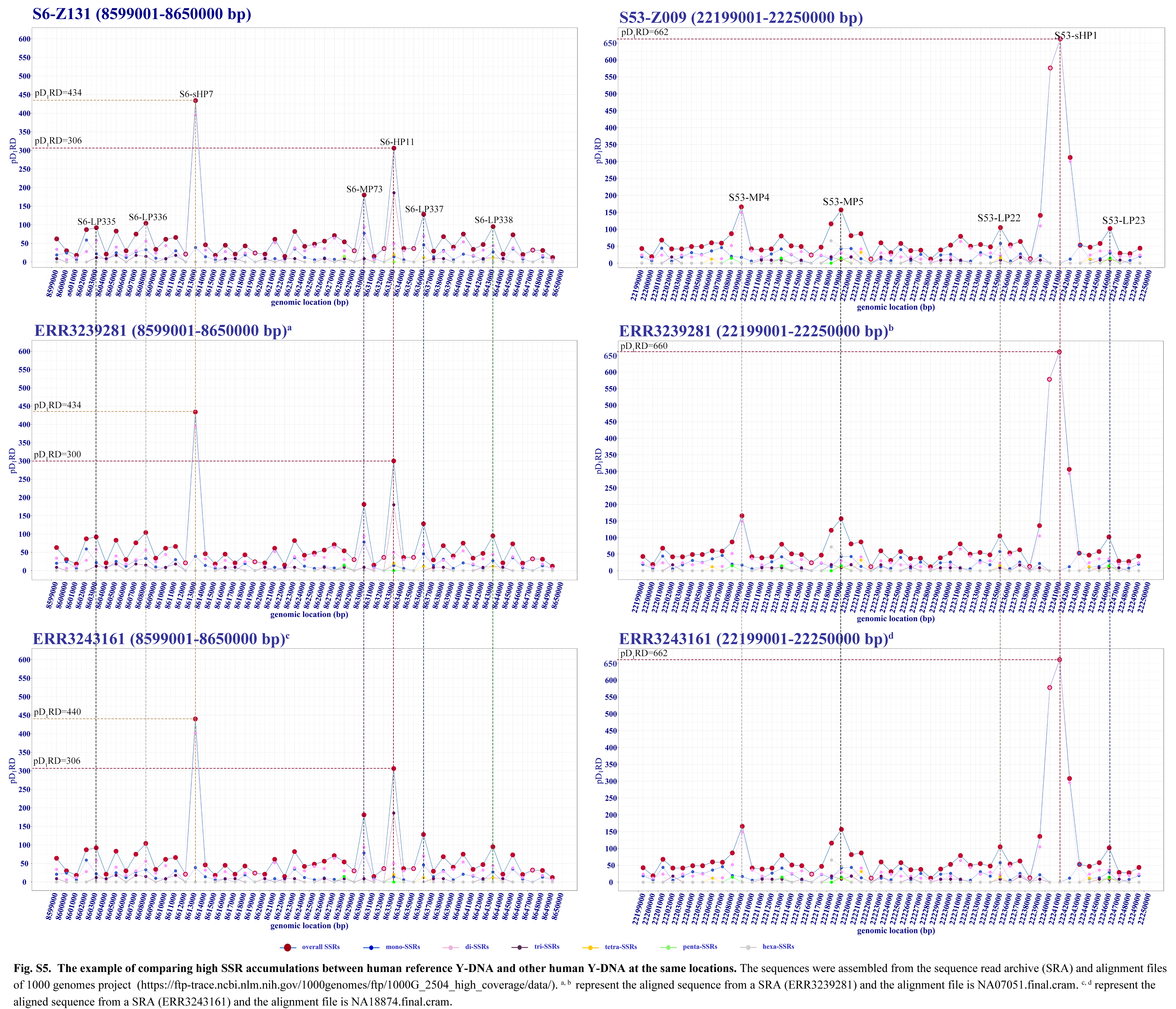

Supplement: Supplementary file 10 — Additional file 10: Figure S5. The example of comparing high SSR accumulations between human reference Y-DNA and other human Y-DNA at the same locations. [file 12864_2021_7389_MOESM10_ESM.tiff]

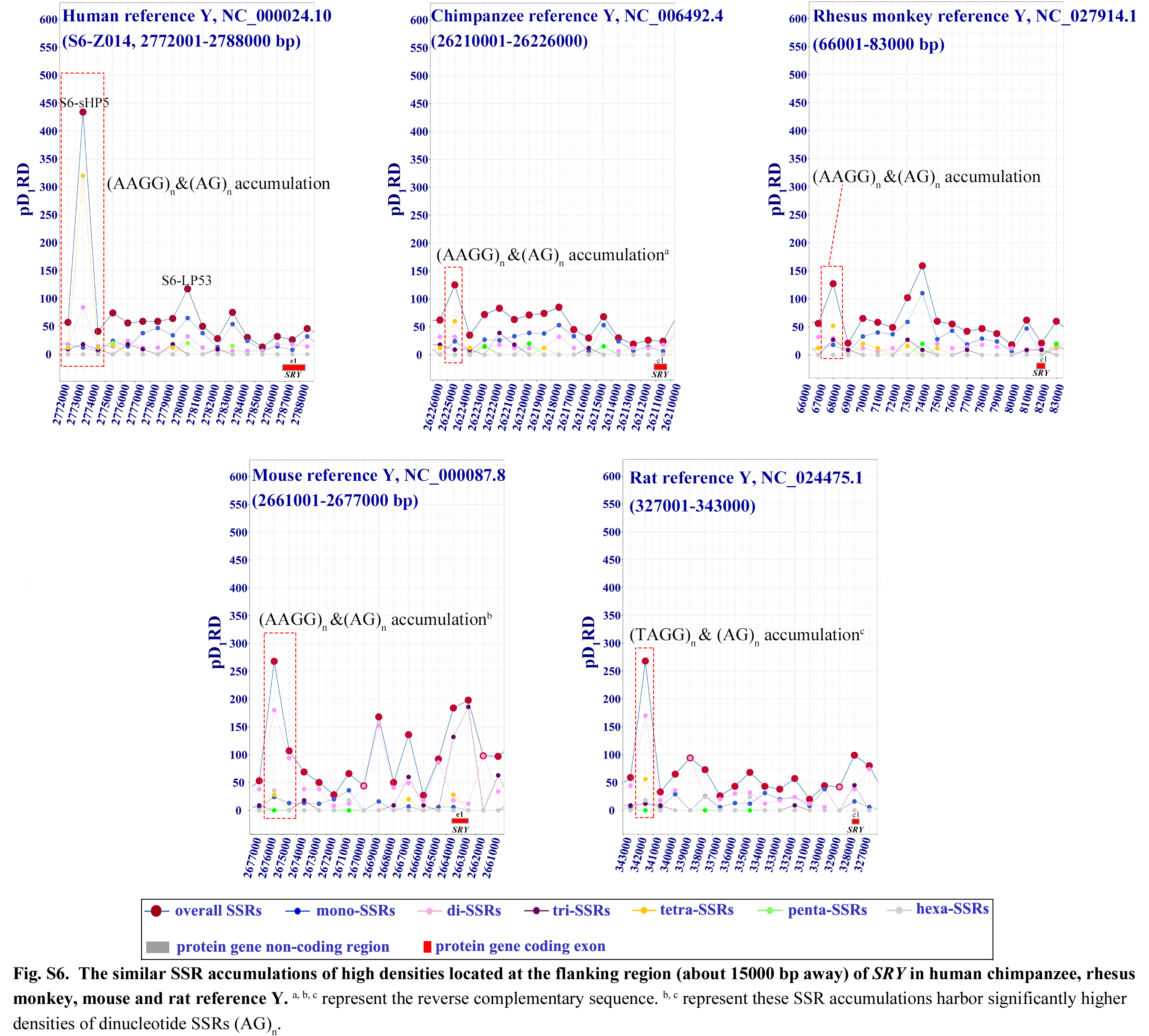

Supplement: Supplementary file 11 — Additional file 11: Figure S6. The similar SSR accumulations of high densities located at the flanking region (about 15000 bp away) of SRY in human chimpanzee, rhesus monkey, mouse and rat reference Y. [file 12864_2021_7389_MOESM11_ESM.tiff]
